# Linking *FOXO3*, *NCOA3*, and *TCF7L2* to Ras pathway phenotypes through a genome-wide forward genetic screen in human colorectal cancer cells

**DOI:** 10.1186/s13073-017-0511-4

**Published:** 2018-01-04

**Authors:** Snehangshu Kundu, Muhammad Akhtar Ali, Niklas Handin, Narendra Padhan, Jimmy Larsson, Maria Karoutsou, Kenneth Ban, Jacek R. Wiśniewski, Per Artursson, Liqun He, Mats Hellström, Tobias Sjöblom

**Affiliations:** 1 Department of Immunology, Science For Life Laboratory, Genetics and Pathology, Rudbeck Laboratory, Dag Hammarskjölds väg 21, Uppsala, 751 85 Sweden; 2 Department of Pharmacy, Uppsala University, Uppsala, 751 23 Sweden; 3 Department of Biochemistry, Yong Loo Lin School of Medicine, National University of Singapore, 8 Medical Drive, #02-06, Singapore, 117597 Republic of Singapore; 4 Institute of Molecular and Cell Biology, A*STAR, Singapore, 138673 Republic of Singapore; 5 Department of Proteomics and Signal Transduction, Biochemical Proteomics Group, Max-Planck Institute of Biochemistry, Martinsried, 82152 Germany; 6 Department of Neurosurgery, Tianjin Medical University General Hospital, Tianjin Neurological Institute, Key Laboratory of Post-Neuroinjury Neuro-Repair and Regeneration in Central Nervous System, Ministry of Education and Tianjin City, Tianjin, 300052 China

**Keywords:** Forward genetics, *piggyBac* transposon, Colorectal cancer, Ras pathway

## Abstract

**Background:**

The Ras pathway genes *KRAS*, *BRAF*, or *ERBBs* have somatic mutations in ~ 60% of human colorectal carcinomas. At present, it is unknown whether the remaining cases lack mutations activating the Ras pathway or whether they have acquired mutations in genes hitherto unknown to belong to the pathway.

**Methods:**

To address the second possibility and extend the compendium of Ras pathway genes, we used genome-wide transposon mutagenesis of two human colorectal cancer cell systems deprived of their activating *KRAS* or *BRAF* allele to identify genes enabling growth in low glucose, a Ras pathway phenotype, when targeted.

**Results:**

Of the 163 recurrently targeted genes in the two different genetic backgrounds, one-third were known cancer genes and one-fifth had links to the EGFR/Ras/MAPK pathway. When compared to cancer genome sequencing datasets, nine genes also mutated in human colorectal cancers were identified. Among these, stable knockdown of *FOXO3*, *NCOA3*, and *TCF7L2* restored growth in low glucose but reduced MEK/MAPK phosphorylation, reduced anchorage-independent growth, and modulated expressions of *GLUT1* and Ras pathway related proteins. Knockdown of *NCOA3* and *FOXO3* significantly decreased the sensitivity to cetuximab of KRAS mutant but not wild-type cells.

**Conclusions:**

This work establishes a proof-of-concept that human cell-based genome-wide forward genetic screens can assign genes to pathways with clinical importance in human colorectal cancer.

**Electronic supplementary material:**

The online version of this article (doi:10.1186/s13073-017-0511-4) contains supplementary material, which is available to authorized users.

## Background

Large-scale mutational analyses are currently unraveling the somatic genetics of human cancer. Apart from mutations in known components of key cancer pathways, such as the Wnt, Ras, and PI3K pathways, moderate to low somatic mutation prevalences have been observed in a large number of genes in colorectal cancers (CRCs) [1]. Concurrently, the fraction of patient tumors where mutations in a given core cancer pathway can be accounted for often amounts to 60% or less, as exemplified by the Ras, PI3K, and TGFB pathways [2]. This conundrum may be due to the importance of yet unknown processes in tumorigenesis, but also to imperfect knowledge of molecular pathways in human cancer cells. Specifically, *KRAS* mutations occur in 35–40% of CRC cases, whereas *BRAF* mutations occur in ~ 10% of cases. Mutations in *KRAS* and *BRAF* are mutually exclusive in CRC, suggesting both confer the same phenotype [3]. In the Ras pathway, K-Ras binds to and activates B-Raf, thereby activating mitogen-activated protein kinase (MAPK) signaling, and oncogenic K-Ras activation enables anchorage-independent growth in vitro [4, 5]. Human CRC cells deprived of their mutant *KRAS* or *BRAF* oncogenes have lost their transcriptional upregulation of the glucose transporter GLUT1 and the associated ability to grow under low-glucose conditions induced by oncogenic Ras pathway activity [6, 7]. Importantly, a subset of clones arising after low-glucose selection of DLD-1 *KRAS*^*wt/-*^ and RKO *BRAF*^*wt/-/-*^ cells had de novo oncogenic mutations in *KRAS* or *BRAF* [6]. This connection between pathway genotype and a distinct phenotype provides a means for classical genetic screens to identify genes in the Ras pathway in CRC. Approaches such as tissue-restricted transposon mutagenesis have identified genes causing CRC and other tumors in transgenic mice [8, 9], some mutated also in human cancers, but only a subset of such experiments provide guidance as to which pathways the genes belong [10]. The *piggyBac* transposon is effective in a wide range of species [11] and one could envision such transposition in human cancer cells with defined somatic mutations to map cancer pathways as activating as well as inactivating mutations can be introduced.

Here, we prove the feasibility of assigning genes to cancer pathways by genome-wide forward genetics using genome-edited human cell systems, link *FOXO3*, *NCOA3*, and *TCF7L2* to phenotypes of the EGFR/Ras/MAPK pathway, and implicate *FOXO3* and *NCOA3* in the response to anti-EGFR therapy.

## Methods

### Cell lines and cell culture

DLD-1 and RKO parental cell lines and DLD-1 *KRAS*^*wt/-*^ [12] and RKO *BRAF*^*wt/-/-*^ [6] knock out cell lines were obtained from Horizon Discovery Ltd. All cells were maintained in DMEM (Invitrogen) medium supplemented with 10% fetal bovine serum and 1% penicillin-streptomycin (Invitrogen) at 37 °C in 5% CO_2._

### Genome-wide *piggyBac* transposon mutagenesis and selection for transposon-mediated low-glucose tolerance

A codon optimized hyperactive *piggyBac* transposase construct (*HyPBase*) [13], gene trap transposons (PB-GT) in three reading frames, and a promoter containing transposon (PB-CAG-SD) were generated (Additional file 1: Figure S2A–C). Five million DLD-1 *KRAS*^*wt/-*^ or RKO *BRAF*^*wt/-/-*^ cells were used in lipofectamine-mediated transfection of *piggyBac* with 12 μg each of transposase and transposon constructs. After 48 h of post-transfection incubation in DMEM, low-glucose selection medium (0.4 mM glucose in DMEM) was applied for approximately three weeks [6]. Next, surviving clones were further selected in regular DMEM with hygromycin (Gibco, 0.2 mg/mL) for 12 days. Clones surviving hygromycin selection were either individually picked or pooled. Trypan Blue staining was used to facilitate clone counting under a microscope.

### Quantification of GLUT1 expression

Total RNA was isolated with the QIAamp RNA Blood Mini Kit (Qiagen) and treated with DNase I (Roche). First strand complementary DNA (cDNA) synthesis was carried out with the RevertAid H-minus First Strand cDNA synthesis kit (Fermentas). The quantitative polymerase chain reactions (qPCRs) were set up with Maxima SYBR Green/ROX qPCR Master Mix (2X) SYBR green (Thermo Scientific). For *GLUT1* transcript level quantification, qPCRs (fwd 5′-GTC ACC ATC CTG GAG CTG TT-3′, rev 5′-GAA GGC CGT GTT GAC GAT AC-3′) were performed in triplicate with β-actin (fwd 5′-CCAACCGCGAGAAGATGA-3′, rev 5′-TCCATCACGATGCCAGTG-3′) as reference gene.

### Splinkerette PCR and sequencing of integration sites

Genomic DNA was isolated from clone pools with the Nucleospin genomic extraction kit (MACHEREY NAGEL). Splinkerette PCR was performed as described [14]. Briefly, genomic DNA was digested with Sau3AI and ligated to adaptors and a primary PCR reaction was performed with a Splink1- 5′-CGAAGAGTAACCGTTGCTAGGAGAGACC-3′ and HMsp1-5′- CGAAGAGTAACCGTTGCTAGGAGAGACC-3′ [15] primer pair. Secondary PCR was carried out with barcoded primers (Additional file 1: Table S6) and the products were treated with ExoSap. The barcoded samples were mixed in equimolar concentrations followed by Illumina sequencing.

### Sequence processing and identification of genes with transposon integration sites

The sequenced transposon-genome junction fragments were first trimmed of 3′ bases with quality value below QV30. The initial six bases of the fragments were aligned to barcode reference sequences (maximum one mismatch allowed) and the pair-end reads, which had identical barcodes identified on both fragments, were assigned to the corresponding samples. To identify the 3′piggyBac inverted terminal Repeats (3′ PB) and splinkerette adaptor (SP) on the sequenced fragments, both sequences were aligned on the fragments to find the adaptor locations. The fragments with correct PB adaptor location and orientation having junctional TTAA sequences were selected for downstream analysis. The trimmed fragments were then aligned to the human genome (hg19) using Smalt software (http://www.sanger.ac.uk/science/tools/smalt-0, version 0.7.5.1, with default parameters). The mapping results were parsed to identify the chromosomal locations of transposon integrations, together with the number of fragments supporting each integration. To remove possible artifacts, integrations sites found in multiple barcode libraries were only assigned to the library with the highest fragment count if they had more than tenfold higher fragment numbers than all the other libraries; integration sites that did not fit these criteria were ignored. Activating (PB-CAG-SD) transposon integrations occurring within a gene or 5 kb upstream of the protein-coding sequences were assigned to the gene regardless of strand or direction of integration. Similarly, inactivating (PB-GT) transposons occurring in introns of a gene were assigned to the gene. As integration of promoter-containing as well as inactivating transposon constructs can, in principle, result in either inactivation or activation of gene function, we regarded them as identical for the purpose of identifying targeted genes. Poisson statistics were applied to each gene to identify the gene candidates with a significant number of integration sites [16], whereas the detection of multiple independent integration sites in both DLD-1 *KRAS*^*wt/-*^ and RKO *BRAF*^*wt/-/-*^ libraries was used as criterium for the selection of a set of recurrently targeted genes. The sequencing data were processed in *R* (version 3.0.1) with several analysis packages (stringr, version 0.6.2; Biostrings, version 2.28.0; Rsamtools, version 1.12.4; BSgenome. Hsapiens. UCSC.hg19, version 1.3.19; plyr, version 1.8, fdrtool, version1.2.11). The raw fastq files from the sequencing were deposited at NCBI Sequence Read Archive (SRA) with BioProject accession number PRJNA419878.

### Pathway analyses and intersections with cancer genome datasets

The genes with significant transposon integrations in both DLD-1 *KRAS*^*wt/-*^ and *BRAF*^*wt/-/-*^ cells were subjected to pathway analysis using KEGG profile (version 1.2.0) with KEGG database version 2.9.1. For a curated pathway analysis, KEGG Pathways and PubMed papers (last accessed 30 January 2015) were searched for the 163 genes with recurring integrations in both DLD-1 *KRAS*^*wt/-*^ and RKO *BRAF*^*wt/-/-*^ to support: (1) their cancer gene status, defined as subject to somatic mutations in several patients with any human cancer; (2) their assignment to a known CRC pathway; or (3) their role in intracellular glucose metabolism. Mutual exclusivity to *KRAS* or *BRAF* mutation in the TCGA COAD dataset [2] was analyzed in cBIOPORTAL (www.cbioportal.org). Non-synonymous somatic mutations, copy number alterations from GISTIC, and messenger RNA (mRNA) expression Z-scores were considered and the strength of the mutual exclusivity relationship was determined by the odds ratios along with *P* values from Fisher’s exact test [10]. To generate control gene lists for the literature-based pathway assignment, random samples of 163 genes were drawn from the 32,746 Ensembl genes using the *sample* function in *R*. Mutation prevalences were obtained from in cBIOPORTAL (www.cbioportal.org) considering copy number aberrations (CNA) and point mutations in the TCGA COAD dataset [2]. The three validated genes were analyzed in the Candidate Cancer Gene Database (CCGD; http://ccgd-starrlab.oit.umn.edu/about.php), a collection of transposon-based forward genetics studies in the mouse [17].

### Validation of target genes by small interfering RNA (siRNA)-mediated knockdown assays

Transient gene knockdown assays were performed by seeding 5000 DLD-1 cells in each well of a 96-well plate followed by siRNA transfections using 100 nM siRNAs (ON TARGET Plus SMART pool siRNAs, GE Healthcare) (Additional file 1: Table S7) and DharmaFECT 2. Next, cDNAs were prepared directly from cell lysates using the Cell-to-Ct kit (Life Technologies). Knockdown efficiency was measured by real-time PCR (RT-PCR) using transcript specific TaqMan assays (Additional file 1: Table S7).

### Generation of stable knockdown cell lines by lentiviral transduction of short hairpin RNAs (shRNAs)

Lentiviral transductions were performed with GIPZ lentiviral particles (GE Healthcare) (Additional file 1: Table S8). The day before transduction, 50,000 cells were plated in each well of a 24-well plate. Viruses were diluted in 250 μL of normal growth medium with 7.5 mg/mL Sequa-Brene per well. The plating medium was removed and 250 μL of diluted virus was added to each well. After 24 h of incubation at 37 °C, virus containing media were replaced with fresh medium. After 48 h of incubation, the cells were FACS sorted for high green fluorescent protein (GFP) and the pool of cells containing the shRNA for a gene of interest as well as with high GFP were maintained in Puromycin selection (Gibco, 1 μg/mL). After expanding for 2–3 passages, knockdown efficiencies were determined by qPCR.

### Clonogenic survival assays

Two thousand cells were plated in each well of a six-well plate and grown in DMEM supplemented with 10% fetal bovine serum and 1% penicillin-streptomycin (Invitrogen) at 37 °C in 5% CO_2_ for ten days_._ The cells were stained in Methylene Blue and scanned in an Epson Perfection V700 photo scanner.

### Real-time growth assays

For the real-time growth assays in low-glucose (0.4 mM) and normal glucose (25 mM) DMEM (Invitrogen) tissue culture medium, 30,000 and 15,000 cells were plated in each well of a 24-well plate, respectively. The plates were kept in an IncuCyte instrument placed inside a tissue culture incubator and the cell confluence monitored at indicated time points.

### Anchorage-independent growth assays

Five thousand cells per well in 0.3% agarose were seeded on top of a 1% agarose layer in a six-well plate. After three weeks of culture at 37 °C and 5% CO_2_, the colonies were stained with Crystal Violet, scanned, and counted using ImageJ v2.0.0-rc-30/1.49 t.

### Determination of MEK and ERK phosphorylation

The protocol was modified from [6]. Cells were lysed in Bicine/CHAPS buffer containing phosphatase and protease inhibitors (ProteinSimple, Santa Clara, CA, USA). The cell lysates were clarified by centrifugation and protein concentration was measured by using BCA Protein Assay Kit (Pierce, Rockford, IL, USA). Lysates were mixed with ampholyte premix (040-972, G2 pH 5-8) and fluorescent pI standards (040-646, pI Standard Ladder 3) before being loaded into the NanoPro 1000 system (ProteinSimple, Santa Clara, CA, USA) for analysis. Isoelectric focusing was performed in capillaries filled with a mixture of cell lysate (0.15 μg/μL protein), fluorescently labeled pI standards, and ampholytes. The separated proteins were cross-linked onto the capillary wall by ultraviolet irradiation followed by immunoprobing with antibodies to ERK (Cell Signaling Technology, #9102, 1:50), pERK (Cell Signaling Technology, #4377, 1:50), MEK (sc-436, Santa Cruz, 1:100), or pMEK (abcam, ab32088, 1:40). Horse Radish Peroxidase conjugated secondary antibodies were from Jackson ImmunoResearch (Donkey Anti-Rabbit IgG, #711-035-152). The signal was visualized by ECL and captured by a charge-coupled device camera. The digital images were analyzed and peak areas quantified with Compass software (ProteinSimple, Santa Clara, CA, USA).

### Sample preparation for proteomic analyses

Cell pellets with 10^6^ cells were lysed in 0.1 M Tris-HCl with pH 7.8, 2% sodium dodecyl sulfate, and 0.05 M dithiothreitol for 5 min at 100 °C. The lysates were sonicated with a Branson-rod-type and then centrifuged at 16100 × *g* for 10 min to clarify the lysates. Samples were then processed in 30-kDa ultrafiltration units with the MED-FASP [18], using Lys-C and trypsin. Concentrations of proteins and peptides were measured with the tryptophan fluorescence assay [19].

### Liquid chromatography-tandem mass spectrometry

Aliquots containing 5 μg of total peptide were chromatographed on a 50-cm column with 75-μm inner diameter packed C18 material. Peptide separation was carried out at 300 nL/min for 75 min using a two-step acetonitrile gradient of 5–40% over the first 60 min and 40–95% for the following 15 min. The temperature of the column oven was 55 °C. Peptide mixtures were then analyzed using a QExactive HF mass spectrometer (Thermo-Fisher Scientific, Palo Alto, CA, USA) in data-dependent mode with survey scans acquired at a resolution of 50,000 at m/z 400 (transient time = 256 ms). Up to the top 15 most abundant isotope patterns with charge ≥ + 2 from the survey scan (300–1650 m/z) were selected with an isolation window of 1.6 m/z and fragmented by HCD with normalized collision energies of 25. The maximum ion injection times for the survey scan and the MS/MS scans were 20 and 60 ms, respectively. The ion target value for MS1 and MS2 scan modes was set to 3 × 10^6^ and 10^5^, respectively. The dynamic exclusion was 25 s and 10 ppm. The MS data were analyzed using MaxQuant software (version 1.5.3.14). Proteins were identified by searching MS and MS/MS data of peptides with a fragment ion mass tolerance of 0.5 Da and parent ion tolerance of 20 ppm against a decoy version of the UniProtKB (August 2015) containing 50,807 sequences. The protein and peptide false discovery rates (FDRs) were set to 1%. Protein abundances were calculated using the “total protein approach” (TPA) method [20]. A two-tailed t-test was performed on the extracted gene products for each cell line. The calculations were performed in Microsoft Excel.

### Proteomic analysis

The quantified proteins were imported to Perseus [21], version 1.5.5.3. Proteins/protein groups with three or more Razor + unique peptides were extracted and used in the downstream analysis. Missing data were imputed with default settings in Perseus. Genes from pathways relevant to either RAS or glucose metabolism were collected from the ConsensusPathDB version 31 [22, 23] from the COAD dataset mentioned above and from the gene family *RAB* (*RAS oncogene GTPases*) and *RABL* (*RAB like GTPases*) from HGNC [24]. These genes were extracted from the dataset of quantified proteins and subjected to a two-sample two-tailed test within Perseus with the following parameters: Student’s t-test; S0 set to 0.05; permutation-based FDR; FDR set to 0.05; 250 randomizations. Volcano plots with the same parameters were also created in Perseus.

### In vitro response to cetuximab

shRNA knockdown cell lines in DLD-1 and isogenic DLD-1 *KRAS*^*Wt/-*^ cells were seeded at 2 × 10^4^ cells per well in 100 μl of complete medium and incubated overnight at 37 °C and 5% CO_2_. The next day, the culture medium was exchanged for serum-free or complete medium with 20 and 40 μg/mL of Erbitux (cetuximab) (Merck KGaA, Germany) followed by incubation for 4–5 days at 37 °C and 5% CO_2_. Cell viability was determined by incubation with Alamar Blue for 4 h followed by counting in a Victor^2^™ 1420 Multilabel counter (Wallac). The viability of treated samples was normalized to that of the untreated samples.

### Candidate Cancer Gene Database (CCGD) analysis

Three validated genes (i.e. FOXO3, NCOA3, and TCF7L2) were analyzed in the CCGD (http://ccgd-starrlab.oit.umn.edu/about.php), a database of collections of transposon-based forward genetics studies [17].

### Molecular network analysis

The three validated genes (*NCOA3*, *FOXO3*, and *TCF7L2*) were subjected to network analysis with the gene set “Ras-Raf-MEK-Erk/JNK signaling” in cBIOPORTAL (www.cbioportal.org) in the COAD dataset. Copy number aberrations, nucleotide level mutations, and mRNA expression patterns were included in the analysis.

### Statistical analysis

All statistical analyses were performed using GraphPad Prism software version 6.0 f. All Students’ t-tests were two-tailed.

### Biosafety declaration

The Swedish work environment authority approved the work with genetically modified and replication deficient lentiviral particles (Arbetsmiljöverket ID 202100-2932 v72). All the experiments with lentiviral particles were conducted under Biosafety Level 2.

## Results

### Genome-wide *piggyBac* forward genetic screen for genes restoring Ras pathway activity

**Fig. 1:**
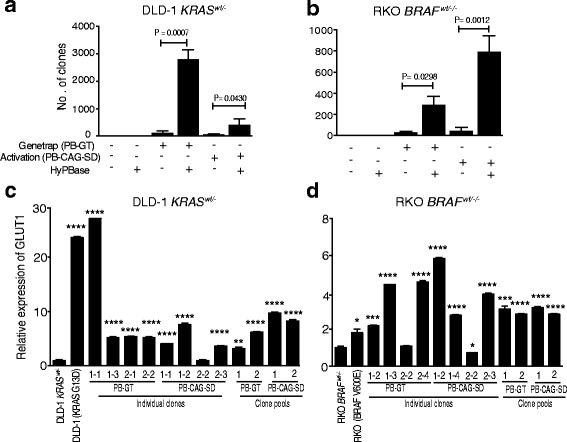
A *piggyBac* transposon screen in human CRC cells identifies putative Ras pathway genes. **a**, **b** Gene trap as well as promoter-containing *piggyBac* transposons can revert human CRC cells deprived of their oncogenic Ras pathway allele to survive growth in low glucose. Co-transfection of DLD-1 *KRAS*^*wt/-*^ and RKO *BRAF*^*wt/-/-*^ cells with transposase (HyPBase) and gene trapping (PB-GT) or promoter-containing (PB-CAG-SD) transposon constructs was followed by selection in DMEM with 0.4 mM L-glucose for three weeks. Clones emerging after glucose deprivation were selected in hygromycin for 12 days to enrich clones with productive transposon integrations (in-frame gene trap or promoter insertion) and the surviving clones were stained and counted. Mean and standard deviation (SD) from three independent experiments. **c**, **d** Transposon mutagenesis and selection in low glucose results in upregulation of GLUT1, a phenotype of Ras pathway activation in human CRC cells. RT-PCR of GLUT1 in parental DLD-1 and RKO cells, their isogenic derivatives DLD-1 *KRAS*^*wt/-*^ and RKO *BRAF*^*wt/-/-*^ with single clones and clone pools derived from mutagenesis with gene trap (PB-GT) or promoter-containing (PB-CAG-SD) transposons. Mean RQs and ΔCt SEs as fold of DLD-1 *KRAS*^*wt/-*^ and RKO *BRAF*^*wt/-/-*^ from three replicates. The *P* < 0.05 values were calculated using Student’s t-test where ****P < 0.0001, ***P < 0.001, **P < 0.01, *P < 0.05

To discover genes in the Ras pathway, we transposon mutagenized human CRC cells having had their active *KRAS* or *BRAF* oncogene removed by genome editing and used a two-step selection procedure where: (1) tolerance to low glucose was selected by culturing three weeks in medium with 0.4 mM L-glucose; and (2) the resulting clones were selected for having productive transposon integrations by culture in hygromycin-containing medium (Additional file 1: Figure S1). The hygromycin selection serves to eliminate background clones generated by transposon-independent mechanisms, such as those having acquired KRAS and BRAF hotspot mutations through the endogenous MMR deficiency [6]. Next, the gene set with recurring transposon integrations is intersected with genes observed mutated in human CRCs to select candidate Ras pathway genes for experimental validation. Mutagenesis of DLD-1 *KRAS*^*wt/-*^ and RKO *BRAF*^*wt/-/-*^ cell lines with transposase and transposon constructs (Additional file 1: Figure S2A–C) followed by selection in low glucose and hygromycin resulted in sixfold to 22-fold more clones than transposon constructs alone (Fig. 1a and b; Additional file 1: Figure S3), demonstrating that transposon mutagenesis could restore growth in low-glucose medium. The majority of selected clones (13/16) and all clone pools had twfold to sixfold higher *GLUT1* expression, a biomarker for Ras pathway activation in human CRC cells [6] compared to DLD-1 *KRAS*^*wt/-*^ and RKO *BRAF*^*wt/-/-*^ cells (Fig. 1c and d). Two gene trap libraries (PB-GT) of 1500 and 2000 clones each, as well as two individual activation libraries (PB-CAG-SD) of 3000 and 2480 clones each in DLD-1 *KRAS*^*wt/-*^ and RKO *BRAF*^*wt/-/-*^ cells, respectively (Fig. 1c and d), were subjected to sequencing. The clone pools were analyzed by splinkerette PCR amplification followed by sequencing of transposon integration sites, resulting in 2029 genes in DLD-1 *KRAS*^*wt/-*^ and 2887 genes in RKO *BRAF*^*wt/-/-*^ pools having more frequent integrations than expected given their TTAA site density (*P* ≤ 0.05, Poisson distribution) on all the chromosomes (Additional file 1: Figure S4; Additional file 1: Table S1 and S2; Additional file 1: Figure S5).

### Enrichment of cancer genes and Ras pathway genes

The significantly targeted genes included *KRAS* (Additional file 1: Table S2) and *BRAF* (Additional file 1: Table S3). When intersecting the significantly targeted genes in DLD-1 *KRAS*^*wt/-*^ and RKO *BRAF*^*wt/-/-*^ cell systems to pinpoint drivers in both genetic backgrounds, 483 genes were identified (Additional file 1: Table S3) with enrichment in CRC pathways such as Wnt, ErbB, and MAPK (*P* = 5.23 × 10^–5^, 0.0045, and 0.046; Additional file 1: Table S4). Next, we identified 623 and 777 genes having at least two unique integration sites in both DLD-1 *KRAS*^*wt/-*^ and RKO *BRAF*^*wt/-/-*^ libraries. After intersecting these two libraries, 163 genes with at least two integration sites were identified (Additional file 1: Figure S6A; *P* = 1.31 × 10^–90^). A combined literature and pathway analysis showed that: (1) 31% of the 163 genes were recurrently mutated in human cancers (*P* = 8.05 × 10^–47^ under the assumption of 400 cancer genes of 20,000 total protein-encoding genes, hypergeometric distribution); (2) 19% had previously been linked to the EGFR/Ras/MAPK pathway and 6% to the associated Hippo pathway; and (3) 11% had been linked to intracellular glucose metabolism. In comparison, 1–10% of the genes had been associated with other CRC pathways such as the Wnt, TGFB, PIK3CA, or TP53 pathways (Additional file 1: Table S5a). The intersections of these 163 genes with driver genes observed in mutational analyses of human CRC and transposon models of mouse CRC were also larger than expected (Additional file 1: Figure S6B and 6C; *P* = 5.63 × 10^–5^ and *P* = 1.01 × 10^–5^). The 31 genes known to act within the EGFR/Ras/MAPK pathway included the ERBB receptor ligands *NRG1* and *NRG3*, the tumor suppressor microRNA mir-31, *FOXP1*, *PARD3*, *PDE4D*, *PTPRG*, *RGS6*, *TRPS1*, *VMP1*, and components of the glutamate transport system. Several genes in intracellular glucose metabolism were also targeted, such as *PHLDB1*, involved in GLUT translocation to the plasma membrane, *HIPK3*, *KCNMA1*, *PDP1*, *SNAP25*, *TXNRD1*, and *UBR5*, along with *DGKB*, *DIS3L2*, *ENTPD1*, *RUNX2*, and *SIAH1* involved in both Ras signaling and glucose metabolism (Additional file 1: Table S5a). In comparison, three sets of 163 randomly picked genes yielded average 6% cancer genes, 4% EGFR/Ras/MAPK pathway genes, and 0–5% of other CRC pathway genes (Additional file 1: Table S5b–d). Thus, the screen enriches for cancer genes in general and Ras pathway genes in particular.

### Identification of putative Ras pathway genes that regulate GLUT1 expression

While mutational analyses and genetic screens provide indications of cancer gene status, experimental validation is required for ultimate proof. To explore the most fruitful approaches to enrich true positives, 13 genes from the intersection of 163 genes having at least two unique integration sites were selected for functional validation based on: (1) mutual exclusivity with *KRAS* or *BRAF* mutation in the TCGA COAD dataset [2] (Additional file 1: Table S3 and 5a; *FOXO3*, *NCOA3*, *SEMA5A*); (ii) intersection with other human CRC exome datasets (Additional file 1: Table S5a and Additional file 1: Figure S6B; *PTPRD*, *CLSTN2*, *CSMD3*, *NAV3*, *ROBO1*, *TCF7L2*); and (3) intersection with datasets from murine transposon models of CRC [8] (Additional file 1: Table S5a and Additional file 1: Figure S6C; *ROCK2*, *SIPA1L1*, *SND1*, *ZCCHC7*). By transient siRNA knockdown, significant effects on GLUT1 transcript levels were observed for FOXO3, NCOA3, and TCF7L2 (Additional file 1: Figure S7A–D) but not for the other ten genes (Additional file 1: Figure S7E and F).

### Functional validation of the putative Ras pathway genes FOXO3, NCOA3, and TCF7L2 by real-time growth assays in low-glucose medium and GLUT1 expression analysis

**Fig. 2:**
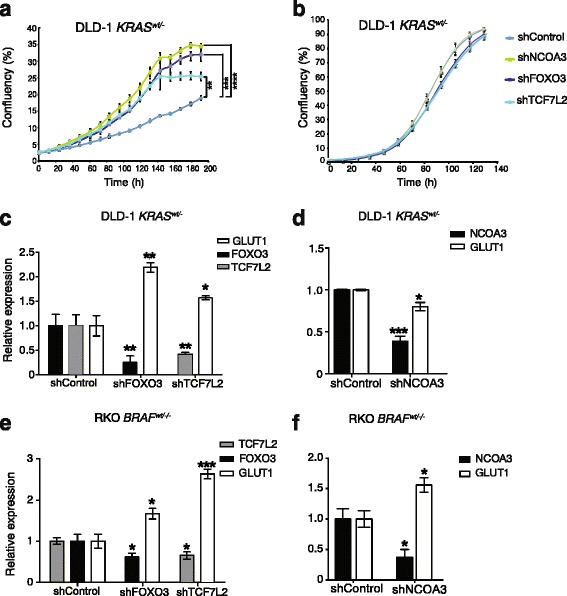
The transposon target genes *FOXO3*, *NCOA3*, and *TCF7L2* modulate the Ras pathway phenotype of growth in low glucose and regulate GLUT1 expression. Real-time growth curves for stable shRNA knockdown lines of *NCOA3*, *FOXO3*, and *TCF7L2* in DLD-1 *KRAS*^*wt/-*^ cells in low-glucose medium (**a**) and normal medium (**b**). GLUT1 expression level after stable knockdown of these three genes in DLD-1 *KRAS*^*wt/-*^ (**c**, **d**) and RKO *BRAF*^*wt/-/-*^ (**e**, **f**). Gene expression levels were measured by qPCR using beta-actin as endogenous control and normalized to shControl. Each experiment was performed three times with three technical replicates. The statistical analysis was performed by Student’s t-test where ****P < 0.0001, ***P < 0.001, **P < 0.01, *P < 0.05

As *FOXO3*, *NCOA3*, and *TCF7L2* were also detected in both transposon data analysis approaches (Additional file 1: Table S3 and 5a) in the gene trap/inactivation library, we generated shRNA-mediated knockdowns in DLD-1 and RKO cells to assess whether these genes were involved in one or several phenotypes associated with the Ras pathway. First, we assessed whether the reduced growth of DLD-1 *KRAS*^*wt/-*^ cells in low-glucose medium could be reverted. Stable knockdown of FOXO3, NCOA3, and TCF7L2 increased growth of DLD-1 *KRAS*^*wt/-*^ cells in low glucose without any growth advantage in normal glucose medium (Fig. 2a and b). The altered growth in low glucose is expected to involve increased GLUT1 expression [6]. In agreement with this postulate, the stable knockdown lines for FOXO3 and TCF7L2 displayed upregulation of GLUT1 in DLD-1 *KRAS*^*wt/-*^ and RKO *BRAF*^*wt/-/-*^ (Fig. 2c and e). Whereas the stable knockdown lines for FOXO3 and TCF7L2 displayed reduced and increased expression of GLUT1 in DLD-1 (KRAS G13D) (Additional file 1: Figure S8A), respectively, stable knockdown of TCF7L2 led to upregulation of GLUT1 in RKO (BRAF V600E) (Additional file 1: Figure S8C) cells. However, stable knockdown of NCOA3 led to upregulation of GLUT1 in RKO *BRAF*^*wt/-/-*^ and DLD-1 (KRAS G13D) cells (Fig. 2f and Additional file 1: Figure S8B, respectively) but not in DLD-1 *KRAS*^*wt/-*^ (Fig. 2d). Stable knockdown of FOXO3, NCOA3, and TCF7L2 in SW48 colorectal cell lines devoid of *KRAS* mutations did not show changes in *GLUT1* expression (Additional file 1: Figure S9A) and the phenotype in low glucose was similar to normal medium (Additional file 1: Figure S9B, C). This demonstrated that these two phenotypes were indeed related to Ras pathway activation and that FOXO3, NCOA3, and TCF7L2 were not directly linked to glucose metabolism. To understand whether these genes could directly regulate GLUT1 transcription, we also used JASPAR (http://jaspar.genereg.net) to determine if binding sites for FOXO3, NCOA3, or TCF7L2 exist in the 3-kb upstream sequence of the initiation site of the GLUT1 gene. While the known positive regulator of GLUT1, HIF1A, had 38 binding sites with a median score of 5.27, this analysis revealed 16 and 13 binding sites for TCF7L2 and FOXO3 with a median score of 5.34 and 5.36, but no direct binding site for NCOA3 (Additional file 1: Table S10). Taken together, stable knockdown of FOXO3, NCOA3, and TCF7L2 in CRC cells deprived of their mutant Ras pathway restored growth in low-glucose medium, which was paralleled by increased GLUT1 expression in the case of FOXO3 and TCF7L2.

### Effects of stable shRNA knockdown of FOXO3, NCOA3, and TCF7L2 on anchorage-independent growth and pERK/MEK levels

**Fig. 3:**
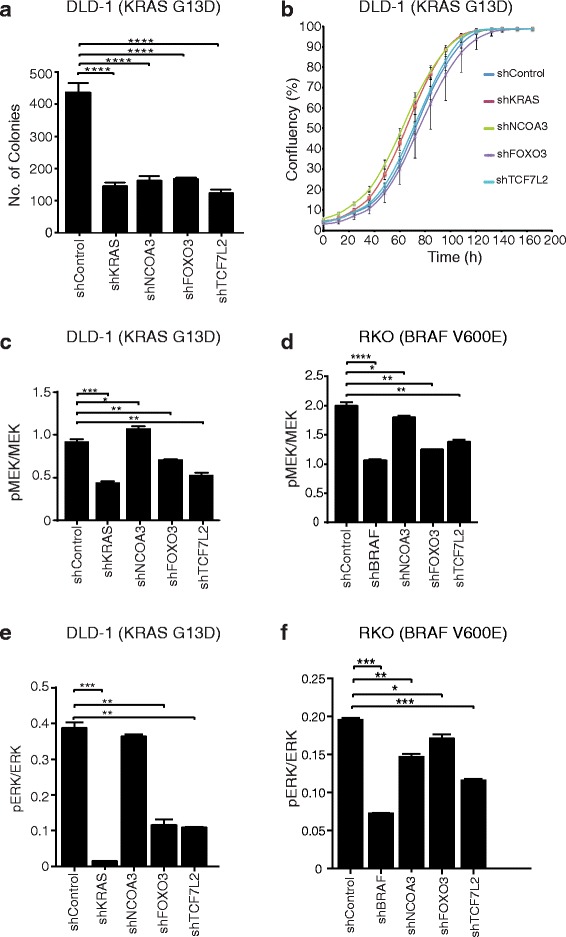
Modulation of the Ras pathway phenotypes of MEK/ERK phosphorylation and anchorage-independent growth by FOXO3, NCOA3, and TCF7L2. Soft agar assays (**a**) and growth curves in normal medium (**b**) in stable shRNA lines of NCOA3*,* FOXO3, and TCF7L2 in DLD-1 cells (KRAS G13D). The impact of NCOA3, FOXO3, and TCF7L2 perturbation on pMEK and pERK in relation to MEK and ERK, respectively, were quantified by NanoPro analysis in DLD-1 (KRAS G13D) (**c**, **e**) and RKO (BRAF V600E) (**d**, **f**) cells with shKRAS and shBRAF as positive controls. Each experiment was performed at least twice with three technical replicates with statistical analysis by Student’s t-test where ****P < 0.0001, ***P < 0.001, **P < 0.01, *P < 0.05

As oncogenic Ras signaling supports anchorage-independent growth in vitro [5, 12, 14], a hallmark of oncogenic transformation, we investigated the effects of FOXO3, TCF7L2, and NCOA3 shRNA knockdown on anchorage-independent growth by colony formation in soft agar. We observed more than twofold reduced anchorage-independent growth in DLD-1 cells with KRAS G13D mutation and activated oncogenic Ras signaling (Fig. 3a and Additional file 1: Figure S10A) while the rates of growth in normal medium and colony formation on plastic were similar (Fig. 3b, c; Additional file 1: Figure S10B). However, the RKO (BRAF V600E) cells did not form colonies in the same soft agar assay. Mutational or expression changes in genes of the EGFR/Ras/MAPK pathway can alter the cellular ratios of phosphorylated MEK to total MEK and of phosphorylated ERK to total ERK [25, 26]. Stable knockdown of FOXO3 or TCF7L2 reduced pMEK and pERK in DLD-1 cells with KRAS G13D mutation and RKO cells with BRAF V600E mutation, to an extent similar to knockdown of KRAS or BRAF (Fig. 3c–f; Additional file 1: Figure S11A, B and Additional file 1: Figure S12A, B). While knockdown of NCOA3 had little effect on pMEK/MEK and no effect on pERK/ERK ratios in DLD-1 cells with KRAS G13D mutation (Fig. 3c and e; Additional file 1: Figure S11A, B), it had significant effect in RKO (BRAF V600E) cells (Fig. 3d and f; Additional file 1: Figure S12A, B). Hence, stable knockdown of FOXO3, NCOA3, and TCF7L2 significantly reduced anchorage-independent growth and MEK/ERK phosphorylation.

### Global expression changes of Ras pathway proteins after knockdown of *NCOA3*, *FOXO3*, and *TCF7L2*

**Fig 4:**
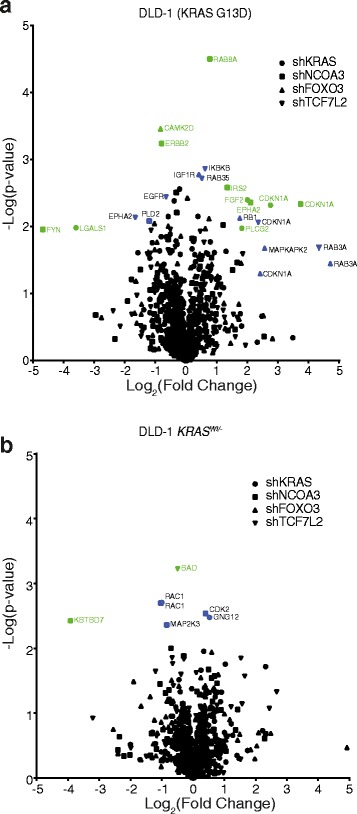
Global proteomic analysis shows expression alterations of Ras-related genes by stable shRNA lines of NCOA3, FOXO3, and TCF7L2. The *volcano plot* depicts the -log(*P* values) vs log_2_(fold change) of Ras-related genes (**a**, **b**) for the stable knockdown genes in relation to a shRNA control. *Colored symbols* indicate genes of interest (display both large fold-change [*x-axis*] and high statistical significance [*y-axis*]). *Light green symbols*, significantly changed (FDR ≤ 0.05); *blue symbols*, not significant (FDR > 0.05). The full list of genes is presented in Additional file 1: Table S9

Next, we reasoned that if a gene had a role in a Ras pathway, perturbation of that gene should change expression levels in proteins associated with the Ras pathway. We therefore performed global proteomic analyses of stable shRNA-mediated knockdown lines of *NCOA3*, *FOXO3*, and *TCF7L2* in both the DLD-1 and isogenic DLD-1 *KRAS*^*wt/-*^ genetic background. A total of 8556 proteins were identified with concentrations covering seven orders of magnitude (Additional file 1: Figure S13), of which 6905 had three or more unique peptides and were kept for downstream analysis. From the pathway analysis, 210 genes associated with the Ras pathway were extracted (Fig. 4a; Additional file 1: Table S9). Notably, CDKN1A (p21/WAF1) was upregulated in all four knockdown lines in mutant KRAS background. Regulation of p21 by Ras through E2F1 has previously been demonstrated [27]. Further, RAB3A was upregulated in NCOA3 and TCF7L2 knockdowns, whereas EPHA2 was upregulated in NCOA3 but downregulated in TCF7L2 knockdowns. In contrast, there were comparatively fewer regulated protein products in DLD-1 *KRAS*^*wt/-*^ cells, with RAC1 downregulation in both KRAS and NCOA3 knockdown lines. Together, knockdown of *NCOA3*, *FOXO3*, and *TCF7L2* modulated Ras pathway-associated proteins primarily in the KRAS-activating mutant background.

### Modulation of cetuximab resistance by NCOA3 and FOXO3

**Fig. 5:**
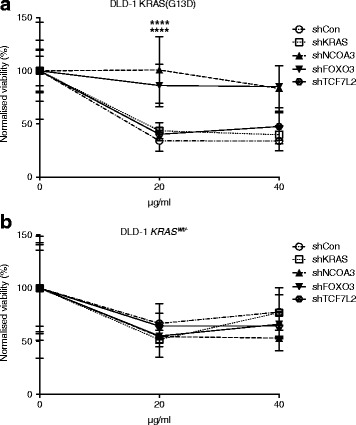
Knockdown of *NCOA3* and *FOXO3* desensitizes DLD-1 cells to cetuximab. The shRNA knockdown lines of *NCOA3*, *FOXO3*, and *TCF7L2* in (**a**) DLD-1 (KRAS G13D) and (**b**) DLD-1 *KRAS*^*wt/-*^ genetic backgrounds were treated with cetuximab (20 μg/mL or 40 μg/mL) for 4–5 days. Each experiment was performed three times with eight technical replicates. Mean percentage of Alamar Blue cell viability was normalized to the mean percentage cell viability of untreated control. Mean percentage of viability and SD. Statistical analysis was performed by Student’s t-test where ****P < 0.0001

The clinical response of CRCs to the EGFR inhibitor cetuximab depends on the KRAS mutation status [28, 29]. To understand whether FOXO3, NCOA3, or TCF7L2 could affect therapy response, we subjected the stable shRNA knockdown cell lines to cetuximab. Knockdown of NCOA3 and FOXO3 caused significantly increased resistance compared to control and KRAS knockdowns (>100% vs 50%) in DLD-1 cells with activating KRAS mutation (Fig. 5a, *P* < 0.001), but not in DLD-1 *KRAS*^*Wt/-*^ cells (Fig. 5b). The knockdown efficiencies of FOXO3 and NCOA3 were 70–80% (Additional file 1: Figure S8A, B; Fig. 2c and d) and the knockdown efficiency of KRAS was 50% (Additional file 1: Figure S15).

## Discussion

While it is now easy to collect genome sequences from large sets of human cancers, it remains challenging to assign infrequently mutated genes as drivers or passengers in cancer pathways without extensive functional studies. Unbiased pathway mapping in human cells has been hampered by a lack of phenotypes amenable to selection. For example, siRNA screens based on growth in soft agar have discriminated driver from passenger genes mutated in CRC but were performed in a background of activating *KRAS* and inactivating *TP53* mutations and could not assign genes to specific pathways [30]. Here, for the primary screen we exploited previous observations that selection of CRC cells deprived of Ras pathway-activating mutations under glucose-limiting conditions yields reverting clones (Fig. 1a and b), of which a subset contain de novo activating hotspot mutations in *KRAS* or *BRAF* [6], followed by subsequent validation of candidate genes in other Ras pathway phenotypes. A challenge in integrating forward genetic screens with human cancer mutational data is that both accumulate passenger mutations in large genes, such as *CSMD3* and *NAV3*. However, 55 of 163 identified genes with multiple integrations in DLD-1 and RKO clone pools were recurrently mutated in human tumors, which is an unbiased indicator of relevance in the cancer context. Thirty-one of the 55 genes had no prior pathway assignment. Whereas transposon integration site biases may explain part of the overlap between the two genetic backgrounds and with murine transposon screens, they are unlikely to selectively affect cancer genes. Therefore, prioritization of genes for validation based on: (1) their degree of mutual exclusivity with mutations in known pathway members in different Ras-driven cancers; (2) gene or transcript size; and (3) status as known cancer gene with unknown function could guide further investigations into this gene set.

Stable shRNA-mediated knockdown of *NCOA3*, *FOXO3*, and *TCF7L2* in DLD-1 *KRAS*^*Wt/-*^ genetic background increased cell growth in low glucose (Fig. 2a), demonstrating the ability of this forward genetics approach to identify mediators of the Ras pathway. In these knockdown lines, the levels of *GLUT1* expression were significantly changed (Fig. 2c–f), which was not the case in corresponding knockdowns in the SW48 colorectal cell line which harbors wild-type KRAS (Additional file 1: Figure S9A). This strengthens the connection between altered *GLUT1* expression and Ras pathway activation demonstrated in [6]. Additionally, Ras signaling controls anchorage-independent growth [5] and the level of pERK as well as pMEK [31]. Here, there were significant reductions in anchorage-independent growth and pERK/pMEK level by stable knockdown of *NCOA3*, *FOXO3*, and *TCF7L2*, similar to knockdown of mutant *KRAS*, in DLD-1 (KRAS G13D) cells. From these validation efforts, *FOXO3*, *NCOA3*, and *TCF7L2* emerged as mediators of Ras pathway phenotypes. It was noteworthy that these genes acted as negative regulators for low-glucose growth (Fig. 2a) and *GLUT1* expression (Fig. 2c–f), but also as positive regulators of anchorage independent growth (Fig. 3a) and pERK/pMEK (Fig. 3c–f). Resistance to cetuximab was reported to be dependent predominantly on the KRAS mutation status [32, 33]. Here, this was not observed in DLD-1 (KRAS G13D) likely due to non-complete knockdown. However, knockdown of *FOXO3* and *NCOA3* conferred significantly higher cetuximab resistance in DLD-1 (KRAS G13D) cells (Fig. 5a). The significance of the role of NCOA3 and FOXO3 in mediating cetuximab resistance will require further studies in vitro and in clinical materials. Several additional mechanisms for cetuximab resistance, other than the KRAS mutation status, have been identified, including accumulation of stress granules [34] and constitutive activation of EGFR effector molecules [35]. Given that cetuximab resistance is primarily dependent on *KRAS* mutation status, and that we observed enhanced cetuximab resistance upon stable knockdown of *FOXO3* and *NCOA3* (Fig. 5a), the data strengthen the links between NCOA3 and FOXO3, and the Ras pathway in CRC. However, the pleiotropic effects of *FOXO3*, *NCOA3*, and *TCF7L2* observed here can potentially be explained by signaling pathways cross-talk and by the dependency of Ras pathway gene phenotypes on other mutations, genetic background, and the environment [36–38].

These three genes (i.e. *FOXO3*, *NCOA3*, and *TCF7L2*) also play roles in other human cancers. The prevalence of somatic *FOXO3* aberrations is 41%, 9%, and 0.5% in breast cancer, prostate cancer, and CRCs in humans, it has been implicated in liver and brain tumors in mice [39] and is a negative regulator of Ras/MAPK [40]. Cancers with activating Ras mutations exhibit enhanced autophagy, where FOXO3 plays a critical role [41]. Copy number alterations and point mutations in *NCOA3* (*AIB1*) have been observed in human breast (31%) and CRCs (14%) [39, 42] and it is a transposon target in murine models of colorectal, liver, pancreas cancer, and melanoma [39, 43]. In vitro phosphorylation of NCOA3 by MAPK has also been demonstrated [44]. The cancer gene *TCF7L2* [45] is a component of the Wnt pathway, a recurrent fusion oncogene in human CRC [46, 47], mutated in 14% of CRCs and breast cancers and a transposon target in murine genetic models of CRCs, liver tumors, and brain tumors [39]. Interestingly, aberrations in *KRAS* and *TCF7L2* in human CRC tended to co-occur [2] (*P* = 0.004, Fisher’s exact test). We observed enrichment of Wnt pathway components among the targeted genes (Additional file 1: Table S3). This suggests that: (1) *TCF7L2* is a cross-talk point between Wnt and Ras pathways; and (2) additional aberrations of Wnt signaling may compensate for a lack of canonical Ras pathway mutations in CRC. It is also noteworthy that there were enrichments of integrations in other pathways, other than the Ras pathway (Additional file 1: Table S4). A plausible explanation would be cross-talk between these cancer pathways. Molecular network analyses showed associations of *FOXO3*, *NCOA3*, and *TCF7L2* with canonical Ras pathway components that had previously been found mutated in human CRC (Additional file 1: Figure S14). TCF7L2 and FOXO3 were directly associated with the c-Myc oncogene, which is overexpressed in subset of human CRCs [48] and regulated by Ras [49]. Collectively, these findings support a role for the three genes *FOXO3*, *NCOA3*, and *TCF7L2* as actors or modulators of the EGFR/Ras/MAPK pathway in human CRC.

To further strengthen the evidence for NCOA3, FOXO3, and TCF7L2 as mediators of the Ras pathway, we performed LC-MS-based global proteomics and observed several significant changes in Ras pathway proteins. These included FGF2, LGALS1, PLCG2, CDKN1A, ERBB2, FYN, EPHA2, RAB8A, MAPKAPK2, RB1, IKBKB, RAB35, RAB3A, and EGFR, with CDKN1A upregulated in all four knockdown lines. In contrast, fewer Ras-related proteins were modulated in the three stable knockdown lines in DLD-1 *KRAS*^*Wt/-*^ genetic background. This is in agreement with *NCOA3*, *FOXO3*, and *TCF7L2* having roles in the activated Ras pathway.

## Conclusions

In summary, we have established proof-of-concept that a pathway-specific forward genetic screen help chart the Ras system in human CRC by identifying known as well as novel pathway members of potential clinical relevance. The gene sets provided here harbor priority candidates for future functional evaluation of their role in Ras pathway phenotypes in CRC and analogous approaches could be envisioned in dissecting other cancer phenotypes and pathways.

## Additional file

Additional file 1: Figures S1–S15 and **Tables S1–S10** with legends. (PDF 9090 kb)

## Abbreviations

CCGD: Candidate Cancer Gene Database
CRC: Colorectal cancer
DMEM: Dulbecco’s Modified Eagle Medium
HCD: Higher-energy collisional dissociation
HGNC: HUGO Gene Nomenclature Committee
KEGG: Kyoto Encyclopedia of Genes and Genomes
PB-CAG-SD: *piggyBac*-CAG promoter-splice donor
PB-GT: *piggyBac*-gene trap
qPCR: Quantitative PCR
RT-PCR: Real-time polymerase chain reaction
TCGA: The Cancer Genome Atlas
