# Supplementary material for: Linking FOXO3, NCOA3, and TCF7L2 to Ras pathway phenotypes through a genome-wide forward genetic screen in human colorectal cancer cells
Source: Genome Med. 2018 Jan 4;10:2. doi: 10.1186/s13073-017-0511-4 (PMC5755028; doi:10.1186/s13073-017-0511-4)
Supplement: Additional file 1: Figures S1–S15 — and Tables S1–S10 with legends. (PDF 9090 kb) [file 13073_2017_511_MOESM1_ESM.pdf]

Linking *FOXO3*, *NCOA3*, and *TCF7L2* to Ras pathway phenotypes through a genome-wide forward genetic screen in human colorectal cancer cells

Authors: Snehangshu Kundu[1], Muhammad Akhtar Ali[1], Niklas Handin[2], Narendra Padhan[1], Jimmy Larsson[1], Maria Karoutsou[1], Kenneth Ban[3, 4], Jacek R. Wiśniewski[5], Per Artursson[2], Liqun He[1], Mats Hellström[1], and Tobias Sjöblom[1*]

Expanded view Materials:

Supplementary Figures with legends S1-15

Tables S1-10

Supplementary Figures and legends:

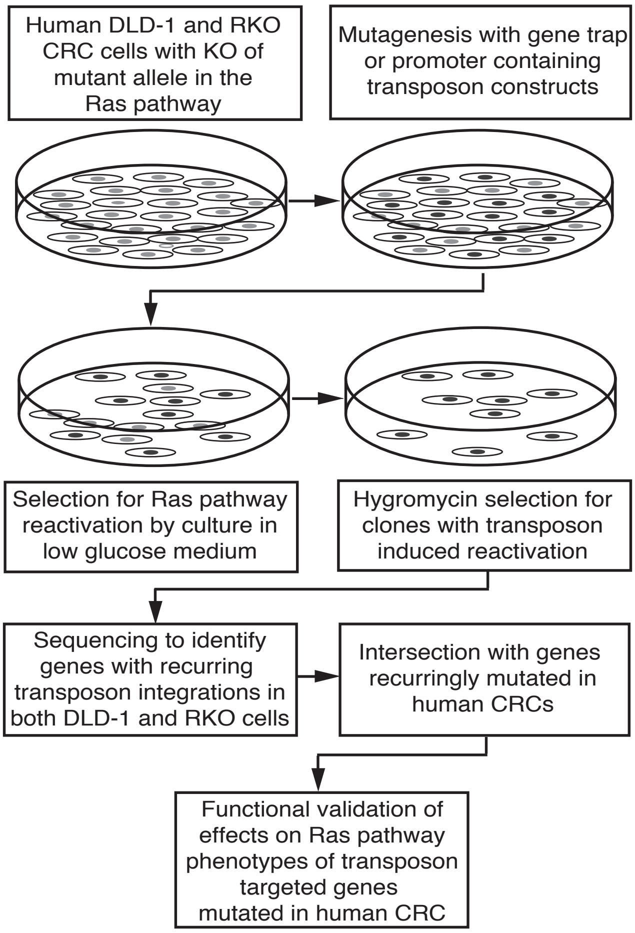

Figure S1. Forward genetic screen to identify genes conferring a Ras pathway phenotype on human colorectal cancer cells. Human DLD-1 and RKO CRC cells deprived of a mutant oncogene in the Ras pathway were transiently transfected with *piggyBac* transposase and gene trap or promoter containing transposons. Cell clones resistant to glucose deprivation were then obtained by selection in medium containing 0.5 mM glucose for ~3 weeks. To eliminate clones generated by endogenous mutagenesis due to MMR deficiency or other non-transposon mediated processes, hygromycin was applied to select for glucose resistant clones with productive transposon integrations. Genes with transposon integration events were identified by splinkerette PCR and sequencing. By intersecting genes with recurring transposon integrations with genes observed mutated in genomic analyses of human CRCs, a cancer relevant set of genes with ability to confer the Ras pathway low glucose growth phenotype can be derived and validated by assessment of other Ras pathway phenotypes.

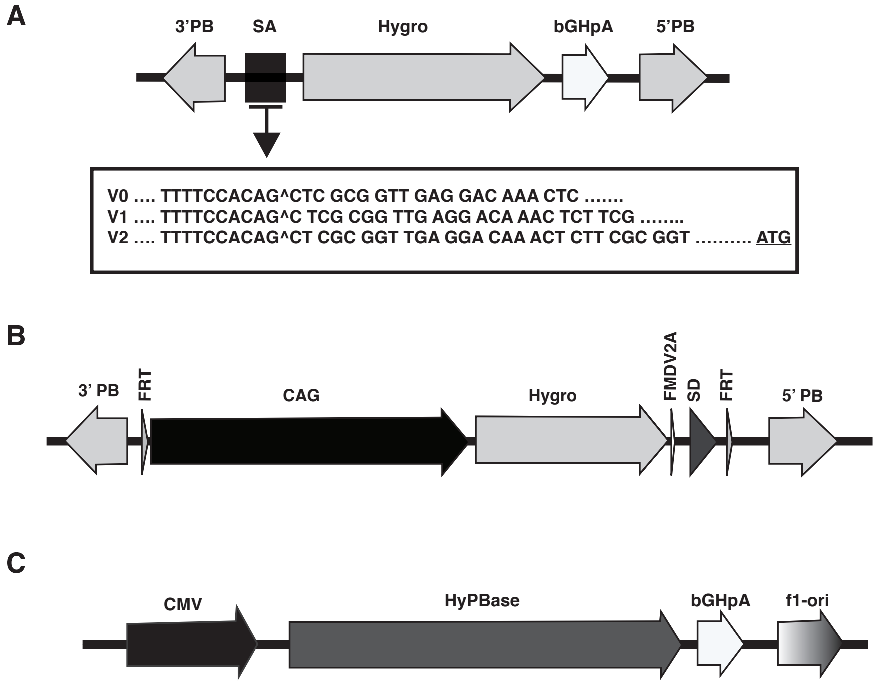

Figure S2. ***piggyBac*** **transposon constructs.** Gene trap constructs (PB-GT) in three reading frames (V0, V1 and V2, inset) (A), activation construct (PB-CAG-SD) (B) and HyPBase transposase construct (C). 3’ and 5’ PB, 3’ and 5’ inverted terminal repeats of *piggyBac (PB)* transposon; SA, adenoviral splice acceptor; Hygro, hygromycin phosphotransferase; bGHpA, bovine growth hormone polyadenylation sequence; FRT, Flippase Recombination Target; CAG, CMV enhancer and chicken beta-actin promoter; FMDV2A, Foot-and-Mouth Disease Virus 2A; SD, splice donor sequence; CMV, cytomegalovirus promoter; HyPBase, Hyperactive mouse codon optimized *piggyBac* transposase; f1-ori, fi-origin of replication sequence.

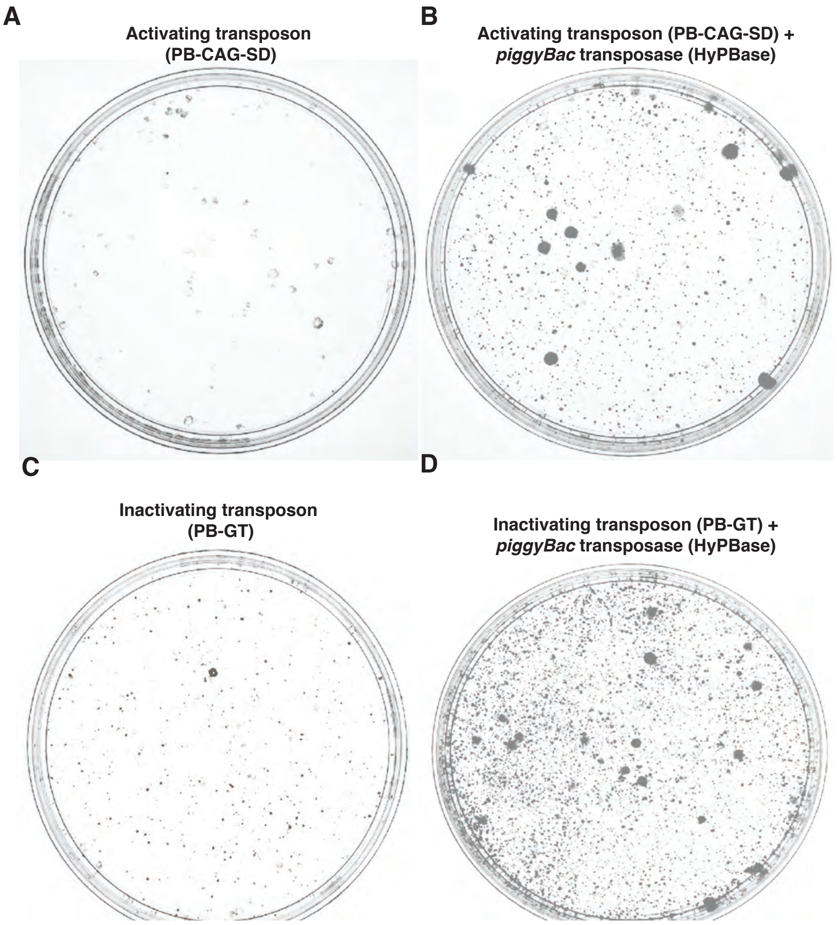

Figure S3. Clonogenic assay for transposon mediated reversion to growth in low glucose medium. Transposon integrations reversing the lost ability to grow under glucose-restricted conditions conferred by Ras pathway activation were generated in a two-step selection procedure. 2.5×10^5 DLD-1 *KRAS*$^{wt/-}$ cells were transfected with a *piggyBac* transposon (either inactivating or activating) along with *piggyBac* transposase (*HyPBase*). Cells were selected in DMEM with 0.4 mM L-glucose for 23 days followed by selection in 0.25 mg/ml hygromycin for 10-12 days and staining in Trypan blue. Activation transposon alone (A), activation transposon + HyPBase (B), gene trap transposon only (C), and gene trap transposon + HyPBase (D).

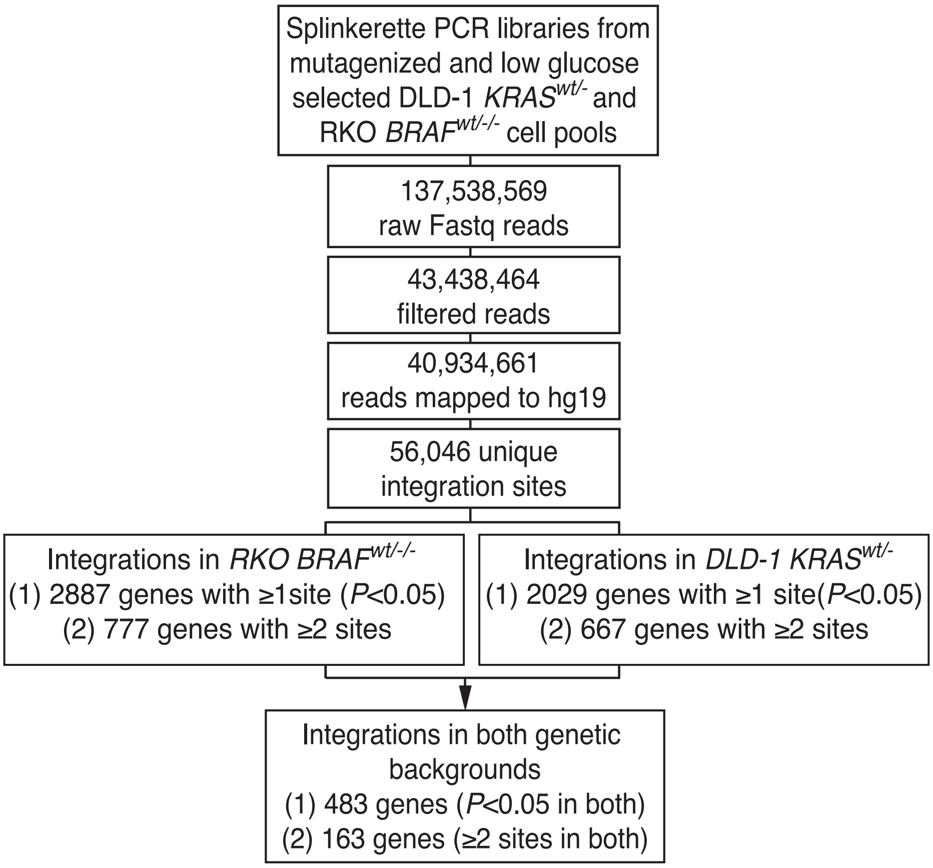

Figure S4. Flow charts for bioinformatic analysis. Flow chart of bioinformatic analysis to identify genes with transposon integrations in the clone pools. Two complementary

approaches to identify genes of interest were used, (1) Poisson statistics or (2) selection of genes with multiple integrations in both genetic backgrounds (see Methods).

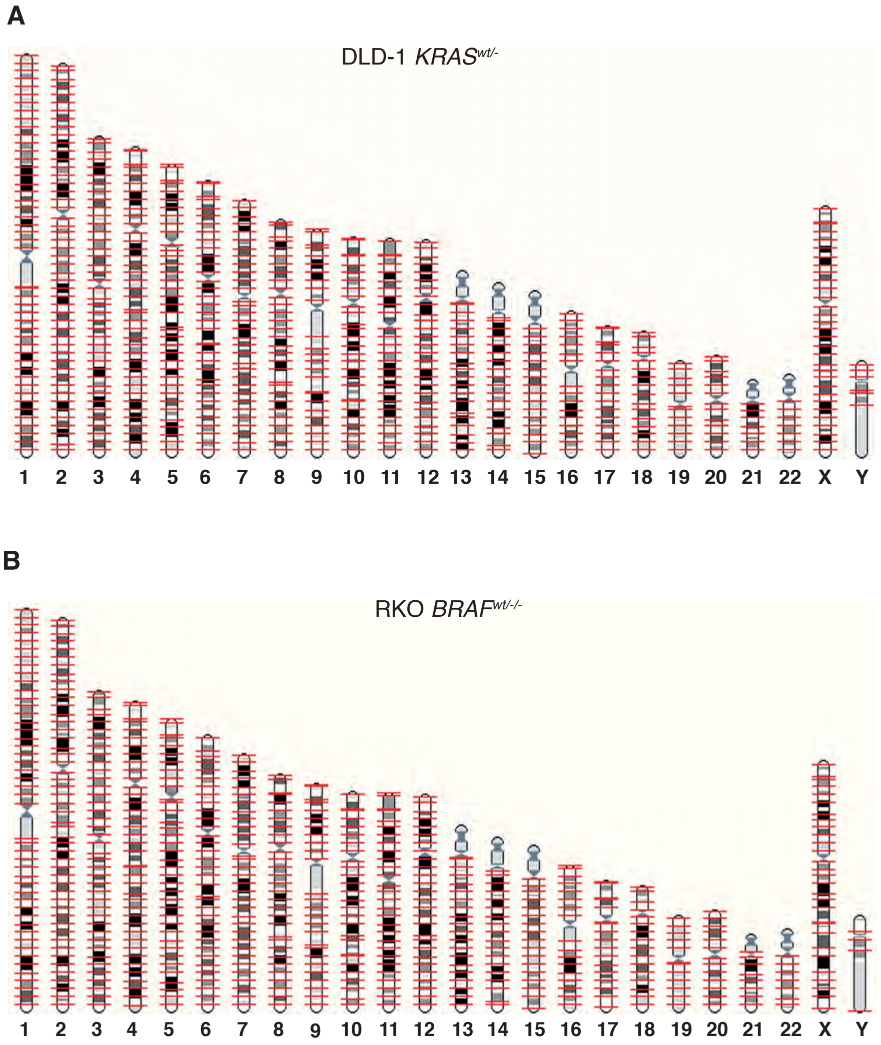

Figure S5. Transposon integration patterns in *DLD-1 KRAS$^{wt/-}$* and *RKO BRAF$^{wt/-/-}$* libraries. Ideograms showing chromosomal integrations of transposons in DLD-1 *KRAS$^{wt/-}$* (A) and RKO *BRAF$^{wt/-/-}$* pools (B).

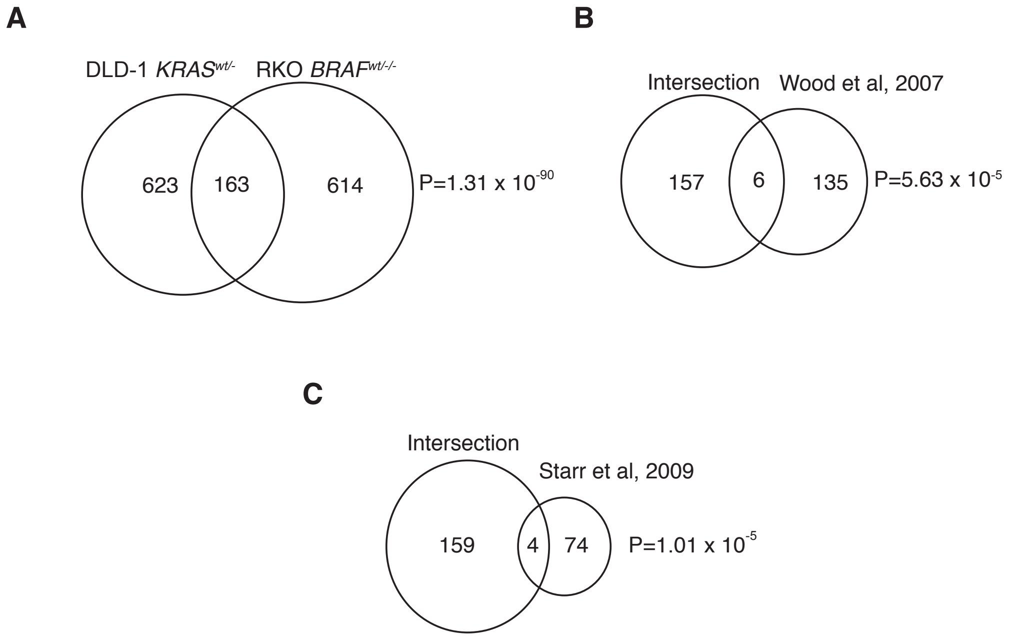

Figure S6. Genes with recurring transposon integration sites in both DLD-1 *KRAS*$^{wt/-}$ and RKO *BRAF*$^{wt/-/-}$ transposon mutagenized libraries selected in low glucose previously linked to CRC. The intersections between genes with recurring transposon insertions in both cell systems **(A)** , with human candidate CRC genes from Wood *et al.* (1) (B) or, mouse candidate CRC genes from Starr *et al.* (2) (C) were visualized by Venn diagrams. The *P* values were calculated using the hypergeometric distribution.

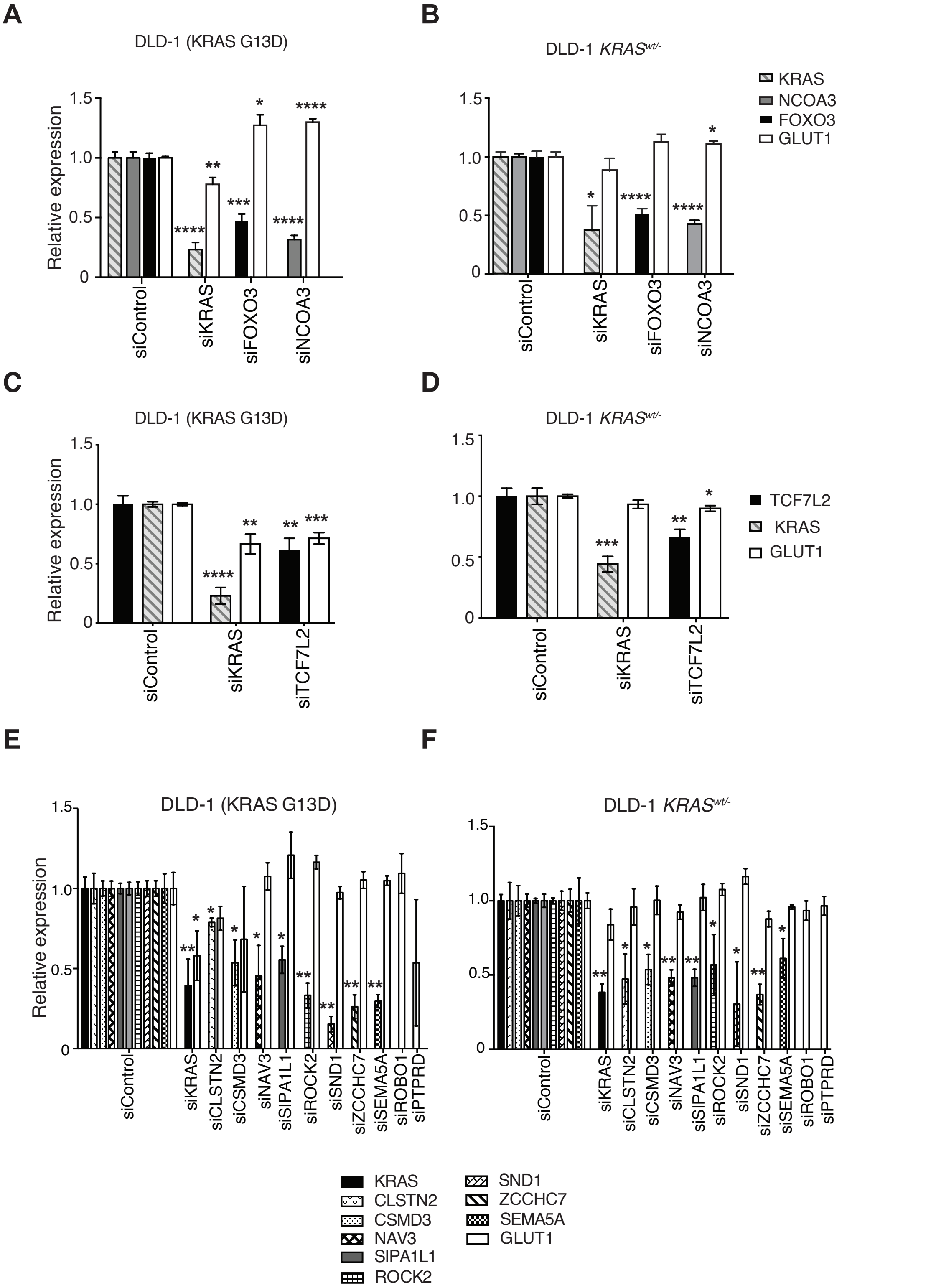

Figure S7. Identification of regulators of GLUT1 expression by transient siRNA knockdown. Whereas transient siRNA mediated knockdown of *FOXO3* and *NCOA3* showed significantly increased *GLUT1* expression in parental DLD-1 *KRAS* G13D (A) and DLD-1

KRAS$^{wt/-}$ (B), knockdown of *TCF7L2* showed significantly reduced *GLUT1* expression in parental DLD-1 KRAS G13D (C) and DLD-1 *KRAS*$^{wt/-}$(D) as compared to siControl. Knockdown of *CLSTN2, CSMD3, NAV3, PTPRD, ROBO1, ROCK2, SIPA1L1, SND1* and *ZCCHC7* did not significantly affect GLUT1 transcript levels (E, F). No transcripts encoding PTPRD and ROBO1 were detected in DLD-1 cells. Transcript levels were determined by qPCR using beta-actin as endogenous control and normalized to control siRNA-transfected cells. Each experiment was performed with three biological replicates and three technical replicates. The *P* values were calculated using Student's *T* test where ****: $p < 0.0001$, ***: $p < 0.001$, **: $p < 0.01$, *: $p< 0.05$.

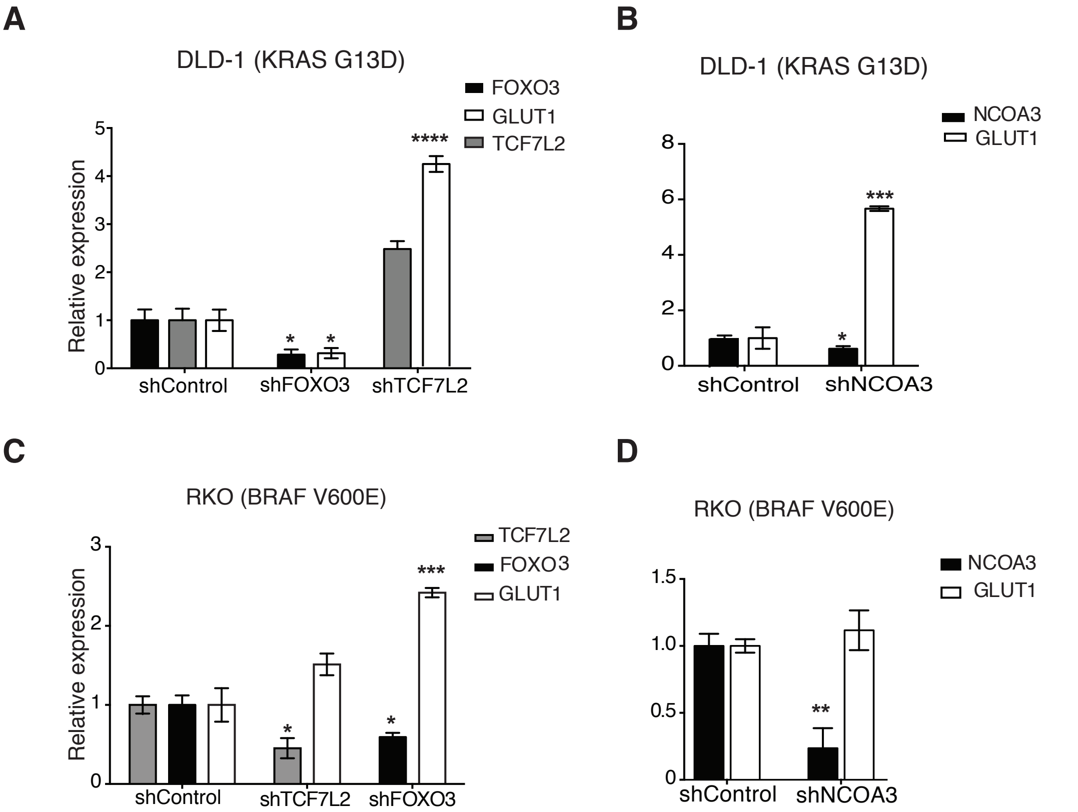

Figure S8. The transposon target genes *FOXO3, NCOA3, and TCF7L2* regulate *GLUT1* expression. Stable knockdown by shRNA of *FOXO3, TCF7L2,* and *NCOA3* in parental DLD-1 (KRAS G13D) (A and B), RKO (BRAF V600E) (C and D). Gene expression levels were measured by qPCR using beta-actin as endogenous control and normalized to

shControl. Each experiment was performed three times with three technical replicates. The statistical analysis was performed by Student's *T* test where ****: $p < 0.0001$, ***: $p < 0.001$, **: $p < 0.01$, *: $p < 0.05$.

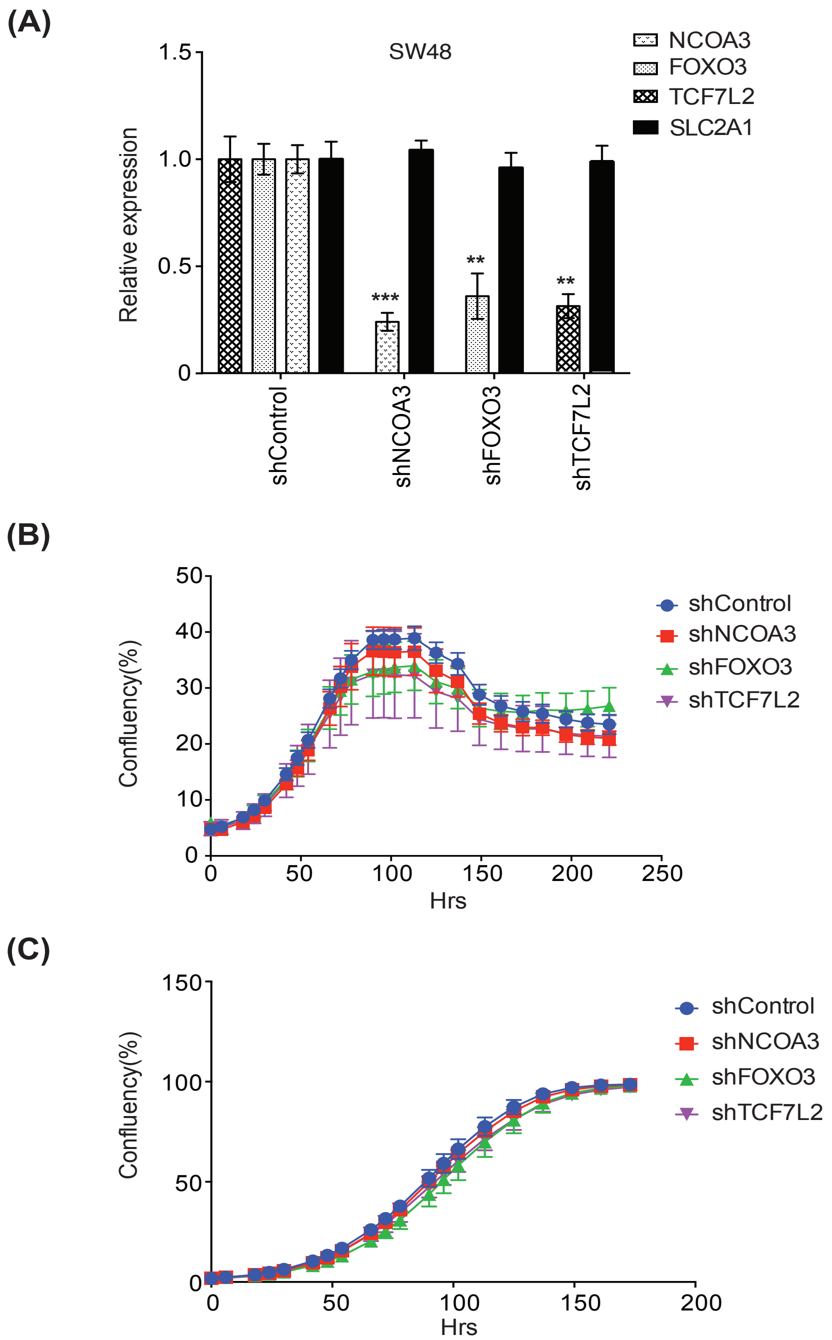

Figure S9. Stable knock-down of *NCOA3, FOXO3* and *TCF7L2* in SW48 genetic background had no effect on SLC2A1 expression and growth advantage in low-glucose (0.4mM) condition. The *GLUT1* expression levels after stable knock down of these three

genes in SW48 cell line **(A)**. Real time growth curves for stable shRNA knockdown lines of *NCOA3, FOXO3* and *TCF7L2* in SW48 cells in low glucose medium (0.4mM) **(B)** and normal medium **(C).** Each experiment was performed twice with four technical replicates. The statistical analysis was performed by Student's *T* test where ****: $p < 0.0001$, ***: $p < 0.001$, **: $p < 0.01$, *: $p < 0.05$.

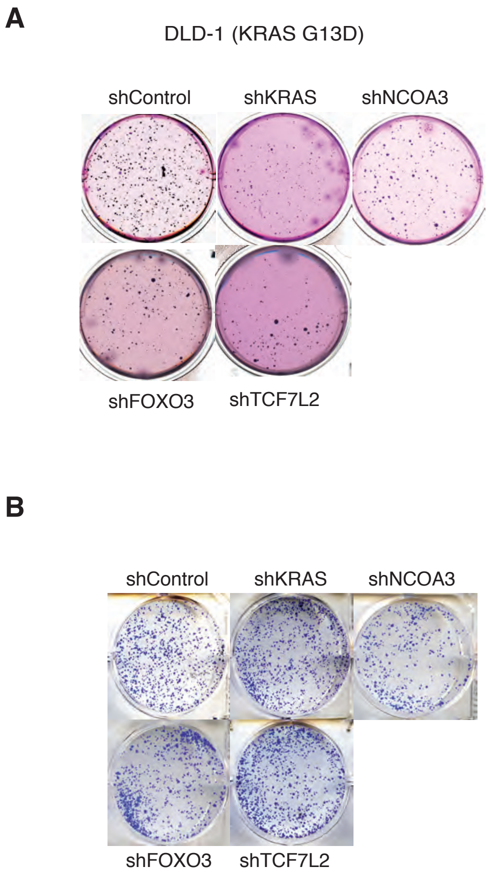

Figure S10. Soft agar assays, growth rates and colony formation on plastic for stable knockdown lines for *KRAS, NCOA3, FOXO3* and *TCF7L2* in DLD-1 (KRAS G13D) cells. Soft agar plates stained with Crystal Violet, growth curve of DLD-1 (KRAS G13D) cells in medium with normal glucose concentration and colony formation assays on plastic stained with Methylene Blue for DLD-1 KRAS G13D (A and B).

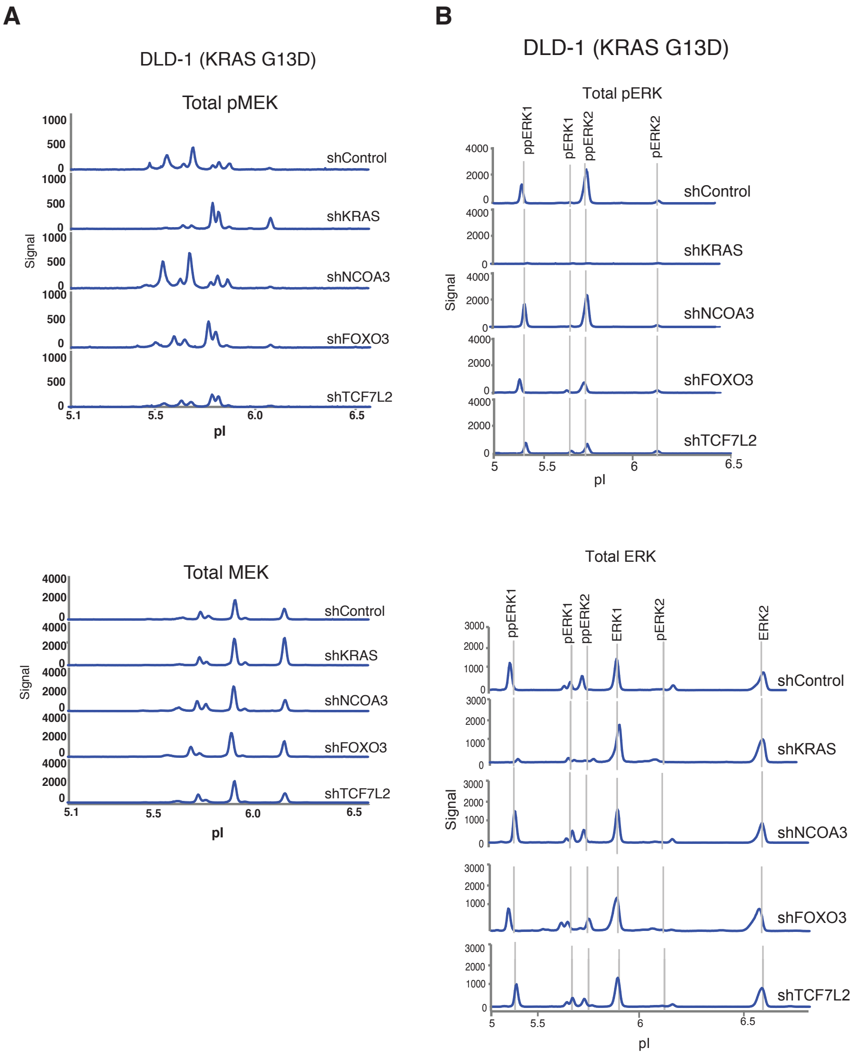

Figure S11. Qualitative data from stable knock-down lines of *FOXO3*, *NCOA3* and *TCF7L2* in DLD-1 (KRAS G13D) for pMEK (A) and pERK (B). (A) The level of pMEK (upper panel) in comparison to MEK (lower panel) was measured by NanoPro analysis in stable shRNA lines of *NCOA3, FOXO3* and *TCF7L2* in DLD-1 (KRAS G13D). Qualitative data were shown as representative electropherograms in the upper and lower panels for total pMEK and total MEK protein respectively. (B) The level of pERK (upper panel) and ERK (lower panel) was measured by NanoPro analysis in stable shRNA lines of NCOA3, FOXO3 and TCF7L2 in DLD-1 cells. Representative electropherograms showing pERK1/2 (ppERK1+pERK1+ppERK2+pERK2) and total ERK1/2 (ppERK1+pERK1+ERK1+ppERK2+pERK2+ERK2). A stable knockdown line for shKRAS

was used as a positive control and a shControl line was used as negative control. Each experiment was performed twice with three technical replicates of each sample.

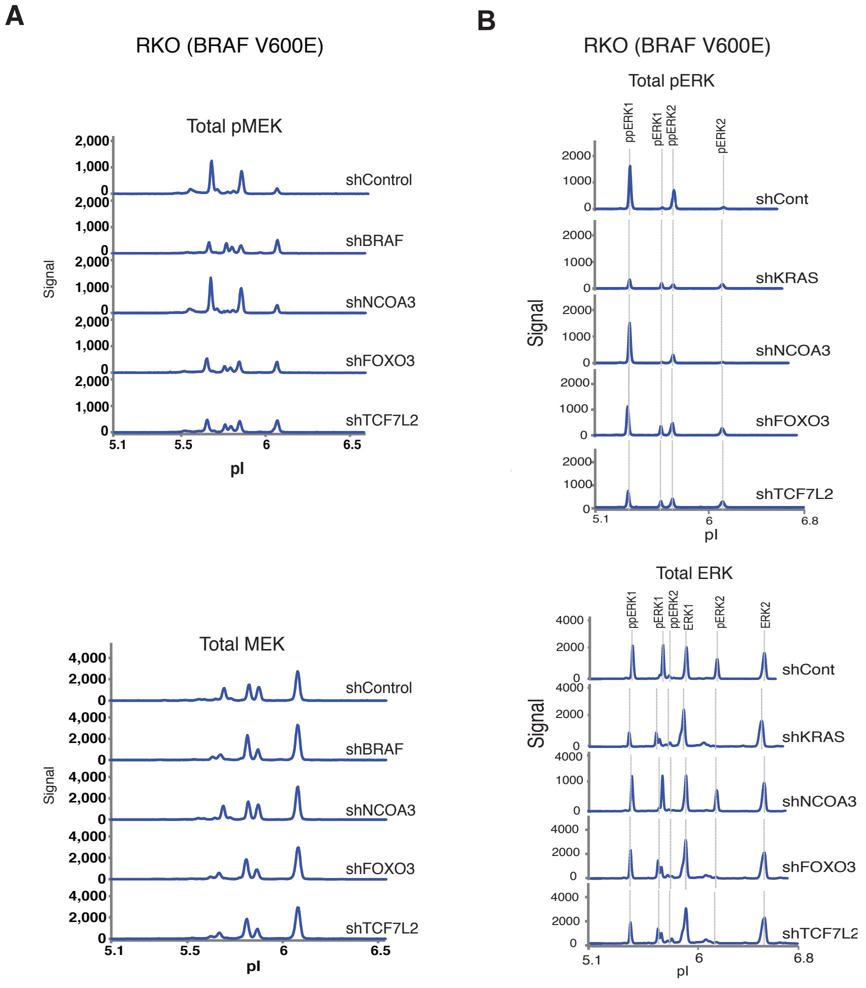

Figure S12. Qualitative data from stable knock-down lines of *FOXO3, NCOA3* and *TCF7L2* in RKO (BRAF V600E) cells for pMEK (A) and pERK (B). (A) The level of pMEK (upper panel) in comparison to MEK (lower panel) was measured by NanoPro analysis in stable shRNA lines of NCOA3, FOXO3 and TCF7L2 in RKO (BRAF V600E) cells. Qualitative data were shown as representative electropherograms for total pMEK and total MEK protein, respectively. (B) The level of pERK (upper panel) in comparison to ERK (lower panel) was measured by NanoPro analysis in stable shRNA lines of NCOA3, FOXO3 and TCF7L2 in RKO (BRAF V600E). Representative electropherograms showing pERK1/2

(ppERK1+pERK1+ppERK2+pERK2) and total ERK1/2 (ppERK1+pERK1+ERK1+ppERK2+pERK2+ERK2). A stable knockdown line for shBRAF was used as a positive control and a shControl line was used as negative control. Each experiment was performed twice with three technical replicates of each sample.

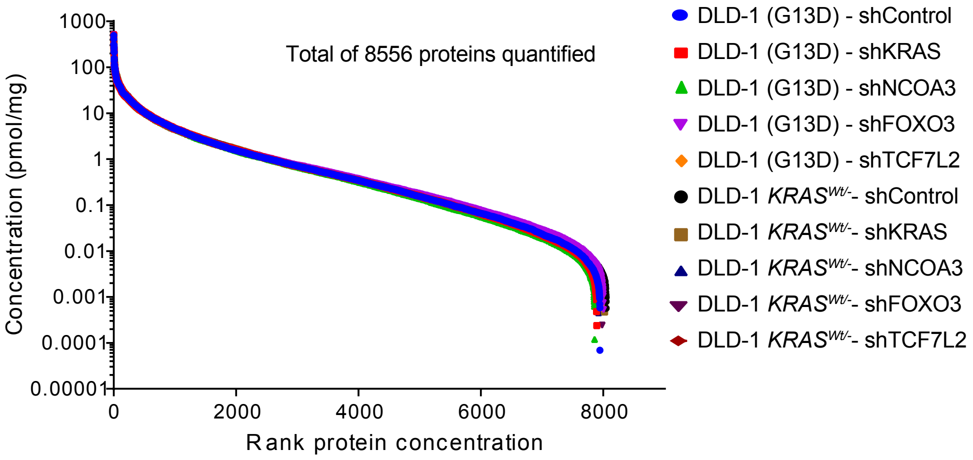

Figure S13. A ranked protein concentration plot from the highest to the lowest concentration for the total proteome of stable shRNA knock-down lines of *KRAS, NCOA3, FOXO3* and *TCF7L2* in DLD-1 and DLD-1 *KRAS*$^{wt/-}$ genetic background showed total of 8556 proteins identified with concentrations covering 7 orders of magnitude.

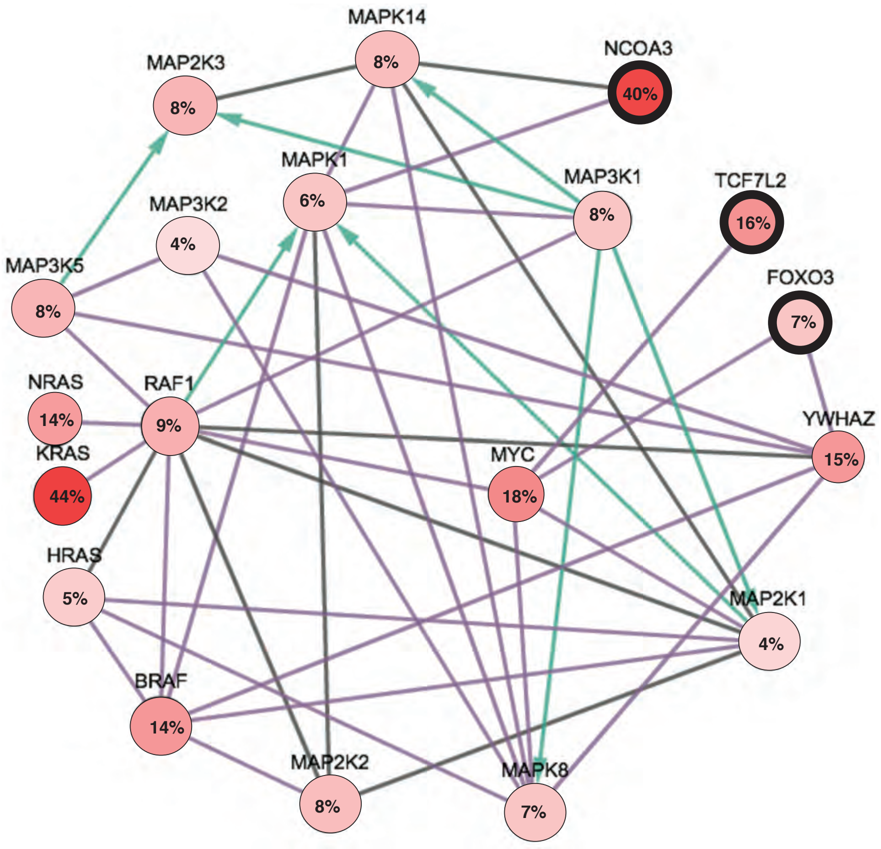

Figure S14. Associations of FOXO3, NCOA3 and TCF7L2 with the most frequently altered canonical Ras pathway genes in human CRC. CytoScape molecular network analysis with numbers denoting frequencies of gene alterations observed in human CRC (3) and connecting lines denoting association by protein-complex-formation, state changes such as phosphorylating-dephosphorylating, or physical interactions. The genes *FOXO3, NCOA3* and *TCF7L2* were used as seeds and 17 of the 50 most frequently altered neighbor genes from a total of 876 genes altered in ≥3% of patients were included.

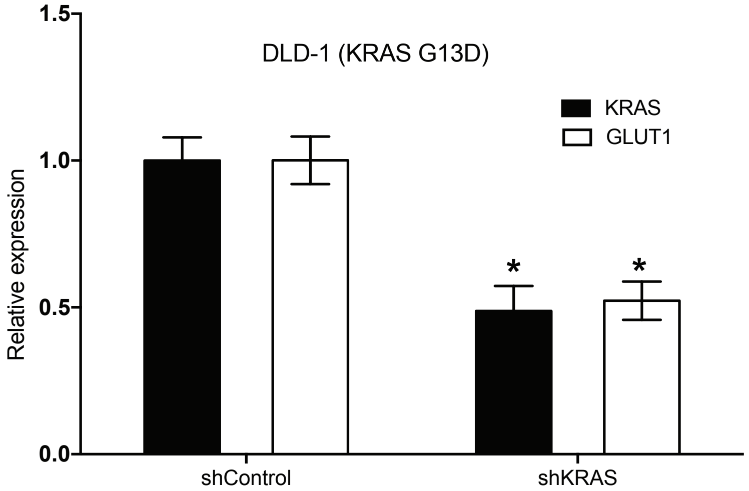

Figure S15. Level of *KRAS* and *GLUT1* transcript in *shKRAS* stable knock-downs in DLD-1 (KRAS G13D) cell line. The levels of *KRAS* and *GLUT1* transcripts were quantified by qPCR using beta-actin as endogenous control. The quantification was repeated twice. The *P* value was calculated by Student's *T* test.

Supplementary Table legends:

Table S1. Genes with significant transposon integrations in ≥1 TTAA site from DLD-1 *KRAS*$^{wt/-}$ transposon mutagenized clone pools after selection in low glucose and hygromycin. The targeted genes from two independent inactivating or activating transposon libraries in DLD-1 *KRAS*$^{wt/-}$ cell lines having at least one *piggyBac* transposon integration were analyzed. The expected number of integrations in a gene was computed as the total number of observed integrations on its chromosome multiplied by the fraction of TTAA sites in the gene versus the chromosome. The *P* values were calculated using Poisson statistics, with False Discovery Rate (FDR) correction for multiple tests and a corrected *P* value <0.05 as cutoff for selection of significant genes.

Table S2. Genes with significant transposon integrations in ≥1 TTAA site from RKO *BRAF*$^{wt/-/-}$ transposon mutagenized clone pools after selection in low glucose and

hygromycin. The targeted genes from two independent inactivating or activating transposon libraries in RKO *BRAF*$^{wt/-/-}$ cell lines having at least one *piggyBac* transposon integration were analyzed. The expected number of integrations in a gene was computed as the total number of observed integrations on its chromosome multiplied by the fraction of TTAA sites in the gene versus the chromosome. The *P* values were calculated using Poisson statistics, with False Discovery Rate (FDR) correction for multiple tests and a corrected *P* value <0.05 as cutoff for selection of significant genes.

Table S3. Common significant transposon targeted genes with ≥1 TTAA site present in both RKO *BRAF*$^{wt/-/-}$ and DLD-1 *KRAS*$^{wt/-}$ transposon mutagenized clone pools after selection in low glucose and hygromycin. Significantly targeted genes in DLD-1 *KRAS*$^{wt/-}$ (Table S1) as well as RKO *BRAF*$^{wt/-/-}$ (Table S2) cell pools after selection in low glucose and hygromycin.

Table S4. Pathway analysis of 483 common significant transposon targeted genes in RKO *BRAF*$^{wt/-/-}$ and DLD-1 *KRAS*$^{wt/-}$ clone pools identifies enrichment in CRC pathways. A KEGG pathway analysis of the 483 common transposon targeted genes with significantly high count of transposon integrations (Supplementary Table S3).

Table S5a. Pathway assignment of 163 genes with at least two unique transposon integration sites in both RKO *BRAF*$^{wt/-/-}$ *and* DLD-1 *KRAS*$^{wt/-}$ transposon mutagenized clone pools after selection in low glucose and hygromycin. Primary publications in PubMed along with the Kyoto Encyclopedia of Genes and Genomes (KEGG Pathways) were searched (1) to determine whether the CIS genes were *bona fide* cancer genes (recurringly somatically mutated by point mutations, amplifications, deletions or translocations) in any

human tumor type, (2) for genetic or functional evidence for assignment to any of the canonical CRC pathways Wnt, EGFR/Ras/MAPK, PIK3CA, or TGFB, or to the Hippo and Rap1 pathways associated with EGFR/Ras/MAPK signaling, (3) for evidence for a role in intracellular glucose metabolism. Numbers, PubMed PMIDs of publication(s) supporting the assignment. KEGG, assignment to pathway based on KEGG Pathways. Mutual exclusivity was analysed as described ((4)). Briefly, MSS tumors from the COAD dataset, CNA with mutations data set along with mRNA expressions data were included and the mutual exclusivity was determined on the basis of Odds Ratio < 0.1 with a P values < 0.05 derived via Fisher's Exact test.

Table S5b-d. Pathway assignments of 163 randomly sampled *ENSEMBL* features. Primary publications in PubMed along with the Kyoto Encyclopedia of Genes and Genomes (KEGG Pathways) were searched (1) to determine whether the genes were *bona fide* cancer genes (recurringly somatically mutated by point mutations, amplifications, deletions or translocations) in any human tumor type, (2) for genetic or functional evidence for assignment to any of the canonical CRC pathways Wnt, EGFR/Ras/MAPK, PIK3CA, or TGFB, or to the Hippo and Rap1 pathways associated with EGFR/Ras/MAPK signaling, (3) for evidence for a role in intracellular glucose metabolism. Numbers, PubMed PMIDs of publication(s) supporting the assignment. KEGG, assignment to pathway based on KEGG Pathways.

Table S6. Barcoded primers for library amplification. Barcodes were denoted with letters whereas the primer sequence was denoted with uppercase letters.

Table S7. Target sequences for siRNAs and TaqMan probes for si/shRNA mediated gene knock-down. Smart pools of three siRNAs and TaqMan assays used for analysing gene expression levels.

Table S8. Lentivirus clones for shRNA mediated gene knock-down. GIPZ shRNA lentiviral particle sets for three validated genes (FOXO3, NCOA3 and TCF7L2) along with two known Ras pathway genes (KRAS and BRAF).

Table S9. Alterations in Ras pathway protein expression levels by stable knockdown of KRAS, FOXO3, NCOA3, and TCF7L2. Lysates from stable shRNA knockdown cell lines in DLD-1 cells with or without mutant KRAS were subjected to global proteomics analysis. All proteins that were quantified with LC-MS and associated with the RAS pathway were subjected to a two sample two-tailed t-test corrected with permutation FDR against a control. The Pathway column is divided into 3 parts, pathway name, id and source, with the "#" as a delimiter. Proteins associated with several pathways have them given after each other also with "#" as the delimiter. Fold changes in the expression as compared to the shControl in respective genetic backgrounds are shown with significant changes (FDR ≤0.05) indicated.

Table S10. *In silico* analysis of the GLUT1 (SLC2A1) promoter reveals binding sites for TCF7L2 and FOXO3. *In silico* analyses were performed by JASPER (http://jasper.genereg.net) to determine whether binding sites for FOXO3, NCOA3 and TCF7L2 exist in the GLUT1 promoter (3kb upstream sequence of the initiation site of the GLUT1 gene). While the known positive regulator of GLUT1, HIF1A, had 38 binding sites with a median score of 5.27, this analysis revealed 16 and 13 binding sites for TCF7L2 and FOXO3 with a median score of 5.34 and 5.36, but no direct binding site for NCOA.

table S1. Genes with significant transposon integrations in DLD-1 *KRAS*$^{wt/-}$ cells after selection in low glucose medium.

Gene ID	Description	Chrom	Start	End	# TTAA sites	Expected	Observed	Libraries	*P*	FDR	Transposon
PCDH7	protocadherin 7 [Source:HGNC Symbol;Acc:8659]	chr4	30722037	31148422	3945	1.292	19	2	0.000E+00	0.000E+00	PB-GT
RAD51B	RAD51 homolog B (S. cerevisiae) [Source:HGNC Symbol;Acc:9822]	chr14	68286496	69196935	6011	2.157	18	2	2.343E-12	3.049E-09	PB-GT
ZBTB20	zinc finger and BTB domain containing 20 [Source:HGNC Symbol;Acc:13503]	chr3	114056941	114866118	7258	2.384	17	2	1.018E-10	8.829E-08	PB-GT
LINC00472	long intergenic non-protein coding RNA 472 [Source:HGNC Symbol;Acc:21380]	chr6	72054047	72130472	706	0.250	7	2	2.987E-10	1.618E-07	PB-GT
PDP1	pyruvate dehyrogenase phosphatase catalytic subunit 1 [Source:HGNC Symbol;Acc:9279]	chr8	94870035	94938294	386	0.151	6	2	3.108E-10	1.618E-07	PB-GT
MIR31HG	MIR31 host gene (non-protein coding) [Source:HGNC Symbol;Acc:37187]	chr9	21455641	21559668	857	0.281	7	2	7.484E-10	3.247E-07	PB-GT
HMGA2	high mobility group AT-hook 2 [Source:HGNC Symbol;Acc:5009]	chr12	66217911	66360075	1139	0.503	8	2	3.595E-09	1.337E-06	PB-GT
PHLDB2	pleckstrin homology-like domain, family B, member 2 [Source:HGNC Symbol;Acc:29573]	chr3	111451344	111695364	1829	0.601	8	2	1.638E-08	5.330E-06	PB-GT
NR2F2	nuclear receptor subfamily 2, group F, member 2 [Source:HGNC Symbol;Acc:7976]	chr15	96869167	96883492	92	0.034	3	1	5.307E-08	1.404E-05	PB-GT
SNORA4	small nucleolar RNA, H/ACA box 4 [Source:HGNC Symbol;Acc:32587]	chr3	186505401	186505538	1	0.000	1	1	5.393E-08	1.404E-05	PB-GT
EIF4G2	eukaryotic translation initiation factor 4 gamma, 2 [Source:HGNC Symbol;Acc:3297]	chr11	10818597	10830657	97	0.035	3	2	6.290E-08	1.489E-05	PB-GT
HK2	hexokinase 2 [Source:HGNC Symbol;Acc:4923]	chr2	75061108	75120486	285	0.105	4	1	9.772E-08	2.120E-05	PB-GT
DLGAP1-AS2	DLGAP1 antisense RNA 2 [Source:HGNC Symbol;Acc:28146]	chr18	3602998	3608334	29	0.009	2	2	1.322E-07	2.648E-05	PB-GT
PBX1	pre-B-cell leukemia homeobox 1 [Source:HGNC Symbol;Acc:8632]	chr1	164524821	164868533	2215	0.785	8	2	1.539E-07	2.823E-05	PB-GT
C14orf182	chromosome 14 open reading frame 182 [Source:HGNC Symbol;Acc:27503]	chr14	50448430	50474238	125	0.045	3	2	1.627E-07	2.823E-05	PB-GT
PLCXD2-AS1	PLCXD2 antisense RNA 1 [Source:HGNC Symbol;Acc:41145]	chr3	111395583	111396280	2	0.001	1	1	2.157E-07	3.509E-05	PB-GT
HIST1H1C	histone cluster 1, H1c [Source:HGNC Symbol;Acc:4716]	chr6	26055968	26056699	2	0.001	1	1	2.497E-07	3.824E-05	PB-GT
SNHG6	small nucleolar RNA host gene 6 (non-protein coding) [Source:HGNC Symbol;Acc:32965]	chr8	67833919	67838633	31	0.012	2	2	2.944E-07	4.257E-05	PB-GT
UCA1	urothelial cancer associated 1 (non-protein coding) [Source:HGNC Symbol;Acc:37126]	chr19	15939757	15947130	37	0.013	2	2	3.396E-07	4.652E-05	PB-GT
KRTAP4-12	keratin associated protein 4-12 [Source:HGNC Symbol;Acc:16776]	chr17	39279343	39280419	2	0.001	1	1	5.612E-07	7.305E-05	PB-GT
DDX39B-AS1	DDX39B antisense RNA 1 [Source:HGNC Symbol;Acc:39771]	chr6	31510081	31510915	4	0.001	1	1	9.984E-07	1.238E-04	PB-GT
ASIC3	acid-sensing (proton-gated) ion channel 3 [Source:HGNC Symbol;Acc:101]	chr7	150745379	150749843	5	0.002	1	1	1.395E-06	1.650E-04	PB-GT
OR51M1	olfactory receptor, family 51, subfamily M, member 1 [Source:HGNC Symbol;Acc:14847]	chr11	5410607	5411664	5	0.002	1	1	1.654E-06	1.871E-04	PB-GT
MRVI1	murine retrovirus integration site 1 homolog [Source:HGNC Symbol;Acc:7237]	chr11	10594638	10715535	532	0.194	4	2	1.930E-06	2.093E-04	PB-GT
ATP6V1G2-DDX39B	ATP6V1G2-DDX39B readthrough (non-protein coding) [Source:HGNC Symbol;Acc:41999]	chr6	31497996	31514385	68	0.024	2	2	2.272E-06	2.309E-04	PB-GT
SIAH1	siah E3 ubiquitin protein ligase 1 [Source:HGNC Symbol;Acc:10857]	chr16	48390275	48482313	466	0.201	4	1	2.306E-06	2.309E-04	PB-GT
EIF2S2P4	eukaryotic translation initiation factor 2, subunit 2 beta pseudogene 4 [Source:HGNC Symbol;Acc:37626]	chr2	171608221	171609562	6	0.002	1	1	2.442E-06	2.355E-04	PB-GT
ART5	ADP-ribosyltransferase 5 [Source:HGNC Symbol;Acc:24049]	chr11	3659733	3663546	7	0.003	1	1	3.239E-06	3.007E-04	PB-GT
CCAT1	colon cancer associated transcript 1 (non-protein coding) [Source:HGNC Symbol;Acc:45128]	chr8	128220111	128231333	70	0.027	2	1	3.351E-06	3.007E-04	PB-GT
KRT18	keratin 18 [Source:HGNC Symbol;Acc:6430]	chr12	53342655	53346685	6	0.003	1	1	3.500E-06	3.036E-04	PB-GT
TXNRD1	thioredoxin reductase 1 [Source:HGNC Symbol;Acc:12437]	chr12	104609557	104744061	923	0.407	5	2	4.481E-06	3.653E-04	PB-GT
ARHGAP18	Rho GTPase activating protein 18 [Source:HGNC Symbol;Acc:21035]	chr6	129897277	130031370	1153	0.408	5	2	4.491E-06	3.653E-04	PB-GT
PRDX2	peroxiredoxin 2 [Source:HGNC Symbol;Acc:9353]	chr19	12907634	12912694	9	0.003	1	1	4.775E-06	3.656E-04	PB-GT
RPL18	ribosomal protein L18 [Source:HGNC Symbol;Acc:10310]	chr19	49118585	49122793	9	0.003	1	1	4.775E-06	3.656E-04	PB-GT
RBM14	RNA binding motif protein 14 [Source:HGNC Symbol;Acc:14219]	chr11	66384053	66406124	87	0.032	2	1	5.166E-06	3.842E-04	PB-GT
SPIN4-AS1	SPIN4 antisense RNA 1 [Source:HGNC Symbol;Acc:41177]	chrX	62569525	62572057	20	0.003	1	1	5.540E-06	3.852E-04	PB-GT
GALNT7	UDP-N-acetyl-alpha-D-galactosamine:polypeptide N-acetylgalactosaminyltransferase 7 (GalNAc-T7) [Source:HGNC Symbol;Acc:4129]	chr4	174089904	174245118	1310	0.429	5	2	6.013E-06	3.852E-04	PB-GT
EVA1C	eva-1 homolog C (C. elegans) [Source:HGNC Symbol;Acc:13239]	chr21	33784314	33887707	446	0.113	3	2	6.168E-06	3.852E-04	PB-GT
GNA14	guanine nucleotide binding protein (G protein), alpha 14 [Source:HGNC Symbol;Acc:4382]	chr9	80037995	80263223	1321	0.433	5	2	6.319E-06	3.852E-04	PB-GT
SH3BP4	SH3-domain binding protein 4 [Source:HGNC Symbol;Acc:10826]	chr2	235860617	235964358	308	0.114	3	2	6.324E-06	3.852E-04	PB-GT
MLL2	myeloid/lymphoid or mixed-lineage leukemia 2 [Source:HGNC Symbol;Acc:7133]	chr12	49412758	49453557	77	0.034	2	2	6.376E-06	3.852E-04	PB-GT
NFKBIA	nuclear factor of kappa light polypeptide gene enhancer in B-cells inhibitor, alpha [Source:HGNC Symbol;Acc:7797]	chr14	35870717	35873955	10	0.004	1	1	6.422E-06	3.852E-04	PB-GT
ZBED2	zinc finger, BED-type containing 2 [Source:HGNC Symbol;Acc:20710]	chr3	111311747	111314290	11	0.004	1	1	6.512E-06	3.852E-04	PB-GT
FRG2C	FSHD region gene 2 family, member C [Source:HGNC Symbol;Acc:33626]	chr3	75713481	75716371	11	0.004	1	1	6.512E-06	3.852E-04	PB-GT
SMIM2-AS1	SMIM2 antisense RNA 1 [Source:HGNC Symbol;Acc:42674]	chr13	44716682	44813010	478	0.115	3	1	6.741E-06	3.899E-04	PB-GT
HIST1H2BC	histone cluster 1, H2bc [Source:HGNC Symbol;Acc:4757]	chr6	26115101	26124154	101	0.036	2	1	7.381E-06	4.157E-04	PB-GT
TNFRSF12A	tumor necrosis factor receptor superfamily, member 12A [Source:HGNC Symbol;Acc:18152]	chr16	3068446	3072384	9	0.004	1	1	7.506E-06	4.157E-04	PB-GT
ARHGAP28	Rho GTPase activating protein 28 [Source:HGNC Symbol;Acc:25509]	chr18	6729717	6915715	1412	0.452	5	2	8.031E-06	4.355E-04	PB-GT
PRKAG1	protein kinase, AMP-activated, gamma 1 non-catalytic subunit [Source:HGNC Symbol;Acc:9385]	chr12	49396057	49412980	84	0.037	2	2	8.258E-06	4.385E-04	PB-GT
MTUS1	microtubule associated tumor suppressor 1 [Source:HGNC Symbol;Acc:29789]	chr8	17501304	17658426	1165	0.456	5	2	8.424E-06	4.385E-04	PB-GT
CDK3	cyclin-dependent kinase 3 [Source:HGNC Symbol;Acc:1772]	chr17	73996987	74002080	8	0.004	1	1	8.961E-06	4.486E-04	PB-GT
TNK1	tyrosine kinase, non-receptor, 1 [Source:HGNC Symbol;Acc:11940]	chr17	7283853	7293093	8	0.004	1	1	8.961E-06	4.486E-04	PB-GT
LIPC	lipase, hepatic [Source:HGNC Symbol;Acc:6619]	chr15	58702768	58861151	737	0.271	4	2	9.717E-06	4.702E-04	PB-GT
MTND5P28	MT-ND5 pseudogene 28 [Source:HGNC Symbol;Acc:42290]	chr2	120972757	120974855	12	0.004	1	1	9.755E-06	4.702E-04	PB-GT
FOSB	FBJ murine osteosarcoma viral oncogene homolog B [Source:HGNC Symbol;Acc:3797]	chr19	45971253	45978437	13	0.004	1	1	9.953E-06	4.711E-04	PB-GT
CALHM2	calcium homeostasis modulator 2 [Source:HGNC Symbol;Acc:23493]	chr10	105206543	105212660	11	0.005	1	1	1.118E-05	5.050E-04	PB-GT
DCBLD2	discoidin, CUB and LCCL domain containing 2 [Source:HGNC Symbol;Acc:24627]	chr3	98514785	98620533	850	0.279	4	2	1.121E-05	5.050E-04	PB-GT
KCNK5	potassium channel, subfamily K, member 5 [Source:HGNC Symbol;Acc:6280]	chr6	39156749	39197226	117	0.041	2	2	1.143E-05	5.050E-04	PB-GT
CRYGA	crystallin, gamma A [Source:HGNC Symbol;Acc:2408]	chr2	209025464	209028300	13	0.005	1	1	1.145E-05	5.050E-04	PB-GT
RHOBTB3	Rho-related BTB domain containing 3 [Source:HGNC Symbol;Acc:18757]	chr5	95049226	95160087	865	0.282	4	2	1.185E-05	5.139E-04	PB-GT
OLR1	oxidized low density lipoprotein (lectin-like) receptor 1 [Source:HGNC Symbol;Acc:8133]	chr12	10310902	10324737	98	0.043	2	1	1.305E-05	5.508E-04	PB-GT
ACTR1B	ARP1 actin-related protein 1 homolog B, centractin beta (yeast) [Source:HGNC Symbol;Acc:168]	chr2	98272431	98280570	14	0.005	1	1	1.327E-05	5.508E-04	PB-GT
PRR25	proline rich 25 [Source:HGNC Symbol;Acc:37230]	chr16	855443	863861	12	0.005	1	1	1.333E-05	5.508E-04	PB-GT
EGLN2	egl nine homolog 2 (C. elegans) [Source:HGNC Symbol;Acc:14660]	chr19	41304901	41314338	16	0.005	1	1	1.507E-05	6.128E-04	PB-GT
SLC25A25	solute carrier family 25 (mitochondrial carrier; phosphate carrier), member 25 [Source:HGNC Symbol;Acc:20663]	chr9	130830480	130871524	143	0.047	2	1	1.656E-05	6.633E-04	PB-GT
POLR2G	polymerase (RNA) II (DNA directed) polypeptide G [Source:HGNC Symbol;Acc:9194]	chr11	62529016	62534182	16	0.006	1	1	1.689E-05	6.640E-04	PB-GT
MET	met proto-oncogene (hepatocyte growth factor receptor) [Source:HGNC Symbol;Acc:7029]	chr7	116312444	116438440	915	0.306	4	2	1.729E-05	6.640E-04	PB-GT
MYL9	myosin, light chain 9, regulatory [Source:HGNC Symbol;Acc:15754]	chr20	35169887	35178228	16	0.006	1	1	1.762E-05	6.640E-04	PB-GT
FHL2	four and a half LIM domains 2 [Source:HGNC Symbol;Acc:3703]	chr2	105974169	106054960	402	0.148	3	2	1.785E-05	6.640E-04	PB-GT
HIST1H3J	histone cluster 1, H3j [Source:HGNC Symbol;Acc:4774]	chr6	27858093	27860884	17	0.006	1	1	1.798E-05	6.640E-04	PB-GT
MBNL2	muscleblind-like splicing regulator 2 [Source:HGNC Symbol;Acc:16746]	chr13	97873688	98046374	1280	0.309	4	1	1.818E-05	6.640E-04	PB-GT
DNAJC17	DnaJ (Hsp40) homolog, subfamily C, member 17 [Source:HGNC Symbol;Acc:25556]	chr15	41060067	41099675	132	0.049	2	2	1.837E-05	6.640E-04	PB-GT
KRT23	keratin 23 (histone deacetylase inducible) [Source:HGNC Symbol;Acc:6438]	chr17	39078948	39093886	94	0.050	2	1	1.985E-05	7.077E-04	PB-GT
VMP1	vacuole membrane protein 1 [Source:HGNC Symbol;Acc:29559]	chr17	57784553	57919616	1005	0.533	5	2	2.012E-05	7.078E-04	PB-GT
OLFML2B	olfactomedin-like 2B [Source:HGNC Symbol;Acc:24558]	chr1	161952982	161993644	145	0.051	2	1	2.174E-05	7.546E-04	PB-GT
SPIN4	spindlin family, member 4 [Source:HGNC Symbol;Acc:27040]	chrX	62567107	62571223	40	0.007	1	1	2.211E-05	7.573E-04	PB-GT
DLGAP1-AS1	DLGAP1 antisense RNA 1 [Source:HGNC Symbol;Acc:31676]	chr18	3593730	3598350	21	0.007	1	1	2.247E-05	7.597E-04	PB-GT
RGS14	regulator of G-protein signaling 14 [Source:HGNC Symbol;Acc:9996]	chr5	176784838	176799602	21	0.007	1	1	2.340E-05	7.689E-04	PB-GT
CCL2	chemokine (C-C motif) ligand 2 [Source:HGNC Symbol;Acc:10618]	chr17	32582237	32584222	13	0.007	1	1	2.362E-05	7.689E-04	PB-GT
CLDN6	claudin 6 [Source:HGNC Symbol;Acc:2048]	chr16	3064713	3070072	16	0.007	1	1	2.367E-05	7.689E-04	PB-GT
RBM14-RBM4	RBM14-RBM4 readthrough [Source:HGNC Symbol;Acc:38840]	chr11	66384097	66413940	146	0.053	2	1	2.402E-05	7.689E-04	PB-GT
CDX2	caudal type homeobox 2 [Source:HGNC Symbol;Acc:1806]	chr13	28536274	28545276	29	0.007	1	1	2.440E-05	7.689E-04	PB-GT
ZNF146	zinc finger protein 146 [Source:HGNC Symbol;Acc:12931]	chr19	36705504	36729676	156	0.054	2	1	2.468E-05	7.689E-04	PB-GT
AQP6	aquaporin 6, kidney specific [Source:HGNC Symbol;Acc:639]	chr12	50360977	50370922	16	0.007	1	1	2.481E-05	7.689E-04	PB-GT
SEMA4F	sema domain, immunoglobulin domain (Ig), transmembrane domain (TM) and short cytoplasmic domain, (semaphorin) 4F [Source:HGNC Symbol;Acc:10734	chr2	74881355	74909186	147	0.054	2	2	2.546E-05	7.798E-04	PB-GT
ERRFI1	ERBB receptor feedback inhibitor 1 [Source:HGNC Symbol;Acc:18185]	chr1	8064464	8086368	155	0.055	2	2	2.649E-05	7.957E-04	PB-GT
RHOXF1	Rhox homeobox family, member 1 [Source:HGNC Symbol;Acc:29993]	chrX	119243011	119249847	44	0.007	1	1	2.674E-05	7.957E-04	PB-GT
FAM219B	family with sequence similarity 219, member B [Source:HGNC Symbol;Acc:24695]	chr15	75192328	75199462	20	0.007	1	1	2.690E-05	7.957E-04	PB-GT
EHBP1L1	EH domain binding protein 1-like 1 [Source:HGNC Symbol;Acc:30682]	chr11	65343509	65360121	21	0.008	1	1	2.906E-05	8.498E-04	PB-GT
PPIB	peptidylprolyl isomerase B (cyclophilin B) [Source:HGNC Symbol;Acc:9255]	chr15	64448011	64455404	21	0.008	1	1	2.965E-05	8.575E-04	PB-GT
MYPOP	Myb-related transcription factor, partner of profilin [Source:HGNC Symbol;Acc:20178]	chr19	46393278	46405862	23	0.008	1	1	3.108E-05	8.785E-04	PB-GT
MBD3	methyl-CpG binding domain protein 3 [Source:HGNC Symbol;Acc:6918]	chr19	1576639	1592882	23	0.008	1	1	3.108E-05	8.785E-04	PB-GT
RDH16	retinol dehydrogenase 16 (all-trans) [Source:HGNC Symbol;Acc:29674]	chr12	57345219	57353158	18	0.008	1	1	3.139E-05	8.785E-04	PB-GT
NME5	NME/NM23 family member 5 [Source:HGNC Symbol;Acc:7853]	chr5	137450866	137475132	180	0.059	2	2	3.238E-05	8.967E-04	PB-GT
ADORA2B	adenosine A2b receptor [Source:HGNC Symbol;Acc:264]	chr17	15848231	15879060	112	0.059	2	1	3.333E-05	8.985E-04	PB-GT
ZNF646	zinc finger protein 646 [Source:HGNC Symbol;Acc:29004]	chr16	31085743	31095517	19	0.008	1	1	3.336E-05	8.985E-04	PB-GT
APIP	APAF1 interacting protein [Source:HGNC Symbol;Acc:17581]	chr11	34874641	34938046	479	0.174	3	1	3.348E-05	8.985E-04	PB-GT
ITM2C	integral membrane protein 2C [Source:HGNC Symbol;Acc:6175]	chr2	231729354	231743963	23	0.008	1	1	3.574E-05	9.379E-04	PB-GT
PRR15L	proline rich 15-like [Source:HGNC Symbol;Acc:28149]	chr17	46029333	46035244	16	0.008	1	1	3.574E-05	9.379E-04	PB-GT
TTC39A	tetratricopeptide repeat domain 39A [Source:HGNC Symbol;Acc:18657]	chr1	51752930	51810788	172	0.061	2	1	3.603E-05	9.379E-04	PB-GT
AP5Z1	adaptor-related protein complex 5, zeta 1 subunit [Source:HGNC Symbol;Acc:22197]	chr7	4815253	4833943	27	0.009	1	1	4.047E-05	1.033E-03	PB-GT
LINC00525	long intergenic non-protein coding RNA 525 [Source:HGNC Symbol;Acc:40290]	chr7	47801074	47806370	27	0.009	1	1	4.047E-05	1.033E-03	PB-GT
AKAP4	A kinase (PRKA) anchor protein 4 [Source:HGNC Symbol;Acc:374]	chrX	49955406	49965664	55	0.009	1	1	4.173E-05	1.035E-03	PB-GT
COQ4	coenzyme Q4 homolog (S. cerevisiae) [Source:HGNC Symbol;Acc:19693]	chr9	131084815	131096351	28	0.009	1	1	4.185E-05	1.035E-03	PB-GT
S100A2	S100 calcium binding protein A2 [Source:HGNC Symbol;Acc:10492]	chr1	153533584	153540366	26	0.009	1	1	4.217E-05	1.035E-03	PB-GT
NPR1	natriuretic peptide receptor A/guanylate cyclase A (atrionatriuretic peptide receptor A) [Source:HGNC Symbol;Acc:7943]	chr1	153651113	153666468	26	0.009	1	1	4.217E-05	1.035E-03	PB-GT
TTYH1	tweety homolog 1 (Drosophila) [Source:HGNC Symbol;Acc:13476]	chr19	54926393	54948080	27	0.009	1	1	4.280E-05	1.035E-03	PB-GT
LAS1L	LAS1-like (S. cerevisiae) [Source:HGNC Symbol;Acc:25726]	chrX	64732462	64754655	56	0.009	1	1	4.326E-05	1.035E-03	PB-GT
AP1S3	adaptor-related protein complex 1, sigma 3 subunit [Source:HGNC Symbol;Acc:18971]	chr2	224616403	224702744	507	0.187	3	1	4.380E-05	1.035E-03	PB-GT
C5orf42	chromosome 5 open reading frame 42 [Source:HGNC Symbol;Acc:25801]	chr5	37106330	37249530	1143	0.373	4	2	4.427E-05	1.035E-03	PB-GT
TFAP2A	transcription factor AP-2 alpha (activating enhancer binding protein 2 alpha) [Source:HGNC Symbol;Acc:11742]	chr6	10393419	10419892	185	0.065	2	2	4.436E-05	1.035E-03	PB-GT
HARS2	histidyl-tRNA synthetase 2, mitochondrial [Source:HGNC Symbol;Acc:4817]	chr5	140071011	140078889	29	0.009	1	1	4.455E-05	1.035E-03	PB-GT
LINC00674	long intergenic non-protein coding RNA 674 [Source:HGNC Symbol;Acc:44355]	chr17	66098049	66111659	125	0.066	2	2	4.610E-05	1.062E-03	PB-GT
DPT	dermatopontin [Source:HGNC Symbol;Acc:3011]	chr1	168664697	168698502	188	0.067	2	1	4.685E-05	1.070E-03	PB-GT
PDLIM4	PDZ and LIM domain 4 [Source:HGNC Symbol;Acc:16501]	chr5	131593364	131609147	30	0.010	1	1	4.766E-05	1.079E-03	PB-GT
AMOT	angiomotin [Source:HGNC Symbol;Acc:17810]	chrX	112017731	112084043	406	0.068	2	1	4.904E-05	1.100E-03	PB-GT
H2AFJ	H2A histone family, member J [Source:HGNC Symbol;Acc:14456]	chr12	14927270	14930936	23	0.010	1	1	5.117E-05	1.138E-03	PB-GT
ZBTB43	zinc finger and BTB domain containing 43 [Source:HGNC Symbol;Acc:17908]	chr9	129567285	129600489	211	0.069	2	2	5.233E-05	1.148E-03	PB-GT
DCTN6	dynactin 6 [Source:HGNC Symbol;Acc:16964]	chr8	30013813	30041156	177	0.069	2	2	5.250E-05	1.148E-03	PB-GT
PRSS21	protease, serine, 21 (testisin) [Source:HGNC Symbol;Acc:9485]	chr16	2867164	2876305	24	0.010	1	1	5.314E-05	1.153E-03	PB-GT
FABP5	fatty acid binding protein 5 (psoriasis-associated) [Source:HGNC Symbol;Acc:3560]	chr8	82192598	82197012	27	0.011	1	1	5.537E-05	1.190E-03	PB-GT
EIF4A1	eukaryotic translation initiation factor 4A1 [Source:HGNC Symbol;Acc:3282]	chr17	7476024	7482323	20	0.011	1	1	5.577E-05	1.190E-03	PB-GT
DNAJB7	DnaJ (Hsp40) homolog, subfamily B, member 7 [Source:HGNC Symbol;Acc:24986]	chr22	41255553	41258130	26	0.011	1	1	5.981E-05	1.257E-03	PB-GT
NIT1	nitrilase 1 [Source:HGNC Symbol;Acc:7828]	chr1	161087876	161095235	31	0.011	1	1	5.987E-05	1.257E-03	PB-GT
LINC00570	long intergenic non-protein coding RNA 570 [Source:HGNC Symbol;Acc:43717]	chr2	11534107	11543203	30	0.011	1	1	6.070E-05	1.260E-03	PB-GT
ADCY7	adenylate cyclase 7 [Source:HGNC Symbol;Acc:238]	chr16	50280048	50352046	169	0.073	2	1	6.101E-05	1.260E-03	PB-GT
LLGL1	lethal giant larvae homolog 1 (Drosophila) [Source:HGNC Symbol;Acc:6628]	chr17	18128901	18148189	21	0.011	1	1	6.146E-05	1.260E-03	PB-GT
CDKN1A	cyclin-dependent kinase inhibitor 1A (p21, Cip1) [Source:HGNC Symbol;Acc:1784]	chr6	36644305	36655116	32	0.011	1	1	6.348E-05	1.291E-03	PB-GT
MLXIPL	MLX interacting protein-like [Source:HGNC Symbol;Acc:12744]	chr7	73007524	73038873	34	0.011	1	1	6.407E-05	1.293E-03	PB-GT
PMAIP1	phorbol-12-myristate-13-acetate-induced protein 1 [Source:HGNC Symbol;Acc:9108]	chr18	57567180	57571538	36	0.012	1	1	6.583E-05	1.318E-03	PB-GT
PWP2	PWP2 periodic tryptophan protein homolog (yeast) [Source:HGNC Symbol;Acc:9711]	chr21	45527171	45551063	46	0.012	1	1	6.717E-05	1.335E-03	PB-GT
BTF3L4	basic transcription factor 3-like 4 [Source:HGNC Symbol;Acc:30547]	chr1	52521797	52556388	214	0.076	2	1	6.863E-05	1.353E-03	PB-GT
RPL7	ribosomal protein L7 [Source:HGNC Symbol;Acc:10363]	chr8	74202506	74208024	31	0.012	1	1	7.291E-05	1.427E-03	PB-GT
EHF	ets homologous factor [Source:HGNC Symbol;Acc:3246]	chr11	34642640	34682604	215	0.078	2	2	7.530E-05	1.463E-03	PB-GT
TMEM14C	transmembrane protein 14C [Source:HGNC Symbol;Acc:20952]	chr6	10723148	10731362	36	0.013	1	1	8.026E-05	1.540E-03	PB-GT
SLC15A3	solute carrier family 15, member 3 [Source:HGNC Symbol;Acc:18068]	chr11	60704556	60720002	35	0.013	1	1	8.044E-05	1.540E-03	PB-GT
SRRM2-AS1	SRRM2 antisense RNA 1 [Source:HGNC Symbol;Acc:44162]	chr16	2787077	2802601	30	0.013	1	1	8.289E-05	1.565E-03	PB-GT
TSPAN13	tetraspanin 13 [Source:HGNC Symbol;Acc:21643]	chr7	16793160	16824161	242	0.081	2	2	8.299E-05	1.565E-03	PB-GT
HLA-DRB6	major histocompatibility complex, class II, DR beta 6 (pseudogene) [Source:HGNC Symbol;Acc:4954]	chr6	32520490	32527799	37	0.013	1	1	8.476E-05	1.587E-03	PB-GT
LINC00310	long intergenic non-protein coding RNA 310 [Source:HGNC Symbol;Acc:16414]	chr21	35552978	35562220	52	0.013	1	1	8.575E-05	1.594E-03	PB-GT
DEFB1	defensin, beta 1 [Source:HGNC Symbol;Acc:2766]	chr8	6728097	6735544	34	0.013	1	1	8.763E-05	1.617E-03	PB-GT
HS1BP3	HCLS1 binding protein 3 [Source:HGNC Symbol;Acc:24979]	chr2	20760208	20850849	224	0.083	2	1	8.821E-05	1.617E-03	PB-GT
IRF2BP2	interferon regulatory factor 2 binding protein 2 [Source:HGNC Symbol;Acc:21729]	chr1	234740015	234745271	38	0.013	1	1	8.982E-05	1.635E-03	PB-GT
PIGW	phosphatidylinositol glycan anchor biosynthesis, class W [Source:HGNC Symbol;Acc:23213]	chr17	34890847	34895159	26	0.014	1	1	9.405E-05	1.693E-03	PB-GT
PDE12	phosphodiesterase 12 [Source:HGNC Symbol;Acc:25386]	chr3	57542003	57547684	42	0.014	1	1	9.429E-05	1.693E-03	PB-GT
BMP4	bone morphogenetic protein 4 [Source:HGNC Symbol;Acc:1071]	chr14	54416454	54425479	39	0.014	1	1	9.700E-05	1.729E-03	PB-GT
EGFLAM-AS4	EGFLAM antisense RNA 4 [Source:HGNC Symbol;Acc:41166]	chr5	38282366	38291088	43	0.014	1	1	9.765E-05	1.729E-03	PB-GT
CBLC	Cbl proto-oncogene, E3 ubiquitin protein ligase C [Source:HGNC Symbol;Acc:15961]	chr19	45281126	45303891	41	0.014	1	1	9.837E-05	1.730E-03	PB-GT
EVPL	envoplakin [Source:HGNC Symbol;Acc:3503]	chr17	74000583	74023533	27	0.014	1	1	1.014E-04	1.771E-03	PB-GT
EVI5L	ecotropic viral integration site 5-like [Source:HGNC Symbol;Acc:30464]	chr19	7895119	7929862	42	0.014	1	1	1.032E-04	1.786E-03	PB-GT
ZNF192P1	zinc finger protein 192 pseudogene 1 [Source:HGNC Symbol;Acc:18777]	chr6	28129559	28137372	41	0.014	1	1	1.040E-04	1.786E-03	PB-GT
VWA2	von Willebrand factor A domain containing 2 [Source:HGNC Symbol;Acc:24709]	chr10	115999018	116051272	203	0.087	2	1	1.043E-04	1.786E-03	PB-GT
RBM4	RNA binding motif protein 4 [Source:HGNC Symbol;Acc:9901]	chr11	66384097	66434153	245	0.089	2	1	1.105E-04	1.880E-03	PB-GT
FXYD5	FXYD domain containing ion transport regulator 5 [Source:HGNC Symbol;Acc:4029]	chr19	35645633	35660786	44	0.015	1	1	1.132E-04	1.908E-03	PB-GT
IL17A	interleukin 17A [Source:HGNC Symbol;Acc:5981]	chr6	52051185	52055436	43	0.015	1	1	1.143E-04	1.908E-03	PB-GT
DNAL4	dynein, axonemal, light chain 4 [Source:HGNC Symbol;Acc:2955]	chr22	39174513	39190203	36	0.015	1	1	1.143E-04	1.908E-03	PB-GT
CHST13	carbohydrate (chondroitin 4) sulfotransferase 13 [Source:HGNC Symbol;Acc:21755]	chr3	126243126	126262134	47	0.015	1	1	1.179E-04	1.955E-03	PB-GT
ABHD6	abhydrolase domain containing 6 [Source:HGNC Symbol;Acc:21398]	chr3	58223233	58281420	281	0.092	2	1	1.223E-04	2.015E-03	PB-GT
TMEM51	transmembrane protein 51 [Source:HGNC Symbol;Acc:25488]	chr1	15479028	15546976	268	0.095	2	2	1.329E-04	2.158E-03	PB-GT

table S1. Genes with significant transposon integrations in DLD-1 *KRAS*$^{wt/-}$ cells after selection in low glucose medium.

Gene ID	Description	Chrom	Start	End	# TTAA sites	Expected	Observed	Libraries	*P*	FDR	Transposon
TWIST1	twist basic helix-loop-helix transcription factor 1 [Source:HGNC Symbol;Acc:12428]	chr7	19060614	19157295	748	0.250	3	2	1.333E-04	2.158E-03	PB-GT
ELP5	elongator acetyltransferase complex subunit 5 [Source:HGNC Symbol;Acc:30617]	chr17	7154735	7163259	31	0.016	1	1	1.335E-04	2.158E-03	PB-GT
ZFP42	ZFP42 zinc finger protein [Source:HGNC Symbol;Acc:30949]	chr4	188916925	188926204	51	0.017	1	1	1.380E-04	2.218E-03	PB-GT
CD300LG	CD300 molecule-like family member g [Source:HGNC Symbol;Acc:30455]	chr17	41924516	41940997	32	0.017	1	1	1.422E-04	2.270E-03	PB-GT
ATXN1L	ataxin 1-like [Source:HGNC Symbol;Acc:33279]	chr16	71879894	71891231	40	0.017	1	1	1.469E-04	2.317E-03	PB-GT
SPINK1	serine peptidase inhibitor, Kazal type 1 [Source:HGNC Symbol;Acc:11244]	chr5	147204131	147211349	53	0.017	1	1	1.480E-04	2.317E-03	PB-GT
RTKN	rhotekin [Source:HGNC Symbol;Acc:10466]	chr2	74652963	74669549	47	0.017	1	1	1.484E-04	2.317E-03	PB-GT
PLD2	phospholipase D2 [Source:HGNC Symbol;Acc:9068]	chr17	4710391	4726729	33	0.017	1	1	1.511E-04	2.317E-03	PB-GT
ITIH2	inter-alpha-trypsin inhibitor heavy chain 2 [Source:HGNC Symbol;Acc:6167]	chr10	7745232	7791483	231	0.099	2	1	1.523E-04	2.317E-03	PB-GT
ADORA1	adenosine A1 receptor [Source:HGNC Symbol;Acc:262]	chr1	203059782	203136533	281	0.100	2	2	1.527E-04	2.317E-03	PB-GT
IFI27	interferon, alpha-inducible protein 27 [Source:HGNC Symbol;Acc:5397]	chr14	94571182	94583033	49	0.018	1	1	1.528E-04	2.317E-03	PB-GT
MAP3K14	mitogen-activated protein kinase kinase kinase 14 [Source:HGNC Symbol;Acc:6853]	chr17	43340488	43394414	188	0.100	2	1	1.530E-04	2.317E-03	PB-GT
NOV	nephroblastoma overexpressed [Source:HGNC Symbol;Acc:7885]	chr8	120428546	120436593	45	0.018	1	1	1.531E-04	2.317E-03	PB-GT
EIF4E1B	eukaryotic translation initiation factor 4E family member 1B [Source:HGNC Symbol;Acc:33179]	chr5	176057683	176073642	55	0.018	1	1	1.593E-04	2.391E-03	PB-GT
FGD2	FYVE, RhoGEF and PH domain containing 2 [Source:HGNC Symbol;Acc:3664]	chr6	36973422	36996846	51	0.018	1	1	1.605E-04	2.391E-03	PB-GT
UBE2L3	ubiquitin-conjugating enzyme E2L 3 [Source:HGNC Symbol;Acc:12488]	chr22	21903736	21978323	240	0.101	2	2	1.607E-04	2.391E-03	PB-GT
SMAD6	SMAD family member 6 [Source:HGNC Symbol;Acc:6772]	chr15	66994566	67074338	280	0.103	2	1	1.683E-04	2.489E-03	PB-GT
CRYBA1	crystallin, beta A1 [Source:HGNC Symbol;Acc:2394]	chr17	27573881	27581512	35	0.019	1	1	1.699E-04	2.498E-03	PB-GT
CXXC5	CXXC finger protein 5 [Source:HGNC Symbol;Acc:26943]	chr5	139026884	139063467	57	0.019	1	1	1.711E-04	2.502E-03	PB-GT
TACC3	transforming, acidic coiled-coil containing protein 3 [Source:HGNC Symbol;Acc:11524]	chr4	1723227	1746898	57	0.019	1	1	1.722E-04	2.503E-03	PB-GT
EIF4A2	eukaryotic translation initiation factor 4A2 [Source:HGNC Symbol;Acc:3284]	chr3	186500994	186507689	57	0.019	1	1	1.731E-04	2.503E-03	PB-GT
ARHGEF1	Rho guanine nucleotide exchange factor (GEF) 1 [Source:HGNC Symbol;Acc:681]	chr19	42387228	42434302	55	0.019	1	1	1.764E-04	2.523E-03	PB-GT
ANXA10	annexin A10 [Source:HGNC Symbol;Acc:534]	chr4	169013666	169108841	823	0.270	3	2	1.776E-04	2.523E-03	PB-GT
HMGN1	high mobility group nucleosome binding domain 1 [Source:HGNC Symbol;Acc:4984]	chr21	40714241	40721573	75	0.019	1	1	1.777E-04	2.523E-03	PB-GT
CKS2	CDC28 protein kinase regulatory subunit 2 [Source:HGNC Symbol;Acc:2000]	chr9	91926113	91931618	58	0.019	1	1	1.784E-04	2.523E-03	PB-GT
DHX16	DEAH (Asp-Glu-Ala-His) box polypeptide 16 [Source:HGNC Symbol;Acc:2739]	chr6	30620896	30640814	54	0.019	1	1	1.798E-04	2.530E-03	PB-GT
SAP30L	SAP30-like [Source:HGNC Symbol;Acc:25663]	chr5	153825517	153835891	59	0.019	1	1	1.832E-04	2.553E-03	PB-GT
NLRX1	NLR family member X1 [Source:HGNC Symbol;Acc:29890]	chr11	119037277	119054725	53	0.019	1	1	1.836E-04	2.553E-03	PB-GT
RTEL1	regulator of telomere elongation helicase 1 [Source:HGNC Symbol;Acc:15888]	chr20	62289163	62328416	52	0.019	1	1	1.844E-04	2.553E-03	PB-GT
SOCS6	suppressor of cytokine signaling 6 [Source:HGNC Symbol;Acc:16833]	chr18	67956137	67997436	334	0.107	2	1	1.878E-04	2.586E-03	PB-GT
RAB4B-EGLN2	RAB4B-EGLN2 readthrough (non-protein coding) [Source:HGNC Symbol;Acc:44465]	chr19	41284147	41314103	57	0.020	1	1	1.894E-04	2.595E-03	PB-GT
DDX39B	DEAD (Asp-Glu-Ala-Asp) box polypeptide 39B [Source:HGNC Symbol;Acc:13917]	chr6	31497996	31510225	56	0.020	1	1	1.933E-04	2.634E-03	PB-GT
ZNF565	zinc finger protein 565 [Source:HGNC Symbol;Acc:26726]	chr19	36673188	36737159	315	0.108	2	1	1.951E-04	2.645E-03	PB-GT
GAA	glucosidase, alpha; acid [Source:HGNC Symbol;Acc:4065]	chr17	78075355	78093678	38	0.020	1	1	2.001E-04	2.662E-03	PB-GT
EEF1A1	eukaryotic translation elongation factor 1 alpha 1 [Source:HGNC Symbol;Acc:3189]	chr6	74225473	74233520	57	0.020	1	1	2.002E-04	2.662E-03	PB-GT
GAS5	growth arrest-specific 5 (non-protein coding) [Source:HGNC Symbol;Acc:16355]	chr1	173833038	173838020	57	0.020	1	1	2.012E-04	2.662E-03	PB-GT
PRDX1	peroxiredoxin 1 [Source:HGNC Symbol;Acc:9352]	chr1	45976708	45988719	57	0.020	1	1	2.012E-04	2.662E-03	PB-GT
BMF	Bcl2 modifying factor [Source:HGNC Symbol;Acc:24132]	chr15	40380091	40401093	55	0.020	1	1	2.017E-04	2.662E-03	PB-GT
SHC2	SHC (Src homology 2 domain containing) transforming protein 2 [Source:HGNC Symbol;Acc:29869]	chr19	416583	460996	59	0.020	1	1	2.029E-04	2.662E-03	PB-GT
C9orf40	chromosome 9 open reading frame 40 [Source:HGNC Symbol;Acc:23433]	chr9	77561497	77567802	62	0.020	1	1	2.037E-04	2.662E-03	PB-GT
MORC1-AS1	MORC1 antisense RNA 1 [Source:HGNC Symbol;Acc:40377]	chr3	108820303	108829189	62	0.020	1	1	2.046E-04	2.662E-03	PB-GT
NANOG	Nanog homeobox [Source:HGNC Symbol;Acc:20857]	chr12	7940390	7948655	47	0.021	1	1	2.122E-04	2.743E-03	PB-GT
RTEL1-TNFRSF6B	RTEL1-TNFRSF6B readthrough (non-protein coding) [Source:HGNC Symbol;Acc:44095]	chr20	62290653	62330037	56	0.021	1	1	2.137E-04	2.743E-03	PB-GT
KDM5C	lysine (K)-specific demethylase 5C [Source:HGNC Symbol;Acc:11114]	chrX	53220503	53254604	125	0.021	1	1	2.139E-04	2.743E-03	PB-GT
LCOR	ligand dependent nuclear receptor corepressor [Source:HGNC Symbol;Acc:29503]	chr10	98592017	98740800	1225	0.528	4	1	2.200E-04	2.801E-03	PB-GT
G3BP1	GTPase activating protein (SH3 domain) binding protein 1 [Source:HGNC Symbol;Acc:30292]	chr5	151150606	151192346	346	0.113	2	2	2.209E-04	2.801E-03	PB-GT
SERPINB1	serpin peptidase inhibitor, clade B (ovalbumin), member 1 [Source:HGNC Symbol;Acc:3311]	chr6	2832566	2842240	60	0.021	1	1	2.217E-04	2.801E-03	PB-GT
RAMP3	receptor (G protein-coupled) activity modifying protein 3 [Source:HGNC Symbol;Acc:9845]	chr7	45197390	45225901	64	0.021	1	1	2.255E-04	2.836E-03	PB-GT
ANKRD49	ankyrin repeat domain 49 [Source:HGNC Symbol;Acc:25970]	chr11	94226795	94232749	59	0.021	1	1	2.272E-04	2.844E-03	PB-GT
DUSP5	dual specificity phosphatase 5 [Source:HGNC Symbol;Acc:3071]	chr10	112257596	112271302	50	0.022	1	1	2.285E-04	2.846E-03	PB-GT
CD27-AS1	CD27 antisense RNA 1 [Source:HGNC Symbol;Acc:43896]	chr12	6548167	6560733	49	0.022	1	1	2.305E-04	2.852E-03	PB-GT
APOD	apolipoprotein D [Source:HGNC Symbol;Acc:612]	chr3	195295573	195311076	66	0.022	1	1	2.316E-04	2.852E-03	PB-GT
SULT1C4	sulfotransferase family, cytosolic, 1C, member 4 [Source:HGNC Symbol;Acc:11457]	chr2	108994367	109004513	59	0.022	1	1	2.331E-04	2.852E-03	PB-GT
ZNF7	zinc finger protein 7 [Source:HGNC Symbol;Acc:13139]	chr8	146052849	146072894	56	0.022	1	1	2.364E-04	2.852E-03	PB-GT
POU5F1	POU class 5 homeobox 1 [Source:HGNC Symbol;Acc:9221]	chr6	31130253	31148508	62	0.022	1	1	2.366E-04	2.852E-03	PB-GT
SMAD2	SMAD family member 2 [Source:HGNC Symbol;Acc:6768]	chr18	45357922	45457515	910	0.291	3	2	2.375E-04	2.852E-03	PB-GT
LINC00700	long intergenic non-protein coding RNA 700 [Source:HGNC Symbol;Acc:27422]	chr10	2047667	2056542	51	0.022	1	1	2.377E-04	2.852E-03	PB-GT
TARS2	threonyl-tRNA synthetase 2, mitochondrial (putative) [Source:HGNC Symbol;Acc:30740]	chr1	150459887	150480078	62	0.022	1	1	2.377E-04	2.852E-03	PB-GT
LIMK1	LIM domain kinase 1 [Source:HGNC Symbol;Acc:6613]	chr7	73497263	73536855	67	0.022	1	1	2.470E-04	2.948E-03	PB-GT
RBFOX3	RNA binding protein, fox-1 homolog (C. elegans) 3 [Source:HGNC Symbol;Acc:27097]	chr17	77085427	77613550	1022	0.542	4	1	2.480E-04	2.948E-03	PB-GT
MORN3	MORN repeat containing 3 [Source:HGNC Symbol;Acc:29807]	chr12	122089024	122110537	51	0.023	1	1	2.495E-04	2.951E-03	PB-GT
LEMD1	LEM domain containing 1 [Source:HGNC Symbol;Acc:18725]	chr1	205350506	205425082	333	0.118	2	1	2.506E-04	2.951E-03	PB-GT
LAD1	ladinin 1 [Source:HGNC Symbol;Acc:6472]	chr1	201342372	201368736	64	0.023	1	1	2.532E-04	2.953E-03	PB-GT
DUSP18	dual specificity phosphatase 18 [Source:HGNC Symbol;Acc:18484]	chr22	31048038	31063877	54	0.023	1	1	2.560E-04	2.953E-03	PB-GT
ERI1	exoribonuclease 1 [Source:HGNC Symbol;Acc:23994]	chr8	8859657	8974256	760	0.297	3	2	2.567E-04	2.953E-03	PB-GT
GALNS	galactosamine (N-acetyl)-6-sulfate sulfatase [Source:HGNC Symbol;Acc:4122]	chr16	88880142	88923378	53	0.023	1	1	2.570E-04	2.953E-03	PB-GT
ZFP62	ZFP62 zinc finger protein [Source:HGNC Symbol;Acc:23241]	chr5	180274611	180288286	70	0.023	1	1	2.573E-04	2.953E-03	PB-GT
ZNF385A	zinc finger protein 385A [Source:HGNC Symbol;Acc:17521]	chr12	54762917	54785082	52	0.023	1	1	2.593E-04	2.953E-03	PB-GT
MRPS10	mitochondrial ribosomal protein S10 [Source:HGNC Symbol;Acc:14502]	chr6	42174539	42185603	65	0.023	1	1	2.599E-04	2.953E-03	PB-GT
DEDD	death effector domain containing [Source:HGNC Symbol;Acc:2755]	chr1	161090764	161102478	65	0.023	1	1	2.611E-04	2.953E-03	PB-GT
SYF2	SYF2 homolog, RNA splicing factor (S. cerevisiae) [Source:HGNC Symbol;Acc:19824]	chr1	25549170	25558993	65	0.023	1	1	2.611E-04	2.953E-03	PB-GT
ARHGAP35	Rho GTPase activating protein 35 [Source:HGNC Symbol;Acc:4591]	chr19	47421933	47508334	350	0.120	2	2	2.652E-04	2.953E-03	PB-GT
TUBA4B	tubulin, alpha 4b (pseudogene) [Source:HGNC Symbol;Acc:18637]	chr2	220117965	220136910	63	0.023	1	1	2.655E-04	2.953E-03	PB-GT
NUP210L	nucleoporin 210kDa-like [Source:HGNC Symbol;Acc:29915]	chr1	153965161	154127592	847	0.300	3	1	2.661E-04	2.953E-03	PB-GT
TOB1	transducer of ERBB2, 1 [Source:HGNC Symbol;Acc:11979]	chr17	48939584	48945339	44	0.023	1	1	2.676E-04	2.953E-03	PB-GT
SHBG	sex hormone-binding globulin [Source:HGNC Symbol;Acc:10839]	chr17	7517382	7536700	44	0.023	1	1	2.676E-04	2.953E-03	PB-GT
EPM2AIP1	EPM2A (laforin) interacting protein 1 [Source:HGNC Symbol;Acc:19735]	chr3	37027357	37034795	71	0.023	1	1	2.677E-04	2.953E-03	PB-GT
TNNI1	troponin I type 1 (skeletal, slow) [Source:HGNC Symbol;Acc:11945]	chr1	201373625	201398994	66	0.023	1	1	2.692E-04	2.956E-03	PB-GT
C3orf38	chromosome 3 open reading frame 38 [Source:HGNC Symbol;Acc:28384]	chr3	88198893	88207118	73	0.024	1	1	2.829E-04	3.094E-03	PB-GT
CYTH4	cytohesin 4 [Source:HGNC Symbol;Acc:9505]	chr22	37678068	37711382	57	0.024	1	1	2.850E-04	3.104E-03	PB-GT
CHST6	carbohydrate (N-acetylglucosamine 6-O) sulfotransferase 6 [Source:HGNC Symbol;Acc:6938]	chr16	75510949	75529282	56	0.024	1	1	2.867E-04	3.109E-03	PB-GT
AATK	apoptosis-associated tyrosine kinase [Source:HGNC Symbol;Acc:21]	chr17	79091095	79139877	46	0.024	1	1	2.923E-04	3.147E-03	PB-GT
PDCD2	programmed cell death 2 [Source:HGNC Symbol;Acc:8762]	chr6	170884383	170893780	69	0.024	1	1	2.926E-04	3.147E-03	PB-GT
FGFR1OP2	FGFR1 oncogene partner 2 [Source:HGNC Symbol;Acc:23098]	chr12	27091316	27119583	284	0.125	2	2	2.988E-04	3.191E-03	PB-GT
RAB9A	RAB9A, member RAS oncogene family [Source:HGNC Symbol;Acc:9792]	chrX	13707244	13728625	148	0.025	1	1	2.991E-04	3.191E-03	PB-GT
CDCP2	CUB domain containing protein 2 [Source:HGNC Symbol;Acc:27297]	chr1	54598747	54619443	70	0.025	1	1	3.025E-04	3.214E-03	PB-GT
C3orf22	chromosome 3 open reading frame 22 [Source:HGNC Symbol;Acc:28534]	chr3	126245842	126277808	76	0.025	1	1	3.064E-04	3.243E-03	PB-GT
RIPK2	receptor-interacting serine-threonine kinase 2 [Source:HGNC Symbol;Acc:10020]	chr8	90769975	90803291	324	0.127	2	2	3.085E-04	3.251E-03	PB-GT
PLAC1L	placenta-specific 1-like [Source:HGNC Symbol;Acc:26699]	chr11	59807748	59815517	69	0.025	1	1	3.101E-04	3.254E-03	PB-GT
MSH3	mutS homolog 3 (E. coli) [Source:HGNC Symbol;Acc:7326]	chr5	79950467	80172279	1752	0.572	4	2	3.180E-04	3.316E-03	PB-GT
HIPK3	homeodomain interacting protein kinase 3 [Source:HGNC Symbol;Acc:4915]	chr11	33278218	33378569	865	0.315	3	1	3.185E-04	3.316E-03	PB-GT
SH3TC1	SH3 domain and tetratricopeptide repeats 1 [Source:HGNC Symbol;Acc:26009]	chr4	8183799	8242830	78	0.026	1	1	3.209E-04	3.328E-03	PB-GT
LINC00612	long intergenic non-protein coding RNA 612 [Source:HGNC Symbol;Acc:28621]	chr12	9208182	9217666	59	0.026	1	1	3.332E-04	3.441E-03	PB-GT
ORC6	origin recognition complex, subunit 6 [Source:HGNC Symbol;Acc:17151]	chr16	46723555	46732306	61	0.026	1	1	3.397E-04	3.481E-03	PB-GT
SLC7A5	solute carrier family 7 (amino acid transporter light chain, L system), member 5 [Source:HGNC Symbol;Acc:11063]	chr16	87863629	87903094	61	0.026	1	1	3.397E-04	3.481E-03	PB-GT
KRT39	keratin 39 [Source:HGNC Symbol;Acc:32971]	chr17	39114669	39123144	50	0.026	1	1	3.449E-04	3.521E-03	PB-GT
PTPRJ	protein tyrosine phosphatase, receptor type, J [Source:HGNC Symbol;Acc:9673]	chr11	48002113	48189670	887	0.323	3	1	3.499E-04	3.558E-03	PB-GT
LYSMD4	LysM, putative peptidoglycan-binding, domain containing 4 [Source:HGNC Symbol;Acc:26571]	chr15	100255902	100273766	73	0.027	1	1	3.538E-04	3.580E-03	PB-GT
C1orf106	chromosome 1 open reading frame 106 [Source:HGNC Symbol;Acc:25599]	chr1	200860176	200884863	76	0.027	1	1	3.561E-04	3.580E-03	PB-GT
TOLLIP	toll interacting protein [Source:HGNC Symbol;Acc:16476]	chr11	1295601	1330884	74	0.027	1	1	3.562E-04	3.580E-03	PB-GT
FOXO3	forkhead box O3 [Source:HGNC Symbol;Acc:3821]	chr6	108881038	109005977	920	0.325	3	2	3.596E-04	3.600E-03	PB-GT
FUS	fused in sarcoma [Source:HGNC Symbol;Acc:4010]	chr16	31191431	31203127	63	0.027	1	1	3.621E-04	3.611E-03	PB-GT
GNL3	guanine nucleotide binding protein-like 3 (nucleolar) [Source:HGNC Symbol;Acc:29931]	chr3	52715172	52728508	83	0.027	1	1	3.649E-04	3.619E-03	PB-GT
RPLP0P2	ribosomal protein, large, P0 pseudogene 2 [Source:HGNC Symbol;Acc:17960]	chr11	61382508	61406921	75	0.027	1	1	3.658E-04	3.619E-03	PB-GT
COPS5	COP9 signalosome subunit 5 [Source:HGNC Symbol;Acc:2240]	chr8	67955314	67996018	344	0.135	2	2	3.671E-04	3.619E-03	PB-GT
EXOSC8	exosome component 8 [Source:HGNC Symbol;Acc:17035]	chr13	37572953	37583750	114	0.028	1	1	3.719E-04	3.645E-03	PB-GT
BMS1	BMS1 homolog, ribosome assembly protein (yeast) [Source:HGNC Symbol;Acc:23505]	chr10	43278249	43326856	314	0.135	2	2	3.725E-04	3.645E-03	PB-GT
USP2	ubiquitin specific peptidase 2 [Source:HGNC Symbol;Acc:12618]	chr11	119225925	119252436	76	0.028	1	1	3.755E-04	3.661E-03	PB-GT
LPP	LIM domain containing preferred translocation partner in lipoma [Source:HGNC Symbol;Acc:6679]	chr3	187871072	188608460	5154	1.693	7	2	3.773E-04	3.664E-03	PB-GT
SLC44A3	solute carrier family 44, member 3 [Source:HGNC Symbol;Acc:28689]	chr1	95285898	95360802	387	0.137	2	2	3.878E-04	3.752E-03	PB-GT
SMG7-AS1	SMG7 antisense RNA 1 [Source:HGNC Symbol;Acc:24518]	chr1	183430009	183441400	80	0.028	1	1	3.942E-04	3.793E-03	PB-GT
CD8BP	CD8b molecule pseudogene [Source:HGNC Symbol;Acc:1708]	chr2	107103824	107123396	77	0.028	1	1	3.953E-04	3.793E-03	PB-GT
LINC00867	long intergenic non-protein coding RNA 867 [Source:HGNC Symbol;Acc:45265]	chr10	120116620	120124615	66	0.028	1	1	3.963E-04	3.793E-03	PB-GT
YWHAQ	tyrosine 3-monooxygenase/tryptophan 5-monooxygenase activation protein, theta polypeptide [Source:HGNC Symbol;Acc:12854]	chr2	9724101	9771143	376	0.139	2	1	4.001E-04	3.815E-03	PB-GT
ANXA3	annexin A3 [Source:HGNC Symbol;Acc:541]	chr4	79472673	79531597	426	0.140	2	1	4.081E-04	3.856E-03	PB-GT
MMRN2	multimerin 2 [Source:HGNC Symbol;Acc:19888]	chr10	88695297	88729238	67	0.029	1	1	4.083E-04	3.856E-03	PB-GT
PLEKHA4	pleckstrin homology domain containing, family A (phosphoinositide binding specific) member 4 [Source:HGNC Symbol;Acc:14339]	chr19	49340354	49371889	84	0.029	1	1	4.089E-04	3.856E-03	PB-GT
LSM5	LSM5 homolog, U6 small nuclear RNA associated (S. cerevisiae) [Source:HGNC Symbol;Acc:17162]	chr7	32524951	32534895	87	0.029	1	1	4.146E-04	3.894E-03	PB-GT
SATB2-AS1	SATB2 antisense RNA 1 [Source:HGNC Symbol;Acc:26490]	chr2	200322423	200341658	79	0.029	1	1	4.159E-04	3.894E-03	PB-GT
ZNF557	zinc finger protein 557 [Source:HGNC Symbol;Acc:28632]	chr19	7069455	7087979	85	0.029	1	1	4.186E-04	3.905E-03	PB-GT
PGAP2	post-GPI attachment to proteins 2 [Source:HGNC Symbol;Acc:17893]	chr11	3818954	3847601	81	0.029	1	1	4.260E-04	3.961E-03	PB-GT
HRH2	histamine receptor H2 [Source:HGNC Symbol;Acc:5183]	chr5	175085033	175113245	91	0.030	1	1	4.328E-04	4.009E-03	PB-GT
TMCO4	transmembrane and coiled-coil domains 4 [Source:HGNC Symbol;Acc:27393]	chr1	20008706	20126438	405	0.143	2	1	4.423E-04	4.083E-03	PB-GT
MVP	major vault protein [Source:HGNC Symbol;Acc:7531]	chr16	29831715	29859355	70	0.030	1	1	4.462E-04	4.098E-03	PB-GT
CD300LD	CD300 molecule-like family member d [Source:HGNC Symbol;Acc:16848]	chr17	72575504	72588422	57	0.030	1	1	4.471E-04	4.098E-03	PB-GT
DLGAP1	discs, large (Drosophila) homolog-associated protein 1 [Source:HGNC Symbol;Acc:2905]	chr18	3496030	4455335	6836	2.187	8	2	4.506E-04	4.106E-03	PB-GT
HARS	histidyl-tRNA synthetase [Source:HGNC Symbol;Acc:4816]	chr5	140052758	140071609	93	0.030	1	1	4.518E-04	4.106E-03	PB-GT
SLMO1	slowmo homolog 1 (Drosophila) [Source:HGNC Symbol;Acc:24639]	chr18	12407895	12432237	95	0.030	1	1	4.527E-04	4.106E-03	PB-GT
EIF2B1	eukaryotic translation initiation factor 2B, subunit 1 alpha, 26kDa [Source:HGNC Symbol;Acc:3257]	chr12	124104953	124118313	69	0.030	1	1	4.543E-04	4.106E-03	PB-GT
LRRIQ4	leucine-rich repeats and IQ motif containing 4 [Source:HGNC Symbol;Acc:34298]	chr3	169539710	169555563	94	0.031	1	1	4.669E-04	4.199E-03	PB-GT
ALOX5AP	arachidonate 5-lipoxygenase-activating protein [Source:HGNC Symbol;Acc:436]	chr13	31309645	31338556	128	0.031	1	1	4.678E-04	4.199E-03	PB-GT
UBAP1	ubiquitin associated protein 1 [Source:HGNC Symbol;Acc:12461]	chr9	34179003	34252521	447	0.146	2	2	4.696E-04	4.201E-03	PB-GT
ODAM	odontogenic, ameloblast asssociated [Source:HGNC Symbol;Acc:26043]	chr4	71062213	71070293	95	0.031	1	1	4.743E-04	4.221E-03	PB-GT
SSFA2	sperm specific antigen 2 [Source:HGNC Symbol;Acc:11319]	chr2	182756560	182795465	399	0.147	2	2	4.751E-04	4.221E-03	PB-GT
SH3KBP1	SH3-domain kinase binding protein 1 [Source:HGNC Symbol;Acc:13867]	chrX	19552093	19905719	2109	0.351	3	2	4.804E-04	4.242E-03	PB-GT
ALG10B	ALG10B, alpha-1,2-glucosyltransferase [Source:HGNC Symbol;Acc:31088]	chr12	38710380	38717784	71	0.031	1	1	4.808E-04	4.242E-03	PB-GT
SSH1	slingshot protein phosphatase 1 [Source:HGNC Symbol;Acc:30579]	chr12	109176466	109251366	336	0.148	2	1	4.864E-04	4.264E-03	PB-GT
ZNF639	zinc finger protein 639 [Source:HGNC Symbol;Acc:30950]	chr3	179040779	179053323	96	0.032	1	1	4.868E-04	4.264E-03	PB-GT
EIF5	eukaryotic translation initiation factor 5 [Source:HGNC Symbol;Acc:3299]	chr14	103799881	103811362	88	0.032	1	1	4.881E-04	4.264E-03	PB-GT
CLK1	CDC-like kinase 1 [Source:HGNC Symbol;Acc:2068]	chr2	201717732	201729422	86	0.032	1	1	4.920E-04	4.274E-03	PB-GT
ISM1	isthmin 1 homolog (zebrafish) [Source:HGNC Symbol;Acc:16213]	chr20	13202418	13281298	401	0.149	2	1	4.938E-04	4.274E-03	PB-GT
ALG10	ALG10, alpha-1,2-glucosyltransferase [Source:HGNC Symbol;Acc:23162]	chr12	34175216	34182629	72	0.032	1	1	4.943E-04	4.274E-03	PB-GT
ZFY-AS1	ZFY antisense RNA 1 [Source:HGNC Symbol;Acc:38798]	chrY	2834885	2870667	306	0.032	1	1	4.958E-04	4.274E-03	PB-GT
DONSON	downstream neighbor of SON [Source:HGNC Symbol;Acc:2993]	chr21	34947783	34961014	127	0.032	1	1	5.051E-04	4.331E-03	PB-GT
P2RX4	purinergic receptor P2X, ligand-gated ion channel, 4 [Source:HGNC Symbol;Acc:8535]	chr12	121647660	121671909	73	0.032	1	1	5.079E-04	4.331E-03	PB-GT
SRP9	signal recognition particle 9kDa [Source:HGNC Symbol;Acc:11304]	chr1	225965531	225978168	91	0.032	1	1	5.087E-04	4.331E-03	PB-GT
HIPK2	homeodomain interacting protein kinase 2 [Source:HGNC Symbol;Acc:14402]	chr7	139246316	139477577	1068	0.357	3	1	5.092E-04	4.331E-03	PB-GT
RBMX	RNA binding motif protein, X-linked [Source:HGNC Symbol;Acc:9910]	chrX	135930163	135962923	195	0.032	1	1	5.165E-04	4.379E-03	PB-GT
PADI3	peptidyl arginine deiminase, type III [Source:HGNC Symbol;Acc:18337]	chr1	17575593	17610728	92	0.033	1	1	5.198E-04	4.387E-03	PB-GT
KDM3B	lysine (K)-specific demethylase 3B [Source:HGNC Symbol;Acc:1337]	chr5	137688285	137772717	465	0.152	2	2	5.208E-04	4.387E-03	PB-GT
GABPB1-AS1	GABPB1 antisense RNA 1 [Source:HGNC Symbol;Acc:44157]	chr15	50647156	50659636	89	0.033	1	1	5.238E-04	4.398E-03	PB-GT
CUX1	cut-like homeobox 1 [Source:HGNC Symbol;Acc:2557]	chr7	101459219	101927250	1918	0.641	4	2	5.309E-04	4.443E-03	PB-GT
TEN1-CDK3	TEN1-CDK3 readthrough (non-protein coding) [Source:HGNC Symbol;Acc:44420]	chr17	73975312	74002080	63	0.033	1	1	5.450E-04	4.533E-03	PB-GT
KRT20	keratin 20 [Source:HGNC Symbol;Acc:20412]	chr17	39032193	39041479	63	0.033	1	1	5.450E-04	4.533E-03	PB-GT
CLEC12B	C-type lectin domain family 12, member B [Source:HGNC Symbol;Acc:31966]	chr12	10163226	10171218	76	0.034	1	1	5.501E-04	4.560E-03	PB-GT
RPS6KA1	ribosomal protein S6 kinase, 90kDa, polypeptide 1 [Source:HGNC Symbol;Acc:10430]	chr1	26856252	26901521	95	0.034	1	1	5.539E-04	4.577E-03	PB-GT
RBBP7	retinoblastoma binding protein 7 [Source:HGNC Symbol;Acc:9890]	chrX	16857406	16888537	203	0.034	1	1	5.593E-04	4.592E-03	PB-GT
SGOL1-AS1	SGOL1 antisense RNA 1 [Source:HGNC Symbol;Acc:41081]	chr3	20215736	20227919	103	0.034	1	1	5.595E-04	4.592E-03	PB-GT
ING5	inhibitor of growth family, member 5 [Source:HGNC Symbol;Acc:19421]	chr2	242641450	242668893	92	0.034	1	1	5.622E-04	4.592E-03	PB-GT

table S1. Genes with significant transposon integrations in DLD-1 *$KRAS^{wt/-}$* cells after selection in low glucose medium.

Gene ID	Description	Chrom	Start	End	# TTAA sites	Expected	Observed	Libraries	*P*	FDR	Transposon
HIST1H2AC	histone cluster 1, H2ac [Source:HGNC Symbol;Acc:4733]	chr6	26124373	26139344	96	0.034	1	1	5.628E-04	4.592E-03	PB-GT
PROSC	proline synthetase co-transcribed homolog (bacterial) [Source:HGNC Symbol;Acc:9457]	chr8	37620111	37637283	87	0.034	1	1	5.659E-04	4.604E-03	PB-GT
LINC00277	long intergenic non-protein coding RNA 277 [Source:HGNC Symbol;Acc:26596]	chr15	69365266	69388160	93	0.034	1	1	5.713E-04	4.633E-03	PB-GT
ZRANB1	zinc finger, RAN-binding domain containing 1 [Source:HGNC Symbol;Acc:18224]	chr10	126630692	126676758	366	0.158	2	2	5.801E-04	4.675E-03	PB-GT
C16orf80	chromosome 16 open reading frame 80 [Source:HGNC Symbol;Acc:29523]	chr16	58147496	58163354	80	0.034	1	1	5.811E-04	4.675E-03	PB-GT
CIB2	calcium and integrin binding family member 2 [Source:HGNC Symbol;Acc:24579]	chr15	78396948	78423886	94	0.035	1	1	5.835E-04	4.675E-03	PB-GT
TUBA4A	tubulin, alpha 4a [Source:HGNC Symbol;Acc:12407]	chr2	220114433	220142892	94	0.035	1	1	5.866E-04	4.675E-03	PB-GT
FAM122B	family with sequence similarity 122B [Source:HGNC Symbol;Acc:30490]	chrX	133903596	133931262	208	0.035	1	1	5.868E-04	4.675E-03	PB-GT
AGA	aspartylglucosaminidase [Source:HGNC Symbol;Acc:318]	chr4	178351924	178363657	106	0.035	1	1	5.891E-04	4.675E-03	PB-GT
ZNF783	zinc finger family member 783 [Source:HGNC Symbol;Acc:27222]	chr7	148959262	148994393	104	0.035	1	1	5.902E-04	4.675E-03	PB-GT
DMTF1	cyclin D binding myb-like transcription factor 1 [Source:HGNC Symbol;Acc:14603]	chr7	86781677	86825653	475	0.159	2	1	5.922E-04	4.675E-03	PB-GT
SLC30A10	solute carrier family 30, member 10 [Source:HGNC Symbol;Acc:25355]	chr1	219858769	220131989	1856	0.658	4	1	5.949E-04	4.675E-03	PB-GT
ZNF689	zinc finger protein 689 [Source:HGNC Symbol;Acc:25173]	chr16	30613879	30635333	81	0.035	1	1	5.955E-04	4.675E-03	PB-GT
MPZL2	myelin protein zero-like 2 [Source:HGNC Symbol;Acc:3496]	chr11	118124118	118135251	96	0.035	1	1	5.963E-04	4.675E-03	PB-GT
CUEDC1	CUE domain containing 1 [Source:HGNC Symbol;Acc:31350]	chr17	55938604	56032684	301	0.160	2	1	6.004E-04	4.688E-03	PB-GT
ADAM17	ADAM metallopeptidase domain 17 [Source:HGNC Symbol;Acc:195]	chr2	9628615	9695921	433	0.160	2	1	6.016E-04	4.688E-03	PB-GT
PDE8A	phosphodiesterase 8A [Source:HGNC Symbol;Acc:8793]	chr15	85523671	85682376	1017	0.374	3	1	6.049E-04	4.700E-03	PB-GT
MALAT1	metastasis associated lung adenocarcinoma transcript 1 (non-protein coding) [Source:HGNC Symbol;Acc:29665]	chr11	65265233	65273940	97	0.035	1	1	6.086E-04	4.715E-03	PB-GT
CPLX1	complexin 1 [Source:HGNC Symbol;Acc:2309]	chr4	778745	819986	108	0.035	1	1	6.113E-04	4.721E-03	PB-GT
LRRFIP1	leucine rich repeat (in FLII) interacting protein 1 [Source:HGNC Symbol;Acc:6702]	chr2	238536219	238722325	1018	0.375	3	1	6.132E-04	4.722E-03	PB-GT
CTSB	cathepsin B [Source:HGNC Symbol;Acc:2527]	chr8	11700033	11726957	92	0.036	1	1	6.320E-04	4.839E-03	PB-GT
TCP1	t-complex 1 [Source:HGNC Symbol;Acc:11655]	chr6	160199530	160210781	102	0.036	1	1	6.344E-04	4.839E-03	PB-GT
ZNF274	zinc finger protein 274 [Source:HGNC Symbol;Acc:13068]	chr19	58694396	58724928	105	0.036	1	1	6.358E-04	4.839E-03	PB-GT
ZFP28	ZFP28 zinc finger protein [Source:HGNC Symbol;Acc:17801]	chr19	57050317	57068169	105	0.036	1	1	6.358E-04	4.839E-03	PB-GT
MAP1LC3B2	microtubule-associated protein 1 light chain 3 beta 2 [Source:HGNC Symbol;Acc:34390]	chr12	116997186	117014425	83	0.037	1	1	6.547E-04	4.969E-03	PB-GT
SLC35E4	solute carrier family 35, member E4 [Source:HGNC Symbol;Acc:17058]	chr22	31031639	31065003	87	0.037	1	1	6.583E-04	4.981E-03	PB-GT
CCDC19	coiled-coil domain containing 19 [Source:HGNC Symbol;Acc:17229]	chr1	159842154	159869953	104	0.037	1	1	6.624E-04	4.983E-03	PB-GT
GJA9	gap junction protein, alpha 9, 59kDa [Source:HGNC Symbol;Acc:19155]	chr1	39330175	39347289	104	0.037	1	1	6.624E-04	4.983E-03	PB-GT
TGFB3	transforming growth factor, beta 3 [Source:HGNC Symbol;Acc:11769]	chr14	76424442	76449334	103	0.037	1	1	6.663E-04	4.998E-03	PB-GT
TCTE3	t-complex-associated-testis-expressed 3 [Source:HGNC Symbol;Acc:11695]	chr6	170140210	170151655	105	0.037	1	1	6.718E-04	5.022E-03	PB-GT
TBC1D10A	TBC1 domain family, member 10A [Source:HGNC Symbol;Acc:23609]	chr22	30687979	30723035	88	0.037	1	1	6.733E-04	5.022E-03	PB-GT
CHST10	carbohydrate sulfotransferase 10 [Source:HGNC Symbol;Acc:19650]	chr2	101008327	101034118	102	0.038	1	1	6.894E-04	5.117E-03	PB-GT
MED31	mediator complex subunit 31 [Source:HGNC Symbol;Acc:24260]	chr17	6546635	6554954	71	0.038	1	1	6.903E-04	5.117E-03	PB-GT
MGARP	mitochondria-localized glutamic acid-rich protein [Source:HGNC Symbol;Acc:29969]	chr4	140187317	140201492	115	0.038	1	1	6.920E-04	5.117E-03	PB-GT
ZNF317	zinc finger protein 317 [Source:HGNC Symbol;Acc:13507]	chr19	9251056	9274100	110	0.038	1	1	6.970E-04	5.139E-03	PB-GT
EML2	echinoderm microtubule associated protein like 2 [Source:HGNC Symbol;Acc:18035]	chr19	46110252	46148887	111	0.038	1	1	7.095E-04	5.200E-03	PB-GT
UBE2Z	ubiquitin-conjugating enzyme E2Z [Source:HGNC Symbol;Acc:25847]	chr17	46985731	47006418	72	0.038	1	1	7.096E-04	5.200E-03	PB-GT
CCT8	chaperonin containing TCP1, subunit 8 (theta) [Source:HGNC Symbol;Acc:1623]	chr21	30428126	30446118	151	0.038	1	1	7.111E-04	5.200E-03	PB-GT
SULT1C2	sulfotransferase family, cytosolic, 1C, member 2 [Source:HGNC Symbol;Acc:11456]	chr2	108905095	108926371	104	0.038	1	1	7.163E-04	5.223E-03	PB-GT
ACTR8	ARP8 actin-related protein 8 homolog (yeast) [Source:HGNC Symbol;Acc:14672]	chr3	53901093	53916229	118	0.039	1	1	7.320E-04	5.322E-03	PB-GT
FMO8P	flavin containing monooxygenase 8 pseudogene [Source:HGNC Symbol;Acc:32209]	chr1	166535415	166549942	110	0.039	1	1	7.400E-04	5.365E-03	PB-GT
ATG4B	autophagy related 4B, cysteine peptidase [Source:HGNC Symbol;Acc:20790]	chr2	242576628	242613272	107	0.039	1	1	7.577E-04	5.476E-03	PB-GT
TSC22D3	TSC22 domain family, member 3 [Source:HGNC Symbol;Acc:3051]	chrX	106956451	107020572	237	0.039	1	1	7.594E-04	5.476E-03	PB-GT
MYCBP	c-myc binding protein [Source:HGNC Symbol;Acc:7554]	chr1	39328636	39347289	112	0.040	1	1	7.668E-04	5.510E-03	PB-GT
HOXA-AS2	HOXA cluster antisense RNA 2 [Source:HGNC Symbol;Acc:43745]	chr7	27147396	27173921	119	0.040	1	1	7.702E-04	5.510E-03	PB-GT
IGSF3	immunoglobulin superfamily, member 3 [Source:HGNC Symbol;Acc:5950]	chr1	117117031	117210375	491	0.174	2	2	7.705E-04	5.510E-03	PB-GT
LPCAT1	lysophosphatidylcholine acyltransferase 1 [Source:HGNC Symbol;Acc:25718]	chr5	1456595	1524092	123	0.040	1	1	7.852E-04	5.600E-03	PB-GT
GPC5-AS1	GPC5 antisense RNA 1 [Source:HGNC Symbol;Acc:39886]	chr13	93353642	93373867	167	0.040	1	1	7.913E-04	5.628E-03	PB-GT
LZIC	leucine zipper and CTNNBIP1 domain containing [Source:HGNC Symbol;Acc:17497]	chr1	9982173	10003465	114	0.040	1	1	7.940E-04	5.632E-03	PB-GT
CAPN1	calpain 1, (mu/l) large subunit [Source:HGNC Symbol;Acc:1476]	chr11	64948037	64979477	112	0.041	1	1	8.084E-04	5.718E-03	PB-GT
IAH1	isoamyl acetate-hydrolyzing esterase 1 homolog (S. cerevisiae) [Source:HGNC Symbol;Acc:27696]	chr2	9613787	9636672	112	0.041	1	1	8.292E-04	5.835E-03	PB-GT
TMEM8B	transmembrane protein 8B [Source:HGNC Symbol;Acc:21427]	chr9	35814448	35854844	126	0.041	1	1	8.295E-04	5.835E-03	PB-GT
EMP1	epithelial membrane protein 1 [Source:HGNC Symbol;Acc:3333]	chr12	13349650	13369708	94	0.041	1	1	8.370E-04	5.873E-03	PB-GT
GGPS1	geranylgeranyl diphosphate synthase 1 [Source:HGNC Symbol;Acc:4249]	chr1	235490665	235507847	118	0.042	1	1	8.499E-04	5.947E-03	PB-GT
KREMEN1	kringle containing transmembrane protein 1 [Source:HGNC Symbol;Acc:17550]	chr22	29469066	29564321	428	0.181	2	2	8.593E-04	5.953E-03	PB-GT
VIPR1	vasoactive intestinal peptide receptor 1 [Source:HGNC Symbol;Acc:12694]	chr3	42530791	42579059	128	0.042	1	1	8.594E-04	5.953E-03	PB-GT
C11orf65	chromosome 11 open reading frame 65 [Source:HGNC Symbol;Acc:28519]	chr11	108179246	108338258	1130	0.411	3	2	8.596E-04	5.953E-03	PB-GT
CHST12	carbohydrate (chondroitin 4) sulfotransferase 12 [Source:HGNC Symbol;Acc:17423]	chr7	2443223	2474242	126	0.042	1	1	8.621E-04	5.953E-03	PB-GT
CPA4	carboxypeptidase A4 [Source:HGNC Symbol;Acc:15740]	chr7	129932974	129964020	126	0.042	1	1	8.621E-04	5.953E-03	PB-GT
DDX11	DEAD/H (Asp-Glu-Ala-Asp/His) box helicase 11 [Source:HGNC Symbol;Acc:2736]	chr12	31226779	31257725	96	0.042	1	1	8.725E-04	6.008E-03	PB-GT
SLC1A3	solute carrier family 1 (glial high affinity glutamate transporter), member 3 [Source:HGNC Symbol;Acc:10941]	chr5	36606457	36688436	557	0.182	2	2	8.753E-04	6.012E-03	PB-GT
THSD4	thrombospondin, type I, domain containing 4 [Source:HGNC Symbol;Acc:25835]	chr15	71389291	72075722	4043	1.486	6	2	8.788E-04	6.020E-03	PB-GT
SNRNP200	small nuclear ribonucleoprotein 200kDa (U5) [Source:HGNC Symbol;Acc:30859]	chr2	96940074	96971297	116	0.043	1	1	8.886E-04	6.071E-03	PB-GT
NOSTRIN	nitric oxide synthase trafficker [Source:HGNC Symbol;Acc:20203]	chr2	169643049	169722024	497	0.183	2	1	8.939E-04	6.091E-03	PB-GT
RGS12	regulator of G-protein signaling 12 [Source:HGNC Symbol;Acc:9994]	chr4	3294755	3441640	561	0.184	2	2	9.018E-04	6.129E-03	PB-GT
ZNF607	zinc finger protein 607 [Source:HGNC Symbol;Acc:28192]	chr19	38187264	38210691	126	0.043	1	1	9.111E-04	6.176E-03	PB-GT
RNF139	ring finger protein 139 [Source:HGNC Symbol;Acc:17023]	chr8	125486979	125500155	111	0.043	1	1	9.155E-04	6.186E-03	PB-GT
SUN2	Sad1 and UNC84 domain containing 2 [Source:HGNC Symbol;Acc:14210]	chr22	39130730	39190148	103	0.043	1	1	9.186E-04	6.186E-03	PB-GT
SERPINB13	serpin peptidase inhibitor, clade B (ovalbumin), member 13 [Source:HGNC Symbol;Acc:8944]	chr18	61254223	61271873	136	0.044	1	1	9.198E-04	6.186E-03	PB-GT
COMMD6	COMM domain containing 6 [Source:HGNC Symbol;Acc:24015]	chr13	76099350	76123575	181	0.044	1	1	9.275E-04	6.222E-03	PB-GT
LINC00426	long intergenic non-protein coding RNA 426 [Source:HGNC Symbol;Acc:42761]	chr13	30914407	30951282	183	0.044	1	1	9.478E-04	6.342E-03	PB-GT
SPINT2	serine peptidase inhibitor, Kunitz type, 2 [Source:HGNC Symbol;Acc:11247]	chr19	38734675	38783254	129	0.044	1	1	9.544E-04	6.370E-03	PB-GT
COL18A1	collagen, type XVIII, alpha 1 [Source:HGNC Symbol;Acc:2195]	chr21	46825052	46933634	177	0.045	1	1	9.729E-04	6.477E-03	PB-GT
NRL	neural retina leucine zipper [Source:HGNC Symbol;Acc:8002]	chr14	24549316	24584223	125	0.045	1	1	9.762E-04	6.482E-03	PB-GT
LINC00422	long intergenic non-protein coding RNA 422 [Source:HGNC Symbol;Acc:42757]	chr13	21877651	21922860	186	0.045	1	1	9.787E-04	6.482E-03	PB-GT
CNIH4	cornichon homolog 4 (Drosophila) [Source:HGNC Symbol;Acc:25013]	chr1	224544552	224567161	127	0.045	1	1	9.824E-04	6.490E-03	PB-GT
CDKN2C	cyclin-dependent kinase inhibitor 2C (p18, inhibits CDK4) [Source:HGNC Symbol;Acc:1789]	chr1	51426417	51440305	128	0.045	1	1	9.977E-04	6.575E-03	PB-GT
HNRNPA3	heterogeneous nuclear ribonucleoprotein A3 [Source:HGNC Symbol;Acc:24941]	chr2	178077291	178088686	124	0.046	1	1	1.013E-03	6.661E-03	PB-GT
XG	Xg blood group [Source:HGNC Symbol;Acc:12806]	chrX	2670091	2734539	275	0.046	1	1	1.018E-03	6.676E-03	PB-GT
ZNF775	zinc finger protein 775 [Source:HGNC Symbol;Acc:28501]	chr7	150065879	150109558	138	0.046	1	1	1.031E-03	6.721E-03	PB-GT
SYTL3	synaptotagmin-like 3 [Source:HGNC Symbol;Acc:15587]	chr6	159071046	159185908	545	0.193	2	2	1.032E-03	6.721E-03	PB-GT
C8orf76	chromosome 8 open reading frame 76 [Source:HGNC Symbol;Acc:25924]	chr8	124232196	124253638	118	0.046	1	1	1.033E-03	6.721E-03	PB-GT
ST14	suppression of tumorigenicity 14 (colon carcinoma) [Source:HGNC Symbol;Acc:11344]	chr11	130029457	130080271	127	0.046	1	1	1.036E-03	6.723E-03	PB-GT
UPF2	UPF2 regulator of nonsense transcripts homolog (yeast) [Source:HGNC Symbol;Acc:17854]	chr10	11962021	12085169	1006	0.433	3	2	1.041E-03	6.737E-03	PB-GT
SARDH	sarcosine dehydrogenase [Source:HGNC Symbol;Acc:10536]	chr9	136528682	136605077	142	0.047	1	1	1.050E-03	6.766E-03	PB-GT
RBM12B	RNA binding motif protein 12B [Source:HGNC Symbol;Acc:32310]	chr8	94741584	94753245	119	0.047	1	1	1.050E-03	6.766E-03	PB-GT
HLF	hepatic leukemia factor [Source:HGNC Symbol;Acc:4977]	chr17	53342373	53402426	367	0.194	2	2	1.060E-03	6.815E-03	PB-GT
ADH5	alcohol dehydrogenase 5 (class III), chi polypeptide [Source:HGNC Symbol;Acc:253]	chr4	99992132	100009952	144	0.047	1	1	1.078E-03	6.913E-03	PB-GT
PPIL2	peptidylprolyl isomerase (cyclophilin)-like 2 [Source:HGNC Symbol;Acc:9261]	chr22	22006559	22052565	112	0.047	1	1	1.083E-03	6.929E-03	PB-GT
ADH1B	alcohol dehydrogenase 1B (class I), beta polypeptide [Source:HGNC Symbol;Acc:250]	chr4	100226121	100242558	145	0.047	1	1	1.093E-03	6.973E-03	PB-GT
LRRC42	leucine rich repeat containing 42 [Source:HGNC Symbol;Acc:28792]	chr1	54411750	54433841	135	0.048	1	1	1.108E-03	7.052E-03	PB-GT
SETD9	SET domain containing 9 [Source:HGNC Symbol;Acc:28508]	chr5	56205087	56221359	147	0.048	1	1	1.116E-03	7.053E-03	PB-GT
NEAT1	nuclear paraspeckle assembly transcript 1 (non-protein coding) [Source:HGNC Symbol;Acc:30815]	chr11	65190245	65213011	132	0.048	1	1	1.118E-03	7.053E-03	PB-GT
ZNF184	zinc finger protein 184 [Source:HGNC Symbol;Acc:12975]	chr6	27418522	27440897	136	0.048	1	1	1.119E-03	7.053E-03	PB-GT
GUSBP5	glucuronidase, beta pseudogene 5 [Source:HGNC Symbol;Acc:42319]	chr4	144480625	144570458	605	0.198	2	2	1.119E-03	7.053E-03	PB-GT
SHROOM3	shroom family member 3 [Source:HGNC Symbol;Acc:30422]	chr4	77356253	77704406	2319	0.760	4	2	1.126E-03	7.067E-03	PB-GT
MAP4K5	mitogen-activated protein kinase kinase kinase kinase 5 [Source:HGNC Symbol;Acc:6867]	chr14	50885219	51027844	1234	0.443	3	2	1.127E-03	7.067E-03	PB-GT
LINC00402	long intergenic non-protein coding RNA 402 [Source:HGNC Symbol;Acc:42732]	chr13	74805594	74834113	201	0.049	1	1	1.140E-03	7.134E-03	PB-GT
CYTH3	cytohesin 3 [Source:HGNC Symbol;Acc:9504]	chr7	6201407	6312275	598	0.200	2	1	1.146E-03	7.152E-03	PB-GT
SLC10A6	solute carrier family 10 (sodium/bile acid cotransporter family), member 6 [Source:HGNC Symbol;Acc:30603]	chr4	87744621	87770416	149	0.049	1	1	1.153E-03	7.152E-03	PB-GT
RAI2	retinoic acid induced 2 [Source:HGNC Symbol;Acc:9835]	chrX	17818169	17879457	293	0.049	1	1	1.154E-03	7.152E-03	PB-GT
MICU1	mitochondrial calcium uptake 1 [Source:HGNC Symbol;Acc:1530]	chr10	74127098	74385899	1774	0.764	4	2	1.154E-03	7.152E-03	PB-GT
SCARA3	scavenger receptor class A, member 3 [Source:HGNC Symbol;Acc:19000]	chr8	27491385	27534293	125	0.049	1	1	1.157E-03	7.152E-03	PB-GT
MAP1LC3B	microtubule-associated protein 1 light chain 3 beta [Source:HGNC Symbol;Acc:13352]	chr16	87417601	87438385	114	0.049	1	1	1.169E-03	7.208E-03	PB-GT
FRMD5	FERM domain containing 5 [Source:HGNC Symbol;Acc:28214]	chr15	44162962	44487450	2088	0.768	4	2	1.179E-03	7.248E-03	PB-GT
PISD	phosphatidylserine decarboxylase [Source:HGNC Symbol;Acc:8999]	chr22	32014477	32058418	117	0.049	1	1	1.181E-03	7.248E-03	PB-GT
PHAX	phosphorylated adaptor for RNA export [Source:HGNC Symbol;Acc:10241]	chr5	125935960	125962944	152	0.050	1	1	1.192E-03	7.281E-03	PB-GT
PHF15	PHD finger protein 15 [Source:HGNC Symbol;Acc:22984]	chr5	133860003	133918918	152	0.050	1	1	1.192E-03	7.281E-03	PB-GT
TCEA3	transcription elongation factor A (SII), 3 [Source:HGNC Symbol;Acc:11615]	chr1	23707554	23751233	141	0.050	1	1	1.207E-03	7.358E-03	PB-GT
TMEM67	transmembrane protein 67 [Source:HGNC Symbol;Acc:28396]	chr8	94767072	94831462	523	0.205	2	2	1.225E-03	7.447E-03	PB-GT
WISP3	WNT1 inducible signaling pathway protein 3 [Source:HGNC Symbol;Acc:12771]	chr6	112375275	112392171	143	0.051	1	1	1.235E-03	7.493E-03	PB-GT
EFCAB14-AS1	EFCAB14 antisense RNA 1 [Source:HGNC Symbol;Acc:44108]	chr1	47139708	47157770	144	0.051	1	1	1.258E-03	7.598E-03	PB-GT
MORN1	MORN repeat containing 1 [Source:HGNC Symbol;Acc:25852]	chr1	2252692	2323146	144	0.051	1	1	1.258E-03	7.598E-03	PB-GT
CD44	CD44 molecule (Indian blood group) [Source:HGNC Symbol;Acc:1681]	chr11	35160417	35253949	569	0.207	2	1	1.268E-03	7.640E-03	PB-GT
FAM111B	family with sequence similarity 111, member B [Source:HGNC Symbol;Acc:24200]	chr11	58874658	58894883	141	0.051	1	1	1.272E-03	7.649E-03	PB-GT
TRPM8	transient receptor potential cation channel, subfamily M, member 8 [Source:HGNC Symbol;Acc:17961]	chr2	234826043	234928166	563	0.208	2	1	1.276E-03	7.654E-03	PB-GT
CIT	citron (rho-interacting, serine/threonine kinase 21) [Source:HGNC Symbol;Acc:1985]	chr12	120123595	120315095	1040	0.459	3	1	1.284E-03	7.664E-03	PB-GT
ZFYVE20	zinc finger, FYVE domain containing 20 [Source:HGNC Symbol;Acc:20759]	chr3	15111580	15140670	157	0.052	1	1	1.285E-03	7.664E-03	PB-GT
MTHFD2	methylenetetrahydrofolate dehydrogenase (NADP+ dependent) 2, methenyltetrahydrofolate cyclohydrolase [Source:HGNC Symbol;Acc:7434]	chr2	74425689	74444692	140	0.052	1	1	1.287E-03	7.664E-03	PB-GT
FOXP4	forkhead box P4 [Source:HGNC Symbol;Acc:20842]	chr6	41514164	41570122	147	0.052	1	1	1.304E-03	7.747E-03	PB-GT
ARMCX5	armadillo repeat containing, X-linked 5 [Source:HGNC Symbol;Acc:25772]	chrX	101854096	101905073	314	0.052	1	1	1.322E-03	7.747E-03	PB-GT
PSD4	pleckstrin and Sec7 domain containing 4 [Source:HGNC Symbol;Acc:19096]	chr2	113914902	113960814	142	0.052	1	1	1.323E-03	7.747E-03	PB-GT
POLDIP3	polymerase (DNA-directed), delta interacting protein 3 [Source:HGNC Symbol;Acc:23782]	chr22	42979727	43010968	124	0.052	1	1	1.324E-03	7.747E-03	PB-GT
MYO3B	myosin IIIB [Source:HGNC Symbol;Acc:15576]	chr2	171034655	171511681	3175	1.170	5	2	1.324E-03	7.747E-03	PB-GT
ALDH6A1	aldehyde dehydrogenase 6 family, member A1 [Source:HGNC Symbol;Acc:7179]	chr14	74523553	74551196	146	0.052	1	1	1.325E-03	7.747E-03	PB-GT
PFDN4	prefoldin subunit 4 [Source:HGNC Symbol;Acc:8868]	chr20	52824386	52844591	141	0.052	1	1	1.326E-03	7.747E-03	PB-GT
DHRS3	dehydrogenase/reductase (SDR family) member 3 [Source:HGNC Symbol;Acc:17693]	chr1	12627939	12677737	148	0.052	1	1	1.328E-03	7.747E-03	PB-GT
ARRDC3	arrestin domain containing 3 [Source:HGNC Symbol;Acc:29263]	chr5	90664541	90679176	161	0.053	1	1	1.334E-03	7.747E-03	PB-GT
SIGLEC5	sialic acid binding Ig-like lectin 5 [Source:HGNC Symbol;Acc:10874]	chr19	52114781	52150151	153	0.053	1	1	1.335E-03	7.747E-03	PB-GT
CPOX	coproporphyrinogen oxidase [Source:HGNC Symbol;Acc:2321]	chr3	98239976	98312567	643	0.211	2	2	1.341E-03	7.747E-03	PB-GT
PRRX2	paired related homeobox 2 [Source:HGNC Symbol;Acc:21338]	chr9	132427920	132484875	161	0.053	1	1	1.344E-03	7.747E-03	PB-GT
RPSAP52	ribosomal protein SA pseudogene 52 [Source:HGNC Symbol;Acc:35752]	chr12	66151800	66220777	479	0.211	2	2	1.345E-03	7.747E-03	PB-GT
COMMD7	COMM domain containing 7 [Source:HGNC Symbol;Acc:16223]	chr20	31290493	31331814	142	0.053	1	1	1.345E-03	7.747E-03	PB-GT
KIAA0907	KIAA0907 [Source:HGNC Symbol;Acc:29145]	chr1	155882834	155904191	149	0.053	1	1	1.345E-03	7.747E-03	PB-GT
TRPM2	transient receptor potential cation channel, subfamily M, member 2 [Source:HGNC Symbol;Acc:12339]	chr21	45770046	45862964	209	0.053	1	1	1.349E-03	7.753E-03	PB-GT
SAMD8	sterile alpha motif domain containing 8 [Source:HGNC Symbol;Acc:26320]	chr10	76859344	76941881	492	0.212	2	1	1.353E-03	7.754E-03	PB-GT
MXRA7	matrix-remodelling associated 7 [Source:HGNC Symbol;Acc:7541]	chr17	74668633	74707098	100	0.053	1	1	1.355E-03	7.754E-03	PB-GT
TPTE2P6	transmembrane phosphoinositide 3-phosphatase and tensin homolog 2 pseudogene 6 [Source:HGNC Symbol;Acc:42644]	chr13	25141011	25171814	220	0.053	1	1	1.362E-03	7.773E-03	PB-GT
PCED1B	PC-esterase domain containing 1B [Source:HGNC Symbol;Acc:28255]	chr12	47473386	47630443	1060	0.468	3	2	1.377E-03	7.841E-03	PB-GT
MAGI2-AS3	MAGI2 antisense RNA 3 [Source:HGNC Symbol;Acc:40862]	chr7	79082198	79100524	161	0.054	1	1	1.397E-03	7.938E-03	PB-GT
AACSP1	acetoacetyl-CoA synthetase pseudogene 1 [Source:HGNC Symbol;Acc:18226]	chr5	178191862	178245436	165	0.054	1	1	1.400E-03	7.941E-03	PB-GT
PER2	period circadian clock 2 [Source:HGNC Symbol;Acc:8846]	chr2	239152679	239198743	147	0.054	1	1	1.416E-03	7.984E-03	PB-GT
WDR82	WD repeat domain 82 [Source:HGNC Symbol;Acc:28826]	chr3	52288437	52322036	165	0.054	1	1	1.417E-03	7.984E-03	PB-GT
CALR4P	calreticulin 4, pseudogene [Source:HGNC Symbol;Acc:35456]	chr1	52027538	52060569	153	0.054	1	1	1.417E-03	7.984E-03	PB-GT
PAIP2	poly(A) binding protein interacting protein 2 [Source:HGNC Symbol;Acc:17970]	chr5	138677276	138705406	167	0.055	1	1	1.434E-03	8.061E-03	PB-GT
NR2C2	nuclear receptor subfamily 2, group C, member 2 [Source:HGNC Symbol;Acc:7972]	chr3	14989091	15095107	660	0.217	2	2	1.444E-03	8.103E-03	PB-GT
FRYL	FRY-like [Source:HGNC Symbol;Acc:29127]	chr4	48499378	48782339	2457	0.805	4	1	1.449E-03	8.103E-03	PB-GT
SETD5	SET domain containing 5 [Source:HGNC Symbol;Acc:25566]	chr3	9439299	9520924	661	0.217	2	1	1.451E-03	8.103E-03	PB-GT
DCAF12	DDB1 and CUL4 associated factor 12 [Source:HGNC Symbol;Acc:19911]	chr9	34086385	34127397	168	0.055	1	1	1.461E-03	8.144E-03	PB-GT
CHST3	carbohydrate (chondroitin 6) sulfotransferase 3 [Source:HGNC Symbol;Acc:1971]	chr10	73724123	73773322	128	0.055	1	1	1.465E-03	8.146E-03	PB-GT
E2F6	E2F transcription factor 6 [Source:HGNC Symbol;Acc:3120]	chr2	11584501	11606297	150	0.055	1	1	1.473E-03	8.173E-03	PB-GT
SLC7A9	solute carrier family 7 (glycoprotein-associated amino acid transporter light chain, bo,+ system), member 9 [Source:HGNC Symbol;Acc:11067]	chr19	33321415	33360672	161	0.055	1	1	1.476E-03	8.173E-03	PB-GT
FCER1A	Fc fragment of IgE, high affinity I, receptor for; alpha polypeptide [Source:HGNC Symbol;Acc:3609]	chr1	159259504	159278014	158	0.056	1	1	1.510E-03	8.324E-03	PB-GT
SRPK1	SRSF protein kinase 1 [Source:HGNC Symbol;Acc:11305]	chr6	35800743	35889119	623	0.220	2	1	1.510E-03	8.324E-03	PB-GT
C16orf46	chromosome 16 open reading frame 46 [Source:HGNC Symbol;Acc:26525]	chr16	81087102	81110872	130	0.056	1	1	1.513E-03	8.324E-03	PB-GT
GPR37	G protein-coupled receptor 37 (endothelin receptor type B-like) [Source:HGNC Symbol;Acc:4494]	chr7	124386051	124405681	169	0.056	1	1	1.536E-03	8.421E-03	PB-GT
ALDH2	aldehyde dehydrogenase 2 family (mitochondrial) [Source:HGNC Symbol;Acc:404]	chr12	112204691	112247782	128	0.056	1	1	1.537E-03	8.421E-03	PB-GT
DAD1	defender against cell death 1 [Source:HGNC Symbol;Acc:2664]	chr14	23033805	23058175	158	0.057	1	1	1.547E-03	8.450E-03	PB-GT
TRIM35	tripartite motif containing 35 [Source:HGNC Symbol;Acc:16285]	chr8	27142404	27168836	145	0.057	1	1	1.549E-03	8.450E-03	PB-GT

table S1. Genes with significant transposon integrations in DLD-1 *KRAS*$^{wt/-}$ cells after selection in low glucose medium.

Gene ID	Description	Chrom	Start	End	# TTAA sites	Expected	Observed	Libraries	*P*	FDR	Transposon
HOXC6	homeobox C6 [Source:HGNC Symbol;Acc:5128]	chr12	54384408	54424607	129	0.057	1	1	1.560E-03	8.497E-03	PB-GT
PTEN	phosphatase and tensin homolog [Source:HGNC Symbol;Acc:9588]	chr10	89622870	89731687	1128	0.486	3	1	1.578E-03	8.526E-03	PB-GT
PRKCH	protein kinase C, eta [Source:HGNC Symbol;Acc:9403]	chr14	61654277	62017694	2288	0.821	4	1	1.579E-03	8.526E-03	PB-GT
MKI67	antigen identified by monoclonal antibody Ki-67 [Source:HGNC Symbol;Acc:7107]	chr10	129894923	129924649	133	0.057	1	1	1.579E-03	8.526E-03	PB-GT
SPIRE1	spire homolog 1 (Drosophila) [Source:HGNC Symbol;Acc:30622]	chr18	12446511	12658133	1520	0.486	3	2	1.585E-03	8.526E-03	PB-GT
C1orf198	chromosome 1 open reading frame 198 [Source:HGNC Symbol;Acc:25900]	chr1	230972865	231005335	162	0.057	1	1	1.585E-03	8.526E-03	PB-GT
RUNX3	runt-related transcription factor 3 [Source:HGNC Symbol;Acc:10473]	chr1	25226002	25291612	162	0.057	1	1	1.585E-03	8.526E-03	PB-GT
TBCCD1	TBCC domain containing 1 [Source:HGNC Symbol;Acc:25546]	chr3	186263862	186288332	176	0.058	1	1	1.608E-03	8.613E-03	PB-GT
ITFG2	integrin alpha FG-GAP repeat containing 2 [Source:HGNC Symbol;Acc:30879]	chr12	2921788	2968957	131	0.058	1	1	1.608E-03	8.613E-03	PB-GT
ERCC1	excision repair cross-complementing rodent repair deficiency, complementation group 1 (includes overlapping antisense sequence) [Source:HGNC Symbol;A	chr19	45910591	45982086	169	0.058	1	1	1.623E-03	8.673E-03	PB-GT
THUMPD3	THUMP domain containing 3 [Source:HGNC Symbol;Acc:24493]	chr3	9404526	9428475	177	0.058	1	1	1.626E-03	8.673E-03	PB-GT
ZBED4	zinc finger, BED-type containing 4 [Source:HGNC Symbol;Acc:20721]	chr22	50247490	50282090	138	0.058	1	1	1.633E-03	8.690E-03	PB-GT
NPSR1	neuropeptide S receptor 1 [Source:HGNC Symbol;Acc:23631]	chr7	34697851	34917944	1468	0.491	3	2	1.636E-03	8.690E-03	PB-GT
NKAIN1	Na+/K+ transporting ATPase interacting 1 [Source:HGNC Symbol;Acc:25743]	chr1	31652592	31712401	165	0.058	1	1	1.644E-03	8.713E-03	PB-GT
NAAA	N-acylethanolamine acid amidase [Source:HGNC Symbol;Acc:736]	chr4	76831809	76862204	179	0.059	1	1	1.653E-03	8.748E-03	PB-GT
TMEM207	transmembrane protein 207 [Source:HGNC Symbol;Acc:33705]	chr3	190146444	190167665	179	0.059	1	1	1.662E-03	8.773E-03	PB-GT
C14orf166	chromosome 14 open reading frame 166 [Source:HGNC Symbol;Acc:23169]	chr14	52456193	52471420	164	0.059	1	1	1.665E-03	8.773E-03	PB-GT
PVT1	Pvt1 oncogene (non-protein coding) [Source:HGNC Symbol;Acc:9709]	chr8	128806779	129113499	1263	0.494	3	2	1.677E-03	8.816E-03	PB-GT
GFPT2	glutamine-fructose-6-phosphate transaminase 2 [Source:HGNC Symbol;Acc:4242]	chr5	179727690	179780387	182	0.059	1	1	1.697E-03	8.888E-03	PB-GT
FANCD2P2	Fanconi anemia, complementation group D2 pseudogene 2 [Source:HGNC Symbol;Acc:44488]	chr3	11901148	11932646	181	0.059	1	1	1.699E-03	8.888E-03	PB-GT
SMPX	small muscle protein, X-linked [Source:HGNC Symbol;Acc:11122]	chrX	21724090	21776281	357	0.059	1	1	1.700E-03	8.888E-03	PB-GT
PKM	pyruvate kinase, muscle [Source:HGNC Symbol;Acc:9021]	chr15	72491370	72524164	162	0.060	1	1	1.705E-03	8.892E-03	PB-GT
EIF2S2	eukaryotic translation initiation factor 2, subunit 2 beta, 38kDa [Source:HGNC Symbol;Acc:3266]	chr20	32676104	32700138	161	0.060	1	1	1.721E-03	8.959E-03	PB-GT
GALNT6	UDP-N-acetyl-alpha-D-galactosamine:polypeptide N-acetylgalactosaminyltransferase 6 (GalNAc-T6) [Source:HGNC Symbol;Acc:4128]	chr12	51745031	51786651	136	0.060	1	1	1.731E-03	8.992E-03	PB-GT
GEMIN6	gem (nuclear organelle) associated protein 6 [Source:HGNC Symbol;Acc:20044]	chr2	38978676	39009598	164	0.060	1	1	1.755E-03	9.085E-03	PB-GT
CDC42EP3	CDC42 effector protein (Rho GTPase binding) 3 [Source:HGNC Symbol;Acc:16943]	chr2	37869032	37965611	630	0.232	2	2	1.756E-03	9.085E-03	PB-GT
GTF2H5	general transcription factor IIH, polypeptide 5 [Source:HGNC Symbol;Acc:21157]	chr6	158589384	158615020	172	0.061	1	1	1.775E-03	9.154E-03	PB-GT
MDH1B	malate dehydrogenase 1B, NAD (soluble) [Source:HGNC Symbol;Acc:17836]	chr2	207602487	207630271	165	0.061	1	1	1.776E-03	9.154E-03	PB-GT
UQCC	ubiquinol-cytochrome c reductase complex chaperone [Source:HGNC Symbol;Acc:15891]	chr20	33890369	33999944	628	0.233	2	2	1.781E-03	9.154E-03	PB-GT
YBX1	Y box binding protein 1 [Source:HGNC Symbol;Acc:8014]	chr1	43148098	43168020	172	0.061	1	1	1.783E-03	9.154E-03	PB-GT
TMEM64	transmembrane protein 64 [Source:HGNC Symbol;Acc:25441]	chr8	91634223	91803860	1291	0.505	3	2	1.815E-03	9.298E-03	PB-GT
LYPD1	LY6/PLAUR domain containing 1 [Source:HGNC Symbol;Acc:28431]	chr2	133402426	133429152	168	0.062	1	1	1.840E-03	9.410E-03	PB-GT
NMNAT1	nicotinamide nucleotide adenylyltransferase 1 [Source:HGNC Symbol;Acc:17877]	chr1	10003486	10045559	175	0.062	1	1	1.844E-03	9.410E-03	PB-GT
MPHOSPH8	M-phase phosphoprotein 8 [Source:HGNC Symbol;Acc:29810]	chr13	20207788	20247599	257	0.062	1	1	1.847E-03	9.410E-03	PB-GT
AMICA1	adhesion molecule, interacts with CXADR antigen 1 [Source:HGNC Symbol;Acc:19084]	chr11	118064455	118095809	171	0.062	1	1	1.858E-03	9.445E-03	PB-GT
UMODL1	uromodulin-like 1 [Source:HGNC Symbol;Acc:12560]	chr21	43483068	43563105	247	0.062	1	1	1.872E-03	9.497E-03	PB-GT
FAM149B1	family with sequence similarity 149, member B1 [Source:HGNC Symbol;Acc:29162]	chr10	74927924	75004262	552	0.238	2	1	1.875E-03	9.497E-03	PB-GT
TMX2	thioredoxin-related transmembrane protein 2 [Source:HGNC Symbol;Acc:30739]	chr11	57480072	57508445	172	0.063	1	1	1.879E-03	9.498E-03	PB-GT
YARS	tyrosyl-tRNA synthetase [Source:HGNC Symbol;Acc:12840]	chr1	33240840	33283754	177	0.063	1	1	1.886E-03	9.501E-03	PB-GT
NAA25	N(alpha)-acetyltransferase 25, NatB auxiliary subunit [Source:HGNC Symbol;Acc:25783]	chr12	112464500	112546826	540	0.238	2	2	1.889E-03	9.501E-03	PB-GT
MIA2	melanoma inhibitory activity 2 [Source:HGNC Symbol;Acc:18432]	chr14	39699435	39722859	175	0.063	1	1	1.891E-03	9.501E-03	PB-GT
IFNAR1	interferon (alpha, beta and omega) receptor 1 [Source:HGNC Symbol;Acc:5432]	chr21	34696734	34732168	249	0.063	1	1	1.902E-03	9.528E-03	PB-GT
ATXN10	ataxin 10 [Source:HGNC Symbol;Acc:10549]	chr22	46067678	46241187	1212	0.512	3	1	1.904E-03	9.528E-03	PB-GT
MFAP5	microfibrillar associated protein 5 [Source:HGNC Symbol;Acc:29673]	chr12	8789942	8815484	143	0.063	1	1	1.910E-03	9.528E-03	PB-GT
PCNXL4	pecanex-like 4 (Drosophila) [Source:HGNC Symbol;Acc:20349]	chr14	60558629	60635851	667	0.239	2	1	1.911E-03	9.528E-03	PB-GT
MAPRE1	microtubule-associated protein, RP/EB family, member 1 [Source:HGNC Symbol;Acc:6890]	chr20	31407699	31438211	170	0.063	1	1	1.914E-03	9.528E-03	PB-GT
C10orf128	chromosome 10 open reading frame 128 [Source:HGNC Symbol;Acc:27274]	chr10	50362773	50396630	148	0.064	1	1	1.947E-03	9.671E-03	PB-GT
FAM122C	family with sequence similarity 122C [Source:HGNC Symbol;Acc:25202]	chrX	133930819	133988640	383	0.064	1	1	1.951E-03	9.672E-03	PB-GT
ZNF627	zinc finger protein 627 [Source:HGNC Symbol;Acc:30570]	chr19	11670189	11729950	186	0.064	1	1	1.959E-03	9.672E-03	PB-GT
CRISP2	cysteine-rich secretory protein 2 [Source:HGNC Symbol;Acc:12024]	chr6	49660073	49681274	181	0.064	1	1	1.961E-03	9.672E-03	PB-GT
CCT2	chaperonin containing TCP1, subunit 2 (beta) [Source:HGNC Symbol;Acc:1615]	chr12	69979114	69995350	145	0.064	1	1	1.962E-03	9.672E-03	PB-GT
RHOA	ras homolog family member A [Source:HGNC Symbol;Acc:667]	chr3	49396578	49450431	195	0.064	1	1	1.966E-03	9.672E-03	PB-GT
CTCFL	CCCTC-binding factor (zinc finger protein)-like [Source:HGNC Symbol;Acc:16234]	chr20	56071021	56100708	173	0.064	1	1	1.981E-03	9.730E-03	PB-GT
TRMT10A	tRNA methyltransferase 10 homolog A (S. cerevisiae) [Source:HGNC Symbol;Acc:28403]	chr4	100467866	100485189	197	0.065	1	1	1.995E-03	9.775E-03	PB-GT
ZNRF1	zinc and ring finger 1, E3 ubiquitin protein ligase [Source:HGNC Symbol;Acc:18452]	chr16	75032928	75144892	564	0.243	2	1	1.998E-03	9.775E-03	PB-GT
ALDH1A2	aldehyde dehydrogenase 1 family, member A2 [Source:HGNC Symbol;Acc:15472]	chr15	58245622	58790065	3460	1.272	5	2	2.004E-03	9.787E-03	PB-GT
DKK3	dickkopf 3 homolog (Xenopus laevis) [Source:HGNC Symbol;Acc:2893]	chr11	11984653	12031316	178	0.065	1	1	2.010E-03	9.796E-03	PB-GT
TGFA	transforming growth factor, alpha [Source:HGNC Symbol;Acc:11765]	chr2	70674412	70781325	664	0.245	2	1	2.036E-03	9.908E-03	PB-GT
TMCO3	transmembrane and coiled-coil domains 3 [Source:HGNC Symbol;Acc:20329]	chr13	114145310	114204542	272	0.066	1	1	2.064E-03	1.001E-02	PB-GT
KY	kyphoscoliosis peptidase [Source:HGNC Symbol;Acc:26576]	chr3	134321980	134370478	200	0.066	1	1	2.066E-03	1.001E-02	PB-GT
ADAL	adenosine deaminase-like [Source:HGNC Symbol;Acc:31853]	chr15	43622872	43646096	179	0.066	1	1	2.073E-03	1.002E-02	PB-GT
HOXA3	homeobox A3 [Source:HGNC Symbol;Acc:5104]	chr7	27145803	27192200	197	0.066	1	1	2.075E-03	1.002E-02	PB-GT
C6orf99	chromosome 6 open reading frame 99 [Source:HGNC Symbol;Acc:21179]	chr6	159290954	159331385	187	0.066	1	1	2.090E-03	1.008E-02	PB-GT
LSM6	LSM6 homolog, U6 small nuclear RNA associated (S. cerevisiae) [Source:HGNC Symbol;Acc:17017]	chr4	147096837	147121152	202	0.066	1	1	2.095E-03	1.008E-02	PB-GT
PGBD3	piggyBac transposable element derived 3 [Source:HGNC Symbol;Acc:19400]	chr10	50723151	50747086	154	0.066	1	1	2.104E-03	1.011E-02	PB-GT
PTGER3	prostaglandin E receptor 3 (subtype EP3) [Source:HGNC Symbol;Acc:9595]	chr1	71318036	71513491	1486	0.526	3	2	2.109E-03	1.011E-02	PB-GT
BEND7	BEN domain containing 7 [Source:HGNC Symbol;Acc:23514]	chr10	13480484	13570974	578	0.249	2	1	2.135E-03	1.021E-02	PB-GT
TBC1D2	TBC1 domain family, member 2 [Source:HGNC Symbol;Acc:18026]	chr9	100961311	101017915	204	0.067	1	1	2.138E-03	1.021E-02	PB-GT
SEPT9	septin 9 [Source:HGNC Symbol;Acc:7323]	chr17	75276651	75496678	471	0.250	2	2	2.152E-03	1.023E-02	PB-GT
PSMC6	proteasome (prosome, macropain) 26S subunit, ATPase, 6 [Source:HGNC Symbol;Acc:9553]	chr14	53173890	53195305	187	0.067	1	1	2.153E-03	1.023E-02	PB-GT
FHL1	four and a half LIM domains 1 [Source:HGNC Symbol;Acc:3702]	chrX	135229559	135293518	403	0.067	1	1	2.156E-03	1.023E-02	PB-GT
ST8SIA6-AS1	ST8SIA6 antisense RNA 1 [Source:HGNC Symbol;Acc:44880]	chr10	17428935	17455502	156	0.067	1	1	2.158E-03	1.023E-02	PB-GT
CNN3	calponin 3, acidic [Source:HGNC Symbol;Acc:2157]	chr1	95362507	95392834	190	0.067	1	1	2.167E-03	1.025E-02	PB-GT
DLEU7-AS1	DLEU7 antisense RNA 1 [Source:HGNC Symbol;Acc:39966]	chr13	51381992	51423190	280	0.068	1	1	2.185E-03	1.032E-02	PB-GT
HHLA3	HERV-H LTR-associating 3 [Source:HGNC Symbol;Acc:4906]	chr1	70820488	70851022	191	0.068	1	1	2.189E-03	1.032E-02	PB-GT
ABHD2	abhydrolase domain containing 2 [Source:HGNC Symbol;Acc:18717]	chr15	89630690	89745591	685	0.252	2	1	2.206E-03	1.039E-02	PB-GT
MAPKAPK2	mitogen-activated protein kinase-activated protein kinase 2 [Source:HGNC Symbol;Acc:6887]	chr1	206858289	206907628	192	0.068	1	1	2.211E-03	1.039E-02	PB-GT
TIAM2	T-cell lymphoma invasion and metastasis 2 [Source:HGNC Symbol;Acc:11806]	chr6	155153831	155578857	2514	0.889	4	2	2.219E-03	1.041E-02	PB-GT
MKX	mohawk homeobox [Source:HGNC Symbol;Acc:23729]	chr10	27961803	28034989	587	0.253	2	2	2.230E-03	1.044E-02	PB-GT
KLHL42	kelch-like family member 42 [Source:HGNC Symbol;Acc:29252]	chr12	27932953	27955973	155	0.068	1	1	2.236E-03	1.045E-02	PB-GT
ZDHHC6	zinc finger, DHHC-type containing 6 [Source:HGNC Symbol;Acc:19160]	chr10	114190058	114206672	159	0.068	1	1	2.240E-03	1.045E-02	PB-GT
ATP8B4	ATPase, class I, type 8B, member 4 [Source:HGNC Symbol;Acc:13536]	chr15	50150435	50475014	2424	0.891	4	2	2.247E-03	1.046E-02	PB-GT
EIF3F	eukaryotic translation initiation factor 3, subunit F [Source:HGNC Symbol;Acc:3275]	chr11	7991798	8023409	190	0.069	1	1	2.283E-03	1.061E-02	PB-GT
DNAJB11	DnaJ (Hsp40) homolog, subfamily B, member 11 [Source:HGNC Symbol;Acc:14889]	chr3	186285192	186315061	211	0.069	1	1	2.294E-03	1.064E-02	PB-GT
GTF2IRD2	GTF2I repeat domain containing 2 [Source:HGNC Symbol;Acc:30775]	chr7	74210483	74267847	208	0.070	1	1	2.307E-03	1.069E-02	PB-GT
DNAJC27	DnaJ (Hsp40) homolog, subfamily C, member 27 [Source:HGNC Symbol;Acc:30290]	chr2	25166505	25194963	190	0.070	1	1	2.341E-03	1.082E-02	PB-GT
ACTN4	actinin, alpha 4 [Source:HGNC Symbol;Acc:166]	chr19	39138289	39222223	205	0.070	1	1	2.369E-03	1.093E-02	PB-GT
CREG2	cellular repressor of E1A-stimulated genes 2 [Source:HGNC Symbol;Acc:14272]	chr2	101965209	102004057	192	0.071	1	1	2.390E-03	1.101E-02	PB-GT
TXNRD3NB	thioredoxin reductase 3 neighbor [Source:HGNC Symbol;Acc:33870]	chr3	126290625	126327398	216	0.071	1	1	2.401E-03	1.102E-02	PB-GT
ALPK3	alpha-kinase 3 [Source:HGNC Symbol;Acc:17574]	chr15	85359911	85416713	193	0.071	1	1	2.401E-03	1.102E-02	PB-GT
EREG	epiregulin [Source:HGNC Symbol;Acc:3443]	chr4	75230860	75254468	217	0.071	1	1	2.410E-03	1.104E-02	PB-GT
TCF7L2	transcription factor 7-like 2 (T-cell specific, HMG-box) [Source:HGNC Symbol;Acc:11641]	chr10	114710009	114927437	1271	0.547	3	1	2.424E-03	1.109E-02	PB-GT
RNF212	ring finger protein 212 [Source:HGNC Symbol;Acc:27729]	chr4	1050038	1107350	219	0.072	1	1	2.453E-03	1.120E-02	PB-GT
ARFGEF2	ADP-ribosylation factor guanine nucleotide-exchange factor 2 (brefeldin A-inhibited) [Source:HGNC Symbol;Acc:15853]	chr20	47538427	47653230	704	0.262	2	1	2.458E-03	1.120E-02	PB-GT
SLC16A4	solute carrier family 16, member 4 (monocarboxylic acid transporter 5) [Source:HGNC Symbol;Acc:10925]	chr1	110905470	110933704	203	0.072	1	1	2.466E-03	1.122E-02	PB-GT
C6orf118	chromosome 6 open reading frame 118 [Source:HGNC Symbol;Acc:21233]	chr6	165693153	165723096	204	0.072	1	1	2.478E-03	1.124E-02	PB-GT
ACIN1	apoptotic chromatin condensation inducer 1 [Source:HGNC Symbol;Acc:17066]	chr14	23527773	23564823	201	0.072	1	1	2.479E-03	1.124E-02	PB-GT
SLC9A6	solute carrier family 9, subfamily A (NHE6, cation proton antiporter 6), member 6 [Source:HGNC Symbol;Acc:11079]	chrX	135067598	135129423	434	0.072	1	1	2.492E-03	1.126E-02	PB-GT
HOXC5	homeobox C5 [Source:HGNC Symbol;Acc:5127]	chr12	54379629	54429145	164	0.072	1	1	2.496E-03	1.126E-02	PB-GT
PHACTR4	phosphatase and actin regulator 4 [Source:HGNC Symbol;Acc:25793]	chr1	28696114	28826881	743	0.263	2	2	2.499E-03	1.126E-02	PB-GT
DIAPH3-AS1	DIAPH3 antisense RNA 1 [Source:HGNC Symbol;Acc:39915]	chr13	60586885	60618491	300	0.072	1	1	2.500E-03	1.126E-02	PB-GT
SERINC5	serine incorporator 5 [Source:HGNC Symbol;Acc:18825]	chr5	79407050	79551898	808	0.264	2	2	2.515E-03	1.130E-02	PB-GT
STOM	stomatin [Source:HGNC Symbol;Acc:3383]	chr9	124101355	124132531	223	0.073	1	1	2.544E-03	1.141E-02	PB-GT
TRA2A	transformer 2 alpha homolog (Drosophila) [Source:HGNC Symbol;Acc:16645]	chr7	23544399	23571660	219	0.073	1	1	2.551E-03	1.141E-02	PB-GT
GSKIP	GSK3B interacting protein [Source:HGNC Symbol;Acc:20343]	chr14	96829814	96853625	204	0.073	1	1	2.552E-03	1.141E-02	PB-GT
NEK6	NIMA-related kinase 6 [Source:HGNC Symbol;Acc:7749]	chr9	127019885	127115586	225	0.074	1	1	2.589E-03	1.156E-02	PB-GT
GAB4	GRB2-associated binding protein family, member 4 [Source:HGNC Symbol;Acc:18325]	chr22	17442826	17489112	175	0.074	1	1	2.599E-03	1.158E-02	PB-GT
SLC40A1	solute carrier family 40 (iron-regulated transporter), member 1 [Source:HGNC Symbol;Acc:10909]	chr2	190425305	190448484	201	0.074	1	1	2.613E-03	1.162E-02	PB-GT
GOT2	glutamic-oxaloacetic transaminase 2, mitochondrial (aspartate aminotransferase 2) [Source:HGNC Symbol;Acc:4433]	chr16	58741035	58768261	172	0.074	1	1	2.616E-03	1.162E-02	PB-GT
SGOL1	shugoshin-like 1 (S. pombe) [Source:HGNC Symbol;Acc:25088]	chr3	20202085	20227784	226	0.074	1	1	2.623E-03	1.163E-02	PB-GT
RAB31	RAB31, member RAS oncogene family [Source:HGNC Symbol;Acc:9771]	chr18	9708162	9862548	838	0.268	2	2	2.631E-03	1.165E-02	PB-GT
PI4K2B	phosphatidylinositol 4-kinase type 2 beta [Source:HGNC Symbol;Acc:18215]	chr4	25162263	25280714	821	0.269	2	2	2.654E-03	1.173E-02	PB-GT
LCT	lactase [Source:HGNC Symbol;Acc:6530]	chr2	136545410	136594750	203	0.075	1	1	2.664E-03	1.175E-02	PB-GT
ALPL	alkaline phosphatase, liver/bone/kidney [Source:HGNC Symbol;Acc:438]	chr1	21835858	21904905	212	0.075	1	1	2.683E-03	1.182E-02	PB-GT
TANGO6	transport and golgi organization 6 homolog (Drosophila) [Source:HGNC Symbol;Acc:25749]	chr16	68877507	69119083	1308	0.564	3	1	2.694E-03	1.184E-02	PB-GT
LYSMD2	LysM, putative peptidoglycan-binding, domain containing 2 [Source:HGNC Symbol;Acc:28571]	chr15	52015208	52043782	206	0.076	1	1	2.727E-03	1.194E-02	PB-GT
TNIP1	TNFAIP3 interacting protein 1 [Source:HGNC Symbol;Acc:16903]	chr5	150409506	150473138	232	0.076	1	1	2.728E-03	1.194E-02	PB-GT
SP2	Sp2 transcription factor [Source:HGNC Symbol;Acc:11207]	chr17	45973516	46006323	143	0.076	1	1	2.730E-03	1.194E-02	PB-GT
MYT1	myelin transcription factor 1 [Source:HGNC Symbol;Acc:7622]	chr20	62783144	62873604	204	0.076	1	1	2.734E-03	1.194E-02	PB-GT
DSCR3	Down syndrome critical region gene 3 [Source:HGNC Symbol;Acc:3044]	chr21	38595721	38640262	300	0.076	1	1	2.738E-03	1.194E-02	PB-GT
TBC1D30	TBC1 domain family, member 30 [Source:HGNC Symbol;Acc:29164]	chr12	65174589	65274812	617	0.272	2	2	2.747E-03	1.196E-02	PB-GT
CLIP4	CAP-GLY domain containing linker protein family, member 4 [Source:HGNC Symbol;Acc:26108]	chr2	29320571	29412509	740	0.273	2	1	2.761E-03	1.200E-02	PB-GT
INSIG2	insulin induced gene 2 [Source:HGNC Symbol;Acc:20452]	chr2	118846028	118868573	207	0.076	1	1	2.767E-03	1.201E-02	PB-GT
PLB1	phospholipase B1 [Source:HGNC Symbol;Acc:30041]	chr2	28680012	28866654	742	0.274	2	2	2.782E-03	1.205E-02	PB-GT
PHF10	PHD finger protein 10 [Source:HGNC Symbol;Acc:18250]	chr6	170104001	170124151	217	0.077	1	1	2.795E-03	1.207E-02	PB-GT
SLC24A6	solute carrier family 24 (sodium/lithium/calcium exchanger), member 6 [Source:HGNC Symbol;Acc:26175]	chr12	113736564	113797298	174	0.077	1	1	2.802E-03	1.207E-02	PB-GT
CTDP1	CTD (carboxy-terminal domain, RNA polymerase II, polypeptide A) phosphatase, subunit 1 [Source:HGNC Symbol;Acc:2498]	chr18	77439801	77514510	240	0.077	1	1	2.802E-03	1.207E-02	PB-GT
FREM3	FRAS1 related extracellular matrix 3 [Source:HGNC Symbol;Acc:25172]	chr4	144498455	144621828	839	0.275	2	2	2.820E-03	1.212E-02	PB-GT
PTCHD1	patched domain containing 1 [Source:HGNC Symbol;Acc:26392]	chrX	23352133	23422489	463	0.077	1	1	2.827E-03	1.212E-02	PB-GT
DHRS7	dehydrogenase/reductase (SDR family) member 7 [Source:HGNC Symbol;Acc:21524]	chr14	60610838	60636574	215	0.077	1	1	2.827E-03	1.212E-02	PB-GT
MINOS1	mitochondrial inner membrane organizing system 1 [Source:HGNC Symbol;Acc:32068]	chr1	19923477	19956314	218	0.077	1	1	2.833E-03	1.212E-02	PB-GT
OSTM1-AS1	OSTM1 antisense RNA 1 [Source:HGNC Symbol;Acc:43666]	chr6	108444719	108480596	219	0.077	1	1	2.845E-03	1.212E-02	PB-GT
TMEM194B	transmembrane protein 194B [Source:HGNC Symbol;Acc:33700]	chr2	191369068	191399448	210	0.077	1	1	2.846E-03	1.212E-02	PB-GT
KIF3C	kinesin family member 3C [Source:HGNC Symbol;Acc:6321]	chr2	26149471	26205618	210	0.077	1	1	2.846E-03	1.212E-02	PB-GT
LGALS8	lectin, galactoside-binding, soluble, 8 [Source:HGNC Symbol;Acc:6569]	chr1	236681300	236716281	219	0.078	1	1	2.859E-03	1.216E-02	PB-GT
ACACB	acetyl-CoA carboxylase beta [Source:HGNC Symbol;Acc:85]	chr12	109554400	109706031	627	0.277	2	1	2.874E-03	1.220E-02	PB-GT
VEZF1	vascular endothelial zinc finger 1 [Source:HGNC Symbol;Acc:12949]	chr17	56048910	56065620	147	0.078	1	1	2.881E-03	1.221E-02	PB-GT
IL13RA1	interleukin 13 receptor, alpha 1 [Source:HGNC Symbol;Acc:5974]	chrX	117861535	117928502	469	0.078	1	1	2.899E-03	1.227E-02	PB-GT
ALDH1A3	aldehyde dehydrogenase 1 family, member A3 [Source:HGNC Symbol;Acc:409]	chr15	101402129	101456831	213	0.078	1	1	2.911E-03	1.230E-02	PB-GT
SRCIN1	SRC kinase signaling inhibitor 1 [Source:HGNC Symbol;Acc:29506]	chr17	36686251	36762183	148	0.078	1	1	2.919E-03	1.232E-02	PB-GT
CD8B	CD8b molecule [Source:HGNC Symbol;Acc:1707]	chr2	87042462	87089047	213	0.079	1	1	2.926E-03	1.232E-02	PB-GT
ERCC6	excision repair cross-complementing rodent repair deficiency, complementation group 6 [Source:HGNC Symbol;Acc:3438]	chr10	50663414	50747584	647	0.279	2	1	2.930E-03	1.232E-02	PB-GT
JAK1	Janus kinase 1 [Source:HGNC Symbol;Acc:6190]	chr1	65298912	65432187	788	0.279	2	2	2.946E-03	1.237E-02	PB-GT
LRRC37A16P	leucine rich repeat containing 37, member A16, pseudogene [Source:HGNC Symbol;Acc:43820]	chr17	66121918	66148609	149	0.079	1	1	2.958E-03	1.238E-02	PB-GT
DAO	D-amino-acid oxidase [Source:HGNC Symbol;Acc:2671]	chr12	109252708	109294819	179	0.079	1	1	2.961E-03	1.238E-02	PB-GT
ACAA2	acetyl-CoA acyltransferase 2 [Source:HGNC Symbol;Acc:83]	chr18	47309869	47340330	247	0.079	1	1	2.963E-03	1.238E-02	PB-GT
UBE2J1	ubiquitin-conjugating enzyme E2, J1 [Source:HGNC Symbol;Acc:17598]	chr6	90036344	90062567	224	0.079	1	1	2.973E-03	1.240E-02	PB-GT
NUDT5	nudix (nucleoside diphosphate linked moiety X)-type motif 5 [Source:HGNC Symbol;Acc:8052]	chr10	12207324	12238143	184	0.079	1	1	2.978E-03	1.240E-02	PB-GT
HOOK2	hook homolog 2 (Drosophila) [Source:HGNC Symbol;Acc:19885]	chr19	12873817	12983554	231	0.079	1	1	2.990E-03	1.243E-02	PB-GT
PPP1R42	protein phosphatase 1, regulatory subunit 42 [Source:HGNC Symbol;Acc:33732]	chr8	67876334	67968839	718	0.281	2	1	2.994E-03	1.243E-02	PB-GT
RAB10	RAB10, member RAS oncogene family [Source:HGNC Symbol;Acc:9759]	chr2	26256976	26360323	765	0.282	2	1	3.030E-03	1.255E-02	PB-GT
CPO	carboxypeptidase O [Source:HGNC Symbol;Acc:21011]	chr2	207804278	207834198	217	0.080	1	1	3.034E-03	1.255E-02	PB-GT
LRRK1	leucine-rich repeat kinase 1 [Source:HGNC Symbol;Acc:18608]	chr15	101459420	101610317	768	0.282	2	1	3.040E-03	1.256E-02	PB-GT
THAP2	THAP domain containing, apoptosis associated protein 2 [Source:HGNC Symbol;Acc:20854]	chr12	72056789	72074419	182	0.080	1	1	3.058E-03	1.262E-02	PB-GT
DNAJC2	DnaJ (Hsp40) homolog, subfamily C, member 2 [Source:HGNC Symbol;Acc:13192]	chr7	102952921	102985320	241	0.081	1	1	3.075E-03	1.266E-02	PB-GT
AKR1D1	aldo-keto reductase family 1, member D1 [Source:HGNC Symbol;Acc:388]	chr7	137687070	137802732	849	0.284	2	1	3.082E-03	1.267E-02	PB-GT
ZC3H3	zinc finger CCCH-type containing 3 [Source:HGNC Symbol;Acc:28972]	chr8	144519825	144623623	207	0.081	1	1	3.106E-03	1.274E-02	PB-GT
BCL2L1	BCL2-like 1 [Source:HGNC Symbol;Acc:992]	chr20	30252255	30311792	218	0.081	1	1	3.111E-03	1.274E-02	PB-GT
CDC14B	cell division cycle 14B [Source:HGNC Symbol;Acc:1719]	chr9	99252523	99382112	869	0.285	2	1	3.114E-03	1.274E-02	PB-GT

table S1. Genes with significant transposon integrations in DLD-1 *KRAS*$^{wt/-}$ cells after selection in low glucose medium.

Gene ID	Description	Chrom	Start	End	# TTAA sites	Expected	Observed	Libraries	*P*	FDR	Transposon
ANKRD10	ankyrin repeat domain 10 [Source:HGNC Symbol;Acc:20265]	chr13	111530887	111567416	336	0.081	1	1	3.118E-03	1.274E-02	PB-GT
TRMT44	tRNA methyltransferase 44 homolog (S. cerevisiae) [Source:HGNC Symbol;Acc:26653]	chr4	8437867	8495258	248	0.081	1	1	3.127E-03	1.276E-02	PB-GT
TNFAIP8	tumor necrosis factor, alpha-induced protein 8 [Source:HGNC Symbol;Acc:17260]	chr5	118604387	118730294	874	0.285	2	2	3.132E-03	1.276E-02	PB-GT
TNIK	TRAF2 and NCK interacting kinase [Source:HGNC Symbol;Acc:30765]	chr3	170779128	171178197	2938	0.965	4	2	3.151E-03	1.282E-02	PB-GT
GOLT1B	golgi transport 1B [Source:HGNC Symbol;Acc:20175]	chr12	21654715	21671342	185	0.082	1	1	3.157E-03	1.282E-02	PB-GT
MECP2	methyl CpG binding protein 2 (Rett syndrome) [Source:HGNC Symbol;Acc:6990]	chrX	153287024	153402578	491	0.082	1	1	3.169E-03	1.283E-02	PB-GT
NASP	nuclear autoantigenic sperm protein (histone-binding) [Source:HGNC Symbol;Acc:7644]	chr1	46049518	46084566	231	0.082	1	1	3.172E-03	1.283E-02	PB-GT
SPCS2	signal peptidase complex subunit 2 homolog (S. cerevisiae) [Source:HGNC Symbol;Acc:28962]	chr11	74660292	74690076	225	0.082	1	1	3.175E-03	1.283E-02	PB-GT
ENDOD1	endonuclease domain containing 1 [Source:HGNC Symbol;Acc:29129]	chr11	94822974	94865809	226	0.082	1	1	3.202E-03	1.292E-02	PB-GT
FTSJD2	FtsJ methyltransferase domain containing 2 [Source:HGNC Symbol;Acc:21077]	chr6	37400995	37450603	233	0.082	1	1	3.210E-03	1.294E-02	PB-GT
ARHGEF18	Rho/Rac guanine nucleotide exchange factor (GEF) 18 [Source:HGNC Symbol;Acc:17090]	chr19	7459999	7537363	240	0.082	1	1	3.221E-03	1.294E-02	PB-GT
TMA16	translation machinery associated 16 homolog (S. cerevisiae) [Source:HGNC Symbol;Acc:25638]	chr4	164415594	164441691	252	0.083	1	1	3.225E-03	1.294E-02	PB-GT
PDLIM3	PDZ and LIM domain 3 [Source:HGNC Symbol;Acc:20767]	chr4	186422851	186456766	252	0.083	1	1	3.225E-03	1.294E-02	PB-GT
ATP9A	ATPase, class II, type 9A [Source:HGNC Symbol;Acc:13540]	chr20	50213053	50385173	778	0.289	2	1	3.250E-03	1.300E-02	PB-GT
CAV1	caveolin 1, caveolae protein, 22kDa [Source:HGNC Symbol;Acc:1527]	chr7	116164839	116201233	248	0.083	1	1	3.251E-03	1.300E-02	PB-GT
COPZ1	coatomer protein complex, subunit zeta 1 [Source:HGNC Symbol;Acc:2243]	chr12	54694986	54745633	188	0.083	1	1	3.257E-03	1.300E-02	PB-GT
CPN1	carboxypeptidase N, polypeptide 1 [Source:HGNC Symbol;Acc:2312]	chr10	101801950	101841634	193	0.083	1	1	3.268E-03	1.303E-02	PB-GT
CISD1	CDGSH iron sulfur domain 1 [Source:HGNC Symbol;Acc:30880]	chr10	60028818	60049346	194	0.084	1	1	3.301E-03	1.312E-02	PB-GT
LRRC27	leucine rich repeat containing 27 [Source:HGNC Symbol;Acc:29346]	chr10	134145614	134195010	194	0.084	1	1	3.301E-03	1.312E-02	PB-GT
CR2	complement component (3d/Epstein Barr virus) receptor 2 [Source:HGNC Symbol;Acc:2336]	chr1	207627575	207663240	236	0.084	1	1	3.307E-03	1.312E-02	PB-GT
ROR1	receptor tyrosine kinase-like orphan receptor 1 [Source:HGNC Symbol;Acc:10256]	chr1	64239693	64647181	2759	0.977	4	2	3.326E-03	1.318E-02	PB-GT
NFKBIZ	nuclear factor of kappa light polypeptide gene enhancer in B-cells inhibitor, zeta [Source:HGNC Symbol;Acc:29805]	chr3	101546835	101579866	256	0.084	1	1	3.343E-03	1.321E-02	PB-GT
EMC3	ER membrane protein complex subunit 3 [Source:HGNC Symbol;Acc:23999]	chr3	10004221	10052800	256	0.084	1	1	3.343E-03	1.321E-02	PB-GT
HSP90AA6P	heat shock protein 90kDa alpha (cytosolic), class A member 6, pseudogene [Source:HGNC Symbol;Acc:32536]	chr4	171502621	171526601	257	0.084	1	1	3.351E-03	1.322E-02	PB-GT
ZBTB20-AS1	ZBTB20 antisense RNA 1 [Source:HGNC Symbol;Acc:40640]	chr3	114070658	114107825	257	0.084	1	1	3.369E-03	1.325E-02	PB-GT
NR1D2	nuclear receptor subfamily 1, group D, member 2 [Source:HGNC Symbol;Acc:7963]	chr3	23986751	24022109	257	0.084	1	1	3.369E-03	1.325E-02	PB-GT
CLIP2	CAP-GLY domain containing linker protein 2 [Source:HGNC Symbol;Acc:2586]	chr7	73703805	73820273	253	0.085	1	1	3.379E-03	1.325E-02	PB-GT
PVRL3	poliovirus receptor-related 3 [Source:HGNC Symbol;Acc:17664]	chr3	110788918	110994410	1830	0.601	3	2	3.380E-03	1.325E-02	PB-GT
CTBP2	C-terminal binding protein 2 [Source:HGNC Symbol;Acc:2495]	chr10	126676421	126849739	682	0.294	2	1	3.393E-03	1.328E-02	PB-GT
CD96	CD96 molecule [Source:HGNC Symbol;Acc:16892]	chr3	111260926	111384597	895	0.294	2	2	3.402E-03	1.330E-02	PB-GT
RHD	Rh blood group, D antigen [Source:HGNC Symbol;Acc:10009]	chr1	25598884	25656936	240	0.085	1	1	3.416E-03	1.333E-02	PB-GT
ARPC2	actin related protein 2/3 complex, subunit 2, 34kDa [Source:HGNC Symbol;Acc:705]	chr2	219081817	219119079	231	0.085	1	1	3.426E-03	1.335E-02	PB-GT
HSP90AA1	heat shock protein 90kDa alpha (cytosolic), class A member 1 [Source:HGNC Symbol;Acc:5253]	chr14	102547075	102606036	238	0.085	1	1	3.445E-03	1.340E-02	PB-GT
PKD2L1	polycystic kidney disease 2-like 1 [Source:HGNC Symbol;Acc:9011]	chr10	102047903	102090243	199	0.086	1	1	3.469E-03	1.345E-02	PB-GT
ADRA1A	adrenoceptor alpha 1A [Source:HGNC Symbol;Acc:277]	chr8	26605667	26724790	757	0.296	2	1	3.470E-03	1.345E-02	PB-GT
ELMSAN1	ELM2 and Myb/SANT-like domain containing 1 [Source:HGNC Symbol;Acc:19853]	chr14	74181825	74256988	239	0.086	1	1	3.473E-03	1.345E-02	PB-GT
NLRP11	NLR family, pyrin domain containing 11 [Source:HGNC Symbol;Acc:22945]	chr19	56296770	56348166	250	0.086	1	1	3.487E-03	1.349E-02	PB-GT
ERP27	endoplasmic reticulum protein 27 [Source:HGNC Symbol;Acc:26495]	chr12	15066969	15092016	195	0.086	1	1	3.497E-03	1.351E-02	PB-GT
ACOX3	acyl-CoA oxidase 3, pristanoyl [Source:HGNC Symbol;Acc:121]	chr4	8368009	8442450	263	0.086	1	1	3.505E-03	1.351E-02	PB-GT
RASA3	RAS p21 protein activator 3 [Source:HGNC Symbol;Acc:20331]	chr13	114747194	114898086	357	0.086	1	1	3.508E-03	1.351E-02	PB-GT
TMEM33	transmembrane protein 33 [Source:HGNC Symbol;Acc:25541]	chr4	41937137	41962589	264	0.086	1	1	3.531E-03	1.356E-02	PB-GT
TCTN1	tectonic family member 1 [Source:HGNC Symbol;Acc:26113]	chr12	111051832	111087235	196	0.086	1	1	3.532E-03	1.356E-02	PB-GT
MKKS	McKusick-Kaufman syndrome [Source:HGNC Symbol;Acc:7108]	chr20	10385832	10414870	233	0.087	1	1	3.541E-03	1.356E-02	PB-GT
ANGPTL5	angiopoietin-like 5 [Source:HGNC Symbol;Acc:19705]	chr11	101761405	101787253	238	0.087	1	1	3.541E-03	1.356E-02	PB-GT
TM6SF1	transmembrane 6 superfamily member 1 [Source:HGNC Symbol;Acc:11860]	chr15	83776159	83813606	237	0.087	1	1	3.582E-03	1.368E-02	PB-GT
SLC25A33	solute carrier family 25 (pyrimidine nucleotide carrier), member 33 [Source:HGNC Symbol;Acc:29681]	chr1	9599541	9642831	246	0.087	1	1	3.584E-03	1.368E-02	PB-GT
MUC13	mucin 13, cell surface associated [Source:HGNC Symbol;Acc:7511]	chr3	124624289	124672663	266	0.087	1	1	3.602E-03	1.373E-02	PB-GT
B4GALT1	UDP-Gal:betaGlcNAc beta 1,4- galactosyltransferase, polypeptide 1 [Source:HGNC Symbol;Acc:924]	chr9	33104080	33167354	268	0.088	1	1	3.638E-03	1.383E-02	PB-GT
FAN1	FANCD2/FANCI-associated nuclease 1 [Source:HGNC Symbol;Acc:29170]	chr15	31196055	31235311	239	0.088	1	1	3.641E-03	1.383E-02	PB-GT
TMCC1	transmembrane and coiled-coil domain family 1 [Source:HGNC Symbol;Acc:29116]	chr3	129366635	129612419	1870	0.614	3	2	3.647E-03	1.383E-02	PB-GT
PREP	prolyl endopeptidase [Source:HGNC Symbol;Acc:9358]	chr6	105725440	105850959	853	0.301	2	2	3.649E-03	1.383E-02	PB-GT
SLC6A6	solute carrier family 6 (neurotransmitter transporter, taurine), member 6 [Source:HGNC Symbol;Acc:11052]	chr3	14444076	14530857	268	0.088	1	1	3.654E-03	1.383E-02	PB-GT
LNPEP	leucyl/cystinyl aminopeptidase [Source:HGNC Symbol;Acc:6656]	chr5	96271098	96373219	925	0.302	2	1	3.668E-03	1.386E-02	PB-GT
IGSF5	immunoglobulin superfamily, member 5 [Source:HGNC Symbol;Acc:5952]	chr21	41117334	41174023	352	0.089	1	1	3.736E-03	1.408E-02	PB-GT
RIPK1	receptor (TNFRSF)-interacting serine-threonine kinase 1 [Source:HGNC Symbol;Acc:10019]	chr6	3064225	3115421	252	0.089	1	1	3.739E-03	1.408E-02	PB-GT
AP3M1	adaptor-related protein complex 3, mu 1 subunit [Source:HGNC Symbol;Acc:569]	chr10	75881524	75910821	207	0.089	1	1	3.745E-03	1.409E-02	PB-GT
WDR76	WD repeat domain 76 [Source:HGNC Symbol;Acc:25773]	chr15	44119161	44160617	243	0.089	1	1	3.761E-03	1.413E-02	PB-GT
NIPAL2	NIPA-like domain containing 2 [Source:HGNC Symbol;Acc:25854]	chr8	99202061	99306621	781	0.305	2	1	3.784E-03	1.418E-02	PB-GT
KLHDC4	kelch domain containing 4 [Source:HGNC Symbol;Acc:25272]	chr16	87730091	87799598	208	0.090	1	1	3.787E-03	1.418E-02	PB-GT
AMZ1	archaelysin family metallopeptidase 1 [Source:HGNC Symbol;Acc:22231]	chr7	2719156	2804759	269	0.090	1	1	3.807E-03	1.424E-02	PB-GT
CELF4	CUGBP, Elav-like family member 4 [Source:HGNC Symbol;Acc:14015]	chr18	34823010	35146000	959	0.307	2	1	3.832E-03	1.431E-02	PB-GT
DDX19B	DEAD (Asp-Glu-Ala-Asp) box polypeptide 19B [Source:HGNC Symbol;Acc:2742]	chr16	70323566	70369186	210	0.091	1	1	3.858E-03	1.439E-02	PB-GT
ZNF430	zinc finger protein 430 [Source:HGNC Symbol;Acc:20808]	chr19	21203426	21242856	264	0.091	1	1	3.876E-03	1.443E-02	PB-GT
ZNF727	zinc finger protein 727 [Source:HGNC Symbol;Acc:22785]	chr7	63505821	63538927	272	0.091	1	1	3.890E-03	1.443E-02	PB-GT
JPH3	junctophilin 3 [Source:HGNC Symbol;Acc:14203]	chr16	87635441	87731762	211	0.091	1	1	3.894E-03	1.443E-02	PB-GT
ACSM2A	acyl-CoA synthetase medium-chain family member 2A [Source:HGNC Symbol;Acc:32017]	chr16	20462783	20498991	211	0.091	1	1	3.894E-03	1.443E-02	PB-GT
PATL1	protein associated with topoisomerase II homolog 1 (yeast) [Source:HGNC Symbol;Acc:26721]	chr11	59404189	59436453	250	0.091	1	1	3.896E-03	1.443E-02	PB-GT
EMILIN2	elastin microfibril interfacer 2 [Source:HGNC Symbol;Acc:19881]	chr18	2847028	2915991	285	0.091	1	1	3.913E-03	1.447E-02	PB-GT
NXN	nucleoredoxin [Source:HGNC Symbol;Acc:18008]	chr17	702553	883010	584	0.309	2	2	3.924E-03	1.449E-02	PB-GT
LINC00348	long intergenic non-protein coding RNA 348 [Source:HGNC Symbol;Acc:42658]	chr13	71589273	71742549	1285	0.310	2	1	3.951E-03	1.457E-02	PB-GT
CEP55	centrosomal protein 55kDa [Source:HGNC Symbol;Acc:1161]	chr10	95256389	95288849	213	0.092	1	1	3.958E-03	1.457E-02	PB-GT
ZBTB8OS	zinc finger and BTB domain containing 8 opposite strand [Source:HGNC Symbol;Acc:24094]	chr1	33065773	33116504	261	0.092	1	1	4.021E-03	1.478E-02	PB-GT
ATP6V0E1	ATPase, H+ transporting, lysosomal 9kDa, V0 subunit e1 [Source:HGNC Symbol;Acc:863]	chr5	172410760	172462448	284	0.093	1	1	4.043E-03	1.484E-02	PB-GT
CDC27	cell division cycle 27 [Source:HGNC Symbol;Acc:1728]	chr17	45195069	45266788	591	0.313	2	2	4.056E-03	1.485E-02	PB-GT
PPA1	pyrophosphatase (inorganic) 1 [Source:HGNC Symbol;Acc:9226]	chr10	71962587	71993667	216	0.093	1	1	4.067E-03	1.485E-02	PB-GT
FAM192A	family with sequence similarity 192, member A [Source:HGNC Symbol;Acc:29856]	chr16	57186378	57220028	216	0.093	1	1	4.075E-03	1.485E-02	PB-GT
LAMTOR5-AS1	LAMTOR5 antisense RNA 1 [Source:HGNC Symbol;Acc:40823]	chr1	110925441	110974982	263	0.093	1	1	4.081E-03	1.485E-02	PB-GT
PMPCB	peptidase (mitochondrial processing) beta [Source:HGNC Symbol;Acc:9119]	chr7	102937869	102969958	279	0.093	1	1	4.086E-03	1.485E-02	PB-GT
ATP6V1E2	ATPase, H+ transporting, lysosomal 31kDa, V1 subunit E2 [Source:HGNC Symbol;Acc:18125]	chr2	46717889	46769696	253	0.093	1	1	4.088E-03	1.485E-02	PB-GT
LSMD1	LSM domain containing 1 [Source:HGNC Symbol;Acc:28212]	chr17	7760003	7788556	176	0.093	1	1	4.088E-03	1.485E-02	PB-GT
TIPARP	TCDD-inducible poly(ADP-ribose) polymerase [Source:HGNC Symbol;Acc:23696]	chr3	156391024	156424559	284	0.093	1	1	4.090E-03	1.485E-02	PB-GT
PATL2	protein associated with topoisomerase II homolog 2 (yeast) [Source:HGNC Symbol;Acc:33630]	chr15	44957930	45003514	254	0.093	1	1	4.098E-03	1.486E-02	PB-GT
OLAH	oleoyl-ACP hydrolase [Source:HGNC Symbol;Acc:25625]	chr10	15074226	15115851	218	0.094	1	1	4.140E-03	1.495E-02	PB-GT
UNC5B	unc-5 homolog B (C. elegans) [Source:HGNC Symbol;Acc:12568]	chr10	72972327	73062621	218	0.094	1	1	4.140E-03	1.495E-02	PB-GT
S100PBP	S100P binding protein [Source:HGNC Symbol;Acc:25768]	chr1	33282368	33324476	265	0.094	1	1	4.141E-03	1.495E-02	PB-GT
ESM1	endothelial cell-specific molecule 1 [Source:HGNC Symbol;Acc:3466]	chr5	54273692	54318499	288	0.094	1	1	4.154E-03	1.498E-02	PB-GT
TOP3A	topoisomerase (DNA) III alpha [Source:HGNC Symbol;Acc:11992]	chr17	18174742	18218321	178	0.094	1	1	4.179E-03	1.503E-02	PB-GT
HPSE	heparanase [Source:HGNC Symbol;Acc:5164]	chr4	84213614	84256306	288	0.094	1	1	4.180E-03	1.503E-02	PB-GT
TSPAN11	tetraspanin 11 [Source:HGNC Symbol;Acc:30795]	chr12	31079362	31149534	215	0.095	1	1	4.227E-03	1.516E-02	PB-GT
C4orf45	chromosome 4 open reading frame 45 [Source:HGNC Symbol;Acc:26342]	chr4	159814286	159959912	971	0.318	2	2	4.233E-03	1.516E-02	PB-GT
SLC30A5	solute carrier family 30 (zinc transporter), member 5 [Source:HGNC Symbol;Acc:19089]	chr5	68389473	68426896	291	0.095	1	1	4.238E-03	1.516E-02	PB-GT
FDFT1	farnesyl-diphosphate farnesyltransferase 1 [Source:HGNC Symbol;Acc:3629]	chr8	11653082	11696818	243	0.095	1	1	4.240E-03	1.516E-02	PB-GT
NPAS2	neuronal PAS domain protein 2 [Source:HGNC Symbol;Acc:7895]	chr2	101436614	101613291	864	0.318	2	1	4.249E-03	1.516E-02	PB-GT
IFT74	intraflagellar transport 74 homolog (Chlamydomonas) [Source:HGNC Symbol;Acc:21424]	chr9	26947037	27062928	972	0.319	2	1	4.251E-03	1.516E-02	PB-GT
POR	P450 (cytochrome) oxidoreductase [Source:HGNC Symbol;Acc:9208]	chr7	75528518	75616173	285	0.095	1	1	4.258E-03	1.516E-02	PB-GT
MLXIP	MLX interacting protein [Source:HGNC Symbol;Acc:17055]	chr12	122516628	122629266	216	0.095	1	1	4.265E-03	1.517E-02	PB-GT
ASH2L	ash2 (absent, small, or homeotic)-like (Drosophila) [Source:HGNC Symbol;Acc:744]	chr8	37962760	38001594	244	0.095	1	1	4.274E-03	1.518E-02	PB-GT
RBM17	RNA binding motif protein 17 [Source:HGNC Symbol;Acc:16944]	chr10	6130950	6159420	222	0.096	1	1	4.289E-03	1.519E-02	PB-GT
GTF2F2	general transcription factor IIF, polypeptide 2, 30kDa [Source:HGNC Symbol;Acc:4653]	chr13	45694650	45858237	1324	0.320	2	2	4.292E-03	1.519E-02	PB-GT
C1orf105	chromosome 1 open reading frame 105 [Source:HGNC Symbol;Acc:29591]	chr1	172389828	172437971	270	0.096	1	1	4.294E-03	1.519E-02	PB-GT
TCEB1	transcription elongation factor B (SIII), polypeptide 1 (15kDa, elongin C) [Source:HGNC Symbol;Acc:11617]	chr8	74851404	74884522	246	0.096	1	1	4.342E-03	1.534E-02	PB-GT
LINC00466	long intergenic non-protein coding RNA 466 [Source:HGNC Symbol;Acc:27294]	chr1	63655660	63782902	907	0.321	2	1	4.354E-03	1.536E-02	PB-GT
GAD1	glutamate decarboxylase 1 (brain, 67kDa) [Source:HGNC Symbol;Acc:4092]	chr2	171669723	171717661	263	0.097	1	1	4.407E-03	1.552E-02	PB-GT
TNRC6A	trinucleotide repeat containing 6A [Source:HGNC Symbol;Acc:11969]	chr16	24741016	24838953	751	0.324	2	2	4.444E-03	1.562E-02	PB-GT
TASP1	taspase, threonine aspartase, 1 [Source:HGNC Symbol;Acc:15859]	chr20	13246709	13619587	2819	1.048	4	2	4.447E-03	1.562E-02	PB-GT
SCAF11	SR-related CTD-associated factor 11 [Source:HGNC Symbol;Acc:10784]	chr12	46312914	46385903	735	0.324	2	1	4.469E-03	1.568E-02	PB-GT
DMBT1	deleted in malignant brain tumors 1 [Source:HGNC Symbol;Acc:2926]	chr10	124320181	124403252	227	0.098	1	1	4.478E-03	1.569E-02	PB-GT
RANBP10	RAN binding protein 10 [Source:HGNC Symbol;Acc:29285]	chr16	67757005	67840555	227	0.098	1	1	4.486E-03	1.570E-02	PB-GT
KIF23	kinesin family member 23 [Source:HGNC Symbol;Acc:6392]	chr15	69706585	69740764	267	0.098	1	1	4.514E-03	1.576E-02	PB-GT
SAAL1	serum amyloid A-like 1 [Source:HGNC Symbol;Acc:25158]	chr11	18091482	18127638	270	0.098	1	1	4.523E-03	1.576E-02	PB-GT
NUFIP1	nuclear fragile X mental retardation protein interacting protein 1 [Source:HGNC Symbol;Acc:8057]	chr13	45513384	45563618	407	0.098	1	1	4.523E-03	1.576E-02	PB-GT
CALCOCO2	calcium binding and coiled-coil domain 2 [Source:HGNC Symbol;Acc:29912]	chr17	46908350	46943884	186	0.099	1	1	4.550E-03	1.583E-02	PB-GT
TRAP1	TNF receptor-associated protein 1 [Source:HGNC Symbol;Acc:16264]	chr16	3701640	3767598	229	0.099	1	1	4.563E-03	1.586E-02	PB-GT
MAPRE3	microtubule-associated protein, RP/EB family, member 3 [Source:HGNC Symbol;Acc:6892]	chr2	27193480	27250064	268	0.099	1	1	4.570E-03	1.586E-02	PB-GT
AGR2	anterior gradient 2 homolog (Xenopus laevis) [Source:HGNC Symbol;Acc:328]	chr7	16831435	16873057	296	0.099	1	1	4.582E-03	1.588E-02	PB-GT
PHF2	PHD finger protein 2 [Source:HGNC Symbol;Acc:8920]	chr9	96338689	96441869	302	0.099	1	1	4.586E-03	1.588E-02	PB-GT
EXTL3	exostosin-like glycosyltransferase 3 [Source:HGNC Symbol;Acc:3518]	chr8	28457986	28613116	838	0.328	2	1	4.598E-03	1.590E-02	PB-GT
SRRM1	serine/arginine repetitive matrix 1 [Source:HGNC Symbol;Acc:16638]	chr1	24958207	24999758	280	0.099	1	1	4.607E-03	1.590E-02	PB-GT
FGD3	FYVE, RhoGEF and PH domain containing 3 [Source:HGNC Symbol;Acc:16027]	chr9	95709733	95798518	304	0.100	1	1	4.645E-03	1.600E-02	PB-GT
GPR19	G protein-coupled receptor 19 [Source:HGNC Symbol;Acc:4473]	chr12	12813825	12849141	226	0.100	1	1	4.655E-03	1.600E-02	PB-GT
ADCY9	adenylate cyclase 9 [Source:HGNC Symbol;Acc:240]	chr16	4003388	4166186	764	0.329	2	2	4.660E-03	1.600E-02	PB-GT
ANKRD62	ankyrin repeat domain 62 [Source:HGNC Symbol;Acc:35241]	chr18	12093848	12129748	312	0.100	1	1	4.663E-03	1.600E-02	PB-GT
FYTTD1	forty-two-three domain containing 1 [Source:HGNC Symbol;Acc:25407]	chr3	197464050	197511317	304	0.100	1	1	4.666E-03	1.600E-02	PB-GT
TNRC18	trinucleotide repeat containing 18 [Source:HGNC Symbol;Acc:11962]	chr7	5346421	5465045	299	0.100	1	1	4.672E-03	1.600E-02	PB-GT
WTAP	Wilms tumor 1 associated protein [Source:HGNC Symbol;Acc:16846]	chr6	160146617	160177351	283	0.100	1	1	4.681E-03	1.601E-02	PB-GT
TMED8	transmembrane emp24 protein transport domain containing 8 [Source:HGNC Symbol;Acc:18633]	chr14	77801364	77843452	279	0.100	1	1	4.688E-03	1.601E-02	PB-GT
SLFN5	schlafen family member 5 [Source:HGNC Symbol;Acc:28286]	chr17	33570055	33600674	189	0.100	1	1	4.693E-03	1.601E-02	PB-GT
DYRK1A	dual-specificity tyrosine-(Y)-phosphorylation regulated kinase 1A [Source:HGNC Symbol;Acc:3091]	chr21	38739236	38887680	1307	0.331	2	2	4.710E-03	1.603E-02	PB-GT
CHP1	calcineurin-like EF-hand protein 1 [Source:HGNC Symbol;Acc:17433]	chr15	41523037	41574043	273	0.100	1	1	4.712E-03	1.603E-02	PB-GT
ATG3	autophagy related 3 [Source:HGNC Symbol;Acc:20962]	chr3	112251356	112280893	306	0.101	1	1	4.725E-03	1.606E-02	PB-GT
PRR5L	proline rich 5 like [Source:HGNC Symbol;Acc:25878]	chr11	36317838	36486754	910	0.331	2	2	4.732E-03	1.606E-02	PB-GT
C10orf129	chromosome 10 open reading frame 129 [Source:HGNC Symbol;Acc:31665]	chr10	96953957	96988685	234	0.101	1	1	4.749E-03	1.610E-02	PB-GT
ING3	inhibitor of growth family, member 3 [Source:HGNC Symbol;Acc:14587]	chr7	120590803	120617270	302	0.101	1	1	4.763E-03	1.612E-02	PB-GT
TRIM66	tripartite motif containing 66 [Source:HGNC Symbol;Acc:29005]	chr11	8633584	8693413	278	0.101	1	1	4.785E-03	1.618E-02	PB-GT
HACL1	2-hydroxyacyl-CoA lyase 1 [Source:HGNC Symbol;Acc:17856]	chr3	15602211	15643338	310	0.102	1	1	4.845E-03	1.634E-02	PB-GT
CMIP	c-Maf inducing protein [Source:HGNC Symbol;Acc:24319]	chr16	81478775	81745367	775	0.334	2	2	4.847E-03	1.634E-02	PB-GT
CDC42	cell division cycle 42 [Source:HGNC Symbol;Acc:1736]	chr1	22379120	22419437	288	0.102	1	1	4.865E-03	1.636E-02	PB-GT
C1orf186	chromosome 1 open reading frame 186 [Source:HGNC Symbol;Acc:25341]	chr1	206238872	206288647	288	0.102	1	1	4.865E-03	1.636E-02	PB-GT
KITLG	KIT ligand [Source:HGNC Symbol;Acc:6343]	chr12	88886570	88974628	759	0.335	2	2	4.883E-03	1.640E-02	PB-GT
VAV1	vav 1 guanine nucleotide exchange factor [Source:HGNC Symbol;Acc:12657]	chr19	6772725	6857377	298	0.102	1	1	4.901E-03	1.644E-02	PB-GT
TRIM29	tripartite motif containing 29 [Source:HGNC Symbol;Acc:17274]	chr11	119981983	120056237	283	0.103	1	1	4.953E-03	1.658E-02	PB-GT
HTR7	5-hydroxytryptamine (serotonin) receptor 7, adenylate cyclase-coupled [Source:HGNC Symbol;Acc:5302]	chr10	92500578	92617671	782	0.337	2	2	4.955E-03	1.658E-02	PB-GT
RAB5A	RAB5A, member RAS oncogene family [Source:HGNC Symbol;Acc:9783]	chr3	19988571	20026667	314	0.103	1	1	4.967E-03	1.660E-02	PB-GT
IL20RA	interleukin 20 receptor, alpha [Source:HGNC Symbol;Acc:6003]	chr6	137321108	137366298	293	0.104	1	1	5.006E-03	1.671E-02	PB-GT
MYO19	myosin XIX [Source:HGNC Symbol;Acc:26234]	chr17	34851477	34899284	196	0.104	1	1	5.035E-03	1.678E-02	PB-GT
ZFAND1	zinc finger, AN1-type domain 1 [Source:HGNC Symbol;Acc:25858]	chr8	82613569	82645138	266	0.104	1	1	5.051E-03	1.680E-02	PB-GT
TRIM22	tripartite motif containing 22 [Source:HGNC Symbol;Acc:16379]	chr11	5710919	5758319	286	0.104	1	1	5.055E-03	1.680E-02	PB-GT
MMAA	methylmalonic aciduria (cobalamin deficiency) cblA type [Source:HGNC Symbol;Acc:18871]	chr4	146539415	146581187	320	0.105	1	1	5.125E-03	1.702E-02	PB-GT
MARCH3	membrane-associated ring finger (C3HC4) 3, E3 ubiquitin protein ligase [Source:HGNC Symbol;Acc:28728]	chr5	126203406	126366500	1045	0.341	2	1	5.138E-03	1.704E-02	PB-GT
ZNF410	zinc finger protein 410 [Source:HGNC Symbol;Acc:20144]	chr14	74353320	74399214	293	0.105	1	1	5.154E-03	1.707E-02	PB-GT
QRSL1	glutaminyl-tRNA synthase (glutamine-hydrolyzing)-like 1 [Source:HGNC Symbol;Acc:21020]	chr6	107077453	107116292	298	0.105	1	1	5.172E-03	1.711E-02	PB-GT
TRAM1	translocation associated membrane protein 1 [Source:HGNC Symbol;Acc:20568]	chr8	71485677	71520622	270	0.106	1	1	5.198E-03	1.715E-02	PB-GT
PARP14	poly (ADP-ribose) polymerase family, member 14 [Source:HGNC Symbol;Acc:29232]	chr3	122399465	122449687	322	0.106	1	1	5.214E-03	1.715E-02	PB-GT
PRRC1	proline-rich coiled-coil 1 [Source:HGNC Symbol;Acc:28164]	chr5	126853301	126890781	324	0.106	1	1	5.217E-03	1.715E-02	PB-GT
CA5B	carbonic anhydrase VB, mitochondrial [Source:HGNC Symbol;Acc:1378]	chrX	15706953	15805747	635	0.106	1	1	5.217E-03	1.715E-02	PB-GT
ZNF850	zinc finger protein 850 [Source:HGNC Symbol;Acc:27994]	chr19	37205285	37263727	308	0.106	1	1	5.223E-03	1.715E-02	PB-GT
ZNF710	zinc finger protein 710 [Source:HGNC Symbol;Acc:25352]	chr15	90544624	90625438	288	0.106	1	1	5.225E-03	1.715E-02	PB-GT
DRAM1	DNA-damage regulated autophagy modulator 1 [Source:HGNC Symbol;Acc:25645]	chr12	102271129	102405908	780	0.344	2	1	5.263E-03	1.723E-02	PB-GT
MROH1	maestro heat-like repeat family member 1 [Source:HGNC Symbol;Acc:26958]	chr8	145202919	145316843	272	0.106	1	1	5.273E-03	1.723E-02	PB-GT

table S1. Genes with significant transposon integrations in DLD-1 *KRAS*$^{wt/-}$ cells after selection in low glucose medium.

Gene ID	Description	Chrom	Start	End	# TTAA sites	Expected	Observed	Libraries	*P*	FDR	Transposon
SETD5-AS1	SETD5 antisense RNA 1 [Source:HGNC Symbol;Acc:44478]	chr3	9391373	9440263	324	0.106	1	1	5.277E-03	1.723E-02	PB-GT
TSEN2	tRNA splicing endonuclease 2 homolog (S. cerevisiae) [Source:HGNC Symbol;Acc:28422]	chr3	12525931	12581122	324	0.106	1	1	5.277E-03	1.723E-02	PB-GT
ZNF713	zinc finger protein 713 [Source:HGNC Symbol;Acc:22043]	chr7	55955169	56009918	319	0.107	1	1	5.295E-03	1.725E-02	PB-GT
SBF2-AS1	SBF2 antisense RNA 1 [Source:HGNC Symbol;Acc:27438]	chr11	9779839	9832866	293	0.107	1	1	5.297E-03	1.725E-02	PB-GT
HMGN3	high mobility group nucleosomal binding domain 3 [Source:HGNC Symbol;Acc:12312]	chr6	79910962	79944406	302	0.107	1	1	5.307E-03	1.725E-02	PB-GT
NKIRAS1	NFKB inhibitor interacting Ras-like 1 [Source:HGNC Symbol;Acc:17899]	chr3	23933151	23988082	325	0.107	1	1	5.308E-03	1.725E-02	PB-GT
PUM1	pumilio homolog 1 (Drosophila) [Source:HGNC Symbol;Acc:14957]	chr1	31404353	31538838	976	0.346	2	2	5.329E-03	1.729E-02	PB-GT
FAT1	FAT tumor suppressor homolog 1 (Drosophila) [Source:HGNC Symbol;Acc:3595]	chr4	187508937	187647876	1056	0.346	2	2	5.335E-03	1.729E-02	PB-GT
RNF175	ring finger protein 175 [Source:HGNC Symbol;Acc:27735]	chr4	154631277	154681387	328	0.107	1	1	5.375E-03	1.740E-02	PB-GT
SPTAN1	spectrin, alpha, non-erythrocytic 1 [Source:HGNC Symbol;Acc:11273]	chr9	131314866	131395941	329	0.108	1	1	5.411E-03	1.750E-02	PB-GT
RNF214	ring finger protein 214 [Source:HGNC Symbol;Acc:25335]	chr11	117103341	117157161	297	0.108	1	1	5.437E-03	1.756E-02	PB-GT
ERLEC1	endoplasmic reticulum lectin 1 [Source:HGNC Symbol;Acc:25222]	chr2	54014181	54045956	294	0.108	1	1	5.465E-03	1.763E-02	PB-GT
ADNP	activity-dependent neuroprotector homeobox [Source:HGNC Symbol;Acc:15766]	chr20	49505453	49547958	293	0.109	1	1	5.517E-03	1.777E-02	PB-GT
LACTB2	lactamase, beta 2 [Source:HGNC Symbol;Acc:18512]	chr8	71547553	71581409	279	0.109	1	1	5.538E-03	1.782E-02	PB-GT
NHSL1	NHS-like 1 [Source:HGNC Symbol;Acc:21021]	chr6	138743180	139013708	1961	0.693	3	2	5.559E-03	1.786E-02	PB-GT
PRKAA1	protein kinase, AMP-activated, alpha 1 catalytic subunit [Source:HGNC Symbol;Acc:9376]	chr5	40759481	40798476	335	0.109	1	1	5.564E-03	1.786E-02	PB-GT
GBP2	guanylate binding protein 2, interferon-inducible [Source:HGNC Symbol;Acc:4183]	chr1	89571815	89616139	309	0.109	1	1	5.573E-03	1.786E-02	PB-GT
ZUFSP	zinc finger with UFM1-specific peptidase domain [Source:HGNC Symbol;Acc:21224]	chr6	116956781	116989957	310	0.110	1	1	5.581E-03	1.787E-02	PB-GT
ACSBG1	acyl-CoA synthetase bubblegum family member 1 [Source:HGNC Symbol;Acc:29567]	chr15	78459810	78538030	300	0.110	1	1	5.653E-03	1.808E-02	PB-GT
NFATC1	nuclear factor of activated T-cells, cytoplasmic, calcineurin-dependent 1 [Source:HGNC Symbol;Acc:7775]	chr18	77155856	77289325	345	0.110	1	1	5.662E-03	1.808E-02	PB-GT
NEGR1-IT1	NEGR1 intronic transcript 1 (non-protein coding) [Source:HGNC Symbol;Acc:41432]	chr1	72259915	72302695	312	0.111	1	1	5.677E-03	1.811E-02	PB-GT
POM121	POM121 transmembrane nucleoporin [Source:HGNC Symbol;Acc:19702]	chr7	72349936	72421979	331	0.111	1	1	5.686E-03	1.811E-02	PB-GT
HLA-AS1	HLA antisense RNA 1 [Source:HGNC Symbol;Acc:42509]	chr1	221006105	221053482	314	0.111	1	1	5.748E-03	1.829E-02	PB-GT
ST3GAL5	ST3 beta-galactoside alpha-2,3-sialyltransferase 5 [Source:HGNC Symbol;Acc:10872]	chr2	86066267	86116137	302	0.111	1	1	5.755E-03	1.829E-02	PB-GT
ITGB1	integrin, beta 1 (fibronectin receptor, beta polypeptide, antigen CD29 includes MDF2, MSK12) [Source:HGNC Symbol;Acc:6153]	chr10	33189247	33294720	828	0.357	2	1	5.797E-03	1.840E-02	PB-GT
ATP7B	ATPase, Cu++ transporting, beta polypeptide [Source:HGNC Symbol;Acc:870]	chr13	52506809	52585630	465	0.112	1	1	5.850E-03	1.850E-02	PB-GT
CSRNP3	cysteine-serine-rich nuclear protein 3 [Source:HGNC Symbol;Acc:30729]	chr2	166326157	166545917	1909	0.704	3	2	5.859E-03	1.850E-02	PB-GT
WDR52	WD repeat domain 52 [Source:HGNC Symbol;Acc:25631]	chr3	113005777	113160457	1090	0.358	2	1	5.862E-03	1.850E-02	PB-GT
MYO9B	myosin IXB [Source:HGNC Symbol;Acc:7609]	chr19	17186591	17325346	327	0.112	1	1	5.862E-03	1.850E-02	PB-GT
PDSS1	prenyl (decaprenyl) diphosphate synthase, subunit 1 [Source:HGNC Symbol;Acc:17759]	chr10	26986588	27035727	261	0.112	1	1	5.863E-03	1.850E-02	PB-GT
PSD3	pleckstrin and Sec7 domain containing 3 [Source:HGNC Symbol;Acc:19093]	chr8	18384811	18942240	4069	1.591	5	2	5.890E-03	1.856E-02	PB-GT
SORL1	sortilin-related receptor, L(DLR class) A repeats containing [Source:HGNC Symbol;Acc:11185]	chr11	121322912	121504402	987	0.359	2	1	5.914E-03	1.862E-02	PB-GT
REEP5	receptor accessory protein 5 [Source:HGNC Symbol;Acc:30077]	chr5	112212084	112258236	347	0.113	1	1	5.954E-03	1.871E-02	PB-GT
UBE2O	ubiquitin-conjugating enzyme E2O [Source:HGNC Symbol;Acc:29554]	chr17	74385532	74449288	214	0.113	1	1	5.964E-03	1.871E-02	PB-GT
EBAG9	estrogen receptor binding site associated, antigen, 9 [Source:HGNC Symbol;Acc:3123]	chr8	110551940	110578225	290	0.113	1	1	5.966E-03	1.871E-02	PB-GT
GRB10	growth factor receptor-bound protein 10 [Source:HGNC Symbol;Acc:4564]	chr7	50657760	50861159	1079	0.361	2	1	5.978E-03	1.873E-02	PB-GT
CLEC3A	C-type lectin domain family 3, member A [Source:HGNC Symbol;Acc:2052]	chr16	78056412	78100658	264	0.114	1	1	6.004E-03	1.878E-02	PB-GT
ZNF704	zinc finger protein 704 [Source:HGNC Symbol;Acc:32291]	chr8	81540686	81787016	1816	0.710	3	1	6.049E-03	1.889E-02	PB-GT
GPD1L	glycerol-3-phosphate dehydrogenase 1-like [Source:HGNC Symbol;Acc:28956]	chr3	32147181	32210205	348	0.114	1	1	6.056E-03	1.889E-02	PB-GT
TMCO5A	transmembrane and coiled-coil domains 5A [Source:HGNC Symbol;Acc:28558]	chr15	38214140	38259925	311	0.114	1	1	6.059E-03	1.889E-02	PB-GT
LAMC2	laminin, gamma 2 [Source:HGNC Symbol;Acc:6493]	chr1	183155373	183214035	323	0.114	1	1	6.069E-03	1.890E-02	PB-GT
SHROOM2	shroom family member 2 [Source:HGNC Symbol;Acc:630]	chrX	9754496	9917483	688	0.115	1	1	6.089E-03	1.890E-02	PB-GT
LINC00265	long intergenic non-protein coding RNA 265 [Source:HGNC Symbol;Acc:28019]	chr7	39773231	39832691	343	0.115	1	1	6.089E-03	1.890E-02	PB-GT
FAM47E-STBD1	FAM47E-STBD1 readthrough [Source:HGNC Symbol;Acc:44667]	chr4	77172886	77232752	350	0.115	1	1	6.091E-03	1.890E-02	PB-GT
RNF170	ring finger protein 170 [Source:HGNC Symbol;Acc:25358]	chr8	42704780	42752433	294	0.115	1	1	6.125E-03	1.898E-02	PB-GT
SLC30A4	solute carrier family 30 (zinc transporter), member 4 [Source:HGNC Symbol;Acc:11015]	chr15	45771809	45815005	313	0.115	1	1	6.134E-03	1.899E-02	PB-GT
ST8SIA2	ST8 alpha-N-acetyl-neuraminide alpha-2,8-sialyltransferase 2 [Source:HGNC Symbol;Acc:10870]	chr15	92937058	93011958	316	0.116	1	1	6.248E-03	1.931E-02	PB-GT
ANKRD30B	ankyrin repeat domain 30B [Source:HGNC Symbol;Acc:24165]	chr18	14728271	14852737	1146	0.367	2	1	6.257E-03	1.932E-02	PB-GT
C2CD3	C2 calcium-dependent domain containing 3 [Source:HGNC Symbol;Acc:24564]	chr11	73723763	73882255	1008	0.367	2	2	6.265E-03	1.932E-02	PB-GT
PDE6D	phosphodiesterase 6D, cGMP-specific, rod, delta [Source:HGNC Symbol;Acc:8788]	chr2	232597135	232650982	317	0.117	1	1	6.318E-03	1.945E-02	PB-GT
KPNA6	karyopherin alpha 6 (importin alpha 7) [Source:HGNC Symbol;Acc:6399]	chr1	32573639	32642169	330	0.117	1	1	6.325E-03	1.945E-02	PB-GT
MUC7	mucin 7, secreted [Source:HGNC Symbol;Acc:7518]	chr4	71296209	71348714	357	0.117	1	1	6.328E-03	1.945E-02	PB-GT
DNAJC11	DnaJ (Hsp40) homolog, subfamily C, member 11 [Source:HGNC Symbol;Acc:25570]	chr1	6694228	6761984	331	0.117	1	1	6.361E-03	1.953E-02	PB-GT
MYO16	myosin XVI [Source:HGNC Symbol;Acc:29822]	chr13	109248500	109860355	4736	1.143	4	2	6.369E-03	1.953E-02	PB-GT
TFRC	transferrin receptor (p90, CD71) [Source:HGNC Symbol;Acc:11763]	chr3	195754054	195809060	358	0.118	1	1	6.395E-03	1.958E-02	PB-GT
ACVR1	activin A receptor, type I [Source:HGNC Symbol;Acc:171]	chr2	158592958	158732374	1006	0.371	2	1	6.453E-03	1.971E-02	PB-GT
P4HA2	prolyl 4-hydroxylase, alpha polypeptide II [Source:HGNC Symbol;Acc:8547]	chr5	131527531	131631008	362	0.118	1	1	6.459E-03	1.971E-02	PB-GT
MAPK9	mitogen-activated protein kinase 9 [Source:HGNC Symbol;Acc:6886]	chr5	179660143	179719099	362	0.118	1	1	6.459E-03	1.971E-02	PB-GT
PARL	presenilin associated, rhomboid-like [Source:HGNC Symbol;Acc:18253]	chr3	183547173	183602721	363	0.119	1	1	6.568E-03	1.999E-02	PB-GT
PRR11	proline rich 11 [Source:HGNC Symbol;Acc:25619]	chr17	57232860	57282066	225	0.119	1	1	6.568E-03	1.999E-02	PB-GT
NUP62CL	nucleoporin 62kDa C-terminal like [Source:HGNC Symbol;Acc:25960]	chrX	106366657	106449670	716	0.119	1	1	6.574E-03	1.999E-02	PB-GT
PCSK6	proprotein convertase subtilisin/kexin type 6 [Source:HGNC Symbol;Acc:8569]	chr15	101840818	102065405	1017	0.374	2	2	6.599E-03	2.004E-02	PB-GT
NAPB	N-ethylmaleimide-sensitive factor attachment protein, beta [Source:HGNC Symbol;Acc:15751]	chr20	23355159	23402125	322	0.120	1	1	6.616E-03	2.007E-02	PB-GT
ERAP1	endoplasmic reticulum aminopeptidase 1 [Source:HGNC Symbol;Acc:18173]	chr5	96096521	96143803	367	0.120	1	1	6.631E-03	2.009E-02	PB-GT
EGFLAM	EGF-like, fibronectin type III and laminin G domains [Source:HGNC Symbol;Acc:26810]	chr5	38258511	38465123	1148	0.375	2	2	6.645E-03	2.011E-02	PB-GT
ST8SIA6	ST8 alpha-N-acetyl-neuraminide alpha-2,8-sialyltransferase 6 [Source:HGNC Symbol;Acc:23317]	chr10	17360382	17496329	871	0.375	2	1	6.657E-03	2.011E-02	PB-GT
MMP20	matrix metallopeptidase 20 [Source:HGNC Symbol;Acc:7167]	chr11	102447566	102496063	330	0.120	1	1	6.659E-03	2.011E-02	PB-GT
YAP1	Yes-associated protein 1 [Source:HGNC Symbol;Acc:16262]	chr11	101981192	102104154	1033	0.376	2	2	6.698E-03	2.017E-02	PB-GT
GFRA2	GDNF family receptor alpha 2 [Source:HGNC Symbol;Acc:4244]	chr8	21547915	21669869	308	0.120	1	1	6.698E-03	2.017E-02	PB-GT
HADHB	hydroxyacyl-CoA dehydrogenase/3-ketoacyl-CoA thiolase/enoyl-CoA hydratase (trifunctional protein), beta subunit [Source:HGNC Symbol;Acc:4803]	chr2	26466038	26513336	327	0.121	1	1	6.707E-03	2.017E-02	PB-GT
ANXA2	annexin A2 [Source:HGNC Symbol;Acc:537]	chr15	60639333	60695082	328	0.121	1	1	6.712E-03	2.017E-02	PB-GT
AGO4	argonaute RISC catalytic component 4 [Source:HGNC Symbol;Acc:18424]	chr1	36273773	36323491	342	0.121	1	1	6.774E-03	2.030E-02	PB-GT
JPH2	junctophilin 2 [Source:HGNC Symbol;Acc:14202]	chr20	42740335	42816218	326	0.121	1	1	6.775E-03	2.030E-02	PB-GT
CDK5RAP2	CDK5 regulatory subunit associated protein 2 [Source:HGNC Symbol;Acc:18672]	chr9	123151147	123342448	1152	0.378	2	1	6.776E-03	2.030E-02	PB-GT
CCDC176	coiled-coil domain containing 176 [Source:HGNC Symbol;Acc:19855]	chr14	74486056	74549566	339	0.122	1	1	6.824E-03	2.041E-02	PB-GT
EEFSEC	eukaryotic elongation factor, selenocysteine-tRNA-specific [Source:HGNC Symbol;Acc:24614]	chr3	127872297	128127485	1153	0.379	2	1	6.833E-03	2.041E-02	PB-GT
PFKP	phosphofructokinase, platelet [Source:HGNC Symbol;Acc:8878]	chr10	3109712	3179904	283	0.122	1	1	6.850E-03	2.041E-02	PB-GT
SMYD4	SET and MYND domain containing 4 [Source:HGNC Symbol;Acc:21067]	chr17	1682779	1733928	230	0.122	1	1	6.851E-03	2.041E-02	PB-GT
STK32A	serine/threonine kinase 32A [Source:HGNC Symbol;Acc:28317]	chr5	146614526	146767415	1161	0.379	2	1	6.852E-03	2.041E-02	PB-GT
CCDC113	coiled-coil domain containing 113 [Source:HGNC Symbol;Acc:25002]	chr16	58265061	58317740	283	0.122	1	1	6.862E-03	2.041E-02	PB-GT
OACYLP	O-acyltransferase like, pseudogene [Source:HGNC Symbol;Acc:44362]	chr18	56663966	56736570	382	0.122	1	1	6.888E-03	2.047E-02	PB-GT
FAM153B	family with sequence similarity 153, member B [Source:HGNC Symbol;Acc:27323]	chr5	175487692	175559261	375	0.122	1	1	6.912E-03	2.049E-02	PB-GT
SNX25	sorting nexin 25 [Source:HGNC Symbol;Acc:21883]	chr4	186125391	186291339	1161	0.380	2	2	6.912E-03	2.049E-02	PB-GT
DYNC1LI1	dynein, cytoplasmic 1, light intermediate chain 1 [Source:HGNC Symbol;Acc:18745]	chr3	32567463	32612366	374	0.123	1	1	6.955E-03	2.058E-02	PB-GT
SH3BGR	SH3 domain binding glutamic acid-rich protein [Source:HGNC Symbol;Acc:10822]	chr21	40817781	40887433	486	0.123	1	1	6.965E-03	2.058E-02	PB-GT
MAPRE2	microtubule-associated protein, RP/EB family, member 2 [Source:HGNC Symbol;Acc:6891]	chr18	32556892	32723434	1192	0.381	2	1	6.966E-03	2.058E-02	PB-GT
GNAI3	guanine nucleotide binding protein (G protein), alpha inhibiting activity polypeptide 3 [Source:HGNC Symbol;Acc:4387]	chr1	110091233	110136975	348	0.123	1	1	7.004E-03	2.067E-02	PB-GT
LTBP2	latent transforming growth factor beta binding protein 2 [Source:HGNC Symbol;Acc:6715]	chr14	74964873	75079306	344	0.123	1	1	7.019E-03	2.069E-02	PB-GT
IPP	intracisternal A particle-promoted polypeptide [Source:HGNC Symbol;Acc:6108]	chr1	46159996	46216322	349	0.124	1	1	7.042E-03	2.074E-02	PB-GT
KDM4B	lysine (K)-specific demethylase 4B [Source:HGNC Symbol;Acc:29136]	chr19	4969125	5153606	360	0.124	1	1	7.052E-03	2.074E-02	PB-GT
ARHGEF37	Rho guanine nucleotide exchange factor (GEF) 37 [Source:HGNC Symbol;Acc:34430]	chr5	148931510	149014531	380	0.124	1	1	7.090E-03	2.083E-02	PB-GT
HERC5	HECT and RLD domain containing E3 ubiquitin protein ligase 5 [Source:HGNC Symbol;Acc:24368]	chr4	89378268	89427314	380	0.124	1	1	7.134E-03	2.092E-02	PB-GT
BTBD3	BTB (POZ) domain containing 3 [Source:HGNC Symbol;Acc:15854]	chr20	11871371	11907257	335	0.125	1	1	7.138E-03	2.092E-02	PB-GT
HIF1A-AS2	HIF1A antisense RNA 2 [Source:HGNC Symbol;Acc:43015]	chr14	62182276	62217815	348	0.125	1	1	7.176E-03	2.100E-02	PB-GT
UBE2N	ubiquitin-conjugating enzyme E2N [Source:HGNC Symbol;Acc:12492]	chr12	93799449	93836038	283	0.125	1	1	7.179E-03	2.100E-02	PB-GT
ARID1B	AT rich interactive domain 1B (SWI1-like) [Source:HGNC Symbol;Acc:18040]	chr6	157099063	157530401	3333	1.178	4	2	7.188E-03	2.100E-02	PB-GT
BLOC1S5	biogenesis of lysosomal organelles complex-1, subunit 5, muted [Source:HGNC Symbol;Acc:18561]	chr6	8013800	8064647	354	0.125	1	1	7.204E-03	2.101E-02	PB-GT
BFSP2	beaded filament structural protein 2, phakinin [Source:HGNC Symbol;Acc:1041]	chr3	133118839	133194066	381	0.125	1	1	7.207E-03	2.101E-02	PB-GT
AMPD3	adenosine monophosphate deaminase 3 [Source:HGNC Symbol;Acc:470]	chr11	10329860	10529126	1063	0.387	2	1	7.240E-03	2.108E-02	PB-GT
KCNQ5	potassium voltage-gated channel, KQT-like subfamily, member 5 [Source:HGNC Symbol;Acc:6299]	chr6	73331520	73908574	4708	1.664	5	2	7.248E-03	2.108E-02	PB-GT
OXCT1	3-oxoacid CoA transferase 1 [Source:HGNC Symbol;Acc:8527]	chr5	41730167	41870621	1186	0.387	2	2	7.260E-03	2.109E-02	PB-GT
VPS54	vacuolar protein sorting 54 homolog (S. cerevisiae) [Source:HGNC Symbol;Acc:18652]	chr2	64120035	64246206	1053	0.388	2	2	7.306E-03	2.120E-02	PB-GT
VRK1	vaccinia related kinase 1 [Source:HGNC Symbol;Acc:12718]	chr14	97263641	97398059	1083	0.389	2	1	7.328E-03	2.121E-02	PB-GT
ATL3	atlastin GTPase 3 [Source:HGNC Symbol;Acc:24526]	chr11	63391559	63439393	347	0.126	1	1	7.333E-03	2.121E-02	PB-GT
SF3B3	splicing factor 3b, subunit 3, 130kDa [Source:HGNC Symbol;Acc:10770]	chr16	70557691	70608820	293	0.126	1	1	7.335E-03	2.121E-02	PB-GT
ROS1	c-ros oncogene 1 , receptor tyrosine kinase [Source:HGNC Symbol;Acc:10261]	chr6	117609463	117747018	1101	0.389	2	2	7.356E-03	2.125E-02	PB-GT
SMARCE1	SWI/SNF related, matrix associated, actin dependent regulator of chromatin, subfamily e, member 1 [Source:HGNC Symbol;Acc:11109]	chr17	38781214	38804760	239	0.127	1	1	7.374E-03	2.126E-02	PB-GT
SYNJ2BP	synaptojanin 2 binding protein [Source:HGNC Symbol;Acc:18955]	chr14	70838148	70883778	353	0.127	1	1	7.375E-03	2.126E-02	PB-GT
ELAVL4	ELAV (embryonic lethal, abnormal vision, Drosophila)-like 4 [Source:HGNC Symbol;Acc:3315]	chr1	50513686	50669458	1100	0.390	2	2	7.386E-03	2.127E-02	PB-GT
CRYBB2P1	crystallin, beta B2 pseudogene 1 [Source:HGNC Symbol;Acc:2399]	chr22	25844072	25916821	301	0.127	1	1	7.422E-03	2.135E-02	PB-GT
CREB5	cAMP responsive element binding protein 5 [Source:HGNC Symbol;Acc:16844]	chr7	28338940	28865511	3559	1.189	4	1	7.474E-03	2.147E-02	PB-GT
VWA3A	von Willebrand factor A domain containing 3A [Source:HGNC Symbol;Acc:27088]	chr16	22103859	22168287	296	0.128	1	1	7.479E-03	2.147E-02	PB-GT
YWHAZ	tyrosine 3-monooxygenase/tryptophan 5-monooxygenase activation protein, zeta polypeptide [Source:HGNC Symbol;Acc:12855]	chr8	101928753	101965616	327	0.128	1	1	7.513E-03	2.154E-02	PB-GT
FAM115A	family with sequence similarity 115, member A [Source:HGNC Symbol;Acc:22201]	chr7	143548468	143599291	383	0.128	1	1	7.525E-03	2.154E-02	PB-GT
PPARG	peroxisome proliferator-activated receptor gamma [Source:HGNC Symbol;Acc:9236]	chr3	12328867	12475855	1195	0.393	2	2	7.531E-03	2.154E-02	PB-GT
LURAP1L	leucine rich adaptor protein 1-like [Source:HGNC Symbol;Acc:31452]	chr9	12775020	12822130	391	0.128	1	1	7.541E-03	2.155E-02	PB-GT
KCNK1	potassium channel, subfamily K, member 1 [Source:HGNC Symbol;Acc:6272]	chr1	233749750	233808258	362	0.128	1	1	7.554E-03	2.156E-02	PB-GT
GLIS3	GLIS family zinc finger 3 [Source:HGNC Symbol;Acc:28510]	chr9	3824127	4348392	3642	1.194	4	2	7.580E-03	2.161E-02	PB-GT
EML1	echinoderm microtubule associated protein like 1 [Source:HGNC Symbol;Acc:3330]	chr14	100204030	100408397	1098	0.394	2	2	7.607E-03	2.166E-02	PB-GT
UCHL3	ubiquitin carboxyl-terminal esterase L3 (ubiquitin thiolesterase) [Source:HGNC Symbol;Acc:12515]	chr13	76123619	76180085	535	0.129	1	1	7.658E-03	2.174E-02	PB-GT
PAX2	paired box 2 [Source:HGNC Symbol;Acc:8616]	chr10	102495360	102589698	300	0.129	1	1	7.660E-03	2.174E-02	PB-GT
POLD3	polymerase (DNA-directed), delta 3, accessory subunit [Source:HGNC Symbol;Acc:20932]	chr11	74204896	74380162	1086	0.395	2	2	7.673E-03	2.174E-02	PB-GT
DNAJB4	DnaJ (Hsp40) homolog, subfamily B, member 4 [Source:HGNC Symbol;Acc:14886]	chr1	78444859	78483648	365	0.129	1	1	7.674E-03	2.174E-02	PB-GT
TMEM209	transmembrane protein 209 [Source:HGNC Symbol;Acc:21898]	chr7	129804555	129847610	387	0.129	1	1	7.677E-03	2.174E-02	PB-GT
IPO5	importin 5 [Source:HGNC Symbol;Acc:6402]	chr13	98605912	98676551	538	0.130	1	1	7.740E-03	2.188E-02	PB-GT
BCO2	beta-carotene oxygenase 2 [Source:HGNC Symbol;Acc:18503]	chr11	112046190	112095422	357	0.130	1	1	7.743E-03	2.188E-02	PB-GT
WDR65	WD repeat domain 65 [Source:HGNC Symbol;Acc:26485]	chr1	43637820	43720029	367	0.130	1	1	7.755E-03	2.189E-02	PB-GT
MAPKAPK5	mitogen-activated protein kinase-activated protein kinase 5 [Source:HGNC Symbol;Acc:6889]	chr12	112279782	112334343	295	0.130	1	1	7.774E-03	2.192E-02	PB-GT
S100Z	S100 calcium binding protein Z [Source:HGNC Symbol;Acc:30367]	chr5	76145826	76217475	399	0.130	1	1	7.784E-03	2.193E-02	PB-GT
PITPNM3	PITPNM family member 3 [Source:HGNC Symbol;Acc:21043]	chr17	6354584	6459814	246	0.130	1	1	7.793E-03	2.193E-02	PB-GT
C20orf112	chromosome 20 open reading frame 112 [Source:HGNC Symbol;Acc:16106]	chr20	31030862	31172876	351	0.130	1	1	7.806E-03	2.194E-02	PB-GT
RCAN1	regulator of calcineurin 1 [Source:HGNC Symbol;Acc:3040]	chr21	35885440	35987441	518	0.131	1	1	7.870E-03	2.208E-02	PB-GT
NKD1	naked cuticle homolog 1 (Drosophila) [Source:HGNC Symbol;Acc:17045]	chr16	50582241	50670647	304	0.131	1	1	7.871E-03	2.208E-02	PB-GT
CSE1L	CSE1 chromosome segregation 1-like (yeast) [Source:HGNC Symbol;Acc:2431]	chr20	47662849	47713489	354	0.132	1	1	7.934E-03	2.223E-02	PB-GT
FGD6	FYVE, RhoGEF and PH domain containing 6 [Source:HGNC Symbol;Acc:21740]	chr12	95470525	95611258	907	0.400	2	1	7.941E-03	2.223E-02	PB-GT
PASD1	PAS domain containing 1 [Source:HGNC Symbol;Acc:20686]	chrX	150732094	150845211	791	0.132	1	1	7.958E-03	2.225E-02	PB-GT
STAT1	signal transducer and activator of transcription 1, 91kDa [Source:HGNC Symbol;Acc:11362]	chr2	191829084	191885686	358	0.132	1	1	7.978E-03	2.228E-02	PB-GT
KIAA0556	KIAA0556 [Source:HGNC Symbol;Acc:29068]	chr16	27561454	27791690	932	0.402	2	1	8.020E-03	2.235E-02	PB-GT
CYB5B	cytochrome b5 type B (outer mitochondrial membrane) [Source:HGNC Symbol;Acc:24374]	chr16	69458428	69500169	307	0.132	1	1	8.020E-03	2.235E-02	PB-GT
GOLGA5	golgin A5 [Source:HGNC Symbol;Acc:4428]	chr14	93260576	93306308	369	0.132	1	1	8.028E-03	2.235E-02	PB-GT
STPG1	sperm-tail PG-rich repeat containing 1 [Source:HGNC Symbol;Acc:28070]	chr1	24683489	24743424	375	0.133	1	1	8.082E-03	2.247E-02	PB-GT
NEDD9	neural precursor cell expressed, developmentally down-regulated 9 [Source:HGNC Symbol;Acc:7733]	chr6	11183531	11382581	1141	0.403	2	1	8.103E-03	2.251E-02	PB-GT
TMPRSS12	transmembrane (C-terminal) protease, serine 12 [Source:HGNC Symbol;Acc:28779]	chr12	51236703	51281667	302	0.133	1	1	8.131E-03	2.256E-02	PB-GT
LIMCH1	LIM and calponin homology domains 1 [Source:HGNC Symbol;Acc:29191]	chr4	41361624	41702061	2367	0.775	3	2	8.168E-03	2.264E-02	PB-GT
TTLL11-IT1	TTLL11 intronic transcript 1 (non-protein coding) [Source:HGNC Symbol;Acc:24214]	chr9	124646915	124725998	409	0.134	1	1	8.219E-03	2.276E-02	PB-GT
GOPC	golgi-associated PDZ and coiled-coil motif containing [Source:HGNC Symbol;Acc:17643]	chr6	117639374	117923691	2199	0.777	3	2	8.233E-03	2.277E-02	PB-GT
NKAPP1	NFKB activating protein pseudogene 1 [Source:HGNC Symbol;Acc:26706]	chrX	119254863	119379122	806	0.134	1	1	8.249E-03	2.277E-02	PB-GT
PRRG1	proline rich Gla (G-carboxyglutamic acid) 1 [Source:HGNC Symbol;Acc:9469]	chrX	37208528	37316548	806	0.134	1	1	8.249E-03	2.277E-02	PB-GT
TMX4	thioredoxin-related transmembrane protein 4 [Source:HGNC Symbol;Acc:25237]	chr20	7957995	8000476	362	0.135	1	1	8.281E-03	2.283E-02	PB-GT
PCDH19	protocadherin 19 [Source:HGNC Symbol;Acc:14270]	chrX	99546642	99665271	808	0.135	1	1	8.288E-03	2.283E-02	PB-GT
PIK3CB	phosphatidylinositol-4,5-bisphosphate 3-kinase, catalytic subunit beta [Source:HGNC Symbol;Acc:8976]	chr3	138372860	138553780	1240	0.407	2	1	8.323E-03	2.288E-02	PB-GT
ICK	intestinal cell (MAK-like) kinase [Source:HGNC Symbol;Acc:21219]	chr6	52866077	52926600	382	0.135	1	1	8.334E-03	2.288E-02	PB-GT
LANCL3	LanC lantibiotic synthetase component C-like 3 (bacterial) [Source:HGNC Symbol;Acc:24767]	chrX	37430822	37543716	811	0.135	1	1	8.347E-03	2.288E-02	PB-GT
TRIM2	tripartite motif containing 2 [Source:HGNC Symbol;Acc:15974]	chr4	154073494	154260472	1245	0.408	2	2	8.353E-03	2.288E-02	PB-GT
ARHGEF38	Rho guanine nucleotide exchange factor (GEF) 38 [Source:HGNC Symbol;Acc:25968]	chr4	106473777	106616835	1245	0.408	2	1	8.353E-03	2.288E-02	PB-GT
NRCAM	neuronal cell adhesion molecule [Source:HGNC Symbol;Acc:7994]	chr7	107788068	108097161	2336	0.781	3	1	8.359E-03	2.288E-02	PB-GT
ARID1A	AT rich interactive domain 1A (SWI-like) [Source:HGNC Symbol;Acc:11110]	chr1	27022524	27108595	382	0.135	1	1	8.372E-03	2.289E-02	PB-GT
DLG1	discs, large homolog 1 (Drosophila) [Source:HGNC Symbol;Acc:2900]	chr3	196769431	197026171	2379	0.781	3	2	8.385E-03	2.290E-02	PB-GT
RYK	receptor-like tyrosine kinase [Source:HGNC Symbol;Acc:10481]	chr3	133794023	133969689	1245	0.409	2	2	8.414E-03	2.296E-02	PB-GT

table S1. Genes with significant transposon integrations in DLD-1 *KRAS*$^{wt/-}$ cells after selection in low glucose medium.

Gene ID	Description	Chrom	Start	End	# TTAA sites	Expected	Observed	Libraries	*P*	FDR	Transposon
IL34	interleukin 34 [Source:HGNC Symbol;Acc:28529]	chr16	70613798	70694585	315	0.136	1	1	8.425E-03	2.296E-02	PB-GT
GCH1	GTP cyclohydrolase 1 [Source:HGNC Symbol;Acc:4193]	chr14	55308726	55369570	379	0.136	1	1	8.449E-03	2.301E-02	PB-GT
EIF3A	eukaryotic translation initiation factor 3, subunit A [Source:HGNC Symbol;Acc:3271]	chr10	120794356	120840396	316	0.136	1	1	8.461E-03	2.301E-02	PB-GT
NDC80	NDC80 kinetochore complex component [Source:HGNC Symbol;Acc:16909]	chr18	2571510	2616634	426	0.136	1	1	8.487E-03	2.303E-02	PB-GT
RNF217	ring finger protein 217 [Source:HGNC Symbol;Acc:21487]	chr6	125283691	125413779	1161	0.410	2	2	8.492E-03	2.303E-02	PB-GT
AP1B1	adaptor-related protein complex 1, beta 1 subunit [Source:HGNC Symbol;Acc:554]	chr22	29723669	29819168	323	0.136	1	1	8.495E-03	2.303E-02	PB-GT
RUVBL1	RuvB-like 1 (E. coli) [Source:HGNC Symbol;Acc:10474]	chr3	127783621	127872757	416	0.137	1	1	8.527E-03	2.308E-02	PB-GT
DCP2	DCP2 decapping enzyme homolog (S. cerevisiae) [Source:HGNC Symbol;Acc:24452]	chr5	112312399	112356667	419	0.137	1	1	8.547E-03	2.308E-02	PB-GT
ABCC4	ATP-binding cassette, sub-family C (CFTR/MRP), member 4 [Source:HGNC Symbol;Acc:55]	chr13	95672083	95953687	1704	0.411	2	2	8.552E-03	2.308E-02	PB-GT
MOK	MOK protein kinase [Source:HGNC Symbol;Acc:9833]	chr14	102690837	102771537	382	0.137	1	1	8.577E-03	2.308E-02	PB-GT
GSPT1	G1 to S phase transition 1 [Source:HGNC Symbol;Acc:4621]	chr16	11961985	12009939	318	0.137	1	1	8.579E-03	2.308E-02	PB-GT
MARK1	MAP/microtubule affinity-regulating kinase 1 [Source:HGNC Symbol;Acc:6896]	chr1	220701568	220837803	1163	0.412	2	1	8.588E-03	2.308E-02	PB-GT
MS4A6E	membrane-spanning 4-domains, subfamily A, member 6E [Source:HGNC Symbol;Acc:14285]	chr11	60102304	60164069	377	0.137	1	1	8.594E-03	2.308E-02	PB-GT
DMC1	DMC1 dosage suppressor of mck1 homolog, meiosis-specific homologous recombination (yeast) [Source:HGNC Symbol;Acc:2927]	chr22	38914954	38966291	325	0.137	1	1	8.596E-03	2.308E-02	PB-GT
CBX5	chromobox homolog 5 [Source:HGNC Symbol;Acc:1555]	chr12	54624724	54673886	311	0.137	1	1	8.600E-03	2.308E-02	PB-GT
HVCN1	hydrogen voltage-gated channel 1 [Source:HGNC Symbol;Acc:28240]	chr12	111065646	111142755	311	0.137	1	1	8.600E-03	2.308E-02	PB-GT
WBP1L	WW domain binding protein 1-like [Source:HGNC Symbol;Acc:23510]	chr10	104503727	104576021	319	0.137	1	1	8.615E-03	2.308E-02	PB-GT
EMB	embigin [Source:HGNC Symbol;Acc:30465]	chr5	49692026	49739082	421	0.137	1	1	8.625E-03	2.308E-02	PB-GT
TTTY14	testis-specific transcript, Y-linked 14 (non-protein coding) [Source:HGNC Symbol;Acc:18495]	chrY	21034387	21239302	1322	0.137	1	1	8.629E-03	2.308E-02	PB-GT
CALCB	calcitonin-related polypeptide beta [Source:HGNC Symbol;Acc:1438]	chr11	14926543	15103888	1136	0.413	2	2	8.665E-03	2.316E-02	PB-GT
AQP4-AS1	AQP4 antisense RNA 1 [Source:HGNC Symbol;Acc:26399]	chr18	24235706	24770662	3860	1.235	4	2	8.696E-03	2.322E-02	PB-GT
ACOXL	acyl-CoA oxidase-like [Source:HGNC Symbol;Acc:25621]	chr2	111490150	111875799	2144	0.790	3	2	8.714E-03	2.324E-02	PB-GT
ANKRD44-IT1	ANKRD44 intronic transcript 1 (non-protein coding) [Source:HGNC Symbol;Acc:41477]	chr2	198115582	198167243	376	0.139	1	1	8.762E-03	2.334E-02	PB-GT
USP47	ubiquitin specific peptidase 47 [Source:HGNC Symbol;Acc:20076]	chr11	11862970	11980870	1141	0.415	2	1	8.769E-03	2.334E-02	PB-GT
MAOB	monoamine oxidase B [Source:HGNC Symbol;Acc:6834]	chrX	43625858	43741693	833	0.139	1	1	8.785E-03	2.336E-02	PB-GT
SPPL3	signal peptide peptidase like 3 [Source:HGNC Symbol;Acc:30424]	chr12	121200313	121342174	942	0.416	2	1	8.796E-03	2.336E-02	PB-GT
GALNT2	UDP-N-acetyl-alpha-D-galactosamine:polypeptide N-acetylgalactosaminyltransferase 2 (GalNAc-T2) [Source:HGNC Symbol;Acc:4124]	chr1	230193536	230417870	1174	0.416	2	1	8.808E-03	2.337E-02	PB-GT
ALOX5	arachidonate 5-lipoxygenase [Source:HGNC Symbol;Acc:435]	chr10	45869661	45941561	323	0.139	1	1	8.822E-03	2.338E-02	PB-GT
RAPGEF4	Rap guanine nucleotide exchange factor (GEF) 4 [Source:HGNC Symbol;Acc:16626]	chr2	173600002	173917621	2153	0.794	3	2	8.838E-03	2.340E-02	PB-GT
HEG1	heart development protein with EGF-like domains 1 [Source:HGNC Symbol;Acc:29227]	chr3	124684554	124774802	425	0.140	1	1	8.883E-03	2.350E-02	PB-GT
REL	v-rel reticuloendotheliosis viral oncogene homolog (avian) [Source:HGNC Symbol;Acc:9954]	chr2	61108656	61150645	379	0.140	1	1	8.896E-03	2.351E-02	PB-GT
OSTF1	osteoclast stimulating factor 1 [Source:HGNC Symbol;Acc:8510]	chr9	77703459	77762181	427	0.140	1	1	8.924E-03	2.356E-02	PB-GT
SCTR	secretin receptor [Source:HGNC Symbol;Acc:10608]	chr2	120197419	120282070	380	0.140	1	1	8.941E-03	2.357E-02	PB-GT
FAM214A	family with sequence similarity 214, member A [Source:HGNC Symbol;Acc:25609]	chr15	52873514	53002014	1138	0.418	2	1	8.948E-03	2.357E-02	PB-GT
NCOA3	nuclear receptor coactivator 3 [Source:HGNC Symbol;Acc:7670]	chr20	46130601	46285621	1127	0.419	2	1	8.979E-03	2.362E-02	PB-GT
MKRN3	makorin ring finger protein 3 [Source:HGNC Symbol;Acc:7114]	chr15	23810454	23873064	382	0.140	1	1	8.985E-03	2.362E-02	PB-GT
TFG	TRK-fused gene [Source:HGNC Symbol;Acc:11758]	chr3	100428205	100467810	428	0.141	1	1	9.003E-03	2.363E-02	PB-GT
WBP2NL	WBP2 N-terminal like [Source:HGNC Symbol;Acc:28389]	chr22	42394729	42454460	333	0.141	1	1	9.004E-03	2.363E-02	PB-GT
RNF128	ring finger protein 128, E3 ubiquitin protein ligase [Source:HGNC Symbol;Acc:21153]	chrX	105937024	106040223	845	0.141	1	1	9.028E-03	2.367E-02	PB-GT
STK39	serine threonine kinase 39 [Source:HGNC Symbol;Acc:17717]	chr2	168810530	169104651	2171	0.800	3	1	9.091E-03	2.381E-02	PB-GT
CLEC9A	C-type lectin domain family 9, member A [Source:HGNC Symbol;Acc:26705]	chr12	10183276	10218565	321	0.142	1	1	9.135E-03	2.390E-02	PB-GT
MAP2K6	mitogen-activated protein kinase kinase 6 [Source:HGNC Symbol;Acc:6846]	chr17	67410839	67539472	796	0.422	2	2	9.148E-03	2.391E-02	PB-GT
MRPS5	mitochondrial ribosomal protein S5 [Source:HGNC Symbol;Acc:14498]	chr2	95752952	95815179	385	0.142	1	1	9.167E-03	2.392E-02	PB-GT
ZNF730	zinc finger protein 730 [Source:HGNC Symbol;Acc:32470]	chr19	23258012	23330021	413	0.142	1	1	9.171E-03	2.392E-02	PB-GT
PTPRC	protein tyrosine phosphatase, receptor type, C [Source:HGNC Symbol;Acc:9666]	chr1	198607801	198726545	1193	0.423	2	2	9.197E-03	2.396E-02	PB-GT
SH3BP5	SH3-domain binding protein 5 (BTK-associated) [Source:HGNC Symbol;Acc:10827]	chr3	15296360	15382875	433	0.142	1	1	9.204E-03	2.396E-02	PB-GT
OPHN1	oligophrenin 1 [Source:HGNC Symbol;Acc:8148]	chrX	67262186	67653755	2541	0.423	2	1	9.238E-03	2.402E-02	PB-GT
CCNYL1	cyclin Y-like 1 [Source:HGNC Symbol;Acc:26868]	chr2	208576264	208626563	387	0.143	1	1	9.258E-03	2.405E-02	PB-GT
SSX2IP	synovial sarcoma, X breakpoint 2 interacting protein [Source:HGNC Symbol;Acc:16509]	chr1	85109390	85156486	404	0.143	1	1	9.317E-03	2.416E-02	PB-GT
SLC2A12	solute carrier family 2 (facilitated glucose transporter), member 12 [Source:HGNC Symbol;Acc:18067]	chr6	134309835	134373774	405	0.143	1	1	9.318E-03	2.416E-02	PB-GT
VCAN-AS1	VCAN antisense RNA 1 [Source:HGNC Symbol;Acc:40163]	chr5	82827171	82877139	439	0.143	1	1	9.343E-03	2.417E-02	PB-GT
IL31RA	interleukin 31 receptor A [Source:HGNC Symbol;Acc:18969]	chr5	55147207	55218678	439	0.143	1	1	9.343E-03	2.417E-02	PB-GT
KIAA1324	KIAA1324 [Source:HGNC Symbol;Acc:29618]	chr1	109656301	109749401	405	0.143	1	1	9.361E-03	2.419E-02	PB-GT
EFR3A	EFR3 homolog A (S. cerevisiae) [Source:HGNC Symbol;Acc:28970]	chr8	132916335	133025889	1088	0.426	2	1	9.366E-03	2.419E-02	PB-GT
CASZ1	castor zinc finger 1 [Source:HGNC Symbol;Acc:26002]	chr1	10696661	10856707	406	0.144	1	1	9.405E-03	2.424E-02	PB-GT
KCNQ5-IT1	KCNQ5 intronic transcript 1 (non-protein coding) [Source:HGNC Symbol;Acc:41354]	chr6	73340223	73388282	407	0.144	1	1	9.406E-03	2.424E-02	PB-GT
CHST15	carbohydrate (N-acetylgalactosamine 4-sulfate 6-O) sulfotransferase 15 [Source:HGNC Symbol;Acc:18137]	chr10	125767184	125853206	336	0.145	1	1	9.511E-03	2.447E-02	PB-GT
SBSPON	somatomedin B and thrombospondin, type 1 domain containing [Source:HGNC Symbol;Acc:30362]	chr8	73976775	74036323	370	0.145	1	1	9.513E-03	2.447E-02	PB-GT
IQSEC1	IQ motif and Sec7 domain 1 [Source:HGNC Symbol;Acc:29112]	chr3	12938719	13114617	441	0.145	1	1	9.531E-03	2.449E-02	PB-GT
C10orf107	chromosome 10 open reading frame 107 [Source:HGNC Symbol;Acc:28678]	chr10	63422719	63526524	995	0.428	2	2	9.542E-03	2.449E-02	PB-GT
PRKAG2	protein kinase, AMP-activated, gamma 2 non-catalytic subunit [Source:HGNC Symbol;Acc:9386]	chr7	151253197	151574210	1283	0.429	2	2	9.560E-03	2.452E-02	PB-GT
EFCAB14	EF-hand calcium binding domain 14 [Source:HGNC Symbol;Acc:29051]	chr1	47124366	47184824	410	0.145	1	1	9.582E-03	2.453E-02	PB-GT
OPN5	opsin 5 [Source:HGNC Symbol;Acc:19992]	chr6	47749718	47800516	411	0.145	1	1	9.582E-03	2.453E-02	PB-GT
SLC17A5	solute carrier family 17 (anion/sugar transporter), member 5 [Source:HGNC Symbol;Acc:10933]	chr6	74303102	74363878	412	0.146	1	1	9.627E-03	2.459E-02	PB-GT
RACGAP1	Rac GTPase activating protein 1 [Source:HGNC Symbol;Acc:9804]	chr12	50370706	50426919	330	0.146	1	1	9.629E-03	2.459E-02	PB-GT
GTF3C1	general transcription factor IIIC, polypeptide 1, alpha 220kDa [Source:HGNC Symbol;Acc:4664]	chr16	27470876	27561234	338	0.146	1	1	9.637E-03	2.459E-02	PB-GT
UBE2V2	ubiquitin-conjugating enzyme E2 variant 2 [Source:HGNC Symbol;Acc:12495]	chr8	48920960	48977268	373	0.146	1	1	9.661E-03	2.463E-02	PB-GT
PRPF18	PRP18 pre-mRNA processing factor 18 homolog (S. cerevisiae) [Source:HGNC Symbol;Acc:17351]	chr10	13628927	13672868	339	0.146	1	1	9.674E-03	2.464E-02	PB-GT
RHBDD1	rhomboid domain containing 1 [Source:HGNC Symbol;Acc:23081]	chr2	227700297	227863931	1170	0.431	2	1	9.710E-03	2.470E-02	PB-GT
ARNT	aryl hydrocarbon receptor nuclear translocator [Source:HGNC Symbol;Acc:700]	chr1	150782181	150849244	413	0.146	1	1	9.716E-03	2.470E-02	PB-GT
EMBP1	embigin pseudogene 1 [Source:HGNC Symbol;Acc:38661]	chr1	121261143	121310591	414	0.147	1	1	9.761E-03	2.479E-02	PB-GT
IFI16	interferon, gamma-inducible protein 16 [Source:HGNC Symbol;Acc:5395]	chr1	158969758	159024945	416	0.147	1	1	9.851E-03	2.497E-02	PB-GT
ACER3	alkaline ceramidase 3 [Source:HGNC Symbol;Acc:16066]	chr11	76571911	76737841	1192	0.434	2	2	9.863E-03	2.497E-02	PB-GT
CAPN7	calpain 7 [Source:HGNC Symbol;Acc:1484]	chr3	15247659	15294425	449	0.147	1	1	9.863E-03	2.497E-02	PB-GT
TTC40	tetratricopeptide repeat domain 40 [Source:HGNC Symbol;Acc:25247]	chr10	134621896	134756327	343	0.148	1	1	9.892E-03	2.500E-02	PB-GT
LANCL2	LanC lantibiotic synthetase component C-like 2 (bacterial) [Source:HGNC Symbol;Acc:6509]	chr7	55433141	55501435	442	0.148	1	1	9.893E-03	2.500E-02	PB-GT
DHX8	DEAH (Asp-Glu-Ala-His) box polypeptide 8 [Source:HGNC Symbol;Acc:2749]	chr17	41561233	41621831	279	0.148	1	1	9.910E-03	2.502E-02	PB-GT
VAPA	VAMP (vesicle-associated membrane protein)-associated protein A, 33kDa [Source:HGNC Symbol;Acc:12648]	chr18	9913999	9960018	464	0.148	1	1	9.988E-03	2.519E-02	PB-GT
C2orf61	chromosome 2 open reading frame 61 [Source:HGNC Symbol;Acc:26850]	chr2	47314130	47382517	403	0.149	1	1	1.000E-02	2.520E-02	PB-GT
SLC35F3	solute carrier family 35, member F3 [Source:HGNC Symbol;Acc:23616]	chr1	234040679	234460262	2326	0.824	3	1	1.003E-02	2.526E-02	PB-GT
AHNAK	AHNAK nucleoprotein [Source:HGNC Symbol;Acc:347]	chr11	62201016	62323707	410	0.149	1	1	1.008E-02	2.535E-02	PB-GT
MAP3K9	mitogen-activated protein kinase kinase kinase 9 [Source:HGNC Symbol;Acc:6861]	chr14	71189243	71276251	416	0.149	1	1	1.009E-02	2.535E-02	PB-GT
PGM1	phosphoglucomutase 1 [Source:HGNC Symbol;Acc:8905]	chr1	64058947	64125916	422	0.150	1	1	1.012E-02	2.541E-02	PB-GT
AAGAB	alpha- and gamma-adaptin binding protein [Source:HGNC Symbol;Acc:25662]	chr15	67493371	67547533	407	0.150	1	1	1.014E-02	2.541E-02	PB-GT
NUP35	nucleoporin 35kDa [Source:HGNC Symbol;Acc:29797]	chr2	183982241	184026408	406	0.150	1	1	1.014E-02	2.541E-02	PB-GT
SYNJ2	synaptojanin 2 [Source:HGNC Symbol;Acc:11504]	chr6	158402888	158520208	424	0.150	1	1	1.017E-02	2.543E-02	PB-GT
IARS2	isoleucyl-tRNA synthetase 2, mitochondrial [Source:HGNC Symbol;Acc:29685]	chr1	220267444	220321380	423	0.150	1	1	1.017E-02	2.543E-02	PB-GT
KIAA0825	KIAA0825 [Source:HGNC Symbol;Acc:28532]	chr5	93488671	93954309	3935	1.285	4	2	1.018E-02	2.543E-02	PB-GT
NSL1	NSL1, MIND kinetochore complex component, homolog (S. cerevisiae) [Source:HGNC Symbol;Acc:24548]	chr1	212899495	212965124	424	0.150	1	1	1.021E-02	2.549E-02	PB-GT
SSUH2	ssu-2 homolog (C. elegans) [Source:HGNC Symbol;Acc:24809]	chr3	8661086	8786726	458	0.150	1	1	1.024E-02	2.552E-02	PB-GT
GIPC2	GIPC PDZ domain containing family, member 2 [Source:HGNC Symbol;Acc:18177]	chr1	78445226	78604133	1242	0.440	2	1	1.025E-02	2.552E-02	PB-GT
SLC9A8	solute carrier family 9, subfamily A (NHE8, cation proton antiporter 8), member 8 [Source:HGNC Symbol;Acc:20728]	chr20	48429250	48508779	406	0.151	1	1	1.030E-02	2.562E-02	PB-GT
KDM1B	lysine (K)-specific demethylase 1B [Source:HGNC Symbol;Acc:21577]	chr6	18155560	18224084	427	0.151	1	1	1.030E-02	2.562E-02	PB-GT
PAXIP1	PAX interacting (with transcription-activation domain) protein 1 [Source:HGNC Symbol;Acc:8624]	chr7	154735397	154794794	452	0.151	1	1	1.032E-02	2.564E-02	PB-GT
HTR5BP	5-hydroxytryptamine (serotonin) receptor 5B, pseudogene [Source:HGNC Symbol;Acc:16291]	chr2	118617003	118692518	411	0.152	1	1	1.038E-02	2.573E-02	PB-GT
UBE2F	ubiquitin-conjugating enzyme E2F (putative) [Source:HGNC Symbol;Acc:12480]	chr2	238875469	238951236	411	0.152	1	1	1.038E-02	2.573E-02	PB-GT
GCSAML	germinal center-associated, signaling and motility-like [Source:HGNC Symbol;Acc:29583]	chr1	247670360	247740992	428	0.152	1	1	1.040E-02	2.575E-02	PB-GT
PHGDH	phosphoglycerate dehydrogenase [Source:HGNC Symbol;Acc:8923]	chr1	120202421	120286838	429	0.152	1	1	1.044E-02	2.584E-02	PB-GT
TMEM246	transmembrane protein 246 [Source:HGNC Symbol;Acc:28180]	chr9	104235453	104295819	465	0.152	1	1	1.050E-02	2.593E-02	PB-GT
FNTB	farnesyltransferase, CAAX box, beta [Source:HGNC Symbol;Acc:3785]	chr14	65453438	65529368	425	0.152	1	1	1.051E-02	2.593E-02	PB-GT
LANCL1	LanC lantibiotic synthetase component C-like 1 (bacterial) [Source:HGNC Symbol;Acc:6508]	chr2	211295973	211342376	414	0.153	1	1	1.053E-02	2.593E-02	PB-GT
RBM44	RNA binding motif protein 44 [Source:HGNC Symbol;Acc:24756]	chr2	238707032	238751451	414	0.153	1	1	1.053E-02	2.593E-02	PB-GT
ABLIM2	actin binding LIM protein family, member 2 [Source:HGNC Symbol;Acc:19195]	chr4	7967039	8160559	466	0.153	1	1	1.053E-02	2.593E-02	PB-GT
CCNYL3	cyclin Y-like 3 [Source:HGNC Symbol;Acc:33206]	chr16	34262756	34318684	355	0.153	1	1	1.058E-02	2.603E-02	PB-GT
TRIM71	tripartite motif containing 71, E3 ubiquitin protein ligase [Source:HGNC Symbol;Acc:32669]	chr3	32859510	32938323	468	0.154	1	1	1.067E-02	2.623E-02	PB-GT
ASB7	ankyrin repeat and SOCS box containing 7 [Source:HGNC Symbol;Acc:17182]	chr15	101142739	101191910	419	0.154	1	1	1.071E-02	2.628E-02	PB-GT
PREPL	prolyl endopeptidase-like [Source:HGNC Symbol;Acc:30228]	chr2	44543420	44589001	418	0.154	1	1	1.072E-02	2.628E-02	PB-GT
MSRA	methionine sulfoxide reductase A [Source:HGNC Symbol;Acc:7377]	chr8	9911778	10286401	2149	0.840	3	2	1.072E-02	2.628E-02	PB-GT
SLC9A4	solute carrier family 9, subfamily A (NHE4, cation proton antiporter 4), member 4 [Source:HGNC Symbol;Acc:11077]	chr2	103089762	103150431	419	0.154	1	1	1.077E-02	2.635E-02	PB-GT
RAD52	RAD52 homolog (S. cerevisiae) [Source:HGNC Symbol;Acc:9824]	chr12	1021243	1100356	350	0.154	1	1	1.077E-02	2.635E-02	PB-GT
GATA4	GATA binding protein 4 [Source:HGNC Symbol;Acc:4173]	chr8	11534468	11617511	396	0.155	1	1	1.082E-02	2.646E-02	PB-GT
RANBP3L	RAN binding protein 3-like [Source:HGNC Symbol;Acc:26353]	chr5	36248536	36302216	475	0.155	1	1	1.085E-02	2.650E-02	PB-GT
AIM1	absent in melanoma 1 [Source:HGNC Symbol;Acc:356]	chr6	106959730	107018326	439	0.155	1	1	1.086E-02	2.650E-02	PB-GT
ZCCHC2	zinc finger, CCHC domain containing 2 [Source:HGNC Symbol;Acc:22916]	chr18	60190240	60254942	486	0.156	1	1	1.091E-02	2.658E-02	PB-GT
LCP1	lymphocyte cytosolic protein 1 (L-plastin) [Source:HGNC Symbol;Acc:6528]	chr13	46700055	46786006	645	0.156	1	1	1.094E-02	2.663E-02	PB-GT
B3GALT5	UDP-Gal:betaGlcNAc beta 1,3-galactosyltransferase, polypeptide 5 [Source:HGNC Symbol;Acc:920]	chr21	40928369	41034816	616	0.156	1	1	1.095E-02	2.664E-02	PB-GT
SEC23IP	SEC23 interacting protein [Source:HGNC Symbol;Acc:17018]	chr10	121652223	121702014	363	0.156	1	1	1.102E-02	2.676E-02	PB-GT
SLCO2A1	solute carrier organic anion transporter family, member 2A1 [Source:HGNC Symbol;Acc:10955]	chr3	133651540	133771028	476	0.156	1	1	1.102E-02	2.676E-02	PB-GT
C1orf63	chromosome 1 open reading frame 63 [Source:HGNC Symbol;Acc:25234]	chr1	25568728	25664704	444	0.157	1	1	1.115E-02	2.704E-02	PB-GT
CYFIP1	cytoplasmic FMR1 interacting protein 1 [Source:HGNC Symbol;Acc:13759]	chr15	22892005	23006016	429	0.158	1	1	1.120E-02	2.715E-02	PB-GT
ACMSD	aminocarboxymuconate semialdehyde decarboxylase [Source:HGNC Symbol;Acc:19288]	chr2	135596117	135659604	428	0.158	1	1	1.121E-02	2.715E-02	PB-GT
MATR3	matrin 3 [Source:HGNC Symbol;Acc:6912]	chr5	138609441	138667360	484	0.158	1	1	1.125E-02	2.720E-02	PB-GT
DNAJB6	DnaJ (Hsp40) homolog, subfamily B, member 6 [Source:HGNC Symbol;Acc:14888]	chr7	157128075	157210133	473	0.158	1	1	1.125E-02	2.720E-02	PB-GT
RAD23B	RAD23 homolog B (S. cerevisiae) [Source:HGNC Symbol;Acc:9813]	chr9	110045418	110094475	484	0.159	1	1	1.133E-02	2.735E-02	PB-GT
LYPLAL1	lysophospholipase-like 1 [Source:HGNC Symbol;Acc:20440]	chr1	219347186	219386207	450	0.159	1	1	1.144E-02	2.757E-02	PB-GT
DHRSX	dehydrogenase/reductase (SDR family) X-linked [Source:HGNC Symbol;Acc:18399]	chrX	2137557	2420846	957	0.159	1	1	1.144E-02	2.757E-02	PB-GT
FAM114A1	family with sequence similarity 114, member A1 [Source:HGNC Symbol;Acc:25087]	chr4	38869298	38947360	488	0.160	1	1	1.149E-02	2.768E-02	PB-GT
ZNF365	zinc finger protein 365 [Source:HGNC Symbol;Acc:18194]	chr10	64133951	64431771	1995	0.859	3	2	1.154E-02	2.775E-02	PB-GT
FAM193A	family with sequence similarity 193, member A [Source:HGNC Symbol;Acc:16822]	chr4	2626988	2734292	490	0.161	1	1	1.158E-02	2.784E-02	PB-GT
AHRR	aryl-hydrocarbon receptor repressor [Source:HGNC Symbol;Acc:346]	chr5	304291	438406	492	0.161	1	1	1.160E-02	2.786E-02	PB-GT
SYDE2	synapse defective 1, Rho GTPase, homolog 2 (C. elegans) [Source:HGNC Symbol;Acc:25841]	chr1	85622556	85666729	454	0.161	1	1	1.163E-02	2.790E-02	PB-GT
EIF4E	eukaryotic translation initiation factor 4E [Source:HGNC Symbol;Acc:3287]	chr4	99792835	99851788	492	0.161	1	1	1.167E-02	2.796E-02	PB-GT
THRAP3	thyroid hormone receptor associated protein 3 [Source:HGNC Symbol;Acc:22964]	chr1	36690017	36770958	455	0.161	1	1	1.168E-02	2.796E-02	PB-GT
CAPN13	calpain 13 [Source:HGNC Symbol;Acc:16663]	chr2	30945637	31043408	438	0.161	1	1	1.171E-02	2.802E-02	PB-GT
ANKRD50	ankyrin repeat domain 50 [Source:HGNC Symbol;Acc:29223]	chr4	125585207	125633887	494	0.162	1	1	1.176E-02	2.810E-02	PB-GT
ZFAT	zinc finger and AT hook domain containing [Source:HGNC Symbol;Acc:19899]	chr8	135490031	135725292	1185	0.463	2	2	1.177E-02	2.810E-02	PB-GT
TMX2-CTNND1	TMX2-CTNND1 readthrough (non-protein coding) [Source:HGNC Symbol;Acc:41992]	chr11	57480077	57559058	445	0.162	1	1	1.178E-02	2.811E-02	PB-GT
ACAP2	ArfGAP with coiled-coil, ankyrin repeat and PH domains 2 [Source:HGNC Symbol;Acc:16469]	chr3	194995465	195163807	1412	0.464	2	2	1.179E-02	2.811E-02	PB-GT
CHST8	carbohydrate (N-acetylgalactosamine 4-0) sulfotransferase 8 [Source:HGNC Symbol;Acc:15993]	chr19	34112861	34264413	476	0.164	1	1	1.201E-02	2.857E-02	PB-GT
DCLRE1CP1	DNA cross-link repair 1C pseudogene 1 [Source:HGNC Symbol;Acc:44877]	chr10	15057071	15130775	380	0.164	1	1	1.201E-02	2.857E-02	PB-GT
CCNG2	cyclin G2 [Source:HGNC Symbol;Acc:1593]	chr4	78078304	78354542	1426	0.467	2	1	1.202E-02	2.857E-02	PB-GT
TMC5	transmembrane channel-like 5 [Source:HGNC Symbol;Acc:22999]	chr16	19421818	19510435	380	0.164	1	1	1.204E-02	2.859E-02	PB-GT
SRSF11	serine/arginine-rich splicing factor 11 [Source:HGNC Symbol;Acc:10782]	chr1	70671365	70718735	465	0.165	1	1	1.217E-02	2.887E-02	PB-GT
WIPF2	WAS/WASL interacting protein family, member 2 [Source:HGNC Symbol;Acc:30923]	chr17	38375556	38440388	311	0.165	1	1	1.218E-02	2.887E-02	PB-GT
PSG8	pregnancy specific beta-1-glycoprotein 8 [Source:HGNC Symbol;Acc:9525]	chr19	43256838	43359843	481	0.165	1	1	1.225E-02	2.901E-02	PB-GT
SCARA5	scavenger receptor class A, member 5 (putative) [Source:HGNC Symbol;Acc:28701]	chr8	27727399	27850244	423	0.165	1	1	1.227E-02	2.902E-02	PB-GT
TUBGCP3	tubulin, gamma complex associated protein 3 [Source:HGNC Symbol;Acc:18598]	chr13	113139325	113242481	686	0.166	1	1	1.229E-02	2.906E-02	PB-GT
PTPN1	protein tyrosine phosphatase, non-receptor type 1 [Source:HGNC Symbol;Acc:9642]	chr20	49126891	49201299	446	0.166	1	1	1.231E-02	2.908E-02	PB-GT
LUC7L3	LUC7-like 3 (S. cerevisiae) [Source:HGNC Symbol;Acc:24309]	chr17	48796905	48833574	313	0.166	1	1	1.233E-02	2.908E-02	PB-GT
A2MP1	alpha-2-macroglobulin pseudogene 1 [Source:HGNC Symbol;Acc:8]	chr12	9381129	9428413	376	0.166	1	1	1.234E-02	2.908E-02	PB-GT
TMEM196	transmembrane protein 196 [Source:HGNC Symbol;Acc:22431]	chr7	19758933	19813221	497	0.166	1	1	1.236E-02	2.911E-02	PB-GT
PDCD10	programmed cell death 10 [Source:HGNC Symbol;Acc:8761]	chr3	167401086	167452727	506	0.166	1	1	1.237E-02	2.912E-02	PB-GT
GPSM2	G-protein signaling modulator 2 [Source:HGNC Symbol;Acc:29501]	chr1	109417972	109473044	470	0.167	1	1	1.242E-02	2.920E-02	PB-GT
CRYL1	crystallin, lambda 1 [Source:HGNC Symbol;Acc:18246]	chr13	20977806	21099996	692	0.167	1	1	1.250E-02	2.934E-02	PB-GT
HERPUD2	HERPUD family member 2 [Source:HGNC Symbol;Acc:21915]	chr7	35672269	35735181	500	0.167	1	1	1.250E-02	2.934E-02	PB-GT
CTNNB1	catenin (cadherin-associated protein), beta 1, 88kDa [Source:HGNC Symbol;Acc:2514]	chr3	41236328	41301587	509	0.167	1	1	1.251E-02	2.934E-02	PB-GT
KBTBD12	kelch repeat and BTB (POZ) domain containing 12 [Source:HGNC Symbol;Acc:25731]	chr3	127634075	127706514	510	0.168	1	1	1.256E-02	2.942E-02	PB-GT
PAQR3	progestin and adipoQ receptor family member III [Source:HGNC Symbol;Acc:30130]	chr4	79808281	79860592	512	0.168	1	1	1.259E-02	2.946E-02	PB-GT
MPZL1	myelin protein zero-like 1 [Source:HGNC Symbol;Acc:7226]	chr1	167690429	167761156	476	0.169	1	1	1.272E-02	2.974E-02	PB-GT

table S1. Genes with significant transposon integrations in DLD-1 *KRAS*$^{wt/-}$ cells after selection in low glucose medium.

Gene ID	Description	Chrom	Start	End	# TTAA sites	Expected	Observed	Libraries	*P*	FDR	Transposon
P2RY14	purinergic receptor P2Y, G-protein coupled, 14 [Source:HGNC Symbol;Acc:16442]	chr3	150929905	150996255	517	0.170	1	1	1.289E-02	3.008E-02	PB-GT
ADCY5	adenylate cyclase 5 [Source:HGNC Symbol;Acc:236]	chr3	123001143	123168605	517	0.170	1	1	1.289E-02	3.008E-02	PB-GT
ZNF519	zinc finger protein 519 [Source:HGNC Symbol;Acc:30574]	chr18	14057456	14132489	532	0.170	1	1	1.294E-02	3.019E-02	PB-GT
PXDN	peroxidasin homolog (Drosophila) [Source:HGNC Symbol;Acc:14966]	chr2	1635659	1748624	462	0.170	1	1	1.296E-02	3.019E-02	PB-GT
SLTM	SAFB-like, transcription modulator [Source:HGNC Symbol;Acc:20709]	chr15	59171244	59225852	464	0.171	1	1	1.300E-02	3.026E-02	PB-GT
EXOC6B	exocyst complex component 6B [Source:HGNC Symbol;Acc:17085]	chr2	72403113	73053170	5139	1.894	5	2	1.305E-02	3.033E-02	PB-GT
ASB5	ankyrin repeat and SOCS box containing 5 [Source:HGNC Symbol;Acc:17180]	chr4	177134824	177198722	522	0.171	1	1	1.306E-02	3.033E-02	PB-GT
CCND3	cyclin D3 [Source:HGNC Symbol;Acc:1585]	chr6	41902671	42018095	484	0.171	1	1	1.307E-02	3.033E-02	PB-GT
CCDC30	coiled-coil domain containing 30 [Source:HGNC Symbol;Acc:26103]	chr1	42929001	43120335	1361	0.482	2	1	1.307E-02	3.033E-02	PB-GT
C7orf49	chromosome 7 open reading frame 49 [Source:HGNC Symbol;Acc:22432]	chr7	134777115	134855547	514	0.172	1	1	1.317E-02	3.052E-02	PB-GT
ARHGAP12	Rho GTPase activating protein 12 [Source:HGNC Symbol;Acc:16348]	chr10	32094365	32217742	1124	0.484	2	2	1.321E-02	3.058E-02	PB-GT
FAM208A	family with sequence similarity 208, member A [Source:HGNC Symbol;Acc:30314]	chr3	56654161	56717265	524	0.172	1	1	1.322E-02	3.058E-02	PB-GT
MMS22L	MMS22-like, DNA repair protein [Source:HGNC Symbol;Acc:21475]	chr6	97590037	97731093	1372	0.485	2	2	1.327E-02	3.065E-02	PB-GT
YTHDF3	YTH domain family, member 3 [Source:HGNC Symbol;Acc:26465]	chr8	64081112	64125346	441	0.172	1	1	1.327E-02	3.065E-02	PB-GT
VDR	vitamin D (1,25- dihydroxyvitamin D3) receptor [Source:HGNC Symbol;Acc:12679]	chr12	48235320	48336831	392	0.173	1	1	1.335E-02	3.080E-02	PB-GT
SMAP2	small ArfGAP2 [Source:HGNC Symbol;Acc:25082]	chr1	40810522	40888998	490	0.174	1	1	1.343E-02	3.097E-02	PB-GT
FBXO38	F-box protein 38 [Source:HGNC Symbol;Acc:28844]	chr5	147763498	147822399	532	0.174	1	1	1.345E-02	3.098E-02	PB-GT
EBNA1BP2	EBNA1 binding protein 2 [Source:HGNC Symbol;Acc:15531]	chr1	43629846	43736607	491	0.174	1	1	1.349E-02	3.101E-02	PB-GT
DHX35	DEAH (Asp-Glu-Ala-His) box polypeptide 35 [Source:HGNC Symbol;Acc:15861]	chr20	37590942	37668366	468	0.174	1	1	1.349E-02	3.101E-02	PB-GT
ANKRD42	ankyrin repeat domain 42 [Source:HGNC Symbol;Acc:26752]	chr11	82904781	82971736	479	0.174	1	1	1.354E-02	3.110E-02	PB-GT
METAP1	methionyl aminopeptidase 1 [Source:HGNC Symbol;Acc:15789]	chr4	99916771	99983964	534	0.175	1	1	1.363E-02	3.128E-02	PB-GT
EFCAB7	EF-hand calcium binding domain 7 [Source:HGNC Symbol;Acc:29379]	chr1	63989043	64038364	494	0.175	1	1	1.364E-02	3.128E-02	PB-GT
HNRNPC	heterogeneous nuclear ribonucleoprotein C (C1/C2) [Source:HGNC Symbol;Acc:5035]	chr14	21677295	21737653	488	0.175	1	1	1.365E-02	3.128E-02	PB-GT
CHIC2	cysteine-rich hydrophobic domain 2 [Source:HGNC Symbol;Acc:1935]	chr4	54875956	54930857	537	0.176	1	1	1.377E-02	3.153E-02	PB-GT
WDR47	WD repeat domain 47 [Source:HGNC Symbol;Acc:29141]	chr1	109512836	109584850	497	0.176	1	1	1.380E-02	3.156E-02	PB-GT
ATP8B5P	ATPase, class I, type 8B, member 5, pseudogene [Source:HGNC Symbol;Acc:27245]	chr9	35406752	35483026	538	0.176	1	1	1.383E-02	3.156E-02	PB-GT
SPTLC1	serine palmitoyltransferase, long chain base subunit 1 [Source:HGNC Symbol;Acc:11277]	chr9	94794281	94877666	538	0.176	1	1	1.383E-02	3.156E-02	PB-GT
IARS	isoleucyl-tRNA synthetase [Source:HGNC Symbol;Acc:5330]	chr9	94972489	95056038	538	0.176	1	1	1.383E-02	3.156E-02	PB-GT
UBE2U	ubiquitin-conjugating enzyme E2U (putative) [Source:HGNC Symbol;Acc:28559]	chr1	64669310	64733053	498	0.176	1	1	1.385E-02	3.157E-02	PB-GT
TOPBP1	topoisomerase (DNA) II binding protein 1 [Source:HGNC Symbol;Acc:17008]	chr3	133317019	133380737	538	0.177	1	1	1.389E-02	3.163E-02	PB-GT
STMN2	stathmin-like 2 [Source:HGNC Symbol;Acc:10577]	chr8	80523049	80578410	452	0.177	1	1	1.390E-02	3.163E-02	PB-GT
SGPL1	sphingosine-1-phosphate lyase 1 [Source:HGNC Symbol;Acc:10817]	chr10	72575717	72640930	411	0.177	1	1	1.393E-02	3.167E-02	PB-GT
CREBRF	CREB3 regulatory factor [Source:HGNC Symbol;Acc:24050]	chr5	172483355	172566291	543	0.177	1	1	1.398E-02	3.175E-02	PB-GT
SOSTDC1	sclerostin domain containing 1 [Source:HGNC Symbol;Acc:21748]	chr7	16501106	16570205	531	0.177	1	1	1.400E-02	3.176E-02	PB-GT
GON4L	gon-4-like (C. elegans) [Source:HGNC Symbol;Acc:25973]	chr1	155719508	155829191	501	0.177	1	1	1.401E-02	3.176E-02	PB-GT
BOLL	bol, boule-like (Drosophila) [Source:HGNC Symbol;Acc:14273]	chr2	198591603	198651486	482	0.178	1	1	1.403E-02	3.179E-02	PB-GT
HUS1	HUS1 checkpoint homolog (S. pombe) [Source:HGNC Symbol;Acc:5309]	chr7	47735328	48019178	1483	0.496	2	2	1.406E-02	3.182E-02	PB-GT
CD2AP	CD2-associated protein [Source:HGNC Symbol;Acc:14258]	chr6	47445525	47594999	1403	0.496	2	1	1.408E-02	3.183E-02	PB-GT
PCOLCE2	procollagen C-endopeptidase enhancer 2 [Source:HGNC Symbol;Acc:8739]	chr3	142534764	142608045	543	0.178	1	1	1.413E-02	3.194E-02	PB-GT
GSN	gelsolin [Source:HGNC Symbol;Acc:4620]	chr9	123970078	124095121	545	0.179	1	1	1.417E-02	3.199E-02	PB-GT
APLP2	amyloid beta (A4) precursor-like protein 2 [Source:HGNC Symbol;Acc:598]	chr11	129939732	130014699	491	0.179	1	1	1.418E-02	3.199E-02	PB-GT
MAML2	mastermind-like 2 (Drosophila) [Source:HGNC Symbol;Acc:16259]	chr11	95709762	96076382	2514	0.915	3	1	1.421E-02	3.202E-02	PB-GT
PLCE1	phospholipase C, epsilon 1 [Source:HGNC Symbol;Acc:17175]	chr10	95753746	96088149	2126	0.916	3	2	1.424E-02	3.203E-02	PB-GT
TMEM220-AS1	TMEM220 antisense RNA 1 [Source:HGNC Symbol;Acc:44357]	chr17	10633113	10718481	338	0.179	1	1	1.425E-02	3.203E-02	PB-GT
BZW2	basic leucine zipper and W2 domains 2 [Source:HGNC Symbol;Acc:18808]	chr7	16685756	16746148	536	0.179	1	1	1.425E-02	3.203E-02	PB-GT
TXNRD3	thioredoxin reductase 3 [Source:HGNC Symbol;Acc:20667]	chr3	126290622	126373998	546	0.179	1	1	1.428E-02	3.208E-02	PB-GT
C3orf70	chromosome 3 open reading frame 70 [Source:HGNC Symbol;Acc:33731]	chr3	184795838	184870802	547	0.180	1	1	1.433E-02	3.215E-02	PB-GT
HMG20A	high mobility group 20A [Source:HGNC Symbol;Acc:5001]	chr15	77712754	77777949	489	0.180	1	1	1.435E-02	3.215E-02	PB-GT
G3BP2	GTPase activating protein (SH3 domain) binding protein 2 [Source:HGNC Symbol;Acc:30291]	chr4	76567966	76649709	549	0.180	1	1	1.436E-02	3.215E-02	PB-GT
TNFRSF21	tumor necrosis factor receptor superfamily, member 21 [Source:HGNC Symbol;Acc:13469]	chr6	47199268	47277641	509	0.180	1	1	1.437E-02	3.215E-02	PB-GT
ARHGAP21	Rho GTPase activating protein 21 [Source:HGNC Symbol;Acc:23725]	chr10	24872538	25012597	1161	0.500	2	1	1.439E-02	3.217E-02	PB-GT
KIAA1324L	KIAA1324-like [Source:HGNC Symbol;Acc:21945]	chr7	86506222	86689015	1497	0.500	2	2	1.441E-02	3.217E-02	PB-GT
MYO1E	myosin IE [Source:HGNC Symbol;Acc:7599]	chr15	59427113	59665099	1361	0.500	2	1	1.441E-02	3.217E-02	PB-GT
FYN	FYN oncogene related to SRC, FGR, YES [Source:HGNC Symbol;Acc:4037]	chr6	111981535	112194655	1416	0.500	2	1	1.442E-02	3.217E-02	PB-GT
AGPAT5	1-acylglycerol-3-phosphate O-acyltransferase 5 [Source:HGNC Symbol;Acc:20886]	chr8	6565878	6617184	462	0.181	1	1	1.449E-02	3.228E-02	PB-GT
ERO1L	ERO1-like (S. cerevisiae) [Source:HGNC Symbol;Acc:13280]	chr14	53106634	53162618	504	0.181	1	1	1.451E-02	3.229E-02	PB-GT
GLRX	glutaredoxin (thioltransferase) [Source:HGNC Symbol;Acc:4330]	chr5	95087023	95158709	554	0.181	1	1	1.452E-02	3.229E-02	PB-GT
FAM184A	family with sequence similarity 184, member A [Source:HGNC Symbol;Acc:20991]	chr6	119280928	119470552	1420	0.502	2	2	1.453E-02	3.230E-02	PB-GT
ZBTB40	zinc finger and BTB domain containing 40 [Source:HGNC Symbol;Acc:29045]	chr1	22778344	22857650	512	0.181	1	1	1.459E-02	3.241E-02	PB-GT
NT5DC3	5'-nucleotidase domain containing 3 [Source:HGNC Symbol;Acc:30826]	chr12	104164231	104234975	412	0.182	1	1	1.466E-02	3.252E-02	PB-GT
LEF1-AS1	LEF1 antisense RNA 1 [Source:HGNC Symbol;Acc:40339]	chr4	109088681	109177992	556	0.182	1	1	1.470E-02	3.259E-02	PB-GT
UBE2D2	ubiquitin-conjugating enzyme E2D 2 [Source:HGNC Symbol;Acc:12475]	chr5	138906016	139008018	558	0.182	1	1	1.471E-02	3.259E-02	PB-GT
IQCB1	IQ motif containing B1 [Source:HGNC Symbol;Acc:28949]	chr3	121488610	121553926	557	0.183	1	1	1.483E-02	3.277E-02	PB-GT
PTDSS1	phosphatidylserine synthase 1 [Source:HGNC Symbol;Acc:9587]	chr8	97273943	97349223	468	0.183	1	1	1.484E-02	3.277E-02	PB-GT
BDNF	brain-derived neurotrophic factor [Source:HGNC Symbol;Acc:1033]	chr11	27676440	27743605	503	0.183	1	1	1.484E-02	3.277E-02	PB-GT
STX3	syntaxin 3 [Source:HGNC Symbol;Acc:11438]	chr11	59480929	59573354	503	0.183	1	1	1.484E-02	3.277E-02	PB-GT
C22orf39	chromosome 22 open reading frame 39 [Source:HGNC Symbol;Acc:27012]	chr22	19338891	19435755	434	0.183	1	1	1.487E-02	3.280E-02	PB-GT
PLAC1	placenta-specific 1 [Source:HGNC Symbol;Acc:9044]	chrX	133699868	133898352	1101	0.183	1	1	1.490E-02	3.282E-02	PB-GT
AFAP1L2	actin filament associated protein 1-like 2 [Source:HGNC Symbol;Acc:25901]	chr10	116054583	116164515	426	0.183	1	1	1.490E-02	3.282E-02	PB-GT
TNS1	tensin 1 [Source:HGNC Symbol;Acc:11973]	chr2	218664512	218867718	498	0.184	1	1	1.492E-02	3.284E-02	PB-GT
COL22A1	collagen, type XXII, alpha 1 [Source:HGNC Symbol;Acc:22989]	chr8	139600478	139926249	1299	0.508	2	2	1.501E-02	3.299E-02	PB-GT
PELI1	pellino E3 ubiquitin protein ligase 1 [Source:HGNC Symbol;Acc:8827]	chr2	64319786	64371588	500	0.184	1	1	1.504E-02	3.300E-02	PB-GT
COL13A1	collagen, type XIII, alpha 1 [Source:HGNC Symbol;Acc:2190]	chr10	71561644	71724031	428	0.184	1	1	1.504E-02	3.300E-02	PB-GT
FAM177A1	family with sequence similarity 177, member A1 [Source:HGNC Symbol;Acc:19829]	chr14	35514113	35582336	514	0.184	1	1	1.505E-02	3.301E-02	PB-GT
KTN1-AS1	KTN1 antisense RNA 1 [Source:HGNC Symbol;Acc:19842]	chr14	55965996	56046828	515	0.185	1	1	1.511E-02	3.310E-02	PB-GT
CYSTM1	cysteine-rich transmembrane module containing 1 [Source:HGNC Symbol;Acc:30239]	chr5	139554227	139661637	568	0.185	1	1	1.521E-02	3.330E-02	PB-GT
C12orf55	chromosome 12 open reading frame 55 [Source:HGNC Symbol;Acc:26456]	chr12	96883349	97040905	1158	0.511	2	2	1.524E-02	3.333E-02	PB-GT
C12orf45	chromosome 12 open reading frame 45 [Source:HGNC Symbol;Acc:28628]	chr12	105380088	105443515	423	0.187	1	1	1.540E-02	3.366E-02	PB-GT
ABLIM3	actin binding LIM protein family, member 3 [Source:HGNC Symbol;Acc:29132]	chr5	148521046	148640105	574	0.187	1	1	1.552E-02	3.388E-02	PB-GT
N4BP2L2	NEDD4 binding protein 2-like 2 [Source:HGNC Symbol;Acc:26916]	chr13	33006554	33112970	779	0.188	1	1	1.562E-02	3.406E-02	PB-GT
GLDC	glycine dehydrogenase (decarboxylating) [Source:HGNC Symbol;Acc:4313]	chr9	6532464	6645650	574	0.188	1	1	1.562E-02	3.406E-02	PB-GT
GLT8D2	glycosyltransferase 8 domain containing 2 [Source:HGNC Symbol;Acc:24890]	chr12	104382762	104457961	427	0.188	1	1	1.568E-02	3.414E-02	PB-GT
SPTB	spectrin, beta, erythrocytic [Source:HGNC Symbol;Acc:11274]	chr14	65213002	65346601	527	0.189	1	1	1.578E-02	3.428E-02	PB-GT
INMT-FAM188B	INMT-FAM188B readthrough (non-protein coding) [Source:HGNC Symbol;Acc:41995]	chr7	30791753	30931696	566	0.189	1	1	1.579E-02	3.428E-02	PB-GT
CPB1	carboxypeptidase B1 (tissue) [Source:HGNC Symbol;Acc:2299]	chr3	148508889	148577974	576	0.189	1	1	1.579E-02	3.428E-02	PB-GT
TSC22D2	TSC22 domain family, member 2 [Source:HGNC Symbol;Acc:29095]	chr3	150126122	150177903	576	0.189	1	1	1.579E-02	3.428E-02	PB-GT
CAMK2D	calcium/calmodulin-dependent protein kinase II delta [Source:HGNC Symbol;Acc:1462]	chr4	114372188	114683083	2885	0.945	3	2	1.580E-02	3.428E-02	PB-GT
LEPROTL1	leptin receptor overlapping transcript-like 1 [Source:HGNC Symbol;Acc:6555]	chr8	29952914	30034724	485	0.190	1	1	1.587E-02	3.440E-02	PB-GT
RPS6KA5	ribosomal protein S6 kinase, 90kDa, polypeptide 5 [Source:HGNC Symbol;Acc:10434]	chr14	91336799	91526980	1448	0.520	2	1	1.591E-02	3.443E-02	PB-GT
PRDM11	PR domain containing 11 [Source:HGNC Symbol;Acc:13996]	chr11	45115564	45247734	522	0.190	1	1	1.591E-02	3.443E-02	PB-GT
PLD1	phospholipase D1, phosphatidylcholine-specific [Source:HGNC Symbol;Acc:9067]	chr3	171318195	171528740	1583	0.520	2	1	1.595E-02	3.446E-02	PB-GT
JAM3	junctional adhesion molecule 3 [Source:HGNC Symbol;Acc:15532]	chr11	133938820	134021896	523	0.190	1	1	1.597E-02	3.446E-02	PB-GT
RAB11FIP2	RAB11 family interacting protein 2 (class I) [Source:HGNC Symbol;Acc:29152]	chr10	119764427	119806114	442	0.190	1	1	1.597E-02	3.446E-02	PB-GT
PPP4R1	protein phosphatase 4, regulatory subunit 1 [Source:HGNC Symbol;Acc:9320]	chr18	9546789	9615238	595	0.190	1	1	1.598E-02	3.446E-02	PB-GT
RTN3	reticulon 3 [Source:HGNC Symbol;Acc:10469]	chr11	63448918	63527363	524	0.191	1	1	1.603E-02	3.454E-02	PB-GT
SLC6A11	solute carrier family 6 (neurotransmitter transporter, GABA), member 11 [Source:HGNC Symbol;Acc:11044]	chr3	10857885	10982419	581	0.191	1	1	1.605E-02	3.454E-02	PB-GT
WNK3	WNK lysine deficient protein kinase 3 [Source:HGNC Symbol;Acc:14543]	chrX	54219256	54385075	1146	0.191	1	1	1.607E-02	3.454E-02	PB-GT
DPYSL5	dihydropyrimidinase-like 5 [Source:HGNC Symbol;Acc:20637]	chr2	27070615	27173219	518	0.191	1	1	1.607E-02	3.454E-02	PB-GT
SH3D19	SH3 domain containing 19 [Source:HGNC Symbol;Acc:30418]	chr4	152023903	152246784	1593	0.522	2	2	1.610E-02	3.457E-02	PB-GT
HIF1A	hypoxia inducible factor 1, alpha subunit (basic helix-loop-helix transcription factor) [Source:HGNC Symbol;Acc:4910]	chr14	62162231	62214976	533	0.191	1	1	1.611E-02	3.458E-02	PB-GT
PPM1A	protein phosphatase, Mg2+/Mn2+ dependent, 1A [Source:HGNC Symbol;Acc:9275]	chr14	60712470	60765805	534	0.192	1	1	1.617E-02	3.466E-02	PB-GT
CXCL13	chemokine (C-X-C motif) ligand 13 [Source:HGNC Symbol;Acc:10639]	chr4	78432907	78532988	585	0.192	1	1	1.618E-02	3.466E-02	PB-GT
FAM47E	family with sequence similarity 47, member E [Source:HGNC Symbol;Acc:34343]	chr4	77135193	77232282	586	0.192	1	1	1.623E-02	3.471E-02	PB-GT
TBK1	TANK-binding kinase 1 [Source:HGNC Symbol;Acc:11584]	chr12	64845660	64895888	435	0.192	1	1	1.623E-02	3.471E-02	PB-GT
MARCH7	membrane-associated ring finger (C3HC4) 7, E3 ubiquitin protein ligase [Source:HGNC Symbol;Acc:17393]	chr2	160569000	160625359	521	0.192	1	1	1.624E-02	3.471E-02	PB-GT
SBF2	SET binding factor 2 [Source:HGNC Symbol;Acc:2135]	chr11	9800214	10315754	3982	1.449	4	2	1.628E-02	3.477E-02	PB-GT
GNA12	guanine nucleotide binding protein (G protein) alpha 12 [Source:HGNC Symbol;Acc:4380]	chr7	2767746	2883958	576	0.193	1	1	1.631E-02	3.481E-02	PB-GT
MAX	MYC associated factor X [Source:HGNC Symbol;Acc:6913]	chr14	65472892	65569413	537	0.193	1	1	1.634E-02	3.483E-02	PB-GT
F5	coagulation factor V (proaccelerin, labile factor) [Source:HGNC Symbol;Acc:3542]	chr1	169483404	169555826	544	0.193	1	1	1.635E-02	3.483E-02	PB-GT
STEAP2	STEAP family member 2, metalloreductase [Source:HGNC Symbol;Acc:17885]	chr7	89796904	89867451	577	0.193	1	1	1.637E-02	3.484E-02	PB-GT
FBXO42	F-box protein 42 [Source:HGNC Symbol;Acc:29249]	chr1	16573334	16678949	545	0.193	1	1	1.641E-02	3.489E-02	PB-GT
AKR1C3	aldo-keto reductase family 1, member C3 [Source:HGNC Symbol;Acc:386]	chr10	5077546	5149878	449	0.193	1	1	1.645E-02	3.495E-02	PB-GT
VKORC1L1	vitamin K epoxide reductase complex, subunit 1-like 1 [Source:HGNC Symbol;Acc:21492]	chr7	65338254	65424550	579	0.194	1	1	1.647E-02	3.498E-02	PB-GT
AKAP9	A kinase (PRKA) anchor protein (yotiao) 9 [Source:HGNC Symbol;Acc:379]	chr7	91570181	91739987	1577	0.527	2	2	1.652E-02	3.505E-02	PB-GT
STX17	syntaxin 17 [Source:HGNC Symbol;Acc:11432]	chr9	102668915	102732618	592	0.194	1	1	1.655E-02	3.506E-02	PB-GT
SOD2	superoxide dismutase 2, mitochondrial [Source:HGNC Symbol;Acc:11180]	chr6	160100096	160183561	549	0.194	1	1	1.656E-02	3.506E-02	PB-GT
F8	coagulation factor VIII, procoagulant component [Source:HGNC Symbol;Acc:3546]	chrX	154064063	154255215	1165	0.194	1	1	1.657E-02	3.506E-02	PB-GT
NCOR2	nuclear receptor corepressor 2 [Source:HGNC Symbol;Acc:7673]	chr12	124808961	125052135	440	0.194	1	1	1.658E-02	3.506E-02	PB-GT
DHRS7B	dehydrogenase/reductase (SDR family) member 7B [Source:HGNC Symbol;Acc:24547]	chr17	21026677	21096578	367	0.194	1	1	1.663E-02	3.509E-02	PB-GT
LPAR3	lysophosphatidic acid receptor 3 [Source:HGNC Symbol;Acc:14298]	chr1	85277285	85358896	549	0.195	1	1	1.663E-02	3.509E-02	PB-GT
SMIM12	small integral membrane protein 12 [Source:HGNC Symbol;Acc:25154]	chr1	35178338	35325417	549	0.195	1	1	1.663E-02	3.509E-02	PB-GT
FAM178A	family with sequence similarity 178, member A [Source:HGNC Symbol;Acc:17814]	chr10	102672720	102724893	452	0.195	1	1	1.666E-02	3.511E-02	PB-GT
RASEF	RAS and EF-hand domain containing [Source:HGNC Symbol;Acc:26464]	chr9	85594500	85678092	595	0.195	1	1	1.671E-02	3.520E-02	PB-GT
EPT1	ethanolaminephosphotransferase 1 (CDP-ethanolamine-specific) [Source:HGNC Symbol;Acc:29361]	chr2	26531415	26618759	530	0.195	1	1	1.677E-02	3.529E-02	PB-GT
NAALAD2	N-acetylated alpha-linked acidic dipeptidase 2 [Source:HGNC Symbol;Acc:14526]	chr11	89864683	89926062	538	0.196	1	1	1.684E-02	3.541E-02	PB-GT
DIRC1	disrupted in renal carcinoma 1 [Source:HGNC Symbol;Acc:15760]	chr2	189598882	189654831	532	0.196	1	1	1.689E-02	3.549E-02	PB-GT
HSPA4L	heat shock 70kDa protein 4-like [Source:HGNC Symbol;Acc:17041]	chr4	128702976	128755226	599	0.196	1	1	1.691E-02	3.550E-02	PB-GT
BEND6	BEN domain containing 6 [Source:HGNC Symbol;Acc:20871]	chr6	56819773	56892140	557	0.197	1	1	1.701E-02	3.568E-02	PB-GT
KLHL23	kelch-like family member 23 [Source:HGNC Symbol;Acc:27506]	chr2	170550998	170633499	535	0.197	1	1	1.707E-02	3.577E-02	PB-GT
KIAA0247	KIAA0247 [Source:HGNC Symbol;Acc:19956]	chr14	70078313	70181859	550	0.197	1	1	1.709E-02	3.579E-02	PB-GT
ADCY1	adenylate cyclase 1 (brain) [Source:HGNC Symbol;Acc:232]	chr7	45613739	45762715	591	0.198	1	1	1.712E-02	3.582E-02	PB-GT
ALDH1L2	aldehyde dehydrogenase 1 family, member L2 [Source:HGNC Symbol;Acc:26777]	chr12	105413568	105478355	448	0.198	1	1	1.715E-02	3.584E-02	PB-GT
PTCHD4	patched domain containing 4 [Source:HGNC Symbol;Acc:21345]	chr6	47845764	48036425	1513	0.535	2	2	1.716E-02	3.584E-02	PB-GT
ARHGAP32	Rho GTPase activating protein 32 [Source:HGNC Symbol;Acc:17399]	chr11	128834955	129149219	2664	0.969	3	2	1.717E-02	3.584E-02	PB-GT
RFX6	regulatory factor X, 6 [Source:HGNC Symbol;Acc:21478]	chr6	117198375	117253326	560	0.198	1	1	1.718E-02	3.584E-02	PB-GT
RND3	Rho family GTPase 3 [Source:HGNC Symbol;Acc:671]	chr2	151324709	151395525	538	0.198	1	1	1.725E-02	3.592E-02	PB-GT
SPC25	SPC25, NDC80 kinetochore complex component, homolog (S. cerevisiae) [Source:HGNC Symbol;Acc:24031]	chr2	169690642	169769881	538	0.198	1	1	1.725E-02	3.592E-02	PB-GT
B4GALT5	UDP-Gal:betaGlcNAc beta 1,4- galactosyltransferase, polypeptide 5 [Source:HGNC Symbol;Acc:928]	chr20	48249482	48330415	534	0.198	1	1	1.728E-02	3.595E-02	PB-GT
ELK3	ELK3, ETS-domain protein (SRF accessory protein 2) [Source:HGNC Symbol;Acc:3325]	chr12	96588160	96663613	450	0.199	1	1	1.729E-02	3.595E-02	PB-GT
GLRA1	glycine receptor, alpha 1 [Source:HGNC Symbol;Acc:4326]	chr5	151202074	151304403	609	0.199	1	1	1.734E-02	3.601E-02	PB-GT
ENO4	enolase family member 4 [Source:HGNC Symbol;Acc:31670]	chr10	118609023	118671299	463	0.199	1	1	1.742E-02	3.615E-02	PB-GT
SGOL2	shugoshin-like 2 (S. pombe) [Source:HGNC Symbol;Acc:30812]	chr2	201374731	201448505	541	0.199	1	1	1.743E-02	3.615E-02	PB-GT
PDE4DIP	phosphodiesterase 4D interacting protein [Source:HGNC Symbol;Acc:15580]	chr1	144836157	145076186	1519	0.538	2	2	1.745E-02	3.616E-02	PB-GT
UBR5	ubiquitin protein ligase E3 component n-recognin 5 [Source:HGNC Symbol;Acc:16806]	chr8	103265240	103425069	1378	0.539	2	2	1.751E-02	3.627E-02	PB-GT
LUZP1	leucine zipper protein 1 [Source:HGNC Symbol;Acc:14985]	chr1	23410516	23504301	565	0.200	1	1	1.755E-02	3.632E-02	PB-GT
TGFBR2	transforming growth factor, beta receptor II (70/80kDa) [Source:HGNC Symbol;Acc:11773]	chr3	30647994	30735634	610	0.200	1	1	1.758E-02	3.632E-02	PB-GT
ZNF106	zinc finger protein 106 [Source:HGNC Symbol;Acc:12886]	chr15	42705021	42783321	545	0.200	1	1	1.758E-02	3.632E-02	PB-GT
CCR3	chemokine (C-C motif) receptor 3 [Source:HGNC Symbol;Acc:1604]	chr3	46205096	46308197	611	0.201	1	1	1.764E-02	3.641E-02	PB-GT
EXPH5	exophilin 5 [Source:HGNC Symbol;Acc:30578]	chr11	108376158	108464465	553	0.201	1	1	1.773E-02	3.657E-02	PB-GT
DNAJC24	DnaJ (Hsp40) homolog, subfamily C, member 24 [Source:HGNC Symbol;Acc:26979]	chr11	31391387	31453396	555	0.202	1	1	1.785E-02	3.676E-02	PB-GT
ARNTL	aryl hydrocarbon receptor nuclear translocator-like [Source:HGNC Symbol;Acc:701]	chr11	13298199	13408813	555	0.202	1	1	1.785E-02	3.676E-02	PB-GT
NF2	neurofibromin 2 (merlin) [Source:HGNC Symbol;Acc:7773]	chr22	29999545	30094587	479	0.202	1	1	1.789E-02	3.681E-02	PB-GT
DNAJC9	DnaJ (Hsp40) homolog, subfamily C, member 9 [Source:HGNC Symbol;Acc:19123]	chr10	74943120	75008620	471	0.203	1	1	1.799E-02	3.695E-02	PB-GT
SAMSN1	SAM domain, SH3 domain and nuclear localization signals 1 [Source:HGNC Symbol;Acc:10528]	chr21	15857549	15955723	802	0.203	1	1	1.800E-02	3.695E-02	PB-GT
MAN1A1	mannosidase, alpha, class 1A, member 1 [Source:HGNC Symbol;Acc:6821]	chr6	119498374	119670926	1541	0.545	2	1	1.800E-02	3.695E-02	PB-GT
MDM1	Mdm1 nuclear protein homolog (mouse) [Source:HGNC Symbol;Acc:29917]	chr12	68666223	68726161	460	0.203	1	1	1.802E-02	3.696E-02	PB-GT
LINC00486	long intergenic non-protein coding RNA 486 [Source:HGNC Symbol;Acc:42946]	chr2	33050510	33151760	551	0.203	1	1	1.804E-02	3.697E-02	PB-GT
PAK2	p21 protein (Cdc42/Rac)-activated kinase 2 [Source:HGNC Symbol;Acc:8591]	chr3	196466728	196559518	619	0.203	1	1	1.807E-02	3.701E-02	PB-GT
NR2F2-AS1	NR2F2 antisense RNA 1 [Source:HGNC Symbol;Acc:44222]	chr15	96670598	96870590	1485	0.546	2	1	1.811E-02	3.706E-02	PB-GT

table S1. Genes with significant transposon integrations in DLD-1 *KRAS*$^{wt/-}$ cells after selection in low glucose medium.

Gene ID	Description	Chrom	Start	End	# TTAA sites	Expected	Observed	Libraries	*P*	FDR	Transposon
KIAA1731	KIAA1731 [Source:HGNC Symbol;Acc:29366]	chr11	93394805	93463522	560	0.204	1	1	1.815E-02	3.711E-02	PB-GT
ABCA4	ATP-binding cassette, sub-family A (ABC1), member 4 [Source:HGNC Symbol;Acc:34]	chr1	94458393	94586688	577	0.204	1	1	1.825E-02	3.728E-02	PB-GT
MALT1	mucosa associated lymphoid tissue lymphoma translocation gene 1 [Source:HGNC Symbol;Acc:6819]	chr18	56338618	56417371	639	0.204	1	1	1.826E-02	3.728E-02	PB-GT
SIDT1	SID1 transmembrane family, member 1 [Source:HGNC Symbol;Acc:25967]	chr3	113251143	113348425	625	0.205	1	1	1.840E-02	3.753E-02	PB-GT
KIF13A	kinesin family member 13A [Source:HGNC Symbol;Acc:14566]	chr6	17759414	17987854	1555	0.550	2	1	1.843E-02	3.756E-02	PB-GT
CCDC53	coiled-coil domain containing 53 [Source:HGNC Symbol;Acc:24256]	chr12	102406705	102455927	466	0.206	1	1	1.846E-02	3.760E-02	PB-GT
PJA2	praja ring finger 2, E3 ubiquitin protein ligase [Source:HGNC Symbol;Acc:17481]	chr5	108670410	108745695	632	0.206	1	1	1.858E-02	3.780E-02	PB-GT
HUNK	hormonally up-regulated Neu-associated kinase [Source:HGNC Symbol;Acc:13326]	chr21	33245628	33416946	816	0.206	1	1	1.859E-02	3.780E-02	PB-GT
DNAJC5B	DnaJ (Hsp40) homolog, subfamily C, member 5 beta [Source:HGNC Symbol;Acc:24138]	chr8	66933795	67012751	528	0.207	1	1	1.860E-02	3.780E-02	PB-GT
C7orf63	chromosome 7 open reading frame 63 [Source:HGNC Symbol;Acc:26107]	chr7	89874488	89940377	620	0.207	1	1	1.872E-02	3.800E-02	PB-GT
DPY19L3	dpy-19-like 3 (C. elegans) [Source:HGNC Symbol;Acc:27120]	chr19	32896655	32976801	603	0.207	1	1	1.873E-02	3.800E-02	PB-GT
SLC38A11	solute carrier family 38, member 11 [Source:HGNC Symbol;Acc:26836]	chr2	165752696	165812035	564	0.208	1	1	1.884E-02	3.819E-02	PB-GT
CSNK1E	casein kinase 1, epsilon [Source:HGNC Symbol;Acc:2453]	chr22	38686697	38794527	494	0.209	1	1	1.895E-02	3.839E-02	PB-GT
ZNF423	zinc finger protein 423 [Source:HGNC Symbol;Acc:16762]	chr16	49521435	49891830	1290	0.556	2	1	1.900E-02	3.845E-02	PB-GT
VPS45	vacuolar protein sorting 45 homolog (S. cerevisiae) [Source:HGNC Symbol;Acc:14579]	chr1	150039369	150117505	591	0.209	1	1	1.909E-02	3.861E-02	PB-GT
C11orf80	chromosome 11 open reading frame 80 [Source:HGNC Symbol;Acc:26197]	chr11	66511922	66610987	576	0.210	1	1	1.913E-02	3.866E-02	PB-GT
EIF2B5	eukaryotic translation initiation factor 2B, subunit 5 epsilon, 82kDa [Source:HGNC Symbol;Acc:3261]	chr3	183852826	184402546	1702	0.559	2	1	1.926E-02	3.887E-02	PB-GT
EYA2	eyes absent homolog 2 (Drosophila) [Source:HGNC Symbol;Acc:3520]	chr20	45523263	45817492	1504	0.559	2	1	1.926E-02	3.887E-02	PB-GT
BRWD3	bromodomain and WD repeat domain containing 3 [Source:HGNC Symbol;Acc:17342]	chrX	79926353	80065187	1264	0.211	1	1	1.930E-02	3.890E-02	PB-GT
SEL1L3	sel-1 suppressor of lin-12-like 3 (C. elegans) [Source:HGNC Symbol;Acc:29108]	chr4	25749055	25865382	648	0.212	1	1	1.958E-02	3.943E-02	PB-GT
C10orf118	chromosome 10 open reading frame 118 [Source:HGNC Symbol;Acc:24349]	chr10	115880621	115933979	493	0.212	1	1	1.959E-02	3.943E-02	PB-GT
KIAA1456	KIAA1456 [Source:HGNC Symbol;Acc:26725]	chr8	12803151	12889012	544	0.213	1	1	1.967E-02	3.956E-02	PB-GT
USP10	ubiquitin specific peptidase 10 [Source:HGNC Symbol;Acc:12608]	chr16	84733584	84813528	494	0.213	1	1	1.970E-02	3.959E-02	PB-GT
MAL2	mal, T-cell differentiation protein 2 (gene/pseudogene) [Source:HGNC Symbol;Acc:13634]	chr8	120177273	120257913	549	0.215	1	1	2.000E-02	4.018E-02	PB-GT
ERGIC2	ERGIC and golgi 2 [Source:HGNC Symbol;Acc:30208]	chr12	29490285	29534122	487	0.215	1	1	2.004E-02	4.022E-02	PB-GT
SH3RF3	SH3 domain containing ring finger 3 [Source:HGNC Symbol;Acc:24699]	chr2	109745804	110262207	1541	0.568	2	2	2.008E-02	4.025E-02	PB-GT
RASGRF2	Ras protein-specific guanine nucleotide-releasing factor 2 [Source:HGNC Symbol;Acc:9876]	chr5	80256491	80525975	1740	0.568	2	1	2.009E-02	4.025E-02	PB-GT
TMED3	transmembrane emp24 protein transport domain containing 3 [Source:HGNC Symbol;Acc:28889]	chr15	79603404	79704334	586	0.215	1	1	2.013E-02	4.030E-02	PB-GT
ZPLD1	zona pellucida-like domain containing 1 [Source:HGNC Symbol;Acc:27022]	chr3	101818088	102198685	3103	1.019	3	1	2.019E-02	4.039E-02	PB-GT
C7orf55-LUC7L2	C7orf55-LUC7L2 readthrough [Source:HGNC Symbol;Acc:44671]	chr7	139025105	139108198	648	0.217	1	1	2.032E-02	4.063E-02	PB-GT
TMLHE	trimethyllysine hydroxylase, epsilon [Source:HGNC Symbol;Acc:18308]	chrX	154719776	154899605	1301	0.217	1	1	2.036E-02	4.067E-02	PB-GT
EXOC5	exocyst complex component 5 [Source:HGNC Symbol;Acc:10696]	chr14	57670518	57735726	605	0.217	1	1	2.041E-02	4.069E-02	PB-GT
ST6GAL2	ST6 beta-galactosamide alpha-2,6-sialyltranferase 2 [Source:HGNC Symbol;Acc:10861]	chr2	107418056	107503564	589	0.217	1	1	2.042E-02	4.069E-02	PB-GT
RANBP2	RAN binding protein 2 [Source:HGNC Symbol;Acc:9848]	chr2	109335937	109402267	589	0.217	1	1	2.042E-02	4.069E-02	PB-GT
CR1L	complement component (3b/4b) receptor 1-like [Source:HGNC Symbol;Acc:2335]	chr1	207818458	207911761	613	0.217	1	1	2.043E-02	4.069E-02	PB-GT
RCOR3	REST corepressor 3 [Source:HGNC Symbol;Acc:25594]	chr1	211431719	211489727	614	0.218	1	1	2.049E-02	4.078E-02	PB-GT
USP43	ubiquitin specific peptidase 43 [Source:HGNC Symbol;Acc:20072]	chr17	9548015	9633001	411	0.218	1	1	2.054E-02	4.083E-02	PB-GT
IWS1	IWS1 homolog (S. cerevisiae) [Source:HGNC Symbol;Acc:25467]	chr2	128193783	128284462	591	0.218	1	1	2.055E-02	4.083E-02	PB-GT
ZC3H6	zinc finger CCCH-type containing 6 [Source:HGNC Symbol;Acc:24762]	chr2	113033171	113097640	592	0.218	1	1	2.061E-02	4.093E-02	PB-GT
RALY	RNA binding protein, autoantigenic (hnRNP-associated with lethal yellow homolog (mouse)) [Source:HGNC Symbol;Acc:15921]	chr20	32581452	32696114	588	0.219	1	1	2.067E-02	4.102E-02	PB-GT
MFSD1	major facilitator superfamily domain containing 1 [Source:HGNC Symbol;Acc:25874]	chr3	158449987	158547508	666	0.219	1	1	2.071E-02	4.105E-02	PB-GT
ANKRD30BL	ankyrin repeat domain 30B-like [Source:HGNC Symbol;Acc:35167]	chr2	132905164	133015542	594	0.219	1	1	2.074E-02	4.109E-02	PB-GT
LIPI	lipase, member I [Source:HGNC Symbol;Acc:18821]	chr21	15481134	15583166	866	0.219	1	1	2.076E-02	4.110E-02	PB-GT
FSBP	fibrinogen silencer binding protein [Source:HGNC Symbol;Acc:43653]	chr8	95390605	95449180	561	0.219	1	1	2.082E-02	4.119E-02	PB-GT
EFHC1	EF-hand domain (C-terminal) containing 1 [Source:HGNC Symbol;Acc:16406]	chr6	52285106	52387892	622	0.220	1	1	2.090E-02	4.130E-02	PB-GT
ROCK2	Rho-associated, coiled-coil containing protein kinase 2 [Source:HGNC Symbol;Acc:10252]	chr2	11319887	11488456	1569	0.578	2	1	2.103E-02	4.154E-02	PB-GT
CCDC93	coiled-coil domain containing 93 [Source:HGNC Symbol;Acc:25611]	chr2	118673054	118771709	599	0.221	1	1	2.107E-02	4.158E-02	PB-GT
ELL2	elongation factor, RNA polymerase II, 2 [Source:HGNC Symbol;Acc:17064]	chr5	95220802	95297775	677	0.221	1	1	2.111E-02	4.164E-02	PB-GT
ITGB8	integrin, beta 8 [Source:HGNC Symbol;Acc:6163]	chr7	20370325	20450419	662	0.221	1	1	2.115E-02	4.167E-02	PB-GT
B3GNTL1	UDP-GlcNAc:betaGal beta-1,3-N-acetylglucosaminyltransferase-like 1 [Source:HGNC Symbol;Acc:21727]	chr17	80900031	81009686	418	0.222	1	1	2.119E-02	4.171E-02	PB-GT
ESF1	ESF1, nucleolar pre-rRNA processing protein, homolog (S. cerevisiae) [Source:HGNC Symbol;Acc:15898]	chr20	13694969	13765532	596	0.222	1	1	2.120E-02	4.171E-02	PB-GT
ZNF131	zinc finger protein 131 [Source:HGNC Symbol;Acc:12915]	chr5	43065278	43192123	679	0.222	1	1	2.123E-02	4.174E-02	PB-GT
PARN	poly(A)-specific ribonuclease [Source:HGNC Symbol;Acc:8609]	chr16	14529558	14726585	1347	0.581	2	2	2.124E-02	4.174E-02	PB-GT
ADARB2	adenosine deaminase, RNA-specific, B2 [Source:HGNC Symbol;Acc:227]	chr10	1228073	1779670	2406	1.036	3	2	2.128E-02	4.175E-02	PB-GT
ST3GAL6	ST3 beta-galactoside alpha-2,3-sialyltransferase 6 [Source:HGNC Symbol;Acc:18080]	chr3	98451080	98540045	676	0.222	1	1	2.129E-02	4.175E-02	PB-GT
SPEN	spen homolog, transcriptional regulator (Drosophila) [Source:HGNC Symbol;Acc:17575]	chr1	16174359	16266955	627	0.222	1	1	2.131E-02	4.175E-02	PB-GT
EIF2AK3	eukaryotic translation initiation factor 2-alpha kinase 3 [Source:HGNC Symbol;Acc:3255]	chr2	88856259	88927094	603	0.222	1	1	2.133E-02	4.175E-02	PB-GT
TDP1	tyrosyl-DNA phosphodiesterase 1 [Source:HGNC Symbol;Acc:18884]	chr14	90421283	90511106	620	0.222	1	1	2.136E-02	4.175E-02	PB-GT
SEC23A	Sec23 homolog A (S. cerevisiae) [Source:HGNC Symbol;Acc:10701]	chr14	39501123	39578850	620	0.222	1	1	2.136E-02	4.175E-02	PB-GT
CDH17	cadherin 17, LI cadherin (liver-intestine) [Source:HGNC Symbol;Acc:1756]	chr8	95139399	95229531	569	0.223	1	1	2.138E-02	4.175E-02	PB-GT
TBL1X	transducin (beta)-like 1X-linked [Source:HGNC Symbol;Acc:11585]	chrX	9431335	9687780	1336	0.223	1	1	2.139E-02	4.175E-02	PB-GT
LRIG1	leucine-rich repeats and immunoglobulin-like domains 1 [Source:HGNC Symbol;Acc:17360]	chr3	66429221	66551687	678	0.223	1	1	2.141E-02	4.175E-02	PB-GT
GLYAT	glycine-N-acyltransferase [Source:HGNC Symbol;Acc:13734]	chr11	58407899	58499447	612	0.223	1	1	2.141E-02	4.175E-02	PB-GT
UBE2R2	ubiquitin-conjugating enzyme E2R 2 [Source:HGNC Symbol;Acc:19907]	chr9	33817565	33920402	680	0.223	1	1	2.143E-02	4.176E-02	PB-GT
NUP214	nucleoporin 214kDa [Source:HGNC Symbol;Acc:8064]	chr9	134000948	134110057	681	0.223	1	1	2.149E-02	4.181E-02	PB-GT
FUT10	fucosyltransferase 10 (alpha (1,3) fucosyltransferase) [Source:HGNC Symbol;Acc:19234]	chr8	33228342	33330940	571	0.223	1	1	2.152E-02	4.181E-02	PB-GT
HIVEP2	human immunodeficiency virus type I enhancer binding protein 2 [Source:HGNC Symbol;Acc:4921]	chr6	143072604	143266338	1651	0.584	2	2	2.152E-02	4.181E-02	PB-GT
PPP3CA	protein phosphatase 3, catalytic subunit, alpha isozyme [Source:HGNC Symbol;Acc:9314]	chr4	101944566	102269435	3174	1.040	3	2	2.152E-02	4.181E-02	PB-GT
PCBP1-AS1	PCBP1 antisense RNA 1 [Source:HGNC Symbol;Acc:42948]	chr2	70189395	70315978	607	0.224	1	1	2.159E-02	4.185E-02	PB-GT
HIRA	HIR histone cell cycle regulation defective homolog A (S. cerevisiae) [Source:HGNC Symbol;Acc:4916]	chr22	19318221	19435224	530	0.224	1	1	2.160E-02	4.185E-02	PB-GT
MAPK6	mitogen-activated protein kinase 6 [Source:HGNC Symbol;Acc:6879]	chr15	52244303	52358462	609	0.224	1	1	2.162E-02	4.185E-02	PB-GT
CAPZB	capping protein (actin filament) muscle Z-line, beta [Source:HGNC Symbol;Acc:1491]	chr1	19665267	19812066	632	0.224	1	1	2.162E-02	4.185E-02	PB-GT
LINC00607	long intergenic non-protein coding RNA 607 [Source:HGNC Symbol;Acc:43944]	chr2	216476286	216708445	1586	0.585	2	1	2.163E-02	4.185E-02	PB-GT
MBP	myelin basic protein [Source:HGNC Symbol;Acc:6925]	chr18	74690783	74845639	702	0.225	1	1	2.175E-02	4.205E-02	PB-GT
PPM1D	protein phosphatase, Mg2+/Mn2+ dependent, 1D [Source:HGNC Symbol;Acc:9277]	chr17	58677544	58741849	424	0.225	1	1	2.176E-02	4.205E-02	PB-GT
ZFHX3	zinc finger homeobox 3 [Source:HGNC Symbol;Acc:777]	chr16	72816784	73093597	1360	0.586	2	1	2.178E-02	4.205E-02	PB-GT
MON2	MON2 homolog (S. cerevisiae) [Source:HGNC Symbol;Acc:29177]	chr12	62860597	62991363	1329	0.587	2	1	2.180E-02	4.207E-02	PB-GT
KRAS	v-Ki-ras2 Kirsten rat sarcoma viral oncogene homolog [Source:HGNC Symbol;Acc:6407]	chr12	25357723	25403870	510	0.225	1	1	2.183E-02	4.209E-02	PB-GT
AP2B1	adaptor-related protein complex 2, beta 1 subunit [Source:HGNC Symbol;Acc:563]	chr17	33905065	34053436	1108	0.587	2	2	2.187E-02	4.213E-02	PB-GT
C5orf64	chromosome 5 open reading frame 64 [Source:HGNC Symbol;Acc:26744]	chr5	60933535	61047590	691	0.226	1	1	2.193E-02	4.220E-02	PB-GT
ZMIZ1	zinc finger, MIZ-type containing 1 [Source:HGNC Symbol;Acc:16493]	chr10	80828792	81076276	524	0.226	1	1	2.194E-02	4.220E-02	PB-GT
DNAH7	dynein, axonemal, heavy chain 7 [Source:HGNC Symbol;Acc:18661]	chr2	196602427	196933536	2838	1.046	3	2	2.195E-02	4.220E-02	PB-GT
TRIP11	thyroid hormone receptor interactor 11 [Source:HGNC Symbol;Acc:12305]	chr14	92432335	92507240	630	0.226	1	1	2.201E-02	4.227E-02	PB-GT
CEP70	centrosomal protein 70kDa [Source:HGNC Symbol;Acc:29972]	chr3	138213186	138313380	690	0.227	1	1	2.211E-02	4.242E-02	PB-GT
SMEK2	SMEK homolog 2, suppressor of mek1 (Dictyostelium) [Source:HGNC Symbol;Acc:29267]	chr2	55774428	55846015	615	0.227	1	1	2.212E-02	4.242E-02	PB-GT
ADCY10	adenylate cyclase 10 (soluble) [Source:HGNC Symbol;Acc:21285]	chr1	167778625	167883453	640	0.227	1	1	2.213E-02	4.242E-02	PB-GT
LRCH3	leucine-rich repeats and calponin homology (CH) domain containing 3 [Source:HGNC Symbol;Acc:28637]	chr3	197518097	197615307	691	0.227	1	1	2.217E-02	4.247E-02	PB-GT
NVL	nuclear VCP-like [Source:HGNC Symbol;Acc:8070]	chr1	224415036	224518089	641	0.227	1	1	2.220E-02	4.248E-02	PB-GT
KLHL3	kelch-like family member 3 [Source:HGNC Symbol;Acc:6354]	chr5	136953189	137071779	696	0.227	1	1	2.222E-02	4.251E-02	PB-GT
COL4A2	collagen, type IV, alpha 2 [Source:HGNC Symbol;Acc:2203]	chr13	110958159	111165374	943	0.228	1	1	2.230E-02	4.262E-02	PB-GT
DOCK11	dedicator of cytokinesis 11 [Source:HGNC Symbol;Acc:23483]	chrX	117629861	117820126	1367	0.228	1	1	2.232E-02	4.262E-02	PB-GT
PIK3R1	phosphoinositide-3-kinase, regulatory subunit 1 (alpha) [Source:HGNC Symbol;Acc:8979]	chr5	67511548	67597649	700	0.229	1	1	2.246E-02	4.282E-02	PB-GT
OSMR	oncostatin M receptor [Source:HGNC Symbol;Acc:8507]	chr5	38845960	38945698	700	0.229	1	1	2.246E-02	4.282E-02	PB-GT
DIRC2	disrupted in renal carcinoma 2 [Source:HGNC Symbol;Acc:16628]	chr3	122513642	122599986	696	0.229	1	1	2.247E-02	4.282E-02	PB-GT
SMURF1	SMAD specific E3 ubiquitin protein ligase 1 [Source:HGNC Symbol;Acc:16807]	chr7	98625061	98741723	686	0.229	1	1	2.259E-02	4.301E-02	PB-GT
GABRA4	gamma-aminobutyric acid (GABA) A receptor, alpha 4 [Source:HGNC Symbol;Acc:4078]	chr4	46920917	46996424	702	0.230	1	1	2.272E-02	4.319E-02	PB-GT
FBXL5	F-box and leucine-rich repeat protein 5 [Source:HGNC Symbol;Acc:13602]	chr4	15606162	15683302	702	0.230	1	1	2.272E-02	4.319E-02	PB-GT
HIP1	huntingtin interacting protein 1 [Source:HGNC Symbol;Acc:4913]	chr7	75162621	75368280	689	0.230	1	1	2.277E-02	4.322E-02	PB-GT
CTNNA1	catenin (cadherin-associated protein), alpha 1, 102kDa [Source:HGNC Symbol;Acc:2509]	chr5	137946656	138270723	1827	0.597	2	1	2.278E-02	4.322E-02	PB-GT
FBXO34	F-box protein 34 [Source:HGNC Symbol;Acc:20201]	chr14	55738021	55828636	642	0.230	1	1	2.279E-02	4.322E-02	PB-GT
TRDMT1	tRNA aspartic acid methyltransferase 1 [Source:HGNC Symbol;Acc:2977]	chr10	17184253	17244053	535	0.230	1	1	2.280E-02	4.322E-02	PB-GT
PITPNM2	phosphatidylinositol transfer protein, membrane-associated 2 [Source:HGNC Symbol;Acc:21044]	chr12	123468027	123634562	523	0.231	1	1	2.287E-02	4.333E-02	PB-GT
DLEU7	deleted in lymphocytic leukemia, 7 [Source:HGNC Symbol;Acc:17567]	chr13	51285144	51418075	957	0.231	1	1	2.292E-02	4.338E-02	PB-GT
ARHGAP17	Rho GTPase activating protein 17 [Source:HGNC Symbol;Acc:18239]	chr16	24930706	25026987	538	0.232	1	1	2.307E-02	4.365E-02	PB-GT
PAX5	paired box 5 [Source:HGNC Symbol;Acc:8619]	chr9	36833272	37034103	708	0.232	1	1	2.310E-02	4.366E-02	PB-GT
SPOPL	speckle-type POZ protein-like [Source:HGNC Symbol;Acc:27934]	chr2	139259371	139331117	630	0.232	1	1	2.313E-02	4.370E-02	PB-GT
GPR156	G protein-coupled receptor 156 [Source:HGNC Symbol;Acc:20844]	chr3	119883492	120003941	708	0.233	1	1	2.319E-02	4.378E-02	PB-GT
KANK1	KN motif and ankyrin repeat domains 1 [Source:HGNC Symbol;Acc:19309]	chr9	470291	746105	1834	0.601	2	1	2.322E-02	4.380E-02	PB-GT
MAN1C1	mannosidase, alpha, class 1C, member 1 [Source:HGNC Symbol;Acc:19080]	chr1	25943959	26112698	658	0.233	1	1	2.330E-02	4.391E-02	PB-GT
SLC7A14	solute carrier family 7 (orphan transporter), member 14 [Source:HGNC Symbol;Acc:29326]	chr3	170182353	170303863	710	0.233	1	1	2.331E-02	4.391E-02	PB-GT
MRPL33	mitochondrial ribosomal protein L33 [Source:HGNC Symbol;Acc:14487]	chr2	27994584	28210954	1635	0.603	2	1	2.338E-02	4.401E-02	PB-GT
SLC22A3	solute carrier family 22 (extraneuronal monoamine transporter), member 3 [Source:HGNC Symbol;Acc:10967]	chr6	160769300	160876014	662	0.234	1	1	2.345E-02	4.411E-02	PB-GT
LIFR-AS1	LIFR antisense RNA 1 [Source:HGNC Symbol;Acc:43600]	chr5	38556888	38671318	717	0.234	1	1	2.348E-02	4.413E-02	PB-GT
WDHD1	WD repeat and HMG-box DNA binding protein 1 [Source:HGNC Symbol;Acc:23170]	chr14	55405668	55493823	653	0.234	1	1	2.351E-02	4.414E-02	PB-GT
C6orf183	chromosome 6 open reading frame 183 [Source:HGNC Symbol;Acc:21562]	chr6	109487036	109592217	663	0.234	1	1	2.352E-02	4.414E-02	PB-GT
CMTM8	CKLF-like MARVEL transmembrane domain containing 8 [Source:HGNC Symbol;Acc:19179]	chr3	32280171	32411817	717	0.236	1	1	2.374E-02	4.452E-02	PB-GT
KIAA0232	KIAA0232 [Source:HGNC Symbol;Acc:28992]	chr4	6783102	6885897	720	0.236	1	1	2.380E-02	4.461E-02	PB-GT
IFT88	intraflagellar transport 88 homolog (Chlamydomonas) [Source:HGNC Symbol;Acc:20606]	chr13	21140585	21265503	978	0.236	1	1	2.385E-02	4.467E-02	PB-GT
C9orf123	chromosome 9 open reading frame 123 [Source:HGNC Symbol;Acc:30536]	chr9	7796490	7888380	721	0.236	1	1	2.388E-02	4.470E-02	PB-GT
SUSD1	sushi domain containing 1 [Source:HGNC Symbol;Acc:25413]	chr9	114803065	114937688	722	0.237	1	1	2.395E-02	4.476E-02	PB-GT
TMCO1	transmembrane and coiled-coil domains 1 [Source:HGNC Symbol;Acc:18188]	chr1	165696032	165796992	668	0.237	1	1	2.396E-02	4.476E-02	PB-GT
SCUBE2	signal peptide, CUB domain, EGF-like 2 [Source:HGNC Symbol;Acc:30425]	chr11	9041071	9159661	651	0.237	1	1	2.400E-02	4.482E-02	PB-GT
NSMAF	neutral sphingomyelinase (N-SMase) activation associated factor [Source:HGNC Symbol;Acc:8017]	chr8	59496063	59572403	608	0.238	1	1	2.417E-02	4.507E-02	PB-GT
XXYLT1	xyloside xylosyltransferase 1 [Source:HGNC Symbol;Acc:26639]	chr3	194789008	194991896	724	0.238	1	1	2.417E-02	4.507E-02	PB-GT
SGCE	sarcoglycan, epsilon [Source:HGNC Symbol;Acc:10808]	chr7	94214542	94285521	714	0.239	1	1	2.432E-02	4.531E-02	PB-GT
CEP120	centrosomal protein 120kDa [Source:HGNC Symbol;Acc:26690]	chr5	122680579	122759286	731	0.239	1	1	2.433E-02	4.531E-02	PB-GT
SLAIN2	SLAIN motif family, member 2 [Source:HGNC Symbol;Acc:29282]	chr4	48343339	48428229	730	0.239	1	1	2.442E-02	4.543E-02	PB-GT
ECT2L	epithelial cell transforming sequence 2 oncogene-like [Source:HGNC Symbol;Acc:21118]	chr6	139117063	139225207	677	0.239	1	1	2.444E-02	4.545E-02	PB-GT
KIF15	kinesin family member 15 [Source:HGNC Symbol;Acc:17273]	chr3	44803209	44914868	730	0.240	1	1	2.454E-02	4.559E-02	PB-GT
ABR	active BCR-related [Source:HGNC Symbol;Acc:81]	chr17	906758	1132315	454	0.241	1	1	2.469E-02	4.582E-02	PB-GT
ZNF516	zinc finger protein 516 [Source:HGNC Symbol;Acc:28990]	chr18	74069644	74207146	752	0.241	1	1	2.470E-02	4.582E-02	PB-GT
IQUB	IQ motif and ubiquitin domain containing [Source:HGNC Symbol;Acc:21995]	chr7	123092454	123175131	721	0.241	1	1	2.476E-02	4.590E-02	PB-GT
NMNAT3	nicotinamide nucleotide adenylyltransferase 3 [Source:HGNC Symbol;Acc:20989]	chr3	139279022	139396859	734	0.241	1	1	2.479E-02	4.590E-02	PB-GT
FAM107B	family with sequence similarity 107, member B [Source:HGNC Symbol;Acc:23726]	chr10	14560556	14816896	1432	0.617	2	2	2.480E-02	4.590E-02	PB-GT
MTNR1A	melatonin receptor 1A [Source:HGNC Symbol;Acc:7463]	chr4	187347700	187476721	737	0.241	1	1	2.485E-02	4.598E-02	PB-GT
ALPK2	alpha-kinase 2 [Source:HGNC Symbol;Acc:20565]	chr18	56148479	56296189	755	0.242	1	1	2.488E-02	4.599E-02	PB-GT
HMGB1	high mobility group box 1 [Source:HGNC Symbol;Acc:4983]	chr13	31032884	31191734	1001	0.242	1	1	2.490E-02	4.600E-02	PB-GT
STAT5B	signal transducer and activator of transcription 5B [Source:HGNC Symbol;Acc:11367]	chr17	40351186	40428725	457	0.242	1	1	2.499E-02	4.609E-02	PB-GT
CALM2	calmodulin 2 (phosphorylase kinase, delta) [Source:HGNC Symbol;Acc:1445]	chr2	47272677	47403740	657	0.242	1	1	2.500E-02	4.609E-02	PB-GT
RASSF3	Ras association (RalGDS/AF-6) domain family member 3 [Source:HGNC Symbol;Acc:14271]	chr12	65004293	65091347	549	0.242	1	1	2.501E-02	4.609E-02	PB-GT
VWDE	von Willebrand factor D and EGF domains [Source:HGNC Symbol;Acc:21897]	chr7	12370511	12443567	725	0.242	1	1	2.502E-02	4.609E-02	PB-GT
PIK3C2G	phosphatidylinositol-4-phosphate 3-kinase, catalytic subunit type 2 gamma [Source:HGNC Symbol;Acc:8973]	chr12	18400548	18801348	3681	1.625	4	2	2.506E-02	4.613E-02	PB-GT
UBASH3B	ubiquitin associated and SH3 domain containing B [Source:HGNC Symbol;Acc:29884]	chr11	122526383	122685181	667	0.243	1	1	2.510E-02	4.616E-02	PB-GT
TEAD1	TEA domain family member 1 (SV40 transcriptional enhancer factor) [Source:HGNC Symbol;Acc:11714]	chr11	12695969	12966298	1703	0.620	2	1	2.511E-02	4.616E-02	PB-GT
WDR11	WD repeat domain 11 [Source:HGNC Symbol;Acc:13831]	chr10	122610687	122669036	564	0.243	1	1	2.513E-02	4.616E-02	PB-GT
KIAA1549L	KIAA1549-like [Source:HGNC Symbol;Acc:24836]	chr11	33563618	33695648	669	0.243	1	1	2.524E-02	4.633E-02	PB-GT
CPNE3	copine III [Source:HGNC Symbol;Acc:2316]	chr8	87497059	87573726	623	0.244	1	1	2.528E-02	4.634E-02	PB-GT
TOP2B	topoisomerase (DNA) II beta 180kDa [Source:HGNC Symbol;Acc:11990]	chr3	25639475	25706398	742	0.244	1	1	2.529E-02	4.634E-02	PB-GT
ARL17B	ADP-ribosylation factor-like 17B [Source:HGNC Symbol;Acc:32387]	chr17	44352150	44439130	460	0.244	1	1	2.530E-02	4.634E-02	PB-GT
ARID4A	AT rich interactive domain 4A (RBP1-like) [Source:HGNC Symbol;Acc:9885]	chr14	58765103	58840605	680	0.244	1	1	2.534E-02	4.638E-02	PB-GT
CROCC	ciliary rootlet coiled-coil, rootletin [Source:HGNC Symbol;Acc:21299]	chr1	17066768	17299474	689	0.244	1	1	2.536E-02	4.639E-02	PB-GT
ZMYND8	zinc finger, MYND-type containing 8 [Source:HGNC Symbol;Acc:9397]	chr20	45837859	45985567	658	0.245	1	1	2.545E-02	4.653E-02	PB-GT
RAB11FIP4	RAB11 family interacting protein 4 (class II) [Source:HGNC Symbol;Acc:30267]	chr17	29718642	29865236	463	0.245	1	1	2.560E-02	4.673E-02	PB-GT
MB21D2	Mab-21 domain containing 2 [Source:HGNC Symbol;Acc:30438]	chr3	192514604	192635950	747	0.245	1	1	2.560E-02	4.673E-02	PB-GT
RBM6	RNA binding motif protein 6 [Source:HGNC Symbol;Acc:9903]	chr3	49977440	50137478	748	0.246	1	1	2.567E-02	4.682E-02	PB-GT
ABLIM1	actin binding LIM protein 1 [Source:HGNC Symbol;Acc:78]	chr10	116190872	116444762	1453	0.626	2	2	2.573E-02	4.691E-02	PB-GT
BCL2L13	BCL2-like 13 (apoptosis facilitator) [Source:HGNC Symbol;Acc:17164]	chr22	18111621	18213388	583	0.246	1	1	2.575E-02	4.691E-02	PB-GT
IPO11	importin 11 [Source:HGNC Symbol;Acc:20628]	chr5	61699799	61924409	1919	0.627	2	1	2.582E-02	4.701E-02	PB-GT
MICAL2	microtubule associated monooxygenase, calponin and LIM domain containing 2 [Source:HGNC Symbol;Acc:24693]	chr11	12115543	12285334	678	0.247	1	1	2.587E-02	4.703E-02	PB-GT

table S1. Genes with significant transposon integrations in DLD-1 *KRAS*$^{wt/-}$ cells after selection in low glucose medium.

Gene ID	Description	Chrom	Start	End	# TTAA sites	Expected	Observed	Libraries	*P*	FDR	Transposon
TEX36	testis expressed 36 [Source:HGNC Symbol;Acc:31653]	chr10	127265091	127371713	573	0.247	1	1	2.587E-02	4.703E-02	PB-GT
ERN1	endoplasmic reticulum to nucleus signaling 1 [Source:HGNC Symbol;Acc:3449]	chr17	62120353	62207504	467	0.247	1	1	2.601E-02	4.724E-02	PB-GT
ETV6	ets variant 6 [Source:HGNC Symbol;Acc:3495]	chr12	11802788	12044558	1425	0.629	2	1	2.606E-02	4.731E-02	PB-GT
TOX2	TOX high mobility group box family member 2 [Source:HGNC Symbol;Acc:16095]	chr20	42543492	42698256	667	0.248	1	1	2.610E-02	4.734E-02	PB-GT
WWC1	WW and C2 domain containing 1 [Source:HGNC Symbol;Acc:29435]	chr5	167718656	167899308	762	0.249	1	1	2.627E-02	4.761E-02	PB-GT
LINC00616	long intergenic non-protein coding RNA 616 [Source:HGNC Symbol;Acc:44065]	chr4	138948576	139051863	760	0.249	1	1	2.630E-02	4.761E-02	PB-GT
TNPO3	transportin 3 [Source:HGNC Symbol;Acc:17103]	chr7	128594948	128695198	745	0.249	1	1	2.630E-02	4.761E-02	PB-GT
AKAP12	A kinase (PRKA) anchor protein 12 [Source:HGNC Symbol;Acc:370]	chr6	151561134	151679692	707	0.250	1	1	2.648E-02	4.789E-02	PB-GT
BTBD10	BTB (POZ) domain containing 10 [Source:HGNC Symbol;Acc:21445]	chr11	13409548	13484844	687	0.250	1	1	2.650E-02	4.791E-02	PB-GT
ZBTB44	zinc finger and BTB domain containing 44 [Source:HGNC Symbol;Acc:25001]	chr11	130096572	130184581	689	0.251	1	1	2.664E-02	4.812E-02	PB-GT
GPR137C	G protein-coupled receptor 137C [Source:HGNC Symbol;Acc:25445]	chr14	53019866	53104431	699	0.251	1	1	2.666E-02	4.812E-02	PB-GT
ANXA4	annexin A4 [Source:HGNC Symbol;Acc:542]	chr2	69947923	70053596	682	0.251	1	1	2.677E-02	4.829E-02	PB-GT
NDUFA12	NADH dehydrogenase (ubiquinone) 1 alpha subcomplex, 12 [Source:HGNC Symbol;Acc:23987]	chr12	95290831	95397546	570	0.252	1	1	2.680E-02	4.831E-02	PB-GT
WASF1	WAS protein family, member 1 [Source:HGNC Symbol;Acc:12732]	chr6	110421022	110501207	712	0.252	1	1	2.682E-02	4.831E-02	PB-GT
EP300	E1A binding protein p300 [Source:HGNC Symbol;Acc:3373]	chr22	41487790	41576081	598	0.252	1	1	2.698E-02	4.856E-02	PB-GT
FLRT2	fibronectin leucine rich transmembrane protein 2 [Source:HGNC Symbol;Acc:3761]	chr14	85996488	86095034	704	0.253	1	1	2.701E-02	4.856E-02	PB-GT
ZNF420	zinc finger protein 420 [Source:HGNC Symbol;Acc:20649]	chr19	37498759	37621216	735	0.253	1	1	2.701E-02	4.856E-02	PB-GT
SH3TC2	SH3 domain and tetratricopeptide repeats 2 [Source:HGNC Symbol;Acc:29427]	chr5	148303202	148442726	774	0.253	1	1	2.703E-02	4.856E-02	PB-GT
LINC00290	long intergenic non-protein coding RNA 290 [Source:HGNC Symbol;Acc:38515]	chr4	181985242	182080302	774	0.254	1	1	2.719E-02	4.882E-02	PB-GT
GPATCH2L	G patch domain containing 2-like [Source:HGNC Symbol;Acc:20210]	chr14	76618259	76720685	707	0.254	1	1	2.722E-02	4.883E-02	PB-GT
SLC8A3	solute carrier family 8 (sodium/calcium exchanger), member 3 [Source:HGNC Symbol;Acc:11070]	chr14	70510934	70655787	708	0.254	1	1	2.729E-02	4.892E-02	PB-GT
VIT	vitrin [Source:HGNC Symbol;Acc:12697]	chr2	36923833	37041935	691	0.255	1	1	2.742E-02	4.913E-02	PB-GT
SYNCRIP	synaptotagmin binding, cytoplasmic RNA interacting protein [Source:HGNC Symbol;Acc:16918]	chr6	86267696	86353510	721	0.255	1	1	2.745E-02	4.913E-02	PB-GT
CHST9	carbohydrate (N-acetylgalactosamine 4-0) sulfotransferase 9 [Source:HGNC Symbol;Acc:19898]	chr18	24495595	24765281	2007	0.642	2	2	2.749E-02	4.917E-02	PB-GT
KIAA1377	KIAA1377 [Source:HGNC Symbol;Acc:29264]	chr11	101785746	101871789	701	0.255	1	1	2.750E-02	4.917E-02	PB-GT
NHLRC2	NHL repeat containing 2 [Source:HGNC Symbol;Acc:24731]	chr10	115614420	115676953	593	0.255	1	1	2.755E-02	4.923E-02	PB-GT
ARL15	ADP-ribosylation factor-like 15 [Source:HGNC Symbol;Acc:25945]	chr5	53179775	53606412	3445	1.125	3	2	2.762E-02	4.931E-02	PB-GT
SLC24A4	solute carrier family 24 (sodium/potassium/calcium exchanger), member 4 [Source:HGNC Symbol;Acc:10978]	chr14	92788925	92962596	715	0.257	1	1	2.779E-02	4.957E-02	PB-GT
FRMPD2	FERM and PDZ domain containing 2 [Source:HGNC Symbol;Acc:28572]	chr10	49364601	49482941	596	0.257	1	1	2.781E-02	4.958E-02	PB-GT
MAP3K1	mitogen-activated protein kinase kinase kinase 1, E3 ubiquitin protein ligase [Source:HGNC Symbol;Acc:6848]	chr5	56111401	56191979	787	0.257	1	1	2.787E-02	4.966E-02	PB-GT
YTHDC2	YTH domain containing 2 [Source:HGNC Symbol;Acc:24721]	chr5	112849380	112930982	788	0.257	1	1	2.794E-02	4.970E-02	PB-GT
C6orf106	chromosome 6 open reading frame 106 [Source:HGNC Symbol;Acc:21215]	chr6	34555065	34664636	728	0.257	1	1	2.794E-02	4.970E-02	PB-GT
ZNF652	zinc finger protein 652 [Source:HGNC Symbol;Acc:29147]	chr17	47366568	47439835	486	0.258	1	1	2.798E-02	4.975E-02	PB-GT
PRDM6	PR domain containing 6 [Source:HGNC Symbol;Acc:9350]	chr5	122424816	122523745	789	0.258	1	1	2.800E-02	4.975E-02	PB-GT
MACC1	metastasis associated in colon cancer 1 [Source:HGNC Symbol;Acc:30215]	chr7	20174278	20257027	772	0.258	1	1	2.808E-02	4.984E-02	PB-GT
RFFL	ring finger and FYVE-like domain containing E3 ubiquitin protein ligase [Source:HGNC Symbol;Acc:24821]	chr17	33333009	33416338	487	0.258	1	1	2.809E-02	4.984E-02	PB-GT
RBMXP1	RNA binding motif protein, X-linked pseudogene 1 [Source:HGNC Symbol;Acc:9911]	chr6	48181340	48187530	44	0.003	2	1	3.668E-09	2.568E-06	PB-CAG-SD
LY6E	lymphocyte antigen 6 complex, locus E [Source:HGNC Symbol;Acc:6727]	chr8	144094399	144105249	7	0.001	1	1	1.370E-07	4.794E-05	PB-CAG-SD
ALPK2	alpha-kinase 2 [Source:HGNC Symbol;Acc:20565]	chr18	56148479	56301189	777	0.051	3	1	2.682E-07	5.015E-05	PB-CAG-SD
MIR4477A	microRNA 4477a [Source:HGNC Symbol;Acc:41859]	chr9	68410308	68415388	14	0.001	1	1	3.397E-07	5.015E-05	PB-CAG-SD
TMSB4XP6	thymosin beta 4, X-linked pseudogene 6 [Source:HGNC Symbol;Acc:11888]	chr20	49457152	49462286	11	0.001	1	1	4.301E-07	5.015E-05	PB-CAG-SD
PTGES2	prostaglandin E synthase 2 [Source:HGNC Symbol;Acc:17822]	chr9	130882972	130895741	17	0.001	1	1	5.008E-07	5.015E-05	PB-CAG-SD
DLGAP1	discs, large (Drosophila) homolog-associated protein 1 [Source:HGNC Symbol;Acc:2905]	chr18	3496030	4460335	6873	0.450	6	2	5.015E-07	5.015E-05	PB-CAG-SD
ABHD11	abhydrolase domain containing 11 [Source:HGNC Symbol;Acc:16407]	chr7	73150424	73158197	18	0.001	1	1	6.393E-07	5.569E-05	PB-CAG-SD
RN7SL278P	RNA, 7SL, cytoplasmic 278, pseudogene [Source:HGNC Symbol;Acc:46294]	chr15	77269258	77274551	17	0.001	1	1	7.806E-07	5.569E-05	PB-CAG-SD
KRCC1	lysine-rich coiled-coil 1 [Source:HGNC Symbol;Acc:28039]	chr2	88326724	88360248	242	0.017	2	2	7.994E-07	5.569E-05	PB-CAG-SD
MIR4514	microRNA 4514 [Source:HGNC Symbol;Acc:41845]	chr15	81289758	81294814	18	0.001	1	1	8.751E-07	5.569E-05	PB-CAG-SD
KRT8P23	keratin 8 pseudogene 23 [Source:HGNC Symbol;Acc:33375]	chr15	77271586	77277792	19	0.001	1	1	9.749E-07	5.687E-05	PB-CAG-SD
PKN3	protein kinase N3 [Source:HGNC Symbol;Acc:17999]	chr9	131459802	131483197	25	0.001	1	1	1.083E-06	5.829E-05	PB-CAG-SD
RNU6-563P	RNA, U6 small nuclear 563, pseudogene [Source:HGNC Symbol;Acc:47526]	chr20	46121722	46126829	19	0.002	1	1	1.282E-06	6.241E-05	PB-CAG-SD
RNF39	ring finger protein 39 [Source:HGNC Symbol;Acc:18064]	chr6	30038043	30048664	27	0.002	1	1	1.479E-06	6.241E-05	PB-CAG-SD
BCAS4	breast carcinoma amplified sequence 4 [Source:HGNC Symbol;Acc:14367]	chr20	49406431	49493714	248	0.021	2	2	1.501E-06	6.241E-05	PB-CAG-SD
ASPG	asparaginase homolog (S. cerevisiae) [Source:HGNC Symbol;Acc:20123]	chr14	104547016	104579098	21	0.002	1	1	1.690E-06	6.241E-05	PB-CAG-SD
TTC9B	tetratricopeptide repeat domain 9B [Source:HGNC Symbol;Acc:26395]	chr19	40721965	40729306	15	0.002	1	1	1.780E-06	6.241E-05	PB-CAG-SD
MIR4779	microRNA 4779 [Source:HGNC Symbol;Acc:41747]	chr2	86420149	86425231	27	0.002	1	1	1.783E-06	6.241E-05	PB-CAG-SD
RNF5	ring finger protein 5, E3 ubiquitin protein ligase [Source:HGNC Symbol;Acc:10068]	chr6	32141131	32151930	31	0.002	1	1	1.949E-06	6.241E-05	PB-CAG-SD
RNA5SP21	RNA, 5S ribosomal pseudogene 21 [Source:HGNC Symbol;Acc:42589]	chr1	78240589	78245684	29	0.002	1	1	2.018E-06	6.241E-05	PB-CAG-SD
DNAJC28	DnaJ (Hsp40) homolog, subfamily C, member 28 [Source:HGNC Symbol;Acc:1297]	chr21	34860497	34869027	38	0.002	1	1	2.095E-06	6.241E-05	PB-CAG-SD
MRPL40P1	mitochondrial ribosomal protein L40 pseudogene 1 [Source:HGNC Symbol;Acc:44532]	chr12	67740216	67745819	36	0.002	1	1	2.126E-06	6.241E-05	PB-CAG-SD
RN7SL108P	RNA, 7SL, cytoplasmic 108, pseudogene [Source:HGNC Symbol;Acc:46124]	chr14	68702962	68708256	25	0.002	1	1	2.394E-06	6.241E-05	PB-CAG-SD
SNORD45C	small nucleolar RNA, C/D box 45C [Source:HGNC Symbol;Acc:32720]	chr1	76247757	76252835	33	0.002	1	1	2.612E-06	6.241E-05	PB-CAG-SD
JUN	jun proto-oncogene [Source:HGNC Symbol;Acc:6204]	chr1	59246465	59254785	33	0.002	1	1	2.612E-06	6.241E-05	PB-CAG-SD
MIR4634	microRNA 4634 [Source:HGNC Symbol;Acc:41559]	chr5	174173737	174178790	36	0.002	1	1	2.720E-06	6.241E-05	PB-CAG-SD
MESDC1	mesoderm development candidate 1 [Source:HGNC Symbol;Acc:13519]	chr15	81288295	81296342	32	0.002	1	1	2.764E-06	6.241E-05	PB-CAG-SD
SNORD45A	small nucleolar RNA, C/D box 45A [Source:HGNC Symbol;Acc:10184]	chr1	76248574	76253657	34	0.002	1	1	2.773E-06	6.241E-05	PB-CAG-SD
GAPDHP29	glyceraldehyde 3 phosphate dehydrogenase pseudogene 29 [Source:HGNC Symbol;Acc:37781]	chr1	94762594	94768578	34	0.002	1	1	2.773E-06	6.241E-05	PB-CAG-SD
TRAJ17	T cell receptor alpha joining 17 [Source:HGNC Symbol;Acc:12045]	chr14	22990812	22995874	28	0.002	1	1	3.003E-06	6.241E-05	PB-CAG-SD
CCL24	chemokine (C-C motif) ligand 24 [Source:HGNC Symbol;Acc:10623]	chr7	75440983	75457674	40	0.003	1	1	3.154E-06	6.241E-05	PB-CAG-SD
PRR15L	proline rich 15-like [Source:HGNC Symbol;Acc:28149]	chr17	46029333	46040244	29	0.003	1	1	3.275E-06	6.241E-05	PB-CAG-SD
ACR	acrosin [Source:HGNC Symbol;Acc:126]	chr22	51171624	51183762	28	0.003	1	1	3.275E-06	6.241E-05	PB-CAG-SD
RNA5SP362	RNA, 5S ribosomal pseudogene 362 [Source:HGNC Symbol;Acc:43262]	chr12	66455001	66460118	45	0.003	1	1	3.320E-06	6.241E-05	PB-CAG-SD
UBAC2-AS1	UBAC2 antisense RNA 1 [Source:HGNC Symbol;Acc:42502]	chr13	99848631	99857964	47	0.003	1	1	3.372E-06	6.241E-05	PB-CAG-SD
AGPAT1	1-acylglycerol-3-phosphate O-acyltransferase 1 [Source:HGNC Symbol;Acc:324]	chr6	32135989	32150873	41	0.003	1	1	3.408E-06	6.241E-05	PB-CAG-SD
TRAJ15	T cell receptor alpha joining 15 [Source:HGNC Symbol;Acc:12043]	chr14	22993580	22998641	30	0.003	1	1	3.447E-06	6.241E-05	PB-CAG-SD
HOXA1	homeobox A1 [Source:HGNC Symbol;Acc:5099]	chr7	27132612	27140615	42	0.003	1	1	3.477E-06	6.241E-05	PB-CAG-SD
RNU7-163P	RNA, U7 small nuclear 163 pseudogene [Source:HGNC Symbol;Acc:45697]	chr10	1088233	1093295	33	0.003	1	1	3.662E-06	6.283E-05	PB-CAG-SD
TRAJ16	T cell receptor alpha joining 16 [Source:HGNC Symbol;Acc:12044]	chr14	22992487	22997546	31	0.003	1	1	3.680E-06	6.283E-05	PB-CAG-SD
TRAJ14	T cell receptor alpha joining 14 [Source:HGNC Symbol;Acc:12042]	chr14	22994278	22999329	32	0.003	1	1	3.921E-06	6.535E-05	PB-CAG-SD
VPS18	vacuolar protein sorting 18 homolog (S. cerevisiae) [Source:HGNC Symbol;Acc:15972]	chr15	41181628	41196173	39	0.003	1	1	4.104E-06	6.680E-05	PB-CAG-SD
SNORD45B	small nucleolar RNA, C/D box 45B [Source:HGNC Symbol;Acc:10185]	chr1	76250162	76255233	43	0.003	1	1	4.433E-06	6.877E-05	PB-CAG-SD
SHCBP1	SHC SH2-domain binding protein 1 [Source:HGNC Symbol;Acc:29547]	chr16	46614466	46660538	315	0.030	2	2	4.514E-06	6.877E-05	PB-CAG-SD
RPEP5	ribulose-5-phosphate-3-epimerase pseudogene 5 [Source:HGNC Symbol;Acc:44524]	chr2	162787709	162792976	43	0.003	1	1	4.519E-06	6.877E-05	PB-CAG-SD
DLGAP1-AS2	DLGAP1 antisense RNA 2 [Source:HGNC Symbol;Acc:28146]	chr18	3597998	3608334	48	0.003	1	1	4.930E-06	7.217E-05	PB-CAG-SD
MYEOV2	myeloma overexpressed 2 [Source:HGNC Symbol;Acc:21314]	chr2	241065980	241081224	45	0.003	1	1	4.949E-06	7.217E-05	PB-CAG-SD
TRAJ13	T cell receptor alpha joining 13 [Source:HGNC Symbol;Acc:12041]	chr14	22995026	23000088	37	0.003	1	1	5.241E-06	7.487E-05	PB-CAG-SD
TRAF3IP1	TNF receptor-associated factor 3 interacting protein 1 [Source:HGNC Symbol;Acc:17861]	chr2	239224082	239309541	467	0.033	2	1	5.678E-06	7.949E-05	PB-CAG-SD
HOTAIRM1	HOXA transcript antisense RNA, myeloid-specific 1 [Source:HGNC Symbol;Acc:37117]	chr7	27130266	27139884	56	0.004	1	1	6.178E-06	8.479E-05	PB-CAG-SD
KLF10	Kruppel-like factor 10 [Source:HGNC Symbol;Acc:11810]	chr8	103661007	103673130	48	0.004	1	1	6.428E-06	8.559E-05	PB-CAG-SD
KRT18P32	keratin 18 pseudogene 32 [Source:HGNC Symbol;Acc:33401]	chr1	238649658	238655944	52	0.004	1	1	6.481E-06	8.559E-05	PB-CAG-SD
SMCO1	single-pass membrane protein with coiled-coil domains 1 [Source:HGNC Symbol;Acc:27407]	chr3	196233748	196247237	59	0.004	1	1	7.923E-06	1.027E-04	PB-CAG-SD
CUX1	cut-like homeobox 1 [Source:HGNC Symbol;Acc:2557]	chr7	101453959	101927249	1947	0.122	3	2	8.469E-06	1.061E-04	PB-CAG-SD
LINC00159	long intergenic non-protein coding RNA 159 [Source:HGNC Symbol;Acc:1285]	chr21	33452629	33575125	694	0.037	2	1	8.486E-06	1.061E-04	PB-CAG-SD
FGD2	FYVE, RhoGEF and PH domain containing 2 [Source:HGNC Symbol;Acc:3664]	chr6	36968422	36996846	66	0.004	1	1	8.822E-06	1.083E-04	PB-CAG-SD
ZNF861P	zinc finger protein 861, pseudogene [Source:HGNC Symbol;Acc:34514]	chr19	15932607	15939614	34	0.004	1	1	9.131E-06	1.093E-04	PB-CAG-SD
PPP1R26-AS1	PPP1R26 antisense RNA 1 [Source:HGNC Symbol;Acc:48717]	chr9	138354573	138377623	73	0.004	1	1	9.213E-06	1.093E-04	PB-CAG-SD
RAD21-AS1	RAD21 antisense RNA 1 [Source:HGNC Symbol;Acc:32158]	chr8	117881663	117889107	62	0.005	1	1	1.072E-05	1.250E-04	PB-CAG-SD
MYC	v-myc avian myelocytomatosis viral oncogene homolog [Source:HGNC Symbol;Acc:7553]	chr8	128742680	128753674	63	0.005	1	1	1.106E-05	1.250E-04	PB-CAG-SD
FAM200A	family with sequence similarity 200, member A [Source:HGNC Symbol;Acc:25401]	chr7	99143931	99161159	75	0.005	1	1	1.107E-05	1.250E-04	PB-CAG-SD
TXLNA	taxilin alpha [Source:HGNC Symbol;Acc:30685]	chr1	32640287	32663886	70	0.005	1	1	1.173E-05	1.291E-04	PB-CAG-SD
ZNF182	zinc finger protein 182 [Source:HGNC Symbol;Acc:13001]	chrX	47834250	47868377	191	0.005	1	1	1.181E-05	1.291E-04	PB-CAG-SD
LMOD2	leiomodin 2 (cardiac) [Source:HGNC Symbol;Acc:6648]	chr7	123290861	123304344	79	0.005	1	1	1.228E-05	1.323E-04	PB-CAG-SD
BCL7B	B-cell CLL/lymphoma 7B [Source:HGNC Symbol;Acc:1005]	chr7	72950686	72977332	80	0.005	1	1	1.259E-05	1.332E-04	PB-CAG-SD
RMND5B	required for meiotic nuclear division 5 homolog B (S. cerevisiae) [Source:HGNC Symbol;Acc:26181]	chr5	177552997	177577566	78	0.005	1	1	1.274E-05	1.332E-04	PB-CAG-SD
FCAMR	Fc receptor, IgA, IgM, high affinity [Source:HGNC Symbol;Acc:24692]	chr1	207131310	207148970	74	0.005	1	1	1.311E-05	1.350E-04	PB-CAG-SD
LNX1-AS2	LNX1 antisense RNA 2 [Source:HGNC Symbol;Acc:41450]	chr4	54454123	54470214	92	0.005	1	1	1.392E-05	1.412E-04	PB-CAG-SD
KIAA0087	KIAA0087 [Source:HGNC Symbol;Acc:22191]	chr7	26572740	26583407	87	0.005	1	1	1.489E-05	1.433E-04	PB-CAG-SD
ARHGAP31-AS1	ARHGAP31 antisense RNA 1 [Source:HGNC Symbol;Acc:41235]	chr3	119033140	119046607	81	0.005	1	1	1.492E-05	1.433E-04	PB-CAG-SD
ZNF597	zinc finger protein 597 [Source:HGNC Symbol;Acc:26573]	chr16	3486104	3498542	57	0.005	1	1	1.494E-05	1.433E-04	PB-CAG-SD
FBLIM1	filamin binding LIM protein 1 [Source:HGNC Symbol;Acc:24686]	chr1	16078102	16113089	79	0.005	1	1	1.494E-05	1.433E-04	PB-CAG-SD
LINC00626	long intergenic non-protein coding RNA 626 [Source:HGNC Symbol;Acc:44257]	chr1	168751177	168762124	82	0.006	1	1	1.609E-05	1.522E-04	PB-CAG-SD
GABARAPL1	GABA(A) receptor-associated protein like 1 [Source:HGNC Symbol;Acc:4068]	chr12	10360057	10375727	100	0.006	1	1	1.636E-05	1.527E-04	PB-CAG-SD
ZNF552	zinc finger protein 552 [Source:HGNC Symbol;Acc:26135]	chr19	58315209	58331281	47	0.006	1	1	1.743E-05	1.591E-04	PB-CAG-SD
BCORP1	BCL6 corepressor pseudogene 1 [Source:HGNC Symbol;Acc:23953]	chrY	21617317	21670039	323	0.006	1	1	1.750E-05	1.591E-04	PB-CAG-SD
SYT12	synaptotagmin XII [Source:HGNC Symbol;Acc:18381]	chr11	66769249	66818334	107	0.006	1	1	1.920E-05	1.723E-04	PB-CAG-SD
THRB-IT1	THRB intronic transcript 1 (non-protein coding) [Source:HGNC Symbol;Acc:41390]	chr3	24496627	24505925	94	0.006	1	1	2.008E-05	1.779E-04	PB-CAG-SD
UNC50	unc-50 homolog (C. elegans) [Source:HGNC Symbol;Acc:16046]	chr2	99220042	99234978	95	0.007	1	1	2.200E-05	1.909E-04	PB-CAG-SD
UPP1	uridine phosphorylase 1 [Source:HGNC Symbol;Acc:12576]	chr7	48123225	48148330	106	0.007	1	1	2.209E-05	1.909E-04	PB-CAG-SD
OSTCP1	oligosaccharyltransferase complex subunit pseudogene 1 [Source:HGNC Symbol;Acc:30530]	chr6	159262149	159283664	107	0.007	1	1	2.315E-05	1.976E-04	PB-CAG-SD
GIMD1	GIMAP family P-loop NTPase domain containing 1 [Source:HGNC Symbol;Acc:44141]	chr4	107279555	107293359	120	0.007	1	1	2.365E-05	1.984E-04	PB-CAG-SD
ALG10	ALG10, alpha-1,2-glucosyltransferase [Source:HGNC Symbol;Acc:23162]	chr12	34170216	34182629	121	0.007	1	1	2.394E-05	1.984E-04	PB-CAG-SD
DLG3	discs, large homolog 3 (Drosophila) [Source:HGNC Symbol;Acc:2902]	chrX	69659711	69725337	273	0.007	1	1	2.409E-05	1.984E-04	PB-CAG-SD
IFIT5	interferon-induced protein with tetratricopeptide repeats 5 [Source:HGNC Symbol;Acc:13328]	chr10	91169343	91180758	88	0.007	1	1	2.596E-05	2.083E-04	PB-CAG-SD
VSIG1	V-set and immunoglobulin domain containing 1 [Source:HGNC Symbol;Acc:28675]	chrX	107283200	107322414	290	0.007	1	1	2.717E-05	2.083E-04	PB-CAG-SD
TJAP1	tight junction associated protein 1 (peripheral) [Source:HGNC Symbol;Acc:17949]	chr6	43440261	43474294	116	0.007	1	1	2.720E-05	2.083E-04	PB-CAG-SD
COQ9	coenzyme Q9 homolog (S. cerevisiae) [Source:HGNC Symbol;Acc:25302]	chr16	57476337	57495187	77	0.007	1	1	2.722E-05	2.083E-04	PB-CAG-SD
TM2D2	TM2 domain containing 2 [Source:HGNC Symbol;Acc:24127]	chr8	38846327	38859343	99	0.007	1	1	2.727E-05	2.083E-04	PB-CAG-SD
CCAT1	colon cancer associated transcript 1 (non-protein coding) [Source:HGNC Symbol;Acc:45128]	chr8	128220111	128236333	99	0.007	1	1	2.727E-05	2.083E-04	PB-CAG-SD
RABGGTB	Rab geranylgeranyltransferase, beta subunit [Source:HGNC Symbol;Acc:9796]	chr1	76246879	76260764	107	0.007	1	1	2.737E-05	2.083E-04	PB-CAG-SD
SFR1	SWI5-dependent recombination repair 1 [Source:HGNC Symbol;Acc:29574]	chr10	105876816	105886143	94	0.008	1	1	2.961E-05	2.229E-04	PB-CAG-SD
CHTF8	CTF8, chromosome transmission fidelity factor 8 homolog (S. cerevisiae) [Source:HGNC Symbol;Acc:24353]	chr16	69151913	69171487	81	0.008	1	1	3.012E-05	2.243E-04	PB-CAG-SD
SRSF3	serine/arginine-rich splicing factor 3 [Source:HGNC Symbol;Acc:10785]	chr6	36557145	36573377	126	0.008	1	1	3.207E-05	2.363E-04	PB-CAG-SD
UCA1	urothelial cancer associated 1 (non-protein coding) [Source:HGNC Symbol;Acc:37126]	chr19	15934757	15947130	65	0.008	1	1	3.329E-05	2.427E-04	PB-CAG-SD
PSMD9	proteasome (prosome, macropain) 26S subunit, non-ATPase, 9 [Source:HGNC Symbol;Acc:9567]	chr12	122321637	122356203	145	0.008	1	1	3.434E-05	2.473E-04	PB-CAG-SD
PROL1	proline rich, lacrimal 1 [Source:HGNC Symbol;Acc:17279]	chr4	71258599	71275911	146	0.008	1	1	3.498E-05	2.473E-04	PB-CAG-SD
IBSP	integrin-binding sialoprotein [Source:HGNC Symbol;Acc:5341]	chr4	88715733	88733074	146	0.008	1	1	3.498E-05	2.473E-04	PB-CAG-SD
CYCTP	cytochrome c, testis, pseudogene [Source:HGNC Symbol;Acc:24424]	chr2	178957230	178974197	123	0.009	1	1	3.684E-05	2.558E-04	PB-CAG-SD
AGA	aspartylglucosaminidase [Source:HGNC Symbol;Acc:318]	chr4	178351924	178368657	150	0.009	1	1	3.691E-05	2.558E-04	PB-CAG-SD
PAWR	PRKC, apoptosis, WT1, regulator [Source:HGNC Symbol;Acc:8614]	chr12	79968759	80089877	1085	0.062	2	2	3.826E-05	2.625E-04	PB-CAG-SD
IDI1	isopentenyl-diphosphate delta isomerase 1 [Source:HGNC Symbol;Acc:5387]	chr10	1085848	1100110	111	0.009	1	1	4.126E-05	2.797E-04	PB-CAG-SD
GNAZ	guanine nucleotide binding protein (G protein), alpha z polypeptide [Source:HGNC Symbol;Acc:4395]	chr22	23407540	23467224	100	0.009	1	1	4.159E-05	2.797E-04	PB-CAG-SD
HNRNPL	heterogeneous nuclear ribonucleoprotein L [Source:HGNC Symbol;Acc:5045]	chr19	39327028	39347987	73	0.009	1	1	4.195E-05	2.797E-04	PB-CAG-SD
CNBP	CCHC-type zinc finger, nucleic acid binding protein [Source:HGNC Symbol;Acc:13164]	chr3	128888327	128907765	139	0.009	1	1	4.382E-05	2.890E-04	PB-CAG-SD
CCDC94	coiled-coil domain containing 94 [Source:HGNC Symbol;Acc:25518]	chr19	4242076	4269087	75	0.009	1	1	4.428E-05	2.890E-04	PB-CAG-SD
BICD2	bicaudal D homolog 2 (Drosophila) [Source:HGNC Symbol;Acc:17208]	chr9	95473645	95532094	161	0.009	1	1	4.466E-05	2.890E-04	PB-CAG-SD
MRPL35	mitochondrial ribosomal protein L35 [Source:HGNC Symbol;Acc:14489]	chr2	86421478	86440917	136	0.010	1	1	4.501E-05	2.890E-04	PB-CAG-SD
ANAPC7	anaphase promoting complex subunit 7 [Source:HGNC Symbol;Acc:17380]	chr12	110810705	110846535	172	0.010	1	1	4.827E-05	3.065E-04	PB-CAG-SD
PFKFB1	6-phosphofructo-2-kinase/fructose-2,6-biphosphatase 1 [Source:HGNC Symbol;Acc:8872]	chrX	54959394	55029967	389	0.010	1	1	4.881E-05	3.065E-04	PB-CAG-SD
HMGA2	high mobility group AT-hook 2 [Source:HGNC Symbol;Acc:5009]	chr12	66212911	66360075	1182	0.068	2	1	4.926E-05	3.065E-04	PB-CAG-SD
LIG3	ligase III, DNA, ATP-dependent [Source:HGNC Symbol;Acc:6600]	chr17	33302513	33332083	113	0.010	1	1	4.947E-05	3.065E-04	PB-CAG-SD
C20orf96	chromosome 20 open reading frame 96 [Source:HGNC Symbol;Acc:16227]	chr20	251504	276390	120	0.010	1	1	5.087E-05	3.123E-04	PB-CAG-SD
ASCC1	activating signal cointegrator 1 complex subunit 1 [Source:HGNC Symbol;Acc:24268]	chr10	73856278	73981892	839	0.069	2	1	5.170E-05	3.147E-04	PB-CAG-SD
HHIPL2	HHIP-like 2 [Source:HGNC Symbol;Acc:25842]	chr1	222695602	222726445	149	0.010	1	1	5.297E-05	3.187E-04	PB-CAG-SD
STEAP3	STEAP family member 3, metalloreductase [Source:HGNC Symbol;Acc:24592]	chr2	119976384	120023228	148	0.010	1	1	5.327E-05	3.187E-04	PB-CAG-SD
SPAG11A	sperm associated antigen 11A [Source:HGNC Symbol;Acc:33342]	chr8	7700398	7726389	141	0.011	1	1	5.521E-05	3.234E-04	PB-CAG-SD
CCT8	chaperonin containing TCP1, subunit 8 (theta) [Source:HGNC Symbol;Acc:1623]	chr21	30428126	30451118	196	0.011	1	1	5.543E-05	3.234E-04	PB-CAG-SD
GPRC5B	G protein-coupled receptor, family C, group 5, member B [Source:HGNC Symbol;Acc:13308]	chr16	19868616	19902489	110	0.011	1	1	5.544E-05	3.234E-04	PB-CAG-SD
SPAG11B	sperm associated antigen 11B [Source:HGNC Symbol;Acc:14534]	chr8	7300206	7326196	142	0.011	1	1	5.599E-05	3.239E-04	PB-CAG-SD
FHL1	four and a half LIM domains 1 [Source:HGNC Symbol;Acc:3702]	chrX	135224559	135293518	423	0.011	1	1	5.768E-05	3.309E-04	PB-CAG-SD
SLC36A3	solute carrier family 36, member 3 [Source:HGNC Symbol;Acc:19659]	chr5	150656323	150688327	167	0.011	1	1	5.820E-05	3.312E-04	PB-CAG-SD

table S1. Genes with significant transposon integrations in DLD-1 *KRAS*$^{wt/-}$ cells after selection in low glucose medium.

Gene ID	Description	Chrom	Start	End	# TTAA sites	Expected	Observed	Libraries	*P*	FDR	Transposon
PLIN2	perilipin 2 [Source:HGNC Symbol;Acc:248]	chr9	19108373	19154288	187	0.011	1	1	6.019E-05	3.390E-04	PB-CAG-SD
SKAP1	src kinase associated phosphoprotein 1 [Source:HGNC Symbol;Acc:15605]	chr17	46210802	46512637	2302	0.203	3	1	6.054E-05	3.390E-04	PB-CAG-SD
ART4	ADP-ribosyltransferase 4 (Dombrock blood group) [Source:HGNC Symbol;Acc:726]	chr12	14978503	15001429	195	0.011	1	1	6.199E-05	3.444E-04	PB-CAG-SD
SOX13	SRY (sex determining region Y)-box 13 [Source:HGNC Symbol;Acc:11192]	chr1	204037243	204096863	166	0.012	1	1	6.570E-05	3.606E-04	PB-CAG-SD
CIAPIN1	cytokine induced apoptosis inhibitor 1 [Source:HGNC Symbol;Acc:28050]	chr16	57462081	57486440	120	0.012	1	1	6.593E-05	3.606E-04	PB-CAG-SD
CDC37	cell division cycle 37 [Source:HGNC Symbol;Acc:1735]	chr19	10501810	10535797	92	0.012	1	1	6.653E-05	3.610E-04	PB-CAG-SD
MSMO1	methylsterol monooxygenase 1 [Source:HGNC Symbol;Acc:10545]	chr4	166243775	166264312	211	0.012	1	1	7.287E-05	3.924E-04	PB-CAG-SD
ZNF630	zinc finger protein 630 [Source:HGNC Symbol;Acc:28855]	chrX	47842756	47936025	484	0.012	1	1	7.543E-05	4.031E-04	PB-CAG-SD
SF3A3	splicing factor 3a, subunit 3, 60kDa [Source:HGNC Symbol;Acc:10767]	chr1	38422647	38461593	179	0.012	1	1	7.634E-05	4.031E-04	PB-CAG-SD
ENPP4	ectonucleotide pyrophosphatase/phosphodiesterase 4 (putative) [Source:HGNC Symbol;Acc:3359]	chr6	46092730	46114436	195	0.012	1	1	7.659E-05	4.031E-04	PB-CAG-SD
PODXL2	podocalyxin-like 2 [Source:HGNC Symbol;Acc:17936]	chr3	127343024	127391652	189	0.013	1	1	8.083E-05	4.223E-04	PB-CAG-SD
COLEC11	collectin sub-family member 11 [Source:HGNC Symbol;Acc:17213]	chr2	3637426	3692048	184	0.013	1	1	8.220E-05	4.249E-04	PB-CAG-SD
POPDC2	popeye domain containing 2 [Source:HGNC Symbol;Acc:17648]	chr3	119355304	119389171	191	0.013	1	1	8.255E-05	4.249E-04	PB-CAG-SD
ASAP3	ArfGAP with SH3 domain, ankyrin repeat and PH domain 3 [Source:HGNC Symbol;Acc:14987]	chr1	23755056	23815685	187	0.013	1	1	8.329E-05	4.256E-04	PB-CAG-SD
CRHBP	corticotropin releasing hormone binding protein [Source:HGNC Symbol;Acc:2356]	chr5	76243538	76276983	203	0.013	1	1	8.586E-05	4.355E-04	PB-CAG-SD
ERRFI1	ERBB receptor feedback inhibitor 1 [Source:HGNC Symbol;Acc:18185]	chr1	8064464	8091368	191	0.013	1	1	8.687E-05	4.375E-04	PB-CAG-SD
STARD4	StAR-related lipid transfer (START) domain containing 4 [Source:HGNC Symbol;Acc:18058]	chr5	110831731	110853288	212	0.014	1	1	9.360E-05	4.659E-04	PB-CAG-SD
WIPI2	WD repeat domain, phosphoinositide interacting 2 [Source:HGNC Symbol;Acc:32225]	chr7	5224819	5273457	219	0.014	1	1	9.384E-05	4.659E-04	PB-CAG-SD
LINC00710	long intergenic non-protein coding RNA 710 [Source:HGNC Symbol;Acc:27386]	chr10	10969021	10999143	171	0.014	1	1	9.759E-05	4.798E-04	PB-CAG-SD
MDH1B	malate dehydrogenase 1B, NAD (soluble) [Source:HGNC Symbol;Acc:17836]	chr2	207602487	207635271	201	0.014	1	1	9.802E-05	4.798E-04	PB-CAG-SD
C6orf99	chromosome 6 open reading frame 99 [Source:HGNC Symbol;Acc:21179]	chr6	159285954	159331385	224	0.014	1	1	1.009E-04	4.869E-04	PB-CAG-SD
ABCC3	ATP-binding cassette, sub-family C (CFTR/MRP), member 3 [Source:HGNC Symbol;Acc:54]	chr17	48707138	48769613	162	0.014	1	1	1.014E-04	4.869E-04	PB-CAG-SD
FER1L5	fer-1-like 5 (C. elegans) [Source:HGNC Symbol;Acc:19044]	chr2	97303474	97370624	205	0.014	1	1	1.019E-04	4.869E-04	PB-CAG-SD
CDKN2A	cyclin-dependent kinase inhibitor 2A [Source:HGNC Symbol;Acc:1787]	chr9	21967751	22000300	244	0.014	1	1	1.022E-04	4.869E-04	PB-CAG-SD
KCTD1	potassium channel tetramerization domain containing 1 [Source:HGNC Symbol;Acc:18249]	chr18	24034874	24242365	1334	0.087	2	1	1.041E-04	4.923E-04	PB-CAG-SD
GPR113	G protein-coupled receptor 113 [Source:HGNC Symbol;Acc:18989]	chr2	26531041	26574685	208	0.015	1	1	1.049E-04	4.929E-04	PB-CAG-SD
NUB1	negative regulator of ubiquitin-like proteins 1 [Source:HGNC Symbol;Acc:17623]	chr7	151033785	151075535	238	0.015	1	1	1.107E-04	5.168E-04	PB-CAG-SD
SLC19A2	solute carrier family 19 (thiamine transporter), member 2 [Source:HGNC Symbol;Acc:10938]	chr1	169433147	169460241	218	0.015	1	1	1.130E-04	5.240E-04	PB-CAG-SD
IL36B	interleukin 36, beta [Source:HGNC Symbol;Acc:15564]	chr2	113779668	113815444	219	0.015	1	1	1.163E-04	5.354E-04	PB-CAG-SD
IDI2-AS1	IDI2 antisense RNA 1 [Source:HGNC Symbol;Acc:30885]	chr10	1063606	1090138	188	0.015	1	1	1.179E-04	5.391E-04	PB-CAG-SD
SUGP1	SURP and G patch domain containing 1 [Source:HGNC Symbol;Acc:18643]	chr19	19386827	19437123	123	0.015	1	1	1.186E-04	5.391E-04	PB-CAG-SD
SPDL1	spindle apparatus coiled-coil protein 1 [Source:HGNC Symbol;Acc:26010]	chr5	169005638	169031782	241	0.016	1	1	1.208E-04	5.456E-04	PB-CAG-SD
VEZF1	vascular endothelial zinc finger 1 [Source:HGNC Symbol;Acc:12949]	chr17	56048910	56070620	178	0.016	1	1	1.223E-04	5.487E-04	PB-CAG-SD
MFSD2B	major facilitator superfamily domain containing 2B [Source:HGNC Symbol;Acc:37207]	chr2	24227951	24286191	230	0.016	1	1	1.282E-04	5.714E-04	PB-CAG-SD
CYB5D2	cytochrome b5 domain containing 2 [Source:HGNC Symbol;Acc:28471]	chr17	4041462	4090605	187	0.017	1	1	1.349E-04	5.944E-04	PB-CAG-SD
NAA60	N(alpha)-acetyltransferase 60, NatF catalytic subunit [Source:HGNC Symbol;Acc:25875]	chr16	3488611	3536963	172	0.017	1	1	1.350E-04	5.944E-04	PB-CAG-SD
RTDR1	rhabdoid tumor deletion region gene 1 [Source:HGNC Symbol;Acc:13437]	chr22	23401593	23492208	184	0.017	1	1	1.401E-04	6.128E-04	PB-CAG-SD
MAPKBP1	mitogen-activated protein kinase binding protein 1 [Source:HGNC Symbol;Acc:29536]	chr15	42061632	42120053	231	0.017	1	1	1.426E-04	6.168E-04	PB-CAG-SD
CEACAM7	carcinoembryonic antigen-related cell adhesion molecule 7 [Source:HGNC Symbol;Acc:1819]	chr19	42177235	42215895	135	0.017	1	1	1.427E-04	6.168E-04	PB-CAG-SD
RNF168	ring finger protein 168, E3 ubiquitin protein ligase [Source:HGNC Symbol;Acc:26661]	chr3	196195654	196235639	259	0.017	1	1	1.513E-04	6.498E-04	PB-CAG-SD
RUFY1	RUN and FYVE domain containing 1 [Source:HGNC Symbol;Acc:19760]	chr5	178972559	179037027	271	0.018	1	1	1.526E-04	6.512E-04	PB-CAG-SD
ABCC8	ATP-binding cassette, sub-family C (CFTR/MRP), member 8 [Source:HGNC Symbol;Acc:59]	chr11	17414432	17503449	304	0.018	1	1	1.538E-04	6.525E-04	PB-CAG-SD
TEAD1	TEA domain family member 1 (SV40 transcriptional enhancer factor) [Source:HGNC Symbol;Acc:11714]	chr11	12690969	12966298	1728	0.100	2	2	1.559E-04	6.539E-04	PB-CAG-SD
RBMS3-AS3	RBMS3 antisense RNA 3 [Source:HGNC Symbol;Acc:39989]	chr3	29305685	29337217	263	0.018	1	1	1.560E-04	6.539E-04	PB-CAG-SD
KCNC4	potassium voltage-gated channel, Shaw-related subfamily, member 4 [Source:HGNC Symbol;Acc:6236]	chr1	110748965	110825722	260	0.018	1	1	1.605E-04	6.686E-04	PB-CAG-SD
C11orf44	chromosome 11 open reading frame 44 [Source:HGNC Symbol;Acc:26805]	chr11	130537851	130587247	312	0.018	1	1	1.620E-04	6.709E-04	PB-CAG-SD
SGOL1	shugoshin-like 1 (S. pombe) [Source:HGNC Symbol;Acc:25088]	chr3	20202085	20232784	270	0.018	1	1	1.644E-04	6.768E-04	PB-CAG-SD
THEM4	thioesterase superfamily member 4 [Source:HGNC Symbol;Acc:17947]	chr1	151846060	151887284	268	0.019	1	1	1.704E-04	6.974E-04	PB-CAG-SD
LYPD5	LY6/PLAUR domain containing 5 [Source:HGNC Symbol;Acc:26397]	chr19	44300081	44336358	148	0.019	1	1	1.714E-04	6.974E-04	PB-CAG-SD
FAT4	FAT atypical cadherin 4 [Source:HGNC Symbol;Acc:23109]	chr4	126232554	126414087	1819	0.104	2	2	1.759E-04	7.113E-04	PB-CAG-SD
TSEN15	tRNA splicing endonuclease 15 homolog (S. cerevisiae) [Source:HGNC Symbol;Acc:16791]	chr1	184015811	184043346	273	0.019	1	1	1.768E-04	7.113E-04	PB-CAG-SD
NMU	neuromedin U [Source:HGNC Symbol;Acc:7859]	chr4	56461396	56507865	333	0.019	1	1	1.807E-04	7.220E-04	PB-CAG-SD
RBM45	RNA binding motif protein 45 [Source:HGNC Symbol;Acc:24468]	chr2	178972151	179001531	274	0.019	1	1	1.815E-04	7.220E-04	PB-CAG-SD
MYO16-AS1	MYO16 antisense RNA 1 [Source:HGNC Symbol;Acc:39913]	chr13	109816250	109858831	353	0.020	1	1	1.881E-04	7.387E-04	PB-CAG-SD
USP42	ubiquitin specific peptidase 42 [Source:HGNC Symbol;Acc:20068]	chr7	6139515	6201195	312	0.020	1	1	1.897E-04	7.387E-04	PB-CAG-SD
APH1B	APH1B gamma secretase subunit [Source:HGNC Symbol;Acc:24080]	chr15	63563217	63601325	267	0.020	1	1	1.902E-04	7.387E-04	PB-CAG-SD
TMEM110	transmembrane protein 110 [Source:HGNC Symbol;Acc:30526]	chr3	52870235	52936612	291	0.020	1	1	1.907E-04	7.387E-04	PB-CAG-SD
GCNT3	glucosaminyl (N-acetyl) transferase 3, mucin type [Source:HGNC Symbol;Acc:4205]	chr15	59882074	59932438	268	0.020	1	1	1.916E-04	7.387E-04	PB-CAG-SD
TMEM110-MUSTN1	TMEM110-MUSTN1 readthrough [Source:HGNC Symbol;Acc:38834]	chr3	52867137	52936578	292	0.020	1	1	1.921E-04	7.387E-04	PB-CAG-SD
ANKRD10	ankyrin repeat domain 10 [Source:HGNC Symbol;Acc:20265]	chr13	111530887	111572416	359	0.020	1	1	1.945E-04	7.422E-04	PB-CAG-SD
ACIN1	apoptotic chromatin condensation inducer 1 [Source:HGNC Symbol;Acc:17066]	chr14	23527773	23569823	227	0.020	1	1	1.951E-04	7.422E-04	PB-CAG-SD
ABCA3	ATP-binding cassette, sub-family A (ABC1), member 3 [Source:HGNC Symbol;Acc:33]	chr16	2325882	2395747	208	0.020	1	1	1.970E-04	7.427E-04	PB-CAG-SD
FAN1	FANCD2/FANCI-associated nuclease 1 [Source:HGNC Symbol;Acc:29170]	chr15	31191055	31235311	272	0.020	1	1	1.973E-04	7.427E-04	PB-CAG-SD
FBXO3	F-box protein 3 [Source:HGNC Symbol;Acc:13582]	chr11	33762485	33801089	346	0.020	1	1	1.989E-04	7.446E-04	PB-CAG-SD
SNAPC3	small nuclear RNA activating complex, polypeptide 3, 50kDa [Source:HGNC Symbol;Acc:11136]	chr9	15417702	15465951	350	0.021	1	1	2.095E-04	7.801E-04	PB-CAG-SD
ZNF138	zinc finger protein 138 [Source:HGNC Symbol;Acc:12922]	chr7	64249766	64294054	329	0.021	1	1	2.108E-04	7.808E-04	PB-CAG-SD
BTLA	B and T lymphocyte associated [Source:HGNC Symbol;Acc:21087]	chr3	112182815	112223408	307	0.021	1	1	2.121E-04	7.814E-04	PB-CAG-SD
GPR64	G protein-coupled receptor 64 [Source:HGNC Symbol;Acc:4516]	chrX	19007427	19145755	816	0.021	1	1	2.132E-04	7.814E-04	PB-CAG-SD
SLC37A1	solute carrier family 37 (glucose-6-phosphate transporter), member 1 [Source:HGNC Symbol;Acc:11024]	chr21	43911128	44001550	392	0.021	1	1	2.202E-04	8.027E-04	PB-CAG-SD
RNF11	ring finger protein 11 [Source:HGNC Symbol;Acc:10056]	chr1	51696943	51739127	307	0.021	1	1	2.232E-04	8.097E-04	PB-CAG-SD
MMP20	matrix metallopeptidase 20 [Source:HGNC Symbol;Acc:7167]	chr11	102447566	102501063	369	0.021	1	1	2.260E-04	8.156E-04	PB-CAG-SD
C14orf105	chromosome 14 open reading frame 105 [Source:HGNC Symbol;Acc:20189]	chr14	57936019	57965585	246	0.022	1	1	2.289E-04	8.179E-04	PB-CAG-SD
ADCY3	adenylate cyclase 3 [Source:HGNC Symbol;Acc:234]	chr2	25042038	25147708	308	0.022	1	1	2.290E-04	8.179E-04	PB-CAG-SD
ACOT7	acyl-CoA thioesterase 7 [Source:HGNC Symbol;Acc:24157]	chr1	6324333	6459451	313	0.022	1	1	2.320E-04	8.243E-04	PB-CAG-SD
RNMT	RNA (guanine-7-) methyltransferase [Source:HGNC Symbol;Acc:10075]	chr18	13721659	13764557	334	0.022	1	1	2.358E-04	8.335E-04	PB-CAG-SD
UGT3A1	UDP glycosyltransferase 3 family, polypeptide A1 [Source:HGNC Symbol;Acc:26625]	chr5	35951112	36006130	341	0.022	1	1	2.408E-04	8.462E-04	PB-CAG-SD
ZNF347	zinc finger protein 347 [Source:HGNC Symbol;Acc:16447]	chr19	53627325	53667328	176	0.022	1	1	2.418E-04	8.462E-04	PB-CAG-SD
ZNF710	zinc finger protein 710 [Source:HGNC Symbol;Acc:25352]	chr15	90539624	90625438	304	0.022	1	1	2.461E-04	8.501E-04	PB-CAG-SD
SENP2	SUMO1/sentrin/SMT3 specific peptidase 2 [Source:HGNC Symbol;Acc:23116]	chr3	185295284	185351339	331	0.022	1	1	2.464E-04	8.501E-04	PB-CAG-SD
DEK	DEK oncogene [Source:HGNC Symbol;Acc:2768]	chr6	18224099	18270054	351	0.022	1	1	2.465E-04	8.501E-04	PB-CAG-SD
MED26	mediator complex subunit 26 [Source:HGNC Symbol;Acc:2376]	chr19	16685718	16744873	179	0.023	1	1	2.500E-04	8.579E-04	PB-CAG-SD
PPP2CB	protein phosphatase 2, catalytic subunit, beta isozyme [Source:HGNC Symbol;Acc:9300]	chr8	30631973	30676830	302	0.023	1	1	2.512E-04	8.579E-04	PB-CAG-SD
WDR93	WD repeat domain 93 [Source:HGNC Symbol;Acc:26924]	chr15	90229028	90286869	309	0.023	1	1	2.542E-04	8.639E-04	PB-CAG-SD
ZNF283	zinc finger protein 283 [Source:HGNC Symbol;Acc:13077]	chr19	44326444	44353307	181	0.023	1	1	2.556E-04	8.644E-04	PB-CAG-SD
CDCP1	CUB domain containing protein 1 [Source:HGNC Symbol;Acc:24357]	chr3	45123770	45192914	339	0.023	1	1	2.583E-04	8.693E-04	PB-CAG-SD
C14orf64	chromosome 14 open reading frame 64 [Source:HGNC Symbol;Acc:20111]	chr14	98391947	98449461	264	0.023	1	1	2.633E-04	8.819E-04	PB-CAG-SD
BCL2L11	BCL2-like 11 (apoptosis facilitator) [Source:HGNC Symbol;Acc:994]	chr2	111871955	111926024	335	0.023	1	1	2.706E-04	9.003E-04	PB-CAG-SD
GPR160	G protein-coupled receptor 160 [Source:HGNC Symbol;Acc:23693]	chr3	169750717	169803191	348	0.024	1	1	2.721E-04	9.003E-04	PB-CAG-SD
GPR139	G protein-coupled receptor 139 [Source:HGNC Symbol;Acc:19995]	chr16	20042807	20090239	245	0.024	1	1	2.726E-04	9.003E-04	PB-CAG-SD
PDCD1LG2	programmed cell death 1 ligand 2 [Source:HGNC Symbol;Acc:18731]	chr9	5505545	5571282	406	0.024	1	1	2.813E-04	9.183E-04	PB-CAG-SD
ZNF665	zinc finger protein 665 [Source:HGNC Symbol;Acc:25885]	chr19	53662466	53701639	190	0.024	1	1	2.814E-04	9.183E-04	PB-CAG-SD
ZFP91	ZFP91 zinc finger protein [Source:HGNC Symbol;Acc:14983]	chr11	58341584	58388515	413	0.024	1	1	2.827E-04	9.183E-04	PB-CAG-SD
LEMD1	LEM domain containing 1 [Source:HGNC Symbol;Acc:18725]	chr1	205350506	205430082	347	0.024	1	1	2.847E-04	9.183E-04	PB-CAG-SD
CYP4B1	cytochrome P450, family 4, subfamily B, polypeptide 1 [Source:HGNC Symbol;Acc:2644]	chr1	47218510	47285085	347	0.024	1	1	2.847E-04	9.183E-04	PB-CAG-SD
KCNK9	potassium channel, subfamily K, member 9 [Source:HGNC Symbol;Acc:6283]	chr8	140613081	140720299	326	0.024	1	1	2.924E-04	9.389E-04	PB-CAG-SD
DDX52	DEAD (Asp-Glu-Ala-Asp) box polypeptide 52 [Source:HGNC Symbol;Acc:20038]	chr17	35969787	36008493	278	0.025	1	1	2.965E-04	9.478E-04	PB-CAG-SD
USP30	ubiquitin specific peptidase 30 [Source:HGNC Symbol;Acc:20065]	chr12	109455894	109525831	439	0.025	1	1	3.113E-04	9.904E-04	PB-CAG-SD
PLEKHA2	pleckstrin homology domain containing, family A (phosphoinositide binding specific) member 2 [Source:HGNC Symbol;Acc:14336]	chr8	38753753	38831428	339	0.025	1	1	3.160E-04	1.000E-03	PB-CAG-SD
ABHD5	abhydrolase domain containing 5 [Source:HGNC Symbol;Acc:21396]	chr3	43726605	43775863	376	0.025	1	1	3.172E-04	1.000E-03	PB-CAG-SD
SLC28A1	solute carrier family 28 (concentrative nucleoside transporter), member 1 [Source:HGNC Symbol;Acc:11001]	chr15	85422885	85518876	347	0.026	1	1	3.200E-04	1.004E-03	PB-CAG-SD
B3GALT1	UDP-Gal:betaGlcNAc beta 1,3-galactosyltransferase, polypeptide 1 [Source:HGNC Symbol;Acc:916]	chr2	168670182	168727366	366	0.026	1	1	3.225E-04	1.004E-03	PB-CAG-SD
UPB1	ureidopropionase, beta [Source:HGNC Symbol;Acc:16297]	chr22	24858206	24924358	281	0.026	1	1	3.248E-04	1.004E-03	PB-CAG-SD
ZFP91-CNTF	ZFP91-CNTF readthrough (NMD candidate) [Source:HGNC Symbol;Acc:33441]	chr11	58341645	58392112	443	0.026	1	1	3.249E-04	1.004E-03	PB-CAG-SD
ERAP1	endoplasmic reticulum aminopeptidase 1 [Source:HGNC Symbol;Acc:18173]	chr5	96096521	96148803	397	0.026	1	1	3.256E-04	1.004E-03	PB-CAG-SD
CDKL2	cyclin-dependent kinase-like 2 (CDC2-related kinase) [Source:HGNC Symbol;Acc:1782]	chr4	76503215	76560900	454	0.026	1	1	3.342E-04	1.026E-03	PB-CAG-SD
RAD21	RAD21 homolog (S. pombe) [Source:HGNC Symbol;Acc:9811]	chr8	117858174	117892105	350	0.026	1	1	3.366E-04	1.029E-03	PB-CAG-SD
TNFRSF11A	tumor necrosis factor receptor superfamily, member 11a, NFKB activator [Source:HGNC Symbol;Acc:11908]	chr18	59987520	60058516	402	0.026	1	1	3.405E-04	1.036E-03	PB-CAG-SD
C9orf3	chromosome 9 open reading frame 3 [Source:HGNC Symbol;Acc:1361]	chr9	97483983	97849441	2237	0.132	2	2	3.453E-04	1.041E-03	PB-CAG-SD
ZDBF2	zinc finger, DBF-type containing 2 [Source:HGNC Symbol;Acc:29313]	chr2	207134387	207179148	379	0.027	1	1	3.456E-04	1.041E-03	PB-CAG-SD
ZNF544	zinc finger protein 544 [Source:HGNC Symbol;Acc:16759]	chr19	58734960	58788815	211	0.027	1	1	3.465E-04	1.041E-03	PB-CAG-SD
STAT1	signal transducer and activator of transcription 1, 91kDa [Source:HGNC Symbol;Acc:11362]	chr2	191829084	191890686	384	0.027	1	1	3.547E-04	1.061E-03	PB-CAG-SD
CD38	CD38 molecule [Source:HGNC Symbol;Acc:1667]	chr4	15774898	15851069	470	0.027	1	1	3.580E-04	1.065E-03	PB-CAG-SD
KANK4	KN motif and ankyrin repeat domains 4 [Source:HGNC Symbol;Acc:27263]	chr1	62702651	62790085	390	0.027	1	1	3.589E-04	1.065E-03	PB-CAG-SD
THEGL	theg spermatid protein-like [Source:HGNC Symbol;Acc:43771]	chr4	57391775	57469673	473	0.027	1	1	3.625E-04	1.071E-03	PB-CAG-SD
ZNF81	zinc finger protein 81 [Source:HGNC Symbol;Acc:13156]	chrX	47691301	47861960	1070	0.027	1	1	3.650E-04	1.074E-03	PB-CAG-SD
TES	testis derived transcript (3 LIM domains) [Source:HGNC Symbol;Acc:14620]	chr7	115845547	115898837	436	0.027	1	1	3.686E-04	1.079E-03	PB-CAG-SD
SYNJ2	synaptojanin 2 [Source:HGNC Symbol;Acc:11504]	chr6	158397888	158520208	433	0.028	1	1	3.739E-04	1.090E-03	PB-CAG-SD
WBP1L	WW domain binding protein 1-like [Source:HGNC Symbol;Acc:23510]	chr10	104498727	104576021	339	0.028	1	1	3.800E-04	1.104E-03	PB-CAG-SD
PLCZ1	phospholipase C, zeta 1 [Source:HGNC Symbol;Acc:19218]	chr12	18836103	18895991	488	0.028	1	1	3.839E-04	1.111E-03	PB-CAG-SD
ESCO2	establishment of sister chromatid cohesion N-acetyltransferase 2 [Source:HGNC Symbol;Acc:27230]	chr8	27624466	27670157	375	0.028	1	1	3.860E-04	1.112E-03	PB-CAG-SD
IMMT	inner membrane protein, mitochondrial [Source:HGNC Symbol;Acc:6047]	chr2	86371055	86427893	402	0.028	1	1	3.884E-04	1.113E-03	PB-CAG-SD
ADORA2A-AS1	ADORA2A antisense RNA 1 [Source:HGNC Symbol;Acc:37122]	chr22	24825178	24896042	308	0.028	1	1	3.895E-04	1.113E-03	PB-CAG-SD
DZIP1	DAZ interacting zinc finger protein 1 [Source:HGNC Symbol;Acc:20908]	chr13	96230457	96301957	511	0.028	1	1	3.918E-04	1.115E-03	PB-CAG-SD
HSD17B12	hydroxysteroid (17-beta) dehydrogenase 12 [Source:HGNC Symbol;Acc:18646]	chr11	43572986	43878167	2381	0.138	2	1	3.966E-04	1.122E-03	PB-CAG-SD
FAM46A	family with sequence similarity 46, member A [Source:HGNC Symbol;Acc:18345]	chr6	82201156	82467491	2170	0.138	2	2	3.976E-04	1.122E-03	PB-CAG-SD
GEN1	GEN1 Holliday junction 5' flap endonuclease [Source:HGNC Symbol;Acc:26881]	chr2	17930125	17966632	416	0.029	1	1	4.157E-04	1.169E-03	PB-CAG-SD
ZNF586	zinc finger protein 586 [Source:HGNC Symbol;Acc:25949]	chr19	58276023	58331307	235	0.030	1	1	4.289E-04	1.198E-03	PB-CAG-SD
CD58	CD58 molecule [Source:HGNC Symbol;Acc:1688]	chr1	117057157	117118661	427	0.030	1	1	4.295E-04	1.198E-03	PB-CAG-SD
IGFBP7	insulin-like growth factor binding protein 7 [Source:HGNC Symbol;Acc:5476]	chr4	57896939	57981551	518	0.030	1	1	4.341E-04	1.206E-03	PB-CAG-SD
ZNF217	zinc finger protein 217 [Source:HGNC Symbol;Acc:13009]	chr20	52183604	52231446	355	0.030	1	1	4.394E-04	1.212E-03	PB-CAG-SD
NRDE2	NRDE-2, necessary for RNA interference, domain containing [Source:HGNC Symbol;Acc:20186]	chr14	90742580	90803481	342	0.030	1	1	4.399E-04	1.212E-03	PB-CAG-SD
ZNF333	zinc finger protein 333 [Source:HGNC Symbol;Acc:15624]	chr19	14795613	14844558	239	0.030	1	1	4.435E-04	1.216E-03	PB-CAG-SD
ENTPD7	ectonucleoside triphosphate diphosphohydrolase 7 [Source:HGNC Symbol;Acc:19745]	chr10	101414263	101465997	367	0.030	1	1	4.447E-04	1.216E-03	PB-CAG-SD
HOOK2	hook homolog 2 (Drosophila) [Source:HGNC Symbol;Acc:19885]	chr19	12873817	12988554	243	0.031	1	1	4.583E-04	1.248E-03	PB-CAG-SD
NEDD4L	neural precursor cell expressed, developmentally down-regulated 4-like, E3 ubiquitin protein ligase [Source:HGNC Symbol;Acc:7728]	chr18	55706599	56068772	2234	0.146	2	2	4.679E-04	1.269E-03	PB-CAG-SD
RPSAP52	ribosomal protein SA pseudogene 52 [Source:HGNC Symbol;Acc:35752]	chr12	66151800	66225754	543	0.031	1	1	4.744E-04	1.282E-03	PB-CAG-SD
SYK	spleen tyrosine kinase [Source:HGNC Symbol;Acc:11491]	chr9	93559069	93660831	530	0.031	1	1	4.770E-04	1.284E-03	PB-CAG-SD
CELF1	CUGBP, Elav-like family member 1 [Source:HGNC Symbol;Acc:2549]	chr11	47487496	47592121	541	0.031	1	1	4.827E-04	1.295E-03	PB-CAG-SD
LINC00371	long intergenic non-protein coding RNA 371 [Source:HGNC Symbol;Acc:42693]	chr13	51656984	51751524	572	0.032	1	1	4.899E-04	1.309E-03	PB-CAG-SD
ZNF492	zinc finger protein 492 [Source:HGNC Symbol;Acc:23707]	chr19	22812126	22850472	252	0.032	1	1	4.925E-04	1.311E-03	PB-CAG-SD
GNB4	guanine nucleotide binding protein (G protein), beta polypeptide 4 [Source:HGNC Symbol;Acc:20731]	chr3	179116990	179174378	472	0.032	1	1	4.978E-04	1.320E-03	PB-CAG-SD
DLGAP5	discs, large (Drosophila) homolog-associated protein 5 [Source:HGNC Symbol;Acc:16864]	chr14	55614830	55663396	368	0.032	1	1	5.085E-04	1.343E-03	PB-CAG-SD
RMI2	RecQ mediated genome instability 2 [Source:HGNC Symbol;Acc:28349]	chr16	11338476	11445619	338	0.032	1	1	5.159E-04	1.358E-03	PB-CAG-SD
AHRR	aryl-hydrocarbon receptor repressor [Source:HGNC Symbol;Acc:346]	chr5	299291	438406	506	0.033	1	1	5.265E-04	1.380E-03	PB-CAG-SD
SLC36A4	solute carrier family 36 (proton/amino acid symporter), member 4 [Source:HGNC Symbol;Acc:19660]	chr11	92877341	92936130	581	0.034	1	1	5.558E-04	1.452E-03	PB-CAG-SD
LEF1-AS1	LEF1 antisense RNA 1 [Source:HGNC Symbol;Acc:40339]	chr4	109083681	109177992	595	0.034	1	1	5.710E-04	1.484E-03	PB-CAG-SD
NAB1	NGFI-A binding protein 1 (EGR1 binding protein 1) [Source:HGNC Symbol;Acc:7626]	chr2	191506472	191557492	489	0.034	1	1	5.724E-04	1.484E-03	PB-CAG-SD
NOVA1-AS1	NOVA1 antisense RNA 1 (head to head) [Source:HGNC Symbol;Acc:19827]	chr14	27062618	27275673	1799	0.158	2	1	5.798E-04	1.498E-03	PB-CAG-SD
ARNTL	aryl hydrocarbon receptor nuclear translocator-like [Source:HGNC Symbol;Acc:701]	chr11	13293199	13408813	597	0.035	1	1	5.865E-04	1.509E-03	PB-CAG-SD
KIAA0895	KIAA0895 [Source:HGNC Symbol;Acc:22206]	chr7	36363830	36434734	553	0.035	1	1	5.900E-04	1.513E-03	PB-CAG-SD
BFSP1	beaded filament structural protein 1, filensin [Source:HGNC Symbol;Acc:1040]	chr20	17474550	17554865	417	0.035	1	1	6.041E-04	1.543E-03	PB-CAG-SD
MOK	MOK protein kinase [Source:HGNC Symbol;Acc:9833]	chr14	102690837	102776537	403	0.035	1	1	6.086E-04	1.548E-03	PB-CAG-SD
ACADM	acyl-CoA dehydrogenase, C-4 to C-12 straight chain [Source:HGNC Symbol;Acc:89]	chr1	76185036	76253260	510	0.035	1	1	6.103E-04	1.548E-03	PB-CAG-SD
MYSM1	Myb-like, SWIRM and MPN domains 1 [Source:HGNC Symbol;Acc:29401]	chr1	59120411	59170764	511	0.035	1	1	6.127E-04	1.548E-03	PB-CAG-SD
BCL11B	B-cell CLL/lymphoma 11B (zinc finger protein) [Source:HGNC Symbol;Acc:13222]	chr14	99635624	99742861	405	0.035	1	1	6.146E-04	1.548E-03	PB-CAG-SD
ELMOD1	ELMO/CED-12 domain containing 1 [Source:HGNC Symbol;Acc:25334]	chr11	107456817	107537505	617	0.036	1	1	6.260E-04	1.566E-03	PB-CAG-SD
DIS3L2	DIS3 mitotic control homolog (S. cerevisiae)-like 2 [Source:HGNC Symbol;Acc:28648]	chr2	232820955	233209060	2313	0.162	2	2	6.264E-04	1.566E-03	PB-CAG-SD
GON4L	gon-4-like (C. elegans) [Source:HGNC Symbol;Acc:25973]	chr1	155719508	155834191	518	0.036	1	1	6.294E-04	1.568E-03	PB-CAG-SD
MORF4L1	mortality factor 4 like 1 [Source:HGNC Symbol;Acc:16989]	chr15	79097829	79190475	503	0.037	1	1	6.673E-04	1.654E-03	PB-CAG-SD

table S1. Genes with significant transposon integrations in DLD-1 *KRAS*$^{wt/-}$ cells after selection in low glucose medium.

Gene ID	Description	Chrom	Start	End	# TTAA sites	Expected	Observed	Libraries	*P*	FDR	Transposon
AGPAT5	1-acylglycerol-3-phosphate O-acyltransferase 5 [Source:HGNC Symbol;Acc:20886]	chr8	6560878	6617184	495	0.037	1	1	6.685E-04	1.654E-03	PB-CAG-SD
STK24	serine/threonine kinase 24 [Source:HGNC Symbol;Acc:11403]	chr13	99102455	99235194	671	0.037	1	1	6.717E-04	1.656E-03	PB-CAG-SD
FBXO36	F-box protein 36 [Source:HGNC Symbol;Acc:27020]	chr2	230782018	230877825	536	0.038	1	1	6.862E-04	1.685E-03	PB-CAG-SD
AP1S3	adaptor-related protein complex 1, sigma 3 subunit [Source:HGNC Symbol;Acc:18971]	chr2	224616403	224707744	537	0.038	1	1	6.887E-04	1.686E-03	PB-CAG-SD
ANO4	anoctamin 4 [Source:HGNC Symbol;Acc:23837]	chr12	101106304	101522419	2928	0.168	2	2	6.949E-04	1.689E-03	PB-CAG-SD
DLG5	discs, large homolog 5 (Drosophila) [Source:HGNC Symbol;Acc:2904]	chr10	79550549	79691378	461	0.038	1	1	6.982E-04	1.689E-03	PB-CAG-SD
PHEX	phosphate regulating endopeptidase homolog, X-linked [Source:HGNC Symbol;Acc:8918]	chrX	22045559	22269427	1486	0.038	1	1	6.991E-04	1.689E-03	PB-CAG-SD
KIFC3	kinesin family member C3 [Source:HGNC Symbol;Acc:6326]	chr16	57792129	57901957	395	0.038	1	1	7.019E-04	1.689E-03	PB-CAG-SD
EPB41L1	erythrocyte membrane protein band 4.1-like 1 [Source:HGNC Symbol;Acc:3378]	chr20	34674426	34820721	451	0.038	1	1	7.053E-04	1.689E-03	PB-CAG-SD
CRYM	crystallin, mu [Source:HGNC Symbol;Acc:2418]	chr16	21250195	21319404	396	0.038	1	1	7.055E-04	1.689E-03	PB-CAG-SD
SLC35G1	solute carrier family 35, member G1 [Source:HGNC Symbol;Acc:26607]	chr10	95648730	95715819	464	0.038	1	1	7.072E-04	1.689E-03	PB-CAG-SD
FAM154A	family with sequence similarity 154, member A [Source:HGNC Symbol;Acc:28566]	chr9	18927656	19038251	651	0.038	1	1	7.163E-04	1.702E-03	PB-CAG-SD
ESRP1	epithelial splicing regulatory protein 1 [Source:HGNC Symbol;Acc:25966]	chr8	95648302	95719694	513	0.038	1	1	7.174E-04	1.702E-03	PB-CAG-SD
PTDSS1	phosphatidylserine synthase 1 [Source:HGNC Symbol;Acc:9587]	chr8	97268943	97349223	514	0.038	1	1	7.201E-04	1.702E-03	PB-CAG-SD
SEL1L3	sel-1 suppressor of lin-12-like 3 (C. elegans) [Source:HGNC Symbol;Acc:29108]	chr4	25749055	25870382	670	0.038	1	1	7.220E-04	1.702E-03	PB-CAG-SD
KIF5B	kinesin family member 5B [Source:HGNC Symbol;Acc:6324]	chr10	32297938	32350359	474	0.039	1	1	7.376E-04	1.732E-03	PB-CAG-SD
PCOLCE2	procollagen C-endopeptidase enhancer 2 [Source:HGNC Symbol;Acc:8739]	chr3	142534764	142613045	577	0.039	1	1	7.404E-04	1.732E-03	PB-CAG-SD
MAP3K7CL	MAP3K7 C-terminal like [Source:HGNC Symbol;Acc:16457]	chr21	30444792	30548210	724	0.039	1	1	7.421E-04	1.732E-03	PB-CAG-SD
CNGA1	cyclic nucleotide gated channel alpha 1 [Source:HGNC Symbol;Acc:2148]	chr4	47937994	48023689	681	0.039	1	1	7.456E-04	1.734E-03	PB-CAG-SD
TMTC3	transmembrane and tetratricopeptide repeat containing 3 [Source:HGNC Symbol;Acc:26899]	chr12	88531073	88593664	686	0.039	1	1	7.530E-04	1.745E-03	PB-CAG-SD
LZTFL1	leucine zipper transcription factor-like 1 [Source:HGNC Symbol;Acc:6741]	chr3	45864808	45962534	585	0.040	1	1	7.608E-04	1.758E-03	PB-CAG-SD
EPT1	ethanolaminephosphotransferase 1 (CDP-ethanolamine-specific) [Source:HGNC Symbol;Acc:29361]	chr2	26526415	26618759	573	0.040	1	1	7.829E-04	1.803E-03	PB-CAG-SD
C7	complement component 7 [Source:HGNC Symbol;Acc:1346]	chr5	40904354	40983041	621	0.040	1	1	7.891E-04	1.811E-03	PB-CAG-SD
GAPVD1	GTPase activating protein and VPS9 domains 1 [Source:HGNC Symbol;Acc:23375]	chr9	128019073	128129486	696	0.041	1	1	8.173E-04	1.862E-03	PB-CAG-SD
CHM	choroideremia (Rab escort protein 1) [Source:HGNC Symbol;Acc:1940]	chrX	85116185	85307566	1610	0.041	1	1	8.189E-04	1.862E-03	PB-CAG-SD
FPR1	formyl peptide receptor 1 [Source:HGNC Symbol;Acc:3826]	chr19	52248425	52312363	326	0.041	1	1	8.192E-04	1.862E-03	PB-CAG-SD
NPTN	neuroplastin [Source:HGNC Symbol;Acc:17867]	chr15	73852355	73931475	559	0.041	1	1	8.219E-04	1.862E-03	PB-CAG-SD
HSDL2	hydroxysteroid dehydrogenase like 2 [Source:HGNC Symbol;Acc:18572]	chr9	115137217	115234690	716	0.042	1	1	8.643E-04	1.950E-03	PB-CAG-SD
ABCA4	ATP-binding cassette, sub-family A (ABC1), member 4 [Source:HGNC Symbol;Acc:34]	chr1	94458393	94591688	609	0.042	1	1	8.663E-04	1.950E-03	PB-CAG-SD
ARNTL2	aryl hydrocarbon receptor nuclear translocator-like 2 [Source:HGNC Symbol;Acc:18984]	chr12	27480787	27576241	739	0.042	1	1	8.721E-04	1.957E-03	PB-CAG-SD
GXYLT2	glucoside xylosyltransferase 2 [Source:HGNC Symbol;Acc:33383]	chr3	72932224	73047289	629	0.042	1	1	8.778E-04	1.963E-03	PB-CAG-SD
GABPB1	GA binding protein transcription factor, beta subunit 1 [Source:HGNC Symbol;Acc:4074]	chr15	50569389	50652605	603	0.044	1	1	9.543E-04	2.127E-03	PB-CAG-SD
UNC5C	unc-5 homolog C (C. elegans) [Source:HGNC Symbol;Acc:12569]	chr4	96083655	96475357	3278	0.188	2	1	9.670E-04	2.149E-03	PB-CAG-SD
RFFL	ring finger and FYVE-like domain containing E3 ubiquitin protein ligase [Source:HGNC Symbol;Acc:24821]	chr17	33333009	33421338	507	0.045	1	1	9.731E-04	2.156E-03	PB-CAG-SD
WASL	Wiskott-Aldrich syndrome-like [Source:HGNC Symbol;Acc:12735]	chr7	123321989	123394121	716	0.045	1	1	9.824E-04	2.162E-03	PB-CAG-SD
DZANK1	double zinc ribbon and ankyrin repeat domains 1 [Source:HGNC Symbol;Acc:15858]	chr20	18364011	18452829	534	0.045	1	1	9.842E-04	2.162E-03	PB-CAG-SD
NCK1	NCK adaptor protein 1 [Source:HGNC Symbol;Acc:7664]	chr3	136576050	136668665	667	0.045	1	1	9.854E-04	2.162E-03	PB-CAG-SD
MARCH6	membrane-associated ring finger (C3HC4) 6, E3 ubiquitin protein ligase [Source:HGNC Symbol;Acc:30550]	chr5	10348815	10440500	700	0.045	1	1	9.993E-04	2.181E-03	PB-CAG-SD
CPOX	coproporphyrinogen oxidase [Source:HGNC Symbol;Acc:2321]	chr3	98239976	98317567	672	0.045	1	1	1.000E-03	2.181E-03	PB-CAG-SD
TTLL5	tubulin tyrosine ligase-like family, member 5 [Source:HGNC Symbol;Acc:19963]	chr14	76094968	76421421	2188	0.192	2	1	1.017E-03	2.211E-03	PB-CAG-SD
ABCA17P	ATP-binding cassette, sub-family A (ABC1), member 17, pseudogene [Source:HGNC Symbol;Acc:32972]	chr16	2384151	2476700	480	0.046	1	1	1.031E-03	2.234E-03	PB-CAG-SD
EBF1	early B-cell factor 1 [Source:HGNC Symbol;Acc:3126]	chr5	158122928	158531769	2984	0.193	2	1	1.045E-03	2.257E-03	PB-CAG-SD
AK7	adenylate kinase 7 [Source:HGNC Symbol;Acc:20091]	chr14	96853448	96955764	535	0.047	1	1	1.064E-03	2.293E-03	PB-CAG-SD
USP12	ubiquitin specific peptidase 12 [Source:HGNC Symbol;Acc:20485]	chr13	27640293	27751033	861	0.048	1	1	1.098E-03	2.358E-03	PB-CAG-SD
LRIG1	leucine-rich repeats and immunoglobulin-like domains 1 [Source:HGNC Symbol;Acc:17360]	chr3	66429221	66556687	711	0.048	1	1	1.117E-03	2.379E-03	PB-CAG-SD
TEK	TEK tyrosine kinase, endothelial [Source:HGNC Symbol;Acc:11724]	chr9	27104139	27230173	816	0.048	1	1	1.118E-03	2.379E-03	PB-CAG-SD
MAP3K7	mitogen-activated protein kinase kinase kinase 7 [Source:HGNC Symbol;Acc:6859]	chr6	91223292	91301764	754	0.048	1	1	1.118E-03	2.379E-03	PB-CAG-SD
TOM1L2	target of myb1-like 2 (chicken) [Source:HGNC Symbol;Acc:11984]	chr17	17746828	17880736	550	0.049	1	1	1.142E-03	2.423E-03	PB-CAG-SD
FAM5B	family with sequence similarity 5, member B [Source:HGNC Symbol;Acc:13746]	chr1	177135633	177251558	705	0.049	1	1	1.156E-03	2.444E-03	PB-CAG-SD
LIFR-AS1	LIFR antisense RNA 1 [Source:HGNC Symbol;Acc:43600]	chr5	38551888	38671318	759	0.049	1	1	1.172E-03	2.471E-03	PB-CAG-SD
MFHAS1	malignant fibrous histiocytoma amplified sequence 1 [Source:HGNC Symbol;Acc:16982]	chr8	8640864	8756155	659	0.049	1	1	1.175E-03	2.471E-03	PB-CAG-SD
OPN3	opsin 3 [Source:HGNC Symbol;Acc:14007]	chr1	241753404	241845678	713	0.049	1	1	1.182E-03	2.477E-03	PB-CAG-SD
OXSR1	oxidative stress responsive 1 [Source:HGNC Symbol;Acc:8508]	chr3	38201580	38296979	733	0.050	1	1	1.186E-03	2.478E-03	PB-CAG-SD
COL9A1	collagen, type IX, alpha 1 [Source:HGNC Symbol;Acc:2217]	chr6	70924764	71017786	778	0.050	1	1	1.189E-03	2.478E-03	PB-CAG-SD
PPM1A	protein phosphatase, Mg2+/Mn2+ dependent, 1A [Source:HGNC Symbol;Acc:9275]	chr14	60707470	60765805	571	0.050	1	1	1.210E-03	2.513E-03	PB-CAG-SD
C12orf60	chromosome 12 open reading frame 60 [Source:HGNC Symbol;Acc:28726]	chr12	14951506	15059520	876	0.050	1	1	1.219E-03	2.517E-03	PB-CAG-SD
INTU	inturned planar cell polarity protein [Source:HGNC Symbol;Acc:29239]	chr4	128539426	128637930	874	0.050	1	1	1.219E-03	2.517E-03	PB-CAG-SD
CMTM8	CKLF-like MARVEL transmembrane domain containing 8 [Source:HGNC Symbol;Acc:19179]	chr3	32275171	32411817	748	0.051	1	1	1.235E-03	2.542E-03	PB-CAG-SD
MXI1	MAX interactor 1, dimerization protein [Source:HGNC Symbol;Acc:7534]	chr10	111962363	112047123	622	0.051	1	1	1.260E-03	2.586E-03	PB-CAG-SD
KIAA1328	KIAA1328 [Source:HGNC Symbol;Acc:29248]	chr18	34404069	34812135	3168	0.207	2	1	1.275E-03	2.610E-03	PB-CAG-SD
ARHGAP31	Rho GTPase activating protein 31 [Source:HGNC Symbol;Acc:29216]	chr3	119008220	119139561	766	0.052	1	1	1.294E-03	2.640E-03	PB-CAG-SD
ZNF720	zinc finger protein 720 [Source:HGNC Symbol;Acc:26987]	chr16	31719550	31806190	548	0.053	1	1	1.338E-03	2.723E-03	PB-CAG-SD
PLB1	phospholipase B1 [Source:HGNC Symbol;Acc:30041]	chr2	28675012	28866654	754	0.053	1	1	1.344E-03	2.727E-03	PB-CAG-SD
PAX3	paired box 3 [Source:HGNC Symbol;Acc:8617]	chr2	223064607	223168715	761	0.053	1	1	1.369E-03	2.765E-03	PB-CAG-SD
CIRH1A	cirrhosis, autosomal recessive 1A (cirhin) [Source:HGNC Symbol;Acc:1983]	chr16	69160194	69265033	556	0.053	1	1	1.377E-03	2.765E-03	PB-CAG-SD
CSMD2	CUB and Sushi multiple domains 2 [Source:HGNC Symbol;Acc:19290]	chr1	33979609	34636443	3076	0.213	2	1	1.378E-03	2.765E-03	PB-CAG-SD
DAPK2	death-associated protein kinase 2 [Source:HGNC Symbol;Acc:2675]	chr15	64199235	64369232	727	0.053	1	1	1.379E-03	2.765E-03	PB-CAG-SD
DLEU2	deleted in lymphocytic leukemia 2 (non-protein coding) [Source:HGNC Symbol;Acc:13748]	chr13	50601269	50704856	974	0.054	1	1	1.400E-03	2.790E-03	PB-CAG-SD
RIN3	Ras and Rab interactor 3 [Source:HGNC Symbol;Acc:18751]	chr14	92975118	93155339	615	0.054	1	1	1.400E-03	2.790E-03	PB-CAG-SD
MTR	5-methyltetrahydrofolate-homocysteine methyltransferase [Source:HGNC Symbol;Acc:7468]	chr1	236953610	237067281	778	0.054	1	1	1.403E-03	2.790E-03	PB-CAG-SD
CCDC170	coiled-coil domain containing 170 [Source:HGNC Symbol;Acc:21177]	chr6	151810165	151942328	849	0.054	1	1	1.412E-03	2.800E-03	PB-CAG-SD
GLIS1	GLIS family zinc finger 1 [Source:HGNC Symbol;Acc:29525]	chr1	53971910	54204877	783	0.054	1	1	1.421E-03	2.809E-03	PB-CAG-SD
JMJD1C	jumonji domain containing 1C [Source:HGNC Symbol;Acc:12313]	chr10	64926981	65230722	2635	0.216	2	1	1.435E-03	2.830E-03	PB-CAG-SD
RDX	radixin [Source:HGNC Symbol;Acc:9944]	chr11	110045605	110172447	942	0.055	1	1	1.441E-03	2.833E-03	PB-CAG-SD
UQCC	ubiquinol-cytochrome c reductase complex chaperone [Source:HGNC Symbol;Acc:15891]	chr20	33890369	34004944	652	0.055	1	1	1.458E-03	2.858E-03	PB-CAG-SD
TEC	tec protein tyrosine kinase [Source:HGNC Symbol;Acc:11719]	chr4	48137800	48276881	966	0.055	1	1	1.484E-03	2.902E-03	PB-CAG-SD
UBE2H	ubiquitin-conjugating enzyme E2H [Source:HGNC Symbol;Acc:12484]	chr7	129470572	129597789	894	0.056	1	1	1.520E-03	2.959E-03	PB-CAG-SD
PREP	prolyl endopeptidase [Source:HGNC Symbol;Acc:9358]	chr6	105725440	105855959	882	0.056	1	1	1.522E-03	2.959E-03	PB-CAG-SD
MMP2	matrix metallopeptidase 2 (gelatinase A, 72kDa gelatinase, 72kDa type IV collagenase) [Source:HGNC Symbol;Acc:7166]	chr16	55418612	55540603	592	0.057	1	1	1.557E-03	3.003E-03	PB-CAG-SD
ZMYND8	zinc finger, MYND-type containing 8 [Source:HGNC Symbol;Acc:9397]	chr20	45837859	45990567	675	0.057	1	1	1.560E-03	3.003E-03	PB-CAG-SD
CDKN2B-AS1	CDKN2B antisense RNA 1 [Source:HGNC Symbol;Acc:34341]	chr9	21989777	22121096	968	0.057	1	1	1.564E-03	3.003E-03	PB-CAG-SD
TRIP11	thyroid hormone receptor interactor 11 [Source:HGNC Symbol;Acc:12305]	chr14	92432335	92512240	651	0.057	1	1	1.565E-03	3.003E-03	PB-CAG-SD
ACVR1C	activin A receptor, type IC [Source:HGNC Symbol;Acc:18123]	chr2	158388888	158490517	815	0.057	1	1	1.566E-03	3.003E-03	PB-CAG-SD
FAM73A	family with sequence similarity 73, member A [Source:HGNC Symbol;Acc:24741]	chr1	78240309	78344106	825	0.057	1	1	1.574E-03	3.010E-03	PB-CAG-SD
CEP290	centrosomal protein 290kDa [Source:HGNC Symbol;Acc:29021]	chr12	88442793	88540993	999	0.057	1	1	1.578E-03	3.010E-03	PB-CAG-SD
ZBTB38	zinc finger and BTB domain containing 38 [Source:HGNC Symbol;Acc:26636]	chr3	141038055	141168634	850	0.057	1	1	1.587E-03	3.019E-03	PB-CAG-SD
NAA15	N(alpha)-acetyltransferase 15, NatA auxiliary subunit [Source:HGNC Symbol;Acc:30782]	chr4	140217609	140341187	1006	0.058	1	1	1.607E-03	3.048E-03	PB-CAG-SD
PPP6R3	protein phosphatase 6, regulatory subunit 3 [Source:HGNC Symbol;Acc:1173]	chr11	68223186	68382802	1000	0.058	1	1	1.620E-03	3.063E-03	PB-CAG-SD
PKD1L1	polycystic kidney disease 1 like 1 [Source:HGNC Symbol;Acc:18053]	chr7	47814250	47993088	925	0.058	1	1	1.625E-03	3.063E-03	PB-CAG-SD
FANK1	fibronectin type III and ankyrin repeat domains 1 [Source:HGNC Symbol;Acc:23527]	chr10	127580108	127698161	709	0.058	1	1	1.629E-03	3.063E-03	PB-CAG-SD
NIPAL1	NIPA-like domain containing 1 [Source:HGNC Symbol;Acc:27194]	chr4	47911159	48042188	1015	0.058	1	1	1.635E-03	3.063E-03	PB-CAG-SD
UBE2E2	ubiquitin-conjugating enzyme E2E 2 [Source:HGNC Symbol;Acc:12478]	chr3	23239511	23633284	3353	0.227	2	1	1.636E-03	3.063E-03	PB-CAG-SD
SEMA5A	sema domain, seven thrombospondin repeats (type 1 and type 1-like), transmembrane domain (TM) and short cytoplasmic domain, (semaphorin) 5A [Source	chr5	9035138	9551187	3500	0.227	2	1	1.644E-03	3.067E-03	PB-CAG-SD
CCDC147	coiled-coil domain containing 147 [Source:HGNC Symbol;Acc:26676]	chr10	106108522	106214848	713	0.059	1	1	1.647E-03	3.067E-03	PB-CAG-SD
COA1	cytochrome c oxidase assembly factor 1 homolog (S. cerevisiae) [Source:HGNC Symbol;Acc:21868]	chr7	43648055	43774316	935	0.059	1	1	1.660E-03	3.080E-03	PB-CAG-SD
IQCH-AS1	IQCH antisense RNA 1 [Source:HGNC Symbol;Acc:44104]	chr15	67695957	67819182	800	0.059	1	1	1.664E-03	3.080E-03	PB-CAG-SD
CAB39	calcium binding protein 39 [Source:HGNC Symbol;Acc:20292]	chr2	231572560	231685790	842	0.059	1	1	1.669E-03	3.080E-03	PB-CAG-SD
TMOD3	tropomodulin 3 (ubiquitous) [Source:HGNC Symbol;Acc:11873]	chr15	52116825	52239492	802	0.059	1	1	1.672E-03	3.080E-03	PB-CAG-SD
GARNL3	GTPase activating Rap/RanGAP domain-like 3 [Source:HGNC Symbol;Acc:25425]	chr9	129981544	130155939	1004	0.059	1	1	1.680E-03	3.087E-03	PB-CAG-SD
MET	met proto-oncogene [Source:HGNC Symbol;Acc:7029]	chr7	116307444	116438440	955	0.060	1	1	1.730E-03	3.166E-03	PB-CAG-SD
ARFGEF2	ADP-ribosylation factor guanine nucleotide-exchange factor 2 (brefeldin A-inhibited) [Source:HGNC Symbol;Acc:15853]	chr20	47533427	47653230	712	0.060	1	1	1.732E-03	3.166E-03	PB-CAG-SD
BCAT1	branched chain amino-acid transaminase 1, cytosolic [Source:HGNC Symbol;Acc:976]	chr12	24964295	25107393	1052	0.060	1	1	1.746E-03	3.183E-03	PB-CAG-SD
PIK3C2A	phosphatidylinositol-4-phosphate 3-kinase, catalytic subunit type 2 alpha [Source:HGNC Symbol;Acc:8971]	chr11	17099277	17234530	1050	0.061	1	1	1.783E-03	3.242E-03	PB-CAG-SD
ZNF573	zinc finger protein 573 [Source:HGNC Symbol;Acc:26420]	chr19	38226734	38312940	485	0.061	1	1	1.789E-03	3.245E-03	PB-CAG-SD
IL16	interleukin 16 [Source:HGNC Symbol;Acc:5980]	chr15	81446916	81605104	846	0.062	1	1	1.856E-03	3.356E-03	PB-CAG-SD
FAT1	FAT atypical cadherin 1 [Source:HGNC Symbol;Acc:3595]	chr4	187508937	187652876	1084	0.062	1	1	1.860E-03	3.356E-03	PB-CAG-SD
SGCG	sarcoglycan, gamma (35kDa dystrophin-associated glycoprotein) [Source:HGNC Symbol;Acc:10809]	chr13	23750091	23899304	1131	0.063	1	1	1.876E-03	3.376E-03	PB-CAG-SD
ABCB11	ATP-binding cassette, sub-family B (MDR/TAP), member 11 [Source:HGNC Symbol;Acc:42]	chr2	169779448	169892832	903	0.063	1	1	1.915E-03	3.437E-03	PB-CAG-SD
PCNXL4	pecanex-like 4 (Drosophila) [Source:HGNC Symbol;Acc:20349]	chr14	60553629	60635851	726	0.064	1	1	1.938E-03	3.470E-03	PB-CAG-SD
SYNRG	synergin, gamma [Source:HGNC Symbol;Acc:557]	chr17	35874900	35974544	722	0.064	1	1	1.949E-03	3.480E-03	PB-CAG-SD
ZZEF1	zinc finger, ZZ-type with EF-hand domain 1 [Source:HGNC Symbol;Acc:29027]	chr17	3907739	4051314	724	0.064	1	1	1.959E-03	3.486E-03	PB-CAG-SD
TRAF3	TNF receptor-associated factor 3 [Source:HGNC Symbol;Acc:12033]	chr14	103238813	103377837	731	0.064	1	1	1.965E-03	3.486E-03	PB-CAG-SD
CCDC60	coiled-coil domain containing 60 [Source:HGNC Symbol;Acc:28610]	chr12	119767517	119978852	1118	0.064	1	1	1.967E-03	3.486E-03	PB-CAG-SD
FLRT2	fibronectin leucine rich transmembrane protein 2 [Source:HGNC Symbol;Acc:3761]	chr14	85991488	86095034	735	0.064	1	1	1.986E-03	3.505E-03	PB-CAG-SD
POLR3B	polymerase (RNA) III (DNA directed) polypeptide B [Source:HGNC Symbol;Acc:30348]	chr12	106746436	106903976	1124	0.064	1	1	1.988E-03	3.505E-03	PB-CAG-SD
ELOVL6	ELOVL fatty acid elongase 6 [Source:HGNC Symbol;Acc:15829]	chr4	110967002	111125355	1126	0.065	1	1	2.004E-03	3.525E-03	PB-CAG-SD
LINC00877	long intergenic non-protein coding RNA 877 [Source:HGNC Symbol;Acc:27706]	chr3	72084451	72296716	959	0.065	1	1	2.010E-03	3.527E-03	PB-CAG-SD
VCAN	versican [Source:HGNC Symbol;Acc:2464]	chr5	82762284	82878122	1013	0.066	1	1	2.065E-03	3.608E-03	PB-CAG-SD
OPHN1	oligophrenin 1 [Source:HGNC Symbol;Acc:8148]	chrX	67262186	67658755	2579	0.066	1	1	2.067E-03	3.608E-03	PB-CAG-SD
CLIC5	chloride intracellular channel 5 [Source:HGNC Symbol;Acc:13517]	chr6	45868045	46053132	1041	0.066	1	1	2.106E-03	3.667E-03	PB-CAG-SD
CASC9	cancer susceptibility candidate 9 (non-protein coding) [Source:HGNC Symbol;Acc:48906]	chr8	76135639	76241976	890	0.067	1	1	2.119E-03	3.681E-03	PB-CAG-SD
RAD51B	RAD51 paralog B [Source:HGNC Symbol;Acc:9822]	chr14	68281496	69196935	6038	0.529	3	2	2.144E-03	3.714E-03	PB-CAG-SD
RAP1GAP2	RAP1 GTPase activating protein 2 [Source:HGNC Symbol;Acc:29176]	chr17	2675350	2941033	759	0.067	1	1	2.149E-03	3.714E-03	PB-CAG-SD
DLEU1	deleted in lymphocytic leukemia 1 (non-protein coding) [Source:HGNC Symbol;Acc:13747]	chr13	50651307	51297372	4542	0.251	2	1	2.190E-03	3.776E-03	PB-CAG-SD
ADAM9	ADAM metallopeptidase domain 9 [Source:HGNC Symbol;Acc:216]	chr8	38849388	38962663	908	0.068	1	1	2.204E-03	3.790E-03	PB-CAG-SD
UBAC2	UBA domain containing 2 [Source:HGNC Symbol;Acc:20486]	chr13	99848028	100038688	1232	0.068	1	1	2.218E-03	3.798E-03	PB-CAG-SD
PACSIN2	protein kinase C and casein kinase substrate in neurons 2 [Source:HGNC Symbol;Acc:8571]	chr22	43231418	43416151	745	0.068	1	1	2.219E-03	3.798E-03	PB-CAG-SD
SLC12A1	solute carrier family 12 (sodium/potassium/chloride transporters), member 1 [Source:HGNC Symbol;Acc:10910]	chr15	48478861	48596275	928	0.068	1	1	2.225E-03	3.798E-03	PB-CAG-SD
TFPI	tissue factor pathway inhibitor (lipoprotein-associated coagulation inhibitor) [Source:HGNC Symbol;Acc:11760]	chr2	188328957	188435487	982	0.069	1	1	2.256E-03	3.842E-03	PB-CAG-SD
CRHR1	corticotropin releasing hormone receptor 1 [Source:HGNC Symbol;Acc:2357]	chr17	43694267	43913194	787	0.070	1	1	2.307E-03	3.919E-03	PB-CAG-SD
ANK1	ankyrin 1, erythrocytic [Source:HGNC Symbol;Acc:492]	chr8	41510739	41759280	942	0.070	1	1	2.368E-03	4.013E-03	PB-CAG-SD
SLC12A2	solute carrier family 12 (sodium/potassium/chloride transporters), member 2 [Source:HGNC Symbol;Acc:10911]	chr5	127414458	127525380	1102	0.071	1	1	2.434E-03	4.116E-03	PB-CAG-SD
CLNK	cytokine-dependent hematopoietic cell linker [Source:HGNC Symbol;Acc:17438]	chr4	10488019	10691489	1250	0.072	1	1	2.458E-03	4.146E-03	PB-CAG-SD
TDRD12	tudor domain containing 12 [Source:HGNC Symbol;Acc:25044]	chr19	33205659	33320483	574	0.072	1	1	2.488E-03	4.186E-03	PB-CAG-SD
ZNRF2	zinc and ring finger 2 [Source:HGNC Symbol;Acc:22316]	chr7	30318923	30452118	1152	0.072	1	1	2.497E-03	4.192E-03	PB-CAG-SD
MYO16	myosin XVI [Source:HGNC Symbol;Acc:29822]	chr13	109243500	109860355	4767	0.264	2	1	2.509E-03	4.202E-03	PB-CAG-SD
KIAA0922	KIAA0922 [Source:HGNC Symbol;Acc:29146]	chr4	154382498	154557863	1268	0.073	1	1	2.528E-03	4.218E-03	PB-CAG-SD
SHC4	SHC (Src homology 2 domain containing) family, member 4 [Source:HGNC Symbol;Acc:16743]	chr15	49115932	49260641	992	0.073	1	1	2.534E-03	4.218E-03	PB-CAG-SD
ST8SIA6	ST8 alpha-N-acetyl-neuraminide alpha-2,8-sialyltransferase 6 [Source:HGNC Symbol;Acc:23317]	chr10	17360382	17501329	890	0.073	1	1	2.542E-03	4.218E-03	PB-CAG-SD
FAM168A	family with sequence similarity 168, member A [Source:HGNC Symbol;Acc:28999]	chr11	73111532	73314234	1259	0.073	1	1	2.543E-03	4.218E-03	PB-CAG-SD
ADCY9	adenylate cyclase 9 [Source:HGNC Symbol;Acc:240]	chr16	4003388	4171186	774	0.074	1	1	2.631E-03	4.354E-03	PB-CAG-SD
SORCS2	sortilin-related VPS10 domain containing receptor 2 [Source:HGNC Symbol;Acc:16698]	chr4	7189265	7744554	1311	0.075	1	1	2.698E-03	4.453E-03	PB-CAG-SD
BCAS1	breast carcinoma amplified sequence 1 [Source:HGNC Symbol;Acc:974]	chr20	52553316	52692304	894	0.075	1	1	2.703E-03	4.453E-03	PB-CAG-SD
SLC2A9	solute carrier family 2 (facilitated glucose transporter), member 9 [Source:HGNC Symbol;Acc:13446]	chr4	9772777	10061560	1320	0.076	1	1	2.734E-03	4.465E-03	PB-CAG-SD
RNF217	ring finger protein 217 [Source:HGNC Symbol;Acc:21487]	chr6	125278691	125413779	1190	0.076	1	1	2.735E-03	4.465E-03	PB-CAG-SD
TCERG1L	transcription elongation regulator 1-like [Source:HGNC Symbol;Acc:23533]	chr10	132890654	133114984	924	0.076	1	1	2.735E-03	4.465E-03	PB-CAG-SD
ARHGAP18	Rho GTPase activating protein 18 [Source:HGNC Symbol;Acc:21035]	chr6	129897277	130036370	1191	0.076	1	1	2.739E-03	4.465E-03	PB-CAG-SD
PCBP3	poly(rC) binding protein 3 [Source:HGNC Symbol;Acc:8651]	chr21	47058608	47362368	1409	0.076	1	1	2.743E-03	4.465E-03	PB-CAG-SD
COL4A4	collagen, type IV, alpha 4 [Source:HGNC Symbol;Acc:2206]	chr2	227867427	228033829	1088	0.076	1	1	2.756E-03	4.476E-03	PB-CAG-SD
SLC23A2	solute carrier family 23 (nucleobase transporters), member 2 [Source:HGNC Symbol;Acc:10973]	chr20	4833002	4995939	911	0.077	1	1	2.805E-03	4.545E-03	PB-CAG-SD
ABI1	abl-interactor 1 [Source:HGNC Symbol;Acc:11320]	chr10	27035522	27155016	946	0.078	1	1	2.863E-03	4.629E-03	PB-CAG-SD
HECTD4	HECT domain containing E3 ubiquitin protein ligase 4 [Source:HGNC Symbol;Acc:26611]	chr12	112597992	112824896	1366	0.078	1	1	2.909E-03	4.693E-03	PB-CAG-SD
EEFSEC	eukaryotic elongation factor, selenocysteine-tRNA-specific [Source:HGNC Symbol;Acc:24614]	chr3	127867297	128127485	1163	0.079	1	1	2.930E-03	4.715E-03	PB-CAG-SD
MBOAT2	membrane bound O-acyltransferase domain containing 2 [Source:HGNC Symbol;Acc:25193]	chr2	8992820	9148942	1127	0.079	1	1	2.952E-03	4.739E-03	PB-CAG-SD
TRAK1	trafficking protein, kinesin binding 1 [Source:HGNC Symbol;Acc:29947]	chr3	42050294	42267381	1170	0.079	1	1	2.964E-03	4.748E-03	PB-CAG-SD
DPY19L3	dpy-19-like 3 (C. elegans) [Source:HGNC Symbol;Acc:27120]	chr19	32891655	32976801	629	0.079	1	1	2.973E-03	4.752E-03	PB-CAG-SD
SH3RF1	SH3 domain containing ring finger 1 [Source:HGNC Symbol;Acc:17650]	chr4	170015407	170197256	1381	0.079	1	1	2.985E-03	4.760E-03	PB-CAG-SD
ARHGEF38	Rho guanine nucleotide exchange factor (GEF) 38 [Source:HGNC Symbol;Acc:25968]	chr4	106468777	106629250	1388	0.080	1	1	3.015E-03	4.796E-03	PB-CAG-SD
FTO	fat mass and obesity associated [Source:HGNC Symbol;Acc:24678]	chr16	53732875	54155853	2935	0.282	2	2	3.028E-03	4.806E-03	PB-CAG-SD

table S1. Genes with significant transposon integrations in DLD-1 *KRAS*$^{wt/-}$ cells after selection in low glucose medium.

Gene ID	Description	Chrom	Start	End	# TTAA sites	Expected	Observed	Libraries	*P*	FDR	Transposon
ELAVL4	ELAV (embryonic lethal, abnormal vision, Drosophila)-like 4 [Source:HGNC Symbol;Acc:3315]	chr1	50508686	50669458	1157	0.080	1	1	3.049E-03	4.829E-03	PB-CAG-SD
WDR64	WD repeat domain 64 [Source:HGNC Symbol;Acc:26570]	chr1	241810580	241965435	1169	0.081	1	1	3.111E-03	4.916E-03	PB-CAG-SD
FREM1	FRAS1 related extracellular matrix 1 [Source:HGNC Symbol;Acc:23399]	chr9	14734664	14915993	1383	0.081	1	1	3.142E-03	4.953E-03	PB-CAG-SD
KIAA1468	KIAA1468 [Source:HGNC Symbol;Acc:29289]	chr18	59849491	59974355	1248	0.082	1	1	3.163E-03	4.976E-03	PB-CAG-SD
SRGAP2B	SLIT-ROBO Rho GTPase activating protein 2B [Source:HGNC Symbol;Acc:35237]	chr1	143908615	144094424	1181	0.082	1	1	3.173E-03	4.981E-03	PB-CAG-SD
TULP4	tubby like protein 4 [Source:HGNC Symbol;Acc:15530]	chr6	158728692	158932860	1289	0.082	1	1	3.195E-03	5.004E-03	PB-CAG-SD
LINC00970	long intergenic non-protein coding RNA 970 [Source:HGNC Symbol;Acc:48730]	chr1	168873143	169061243	1189	0.082	1	1	3.215E-03	5.024E-03	PB-CAG-SD
DNAJC13	DnaJ (Hsp40) homolog, subfamily C, member 13 [Source:HGNC Symbol;Acc:30343]	chr3	132131370	132257876	1231	0.083	1	1	3.273E-03	5.091E-03	PB-CAG-SD
PAN3	PAN3 poly(A) specific ribonuclease subunit homolog (S. cerevisiae) [Source:HGNC Symbol;Acc:29991]	chr13	28707643	28869475	1504	0.083	1	1	3.273E-03	5.091E-03	PB-CAG-SD
DDX60L	DEAD (Asp-Glu-Ala-Asp) box polypeptide 60-like [Source:HGNC Symbol;Acc:26429]	chr4	169277886	169463937	1463	0.084	1	1	3.340E-03	5.184E-03	PB-CAG-SD
LNX1	ligand of numb-protein X 1, E3 ubiquitin protein ligase [Source:HGNC Symbol;Acc:6657]	chr4	54325468	54572572	1470	0.084	1	1	3.371E-03	5.221E-03	PB-CAG-SD
CPVL	carboxypeptidase, vitellogenic-like [Source:HGNC Symbol;Acc:14399]	chr7	29034847	29240067	1374	0.086	1	1	3.520E-03	5.439E-03	PB-CAG-SD
LEPREL1	leprecan-like 1 [Source:HGNC Symbol;Acc:19317]	chr3	189674517	189845226	1297	0.088	1	1	3.622E-03	5.576E-03	PB-CAG-SD
HOMER1	homer homolog 1 (Drosophila) [Source:HGNC Symbol;Acc:17512]	chr5	78668459	78815040	1352	0.088	1	1	3.625E-03	5.576E-03	PB-CAG-SD
MORC1	MORC family CW-type zinc finger 1 [Source:HGNC Symbol;Acc:7198]	chr3	108677086	108841989	1310	0.089	1	1	3.693E-03	5.664E-03	PB-CAG-SD
FANCC	Fanconi anemia, complementation group C [Source:HGNC Symbol;Acc:3584]	chr9	97861336	98084991	1504	0.089	1	1	3.698E-03	5.664E-03	PB-CAG-SD
SYT1	synaptotagmin I [Source:HGNC Symbol;Acc:11509]	chr12	79252773	79845788	5297	0.304	2	2	3.721E-03	5.675E-03	PB-CAG-SD
SYT9	synaptotagmin IX [Source:HGNC Symbol;Acc:19265]	chr11	7255009	7490273	1531	0.089	1	1	3.721E-03	5.675E-03	PB-CAG-SD
ZFAT	zinc finger and AT hook domain containing [Source:HGNC Symbol;Acc:19899]	chr8	135490031	135730292	1210	0.090	1	1	3.855E-03	5.867E-03	PB-CAG-SD
ITSN1	intersectin 1 (SH3 domain protein) [Source:HGNC Symbol;Acc:6183]	chr21	35009706	35272165	1701	0.092	1	1	3.956E-03	6.007E-03	PB-CAG-SD
SMC6	structural maintenance of chromosomes 6 [Source:HGNC Symbol;Acc:20466]	chr2	17845079	17986509	1314	0.092	1	1	3.978E-03	6.027E-03	PB-CAG-SD
STARD13	StAR-related lipid transfer (START) domain containing 13 [Source:HGNC Symbol;Acc:19164]	chr13	33677272	33929767	1681	0.093	1	1	4.062E-03	6.142E-03	PB-CAG-SD
ADAM32	ADAM metallopeptidase domain 32 [Source:HGNC Symbol;Acc:15479]	chr8	38959509	39142430	1254	0.094	1	1	4.132E-03	6.234E-03	PB-CAG-SD
FAM65B	family with sequence similarity 65, member B [Source:HGNC Symbol;Acc:13872]	chr6	24797601	25047238	1494	0.095	1	1	4.256E-03	6.406E-03	PB-CAG-SD
HUS1	HUS1 checkpoint homolog (S. pombe) [Source:HGNC Symbol;Acc:5309]	chr7	47735328	48024178	1520	0.096	1	1	4.282E-03	6.414E-03	PB-CAG-SD
ARHGAP12	Rho GTPase activating protein 12 [Source:HGNC Symbol;Acc:16348]	chr10	32094365	32222742	1164	0.096	1	1	4.284E-03	6.414E-03	PB-CAG-SD
DOCK8	dedicator of cytokinesis 8 [Source:HGNC Symbol;Acc:19191]	chr9	209854	465259	1625	0.096	1	1	4.297E-03	6.414E-03	PB-CAG-SD
NR2F1-AS1	NR2F1 antisense RNA 1 [Source:HGNC Symbol;Acc:48622]	chr5	92745065	92926354	1476	0.096	1	1	4.297E-03	6.414E-03	PB-CAG-SD
NRG1	neuregulin 1 [Source:HGNC Symbol;Acc:7997]	chr8	31491902	32622548	8648	0.647	3	2	4.371E-03	6.509E-03	PB-CAG-SD
SORCS3	sortilin-related VPS10 domain containing receptor 3 [Source:HGNC Symbol;Acc:16699]	chr10	106395859	107024993	3931	0.323	2	1	4.404E-03	6.546E-03	PB-CAG-SD
MYO1B	myosin IB [Source:HGNC Symbol;Acc:7596]	chr2	192104911	192290115	1389	0.097	1	1	4.429E-03	6.569E-03	PB-CAG-SD
BPTF	bromodomain PHD finger transcription factor [Source:HGNC Symbol;Acc:3581]	chr17	65816640	65980494	1103	0.097	1	1	4.448E-03	6.583E-03	PB-CAG-SD
FARP1	FERM, RhoGEF (ARHGEF) and pleckstrin domain protein 1 (chondrocyte-derived) [Source:HGNC Symbol;Acc:3591]	chr13	98789816	99102027	1769	0.098	1	1	4.484E-03	6.622E-03	PB-CAG-SD
CDH12	cadherin 12, type 2 (N-cadherin 2) [Source:HGNC Symbol;Acc:1751]	chr5	21750782	22858731	10060	0.652	3	1	4.502E-03	6.623E-03	PB-CAG-SD
RBPMS	RNA binding protein with multiple splicing [Source:HGNC Symbol;Acc:19097]	chr8	30236944	30429778	1311	0.098	1	1	4.503E-03	6.623E-03	PB-CAG-SD
FAM49B	family with sequence similarity 49, member B [Source:HGNC Symbol;Acc:25216]	chr8	130851839	131034375	1330	0.099	1	1	4.631E-03	6.795E-03	PB-CAG-SD
EXOC2	exocyst complex component 2 [Source:HGNC Symbol;Acc:24968]	chr6	485133	698117	1569	0.100	1	1	4.679E-03	6.852E-03	PB-CAG-SD
MYO6	myosin VI [Source:HGNC Symbol;Acc:7605]	chr6	76453909	76629254	1575	0.100	1	1	4.713E-03	6.888E-03	PB-CAG-SD
ADAM10	ADAM metallopeptidase domain 10 [Source:HGNC Symbol;Acc:188]	chr15	58887403	59047177	1382	0.102	1	1	4.826E-03	7.038E-03	PB-CAG-SD
AP2B1	adaptor-related protein complex 2, beta 1 subunit [Source:HGNC Symbol;Acc:563]	chr17	33900065	34053436	1154	0.102	1	1	4.854E-03	7.064E-03	PB-CAG-SD
MAN1A1	mannosidase, alpha, class 1A, member 1 [Source:HGNC Symbol;Acc:6821]	chr6	119498374	119675926	1602	0.102	1	1	4.871E-03	7.074E-03	PB-CAG-SD
RPH3A	rabphilin 3A homolog (mouse) [Source:HGNC Symbol;Acc:17056]	chr12	113003184	113336686	1787	0.102	1	1	4.900E-03	7.102E-03	PB-CAG-SD
MSRB3	methionine sulfoxide reductase B3 [Source:HGNC Symbol;Acc:27375]	chr12	65667423	65882024	1798	0.103	1	1	4.959E-03	7.172E-03	PB-CAG-SD
LIPA	lipase A, lysosomal acid, cholesterol esterase [Source:HGNC Symbol;Acc:6617]	chr10	90973326	91179314	1260	0.103	1	1	4.994E-03	7.200E-03	PB-CAG-SD
GAS7	growth arrest-specific 7 [Source:HGNC Symbol;Acc:4169]	chr17	9813926	10106868	1172	0.104	1	1	5.002E-03	7.200E-03	PB-CAG-SD
LCOR	ligand dependent nuclear receptor corepressor [Source:HGNC Symbol;Acc:29503]	chr10	98587017	98740800	1262	0.104	1	1	5.009E-03	7.200E-03	PB-CAG-SD
OSBPL6	oxysterol binding protein-like 6 [Source:HGNC Symbol;Acc:16388]	chr2	179054208	179264160	1515	0.106	1	1	5.239E-03	7.515E-03	PB-CAG-SD
PPM1H	protein phosphatase, Mg2+/Mn2+ dependent, 1H [Source:HGNC Symbol;Acc:18583]	chr12	63037762	63333817	1861	0.107	1	1	5.300E-03	7.586E-03	PB-CAG-SD
SEMA3C	sema domain, immunoglobulin domain (Ig), short basic domain, secreted, (semaphorin) 3C [Source:HGNC Symbol;Acc:10725]	chr7	80371854	80556675	1709	0.107	1	1	5.370E-03	7.672E-03	PB-CAG-SD
SPON1	spondin 1, extracellular matrix protein [Source:HGNC Symbol;Acc:11252]	chr11	13978914	14289646	1860	0.108	1	1	5.423E-03	7.732E-03	PB-CAG-SD
LHFPL2	lipoma HMGIC fusion partner-like 2 [Source:HGNC Symbol;Acc:6588]	chr5	77781038	78070844	1674	0.109	1	1	5.481E-03	7.783E-03	PB-CAG-SD
LPP	LIM domain containing preferred translocation partner in lipoma [Source:HGNC Symbol;Acc:6679]	chr3	187866072	188608460	5171	0.349	2	1	5.481E-03	7.783E-03	PB-CAG-SD
NR2F2-AS1	NR2F2 antisense RNA 1 [Source:HGNC Symbol;Acc:44222]	chr15	96670598	96875590	1510	0.111	1	1	5.726E-03	8.113E-03	PB-CAG-SD
TMEM260	transmembrane protein 260 [Source:HGNC Symbol;Acc:20185]	chr14	56950072	57117324	1273	0.112	1	1	5.774E-03	8.165E-03	PB-CAG-SD
GRIK4	glutamate receptor, ionotropic, kainate 4 [Source:HGNC Symbol;Acc:4582]	chr11	120377468	120859613	1947	0.113	1	1	5.923E-03	8.359E-03	PB-CAG-SD
PDZRN3	PDZ domain containing ring finger 3 [Source:HGNC Symbol;Acc:17704]	chr3	73431584	73679091	1695	0.115	1	1	6.077E-03	8.560E-03	PB-CAG-SD
LINC00511	long intergenic non-protein coding RNA 511 [Source:HGNC Symbol;Acc:43564]	chr17	70319264	70641611	1307	0.115	1	1	6.171E-03	8.675E-03	PB-CAG-SD
GPC5	glypican 5 [Source:HGNC Symbol;Acc:4453]	chr13	92045929	93519490	12929	0.715	3	2	6.190E-03	8.683E-03	PB-CAG-SD
CACNB4	calcium channel, voltage-dependent, beta 4 subunit [Source:HGNC Symbol;Acc:1404]	chr2	152689290	152960593	1659	0.116	1	1	6.240E-03	8.737E-03	PB-CAG-SD
RAPGEF5	Rap guanine nucleotide exchange factor (GEF) 5 [Source:HGNC Symbol;Acc:16862]	chr7	22157856	22401763	1851	0.116	1	1	6.263E-03	8.750E-03	PB-CAG-SD
UGGT2	UDP-glucose glycoprotein glucosyltransferase 2 [Source:HGNC Symbol;Acc:15664]	chr13	96453834	96710736	2122	0.117	1	1	6.370E-03	8.882E-03	PB-CAG-SD
ADAMTS17	ADAM metallopeptidase with thrombospondin type 1 motif, 17 [Source:HGNC Symbol;Acc:17109]	chr15	100511794	100887210	1601	0.118	1	1	6.408E-03	8.918E-03	PB-CAG-SD
PDGFC	platelet derived growth factor C [Source:HGNC Symbol;Acc:8801]	chr4	157681606	157897546	2052	0.118	1	1	6.425E-03	8.924E-03	PB-CAG-SD
NDUFAF2	NADH dehydrogenase (ubiquinone) complex I, assembly factor 2 [Source:HGNC Symbol;Acc:28086]	chr5	60235956	60448853	1820	0.118	1	1	6.438E-03	8.924E-03	PB-CAG-SD
EPB41L2	erythrocyte membrane protein band 4.1-like 2 [Source:HGNC Symbol;Acc:3379]	chr6	131160487	131389462	1862	0.119	1	1	6.509E-03	8.986E-03	PB-CAG-SD
TEX9	testis expressed 9 [Source:HGNC Symbol;Acc:29585]	chr15	56531207	56738195	1614	0.119	1	1	6.509E-03	8.986E-03	PB-CAG-SD
RNF180	ring finger protein 180 [Source:HGNC Symbol;Acc:27752]	chr5	63456671	63668696	1842	0.119	1	1	6.588E-03	9.078E-03	PB-CAG-SD
MCPH1	microcephalin 1 [Source:HGNC Symbol;Acc:6954]	chr8	6259113	6501144	1599	0.120	1	1	6.605E-03	9.083E-03	PB-CAG-SD
SFMBT2	Scm-like with four mbt domains 2 [Source:HGNC Symbol;Acc:20256]	chr10	7200586	7458450	1460	0.120	1	1	6.632E-03	9.103E-03	PB-CAG-SD
ANTXR2	anthrax toxin receptor 2 [Source:HGNC Symbol;Acc:21732]	chr4	80822303	81051608	2091	0.120	1	1	6.662E-03	9.126E-03	PB-CAG-SD
PBX3	pre-B-cell leukemia homeobox 3 [Source:HGNC Symbol;Acc:8634]	chr9	128504624	128729656	2049	0.121	1	1	6.720E-03	9.187E-03	PB-CAG-SD
MEF2C	myocyte enhancer factor 2C [Source:HGNC Symbol;Acc:6996]	chr5	88013975	88204922	1881	0.122	1	1	6.859E-03	9.359E-03	PB-CAG-SD
CAST	calpastatin [Source:HGNC Symbol;Acc:1515]	chr5	95855971	96115299	1887	0.122	1	1	6.901E-03	9.398E-03	PB-CAG-SD
APP	amyloid beta (A4) precursor protein [Source:HGNC Symbol;Acc:620]	chr21	27252861	27548446	2276	0.123	1	1	6.939E-03	9.431E-03	PB-CAG-SD
SLC30A8	solute carrier family 30 (zinc transporter), member 8 [Source:HGNC Symbol;Acc:20303]	chr8	117957512	118188953	1645	0.123	1	1	6.974E-03	9.461E-03	PB-CAG-SD
TMTC1	transmembrane and tetratricopeptide repeat containing 1 [Source:HGNC Symbol;Acc:24099]	chr12	29653773	29942692	2157	0.124	1	1	7.040E-03	9.532E-03	PB-CAG-SD
ZNF423	zinc finger protein 423 [Source:HGNC Symbol;Acc:16762]	chr16	49521435	49896830	1299	0.125	1	1	7.167E-03	9.675E-03	PB-CAG-SD
ZSWIM5	zinc finger, SWIM-type containing 5 [Source:HGNC Symbol;Acc:29299]	chr1	45482071	45776881	1801	0.125	1	1	7.173E-03	9.675E-03	PB-CAG-SD
IPO11	importin 11 [Source:HGNC Symbol;Acc:20628]	chr5	61694799	61924409	1939	0.126	1	1	7.270E-03	9.787E-03	PB-CAG-SD
MAP4K3	mitogen-activated protein kinase kinase kinase kinase 3 [Source:HGNC Symbol;Acc:6865]	chr2	39476407	39669453	1799	0.126	1	1	7.291E-03	9.796E-03	PB-CAG-SD
SATB2	SATB homeobox 2 [Source:HGNC Symbol;Acc:21637]	chr2	200134223	200340989	1807	0.126	1	1	7.353E-03	9.860E-03	PB-CAG-SD
ADAMTS18	ADAM metallopeptidase with thrombospondin type 1 motif, 18 [Source:HGNC Symbol;Acc:17110]	chr16	77281710	77474011	1349	0.130	1	1	7.705E-03	1.031E-02	PB-CAG-SD
ITGA9	integrin, alpha 9 [Source:HGNC Symbol;Acc:6145]	chr3	37488606	37865005	1923	0.130	1	1	7.743E-03	1.034E-02	PB-CAG-SD
KHDRBS3	KH domain containing, RNA binding, signal transduction associated 3 [Source:HGNC Symbol;Acc:18117]	chr8	136464700	136668965	1741	0.130	1	1	7.775E-03	1.037E-02	PB-CAG-SD
KAZN	kazrin, periplakin interacting protein [Source:HGNC Symbol;Acc:29173]	chr1	14920200	15444539	1894	0.131	1	1	7.900E-03	1.051E-02	PB-CAG-SD
DIO2	deiodinase, iodothyronine, type II [Source:HGNC Symbol;Acc:2884]	chr14	80663873	80859100	1503	0.132	1	1	7.942E-03	1.055E-02	PB-CAG-SD
DPYD-AS1	DPYD antisense RNA 1 [Source:HGNC Symbol;Acc:40195]	chr1	97556479	97788511	1922	0.133	1	1	8.125E-03	1.075E-02	PB-CAG-SD
PKP4	plakophilin 4 [Source:HGNC Symbol;Acc:9026]	chr2	159308476	159539391	1904	0.133	1	1	8.127E-03	1.075E-02	PB-CAG-SD
DDX10	DEAD (Asp-Glu-Ala-Asp) box polypeptide 10 [Source:HGNC Symbol;Acc:2735]	chr11	108530752	108811657	2303	0.134	1	1	8.174E-03	1.080E-02	PB-CAG-SD
TMEM178B	transmembrane protein 178B [Source:HGNC Symbol;Acc:44112]	chr7	140769032	141180180	2131	0.134	1	1	8.205E-03	1.082E-02	PB-CAG-SD
CPEB3	cytoplasmic polyadenylation element binding protein 3 [Source:HGNC Symbol;Acc:21746]	chr10	93806449	94055844	1633	0.134	1	1	8.220E-03	1.082E-02	PB-CAG-SD
MEIS2	Meis homeobox 2 [Source:HGNC Symbol;Acc:7001]	chr15	37181406	37398504	1837	0.135	1	1	8.341E-03	1.094E-02	PB-CAG-SD
TFEC	transcription factor EC [Source:HGNC Symbol;Acc:11754]	chr7	115575202	115804950	2150	0.135	1	1	8.345E-03	1.094E-02	PB-CAG-SD
NTRK2	neurotrophic tyrosine kinase, receptor, type 2 [Source:HGNC Symbol;Acc:8032]	chr9	87278466	87638505	2320	0.137	1	1	8.525E-03	1.114E-02	PB-CAG-SD
MYO5B	myosin VB [Source:HGNC Symbol;Acc:7603]	chr18	47349183	47726463	2087	0.137	1	1	8.531E-03	1.114E-02	PB-CAG-SD
IQCH	IQ motif containing H [Source:HGNC Symbol;Acc:25721]	chr15	67542138	67794598	1866	0.137	1	1	8.594E-03	1.120E-02	PB-CAG-SD
PTPN4	protein tyrosine phosphatase, non-receptor type 4 (megakaryocyte) [Source:HGNC Symbol;Acc:9656]	chr2	120512207	120741394	1985	0.139	1	1	8.800E-03	1.145E-02	PB-CAG-SD
ZNF704	zinc finger protein 704 [Source:HGNC Symbol;Acc:32291]	chr8	81540686	81792016	1860	0.139	1	1	8.823E-03	1.146E-02	PB-CAG-SD
MGAT5	mannosyl (alpha-1,6-)-glycoprotein beta-1,6-N-acetyl-glucosaminyltransferase [Source:HGNC Symbol;Acc:7049]	chr2	134872554	135212192	2015	0.141	1	1	9.056E-03	1.174E-02	PB-CAG-SD
PKIB	protein kinase (cAMP-dependent, catalytic) inhibitor beta [Source:HGNC Symbol;Acc:9018]	chr6	122788076	123047518	2231	0.142	1	1	9.200E-03	1.189E-02	PB-CAG-SD
RAPGEF2	Rap guanine nucleotide exchange factor (GEF) 2 [Source:HGNC Symbol;Acc:16854]	chr4	160020330	160281321	2476	0.142	1	1	9.206E-03	1.189E-02	PB-CAG-SD
PCNXL2	pecanex-like 2 (Drosophila) [Source:HGNC Symbol;Acc:8736]	chr1	233119881	233436459	2054	0.142	1	1	9.223E-03	1.189E-02	PB-CAG-SD
TLK1	tousled-like kinase 1 [Source:HGNC Symbol;Acc:11841]	chr2	171847333	172092824	2078	0.145	1	1	9.603E-03	1.236E-02	PB-CAG-SD
RNF150	ring finger protein 150 [Source:HGNC Symbol;Acc:23138]	chr4	141786725	142139031	2553	0.147	1	1	9.759E-03	1.253E-02	PB-CAG-SD
MBNL1	muscleblind-like splicing regulator 1 [Source:HGNC Symbol;Acc:6923]	chr3	151956617	152183569	2210	0.149	1	1	1.010E-02	1.294E-02	PB-CAG-SD
NSMCE2	non-SMC element 2, MMS21 homolog (S. cerevisiae) [Source:HGNC Symbol;Acc:26513]	chr8	126098921	126379362	1998	0.149	1	1	1.011E-02	1.294E-02	PB-CAG-SD
EFCAB6	EF-hand calcium binding domain 6 [Source:HGNC Symbol;Acc:24204]	chr22	43924624	44213217	1646	0.151	1	1	1.026E-02	1.311E-02	PB-CAG-SD
CHCHD3	coiled-coil-helix-coiled-coil-helix domain containing 3 [Source:HGNC Symbol;Acc:21906]	chr7	132469629	132771848	2402	0.151	1	1	1.031E-02	1.314E-02	PB-CAG-SD
GABRB2	gamma-aminobutyric acid (GABA) A receptor, beta 2 [Source:HGNC Symbol;Acc:4082]	chr5	160715436	160981050	2350	0.152	1	1	1.049E-02	1.335E-02	PB-CAG-SD
PRICKLE2	prickle homolog 2 (Drosophila) [Source:HGNC Symbol;Acc:20340]	chr3	64079543	64436152	2262	0.153	1	1	1.055E-02	1.341E-02	PB-CAG-SD
DIP2C	DIP2 disco-interacting protein 2 homolog C (Drosophila) [Source:HGNC Symbol;Acc:29150]	chr10	320130	740683	1872	0.154	1	1	1.066E-02	1.352E-02	PB-CAG-SD
CCDC148	coiled-coil domain containing 148 [Source:HGNC Symbol;Acc:25191]	chr2	159027593	159318265	2206	0.154	1	1	1.076E-02	1.362E-02	PB-CAG-SD
PBX1	pre-B-cell leukemia homeobox 1 [Source:HGNC Symbol;Acc:8632]	chr1	164519821	164868533	2238	0.155	1	1	1.086E-02	1.372E-02	PB-CAG-SD
USP34	ubiquitin specific peptidase 34 [Source:HGNC Symbol;Acc:20066]	chr2	61414598	61702904	2230	0.156	1	1	1.098E-02	1.385E-02	PB-CAG-SD
MKL2	MKL/myocardin-like 2 [Source:HGNC Symbol;Acc:29819]	chr16	14160178	14360630	1671	0.161	1	1	1.158E-02	1.458E-02	PB-CAG-SD
ACYP2	acylphosphatase 2, muscle type [Source:HGNC Symbol;Acc:180]	chr2	54192975	54532437	2303	0.161	1	1	1.167E-02	1.467E-02	PB-CAG-SD
TIAM2	T-cell lymphoma invasion and metastasis 2 [Source:HGNC Symbol;Acc:11806]	chr6	155148831	155578857	2556	0.163	1	1	1.191E-02	1.494E-02	PB-CAG-SD
ATG10	autophagy related 10 [Source:HGNC Symbol;Acc:20315]	chr5	81262844	81572676	2530	0.164	1	1	1.207E-02	1.511E-02	PB-CAG-SD
ANGPT1	angiopoietin 1 [Source:HGNC Symbol;Acc:484]	chr8	108261721	108515283	2195	0.164	1	1	1.209E-02	1.511E-02	PB-CAG-SD
DIO2-AS1	DIO2 antisense RNA 1 [Source:HGNC Symbol;Acc:44153]	chr14	80672762	80921812	1884	0.165	1	1	1.221E-02	1.523E-02	PB-CAG-SD
DYNC1I1	dynein, cytoplasmic 1, intermediate chain 1 [Source:HGNC Symbol;Acc:2963]	chr7	95396866	95739634	2640	0.166	1	1	1.233E-02	1.534E-02	PB-CAG-SD
EYA4	eyes absent homolog 4 (Drosophila) [Source:HGNC Symbol;Acc:3522]	chr6	133556736	133853258	2604	0.166	1	1	1.234E-02	1.534E-02	PB-CAG-SD
C3orf67	chromosome 3 open reading frame 67 [Source:HGNC Symbol;Acc:24763]	chr3	58703092	59040810	2473	0.167	1	1	1.250E-02	1.550E-02	PB-CAG-SD
MAML3	mastermind-like 3 (Drosophila) [Source:HGNC Symbol;Acc:16272]	chr4	140637907	141080338	2910	0.167	1	1	1.251E-02	1.550E-02	PB-CAG-SD
CAMK2D	calcium/calmodulin-dependent protein kinase II delta [Source:HGNC Symbol;Acc:1462]	chr4	114372188	114688083	2918	0.168	1	1	1.257E-02	1.555E-02	PB-CAG-SD
STEAP2-AS1	STEAP2 antisense RNA 1 [Source:HGNC Symbol;Acc:40820]	chr7	89511667	89845949	2699	0.170	1	1	1.286E-02	1.587E-02	PB-CAG-SD
HECW1	HECT, C2 and WW domain containing E3 ubiquitin protein ligase 1 [Source:HGNC Symbol;Acc:22195]	chr7	43147198	43602938	2724	0.171	1	1	1.308E-02	1.612E-02	PB-CAG-SD
CSGALNACT1	chondroitin sulfate N-acetylgalactosaminyltransferase 1 [Source:HGNC Symbol;Acc:24290]	chr8	19261672	19620540	2292	0.171	1	1	1.311E-02	1.613E-02	PB-CAG-SD
DISC1	disrupted in schizophrenia 1 [Source:HGNC Symbol;Acc:2888]	chr1	231757561	232177018	2478	0.172	1	1	1.317E-02	1.617E-02	PB-CAG-SD
DGKB	diacylglycerol kinase, beta 90kDa [Source:HGNC Symbol;Acc:2850]	chr7	14184674	15019402	7751	0.487	2	1	1.343E-02	1.646E-02	PB-CAG-SD
DMD	dystrophin [Source:HGNC Symbol;Acc:2928]	chrX	31137336	33362558	19215	0.490	2	1	1.361E-02	1.666E-02	PB-CAG-SD
ZBTB20	zinc finger and BTB domain containing 20 [Source:HGNC Symbol;Acc:13503]	chr3	114056941	114871118	7284	0.492	2	1	1.380E-02	1.685E-02	PB-CAG-SD
GNG12-AS1	GNG12 antisense RNA 1 [Source:HGNC Symbol;Acc:43938]	chr1	68292986	68668670	2552	0.177	1	1	1.392E-02	1.697E-02	PB-CAG-SD
PALLD	palladin, cytoskeletal associated protein [Source:HGNC Symbol;Acc:17068]	chr4	169413217	169849608	3150	0.181	1	1	1.453E-02	1.768E-02	PB-CAG-SD
FHOD3	formin homology 2 domain containing 3 [Source:HGNC Symbol;Acc:26178]	chr18	33872677	34360018	2777	0.182	1	1	1.466E-02	1.782E-02	PB-CAG-SD
PPP3CA	protein phosphatase 3, catalytic subunit, alpha isozyme [Source:HGNC Symbol;Acc:9314]	chr4	101944566	102274435	3207	0.184	1	1	1.502E-02	1.822E-02	PB-CAG-SD
MPP7	membrane protein, palmitoylated 7 (MAGUK p55 subfamily member 7) [Source:HGNC Symbol;Acc:26542]	chr10	28339922	28628415	2246	0.184	1	1	1.504E-02	1.822E-02	PB-CAG-SD
PRIM2	primase, DNA, polypeptide 2 (58kDa) [Source:HGNC Symbol;Acc:9370]	chr6	57174603	57513375	2917	0.186	1	1	1.528E-02	1.848E-02	PB-CAG-SD
PREX2	phosphatidylinositol-3,4,5-trisphosphate-dependent Rac exchange factor 2 [Source:HGNC Symbol;Acc:22950]	chr8	68859353	69149265	2493	0.186	1	1	1.536E-02	1.854E-02	PB-CAG-SD
CNTN4	contactin 4 [Source:HGNC Symbol;Acc:2174]	chr3	2135497	3099645	7597	0.513	2	1	1.541E-02	1.857E-02	PB-CAG-SD
PCDH9	protocadherin 9 [Source:HGNC Symbol;Acc:8661]	chr13	66876967	67809468	9288	0.514	2	2	1.544E-02	1.857E-02	PB-CAG-SD
SPATA5	spermatogenesis associated 5 [Source:HGNC Symbol;Acc:18119]	chr4	123839229	124235809	3315	0.190	1	1	1.599E-02	1.920E-02	PB-CAG-SD
ROR1	receptor tyrosine kinase-like orphan receptor 1 [Source:HGNC Symbol;Acc:10256]	chr1	64234693	64647181	2788	0.193	1	1	1.643E-02	1.970E-02	PB-CAG-SD
PCSK5	proprotein convertase subtilisin/kexin type 5 [Source:HGNC Symbol;Acc:8747]	chr9	78500560	78977255	3291	0.194	1	1	1.652E-02	1.977E-02	PB-CAG-SD
TLN2	talin 2 [Source:HGNC Symbol;Acc:15447]	chr15	62677725	63136830	2674	0.197	1	1	1.697E-02	2.027E-02	PB-CAG-SD
ADAMTS6	ADAM metallopeptidase with thrombospondin type 1 motif, 6 [Source:HGNC Symbol;Acc:222]	chr5	64444563	64782747	3048	0.198	1	1	1.713E-02	2.043E-02	PB-CAG-SD
MCC	mutated in colorectal cancers [Source:HGNC Symbol;Acc:6935]	chr5	112357796	112829527	3059	0.198	1	1	1.725E-02	2.053E-02	PB-CAG-SD
SYNPR	synaptoporin [Source:HGNC Symbol;Acc:16507]	chr3	63208991	63602597	2938	0.198	1	1	1.728E-02	2.053E-02	PB-CAG-SD
RARB	retinoic acid receptor, beta [Source:HGNC Symbol;Acc:9865]	chr3	25210823	25639423	2944	0.199	1	1	1.734E-02	2.054E-02	PB-CAG-SD
COL19A1	collagen, type XIX, alpha 1 [Source:HGNC Symbol;Acc:2196]	chr6	70571463	70919679	3121	0.199	1	1	1.735E-02	2.054E-02	PB-CAG-SD
STAU2	staufen double-stranded RNA binding protein 2 [Source:HGNC Symbol;Acc:11371]	chr8	74332604	74664943	2676	0.200	1	1	1.754E-02	2.074E-02	PB-CAG-SD
THRB	thyroid hormone receptor, beta [Source:HGNC Symbol;Acc:11799]	chr3	24158651	24541773	2983	0.202	1	1	1.778E-02	2.098E-02	PB-CAG-SD
DGKI	diacylglycerol kinase, iota [Source:HGNC Symbol;Acc:2855]	chr7	137073563	137536838	3212	0.202	1	1	1.783E-02	2.101E-02	PB-CAG-SD
SLIT3	slit homolog 3 (Drosophila) [Source:HGNC Symbol;Acc:11087]	chr5	168088745	168733133	3127	0.203	1	1	1.797E-02	2.115E-02	PB-CAG-SD
MAP2	microtubule-associated protein 2 [Source:HGNC Symbol;Acc:6839]	chr2	210283782	210598842	2910	0.204	1	1	1.813E-02	2.129E-02	PB-CAG-SD
AKAP13	A kinase (PRKA) anchor protein 13 [Source:HGNC Symbol;Acc:371]	chr15	85918802	86292586	2780	0.204	1	1	1.825E-02	2.140E-02	PB-CAG-SD
MMP16	matrix metallopeptidase 16 (membrane-inserted) [Source:HGNC Symbol;Acc:7162]	chr8	89044237	89345254	2766	0.207	1	1	1.866E-02	2.184E-02	PB-CAG-SD
DPYD	dihydropyrimidine dehydrogenase [Source:HGNC Symbol;Acc:3012]	chr1	97543299	98391605	8004	0.555	2	1	1.889E-02	2.207E-02	PB-CAG-SD
BICC1	bicaudal C homolog 1 (Drosophila) [Source:HGNC Symbol;Acc:19351]	chr10	60267900	60591195	2540	0.208	1	1	1.894E-02	2.209E-02	PB-CAG-SD

table S1. Genes with significant transposon integrations in DLD-1 *KRAS*$^{wt/-}$ cells after selection in low glucose medium.

Gene ID	Description	Chrom	Start	End	# TTAA sites	Expected	Observed	Libraries	*P*	FDR	Transposon
STAG1	stromal antigen 1 [Source:HGNC Symbol;Acc:11354]	chr3	136055077	136476220	3111	0.210	1	1	1.922E-02	2.239E-02	PB-CAG-SD
MYT1L	myelin transcription factor 1-like [Source:HGNC Symbol;Acc:7623]	chr2	1792885	2340032	3020	0.211	1	1	1.942E-02	2.259E-02	PB-CAG-SD
ZNF385D	zinc finger protein 385D [Source:HGNC Symbol;Acc:26191]	chr3	21459915	22419812	8331	0.563	2	1	1.960E-02	2.276E-02	PB-CAG-SD
INADL	InaD-like (Drosophila) [Source:HGNC Symbol;Acc:28881]	chr1	62203149	62629592	3073	0.213	1	1	1.971E-02	2.284E-02	PB-CAG-SD
DYNC2H1	dynein, cytoplasmic 2, heavy chain 1 [Source:HGNC Symbol;Acc:2962]	chr11	102975160	103350591	3691	0.214	1	1	1.991E-02	2.304E-02	PB-CAG-SD
DYM	dymeclin [Source:HGNC Symbol;Acc:21317]	chr18	46570039	46992717	3301	0.216	1	1	2.026E-02	2.338E-02	PB-CAG-SD
ESR1	estrogen receptor 1 [Source:HGNC Symbol;Acc:3467]	chr6	151972826	152450754	3393	0.216	1	1	2.027E-02	2.338E-02	PB-CAG-SD
GLIS3	GLIS family zinc finger 3 [Source:HGNC Symbol;Acc:28510]	chr9	3824127	4353392	3677	0.217	1	1	2.032E-02	2.339E-02	PB-CAG-SD
XKR4	XK, Kell blood group complex subunit-related family, member 4 [Source:HGNC Symbol;Acc:29394]	chr8	56009949	56438714	2920	0.218	1	1	2.064E-02	2.372E-02	PB-CAG-SD
AGAP1	ArfGAP with GTPase domain, ankyrin repeat and PH domain 1 [Source:HGNC Symbol;Acc:16922]	chr2	236397733	237035198	3124	0.219	1	1	2.069E-02	2.372E-02	PB-CAG-SD
SYN3	synapsin III [Source:HGNC Symbol;Acc:11496]	chr22	32908539	33459358	2391	0.219	1	1	2.070E-02	2.372E-02	PB-CAG-SD
FBN2	fibrillin 2 [Source:HGNC Symbol;Acc:3604]	chr5	127593601	127999878	3412	0.221	1	1	2.114E-02	2.418E-02	PB-CAG-SD
BNC2	basonuclin 2 [Source:HGNC Symbol;Acc:30988]	chr9	16409501	16875841	3783	0.223	1	1	2.142E-02	2.446E-02	PB-CAG-SD
MYO3B	myosin IIIB [Source:HGNC Symbol;Acc:15576]	chr2	171029655	171511681	3207	0.224	1	1	2.172E-02	2.476E-02	PB-CAG-SD
PTPRN2	protein tyrosine phosphatase, receptor type, N polypeptide 2 [Source:HGNC Symbol;Acc:9677]	chr7	157331750	158385480	3581	0.225	1	1	2.182E-02	2.484E-02	PB-CAG-SD
TUSC3	tumor suppressor candidate 3 [Source:HGNC Symbol;Acc:30242]	chr8	15269724	15624158	3028	0.226	1	1	2.208E-02	2.509E-02	PB-CAG-SD
PCDH7	protocadherin 7 [Source:HGNC Symbol;Acc:8659]	chr4	30717037	31148422	3978	0.229	1	1	2.245E-02	2.547E-02	PB-CAG-SD
GALNTL6	UDP-N-acetyl-alpha-D-galactosamine:polypeptide N-acetylgalactosaminyltransferase-like 6 [Source:HGNC Symbol;Acc:33844]	chr4	172728405	173962710	10348	0.594	2	1	2.257E-02	2.557E-02	PB-CAG-SD
TRAPPC9	trafficking protein particle complex 9 [Source:HGNC Symbol;Acc:30832]	chr8	140742586	141473678	3094	0.231	1	1	2.298E-02	2.598E-02	PB-CAG-SD
SLC4A10	solute carrier family 4, sodium bicarbonate transporter, member 10 [Source:HGNC Symbol;Acc:13811]	chr2	162475845	162841792	3345	0.234	1	1	2.348E-02	2.651E-02	PB-CAG-SD
CACNB2	calcium channel, voltage-dependent, beta 2 subunit [Source:HGNC Symbol;Acc:1402]	chr10	18424606	18830798	2857	0.235	1	1	2.355E-02	2.655E-02	PB-CAG-SD
SLC8A1-AS1	SLC8A1 antisense RNA 1 [Source:HGNC Symbol;Acc:44102]	chr2	40008593	40482349	3402	0.238	1	1	2.422E-02	2.726E-02	PB-CAG-SD
CCDC171	coiled-coil domain containing 171 [Source:HGNC Symbol;Acc:29828]	chr9	15547895	16061661	4070	0.240	1	1	2.452E-02	2.755E-02	PB-CAG-SD
PHACTR1	phosphatase and actin regulator 1 [Source:HGNC Symbol;Acc:20990]	chr6	12712893	13288645	3857	0.246	1	1	2.569E-02	2.882E-02	PB-CAG-SD
PDE11A	phosphodiesterase 11A [Source:HGNC Symbol;Acc:8773]	chr2	178492797	178978066	3549	0.248	1	1	2.618E-02	2.930E-02	PB-CAG-SD
NEBL	nebulette [Source:HGNC Symbol;Acc:16932]	chr10	21068902	21468116	3027	0.248	1	1	2.620E-02	2.930E-02	PB-CAG-SD
ZNF385B	zinc finger protein 385B [Source:HGNC Symbol;Acc:26332]	chr2	180306709	180731232	3597	0.252	1	1	2.684E-02	2.996E-02	PB-CAG-SD
ITFG1	integrin alpha FG-GAP repeat containing 1 [Source:HGNC Symbol;Acc:30697]	chr16	47188298	47503060	2628	0.252	1	1	2.698E-02	3.007E-02	PB-CAG-SD
SLC27A6	solute carrier family 27 (fatty acid transporter), member 6 [Source:HGNC Symbol;Acc:11000]	chr5	127868706	128369335	3928	0.255	1	1	2.742E-02	3.051E-02	PB-CAG-SD
HYDIN	HYDIN, axonemal central pair apparatus protein [Source:HGNC Symbol;Acc:19368]	chr16	70841281	71269625	2691	0.259	1	1	2.818E-02	3.131E-02	PB-CAG-SD
SYNE1	spectrin repeat containing, nuclear envelope 1 [Source:HGNC Symbol;Acc:17089]	chr6	152442819	152963936	4072	0.260	1	1	2.838E-02	3.148E-02	PB-CAG-SD
HCN1	hyperpolarization activated cyclic nucleotide-gated potassium channel 1 [Source:HGNC Symbol;Acc:4845]	chr5	45259349	45701253	4107	0.266	1	1	2.974E-02	3.295E-02	PB-CAG-SD
PTH2R	parathyroid hormone 2 receptor [Source:HGNC Symbol;Acc:9609]	chr2	209219438	209719227	3940	0.276	1	1	3.170E-02	3.506E-02	PB-CAG-SD
EDIL3	EGF-like repeats and discoidin I-like domains 3 [Source:HGNC Symbol;Acc:3173]	chr5	83236373	83685611	4298	0.279	1	1	3.231E-02	3.568E-02	PB-CAG-SD
ALK	anaplastic lymphoma receptor tyrosine kinase [Source:HGNC Symbol;Acc:427]	chr2	29415640	30149432	4001	0.280	1	1	3.260E-02	3.594E-02	PB-CAG-SD
SIPA1L1	signal-induced proliferation-associated 1 like 1 [Source:HGNC Symbol;Acc:20284]	chr14	71782166	72207946	3204	0.281	1	1	3.274E-02	3.603E-02	PB-CAG-SD
ST6GALNAC3	ST6 (alpha-N-acetyl-neuraminyl-2,3-beta-galactosyl-1,3)-N-acetylgalactosaminide alpha-2,6-sialyltransferase 3 [Source:HGNC Symbol;Acc:19343]	chr1	76535404	77100286	4114	0.285	1	1	3.370E-02	3.702E-02	PB-CAG-SD
KCNH5	potassium voltage-gated channel, subfamily H (eag-related), member 5 [Source:HGNC Symbol;Acc:6254]	chr14	63173287	63573755	3258	0.285	1	1	3.374E-02	3.702E-02	PB-CAG-SD
RGS7	regulator of G-protein signaling 7 [Source:HGNC Symbol;Acc:10003]	chr1	240931554	241525530	4242	0.294	1	1	3.562E-02	3.902E-02	PB-CAG-SD
SLC9A9	solute carrier family 9, subfamily A (NHE9, cation proton antiporter 9), member 9 [Source:HGNC Symbol;Acc:20653]	chr3	142984064	143572373	4374	0.296	1	1	3.594E-02	3.931E-02	PB-CAG-SD
MAST4	microtubule associated serine/threonine kinase family member 4 [Source:HGNC Symbol;Acc:19037]	chr5	65887176	66465423	4572	0.296	1	1	3.615E-02	3.947E-02	PB-CAG-SD
NRG3	neuregulin 3 [Source:HGNC Symbol;Acc:7999]	chr10	83630070	84746935	8823	0.724	2	2	3.716E-02	4.051E-02	PB-CAG-SD
CDH8	cadherin 8, type 2 [Source:HGNC Symbol;Acc:1767]	chr16	61681146	62075939	3150	0.303	1	1	3.752E-02	4.084E-02	PB-CAG-SD
TANC2	tetratricopeptide repeat, ankyrin repeat and coiled-coil containing 2 [Source:HGNC Symbol;Acc:30212]	chr17	61081917	61505060	3442	0.304	1	1	3.783E-02	4.112E-02	PB-CAG-SD
LARGE	like-glycosyltransferase [Source:HGNC Symbol;Acc:6511]	chr22	33668847	34323829	3368	0.308	1	1	3.875E-02	4.206E-02	PB-CAG-SD
KCNH7	potassium voltage-gated channel, subfamily H (eag-related), member 7 [Source:HGNC Symbol;Acc:18863]	chr2	163227917	163700240	4495	0.315	1	1	4.023E-02	4.359E-02	PB-CAG-SD
PDE4B	phosphodiesterase 4B, cAMP-specific [Source:HGNC Symbol;Acc:8781]	chr1	66253197	66840259	4562	0.316	1	1	4.061E-02	4.394E-02	PB-CAG-SD
FGF14	fibroblast growth factor 14 [Source:HGNC Symbol;Acc:3671]	chr13	102372134	103059124	5774	0.319	1	1	4.132E-02	4.464E-02	PB-CAG-SD
MEF2C-AS1	MEF2C antisense RNA 1 [Source:HGNC Symbol;Acc:48908]	chr5	88174145	88762215	5171	0.335	1	1	4.509E-02	4.863E-02	PB-CAG-SD
ULK4	unc-51 like kinase 4 [Source:HGNC Symbol;Acc:15784]	chr3	41288090	42008922	4978	0.336	1	1	4.534E-02	4.883E-02	PB-CAG-SD
PTPRK	protein tyrosine phosphatase, receptor type, K [Source:HGNC Symbol;Acc:9674]	chr6	128289924	128846870	5285	0.337	1	1	4.546E-02	4.889E-02	PB-CAG-SD
RGS6	regulator of G-protein signaling 6 [Source:HGNC Symbol;Acc:10002]	chr14	72394156	73030654	3868	0.339	1	1	4.594E-02	4.932E-02	PB-CAG-SD

table S1. Genes with significant transposon integrations in DLD-1 *KRAS*$^{wt/-}$ cells after selection in low glucose medium. Two libraries were generated by mutagenesis with gene trap (PB-GT) and two by promoter containing (PB-CAG-SD) transposon constructs followed by serial selection in low glucose and hygromycin**.** Gene IDs, descriptions and chromosomal locations were obtained from Ensembl. The number of TTAA sites corresponds to intronic TTAA sites in case of PB-GT and also includes TTAA sites within 5 kB of the gene for PB-CAG-SD transposon libraries. Expected, the number of expected random integrations; Observed, the number of independent integration sites observed by sequencing of splinkerette PCR products from the libraries; Libraries, number of libraries (out of 2) containing integrations in the gene; P, P value calculated using Poisson statistics, FDR, False Discovery Rate correction for multiple testing. An FDR adjusted P value <0.05 was used as cutoff for selecting significant genes.

table S2. Genes with significant transposon integrations in RKO *BRAF*$^{wt/-/-}$ cells after selection in low glucose medium

Gene ID	Description	Chrom	Start	End	# TTAA sites	Expected	Observed	Libraries	*P*	FDR	Transposon
SFTA1P	surfactant associated 1, pseudogene [Source:HGNC Symbol;Acc:18383]	chr10	10826400	10836943	79	0.028	6	1	2.665E-15	7.629E-12	PB-GT
ZNF271	zinc finger protein 271 [Source:HGNC Symbol;Acc:13065]	chr18	32870246	32890730	130	0.031	6	2	5.440E-15	7.787E-12	PB-GT
VMP1	vacuole membrane protein 1 [Source:HGNC Symbol;Acc:29559]	chr17	57784553	57919616	1005	0.509	12	2	1.554E-14	1.483E-11	PB-GT
LINC00472	long intergenic non-protein coding RNA 472 [Source:HGNC Symbol;Acc:21380]	chr6	72054047	72130472	706	0.243	9	2	1.555E-13	1.113E-10	PB-GT
RRP15	ribosomal RNA processing 15 homolog (S. cerevisiae) [Source:HGNC Symbol;Acc:24255]	chr1	218458629	218511325	476	0.169	7	2	1.419E-11	8.124E-09	PB-GT
DLGAP1-AS2	DLGAP1 antisense RNA 2 [Source:HGNC Symbol;Acc:28146]	chr18	3602998	3608334	29	0.007	3	2	9.643E-11	4.601E-08	PB-GT
CRIM1	cysteine rich transmembrane BMP regulator 1 (chordin-like) [Source:HGNC Symbol;Acc:2359]	chr2	36583069	36778278	1464	0.419	8	2	7.476E-10	3.058E-07	PB-GT
ELOVL1	ELOVL fatty acid elongase 1 [Source:HGNC Symbol;Acc:14418]	chr1	43829068	43833696	5	0.002	2	1	9.307E-10	3.331E-07	PB-GT
FEZ2	fasciculation and elongation protein zeta 2 (zygin II) [Source:HGNC Symbol;Acc:3660]	chr2	36778570	36873230	675	0.193	6	2	1.674E-09	4.559E-07	PB-GT
DCLK1	doublecortin-like kinase 1 [Source:HGNC Symbol;Acc:2700]	chr13	36345478	36705443	2630	0.639	9	2	1.740E-09	4.559E-07	PB-GT
PABPC1	poly(A) binding protein, cytoplasmic 1 [Source:HGNC Symbol;Acc:8554]	chr8	101698044	101735037	308	0.106	5	2	1.752E-09	4.559E-07	PB-GT
HOXB8	homeobox B8 [Source:HGNC Symbol;Acc:5119]	chr17	46688739	46692478	6	0.003	2	2	4.668E-09	1.114E-06	PB-GT
EIF3J	eukaryotic translation initiation factor 3, subunit J [Source:HGNC Symbol;Acc:3270]	chr15	44829255	44855227	175	0.061	4	2	6.614E-09	1.457E-06	PB-GT
AVPR2	arginine vasopressin receptor 2 [Source:HGNC Symbol;Acc:897]	chrX	153167985	153172620	1	0.000	1	1	1.388E-08	2.584E-06	PB-GT
RNA28S5	RNA, 28S ribosomal 5 [Source:HGNC Symbol;Acc:37659]	chrX	108297361	108297792	1	0.000	1	1	1.388E-08	2.584E-06	PB-GT
SET	SET nuclear oncogene [Source:HGNC Symbol;Acc:10760]	chr9	131445703	131458679	65	0.024	3	2	1.444E-08	2.584E-06	PB-GT
ADAMTSL1	ADAMTS-like 1 [Source:HGNC Symbol;Acc:14632]	chr9	18473892	18910948	3065	1.150	10	2	4.066E-08	6.848E-06	PB-GT
HMGN2	high mobility group nucleosomal binding domain 2 [Source:HGNC Symbol;Acc:4986]	chr1	26798941	26802463	20	0.007	2	1	5.933E-08	9.070E-06	PB-GT
NUDT8	nudix (nucleoside diphosphate linked moiety X)-type motif 8 [Source:HGNC Symbol;Acc:8055]	chr11	67395409	67397401	1	0.000	1	1	6.019E-08	9.070E-06	PB-GT
PCNPP3	PEST containing nuclear protein pseudogene 3 [Source:HGNC Symbol;Acc:41975]	chr12	66039772	66040242	1	0.000	1	1	7.327E-08	1.028E-05	PB-GT
SETD5-AS1	SETD5 antisense RNA 1 [Source:HGNC Symbol;Acc:44478]	chr3	9391373	9440263	324	0.100	4	2	7.544E-08	1.028E-05	PB-GT
BSG	basigin (Ok blood group) [Source:HGNC Symbol;Acc:1116]	chr19	571297	583493	15	0.008	2	2	8.216E-08	1.069E-05	PB-GT
RPLP0	ribosomal protein, large, P0 [Source:HGNC Symbol;Acc:10371]	chr12	120634489	120639038	22	0.008	2	1	9.897E-08	1.188E-05	PB-GT
ZNF397	zinc finger protein 397 [Source:HGNC Symbol;Acc:18818]	chr18	32820994	32847097	166	0.040	3	2	1.008E-07	1.188E-05	PB-GT
LYPD6B	LY6/PLAUR domain containing 6B [Source:HGNC Symbol;Acc:27018]	chr2	149894621	150071776	1242	0.355	6	2	1.038E-07	1.188E-05	PB-GT
RNA5-8SP4	RNA, 5.8S ribosomal pseudogene 4 [Source:HGNC Symbol;Acc:41958]	chr19	24187160	24187309	1	0.001	1	1	1.392E-07	1.532E-05	PB-GT
LAMTOR3	late endosomal/lysosomal adaptor, MAPK and MTOR activator 3 [Source:HGNC Symbol;Acc:15606]	chr4	100799493	100815647	162	0.046	3	2	1.778E-07	1.885E-05	PB-GT
EIF4A2P4	eukaryotic translation initiation factor 4A2 pseudogene 4 [Source:HGNC Symbol;Acc:45101]	chrX	52861754	52862949	4	0.001	1	1	2.220E-07	2.270E-05	PB-GT
ASB9P1	ankyrin repeat and SOCS box containing 9 pseudogene 1 [Source:HGNC Symbol;Acc:20972]	chr15	93338877	93339669	2	0.001	1	1	2.418E-07	2.322E-05	PB-GT
ATAD2	ATPase family, AAA domain containing 2 [Source:HGNC Symbol;Acc:30123]	chr8	124332090	124428590	715	0.245	5	2	2.434E-07	2.322E-05	PB-GT
RNY5	RNA, Ro-associated Y5 [Source:HGNC Symbol;Acc:10248]	chr7	148638580	148638658	2	0.001	1	1	2.756E-07	2.546E-05	PB-GT
USP11	ubiquitin specific peptidase 11 [Source:HGNC Symbol;Acc:12609]	chrX	47092089	47107727	72	0.012	2	2	2.852E-07	2.551E-05	PB-GT
HOXA9	homeobox A9 [Source:HGNC Symbol;Acc:5109]	chr7	27202054	27210117	34	0.013	2	1	3.323E-07	2.883E-05	PB-GT
THUMPD3	THUMP domain containing 3 [Source:HGNC Symbol;Acc:24493]	chr3	9404526	9428475	177	0.054	3	2	3.506E-07	2.952E-05	PB-GT
PDE7B	phosphodiesterase 7B [Source:HGNC Symbol;Acc:8792]	chr6	136172834	136516712	2575	0.885	8	2	4.134E-07	3.381E-05	PB-GT
HIST2H2BC	histone cluster 2, H2bc (pseudogene) [Source:HGNC Symbol;Acc:20516]	chr1	149821760	149822339	3	0.001	1	1	5.667E-07	4.385E-05	PB-GT
ENO1-AS1	ENO1 antisense RNA 1 [Source:HGNC Symbol;Acc:40214]	chr1	8938894	8939953	3	0.001	1	1	5.667E-07	4.385E-05	PB-GT
BRWD1-IT2	BRWD1 intronic transcript 2 (non-protein coding) [Source:HGNC Symbol;Acc:16423]	chr21	40685887	40686886	4	0.001	1	1	6.544E-07	4.931E-05	PB-GT
LYPLAL1	lysophospholipase-like 1 [Source:HGNC Symbol;Acc:20440]	chr1	219347186	219386207	450	0.160	4	2	7.590E-07	5.572E-05	PB-GT
EIF4A2	eukaryotic translation initiation factor 4A2 [Source:HGNC Symbol;Acc:3284]	chr3	186500994	186507689	57	0.018	2	1	8.868E-07	6.114E-05	PB-GT
MAGIX	MAGI family member, X-linked [Source:HGNC Symbol;Acc:30006]	chrX	49019061	49024822	8	0.001	1	1	8.875E-07	6.114E-05	PB-GT
MT1X	metallothionein 1X [Source:HGNC Symbol;Acc:7405]	chr16	56716336	56718108	4	0.001	1	1	8.969E-07	6.114E-05	PB-GT
ZNF123P	zinc finger protein 123, pseudogene [Source:HGNC Symbol;Acc:12906]	chr11	129165181	129166376	4	0.001	1	1	9.624E-07	6.328E-05	PB-GT
DNAJC9	DnaJ (Hsp40) homolog, subfamily C, member 9 [Source:HGNC Symbol;Acc:19123]	chr10	74943120	75008620	471	0.168	4	2	9.724E-07	6.328E-05	PB-GT
PFKM	phosphofructokinase, muscle [Source:HGNC Symbol;Acc:8877]	chr12	48498922	48540187	186	0.071	3	1	1.012E-06	6.440E-05	PB-GT
MCOLN3	mucolipin 3 [Source:HGNC Symbol;Acc:13358]	chr1	85483765	85514182	204	0.072	3	2	1.082E-06	6.731E-05	PB-GT
RPS19	ribosomal protein S19 [Source:HGNC Symbol;Acc:10402]	chr19	42363988	42376994	36	0.019	2	1	1.126E-06	6.740E-05	PB-GT
RPL17P43	ribosomal protein L17 pseudogene 43 [Source:HGNC Symbol;Acc:35996]	chr17	19400256	19400841	3	0.002	1	1	1.153E-06	6.740E-05	PB-GT
RNASEK	ribonuclease, RNase K [Source:HGNC Symbol;Acc:33911]	chr17	6915736	6917851	3	0.002	1	1	1.153E-06	6.740E-05	PB-GT
UCA1	urothelial cancer associated 1 (non-protein coding) [Source:HGNC Symbol;Acc:37126]	chr19	15939757	15947130	37	0.020	2	2	1.222E-06	6.999E-05	PB-GT
TTI2	TELO2 interacting protein 2 [Source:HGNC Symbol;Acc:26262]	chr8	33330904	33371119	232	0.079	3	1	1.561E-06	8.761E-05	PB-GT
LINC00102	long intergenic non-protein coding RNA 102 [Source:HGNC Symbol;Acc:30470]	chrX	2531029	2533388	11	0.002	1	1	1.677E-06	8.856E-05	PB-GT
WDR85	WD repeat domain 85 [Source:HGNC Symbol;Acc:25199]	chr9	140449356	140473387	58	0.022	2	1	1.688E-06	8.856E-05	PB-GT
CKS2	CDC28 protein kinase regulatory subunit 2 [Source:HGNC Symbol;Acc:2000]	chr9	91926113	91931618	58	0.022	2	2	1.688E-06	8.856E-05	PB-GT
HYAL2	hyaluronoglucosaminidase 2 [Source:HGNC Symbol;Acc:5321]	chr3	50355221	50360337	6	0.002	1	1	1.701E-06	8.856E-05	PB-GT
ATP5A1P7	ATP synthase, H+ transporting, mitochondrial F1 complex, alpha subunit 1 pseudogene 7 [Source:HGNC Symbol;Acc:37665]	chr9	46998213	46999491	5	0.002	1	1	1.756E-06	8.980E-05	PB-GT
TEX29	testis expressed 29 [Source:HGNC Symbol;Acc:20370]	chr13	111968531	111996596	93	0.023	2	2	1.886E-06	9.475E-05	PB-GT
CHCHD10	coiled-coil-helix-coiled-coil-helix domain containing 10 [Source:HGNC Symbol;Acc:15559]	chr22	24108021	24110630	4	0.002	1	1	1.994E-06	9.844E-05	PB-GT
CDRT7	CMT1A duplicated region transcript 7 (non-protein coding) [Source:HGNC Symbol;Acc:14386]	chr17	14934292	14935274	4	0.002	1	1	2.050E-06	9.919E-05	PB-GT
PIGA	phosphatidylinositol glycan anchor biosynthesis, class A [Source:HGNC Symbol;Acc:8957]	chrX	15337573	15353676	140	0.023	2	1	2.079E-06	9.919E-05	PB-GT
OR2U1P	olfactory receptor, family 2, subfamily U, member 1 pseudogene [Source:HGNC Symbol;Acc:8278]	chr6	29230480	29231856	6	0.002	1	1	2.121E-06	9.955E-05	PB-GT
TUSC2	tumor suppressor candidate 2 [Source:HGNC Symbol;Acc:17034]	chr3	50357458	50365682	7	0.002	1	1	2.315E-06	1.048E-04	PB-GT
LINC00526	long intergenic non-protein coding RNA 526 [Source:HGNC Symbol;Acc:28278]	chr18	5236723	5238597	9	0.002	1	1	2.320E-06	1.048E-04	PB-GT
WBP5	WW domain binding protein 5 [Source:HGNC Symbol;Acc:30084]	chrX	102611373	102613397	13	0.002	1	1	2.342E-06	1.048E-04	PB-GT
PDSS1	prenyl (decaprenyl) diphosphate synthase, subunit 1 [Source:HGNC Symbol;Acc:17759]	chr10	26986588	27035727	261	0.093	3	1	2.912E-06	1.283E-04	PB-GT
PABPC4	poly(A) binding protein, cytoplasmic 4 (inducible form) [Source:HGNC Symbol;Acc:8557]	chr1	40026488	40042462	74	0.026	2	2	2.962E-06	1.285E-04	PB-GT
C1orf229	chromosome 1 open reading frame 229 [Source:HGNC Symbol;Acc:33759]	chr1	247273462	247275719	7	0.002	1	1	3.082E-06	1.311E-04	PB-GT
C22orf15	chromosome 22 open reading frame 15 [Source:HGNC Symbol;Acc:15558]	chr22	24105208	24108048	5	0.002	1	1	3.115E-06	1.311E-04	PB-GT
EXOSC8	exosome component 8 [Source:HGNC Symbol;Acc:17035]	chr13	37572953	37583750	114	0.028	2	2	3.461E-06	1.421E-04	PB-GT
ALKBH6	alkB, alkylation repair homolog 6 (E. coli) [Source:HGNC Symbol;Acc:28243]	chr19	36500022	36505141	5	0.003	1	1	3.474E-06	1.421E-04	PB-GT
TBX6	T-box 6 [Source:HGNC Symbol;Acc:11605]	chr16	30097114	30103208	8	0.003	1	1	3.585E-06	1.445E-04	PB-GT
GPNMB	glycoprotein (transmembrane) nmb [Source:HGNC Symbol;Acc:4462]	chr7	23275586	23314727	270	0.100	3	2	3.886E-06	1.545E-04	PB-GT
MIS18A-AS1	MIS18A antisense RNA 1 [Source:HGNC Symbol;Acc:40106]	chr21	33650174	33653299	10	0.003	1	1	4.086E-06	1.602E-04	PB-GT
RBM6	RNA binding motif protein 6 [Source:HGNC Symbol;Acc:9903]	chr3	49977440	50137478	748	0.230	4	1	4.440E-06	1.718E-04	PB-GT
NCBP2-AS1	NCBP2 antisense RNA 1 [Source:HGNC Symbol;Acc:40392]	chr3	196666748	196669405	10	0.003	1	1	4.722E-06	1.759E-04	PB-GT
IFRD2	interferon-related developmental regulator 2 [Source:HGNC Symbol;Acc:5457]	chr3	50325163	50330349	10	0.003	1	1	4.722E-06	1.759E-04	PB-GT
RRS1	RRS1 ribosome biogenesis regulator homolog (S. cerevisiae) [Source:HGNC Symbol;Acc:17083]	chr8	67341263	67342966	9	0.003	1	1	4.744E-06	1.759E-04	PB-GT
ECT2	epithelial cell transforming sequence 2 oncogene [Source:HGNC Symbol;Acc:3155]	chr3	172468472	172539264	760	0.234	4	2	4.793E-06	1.759E-04	PB-GT
INO80B	INO80 complex subunit B [Source:HGNC Symbol;Acc:13324]	chr2	74682150	74688011	11	0.003	1	1	4.937E-06	1.789E-04	PB-GT
ID3	inhibitor of DNA binding 3, dominant negative helix-loop-helix protein [Source:HGNC Symbol;Acc:5362]	chr1	23884409	23886285	9	0.003	1	1	5.093E-06	1.823E-04	PB-GT
REPIN1	replication initiator 1 [Source:HGNC Symbol;Acc:17922]	chr7	150065278	150071133	9	0.003	1	1	5.572E-06	1.970E-04	PB-GT
NICN1	nicolin 1 [Source:HGNC Symbol;Acc:18317]	chr3	49460379	49466759	11	0.003	1	1	5.712E-06	1.987E-04	PB-GT
PPID	peptidylprolyl isomerase D [Source:HGNC Symbol;Acc:9257]	chr4	159630286	159644548	116	0.033	2	2	5.761E-06	1.987E-04	PB-GT
FBXL22	F-box and leucine-rich repeat protein 22 [Source:HGNC Symbol;Acc:27537]	chr15	63889552	63894627	10	0.003	1	1	6.033E-06	2.047E-04	PB-GT
C2orf69	chromosome 2 open reading frame 69 [Source:HGNC Symbol;Acc:26799]	chr2	200775979	200820658	393	0.112	3	1	6.077E-06	2.047E-04	PB-GT
MALAT1	metastasis associated lung adenocarcinoma transcript 1 (non-protein coding) [Source:HGNC Symbol;Acc:29665]	chr11	65265233	65273940	97	0.034	2	2	6.197E-06	2.060E-04	PB-GT
RNASEK-C17orf4	RNASEK-C17orf49 readthrough (non-protein coding) [Source:HGNC Symbol;Acc:44419]	chr17	6915954	6920839	7	0.004	1	1	6.272E-06	2.060E-04	PB-GT
C14orf182	chromosome 14 open reading frame 182 [Source:HGNC Symbol;Acc:27503]	chr14	50448430	50474238	125	0.034	2	2	6.331E-06	2.060E-04	PB-GT
UCKL1	uridine-cytidine kinase 1-like 1 [Source:HGNC Symbol;Acc:15938]	chr20	62571186	62587769	10	0.004	1	1	6.549E-06	2.107E-04	PB-GT
RPL27A	ribosomal protein L27a [Source:HGNC Symbol;Acc:10329]	chr11	8703958	8736306	101	0.035	2	2	6.989E-06	2.223E-04	PB-GT
NUDT10	nudix (nucleoside diphosphate linked moiety X)-type motif 10 [Source:HGNC Symbol;Acc:17621]	chrX	51075083	51080377	23	0.004	1	1	7.324E-06	2.304E-04	PB-GT
ARHGEF10L	Rho guanine nucleotide exchange factor (GEF) 10-like [Source:HGNC Symbol;Acc:25540]	chr1	17866330	18024369	337	0.120	3	2	7.757E-06	2.414E-04	PB-GT
LARP1	La ribonucleoprotein domain family, member 1 [Source:HGNC Symbol;Acc:29531]	chr5	154092462	154197167	454	0.122	3	2	8.468E-06	2.583E-04	PB-GT
IRS1	insulin receptor substrate 1 [Source:HGNC Symbol;Acc:6125]	chr2	227596757	227664475	428	0.122	3	2	8.480E-06	2.583E-04	PB-GT
TRIM13	tripartite motif containing 13 [Source:HGNC Symbol;Acc:9976]	chr13	50570024	50594617	158	0.038	2	2	9.141E-06	2.755E-04	PB-GT
DOK3	docking protein 3 [Source:HGNC Symbol;Acc:24583]	chr5	176928908	176938275	16	0.004	1	1	9.270E-06	2.765E-04	PB-GT
PRSS36	protease, serine, 36 [Source:HGNC Symbol;Acc:26906]	chr16	31150246	31161415	13	0.004	1	1	9.455E-06	2.791E-04	PB-GT
ZNHIT1	zinc finger, HIT-type containing 1 [Source:HGNC Symbol;Acc:21688]	chr7	100860949	100867471	12	0.004	1	1	9.899E-06	2.892E-04	PB-GT
MORF4L2-AS1	MORF4L2 antisense RNA 1 [Source:HGNC Symbol;Acc:27991]	chrX	102942212	102946700	27	0.004	1	1	1.009E-05	2.917E-04	PB-GT
MIR4313-2	microRNA 4313 [Source:HGNC Symbol;Acc:38310]	chr15	76051842	76054746	13	0.005	1	1	1.019E-05	2.917E-04	PB-GT
TM4SF19-AS1	TM4SF19 antisense RNA 1 [Source:HGNC Symbol;Acc:41085]	chr3	196045201	196052441	15	0.005	1	1	1.061E-05	2.980E-04	PB-GT
JUN	jun proto-oncogene [Source:HGNC Symbol;Acc:6204]	chr1	59246465	59249785	13	0.005	1	1	1.062E-05	2.980E-04	PB-GT
IPO5	importin 5 [Source:HGNC Symbol;Acc:6402]	chr13	98605912	98676551	538	0.131	3	2	1.093E-05	3.038E-04	PB-GT
VPS37C	vacuolar protein sorting 37 homolog C (S. cerevisiae) [Source:HGNC Symbol;Acc:26097]	chr11	60897728	60929089	118	0.041	2	2	1.110E-05	3.055E-04	PB-GT
TSPYL4	TSPY-like 4 [Source:HGNC Symbol;Acc:21559]	chr6	116571151	116575261	14	0.005	1	1	1.153E-05	3.119E-04	PB-GT
TIGD2	tigger transposable element derived 2 [Source:HGNC Symbol;Acc:18333]	chr4	90033968	90036050	17	0.005	1	1	1.155E-05	3.119E-04	PB-GT
DUSP8	dual specificity phosphatase 8 [Source:HGNC Symbol;Acc:3074]	chr11	1575274	1593150	14	0.005	1	1	1.176E-05	3.141E-04	PB-GT
SNHG7	small nucleolar RNA host gene 7 (non-protein coding) [Source:HGNC Symbol;Acc:28254]	chr9	139615818	139622636	13	0.005	1	1	1.185E-05	3.141E-04	PB-GT
MAK16	MAK16 homolog (S. cerevisiae) [Source:HGNC Symbol;Acc:13703]	chr8	33342268	33358778	123	0.042	2	1	1.208E-05	3.174E-04	PB-GT
MAMSTR	MEF2 activating motif and SAP domain containing transcriptional regulator [Source:HGNC Symbol;Acc:26689]	chr19	49215999	49222978	10	0.005	1	1	1.387E-05	3.578E-04	PB-GT
ACPT	acid phosphatase, testicular [Source:HGNC Symbol;Acc:14376]	chr19	51293672	51298481	10	0.005	1	1	1.387E-05	3.578E-04	PB-GT
ARAF	v-raf murine sarcoma 3611 viral oncogene homolog [Source:HGNC Symbol;Acc:646]	chrX	47420516	47431307	32	0.005	1	1	1.416E-05	3.620E-04	PB-GT
C12orf61	chromosome 12 open reading frame 61 [Source:HGNC Symbol;Acc:26364]	chr12	62995531	62997214	14	0.005	1	1	1.431E-05	3.627E-04	PB-GT
MRPL32	mitochondrial ribosomal protein L32 [Source:HGNC Symbol;Acc:14035]	chr7	42971799	42988557	121	0.045	2	1	1.462E-05	3.671E-04	PB-GT
SLC37A4	solute carrier family 37 (glucose-6-phosphate transporter), member 4 [Source:HGNC Symbol;Acc:4061]	chr11	118894824	118901616	16	0.006	1	1	1.536E-05	3.757E-04	PB-GT
RPUSD2	RNA pseudouridylate synthase domain containing 2 [Source:HGNC Symbol;Acc:24180]	chr15	40861499	40866659	16	0.006	1	1	1.542E-05	3.757E-04	PB-GT
VAMP2	vesicle-associated membrane protein 2 (synaptobrevin 2) [Source:HGNC Symbol;Acc:12643]	chr17	8062467	8066864	11	0.006	1	1	1.547E-05	3.757E-04	PB-GT
MAT2B	methionine adenosyltransferase II, beta [Source:HGNC Symbol;Acc:6905]	chr5	162930120	162946342	170	0.046	2	1	1.549E-05	3.757E-04	PB-GT
GPX3	glutathione peroxidase 3 (plasma) [Source:HGNC Symbol;Acc:4555]	chr5	150400124	150408554	21	0.006	1	1	1.595E-05	3.834E-04	PB-GT
PRMT6	protein arginine methyltransferase 6 [Source:HGNC Symbol;Acc:18241]	chr1	107599267	107601916	16	0.006	1	1	1.607E-05	3.834E-04	PB-GT
DBI	diazepam binding inhibitor (GABA receptor modulator, acyl-CoA binding protein) [Source:HGNC Symbol;Acc:2690]	chr2	120124497	120130126	20	0.006	1	1	1.629E-05	3.855E-04	PB-GT
MYL6B	myosin, light chain 6B, alkali, smooth muscle and non-muscle [Source:HGNC Symbol;Acc:29823]	chr12	56546040	56551771	15	0.006	1	1	1.643E-05	3.855E-04	PB-GT
C8orf44	chromosome 8 open reading frame 44 [Source:HGNC Symbol;Acc:25646]	chr8	67579850	67597799	137	0.047	2	2	1.664E-05	3.873E-04	PB-GT
ZFX-AS1	ZFX antisense RNA 1 [Source:HGNC Symbol;Acc:40617]	chrX	24164342	24167771	35	0.006	1	1	1.694E-05	3.911E-04	PB-GT
LURAP1L	leucine rich adaptor protein 1-like [Source:HGNC Symbol;Acc:31452]	chr9	12775020	12822130	391	0.147	3	1	1.715E-05	3.926E-04	PB-GT
SC5D	sterol-C5-desaturase [Source:HGNC Symbol;Acc:10547]	chr11	121163162	121179403	137	0.048	2	1	1.728E-05	3.926E-04	PB-GT
SSR3	signal sequence receptor, gamma (translocon-associated protein gamma) [Source:HGNC Symbol;Acc:11325]	chr3	156257929	156272973	156	0.048	2	1	1.777E-05	4.006E-04	PB-GT
PNPLA8	patatin-like phospholipase domain containing 8 [Source:HGNC Symbol;Acc:28900]	chr7	108110866	108210110	831	0.309	4	1	1.805E-05	4.037E-04	PB-GT
SEL1L	sel-1 suppressor of lin-12-like (C. elegans) [Source:HGNC Symbol;Acc:10717]	chr14	81937893	82000205	550	0.149	3	2	1.831E-05	4.064E-04	PB-GT
PIM1	pim-1 oncogene [Source:HGNC Symbol;Acc:8986]	chr6	37137979	37143202	18	0.006	1	1	1.904E-05	4.192E-04	PB-GT
THAP5	THAP domain containing 5 [Source:HGNC Symbol;Acc:23188]	chr7	108194987	108210194	133	0.049	2	1	1.935E-05	4.229E-04	PB-GT
PLOD3	procollagen-lysine, 2-oxoglutarate 5-dioxygenase 3 [Source:HGNC Symbol;Acc:9083]	chr7	100849258	100861701	17	0.006	1	1	1.984E-05	4.304E-04	PB-GT
RPL39P5	ribosomal protein L39 pseudogene 5 [Source:HGNC Symbol;Acc:26015]	chr3	134070694	134075403	21	0.006	1	1	2.078E-05	4.439E-04	PB-GT
COX15	cytochrome c oxidase assembly homolog 15 (yeast) [Source:HGNC Symbol;Acc:2263]	chr10	101471601	101491857	142	0.051	2	1	2.088E-05	4.439E-04	PB-GT
PSMC6	proteasome (prosome, macropain) 26S subunit, ATPase, 6 [Source:HGNC Symbol;Acc:9553]	chr14	53173890	53195305	187	0.051	2	2	2.093E-05	4.439E-04	PB-GT
TPP1	tripeptidyl peptidase I [Source:HGNC Symbol;Acc:2073]	chr11	6634000	6640692	19	0.007	1	1	2.164E-05	4.515E-04	PB-GT
IFITM2	interferon induced transmembrane protein 2 [Source:HGNC Symbol;Acc:5413]	chr11	307631	315272	19	0.007	1	1	2.164E-05	4.515E-04	PB-GT
FAM206A	family with sequence similarity 206, member A [Source:HGNC Symbol;Acc:1364]	chr9	111696461	111713024	137	0.051	2	2	2.176E-05	4.515E-04	PB-GT

table S2. Genes with significant transposon integrations in RKO *BRAF*$^{wt/-/-}$ cells after selection in low glucose medium

Gene ID	Description	Chrom	Start	End	# TTAA sites	Expected	Observed	Libraries	*P*	FDR	Transposon
RPL7A	ribosomal protein L7a [Source:HGNC Symbol;Acc:10364]	chr9	136215069	136218281	18	0.007	1	1	2.269E-05	4.673E-04	PB-GT
RBM5	RNA binding motif protein 5 [Source:HGNC Symbol;Acc:9902]	chr3	50126341	50156454	171	0.053	2	1	2.332E-05	4.760E-04	PB-GT
ANKZF1	ankyrin repeat and zinc finger domain containing 1 [Source:HGNC Symbol;Acc:25527]	chr2	220094479	220101391	24	0.007	1	1	2.344E-05	4.760E-04	PB-GT
CDX2	caudal type homeobox 2 [Source:HGNC Symbol;Acc:1806]	chr13	28536274	28545276	29	0.007	1	1	2.467E-05	4.974E-04	PB-GT
FAM86KP	family with sequence similarity 86, member K, pseudogene [Source:HGNC Symbol;Acc:44098]	chr4	9155022	9167177	25	0.007	1	1	2.493E-05	4.976E-04	PB-GT
RPL19	ribosomal protein L19 [Source:HGNC Symbol;Acc:10312]	chr17	37356536	37360980	14	0.007	1	1	2.503E-05	4.976E-04	PB-GT
PTRHD1	peptidyl-tRNA hydrolase domain containing 1 [Source:HGNC Symbol;Acc:33782]	chr2	25012855	25016251	25	0.007	1	1	2.543E-05	5.022E-04	PB-GT
AP1S2	adaptor-related protein complex 1, sigma 2 subunit [Source:HGNC Symbol;Acc:560]	chrX	15843929	15873054	328	0.055	2	1	2.611E-05	5.120E-04	PB-GT
NUDT13	nudix (nucleoside diphosphate linked moiety X)-type motif 13 [Source:HGNC Symbol;Acc:18827]	chr10	74870217	74891586	154	0.055	2	2	2.655E-05	5.172E-04	PB-GT
COX6C	cytochrome c oxidase subunit VIc [Source:HGNC Symbol;Acc:2285]	chr8	100885428	100906290	161	0.055	2	1	2.684E-05	5.192E-04	PB-GT
GATA6	GATA binding protein 6 [Source:HGNC Symbol;Acc:4174]	chr18	19749404	19782491	231	0.055	2	1	2.708E-05	5.203E-04	PB-GT
PSMA3	proteasome (prosome, macropain) subunit, alpha type, 3 [Source:HGNC Symbol;Acc:9532]	chr14	58711549	58738730	206	0.056	2	2	2.787E-05	5.320E-04	PB-GT
RCCD1	RCC1 domain containing 1 [Source:HGNC Symbol;Acc:30457]	chr15	91498100	91506349	22	0.008	1	1	2.912E-05	5.521E-04	PB-GT
LAMTOR4	late endosomal/lysosomal adaptor, MAPK and MTOR activator 4 [Source:HGNC Symbol;Acc:33772]	chr7	99746530	99753567	21	0.008	1	1	3.025E-05	5.568E-04	PB-GT
NSUN5	NOP2/Sun domain family, member 5 [Source:HGNC Symbol;Acc:16385]	chr7	72716514	72722864	21	0.008	1	1	3.025E-05	5.568E-04	PB-GT
GIMAP6	GTPase, IMAP family member 6 [Source:HGNC Symbol;Acc:21918]	chr7	150322463	150329473	21	0.008	1	1	3.025E-05	5.568E-04	PB-GT
MIIP	migration and invasion inhibitory protein [Source:HGNC Symbol;Acc:25715]	chr1	12079523	12092102	22	0.008	1	1	3.034E-05	5.568E-04	PB-GT
TCHH	trichohyalin [Source:HGNC Symbol;Acc:11791]	chr1	152078793	152086556	22	0.008	1	1	3.034E-05	5.568E-04	PB-GT
ZNF576	zinc finger protein 576 [Source:HGNC Symbol;Acc:28357]	chr19	44100544	44105309	15	0.008	1	1	3.116E-05	5.621E-04	PB-GT
BBC3	BCL2 binding component 3 [Source:HGNC Symbol;Acc:17868]	chr19	47724081	47736023	15	0.008	1	1	3.116E-05	5.621E-04	PB-GT
SCLY	selenocysteine lyase [Source:HGNC Symbol;Acc:18161]	chr2	238969530	239008054	203	0.058	2	2	3.122E-05	5.621E-04	PB-GT
HPX	hemopexin [Source:HGNC Symbol;Acc:5171]	chr11	6452279	6463847	23	0.008	1	1	3.168E-05	5.633E-04	PB-GT
ACP2	acid phosphatase 2, lysosomal [Source:HGNC Symbol;Acc:123]	chr11	47260853	47270457	23	0.008	1	1	3.168E-05	5.633E-04	PB-GT
BIRC6-AS1	BIRC6 antisense RNA 1 [Source:HGNC Symbol;Acc:40641]	chr2	32602699	32604667	28	0.008	1	1	3.189E-05	5.635E-04	PB-GT
KRR1	KRR1, small subunit (SSU) processome component, homolog (yeast) [Source:HGNC Symbol;Acc:5176]	chr12	75890684	75905416	154	0.059	2	1	3.269E-05	5.741E-04	PB-GT
KDELC2	KDEL (Lys-Asp-Glu-Leu) containing 2 [Source:HGNC Symbol;Acc:28496]	chr11	108342832	108369159	172	0.060	2	2	3.389E-05	5.915E-04	PB-GT
PEX2	peroxisomal biogenesis factor 2 [Source:HGNC Symbol;Acc:9717]	chr8	77892494	77913280	175	0.060	2	2	3.434E-05	5.948E-04	PB-GT
COA4	cytochrome c oxidase assembly factor 4 homolog (S. cerevisiae) [Source:HGNC Symbol;Acc:24604]	chr11	73583712	73588033	24	0.008	1	1	3.449E-05	5.948E-04	PB-GT
ZNF225	zinc finger protein 225 [Source:HGNC Symbol;Acc:13018]	chr19	44616334	44637027	115	0.061	2	1	3.559E-05	6.101E-04	PB-GT
C7orf25	chromosome 7 open reading frame 25 [Source:HGNC Symbol;Acc:21703]	chr7	42948325	42951904	23	0.009	1	1	3.627E-05	6.180E-04	PB-GT
RRP1	ribosomal RNA processing 1 homolog (S. cerevisiae) [Source:HGNC Symbol;Acc:18785]	chr21	45209394	45225174	30	0.009	1	1	3.663E-05	6.206E-04	PB-GT
CCDC86	coiled-coil domain containing 86 [Source:HGNC Symbol;Acc:28359]	chr11	60609544	60618554	25	0.009	1	1	3.741E-05	6.300E-04	PB-GT
HOXB7	homeobox B7 [Source:HGNC Symbol;Acc:5118]	chr17	46684594	46724385	123	0.062	2	2	3.847E-05	6.441E-04	PB-GT
RPL17	ribosomal protein L17 [Source:HGNC Symbol;Acc:10307]	chr18	47014851	47018893	37	0.009	1	1	3.903E-05	6.443E-04	PB-GT
EXOC8	exocyst complex component 8 [Source:HGNC Symbol;Acc:24659]	chr1	231468480	231473598	25	0.009	1	1	3.915E-05	6.443E-04	PB-GT
RAD23B	RAD23 homolog B (S. cerevisiae) [Source:HGNC Symbol;Acc:9813]	chr9	110045418	110094475	484	0.182	3	2	3.916E-05	6.443E-04	PB-GT
YARS	tyrosyl-tRNA synthetase [Source:HGNC Symbol;Acc:12840]	chr1	33240840	33283754	177	0.063	2	2	3.944E-05	6.451E-04	PB-GT
CIRBP	cold inducible RNA binding protein [Source:HGNC Symbol;Acc:1982]	chr19	1259384	1274879	17	0.009	1	1	3.999E-05	6.451E-04	PB-GT
ZNF865	zinc finger protein 865 [Source:HGNC Symbol;Acc:38705]	chr19	56116771	56128635	17	0.009	1	1	3.999E-05	6.451E-04	PB-GT
DR1	down-regulator of transcription 1, TBP-binding (negative cofactor 2) [Source:HGNC Symbol;Acc:3017]	chr1	93811445	93828149	178	0.063	2	2	4.011E-05	6.451E-04	PB-GT
TRAPPC4	trafficking protein particle complex 4 [Source:HGNC Symbol;Acc:19943]	chr11	118889142	118896164	26	0.009	1	1	4.045E-05	6.470E-04	PB-GT
LINC00572	long intergenic non-protein coding RNA 572 [Source:HGNC Symbol;Acc:43722]	chr13	30492784	30500788	38	0.009	1	1	4.230E-05	6.728E-04	PB-GT
RGS20	regulator of G-protein signaling 20 [Source:HGNC Symbol;Acc:14600]	chr8	54764368	54871863	542	0.186	3	2	4.272E-05	6.757E-04	PB-GT
CDC42EP5	CDC42 effector protein (Rho GTPase binding) 5 [Source:HGNC Symbol;Acc:17408]	chr19	54976210	54984411	18	0.009	1	1	4.482E-05	7.029E-04	PB-GT
NUDT5	nudix (nucleoside diphosphate linked moiety X)-type motif 5 [Source:HGNC Symbol;Acc:8052]	chr10	12207324	12238143	184	0.066	2	2	4.493E-05	7.029E-04	PB-GT
LINC00184	long intergenic non-protein coding RNA 184 [Source:HGNC Symbol;Acc:37192]	chr1	234765057	234770526	27	0.010	1	1	4.564E-05	7.036E-04	PB-GT
TMCO3	transmembrane and coiled-coil domains 3 [Source:HGNC Symbol;Acc:20329]	chr13	114145310	114204542	272	0.066	2	1	4.568E-05	7.036E-04	PB-GT
FAM86B1	family with sequence similarity 86, member B1 [Source:HGNC Symbol;Acc:28268]	chr8	12039605	12051642	28	0.010	1	1	4.572E-05	7.036E-04	PB-GT
HIST1H3D	histone cluster 1, H3d [Source:HGNC Symbol;Acc:4767]	chr6	26197068	26199521	28	0.010	1	1	4.596E-05	7.036E-04	PB-GT
ILK	integrin-linked kinase [Source:HGNC Symbol;Acc:6040]	chr11	6624961	6632102	28	0.010	1	1	4.690E-05	7.091E-04	PB-GT
EIF1AD	eukaryotic translation initiation factor 1A domain containing [Source:HGNC Symbol;Acc:28147]	chr11	65764016	65769647	28	0.010	1	1	4.690E-05	7.091E-04	PB-GT
PDCD6IP	programmed cell death 6 interacting protein [Source:HGNC Symbol;Acc:8766]	chr3	33839844	33911194	619	0.190	3	1	4.706E-05	7.091E-04	PB-GT
SERTAD1	SERTA domain containing 1 [Source:HGNC Symbol;Acc:17932]	chr19	40927499	40931932	19	0.010	1	1	4.992E-05	7.461E-04	PB-GT
MAPKAPK2	mitogen-activated protein kinase-activated protein kinase 2 [Source:HGNC Symbol;Acc:6887]	chr1	206858289	206907628	192	0.068	2	2	5.015E-05	7.461E-04	PB-GT
THYN1	thymocyte nuclear protein 1 [Source:HGNC Symbol;Acc:29560]	chr11	134118173	134123264	29	0.010	1	1	5.029E-05	7.461E-04	PB-GT
EIF4A1	eukaryotic translation initiation factor 4A1 [Source:HGNC Symbol;Acc:3282]	chr17	7476024	7482323	20	0.010	1	1	5.097E-05	7.522E-04	PB-GT
ANXA4	annexin A4 [Source:HGNC Symbol;Acc:542]	chr2	69947923	70053596	682	0.195	3	2	5.160E-05	7.576E-04	PB-GT
HES2	hairy and enhancer of split 2 (Drosophila) [Source:HGNC Symbol;Acc:16005]	chr1	6472478	6484730	29	0.010	1	1	5.263E-05	7.615E-04	PB-GT
PINK1-AS	PINK1 antisense RNA [Source:HGNC Symbol;Acc:38872]	chr1	20969150	20978686	29	0.010	1	1	5.263E-05	7.615E-04	PB-GT
NOTCH2	notch 2 [Source:HGNC Symbol;Acc:7882]	chr1	120454176	120612240	1091	0.387	4	2	5.266E-05	7.615E-04	PB-GT
GLO1	glyoxalase I [Source:HGNC Symbol;Acc:4323]	chr6	38643701	38670917	204	0.070	2	2	5.442E-05	7.824E-04	PB-GT
DDT	D-dopachrome tautomerase [Source:HGNC Symbol;Acc:2732]	chr22	24313554	24322660	21	0.010	1	1	5.465E-05	7.824E-04	PB-GT
RPL7	ribosomal protein L7 [Source:HGNC Symbol;Acc:10363]	chr8	74202506	74208024	31	0.011	1	1	5.600E-05	7.941E-04	PB-GT
LINC00240	long intergenic non-protein coding RNA 240 [Source:HGNC Symbol;Acc:18772]	chr6	26988232	26991703	31	0.011	1	1	5.630E-05	7.941E-04	PB-GT
GPR61	G protein-coupled receptor 61 [Source:HGNC Symbol;Acc:13300]	chr1	110082494	110091028	30	0.011	1	1	5.631E-05	7.941E-04	PB-GT
TMA16	translation machinery associated 16 homolog (S. cerevisiae) [Source:HGNC Symbol;Acc:25638]	chr4	164415594	164441691	252	0.071	2	1	5.739E-05	8.054E-04	PB-GT
RBM28	RNA binding motif protein 28 [Source:HGNC Symbol;Acc:21863]	chr7	127950437	127983962	195	0.072	2	1	5.994E-05	8.342E-04	PB-GT
L1CAM	L1 cell adhesion molecule [Source:HGNC Symbol;Acc:6470]	chrX	153126969	153174677	66	0.011	1	1	6.002E-05	8.342E-04	PB-GT
GNB2L1	guanine nucleotide binding protein (G protein), beta polypeptide 2-like 1 [Source:HGNC Symbol;Acc:4399]	chr5	180663909	180675096	41	0.011	1	1	6.060E-05	8.381E-04	PB-GT
SLC44A4	solute carrier family 44, member 4 [Source:HGNC Symbol;Acc:13941]	chr6	31830969	31846823	33	0.011	1	1	6.376E-05	8.730E-04	PB-GT
FLAD1	flavin adenine dinucleotide synthetase 1 [Source:HGNC Symbol;Acc:24671]	chr1	154955814	154965587	32	0.011	1	1	6.403E-05	8.730E-04	PB-GT
TPRG1L	tumor protein p63 regulated 1-like [Source:HGNC Symbol;Acc:27007]	chr1	3541566	3546691	32	0.011	1	1	6.403E-05	8.730E-04	PB-GT
STX1B	syntaxin 1B [Source:HGNC Symbol;Acc:18539]	chr16	31000577	31021949	34	0.011	1	1	6.437E-05	8.734E-04	PB-GT
SPRY1	sprouty homolog 1, antagonist of FGF signaling (Drosophila) [Source:HGNC Symbol;Acc:11269]	chr4	124317950	124324910	41	0.012	1	1	6.686E-05	8.987E-04	PB-GT
C19orf54	chromosome 19 open reading frame 54 [Source:HGNC Symbol;Acc:24758]	chr19	41246761	41257458	22	0.012	1	1	6.686E-05	8.987E-04	PB-GT
CHRAC1	chromatin accessibility complex 1 [Source:HGNC Symbol;Acc:13544]	chr8	141521397	141527236	34	0.012	1	1	6.732E-05	9.006E-04	PB-GT
C4orf46	chromosome 4 open reading frame 46 [Source:HGNC Symbol;Acc:27320]	chr4	159587831	159593407	42	0.012	1	1	7.014E-05	9.341E-04	PB-GT
GRHPR	glyoxylate reductase/hydroxypyruvate reductase [Source:HGNC Symbol;Acc:4570]	chr9	37422663	37436987	32	0.012	1	1	7.146E-05	9.453E-04	PB-GT
VPS36	vacuolar protein sorting 36 homolog (S. cerevisiae) [Source:HGNC Symbol;Acc:20312]	chr13	52986737	53024763	317	0.077	2	1	7.173E-05	9.453E-04	PB-GT
TXNIP	thioredoxin interacting protein [Source:HGNC Symbol;Acc:16952]	chr1	145438469	145442635	34	0.012	1	1	7.226E-05	9.453E-04	PB-GT
MSRB3	methionine sulfoxide reductase B3 [Source:HGNC Symbol;Acc:27375]	chr12	65672423	65882024	1756	0.672	5	2	7.234E-05	9.453E-04	PB-GT
NR2F6	nuclear receptor subfamily 2, group F, member 6 [Source:HGNC Symbol;Acc:7977]	chr19	17342692	17356749	23	0.012	1	1	7.305E-05	9.453E-04	PB-GT
WDR74	WD repeat domain 74 [Source:HGNC Symbol;Acc:25529]	chr11	62599814	62609281	35	0.012	1	1	7.316E-05	9.453E-04	PB-GT
FTSJ3	FtsJ homolog 3 (E. coli) [Source:HGNC Symbol;Acc:17136]	chr17	61896793	61907372	24	0.012	1	1	7.330E-05	9.453E-04	PB-GT
PIP5K1A	phosphatidylinositol-4-phosphate 5-kinase, type I, alpha [Source:HGNC Symbol;Acc:8994]	chr1	151170425	151222012	220	0.078	2	1	7.488E-05	9.583E-04	PB-GT
DCTN1-AS1	DCTN1 antisense RNA 1 [Source:HGNC Symbol;Acc:44151]	chr2	74612613	74621009	43	0.012	1	1	7.499E-05	9.583E-04	PB-GT
CARS2	cysteinyl-tRNA synthetase 2, mitochondrial (putative) [Source:HGNC Symbol;Acc:25695]	chr13	111293759	111365950	323	0.078	2	2	7.580E-05	9.583E-04	PB-GT
HIST1H2BJ	histone cluster 1, H2bj [Source:HGNC Symbol;Acc:4761]	chr6	27094058	27100541	36	0.012	1	1	7.583E-05	9.583E-04	PB-GT
KLHL9	kelch-like family member 9 [Source:HGNC Symbol;Acc:18732]	chr9	21329670	21335379	33	0.012	1	1	7.598E-05	9.583E-04	PB-GT
NR6A1	nuclear receptor subfamily 6, group A, member 1 [Source:HGNC Symbol;Acc:7985]	chr9	127279554	127533589	1814	0.680	5	2	7.720E-05	9.680E-04	PB-GT
BCL2L1	BCL2-like 1 [Source:HGNC Symbol;Acc:992]	chr20	30252255	30311792	218	0.079	2	2	7.743E-05	9.680E-04	PB-GT
NUDT6	nudix (nucleoside diphosphate linked moiety X)-type motif 6 [Source:HGNC Symbol;Acc:8053]	chr4	123813730	123844123	280	0.079	2	2	7.825E-05	9.741E-04	PB-GT
PPAPDC1B	phosphatidic acid phosphatase type 2 domain containing 1B [Source:HGNC Symbol;Acc:25026]	chr8	38120648	38126761	37	0.013	1	1	7.967E-05	9.874E-04	PB-GT
C1orf123	chromosome 1 open reading frame 123 [Source:HGNC Symbol;Acc:26059]	chr1	53679771	53686289	36	0.013	1	1	8.097E-05	9.992E-04	PB-GT
RPL35A	ribosomal protein L35a [Source:HGNC Symbol;Acc:10345]	chr3	197676858	197683481	42	0.013	1	1	8.275E-05	1.012E-03	PB-GT
RPL15	ribosomal protein L15 [Source:HGNC Symbol;Acc:10306]	chr3	23958036	23965183	42	0.013	1	1	8.275E-05	1.012E-03	PB-GT
ZNF283	zinc finger protein 283 [Source:HGNC Symbol;Acc:13077]	chr19	44331444	44353307	154	0.081	2	2	8.416E-05	1.023E-03	PB-GT
GID8	GID complex subunit 8 homolog (S. cerevisiae) [Source:HGNC Symbol;Acc:15857]	chr20	61569471	61576996	36	0.013	1	1	8.434E-05	1.023E-03	PB-GT
EIF1	eukaryotic translation initiation factor 1 [Source:HGNC Symbol;Acc:3249]	chr17	39845137	39848920	26	0.013	1	1	8.597E-05	1.037E-03	PB-GT
NFYB	nuclear transcription factor Y, beta [Source:HGNC Symbol;Acc:7805]	chr12	104510855	104532067	214	0.082	2	1	8.622E-05	1.037E-03	PB-GT
C12orf23	chromosome 12 open reading frame 23 [Source:HGNC Symbol;Acc:28281]	chr12	107349497	107372556	216	0.083	2	2	8.860E-05	1.061E-03	PB-GT
TUT1	terminal uridylyl transferase 1, U6 snRNA-specific [Source:HGNC Symbol;Acc:26184]	chr11	62342517	62359649	39	0.014	1	1	9.075E-05	1.083E-03	PB-GT
ZNF227	zinc finger protein 227 [Source:HGNC Symbol;Acc:13020]	chr19	44711700	44741421	159	0.084	2	1	9.244E-05	1.098E-03	PB-GT
C6orf120	chromosome 6 open reading frame 120 [Source:HGNC Symbol;Acc:21247]	chr6	170102233	170106401	41	0.014	1	1	9.825E-05	1.160E-03	PB-GT
GNPDA2	glucosamine-6-phosphate deaminase 2 [Source:HGNC Symbol;Acc:21526]	chr4	44703885	44728612	303	0.086	2	1	9.868E-05	1.160E-03	PB-GT
DSCR3	Down syndrome critical region gene 3 [Source:HGNC Symbol;Acc:3044]	chr21	38595721	38640262	300	0.086	2	1	9.885E-05	1.160E-03	PB-GT
HIST2H4A	histone cluster 2, H4a [Source:HGNC Symbol;Acc:4794]	chr1	149804221	149811339	40	0.014	1	1	9.987E-05	1.162E-03	PB-GT
HIST2H4B	histone cluster 2, H4b [Source:HGNC Symbol;Acc:29607]	chr1	149825607	149832714	40	0.014	1	1	9.987E-05	1.162E-03	PB-GT
BCL2L2	BCL2-like 2 [Source:HGNC Symbol;Acc:995]	chr14	23767999	23780968	53	0.014	1	1	1.023E-04	1.186E-03	PB-GT
NDUFAF6	NADH dehydrogenase (ubiquinone) complex I, assembly factor 6 [Source:HGNC Symbol;Acc:28625]	chr8	95907995	96128683	1306	0.447	4	2	1.031E-04	1.191E-03	PB-GT
C1orf195	chromosome 1 open reading frame 195 [Source:HGNC Symbol;Acc:32332]	chr1	15490840	15497813	41	0.015	1	1	1.049E-04	1.206E-03	PB-GT
ZNRD1	zinc ribbon domain containing 1 [Source:HGNC Symbol;Acc:13182]	chr6	30026676	30032686	43	0.015	1	1	1.080E-04	1.233E-03	PB-GT
PGLS	6-phosphogluconolactonase [Source:HGNC Symbol;Acc:8903]	chr19	17622438	17632097	28	0.015	1	1	1.081E-04	1.233E-03	PB-GT
PMVK	phosphomevalonate kinase [Source:HGNC Symbol;Acc:9141]	chr1	154897210	154909467	42	0.015	1	1	1.100E-04	1.250E-03	PB-GT
NCBP2	nuclear cap binding protein subunit 2, 20kDa [Source:HGNC Symbol;Acc:7659]	chr3	196662273	196669468	49	0.015	1	1	1.125E-04	1.268E-03	PB-GT
SEMA3F	sema domain, immunoglobulin domain (Ig), short basic domain, secreted, (semaphorin) 3F [Source:HGNC Symbol;Acc:10728]	chr3	50192478	50226508	49	0.015	1	1	1.125E-04	1.268E-03	PB-GT
BYSL	bystin-like [Source:HGNC Symbol;Acc:1157]	chr6	41888926	41900784	44	0.015	1	1	1.131E-04	1.270E-03	PB-GT
MANSC4	MANSC domain containing 4 [Source:HGNC Symbol;Acc:40023]	chr12	27915671	27924209	40	0.015	1	1	1.161E-04	1.298E-03	PB-GT
HIBADH	3-hydroxyisobutyrate dehydrogenase [Source:HGNC Symbol;Acc:4907]	chr7	27565061	27702614	1239	0.460	4	1	1.173E-04	1.305E-03	PB-GT
ZFAND1	zinc finger, AN1-type domain 1 [Source:HGNC Symbol;Acc:25858]	chr8	82613569	82645138	266	0.091	2	1	1.178E-04	1.305E-03	PB-GT
GGCX	gamma-glutamyl carboxylase [Source:HGNC Symbol;Acc:4247]	chr2	85774743	85788670	54	0.015	1	1	1.180E-04	1.305E-03	PB-GT
CDCA5	cell division cycle associated 5 [Source:HGNC Symbol;Acc:14626]	chr11	64833772	64851636	45	0.016	1	1	1.207E-04	1.329E-03	PB-GT
RNF123	ring finger protein 123 [Source:HGNC Symbol;Acc:21148]	chr3	49726932	49758962	51	0.016	1	1	1.218E-04	1.336E-03	PB-GT
EIF4B	eukaryotic translation initiation factor 4B [Source:HGNC Symbol;Acc:3285]	chr12	53399942	53435993	242	0.093	2	1	1.237E-04	1.352E-03	PB-GT
C7orf55	chromosome 7 open reading frame 55 [Source:HGNC Symbol;Acc:26946]	chr7	139024203	139031065	43	0.016	1	1	1.261E-04	1.373E-03	PB-GT
BLVRA	biliverdin reductase A [Source:HGNC Symbol;Acc:1062]	chr7	43798279	43846939	253	0.094	2	1	1.288E-04	1.393E-03	PB-GT
BTN2A3P	butyrophilin, subfamily 2, member A3, pseudogene [Source:HGNC Symbol;Acc:13229]	chr6	26421619	26432611	47	0.016	1	1	1.289E-04	1.393E-03	PB-GT
ATG3	autophagy related 3 [Source:HGNC Symbol;Acc:20962]	chr3	112251356	112280893	306	0.094	2	2	1.296E-04	1.394E-03	PB-GT
RPL38	ribosomal protein L38 [Source:HGNC Symbol;Acc:10349]	chr17	72199721	72206794	32	0.016	1	1	1.300E-04	1.394E-03	PB-GT
MATN4	matrilin 4 [Source:HGNC Symbol;Acc:6910]	chr20	43922085	43937169	45	0.016	1	1	1.315E-04	1.404E-03	PB-GT
NDUFS5	NADH dehydrogenase (ubiquinone) Fe-S protein 5, 15kDa (NADH-coenzyme Q reductase) [Source:HGNC Symbol;Acc:7712]	chr1	39491990	39500308	46	0.016	1	1	1.319E-04	1.404E-03	PB-GT
SON	SON DNA binding protein [Source:HGNC Symbol;Acc:11183]	chr21	34914924	34949812	332	0.095	2	1	1.331E-04	1.408E-03	PB-GT
TMEM39A	transmembrane protein 39A [Source:HGNC Symbol;Acc:25600]	chr3	119148347	119187677	309	0.095	2	2	1.333E-04	1.408E-03	PB-GT
GNG10	guanine nucleotide binding protein (G protein), gamma 10 [Source:HGNC Symbol;Acc:4402]	chr9	114423615	114432526	44	0.017	1	1	1.347E-04	1.413E-03	PB-GT
HSPA5	heat shock 70kDa protein 5 (glucose-regulated protein, 78kDa) [Source:HGNC Symbol;Acc:5238]	chr9	127997132	128003609	44	0.017	1	1	1.347E-04	1.413E-03	PB-GT
SYNJ2BP	synaptojanin 2 binding protein [Source:HGNC Symbol;Acc:18955]	chr14	70838148	70883778	353	0.096	2	1	1.361E-04	1.417E-03	PB-GT
ZNF124	zinc finger protein 124 [Source:HGNC Symbol;Acc:12907]	chr1	247285277	247335318	270	0.096	2	1	1.366E-04	1.417E-03	PB-GT
DNAJC25-GNG1(	DNAJC25-GNG10 readthrough [Source:HGNC Symbol;Acc:37501]	chr9	114393666	114432509	256	0.096	2	1	1.373E-04	1.417E-03	PB-GT

table S2. Genes with significant transposon integrations in RKO *BRAF*$^{wt/-/-}$ cells after selection in low glucose medium

Gene ID	Description	Chrom	Start	End	# TTAA sites	Expected	Observed	Libraries	*P*	FDR	Transposon
DNAJC25	DnaJ (Hsp40) homolog, subfamily C , member 25 [Source:HGNC Symbol;Acc:34187]	chr9	114393632	114432508	256	0.096	2	1	1.373E-04	1.417E-03	PB-GT
LINC00483	long intergenic non-protein coding RNA 483 [Source:HGNC Symbol;Acc:26080]	chr17	48838390	48844918	33	0.017	1	1	1.382E-04	1.417E-03	PB-GT
OSGIN1	oxidative stress induced growth inhibitor 1 [Source:HGNC Symbol;Acc:30093]	chr16	83981887	83999937	50	0.017	1	1	1.387E-04	1.417E-03	PB-GT
DIAPH3-AS2	DIAPH3 antisense RNA 2 [Source:HGNC Symbol;Acc:39916]	chr13	60718832	60727639	69	0.017	1	1	1.388E-04	1.417E-03	PB-GT
KLF6	Kruppel-like factor 6 [Source:HGNC Symbol;Acc:2235]	chr10	3818188	3827473	47	0.017	1	1	1.391E-04	1.417E-03	PB-GT
PEBP1	phosphatidylethanolamine binding protein 1 [Source:HGNC Symbol;Acc:8630]	chr12	118573663	118583389	44	0.017	1	1	1.403E-04	1.424E-03	PB-GT
SLC1A5	solute carrier family 1 (neutral amino acid transporter), member 5 [Source:HGNC Symbol;Acc:10943]	chr19	47278140	47291851	32	0.017	1	1	1.410E-04	1.426E-03	PB-GT
SFR1	SWI5-dependent recombination repair 1 [Source:HGNC Symbol;Acc:29574]	chr10	105881816	105886143	48	0.017	1	1	1.451E-04	1.462E-03	PB-GT
MYO1C	myosin IC [Source:HGNC Symbol;Acc:7597]	chr17	1367392	1396106	34	0.017	1	1	1.466E-04	1.470E-03	PB-GT
SAP18	Sin3A-associated protein, 18kDa [Source:HGNC Symbol;Acc:10530]	chr13	21714653	21723221	71	0.017	1	1	1.469E-04	1.470E-03	PB-GT
URGCP	upregulator of cell proliferation [Source:HGNC Symbol;Acc:30890]	chr7	43915493	43966010	265	0.098	2	2	1.475E-04	1.472E-03	PB-GT
CALM1	calmodulin 1 (phosphorylase kinase, delta) [Source:HGNC Symbol;Acc:1442]	chr14	90862846	90874605	64	0.017	1	1	1.489E-04	1.480E-03	PB-GT
LAMTOR5	late endosomal/lysosomal adaptor, MAPK and MTOR activator 5 [Source:HGNC Symbol;Acc:17955]	chr1	110943871	110950564	49	0.017	1	1	1.495E-04	1.481E-03	PB-GT
EEF2K	eukaryotic elongation factor-2 kinase [Source:HGNC Symbol;Acc:24615]	chr16	22217603	22298554	296	0.099	2	1	1.509E-04	1.481E-03	PB-GT
KIAA1967	KIAA1967 [Source:HGNC Symbol;Acc:23360]	chr8	22462145	22479027	51	0.017	1	1	1.509E-04	1.481E-03	PB-GT
KCTD4	potassium channel tetramerisation domain containing 4 [Source:HGNC Symbol;Acc:23227]	chr13	45766988	45775175	72	0.017	1	1	1.510E-04	1.481E-03	PB-GT
AMOTL2	angiomotin like 2 [Source:HGNC Symbol;Acc:17812]	chr3	134074716	134094321	57	0.018	1	1	1.519E-04	1.485E-03	PB-GT
DNAAF2	dynein, axonemal, assembly factor 2 [Source:HGNC Symbol;Acc:20188]	chr14	50091892	50101948	65	0.018	1	1	1.536E-04	1.495E-03	PB-GT
CALCOCO1	calcium binding and coiled-coil domain 1 [Source:HGNC Symbol;Acc:29306]	chr12	54104903	54121529	47	0.018	1	1	1.600E-04	1.546E-03	PB-GT
NUDT2	nudix (nucleoside diphosphate linked moiety X)-type motif 2 [Source:HGNC Symbol;Acc:8049]	chr9	34329504	34343709	48	0.018	1	1	1.601E-04	1.546E-03	PB-GT
UNC50	unc-50 homolog (C. elegans) [Source:HGNC Symbol;Acc:16046]	chr2	99225042	99234978	63	0.018	1	1	1.604E-04	1.546E-03	PB-GT
RPL37	ribosomal protein L37 [Source:HGNC Symbol;Acc:10347]	chr5	40825364	40835437	67	0.018	1	1	1.611E-04	1.547E-03	PB-GT
BCAR1	breast cancer anti-estrogen resistance 1 [Source:HGNC Symbol;Acc:971]	chr16	75262928	75301951	54	0.018	1	1	1.617E-04	1.548E-03	PB-GT
METTL2B	methyltransferase like 2B [Source:HGNC Symbol;Acc:18272]	chr7	128095894	128146656	274	0.102	2	2	1.627E-04	1.553E-03	PB-GT
SMPD4	sphingomyelin phosphodiesterase 4, neutral membrane (neutral sphingomyelinase-3) [Source:HGNC Symbol;Acc:32949]	chr2	130908981	130940323	64	0.018	1	1	1.655E-04	1.574E-03	PB-GT
HIP1R	huntingtin interacting protein 1 related [Source:HGNC Symbol;Acc:18415]	chr12	123319000	123347507	48	0.018	1	1	1.668E-04	1.575E-03	PB-GT
PSMD7	proteasome (prosome, macropain) 26S subunit, non-ATPase, 7 [Source:HGNC Symbol;Acc:9565]	chr16	74330673	74340186	55	0.018	1	1	1.677E-04	1.575E-03	PB-GT
CYB5B	cytochrome b5 type B (outer mitochondrial membrane) [Source:HGNC Symbol;Acc:24374]	chr16	69458428	69500169	307	0.103	2	2	1.679E-04	1.575E-03	PB-GT
TMEM69	transmembrane protein 69 [Source:HGNC Symbol;Acc:28035]	chr1	46152886	46160115	52	0.018	1	1	1.683E-04	1.575E-03	PB-GT
TSACC	TSSK6 activating co-chaperone [Source:HGNC Symbol;Acc:30636]	chr1	156307105	156316786	52	0.018	1	1	1.683E-04	1.575E-03	PB-GT
DHX16	DEAH (Asp-Glu-Ala-His) box polypeptide 16 [Source:HGNC Symbol;Acc:2739]	chr6	30620896	30640814	54	0.019	1	1	1.699E-04	1.585E-03	PB-GT
FMNL1	formin-like 1 [Source:HGNC Symbol;Acc:1212]	chr17	43298811	43324687	37	0.019	1	1	1.735E-04	1.598E-03	PB-GT
NUTM1	NUT midline carcinoma, family member 1 [Source:HGNC Symbol;Acc:29919]	chr15	34635516	34649938	54	0.019	1	1	1.741E-04	1.598E-03	PB-GT
ARF1	ADP-ribosylation factor 1 [Source:HGNC Symbol;Acc:652]	chr1	228270361	228286912	53	0.019	1	1	1.748E-04	1.598E-03	PB-GT
RABIF	RAB interacting factor [Source:HGNC Symbol;Acc:9797]	chr1	202848085	202858263	53	0.019	1	1	1.748E-04	1.598E-03	PB-GT
SNRPE	small nuclear ribonucleoprotein polypeptide E [Source:HGNC Symbol;Acc:11161]	chr1	203830731	203839678	53	0.019	1	1	1.748E-04	1.598E-03	PB-GT
TRAPPC3	trafficking protein particle complex 3 [Source:HGNC Symbol;Acc:19942]	chr1	36602173	36615098	53	0.019	1	1	1.748E-04	1.598E-03	PB-GT
EIF2AK2	eukaryotic translation initiation factor 2-alpha kinase 2 [Source:HGNC Symbol;Acc:9437]	chr2	37326353	37384208	365	0.104	2	1	1.753E-04	1.598E-03	PB-GT
ZNRF3	zinc and ring finger 3 [Source:HGNC Symbol;Acc:18126]	chr22	29279580	29453475	1007	0.503	4	2	1.771E-04	1.609E-03	PB-GT
ALG5	ALG5, dolichyl-phosphate beta-glucosyltransferase [Source:HGNC Symbol;Acc:20266]	chr13	37523912	37574398	432	0.105	2	2	1.778E-04	1.611E-03	PB-GT
DHODH	dihydroorotate dehydrogenase (quinone) [Source:HGNC Symbol;Acc:2867]	chr16	72042487	72058954	57	0.019	1	1	1.800E-04	1.622E-03	PB-GT
ARPC5L	actin related protein 2/3 complex, subunit 5-like [Source:HGNC Symbol;Acc:23366]	chr9	127624409	127640003	51	0.019	1	1	1.807E-04	1.622E-03	PB-GT
GRPEL2	GrpE-like 2, mitochondrial (E. coli) [Source:HGNC Symbol;Acc:21060]	chr5	148724993	148734146	71	0.019	1	1	1.807E-04	1.622E-03	PB-GT
SDCBP2	syndecan binding protein (syntenin) 2 [Source:HGNC Symbol;Acc:15756]	chr20	1290619	1309883	53	0.019	1	1	1.821E-04	1.626E-03	PB-GT
DDX39B	DEAD (Asp-Glu-Ala-Asp) box polypeptide 39B [Source:HGNC Symbol;Acc:13917]	chr6	31497996	31510225	56	0.019	1	1	1.827E-04	1.626E-03	PB-GT
TXNDC17	thioredoxin domain containing 17 [Source:HGNC Symbol;Acc:28218]	chr17	6544078	6547861	38	0.019	1	1	1.829E-04	1.626E-03	PB-GT
SCAND2P	SCAN domain containing 2 pseudogene [Source:HGNC Symbol;Acc:10567]	chr15	85174682	85185695	56	0.019	1	1	1.872E-04	1.659E-03	PB-GT
DFFA	DNA fragmentation factor, 45kDa, alpha polypeptide [Source:HGNC Symbol;Acc:2772]	chr1	10516579	10532583	55	0.020	1	1	1.881E-04	1.662E-03	PB-GT
EEF1A1	eukaryotic translation elongation factor 1 alpha 1 [Source:HGNC Symbol;Acc:3189]	chr6	74225473	74233520	57	0.020	1	1	1.892E-04	1.667E-03	PB-GT
RPL17-C18orf32	RPL17-C18orf32 readthrough [Source:HGNC Symbol;Acc:44661]	chr18	47008051	47018906	82	0.020	1	1	1.903E-04	1.671E-03	PB-GT
CHAMP1	chromosome alignment maintaining phosphoprotein 1 [Source:HGNC Symbol;Acc:20311]	chr13	115079988	115092796	81	0.020	1	1	1.909E-04	1.671E-03	PB-GT
SRSF1	serine/arginine-rich splicing factor 1 [Source:HGNC Symbol;Acc:10780]	chr17	56080854	56084707	39	0.020	1	1	1.926E-04	1.677E-03	PB-GT
ARL2BP	ADP-ribosylation factor-like 2 binding protein [Source:HGNC Symbol;Acc:17146]	chr16	57279010	57287516	59	0.020	1	1	1.928E-04	1.677E-03	PB-GT
RASAL2	RAS protein activator like 2 [Source:HGNC Symbol;Acc:9874]	chr1	178062864	178448644	3248	1.153	6	2	1.977E-04	1.715E-03	PB-GT
SRRM2	serine/arginine repetitive matrix 2 [Source:HGNC Symbol;Acc:16639]	chr16	2802330	2822539	60	0.020	1	1	1.993E-04	1.724E-03	PB-GT
PRDX1	peroxiredoxin 1 [Source:HGNC Symbol;Acc:9352]	chr1	45976708	45988719	57	0.020	1	1	2.020E-04	1.735E-03	PB-GT
PINK1	PTEN induced putative kinase 1 [Source:HGNC Symbol;Acc:14581]	chr1	20959948	20978004	57	0.020	1	1	2.020E-04	1.735E-03	PB-GT
BCORP1	BCL6 corepressor pseudogene 1 [Source:HGNC Symbol;Acc:23953]	chrY	21617317	21665039	301	0.020	1	1	2.024E-04	1.735E-03	PB-GT
KIAA1430	KIAA1430 [Source:HGNC Symbol;Acc:29276]	chr4	186080819	186130658	390	0.110	2	1	2.066E-04	1.766E-03	PB-GT
SCAF11	SR-related CTD-associated factor 11 [Source:HGNC Symbol;Acc:10784]	chr12	46312914	46385903	735	0.281	3	1	2.088E-04	1.768E-03	PB-GT
GSK3A	glycogen synthase kinase 3 alpha [Source:HGNC Symbol;Acc:4616]	chr19	42734338	42746777	39	0.021	1	1	2.089E-04	1.768E-03	PB-GT
HFE	hemochromatosis [Source:HGNC Symbol;Acc:4886]	chr6	26087509	26098571	60	0.021	1	1	2.095E-04	1.768E-03	PB-GT
MED20	mediator complex subunit 20 [Source:HGNC Symbol;Acc:16840]	chr6	41873092	41888877	60	0.021	1	1	2.095E-04	1.768E-03	PB-GT
TNRC18	trinucleotide repeat containing 18 [Source:HGNC Symbol;Acc:11962]	chr7	5346421	5465045	299	0.111	2	2	2.099E-04	1.768E-03	PB-GT
FN3K	fructosamine 3 kinase [Source:HGNC Symbol;Acc:24822]	chr17	80693451	80709073	41	0.021	1	1	2.127E-04	1.786E-03	PB-GT
SELRC1	Sel1 repeat containing 1 [Source:HGNC Symbol;Acc:25716]	chr1	53152508	53164038	59	0.021	1	1	2.163E-04	1.811E-03	PB-GT
SYS1	SYS1 Golgi-localized integral membrane protein homolog (S. cerevisiae) [Source:HGNC Symbol;Acc:16162]	chr20	43990577	44005438	58	0.021	1	1	2.178E-04	1.812E-03	PB-GT
PLCE1-AS1	PLCE1 antisense RNA 1 [Source:HGNC Symbol;Acc:45193]	chr10	96039034	96046828	59	0.021	1	1	2.186E-04	1.812E-03	PB-GT
WDSUB1	WD repeat, sterile alpha motif and U-box domain containing 1 [Source:HGNC Symbol;Acc:26697]	chr2	160092304	160143310	394	0.113	2	2	2.191E-04	1.812E-03	PB-GT
GABARAPL2	GABA(A) receptor-associated protein-like 2 [Source:HGNC Symbol;Acc:13291]	chr16	75600249	75611779	63	0.021	1	1	2.196E-04	1.812E-03	PB-GT
E2F7	E2F transcription factor 7 [Source:HGNC Symbol;Acc:23820]	chr12	77415027	77459360	295	0.113	2	2	2.207E-04	1.812E-03	PB-GT
FIGNL1	fidgetin-like 1 [Source:HGNC Symbol;Acc:13286]	chr7	50511831	50518088	57	0.021	1	1	2.209E-04	1.812E-03	PB-GT
MNX1	motor neuron and pancreas homeobox 1 [Source:HGNC Symbol;Acc:4979]	chr7	156786745	156803345	57	0.021	1	1	2.209E-04	1.812E-03	PB-GT
ZNF217	zinc finger protein 217 [Source:HGNC Symbol;Acc:13009]	chr20	52183604	52226446	313	0.113	2	2	2.234E-04	1.827E-03	PB-GT
TMEM245	transmembrane protein 245 [Source:HGNC Symbol;Acc:1363]	chr9	111777432	111882225	766	0.287	3	2	2.259E-04	1.843E-03	PB-GT
NBR1	neighbor of BRCA1 gene 1 [Source:HGNC Symbol;Acc:6746]	chr17	41322498	41363708	225	0.114	2	1	2.266E-04	1.843E-03	PB-GT
DNAJB12	DnaJ (Hsp40) homolog, subfamily B, member 12 [Source:HGNC Symbol;Acc:14891]	chr10	74092588	74114988	61	0.022	1	1	2.336E-04	1.890E-03	PB-GT
ZNF200	zinc finger protein 200 [Source:HGNC Symbol;Acc:12993]	chr16	3272325	3286221	65	0.022	1	1	2.336E-04	1.890E-03	PB-GT
BMS1P3	BMS1 pseudogene 3 [Source:HGNC Symbol;Acc:23651]	chr10	88752010	88760192	62	0.022	1	1	2.412E-04	1.945E-03	PB-GT
PREP	prolyl endopeptidase [Source:HGNC Symbol;Acc:9358]	chr6	105725440	105850959	853	0.293	3	2	2.432E-04	1.956E-03	PB-GT
NYX	nyctalopin [Source:HGNC Symbol;Acc:8082]	chrX	41306687	41334963	134	0.022	1	1	2.456E-04	1.959E-03	PB-GT
HIST1H2BK	histone cluster 1, H2bk [Source:HGNC Symbol;Acc:13954]	chr6	27106073	27114619	65	0.022	1	1	2.456E-04	1.959E-03	PB-GT
LIG4	ligase IV, DNA, ATP-dependent [Source:HGNC Symbol;Acc:6601]	chr13	108859787	108870716	92	0.022	1	1	2.458E-04	1.959E-03	PB-GT
MST1L	macrophage stimulating 1-like [Source:HGNC Symbol;Acc:7390]	chr1	17081405	17096732	63	0.022	1	1	2.464E-04	1.959E-03	PB-GT
RBM39	RNA binding motif protein 39 [Source:HGNC Symbol;Acc:15923]	chr20	34291531	34330234	326	0.118	2	1	2.515E-04	1.990E-03	PB-GT
RMDN3	regulator of microtubule dynamics 3 [Source:HGNC Symbol;Acc:25550]	chr15	41028082	41048049	65	0.023	1	1	2.517E-04	1.990E-03	PB-GT
HSBP1	heat shock factor binding protein 1 [Source:HGNC Symbol;Acc:5203]	chr16	83841448	83853342	68	0.023	1	1	2.555E-04	2.015E-03	PB-GT
DCTN1	dynactin 1 [Source:HGNC Symbol;Acc:2711]	chr2	74588281	74619214	81	0.023	1	1	2.642E-04	2.072E-03	PB-GT
RAB17	RAB17, member RAS oncogene family [Source:HGNC Symbol;Acc:16523]	chr2	238482965	238510257	81	0.023	1	1	2.642E-04	2.072E-03	PB-GT
AMZ2	archaelysin family metallopeptidase 2 [Source:HGNC Symbol;Acc:28041]	chr17	66243715	66253297	46	0.023	1	1	2.673E-04	2.084E-03	PB-GT
NHLRC3	NHL repeat containing 3 [Source:HGNC Symbol;Acc:33751]	chr13	39612443	39624246	96	0.023	1	1	2.674E-04	2.084E-03	PB-GT
ATP6V1G2-DDX3	ATP6V1G2-DDX39B readthrough (non-protein coding) [Source:HGNC Symbol;Acc:41999]	chr6	31497996	31514385	68	0.023	1	1	2.686E-04	2.084E-03	PB-GT
ATAT1	alpha tubulin acetyltransferase 1 [Source:HGNC Symbol;Acc:21186]	chr6	30594619	30614600	68	0.023	1	1	2.686E-04	2.084E-03	PB-GT
SMARCE1	SWI/SNF related, matrix associated, actin dependent regulator of chromatin, subfamily e, member 1 [Source:HGNC Symbol;Ac	chr17	38781214	38804760	239	0.121	2	1	2.701E-04	2.090E-03	PB-GT
TCN2	transcobalamin II [Source:HGNC Symbol;Acc:11653]	chr22	31002825	31023265	47	0.023	1	1	2.714E-04	2.094E-03	PB-GT
FASTKD5	FAST kinase domains 5 [Source:HGNC Symbol;Acc:25790]	chr20	3127165	3140543	65	0.024	1	1	2.730E-04	2.101E-03	PB-GT
METTL3	methyltransferase like 3 [Source:HGNC Symbol;Acc:17563]	chr14	21966277	21979517	87	0.024	1	1	2.740E-04	2.103E-03	PB-GT
PDCD2	programmed cell death 2 [Source:HGNC Symbol;Acc:8762]	chr6	170884383	170893780	69	0.024	1	1	2.765E-04	2.116E-03	PB-GT
TYK2	tyrosine kinase 2 [Source:HGNC Symbol;Acc:12440]	chr19	10461209	10491352	45	0.024	1	1	2.775E-04	2.116E-03	PB-GT
ATG16L1	autophagy related 16-like 1 (S. cerevisiae) [Source:HGNC Symbol;Acc:21498]	chr2	234118697	234204320	428	0.122	2	2	2.789E-04	2.116E-03	PB-GT
KIAA1257	KIAA1257 [Source:HGNC Symbol;Acc:29231]	chr3	128628709	128721533	398	0.122	2	2	2.791E-04	2.116E-03	PB-GT
EIF1AX	eukaryotic translation initiation factor 1A, X-linked [Source:HGNC Symbol;Acc:3250]	chrX	20142636	20159962	143	0.024	1	1	2.794E-04	2.116E-03	PB-GT
BET3L	BET3 like (S. cerevisiae) [Source:HGNC Symbol;Acc:21090]	chr6	116816152	116866773	357	0.123	2	1	2.804E-04	2.118E-03	PB-GT
PLAC1L	placenta-specific 1-like [Source:HGNC Symbol;Acc:26699]	chr11	59807748	59815517	69	0.024	1	1	2.821E-04	2.118E-03	PB-GT
CCAT1	colon cancer associated transcript 1 (non-protein coding) [Source:HGNC Symbol;Acc:45128]	chr8	128220111	128231333	70	0.024	1	1	2.830E-04	2.118E-03	PB-GT
ETV5	ets variant 5 [Source:HGNC Symbol;Acc:3494]	chr3	185764097	185828107	400	0.123	2	2	2.832E-04	2.118E-03	PB-GT
TKT	transketolase [Source:HGNC Symbol;Acc:11834]	chr3	53258723	53290068	78	0.024	1	1	2.833E-04	2.118E-03	PB-GT
KIFC3	kinesin family member C3 [Source:HGNC Symbol;Acc:6326]	chr16	57792129	57896957	368	0.123	2	2	2.847E-04	2.123E-03	PB-GT
SPTAN1	spectrin, alpha, non-erythrocytic 1 [Source:HGNC Symbol;Acc:11273]	chr9	131314866	131395941	329	0.123	2	1	2.856E-04	2.123E-03	PB-GT
RNPS1	RNA binding protein S1, serine-rich domain [Source:HGNC Symbol;Acc:10080]	chr16	2303117	2318413	72	0.024	1	1	2.862E-04	2.123E-03	PB-GT
USP3	ubiquitin specific peptidase 3 [Source:HGNC Symbol;Acc:12626]	chr15	63796793	63886839	881	0.306	3	2	2.877E-04	2.128E-03	PB-GT
HDGF	hepatoma-derived growth factor [Source:HGNC Symbol;Acc:4856]	chr1	156711899	156736717	69	0.024	1	1	2.951E-04	2.168E-03	PB-GT
HNRNPA1	heterogeneous nuclear ribonucleoprotein A1 [Source:HGNC Symbol;Acc:5031]	chr12	54673977	54680872	64	0.025	1	1	2.953E-04	2.168E-03	PB-GT
FOXM1	forkhead box M1 [Source:HGNC Symbol;Acc:3818]	chr12	2966847	2986206	64	0.025	1	1	2.953E-04	2.168E-03	PB-GT
TM4SF19	transmembrane 4 L six family member 19 [Source:HGNC Symbol;Acc:25167]	chr3	196042953	196065374	80	0.025	1	1	2.979E-04	2.181E-03	PB-GT
TICRR	TOPBP1-interacting checkpoint and replication regulator [Source:HGNC Symbol;Acc:28704]	chr15	90118713	90174287	361	0.126	2	2	3.002E-04	2.193E-03	PB-GT
SF3B2	splicing factor 3b, subunit 2, 145kDa [Source:HGNC Symbol;Acc:10769]	chr11	65818200	65836779	72	0.025	1	1	3.069E-04	2.236E-03	PB-GT
LINC00667	long intergenic non-protein coding RNA 667 [Source:HGNC Symbol;Acc:27906]	chr18	5232875	5246507	105	0.025	1	1	3.110E-04	2.260E-03	PB-GT
STK38	serine/threonine kinase 38 [Source:HGNC Symbol;Acc:17847]	chr6	36461669	36515247	371	0.127	2	1	3.136E-04	2.270E-03	PB-GT
GEMIN8	gem (nuclear organelle) associated protein 8 [Source:HGNC Symbol;Acc:26044]	chrX	14026398	14048012	152	0.025	1	1	3.153E-04	2.270E-03	PB-GT
AMZ2P1	archaelysin family metallopeptidase 2 pseudogene 1 [Source:HGNC Symbol;Acc:26491]	chr17	62962668	62971694	50	0.025	1	1	3.154E-04	2.270E-03	PB-GT
BRIX1	BRX1, biogenesis of ribosomes, homolog (S. cerevisiae) [Source:HGNC Symbol;Acc:24170]	chr5	34915481	34926101	94	0.025	1	1	3.155E-04	2.270E-03	PB-GT
HIAT1	hippocampus abundant transcript 1 [Source:HGNC Symbol;Acc:23363]	chr1	100503653	100548933	361	0.128	2	2	3.187E-04	2.287E-03	PB-GT
RNF7	ring finger protein 7 [Source:HGNC Symbol;Acc:10070]	chr3	141457046	141466402	83	0.026	1	1	3.205E-04	2.288E-03	PB-GT
NADK	NAD kinase [Source:HGNC Symbol;Acc:29831]	chr1	1682671	1711896	72	0.026	1	1	3.211E-04	2.288E-03	PB-GT
C14orf37	chromosome 14 open reading frame 37 [Source:HGNC Symbol;Acc:19846]	chr14	58466453	58764857	2114	0.573	4	2	3.212E-04	2.288E-03	PB-GT
OXSM	3-oxoacyl-ACP synthase, mitochondrial [Source:HGNC Symbol;Acc:26063]	chr3	25824408	25836025	84	0.026	1	1	3.282E-04	2.321E-03	PB-GT
ALAS1	aminolevulinate, delta-, synthase 1 [Source:HGNC Symbol;Acc:396]	chr3	52232102	52248343	84	0.026	1	1	3.282E-04	2.321E-03	PB-GT
SRRM5	serine/arginine repetitive matrix 5 [Source:HGNC Symbol;Acc:37248]	chr19	44100769	44118649	49	0.026	1	1	3.286E-04	2.321E-03	PB-GT
DNAJB4	DnaJ (Hsp40) homolog, subfamily B, member 4 [Source:HGNC Symbol;Acc:14886]	chr1	78444859	78483648	365	0.130	2	1	3.291E-04	2.321E-03	PB-GT
CABP1	calcium binding protein 1 [Source:HGNC Symbol;Acc:1384]	chr12	121078355	121105127	68	0.026	1	1	3.331E-04	2.337E-03	PB-GT
LINC00238	long intergenic non-protein coding RNA 238 [Source:HGNC Symbol;Acc:20100]	chr14	66953072	66965271	96	0.026	1	1	3.331E-04	2.337E-03	PB-GT
MIR31HG	MIR31 host gene (non-protein coding) [Source:HGNC Symbol;Acc:37187]	chr9	21455641	21559668	857	0.321	3	2	3.445E-04	2.411E-03	PB-GT
DNM2	dynamin 2 [Source:HGNC Symbol;Acc:2974]	chr19	10828755	10944164	250	0.132	2	1	3.467E-04	2.420E-03	PB-GT
SLC19A1	solute carrier family 19 (folate transporter), member 1 [Source:HGNC Symbol;Acc:10937]	chr21	46913486	46964325	93	0.027	1	1	3.478E-04	2.420E-03	PB-GT
SLC2A1	solute carrier family 2 (facilitated glucose transporter), member 1 [Source:HGNC Symbol;Acc:11005]	chr1	43391052	43424530	75	0.027	1	1	3.482E-04	2.420E-03	PB-GT
SCML1	sex comb on midleg-like 1 (Drosophila) [Source:HGNC Symbol;Acc:10580]	chrX	17755588	17773105	160	0.027	1	1	3.491E-04	2.420E-03	PB-GT
NDUFA4	NADH dehydrogenase (ubiquinone) 1 alpha subcomplex, 4, 9kDa [Source:HGNC Symbol;Acc:7687]	chr7	10971578	10979883	72	0.027	1	1	3.511E-04	2.428E-03	PB-GT

table S2. Genes with significant transposon integrations in RKO *BRAF*$^{wt/-/-}$ cells after selection in low glucose medium

Gene ID	Description	Chrom	Start	End	# TTAA sites	Expected	Observed	Libraries	*P*	FDR	Transposon
ANKRD46	ankyrin repeat domain 46 [Source:HGNC Symbol;Acc:27229]	chr8	101521980	101572012	388	0.133	2	1	3.544E-04	2.445E-03	PB-GT
FAM174B	family with sequence similarity 174, member B [Source:HGNC Symbol;Acc:34339]	chr15	93160673	93353114	932	0.324	3	2	3.553E-04	2.445E-03	PB-GT
SUGT1P3	suppressor of G2 allele of SKP1 (S. cerevisiae) pseudogene 3 [Source:HGNC Symbol;Acc:20513]	chr13	41482295	41495910	111	0.027	1	1	3.567E-04	2.449E-03	PB-GT
PPP2R4	protein phosphatase 2A activator, regulatory subunit 4 [Source:HGNC Symbol;Acc:9308]	chr9	131873229	131911225	72	0.027	1	1	3.582E-04	2.453E-03	PB-GT
DPF2	D4, zinc and double PHD fingers family 2 [Source:HGNC Symbol;Acc:9964]	chr11	65101225	65120720	78	0.027	1	1	3.597E-04	2.458E-03	PB-GT
MYL12B	myosin, light chain 12B, regulatory [Source:HGNC Symbol;Acc:29827]	chr18	3261907	3278282	114	0.027	1	1	3.660E-04	2.485E-03	PB-GT
TRIP4	thyroid hormone receptor interactor 4 [Source:HGNC Symbol;Acc:12310]	chr15	64679947	64747502	387	0.135	2	1	3.674E-04	2.485E-03	PB-GT
COA1	cytochrome c oxidase assembly factor 1 homolog (S. cerevisiae) [Source:HGNC Symbol;Acc:21868]	chr7	43648055	43769316	881	0.327	3	1	3.679E-04	2.485E-03	PB-GT
PRDM14	PR domain containing 14 [Source:HGNC Symbol;Acc:14001]	chr8	70963886	70983928	80	0.027	1	1	3.688E-04	2.485E-03	PB-GT
TMEM126A	transmembrane protein 126A [Source:HGNC Symbol;Acc:25382]	chr11	85359011	85367591	79	0.027	1	1	3.689E-04	2.485E-03	PB-GT
DHCR7	7-dehydrocholesterol reductase [Source:HGNC Symbol;Acc:2860]	chr11	71139239	71163914	79	0.027	1	1	3.689E-04	2.485E-03	PB-GT
ZEB2	zinc finger E-box binding homeobox 2 [Source:HGNC Symbol;Acc:14881]	chr2	145145568	145282147	1147	0.328	3	2	3.715E-04	2.497E-03	PB-GT
ALG10	ALG10, alpha-1,2-glucosyltransferase [Source:HGNC Symbol;Acc:23162]	chr12	34175216	34182629	72	0.028	1	1	3.730E-04	2.501E-03	PB-GT
MLL3	myeloid/lymphoid or mixed-lineage leukemia 3 [Source:HGNC Symbol;Acc:13726]	chr7	151832010	152133090	2464	0.915	5	2	3.745E-04	2.505E-03	PB-GT
C14orf93	chromosome 14 open reading frame 93 [Source:HGNC Symbol;Acc:20162]	chr14	23456110	23479375	102	0.028	1	1	3.756E-04	2.507E-03	PB-GT
WDR63	WD repeat domain 63 [Source:HGNC Symbol;Acc:30711]	chr1	85464830	85598821	928	0.329	3	2	3.776E-04	2.507E-03	PB-GT
HAUS1	HAUS augmin-like complex, subunit 1 [Source:HGNC Symbol;Acc:25174]	chr18	43684298	43708299	116	0.028	1	1	3.789E-04	2.507E-03	PB-GT
RABEP2	rabaptin, RAB GTPase binding effector protein 2 [Source:HGNC Symbol;Acc:24817]	chr16	28915742	28947847	83	0.028	1	1	3.794E-04	2.507E-03	PB-GT
ZFPM1	zinc finger protein, FOG family member 1 [Source:HGNC Symbol;Acc:19762]	chr16	88519725	88601572	83	0.028	1	1	3.794E-04	2.507E-03	PB-GT
SRSF3	serine/arginine-rich splicing factor 3 [Source:HGNC Symbol;Acc:10785]	chr6	36562145	36571209	81	0.028	1	1	3.800E-04	2.507E-03	PB-GT
OPA1-AS1	OPA1 antisense RNA 1 [Source:HGNC Symbol;Acc:40421]	chr3	193336398	193345126	91	0.028	1	1	3.846E-04	2.531E-03	PB-GT
KIAA1524	KIAA1524 [Source:HGNC Symbol;Acc:29302]	chr3	108268716	108308491	445	0.137	2	2	3.860E-04	2.534E-03	PB-GT
FAM21C	family with sequence similarity 21, member C [Source:HGNC Symbol;Acc:23414]	chr10	46222648	46288409	384	0.137	2	1	3.872E-04	2.537E-03	PB-GT
SMIM21	small integral membrane protein 21 [Source:HGNC Symbol;Acc:27598]	chr18	73121431	73139658	119	0.029	1	1	3.985E-04	2.601E-03	PB-GT
OSTCP1	oligosaccharyltransferase complex subunit pseudogene 1 [Source:HGNC Symbol;Acc:30530]	chr6	159262149	159278664	83	0.029	1	1	3.988E-04	2.601E-03	PB-GT
PFKFB4	6-phosphofructo-2-kinase/fructose-2,6-biphosphatase 4 [Source:HGNC Symbol;Acc:8875]	chr3	48555117	48599448	93	0.029	1	1	4.015E-04	2.610E-03	PB-GT
CD99P1	CD99 molecule pseudogene 1 [Source:HGNC Symbol;Acc:7083]	chrX	2527389	2575270	172	0.029	1	1	4.029E-04	2.610E-03	PB-GT
PRDX4	peroxiredoxin 4 [Source:HGNC Symbol;Acc:17169]	chrX	23682379	23704516	172	0.029	1	1	4.029E-04	2.610E-03	PB-GT
HSPA9	heat shock 70kDa protein 9 (mortalin) [Source:HGNC Symbol;Acc:5244]	chr5	137890571	137911133	107	0.029	1	1	4.079E-04	2.636E-03	PB-GT
MLLT6	myeloid/lymphoid or mixed-lineage leukemia (trithorax homolog, Drosophila); translocated to, 6 [Source:HGNC Symbol;Acc:713	chr17	36861795	36886056	57	0.029	1	1	4.089E-04	2.637E-03	PB-GT
BRWD1	bromodomain and WD repeat domain containing 1 [Source:HGNC Symbol;Acc:12760]	chr21	40556102	40693485	1180	0.338	3	2	4.139E-04	2.656E-03	PB-GT
CNOT6	CCR4-NOT transcription complex, subunit 6 [Source:HGNC Symbol;Acc:14099]	chr5	179921412	180005405	521	0.140	2	1	4.154E-04	2.656E-03	PB-GT
KLHL21	kelch-like family member 21 [Source:HGNC Symbol;Acc:29041]	chr1	6650784	6674667	82	0.029	1	1	4.155E-04	2.656E-03	PB-GT
PPP1R15B	protein phosphatase 1, regulatory subunit 15B [Source:HGNC Symbol;Acc:14951]	chr1	204372515	204380919	82	0.029	1	1	4.155E-04	2.656E-03	PB-GT
TCEANC	transcription elongation factor A (SII) N-terminal and central domain containing [Source:HGNC Symbol;Acc:28277]	chrX	13671225	13700083	175	0.029	1	1	4.169E-04	2.658E-03	PB-GT
INSIG1	insulin induced gene 1 [Source:HGNC Symbol;Acc:6083]	chr7	155089486	155101945	79	0.029	1	1	4.220E-04	2.679E-03	PB-GT
LATS1	large tumor suppressor kinase 1 [Source:HGNC Symbol;Acc:6514]	chr6	149979289	150039392	411	0.141	2	1	4.220E-04	2.679E-03	PB-GT
H3F3B	H3 histone, family 3B (H3.3B) [Source:HGNC Symbol;Acc:4765]	chr17	73772515	73781974	58	0.029	1	1	4.232E-04	2.681E-03	PB-GT
SLC17A5	solute carrier family 17 (anion/sugar transporter), member 5 [Source:HGNC Symbol;Acc:10933]	chr6	74303102	74363878	412	0.142	2	2	4.250E-04	2.682E-03	PB-GT
HJURP	Holliday junction recognition protein [Source:HGNC Symbol;Acc:25444]	chr2	234742062	234763212	103	0.029	1	1	4.254E-04	2.682E-03	PB-GT
SF1	splicing factor 1 [Source:HGNC Symbol;Acc:12950]	chr11	64532078	64546258	85	0.029	1	1	4.265E-04	2.684E-03	PB-GT
SERF2	small EDRK-rich factor 2 [Source:HGNC Symbol;Acc:10757]	chr15	44069285	44094787	85	0.030	1	1	4.284E-04	2.690E-03	PB-GT
FAM115A	family with sequence similarity 115, member A [Source:HGNC Symbol;Acc:22201]	chr7	143548468	143599291	383	0.142	2	1	4.311E-04	2.701E-03	PB-GT
TET2	tet methylcytosine dioxygenase 2 [Source:HGNC Symbol;Acc:25941]	chr4	106067032	106200973	1207	0.342	3	2	4.330E-04	2.707E-03	PB-GT
ZNF143	zinc finger protein 143 [Source:HGNC Symbol;Acc:12928]	chr11	9481866	9550071	411	0.143	2	1	4.346E-04	2.711E-03	PB-GT
C1RL	complement component 1, r subcomponent-like [Source:HGNC Symbol;Acc:21265]	chr12	7242183	7261869	78	0.030	1	1	4.371E-04	2.717E-03	PB-GT
DEF6	differentially expressed in FDCP 6 homolog (mouse) [Source:HGNC Symbol;Acc:2760]	chr6	35265595	35289548	87	0.030	1	1	4.378E-04	2.717E-03	PB-GT
MFAP1	microfibrillar-associated protein 1 [Source:HGNC Symbol;Acc:7032]	chr15	44096690	44117000	86	0.030	1	1	4.384E-04	2.717E-03	PB-GT
ZNF182	zinc finger protein 182 [Source:HGNC Symbol;Acc:13001]	chrX	47834250	47863377	180	0.030	1	1	4.408E-04	2.724E-03	PB-GT
AGA	aspartylglucosaminidase [Source:HGNC Symbol;Acc:318]	chr4	178351924	178363657	106	0.030	1	1	4.414E-04	2.724E-03	PB-GT
FBXL12	F-box and leucine-rich repeat protein 12 [Source:HGNC Symbol;Acc:13611]	chr19	9920943	9938492	57	0.030	1	1	4.434E-04	2.730E-03	PB-GT
RPL5	ribosomal protein L5 [Source:HGNC Symbol;Acc:10360]	chr1	93297582	93307481	85	0.030	1	1	4.462E-04	2.732E-03	PB-GT
PRPF4B	PRP4 pre-mRNA processing factor 4 homolog B (yeast) [Source:HGNC Symbol;Acc:17346]	chr6	4021501	4065217	419	0.144	2	1	4.462E-04	2.732E-03	PB-GT
TBRG1	transforming growth factor beta regulator 1 [Source:HGNC Symbol;Acc:29551]	chr11	124492732	124505287	87	0.030	1	1	4.466E-04	2.732E-03	PB-GT
CCT5	chaperonin containing TCP1, subunit 5 (epsilon) [Source:HGNC Symbol;Acc:1618]	chr5	10250033	10266524	113	0.030	1	1	4.544E-04	2.774E-03	PB-GT
AP1S3	adaptor-related protein complex 1, sigma 3 subunit [Source:HGNC Symbol;Acc:18971]	chr2	224616403	224702744	507	0.145	2	2	4.558E-04	2.777E-03	PB-GT
MIR3654	microRNA 3654 [Source:HGNC Symbol;Acc:38896]	chr11	62327075	62359003	88	0.031	1	1	4.568E-04	2.777E-03	PB-GT
BRD3	bromodomain containing 3 [Source:HGNC Symbol;Acc:1104]	chr9	136895427	136933657	82	0.031	1	1	4.634E-04	2.800E-03	PB-GT
CIZ1	CDKN1A interacting zinc finger protein 1 [Source:HGNC Symbol;Acc:16744]	chr9	130928343	130966662	82	0.031	1	1	4.634E-04	2.800E-03	PB-GT
UMPS	uridine monophosphate synthetase [Source:HGNC Symbol;Acc:12563]	chr3	124449213	124464040	100	0.031	1	1	4.636E-04	2.800E-03	PB-GT
SLC3A2	solute carrier family 3 (activators of dibasic and neutral amino acid transport), member 2 [Source:HGNC Symbol;Acc:11026]	chr11	62623518	62656352	89	0.031	1	1	4.672E-04	2.810E-03	PB-GT
HSPA13	heat shock protein 70kDa family, member 13 [Source:HGNC Symbol;Acc:11375]	chr21	15743436	15755805	108	0.031	1	1	4.677E-04	2.810E-03	PB-GT
ZFYVE16	zinc finger, FYVE domain containing 16 [Source:HGNC Symbol;Acc:20756]	chr5	79703832	79775169	543	0.146	2	2	4.682E-04	2.810E-03	PB-GT
CD97	CD97 molecule [Source:HGNC Symbol;Acc:1711]	chr19	14491313	14519537	59	0.031	1	1	4.747E-04	2.837E-03	PB-GT
HSD17B14	hydroxysteroid (17-beta) dehydrogenase 14 [Source:HGNC Symbol;Acc:23238]	chr19	49316274	49339935	59	0.031	1	1	4.747E-04	2.837E-03	PB-GT
ABL1	c-abl oncogene 1, non-receptor tyrosine kinase [Source:HGNC Symbol;Acc:76]	chr9	133589333	133763062	937	0.351	3	1	4.807E-04	2.861E-03	PB-GT
TAC3	tachykinin 3 [Source:HGNC Symbol;Acc:11521]	chr12	57403784	57422667	82	0.031	1	1	4.826E-04	2.861E-03	PB-GT
TNS4	tensin 4 [Source:HGNC Symbol;Acc:24352]	chr17	38632080	38657849	62	0.031	1	1	4.830E-04	2.861E-03	PB-GT
SPATA4	spermatogenesis associated 4 [Source:HGNC Symbol;Acc:17333]	chr4	177105789	177116822	111	0.031	1	1	4.836E-04	2.861E-03	PB-GT
FBXO9	F-box protein 9 [Source:HGNC Symbol;Acc:13588]	chr6	52916789	52965671	431	0.148	2	2	4.842E-04	2.861E-03	PB-GT
DLST	dihydrolipoamide S-succinyltransferase (E2 component of 2-oxo-glutarate complex) [Source:HGNC Symbol;Acc:2911]	chr14	75348594	75370448	116	0.031	1	1	4.846E-04	2.861E-03	PB-GT
GATAD1	GATA zinc finger domain containing 1 [Source:HGNC Symbol;Acc:29941]	chr7	92076767	92088150	85	0.032	1	1	4.878E-04	2.872E-03	PB-GT
SMAD7	SMAD family member 7 [Source:HGNC Symbol;Acc:6773]	chr18	46446223	46477081	132	0.032	1	1	4.893E-04	2.872E-03	PB-GT
ZNF770	zinc finger protein 770 [Source:HGNC Symbol;Acc:26061]	chr15	35270542	35280488	91	0.032	1	1	4.903E-04	2.872E-03	PB-GT
TXNRD1	thioredoxin reductase 1 [Source:HGNC Symbol;Acc:12437]	chr12	104609557	104744061	923	0.353	3	2	4.906E-04	2.872E-03	PB-GT
BAG2	BCL2-associated athanogene 2 [Source:HGNC Symbol;Acc:938]	chr6	57037124	57049735	93	0.032	1	1	4.995E-04	2.919E-03	PB-GT
ETS2	v-ets erythroblastosis virus E26 oncogene homolog 2 (avian) [Source:HGNC Symbol;Acc:3489]	chr21	40177231	40196879	112	0.032	1	1	5.026E-04	2.923E-03	PB-GT
GNPDA1	glucosamine-6-phosphate deaminase 1 [Source:HGNC Symbol;Acc:4417]	chr5	141371314	141392606	119	0.032	1	1	5.034E-04	2.923E-03	PB-GT
SLC1A3	solute carrier family 1 (glial high affinity glutamate transporter), member 3 [Source:HGNC Symbol;Acc:10941]	chr5	36606457	36688436	557	0.150	2	2	5.039E-04	2.923E-03	PB-GT
IARS2	isoleucyl-tRNA synthetase 2, mitochondrial [Source:HGNC Symbol;Acc:29685]	chr1	220267444	220321380	423	0.150	2	1	5.044E-04	2.923E-03	PB-GT
MAF	v-maf musculoaponeurotic fibrosarcoma oncogene homolog (avian) [Source:HGNC Symbol;Acc:6776]	chr16	79619740	79634611	96	0.032	1	1	5.061E-04	2.927E-03	PB-GT
GPBP1L1	GC-rich promoter binding protein 1-like 1 [Source:HGNC Symbol;Acc:28843]	chr1	46092976	46153785	424	0.151	2	2	5.079E-04	2.932E-03	PB-GT
PSMA1	proteasome (prosome, macropain) subunit, alpha type, 1 [Source:HGNC Symbol;Acc:9530]	chr11	14515329	14665181	1029	0.357	3	2	5.099E-04	2.937E-03	PB-GT
LUM	lumican [Source:HGNC Symbol;Acc:6724]	chr12	91496406	91505608	85	0.033	1	1	5.182E-04	2.973E-03	PB-GT
TBX3	T-box 3 [Source:HGNC Symbol;Acc:11602]	chr12	115108059	115121969	85	0.033	1	1	5.182E-04	2.973E-03	PB-GT
TRIM3	tripartite motif containing 3 [Source:HGNC Symbol;Acc:10064]	chr11	6469843	6495689	94	0.033	1	1	5.205E-04	2.981E-03	PB-GT
CUTC	cutC copper transporter homolog (E. coli) [Source:HGNC Symbol;Acc:24271]	chr10	101462315	101515891	428	0.153	2	1	5.299E-04	3.018E-03	PB-GT
PIGS	phosphatidylinositol glycan anchor biosynthesis, class S [Source:HGNC Symbol;Acc:14937]	chr17	26880401	26898890	65	0.033	1	1	5.303E-04	3.018E-03	PB-GT
NACA	nascent polypeptide-associated complex alpha subunit [Source:HGNC Symbol;Acc:7629]	chr12	57106212	57125412	86	0.033	1	1	5.303E-04	3.018E-03	PB-GT
DNTTIP2	deoxynucleotidyltransferase, terminal, interacting protein 2 [Source:HGNC Symbol;Acc:24013]	chr1	94333373	94345474	93	0.033	1	1	5.331E-04	3.028E-03	PB-GT
CENPH	centromere protein H [Source:HGNC Symbol;Acc:17268]	chr5	68485375	68506184	123	0.033	1	1	5.374E-04	3.045E-03	PB-GT
SNRNP200	small nuclear ribonucleoprotein 200kDa (U5) [Source:HGNC Symbol;Acc:30859]	chr2	96940074	96971297	116	0.033	1	1	5.382E-04	3.045E-03	PB-GT
OGG1	8-oxoguanine DNA glycosylase [Source:HGNC Symbol;Acc:8125]	chr3	9791628	9829903	108	0.033	1	1	5.398E-04	3.048E-03	PB-GT
TCEB3	transcription elongation factor B (SIII), polypeptide 3 (110kDa, elongin A) [Source:HGNC Symbol;Acc:11620]	chr1	24069645	24088549	94	0.033	1	1	5.445E-04	3.069E-03	PB-GT
OTUD7B	OTU domain containing 7B [Source:HGNC Symbol;Acc:16683]	chr1	149912231	149982625	435	0.154	2	1	5.468E-04	3.076E-03	PB-GT
KCTD13	potassium channel tetramerisation domain containing 13 [Source:HGNC Symbol;Acc:22234]	chr16	29916333	29938356	100	0.033	1	1	5.487E-04	3.080E-03	PB-GT
BZW1	basic leucine zipper and W2 domains 1 [Source:HGNC Symbol;Acc:18380]	chr2	201675317	201688569	118	0.034	1	1	5.567E-04	3.113E-03	PB-GT
ASB1	ankyrin repeat and SOCS box containing 1 [Source:HGNC Symbol;Acc:16011]	chr2	239335383	239360891	118	0.034	1	1	5.567E-04	3.113E-03	PB-GT
IGF1R	insulin-like growth factor 1 receptor [Source:HGNC Symbol;Acc:5465]	chr15	99192200	99507759	1864	0.648	4	2	5.582E-04	3.115E-03	PB-GT
HIF1AN	hypoxia inducible factor 1, alpha subunit inhibitor [Source:HGNC Symbol;Acc:17113]	chr10	102288829	102309763	95	0.034	1	1	5.619E-04	3.128E-03	PB-GT
SIAH1	siah E3 ubiquitin protein ligase 1 [Source:HGNC Symbol;Acc:10857]	chr16	48390275	48482313	466	0.156	2	1	5.642E-04	3.128E-03	PB-GT
SPDYA	speedy homolog A (Xenopus laevis) [Source:HGNC Symbol;Acc:30613]	chr2	29005383	29073477	546	0.156	2	1	5.646E-04	3.128E-03	PB-GT
NABP1	nucleic acid binding protein 1 [Source:HGNC Symbol;Acc:26232]	chr2	192542794	192553251	119	0.034	1	1	5.661E-04	3.128E-03	PB-GT
SLC20A1	solute carrier family 20 (phosphate transporter), member 1 [Source:HGNC Symbol;Acc:10946]	chr2	113403434	113421404	119	0.034	1	1	5.661E-04	3.128E-03	PB-GT
C20orf111	chromosome 20 open reading frame 111 [Source:HGNC Symbol;Acc:16105]	chr20	42825136	42839431	94	0.034	1	1	5.670E-04	3.128E-03	PB-GT
SLC46A3	solute carrier family 46, member 3 [Source:HGNC Symbol;Acc:27501]	chr13	29274201	29293107	141	0.034	1	1	5.727E-04	3.146E-03	PB-GT
TUBA1C	tubulin, alpha 1c [Source:HGNC Symbol;Acc:20768]	chr12	49582519	49667114	410	0.157	2	1	5.733E-04	3.146E-03	PB-GT
PARP10	poly (ADP-ribose) polymerase family, member 10 [Source:HGNC Symbol;Acc:25895]	chr8	145051321	145086940	100	0.034	1	1	5.736E-04	3.146E-03	PB-GT
CA13	carbonic anhydrase XIII [Source:HGNC Symbol;Acc:14914]	chr8	86132816	86196302	459	0.157	2	1	5.763E-04	3.155E-03	PB-GT
WNT7B	wingless-type MMTV integration site family, member 7B [Source:HGNC Symbol;Acc:12787]	chr22	46316242	46373009	69	0.034	1	1	5.807E-04	3.173E-03	PB-GT
MYH9	myosin, heavy chain 9, non-muscle [Source:HGNC Symbol;Acc:7579]	chr22	36677327	36784063	317	0.158	2	2	5.882E-04	3.208E-03	PB-GT
ZDHHC23	zinc finger, DHHC-type containing 23 [Source:HGNC Symbol;Acc:28654]	chr3	113666748	113684248	113	0.035	1	1	5.903E-04	3.213E-03	PB-GT
AKAP8	A kinase (PRKA) anchor protein 8 [Source:HGNC Symbol;Acc:378]	chr19	15464196	15490612	66	0.035	1	1	5.925E-04	3.219E-03	PB-GT
FTX	FTX transcript, XIST regulator (non-protein coding) [Source:HGNC Symbol;Acc:37190]	chrX	73183790	73513409	2235	0.372	3	2	5.959E-04	3.231E-03	PB-GT
COMT	catechol-O-methyltransferase [Source:HGNC Symbol;Acc:2228]	chr22	19929130	19957498	70	0.035	1	1	5.975E-04	3.234E-03	PB-GT
TCP1	t-complex 1 [Source:HGNC Symbol;Acc:11655]	chr6	160199530	160210781	102	0.035	1	1	5.997E-04	3.236E-03	PB-GT
RB1CC1	RB1-inducible coiled-coil 1 [Source:HGNC Symbol;Acc:15574]	chr8	53535016	53658403	1089	0.373	3	1	6.001E-04	3.236E-03	PB-GT
NAP1L1	nucleosome assembly protein 1-like 1 [Source:HGNC Symbol;Acc:7637]	chr12	76438670	76478813	418	0.160	2	1	6.062E-04	3.262E-03	PB-GT
HNRNPA3	heterogeneous nuclear ribonucleoprotein A3 [Source:HGNC Symbol;Acc:24941]	chr2	178077291	178088686	124	0.035	1	1	6.141E-04	3.288E-03	PB-GT
ASB9	ankyrin repeat and SOCS box containing 9 [Source:HGNC Symbol;Acc:17184]	chrX	15253410	15288589	213	0.035	1	1	6.150E-04	3.288E-03	PB-GT
ZGPAT	zinc finger, CCCH-type with G patch domain [Source:HGNC Symbol;Acc:15948]	chr20	62338794	62370456	98	0.036	1	1	6.157E-04	3.288E-03	PB-GT
RNF6	ring finger protein (C3H2C3 type) 6 [Source:HGNC Symbol;Acc:10069]	chr13	26706253	26796791	663	0.161	2	1	6.164E-04	3.288E-03	PB-GT
SYDE2	synapse defective 1, Rho GTPase, homolog 2 (C. elegans) [Source:HGNC Symbol;Acc:25841]	chr1	85622556	85666729	454	0.161	2	2	6.186E-04	3.288E-03	PB-GT
GSG1	germ cell associated 1 [Source:HGNC Symbol;Acc:19716]	chr12	13236494	13256619	93	0.036	1	1	6.190E-04	3.288E-03	PB-GT
C9orf24	chromosome 9 open reading frame 24 [Source:HGNC Symbol;Acc:19919]	chr9	34379017	34397830	95	0.036	1	1	6.200E-04	3.288E-03	PB-GT
FAM178A	family with sequence similarity 178, member A [Source:HGNC Symbol;Acc:17814]	chr10	102672720	102724893	452	0.161	2	1	6.202E-04	3.288E-03	PB-GT
SAMD4A	sterile alpha motif domain containing 4A [Source:HGNC Symbol;Acc:23023]	chr14	55033815	55260033	1389	0.377	3	2	6.220E-04	3.291E-03	PB-GT
SNRPC	small nuclear ribonucleoprotein polypeptide C [Source:HGNC Symbol;Acc:11157]	chr6	34725183	34741571	104	0.036	1	1	6.231E-04	3.291E-03	PB-GT
WDFY1	WD repeat and FYVE domain containing 1 [Source:HGNC Symbol;Acc:20451]	chr2	224720433	224810104	566	0.162	2	1	6.263E-04	3.302E-03	PB-GT
PPM1F	protein phosphatase, Mg2+/Mn2+ dependent, 1F [Source:HGNC Symbol;Acc:19388]	chr22	22273793	22307209	72	0.036	1	1	6.317E-04	3.309E-03	PB-GT
PPP6R2	protein phosphatase 6, regulatory subunit 2 [Source:HGNC Symbol;Acc:19253]	chr22	50781733	50883514	325	0.162	2	1	6.320E-04	3.309E-03	PB-GT
SETD8	SET domain containing (lysine methyltransferase) 8 [Source:HGNC Symbol;Acc:29489]	chr12	123868320	123893905	94	0.036	1	1	6.323E-04	3.309E-03	PB-GT
EMP1	epithelial membrane protein 1 [Source:HGNC Symbol;Acc:3333]	chr12	13349650	13369708	94	0.036	1	1	6.323E-04	3.309E-03	PB-GT
MLKL	mixed lineage kinase domain-like [Source:HGNC Symbol;Acc:26617]	chr16	74705753	74734858	108	0.036	1	1	6.389E-04	3.336E-03	PB-GT
TRERF1	transcriptional regulating factor 1 [Source:HGNC Symbol;Acc:18273]	chr6	42192669	42419789	1105	0.380	3	1	6.396E-04	3.336E-03	PB-GT
ACTR8	ARP8 actin-related protein 8 homolog (yeast) [Source:HGNC Symbol;Acc:14672]	chr3	53901093	53916229	118	0.036	1	1	6.431E-04	3.348E-03	PB-GT
CCT6A	chaperonin containing TCP1, subunit 6A (zeta 1) [Source:HGNC Symbol;Acc:1620]	chr7	56119323	56131682	98	0.036	1	1	6.463E-04	3.358E-03	PB-GT
PNPLA4	patatin-like phospholipase domain containing 4 [Source:HGNC Symbol;Acc:24887]	chrX	7866288	7895780	219	0.036	1	1	6.497E-04	3.370E-03	PB-GT

table S2. Genes with significant transposon integrations in RKO *BRAF*$^{wt/-/-}$ cells after selection in low glucose medium

Gene ID	Description	Chrom	Start	End	# TTAA sites	Expected	Observed	Libraries	P	FDR	Transposon
EXOC5	exocyst complex component 5 [Source:HGNC Symbol;Acc:10696]	chr14	57670518	57735726	605	0.164	2	1	6.513E-04	3.371E-03	PB-GT
RPL22	ribosomal protein L22 [Source:HGNC Symbol;Acc:10315]	chr1	6241329	6269449	103	0.037	1	1	6.524E-04	3.371E-03	PB-GT
ACAT2	acetyl-CoA acetyltransferase 2 [Source:HGNC Symbol;Acc:94]	chr6	160181360	160200144	107	0.037	1	1	6.591E-04	3.400E-03	PB-GT
CORO7	coronin 7 [Source:HGNC Symbol;Acc:26161]	chr16	4404543	4475706	110	0.037	1	1	6.625E-04	3.406E-03	PB-GT
SRSF11	serine/arginine-rich splicing factor 11 [Source:HGNC Symbol;Acc:10782]	chr1	70671365	70718735	465	0.165	2	2	6.627E-04	3.406E-03	PB-GT
GTF3C2	general transcription factor IIIC, polypeptide 2, beta 110kDa [Source:HGNC Symbol;Acc:4665]	chr2	27548716	27579868	129	0.037	1	1	6.639E-04	3.406E-03	PB-GT
NEK2	NIMA-related kinase 2 [Source:HGNC Symbol;Acc:7745]	chr1	211836114	211848960	104	0.037	1	1	6.650E-04	3.406E-03	PB-GT
SS18L1	synovial sarcoma translocation gene on chromosome 18-like 1 [Source:HGNC Symbol;Acc:15592]	chr20	60718822	60757540	102	0.037	1	1	6.664E-04	3.407E-03	PB-GT
CCND3	cyclin D3 [Source:HGNC Symbol;Acc:1585]	chr6	41902671	42018095	484	0.166	2	2	6.765E-04	3.452E-03	PB-GT
MAD1L1	MAD1 mitotic arrest deficient-like 1 (yeast) [Source:HGNC Symbol;Acc:6762]	chr7	1855429	2272878	1039	0.386	3	2	6.795E-04	3.461E-03	PB-GT
TNKS2-AS1	TNKS2 antisense RNA 1 (head to head) [Source:HGNC Symbol;Acc:45173]	chr10	93542596	93558048	105	0.037	1	1	6.848E-04	3.482E-03	PB-GT
VDAC1	voltage-dependent anion channel 1 [Source:HGNC Symbol;Acc:12669]	chr5	133307606	133340824	140	0.038	1	1	6.941E-04	3.523E-03	PB-GT
BMP2K	BMP2 inducible kinase [Source:HGNC Symbol;Acc:18041]	chr4	79697496	79837526	1372	0.388	3	2	6.967E-04	3.531E-03	PB-GT
RAB7A	RAB7A, member RAS oncogene family [Source:HGNC Symbol;Acc:9788]	chr3	128444965	128533639	549	0.169	2	1	7.077E-04	3.580E-03	PB-GT
ZC3H6	zinc finger CCCH-type containing 6 [Source:HGNC Symbol;Acc:24762]	chr2	113033171	113097640	592	0.169	2	1	7.126E-04	3.594E-03	PB-GT
ZNF786	zinc finger protein 786 [Source:HGNC Symbol;Acc:21806]	chr7	148766735	148787874	103	0.038	1	1	7.131E-04	3.594E-03	PB-GT
SLC2A3	solute carrier family 2 (facilitated glucose transporter), member 3 [Source:HGNC Symbol;Acc:11007]	chr12	8071826	8088871	100	0.038	1	1	7.145E-04	3.595E-03	PB-GT
ANKRD30BL	ankyrin repeat domain 30B-like [Source:HGNC Symbol;Acc:35167]	chr2	132905164	133015542	594	0.170	2	1	7.196E-04	3.609E-03	PB-GT
DST	dystonin [Source:HGNC Symbol;Acc:1090]	chr6	56322785	56819426	4181	1.436	6	2	7.213E-04	3.609E-03	PB-GT
MARCH6	membrane-associated ring finger (C3HC4) 6, E3 ubiquitin protein ligase [Source:HGNC Symbol;Acc:30550]	chr5	10353815	10435491	631	0.170	2	1	7.218E-04	3.609E-03	PB-GT
TRIM25	tripartite motif containing 25 [Source:HGNC Symbol;Acc:12932]	chr17	54965270	54991399	76	0.038	1	1	7.223E-04	3.609E-03	PB-GT
PJA2	praja ring finger 2, E3 ubiquitin protein ligase [Source:HGNC Symbol;Acc:17481]	chr5	108670410	108745695	632	0.170	2	2	7.251E-04	3.610E-03	PB-GT
COQ10B	coenzyme Q10 homolog B (S. cerevisiae) [Source:HGNC Symbol;Acc:25819]	chr2	198318147	198340032	135	0.039	1	1	7.263E-04	3.610E-03	PB-GT
DUSP11	dual specificity phosphatase 11 (RNA/RNP complex 1-interacting) [Source:HGNC Symbol;Acc:3066]	chr2	73989311	74007284	135	0.039	1	1	7.263E-04	3.610E-03	PB-GT
NARS	asparaginyl-tRNA synthetase [Source:HGNC Symbol;Acc:7643]	chr18	55267888	55289445	162	0.039	1	1	7.335E-04	3.640E-03	PB-GT
CORO7-PAM16	CORO7-PAM16 readthrough [Source:HGNC Symbol;Acc:44424]	chr16	4390252	4470495	116	0.039	1	1	7.357E-04	3.641E-03	PB-GT
TBC1D8	TBC1 domain family, member 8 (with GRAM domain) [Source:HGNC Symbol;Acc:17791]	chr2	101624079	101869328	1379	0.394	3	2	7.366E-04	3.641E-03	PB-GT
IQCB1	IQ motif containing B1 [Source:HGNC Symbol;Acc:28949]	chr3	121488610	121553926	557	0.171	2	1	7.377E-04	3.641E-03	PB-GT
FBF1	Fas (TNFRSF6) binding factor 1 [Source:HGNC Symbol;Acc:24674]	chr17	73905655	73937221	77	0.039	1	1	7.412E-04	3.652E-03	PB-GT
NR4A2	nuclear receptor subfamily 4, group A, member 2 [Source:HGNC Symbol;Acc:7981]	chr2	157180944	157198860	138	0.039	1	1	7.585E-04	3.731E-03	PB-GT
FAM3C	family with sequence similarity 3, member C [Source:HGNC Symbol;Acc:18664]	chr7	120988905	121036418	467	0.173	2	1	7.636E-04	3.747E-03	PB-GT
SETD9	SET domain containing 9 [Source:HGNC Symbol;Acc:28508]	chr5	56205087	56221359	147	0.040	1	1	7.643E-04	3.747E-03	PB-GT
PCBP1-AS1	PCBP1 antisense RNA 1 [Source:HGNC Symbol;Acc:42948]	chr2	70189395	70315978	607	0.174	2	2	7.658E-04	3.747E-03	PB-GT
ARL6IP5	ADP-ribosylation-like factor 6 interacting protein 5 [Source:HGNC Symbol;Acc:16937]	chr3	69134095	69155217	129	0.040	1	1	7.668E-04	3.747E-03	PB-GT
ETV3	ets variant 3 [Source:HGNC Symbol;Acc:3492]	chr1	157090983	157108266	112	0.040	1	1	7.697E-04	3.754E-03	PB-GT
FBXO34	F-box protein 34 [Source:HGNC Symbol;Acc:20201]	chr14	55738021	55828636	642	0.174	2	2	7.725E-04	3.761E-03	PB-GT
GPR75-ASB3	GPR75-ASB3 readthrough [Source:HGNC Symbol;Acc:40043]	chr2	53759810	54087170	2440	0.698	4	2	7.745E-04	3.765E-03	PB-GT
CCDC174	coiled-coil domain containing 174 [Source:HGNC Symbol;Acc:28033]	chr3	14693271	14714166	130	0.040	1	1	7.786E-04	3.778E-03	PB-GT
ZKSCAN8	zinc finger with KRAB and SCAN domains 8 [Source:HGNC Symbol;Acc:12983]	chr6	28109716	28124089	117	0.040	1	1	7.863E-04	3.801E-03	PB-GT
LRP1	low density lipoprotein receptor-related protein 1 [Source:HGNC Symbol;Acc:6692]	chr12	57522276	57607134	105	0.040	1	1	7.867E-04	3.801E-03	PB-GT
METAP1D	methionyl aminopeptidase type 1D (mitochondrial) [Source:HGNC Symbol;Acc:32583]	chr2	172864490	172947158	613	0.175	2	2	7.877E-04	3.801E-03	PB-GT
RBM7	RNA binding motif protein 7 [Source:HGNC Symbol;Acc:9904]	chr11	114270752	114284925	116	0.040	1	1	7.887E-04	3.801E-03	PB-GT
NCL	nucleolin [Source:HGNC Symbol;Acc:7667]	chr2	232318242	232348352	141	0.040	1	1	7.914E-04	3.804E-03	PB-GT
PRMT3	protein arginine methyltransferase 3 [Source:HGNC Symbol;Acc:30163]	chr11	20409076	20530840	1159	0.402	3	2	7.919E-04	3.804E-03	PB-GT
STMN1	stathmin 1 [Source:HGNC Symbol;Acc:6510]	chr1	26210672	26233482	114	0.040	1	1	7.971E-04	3.822E-03	PB-GT
TRIO	trio Rho guanine nucleotide exchange factor [Source:HGNC Symbol;Acc:12303]	chr5	14143811	14532235	2607	0.703	4	2	7.984E-04	3.822E-03	PB-GT
TMEM219	transmembrane protein 219 [Source:HGNC Symbol;Acc:25201]	chr16	29952206	29984373	121	0.041	1	1	7.996E-04	3.822E-03	PB-GT
RBM12B	RNA binding motif protein 12B [Source:HGNC Symbol;Acc:32310]	chr8	94741584	94753245	119	0.041	1	1	8.088E-04	3.859E-03	PB-GT
ATOX1	ATX1 antioxidant protein 1 homolog (yeast) [Source:HGNC Symbol;Acc:798]	chr5	151121877	151152093	152	0.041	1	1	8.164E-04	3.889E-03	PB-GT
SPCS3	signal peptidase complex subunit 3 homolog (S. cerevisiae) [Source:HGNC Symbol;Acc:26212]	chr4	177241115	177253396	145	0.041	1	1	8.200E-04	3.900E-03	PB-GT
SLC47A1	solute carrier family 47, member 1 [Source:HGNC Symbol;Acc:25588]	chr17	19398698	19482347	353	0.179	2	1	8.338E-04	3.955E-03	PB-GT
LETM2	leucine zipper-EF-hand containing transmembrane protein 2 [Source:HGNC Symbol;Acc:14648]	chr8	38243725	38267045	121	0.041	1	1	8.359E-04	3.955E-03	PB-GT
SYAP1	synapse associated protein 1 [Source:HGNC Symbol;Acc:16273]	chrX	16737755	16783459	249	0.041	1	1	8.371E-04	3.955E-03	PB-GT
SMIM11	small integral membrane protein 11 [Source:HGNC Symbol;Acc:1293]	chr21	35747779	35780164	145	0.041	1	1	8.372E-04	3.955E-03	PB-GT
AMBN	ameloblastin (enamel matrix protein) [Source:HGNC Symbol;Acc:452]	chr4	71457973	71473005	147	0.042	1	1	8.424E-04	3.974E-03	PB-GT
PXN	paxillin [Source:HGNC Symbol;Acc:9718]	chr12	120648250	120703574	109	0.042	1	1	8.469E-04	3.988E-03	PB-GT
IL20RB-AS1	IL20RB antisense RNA 1 [Source:HGNC Symbol;Acc:40298]	chr3	136677967	136701038	136	0.042	1	1	8.511E-04	3.995E-03	PB-GT
ZNF516	zinc finger protein 516 [Source:HGNC Symbol;Acc:28990]	chr18	74069644	74207146	752	0.180	2	1	8.512E-04	3.995E-03	PB-GT
STARD7	StAR-related lipid transfer (START) domain containing 7 [Source:HGNC Symbol;Acc:18063]	chr2	96850597	96874563	147	0.042	1	1	8.592E-04	4.014E-03	PB-GT
PER2	period circadian clock 2 [Source:HGNC Symbol;Acc:8846]	chr2	239152679	239198743	147	0.042	1	1	8.592E-04	4.014E-03	PB-GT
NARF	nuclear prelamin A recognition factor [Source:HGNC Symbol;Acc:29916]	chr17	80416056	80446143	83	0.042	1	1	8.594E-04	4.014E-03	PB-GT
NDFIP2	Nedd4 family interacting protein 2 [Source:HGNC Symbol;Acc:18537]	chr13	80055287	80130210	748	0.182	2	1	8.717E-04	4.064E-03	PB-GT
ZER1	zyg-11 related, cell cycle regulator [Source:HGNC Symbol;Acc:30960]	chr9	131492065	131534693	113	0.042	1	1	8.733E-04	4.065E-03	PB-GT
RTN3	reticulon 3 [Source:HGNC Symbol;Acc:10469]	chr11	63448918	63527363	524	0.182	2	2	8.747E-04	4.066E-03	PB-GT
GPRC5A	G protein-coupled receptor, family C, group 5, member A [Source:HGNC Symbol;Acc:9836]	chr12	13043716	13070871	111	0.042	1	1	8.778E-04	4.073E-03	PB-GT
KIF24	kinesin family member 24 [Source:HGNC Symbol;Acc:19916]	chr9	34252379	34329198	486	0.182	2	1	8.812E-04	4.075E-03	PB-GT
UBE2B	ubiquitin-conjugating enzyme E2B [Source:HGNC Symbol;Acc:12473]	chr5	133706870	133727683	158	0.043	1	1	8.812E-04	4.075E-03	PB-GT
DUSP19	dual specificity phosphatase 19 [Source:HGNC Symbol;Acc:18894]	chr2	183943287	183964733	149	0.043	1	1	8.824E-04	4.075E-03	PB-GT
ZNF649	zinc finger protein 649 [Source:HGNC Symbol;Acc:25741]	chr19	52392477	52408293	81	0.043	1	1	8.878E-04	4.090E-03	PB-GT
KBTBD8	kelch repeat and BTB (POZ) domain containing 8 [Source:HGNC Symbol;Acc:30691]	chr3	67048727	67061634	139	0.043	1	1	8.885E-04	4.090E-03	PB-GT
FKBP3	FK506 binding protein 3, 25kDa [Source:HGNC Symbol;Acc:3719]	chr14	45584803	45604522	158	0.043	1	1	8.923E-04	4.101E-03	PB-GT
GAS2L3	growth arrest-specific 2 like 3 [Source:HGNC Symbol;Acc:27475]	chr12	100967461	101022064	479	0.183	2	2	8.964E-04	4.107E-03	PB-GT
TMEM183A	transmembrane protein 183A [Source:HGNC Symbol;Acc:20173]	chr1	202976514	202993976	121	0.043	1	1	8.965E-04	4.107E-03	PB-GT
EGLN1	egl nine homolog 1 (C. elegans) [Source:HGNC Symbol;Acc:1232]	chr1	231499497	231560790	517	0.184	2	1	8.984E-04	4.109E-03	PB-GT
SHPK	sedoheptulokinase [Source:HGNC Symbol;Acc:1492]	chr17	3511556	3539616	85	0.043	1	1	9.007E-04	4.113E-03	PB-GT
RAG1	recombination activating gene 1 [Source:HGNC Symbol;Acc:9831]	chr11	36532259	36614706	531	0.184	2	1	9.086E-04	4.140E-03	PB-GT
SMG9	smg-9 homolog, nonsense mediated mRNA decay factor (C. elegans) [Source:HGNC Symbol;Acc:25763]	chr19	44235301	44259142	82	0.043	1	1	9.095E-04	4.140E-03	PB-GT
MED23	mediator complex subunit 23 [Source:HGNC Symbol;Acc:2372]	chr6	131895106	131949369	537	0.184	2	1	9.115E-04	4.142E-03	PB-GT
ARRDC3	arrestin domain containing 3 [Source:HGNC Symbol;Acc:29263]	chr5	90664541	90679176	161	0.043	1	1	9.145E-04	4.144E-03	PB-GT
ATP5S	ATP synthase, H+ transporting, mitochondrial Fo complex, subunit s (factor B) [Source:HGNC Symbol;Acc:18799]	chr14	50779044	50802276	160	0.043	1	1	9.147E-04	4.144E-03	PB-GT
NANS	N-acetylneuraminic acid synthase [Source:HGNC Symbol;Acc:19237]	chr9	100819021	100845357	116	0.044	1	1	9.196E-04	4.159E-03	PB-GT
NUP43	nucleoporin 43kDa [Source:HGNC Symbol;Acc:21182]	chr6	150045451	150070801	127	0.044	1	1	9.243E-04	4.174E-03	PB-GT
ABHD10	abhydrolase domain containing 10 [Source:HGNC Symbol;Acc:25656]	chr3	111697857	111712210	142	0.044	1	1	9.267E-04	4.175E-03	PB-GT
TFDP1	transcription factor Dp-1 [Source:HGNC Symbol;Acc:11749]	chr13	114239013	114295785	180	0.044	1	1	9.276E-04	4.175E-03	PB-GT
HNRNPH3	heterogeneous nuclear ribonucleoprotein H3 (2H9) [Source:HGNC Symbol;Acc:5043]	chr10	70090931	70102948	123	0.044	1	1	9.357E-04	4.206E-03	PB-GT
YIPF6	Yip1 domain family, member 6 [Source:HGNC Symbol;Acc:28304]	chrX	67718165	67757127	264	0.044	1	1	9.395E-04	4.216E-03	PB-GT
CCDC102B	coiled-coil domain containing 102B [Source:HGNC Symbol;Acc:26295]	chr18	66382446	66722426	3052	0.731	4	2	9.510E-04	4.261E-03	PB-GT
TMEM53	transmembrane protein 53 [Source:HGNC Symbol;Acc:26186]	chr1	45100910	45140227	125	0.044	1	1	9.559E-04	4.276E-03	PB-GT
CKAP2	cytoskeleton associated protein 2 [Source:HGNC Symbol;Acc:1990]	chr13	53029564	53050763	183	0.044	1	1	9.583E-04	4.280E-03	PB-GT
HNRNPA2B1	heterogeneous nuclear ribonucleoprotein A2/B1 [Source:HGNC Symbol;Acc:5033]	chr7	26229547	26241149	120	0.045	1	1	9.638E-04	4.298E-03	PB-GT
TRAK2	trafficking protein, kinesin binding 2 [Source:HGNC Symbol;Acc:13206]	chr2	202241930	202316302	659	0.188	2	2	9.691E-04	4.315E-03	PB-GT
SOD2	superoxide dismutase 2, mitochondrial [Source:HGNC Symbol;Acc:11180]	chr6	160100096	160183561	549	0.189	2	1	9.710E-04	4.317E-03	PB-GT
BCL7A	B-cell CLL/lymphoma 7A [Source:HGNC Symbol;Acc:1004]	chr12	122457328	122499948	117	0.045	1	1	9.738E-04	4.317E-03	PB-GT
PTER	phosphotriesterase related [Source:HGNC Symbol;Acc:9590]	chr10	16478942	16555736	529	0.189	2	2	9.741E-04	4.317E-03	PB-GT
PRPF4	PRP4 pre-mRNA processing factor 4 homolog (yeast) [Source:HGNC Symbol;Acc:17349]	chr9	116037623	116055185	120	0.045	1	1	9.831E-04	4.344E-03	PB-GT
UBE2D3	ubiquitin-conjugating enzyme E2D 3 [Source:HGNC Symbol;Acc:12476]	chr4	103715540	103790053	669	0.189	2	1	9.833E-04	4.344E-03	PB-GT
CEP135	centrosomal protein 135kDa [Source:HGNC Symbol;Acc:29086]	chr4	56815037	56899529	670	0.190	2	2	9.875E-04	4.350E-03	PB-GT
ACTR3C	ARP3 actin-related protein 3 homolog C (yeast) [Source:HGNC Symbol;Acc:37282]	chr7	149941005	150020814	511	0.190	2	1	9.884E-04	4.350E-03	PB-GT
RPL31	ribosomal protein L31 [Source:HGNC Symbol;Acc:10334]	chr2	101618177	101640494	158	0.045	1	1	9.905E-04	4.350E-03	PB-GT
FAM96A	family with sequence similarity 96, member A [Source:HGNC Symbol;Acc:26235]	chr15	64364758	64386217	130	0.045	1	1	9.917E-04	4.350E-03	PB-GT
TRIM42	tripartite motif containing 42 [Source:HGNC Symbol;Acc:19014]	chr3	140396881	140419992	147	0.045	1	1	9.921E-04	4.350E-03	PB-GT
TAF1B	TATA box binding protein (TBP)-associated factor, RNA polymerase I, B, 63kDa [Source:HGNC Symbol;Acc:11533]	chr2	9983483	10074545	665	0.190	2	2	9.946E-04	4.350E-03	PB-GT
PITX2	paired-like homeodomain 2 [Source:HGNC Symbol;Acc:9005]	chr4	111538579	111563279	160	0.045	1	1	9.956E-04	4.350E-03	PB-GT
WDFY3	WD repeat and FYVE domain containing 3 [Source:HGNC Symbol;Acc:20751]	chr4	85590704	85887544	2610	0.739	4	1	9.976E-04	4.350E-03	PB-GT
PAK2	p21 protein (Cdc42/Rac)-activated kinase 2 [Source:HGNC Symbol;Acc:8591]	chr3	196466728	196559518	619	0.190	2	2	9.982E-04	4.350E-03	PB-GT
CDKN2C	cyclin-dependent kinase inhibitor 2C (p18, inhibits CDK4) [Source:HGNC Symbol;Acc:1789]	chr1	51426417	51440305	128	0.045	1	1	1.002E-03	4.351E-03	PB-GT
FLG	filaggrin [Source:HGNC Symbol;Acc:3748]	chr1	152274651	152297679	128	0.045	1	1	1.002E-03	4.351E-03	PB-GT
TWIST2	twist basic helix-loop-helix transcription factor 2 [Source:HGNC Symbol;Acc:20670]	chr2	239756673	239795893	159	0.045	1	1	1.003E-03	4.351E-03	PB-GT
GSK3B	glycogen synthase kinase 3 beta [Source:HGNC Symbol;Acc:4617]	chr3	119540170	119813264	2407	0.740	4	2	1.006E-03	4.359E-03	PB-GT
RIMS3	regulating synaptic membrane exocytosis 3 [Source:HGNC Symbol;Acc:21292]	chr1	41086351	41131329	129	0.046	1	1	1.017E-03	4.399E-03	PB-GT
C19orf38	chromosome 19 open reading frame 38 [Source:HGNC Symbol;Acc:34073]	chr19	10947251	10980466	87	0.046	1	1	1.022E-03	4.413E-03	PB-GT
ARNTL	aryl hydrocarbon receptor nuclear translocator-like [Source:HGNC Symbol;Acc:701]	chr11	13298199	13408813	555	0.193	2	2	1.031E-03	4.446E-03	PB-GT
NARG2	NMDA receptor regulated 2 [Source:HGNC Symbol;Acc:29885]	chr15	60711808	60771359	555	0.193	2	1	1.038E-03	4.467E-03	PB-GT
WDR70	WD repeat domain 70 [Source:HGNC Symbol;Acc:25495]	chr5	37379314	37753537	2769	0.746	4	2	1.041E-03	4.477E-03	PB-GT
NCR3LG1	natural killer cell cytotoxicity receptor 3 ligand 1 [Source:HGNC Symbol;Acc:42400]	chr11	17373273	17398888	134	0.046	1	1	1.048E-03	4.486E-03	PB-GT
LINC00582	long intergenic non-protein coding RNA 582 [Source:HGNC Symbol;Acc:43842]	chr1	231727038	231747836	131	0.047	1	1	1.048E-03	4.486E-03	PB-GT
LIX1L	Lix1 homolog (mouse)-like [Source:HGNC Symbol;Acc:28715]	chr1	145477085	145501669	131	0.047	1	1	1.048E-03	4.486E-03	PB-GT
KIAA1467	KIAA1467 [Source:HGNC Symbol;Acc:29288]	chr12	13197218	13295455	507	0.194	2	2	1.055E-03	4.502E-03	PB-GT
KIAA1462	KIAA1462 [Source:HGNC Symbol;Acc:29283]	chr10	30301729	30404423	544	0.194	2	1	1.055E-03	4.502E-03	PB-GT
ZNF184	zinc finger protein 184 [Source:HGNC Symbol;Acc:12975]	chr6	27418522	27440897	136	0.047	1	1	1.058E-03	4.507E-03	PB-GT
GEMIN2	gem (nuclear organelle) associated protein 2 [Source:HGNC Symbol;Acc:10884]	chr14	39583427	39606177	173	0.047	1	1	1.067E-03	4.539E-03	PB-GT
ZMAT5	zinc finger, matrin-type 5 [Source:HGNC Symbol;Acc:28046]	chr22	30126945	30163000	94	0.047	1	1	1.069E-03	4.540E-03	PB-GT
PLRG1	pleiotropic regulator 1 [Source:HGNC Symbol;Acc:9089]	chr4	155456158	155471587	166	0.047	1	1	1.070E-03	4.540E-03	PB-GT
PIGG	phosphatidylinositol glycan anchor biosynthesis, class G [Source:HGNC Symbol;Acc:25985]	chr4	492989	533985	167	0.047	1	1	1.083E-03	4.588E-03	PB-GT
FKBP14	FK506 binding protein 14, 22 kDa [Source:HGNC Symbol;Acc:18625]	chr7	30050203	30066300	128	0.048	1	1	1.094E-03	4.628E-03	PB-GT
SARS	seryl-tRNA synthetase [Source:HGNC Symbol;Acc:10537]	chr1	109756540	109780791	134	0.048	1	1	1.096E-03	4.629E-03	PB-GT
FAM149B1	family with sequence similarity 149, member B1 [Source:HGNC Symbol;Acc:29162]	chr10	74927924	75004262	552	0.197	2	2	1.100E-03	4.638E-03	PB-GT
NOM1	nucleolar protein with MIF4G domain 1 [Source:HGNC Symbol;Acc:13244]	chr7	156742417	156765876	129	0.048	1	1	1.111E-03	4.679E-03	PB-GT
BTBD7	BTB (POZ) domain containing 7 [Source:HGNC Symbol;Acc:18269]	chr14	93703896	93799438	732	0.199	2	1	1.124E-03	4.727E-03	PB-GT
C3orf17	chromosome 3 open reading frame 17 [Source:HGNC Symbol;Acc:24496]	chr3	112721287	112738708	157	0.048	1	1	1.129E-03	4.736E-03	PB-GT
MMADHC	methylmalonic aciduria (cobalamin deficiency) cblD type, with homocystinuria [Source:HGNC Symbol;Acc:25221]	chr2	150426148	150444330	169	0.048	1	1	1.131E-03	4.736E-03	PB-GT
TGM5	transglutaminase 5 [Source:HGNC Symbol;Acc:11781]	chr15	43524793	43559055	139	0.048	1	1	1.131E-03	4.736E-03	PB-GT
ENOSF1	enolase superfamily member 1 [Source:HGNC Symbol;Acc:30365]	chr18	672543	712676	202	0.048	1	1	1.133E-03	4.736E-03	PB-GT
TTLL3	tubulin tyrosine ligase-like family, member 3 [Source:HGNC Symbol;Acc:24483]	chr3	9849770	9896822	158	0.049	1	1	1.144E-03	4.768E-03	PB-GT
CNNM4	cyclin M4 [Source:HGNC Symbol;Acc:105]	chr2	97426639	97477628	170	0.049	1	1	1.144E-03	4.768E-03	PB-GT
GPR113	G protein-coupled receptor 113 [Source:HGNC Symbol;Acc:18989]	chr2	26531041	26569685	171	0.049	1	1	1.157E-03	4.816E-03	PB-GT
ALDH2	aldehyde dehydrogenase 2 family (mitochondrial) [Source:HGNC Symbol;Acc:404]	chr12	112204691	112247782	128	0.049	1	1	1.162E-03	4.830E-03	PB-GT
SUPT5H	suppressor of Ty 5 homolog (S. cerevisiae) [Source:HGNC Symbol;Acc:11469]	chr19	39926796	39967310	93	0.049	1	1	1.165E-03	4.836E-03	PB-GT

table S2. Genes with significant transposon integrations in RKO *BRAF*$^{wt/-/-}$ cells after selection in low glucose medium

Gene ID	Description	Chrom	Start	End	# TTAA sites	Expected	Observed	Libraries	*P*	FDR	Transposon
FAM199X	family with sequence similarity 199, X-linked [Source:HGNC Symbol;Acc:25195]	chrX	103411301	103440583	295	0.049	1	1	1.169E-03	4.838E-03	PB-GT
NUDCD3	NudC domain containing 3 [Source:HGNC Symbol;Acc:22208]	chr7	44421969	44530479	542	0.201	2	1	1.169E-03	4.838E-03	PB-GT
MGME1	mitochondrial genome maintenance exonuclease 1 [Source:HGNC Symbol;Acc:16205]	chr20	17949556	17971765	136	0.049	1	1	1.175E-03	4.854E-03	PB-GT
MIAT	myocardial infarction associated transcript (non-protein coding) [Source:HGNC Symbol;Acc:33425]	chr22	27042392	27072438	99	0.049	1	1	1.184E-03	4.883E-03	PB-GT
SLC38A2	solute carrier family 38, member 2 [Source:HGNC Symbol;Acc:13448]	chr12	46751972	46766650	130	0.050	1	1	1.198E-03	4.936E-03	PB-GT
CCDC69	coiled-coil domain containing 69 [Source:HGNC Symbol;Acc:24487]	chr5	150560613	150603706	185	0.050	1	1	1.202E-03	4.945E-03	PB-GT
SETD5	SET domain containing 5 [Source:HGNC Symbol;Acc:25566]	chr3	9439299	9520924	661	0.203	2	1	1.204E-03	4.945E-03	PB-GT
MOB1A	MOB kinase activator 1A [Source:HGNC Symbol;Acc:16015]	chr2	74382165	74406025	175	0.050	1	1	1.211E-03	4.968E-03	PB-GT
RWDD4	RWD domain containing 4 [Source:HGNC Symbol;Acc:23750]	chr4	184560788	184580378	177	0.050	1	1	1.215E-03	4.974E-03	PB-GT
NAA60	N(alpha)-acetyltransferase 60, NatF catalytic subunit [Source:HGNC Symbol;Acc:25875]	chr16	3493611	3536963	150	0.050	1	1	1.221E-03	4.992E-03	PB-GT
GSN	gelsolin [Source:HGNC Symbol;Acc:4620]	chr9	123970078	124095121	545	0.204	2	2	1.222E-03	4.992E-03	PB-GT
IKZF5	IKAROS family zinc finger 5 (Pegasus) [Source:HGNC Symbol;Acc:14283]	chr10	124750322	124768333	141	0.050	1	1	1.224E-03	4.994E-03	PB-GT
EGF	epidermal growth factor [Source:HGNC Symbol;Acc:3229]	chr4	110834040	110933422	725	0.205	2	2	1.237E-03	5.037E-03	PB-GT
RBM14-RBM4	RBM14-RBM4 readthrough [Source:HGNC Symbol;Acc:38840]	chr11	66384097	66413940	146	0.051	1	1	1.241E-03	5.046E-03	PB-GT
MYNN	myoneurin [Source:HGNC Symbol;Acc:14955]	chr3	169490619	169507504	165	0.051	1	1	1.245E-03	5.046E-03	PB-GT
UBE2E1	ubiquitin-conjugating enzyme E2E 1 [Source:HGNC Symbol;Acc:12477]	chr3	23847394	23932807	669	0.206	2	2	1.246E-03	5.046E-03	PB-GT
SPATA5L1	spermatogenesis associated 5-like 1 [Source:HGNC Symbol;Acc:28762]	chr15	45694529	45713617	146	0.051	1	1	1.246E-03	5.046E-03	PB-GT
ZFHX3	zinc finger homeobox 3 [Source:HGNC Symbol;Acc:777]	chr16	72816784	73093597	1360	0.456	3	2	1.250E-03	5.056E-03	PB-GT
AAMDC	adipogenesis associated, Mth938 domain containing [Source:HGNC Symbol;Acc:30205]	chr11	77532155	77629478	596	0.207	2	1	1.263E-03	5.102E-03	PB-GT
TGDS	TDP-glucose 4,6-dehydratase [Source:HGNC Symbol;Acc:20324]	chr13	95226308	95248511	211	0.051	1	1	1.268E-03	5.110E-03	PB-GT
ZNF775	zinc finger protein 775 [Source:HGNC Symbol;Acc:28501]	chr7	150065879	150109558	138	0.051	1	1	1.269E-03	5.110E-03	PB-GT
EHHADH-AS1	EHHADH antisense RNA 1 [Source:HGNC Symbol;Acc:44133]	chr3	184880659	184909743	167	0.051	1	1	1.275E-03	5.121E-03	PB-GT
C3orf14	chromosome 3 open reading frame 14 [Source:HGNC Symbol;Acc:25024]	chr3	62304648	62321888	167	0.051	1	1	1.275E-03	5.121E-03	PB-GT
COMMD7	COMM domain containing 7 [Source:HGNC Symbol;Acc:16223]	chr20	31290493	31331814	142	0.051	1	1	1.279E-03	5.126E-03	PB-GT
CREG1	cellular repressor of E1A-stimulated genes 1 [Source:HGNC Symbol;Acc:2351]	chr1	167498914	167523004	145	0.051	1	1	1.280E-03	5.126E-03	PB-GT
LTV1	LTV1 homolog (S. cerevisiae) [Source:HGNC Symbol;Acc:21173]	chr6	144164481	144184949	150	0.052	1	1	1.283E-03	5.129E-03	PB-GT
LYRM5	LYR motif containing 5 [Source:HGNC Symbol;Acc:27052]	chr12	25348150	25362579	135	0.052	1	1	1.291E-03	5.151E-03	PB-GT
SMIM7	small integral membrane protein 7 [Source:HGNC Symbol;Acc:28419]	chr19	16741562	16771253	98	0.052	1	1	1.292E-03	5.151E-03	PB-GT
FEM1B	fem-1 homolog b (C. elegans) [Source:HGNC Symbol;Acc:3649]	chr15	68570141	68588203	149	0.052	1	1	1.297E-03	5.157E-03	PB-GT
KIAA0101	KIAA0101 [Source:HGNC Symbol;Acc:28961]	chr15	64657193	64679886	149	0.052	1	1	1.297E-03	5.157E-03	PB-GT
SKI	v-ski sarcoma viral oncogene homolog (avian) [Source:HGNC Symbol;Acc:10896]	chr1	2160134	2241558	147	0.052	1	1	1.315E-03	5.218E-03	PB-GT
PCID2	PCI domain containing 2 [Source:HGNC Symbol;Acc:25653]	chr13	113831891	113863029	215	0.052	1	1	1.316E-03	5.218E-03	PB-GT
MED27	mediator complex subunit 27 [Source:HGNC Symbol;Acc:2377]	chr9	134735494	134955295	1234	0.463	3	2	1.325E-03	5.245E-03	PB-GT
MAP3K1	mitogen-activated protein kinase kinase kinase 1, E3 ubiquitin protein ligase [Source:HGNC Symbol;Acc:6848]	chr5	56111401	56191979	787	0.212	2	1	1.357E-03	5.368E-03	PB-GT
ZNF766	zinc finger protein 766 [Source:HGNC Symbol;Acc:28063]	chr19	52772824	52795977	101	0.053	1	1	1.371E-03	5.405E-03	PB-GT
ZNF528	zinc finger protein 528 [Source:HGNC Symbol;Acc:29384]	chr19	52901102	52921665	101	0.053	1	1	1.371E-03	5.405E-03	PB-GT
WEE1	WEE1 homolog (S. pombe) [Source:HGNC Symbol;Acc:12761]	chr11	9595228	9615004	154	0.053	1	1	1.378E-03	5.426E-03	PB-GT
THAP4	THAP domain containing 4 [Source:HGNC Symbol;Acc:23187]	chr2	242523820	242576864	188	0.054	1	1	1.394E-03	5.484E-03	PB-GT
LRRC23	leucine rich repeat containing 23 [Source:HGNC Symbol;Acc:19138]	chr12	6982733	7023407	141	0.054	1	1	1.406E-03	5.521E-03	PB-GT
DARS	aspartyl-tRNA synthetase [Source:HGNC Symbol;Acc:2678]	chr2	136664247	136743670	753	0.215	2	1	1.417E-03	5.552E-03	PB-GT
ZKSCAN1	zinc finger with KRAB and SCAN domains 1 [Source:HGNC Symbol;Acc:13101]	chr7	99613204	99639312	146	0.054	1	1	1.418E-03	5.552E-03	PB-GT
PRPF3	PRP3 pre-mRNA processing factor 3 homolog (S. cerevisiae) [Source:HGNC Symbol;Acc:17348]	chr1	150293925	150325671	153	0.054	1	1	1.423E-03	5.556E-03	PB-GT
UBR5	ubiquitin protein ligase E3 component n-recognin 5 [Source:HGNC Symbol;Acc:16806]	chr8	103265240	103425069	1378	0.472	3	2	1.423E-03	5.556E-03	PB-GT
ZNF510	zinc finger protein 510 [Source:HGNC Symbol;Acc:29161]	chr9	99518147	99540411	145	0.054	1	1	1.426E-03	5.556E-03	PB-GT
LRSAM1	leucine rich repeat and sterile alpha motif containing 1 [Source:HGNC Symbol;Acc:25135]	chr9	130213765	130265780	145	0.054	1	1	1.426E-03	5.556E-03	PB-GT
SLC25A32	solute carrier family 25 (mitochondrial folate carrier), member 32 [Source:HGNC Symbol;Acc:29683]	chr8	104410863	104427417	159	0.054	1	1	1.431E-03	5.566E-03	PB-GT
PCDH9-AS2	PCDH9 antisense RNA 2 [Source:HGNC Symbol;Acc:39896]	chr13	67399301	67489163	891	0.216	2	1	1.436E-03	5.577E-03	PB-GT
CIRH1A	cirrhosis, autosomal recessive 1A (cirhin) [Source:HGNC Symbol;Acc:1983]	chr16	69165194	69202941	164	0.055	1	1	1.455E-03	5.637E-03	PB-GT
NUPL2	nucleoporin like 2 [Source:HGNC Symbol;Acc:17010]	chr7	23221446	23240630	148	0.055	1	1	1.456E-03	5.637E-03	PB-GT
NECAB1	N-terminal EF-hand calcium binding protein 1 [Source:HGNC Symbol;Acc:20983]	chr8	91803778	91971636	1387	0.475	3	2	1.457E-03	5.637E-03	PB-GT
ERRFI1	ERBB receptor feedback inhibitor 1 [Source:HGNC Symbol;Acc:18185]	chr1	8064464	8086368	155	0.055	1	1	1.459E-03	5.639E-03	PB-GT
ADPRM	ADP-ribose/CDP-alcohol diphosphatase, manganese-dependent [Source:HGNC Symbol;Acc:30925]	chr17	10600911	10614550	109	0.055	1	1	1.469E-03	5.669E-03	PB-GT
USO1	USO1 vesicle transport factor [Source:HGNC Symbol;Acc:30904]	chr4	76649777	76735382	771	0.218	2	2	1.473E-03	5.677E-03	PB-GT
CATSPER3	cation channel, sperm associated 3 [Source:HGNC Symbol;Acc:20819]	chr5	134303596	134347392	206	0.056	1	1	1.485E-03	5.715E-03	PB-GT
KDM4C	lysine (K)-specific demethylase 4C [Source:HGNC Symbol;Acc:17071]	chr9	6720863	7175648	3194	1.198	5	2	1.488E-03	5.718E-03	PB-GT
TIMM23B	translocase of inner mitochondrial membrane 23 homolog B (yeast) [Source:HGNC Symbol;Acc:23581]	chr10	51371390	51387768	156	0.056	1	1	1.493E-03	5.732E-03	PB-GT
C4orf21	chromosome 4 open reading frame 21 [Source:HGNC Symbol;Acc:25654]	chr4	113460492	113558151	777	0.220	2	2	1.506E-03	5.772E-03	PB-GT
PSAP	prosaposin [Source:HGNC Symbol;Acc:9498]	chr10	73576055	73611126	157	0.056	1	1	1.512E-03	5.788E-03	PB-GT
NBR2	neighbor of BRCA1 gene 2 (non-protein coding) [Source:HGNC Symbol;Acc:20691]	chr17	41277627	41305688	111	0.056	1	1	1.523E-03	5.820E-03	PB-GT
PKM	pyruvate kinase, muscle [Source:HGNC Symbol;Acc:9021]	chr15	72491370	72524164	162	0.056	1	1	1.529E-03	5.828E-03	PB-GT
FAM221A	family with sequence similarity 221, member A [Source:HGNC Symbol;Acc:27977]	chr7	23719749	23742868	152	0.056	1	1	1.534E-03	5.828E-03	PB-GT
USP6NL	USP6 N-terminal like [Source:HGNC Symbol;Acc:16858]	chr10	11502509	11653753	1351	0.482	3	2	1.536E-03	5.828E-03	PB-GT
TXLNG	taxilin gamma [Source:HGNC Symbol;Acc:18578]	chrX	16804550	16862642	339	0.056	1	1	1.536E-03	5.828E-03	PB-GT
MYO9A	myosin IXA [Source:HGNC Symbol;Acc:7608]	chr15	72114632	72410918	2346	0.816	4	2	1.537E-03	5.828E-03	PB-GT
SPTLC2	serine palmitoyltransferase, long chain base subunit 2 [Source:HGNC Symbol;Acc:11278]	chr14	77972340	78083116	817	0.222	2	2	1.537E-03	5.828E-03	PB-GT
BICD2	bicaudal D homolog 2 (Drosophila) [Source:HGNC Symbol;Acc:17208]	chr9	95473645	95527094	151	0.057	1	1	1.545E-03	5.833E-03	PB-GT
CAMK2D	calcium/calmodulin-dependent protein kinase II delta [Source:HGNC Symbol;Acc:1462]	chr4	114372188	114683083	2885	0.817	4	2	1.545E-03	5.833E-03	PB-GT
HEBP1	heme binding protein 1 [Source:HGNC Symbol;Acc:17176]	chr12	13127798	13153207	148	0.057	1	1	1.546E-03	5.833E-03	PB-GT
STX17	syntaxin 17 [Source:HGNC Symbol;Acc:11432]	chr9	102668915	102732618	592	0.222	2	2	1.546E-03	5.833E-03	PB-GT
STRADB	STE20-related kinase adaptor beta [Source:HGNC Symbol;Acc:13205]	chr2	202252581	202345569	778	0.222	2	2	1.555E-03	5.843E-03	PB-GT
SPEN	spen homolog, transcriptional regulator (Drosophila) [Source:HGNC Symbol;Acc:17575]	chr1	16174359	16266955	627	0.223	2	1	1.557E-03	5.843E-03	PB-GT
SLC26A2	solute carrier family 26 (sulfate transporter), member 2 [Source:HGNC Symbol;Acc:10994]	chr5	149340300	149373018	211	0.057	1	1	1.557E-03	5.843E-03	PB-GT
TBL1X	transducin (beta)-like 1X-linked [Source:HGNC Symbol;Acc:11585]	chrX	9431335	9687780	1336	0.223	2	1	1.557E-03	5.843E-03	PB-GT
UBN1	ubinuclein 1 [Source:HGNC Symbol;Acc:12506]	chr16	4896666	4932361	171	0.057	1	1	1.579E-03	5.907E-03	PB-GT
MARK3	MAP/microtubule affinity-regulating kinase 3 [Source:HGNC Symbol;Acc:6897]	chr14	103851729	103970168	825	0.224	2	2	1.580E-03	5.907E-03	PB-GT
PHLPP1	PH domain and leucine rich repeat protein phosphatase 1 [Source:HGNC Symbol;Acc:20610]	chr18	60382672	60647666	2029	0.486	3	2	1.580E-03	5.907E-03	PB-GT
XPO6	exportin 6 [Source:HGNC Symbol;Acc:19733]	chr16	28109300	28223241	669	0.224	2	1	1.587E-03	5.925E-03	PB-GT
C1orf198	chromosome 1 open reading frame 198 [Source:HGNC Symbol;Acc:25900]	chr1	230972865	231005335	162	0.058	1	1	1.592E-03	5.933E-03	PB-GT
NEU3	sialidase 3 (membrane sialidase) [Source:HGNC Symbol;Acc:7760]	chr11	74699179	74729938	166	0.058	1	1	1.597E-03	5.944E-03	PB-GT
BAZ2A	bromodomain adjacent to zinc finger domain, 2A [Source:HGNC Symbol;Acc:962]	chr12	56989380	57030600	151	0.058	1	1	1.608E-03	5.979E-03	PB-GT
RARS2	arginyl-tRNA synthetase 2, mitochondrial [Source:HGNC Symbol;Acc:21406]	chr6	88224096	88299721	656	0.225	2	1	1.612E-03	5.986E-03	PB-GT
LRP5L	low density lipoprotein receptor-related protein 5-like [Source:HGNC Symbol;Acc:25323]	chr22	25747385	25801344	116	0.058	1	1	1.616E-03	5.992E-03	PB-GT
FAM129B	family with sequence similarity 129, member B [Source:HGNC Symbol;Acc:25282]	chr9	130267618	130341268	155	0.058	1	1	1.626E-03	6.022E-03	PB-GT
PYGB	phosphorylase, glycogen; brain [Source:HGNC Symbol;Acc:9723]	chr20	25228705	25278650	161	0.058	1	1	1.637E-03	6.047E-03	PB-GT
ZC3H15	zinc finger CCCH-type containing 15 [Source:HGNC Symbol;Acc:29528]	chr2	187350883	187374090	204	0.058	1	1	1.637E-03	6.047E-03	PB-GT
C9orf3	chromosome 9 open reading frame 3 [Source:HGNC Symbol;Acc:1361]	chr9	97488983	97849441	2208	0.828	4	2	1.640E-03	6.047E-03	PB-GT
PCNP	PEST proteolytic signal containing nuclear protein [Source:HGNC Symbol;Acc:30023]	chr3	101292939	101313281	190	0.058	1	1	1.643E-03	6.047E-03	PB-GT
PISD	phosphatidylserine decarboxylase [Source:HGNC Symbol;Acc:8999]	chr22	32014477	32058418	117	0.058	1	1	1.643E-03	6.047E-03	PB-GT
FAM222B	family with sequence similarity 222, member B [Source:HGNC Symbol;Acc:25563]	chr17	27082996	27182250	449	0.227	2	1	1.655E-03	6.082E-03	PB-GT
POM121L2	POM121 transmembrane nucleoporin-like 2 [Source:HGNC Symbol;Acc:13973]	chr6	27253682	27279949	171	0.059	1	1	1.659E-03	6.082E-03	PB-GT
RAB40B	RAB40B, member RAS oncogene family [Source:HGNC Symbol;Acc:18284]	chr17	80612849	80656604	116	0.059	1	1	1.660E-03	6.082E-03	PB-GT
C6orf183	chromosome 6 open reading frame 183 [Source:HGNC Symbol;Acc:21562]	chr6	109487036	109592217	663	0.228	2	1	1.661E-03	6.082E-03	PB-GT
EMC1	ER membrane protein complex subunit 1 [Source:HGNC Symbol;Acc:28957]	chr1	19542158	19578046	166	0.059	1	1	1.670E-03	6.097E-03	PB-GT
PTPRF	protein tyrosine phosphatase, receptor type, F [Source:HGNC Symbol;Acc:9670]	chr1	43990858	44089343	166	0.059	1	1	1.670E-03	6.097E-03	PB-GT
RP9P	retinitis pigmentosa 9 pseudogene [Source:HGNC Symbol;Acc:33969]	chr7	32956427	32982788	159	0.059	1	1	1.676E-03	6.112E-03	PB-GT
CORO1C	coronin, actin binding protein, 1C [Source:HGNC Symbol;Acc:2254]	chr12	109038885	109125372	597	0.229	2	1	1.678E-03	6.113E-03	PB-GT
MEIG1	meiosis expressed gene 1 homolog (mouse) [Source:HGNC Symbol;Acc:23429]	chr10	15001438	15030049	166	0.059	1	1	1.687E-03	6.131E-03	PB-GT
MDS2	myelodysplastic syndrome 2 translocation associated [Source:HGNC Symbol;Acc:29633]	chr1	23907985	23967058	167	0.059	1	1	1.689E-03	6.131E-03	PB-GT
SRP19	signal recognition particle 19kDa [Source:HGNC Symbol;Acc:11300]	chr5	112196885	112228776	220	0.059	1	1	1.690E-03	6.131E-03	PB-GT
MMD2	monocyte to macrophage differentiation-associated 2 [Source:HGNC Symbol;Acc:30133]	chr7	4945620	4998844	160	0.059	1	1	1.697E-03	6.149E-03	PB-GT
KCNK12	potassium channel, subfamily K, member 12 [Source:HGNC Symbol;Acc:6274]	chr2	47747910	47798078	208	0.059	1	1	1.700E-03	6.155E-03	PB-GT
ZNF433	zinc finger protein 433 [Source:HGNC Symbol;Acc:20811]	chr19	12125547	12146556	113	0.060	1	1	1.709E-03	6.176E-03	PB-GT
RNFT1	ring finger protein, transmembrane 1 [Source:HGNC Symbol;Acc:30206]	chr17	58029601	58042122	118	0.060	1	1	1.717E-03	6.198E-03	PB-GT
ZMYM5	zinc finger, MYM-type 5 [Source:HGNC Symbol;Acc:13029]	chr13	20397622	20437776	247	0.060	1	1	1.728E-03	6.230E-03	PB-GT
TLR6	toll-like receptor 6 [Source:HGNC Symbol;Acc:16711]	chr4	38825336	38858438	212	0.060	1	1	1.731E-03	6.233E-03	PB-GT
EZH2	enhancer of zeste homolog 2 (Drosophila) [Source:HGNC Symbol;Acc:3527]	chr7	148504475	148581413	623	0.231	2	2	1.737E-03	6.237E-03	PB-GT
XRCC1	X-ray repair complementing defective repair in Chinese hamster cells 1 [Source:HGNC Symbol;Acc:12828]	chr19	44047192	44084625	114	0.060	1	1	1.738E-03	6.237E-03	PB-GT
KDELR2	KDEL (Lys-Asp-Glu-Leu) endoplasmic reticulum protein retention receptor 2 [Source:HGNC Symbol;Acc:6305]	chr7	6485584	6523873	162	0.060	1	1	1.738E-03	6.237E-03	PB-GT
METTL8	methyltransferase like 8 [Source:HGNC Symbol;Acc:25856]	chr2	172179761	172291312	812	0.232	2	1	1.755E-03	6.289E-03	PB-GT
ASAP1	ArfGAP with SH3 domain, ankyrin repeat and PH domain 1 [Source:HGNC Symbol;Acc:2720]	chr8	131064353	131455906	2457	0.842	4	2	1.759E-03	6.296E-03	PB-GT
CLTA	clathrin, light chain A [Source:HGNC Symbol;Acc:2090]	chr9	36190853	36304778	620	0.233	2	1	1.763E-03	6.300E-03	PB-GT
RQCD1	RCD1 required for cell differentiation1 homolog (S. pombe) [Source:HGNC Symbol;Acc:10445]	chr2	219433303	219461803	212	0.061	1	1	1.765E-03	6.301E-03	PB-GT
ZNF69	zinc finger protein 69 [Source:HGNC Symbol;Acc:13138]	chr19	11998599	12025144	115	0.061	1	1	1.768E-03	6.305E-03	PB-GT
ADNP2	ADNP homeobox 2 [Source:HGNC Symbol;Acc:23803]	chr18	77866915	77905406	254	0.061	1	1	1.777E-03	6.328E-03	PB-GT
CUBNP3	cubilin (intrinsic factor-cobalamin receptor) pseudogene 3 [Source:HGNC Symbol;Acc:44985]	chr10	45622766	45644362	171	0.061	1	1	1.788E-03	6.349E-03	PB-GT
YBX1	Y box binding protein 1 [Source:HGNC Symbol;Acc:8014]	chr1	43148098	43168020	172	0.061	1	1	1.790E-03	6.349E-03	PB-GT
BDP1	B double prime 1, subunit of RNA polymerase III transcription initiation factor IIIB [Source:HGNC Symbol;Acc:13652]	chr5	70751442	70863649	869	0.234	2	2	1.798E-03	6.349E-03	PB-GT
ACTL6A	actin-like 6A [Source:HGNC Symbol;Acc:24124]	chr3	179280668	179306196	199	0.061	1	1	1.799E-03	6.349E-03	PB-GT
KLRC2	killer cell lectin-like receptor subfamily C, member 2 [Source:HGNC Symbol;Acc:6375]	chr12	10579453	10594899	160	0.061	1	1	1.801E-03	6.349E-03	PB-GT
NSMCE1	non-SMC element 1 homolog (S. cerevisiae) [Source:HGNC Symbol;Acc:29897]	chr16	27236312	27280115	183	0.061	1	1	1.804E-03	6.349E-03	PB-GT
ZSCAN30	zinc finger and SCAN domain containing 30 [Source:HGNC Symbol;Acc:33517]	chr18	32831032	32870196	256	0.061	1	1	1.805E-03	6.349E-03	PB-GT
CHRNB3	cholinergic receptor, nicotinic, beta 3 (neuronal) [Source:HGNC Symbol;Acc:1963]	chr8	42552519	42592550	179	0.061	1	1	1.805E-03	6.349E-03	PB-GT
GOLGA7	golgin A7 [Source:HGNC Symbol;Acc:24876]	chr8	41347915	41368499	179	0.061	1	1	1.805E-03	6.349E-03	PB-GT
OTUD6B	OTU domain containing 6B [Source:HGNC Symbol;Acc:24281]	chr8	92082424	92099323	179	0.061	1	1	1.805E-03	6.349E-03	PB-GT
ARL13B	ADP-ribosylation factor-like 13B [Source:HGNC Symbol;Acc:25419]	chr3	93698983	93774512	763	0.235	2	2	1.809E-03	6.355E-03	PB-GT
CMC1	COX assembly mitochondrial protein 1 homolog (S. cerevisiae) [Source:HGNC Symbol;Acc:28783]	chr3	28283075	28366633	765	0.235	2	1	1.823E-03	6.395E-03	PB-GT
MAP4K3	mitogen-activated protein kinase kinase kinase kinase 3 [Source:HGNC Symbol;Acc:6865]	chr2	39476407	39664453	1769	0.506	3	1	1.827E-03	6.403E-03	PB-GT
LEO1	Leo1, Paf1/RNA polymerase II complex component, homolog (S. cerevisiae) [Source:HGNC Symbol;Acc:30401]	chr15	52230222	52264003	178	0.062	1	1	1.839E-03	6.435E-03	PB-GT
ANKRD17	ankyrin repeat domain 17 [Source:HGNC Symbol;Acc:23575]	chr4	73939093	74124515	1791	0.507	3	1	1.843E-03	6.436E-03	PB-GT
SDHA	succinate dehydrogenase complex, subunit A, flavoprotein (Fp) [Source:HGNC Symbol;Acc:10680]	chr5	218356	256815	230	0.062	1	1	1.843E-03	6.436E-03	PB-GT
INTU	inturned planar cell polarity protein [Source:HGNC Symbol;Acc:29239]	chr4	128544426	128637930	835	0.236	2	1	1.846E-03	6.439E-03	PB-GT
NMNAT1	nicotinamide nucleotide adenylyltransferase 1 [Source:HGNC Symbol;Acc:17877]	chr1	10003486	10045559	175	0.062	1	1	1.852E-03	6.449E-03	PB-GT
GM2A	GM2 ganglioside activator [Source:HGNC Symbol;Acc:4367]	chr5	150591711	150650001	231	0.062	1	1	1.859E-03	6.462E-03	PB-GT
TMCO1	transmembrane and coiled-coil domains 1 [Source:HGNC Symbol;Acc:18188]	chr1	165696032	165796992	668	0.237	2	2	1.862E-03	6.462E-03	PB-GT
SERINC3	serine incorporator 3 [Source:HGNC Symbol;Acc:11699]	chr20	43124862	43150750	172	0.062	1	1	1.863E-03	6.462E-03	PB-GT
POLR1B	polymerase (RNA) I polypeptide B, 128kDa [Source:HGNC Symbol;Acc:20454]	chr2	113299492	113334635	218	0.062	1	1	1.864E-03	6.462E-03	PB-GT
ABCB9	ATP-binding cassette, sub-family B (MDR/TAP), member 9 [Source:HGNC Symbol;Acc:50]	chr12	123405498	123466196	163	0.062	1	1	1.868E-03	6.467E-03	PB-GT
PAFAH1B2	platelet-activating factor acetylhydrolase 1b, catalytic subunit 2 (30kDa) [Source:HGNC Symbol;Acc:8575]	chr11	117014983	117047610	180	0.062	1	1	1.871E-03	6.470E-03	PB-GT

table S2. Genes with significant transposon integrations in RKO *BRAF*$^{wt/-/-}$ cells after selection in low glucose medium

Gene ID	Description	Chrom	Start	End	# TTAA sites	Expected	Observed	Libraries	*P*	FDR	Transposon
CDK19	cyclin-dependent kinase 19 [Source:HGNC Symbol;Acc:19338]	chr6	110931181	111137161	1484	0.510	3	2	1.878E-03	6.478E-03	PB-GT
ABCC1	ATP-binding cassette, sub-family C (CFTR/MRP), member 1 [Source:HGNC Symbol;Acc:51]	chr16	16043434	16236931	710	0.238	2	1	1.878E-03	6.478E-03	PB-GT
HIF3A	hypoxia inducible factor 3, alpha subunit [Source:HGNC Symbol;Acc:15825]	chr19	46800303	46846690	119	0.063	1	1	1.891E-03	6.515E-03	PB-GT
MELK	maternal embryonic leucine zipper kinase [Source:HGNC Symbol;Acc:16870]	chr9	36572859	36677678	636	0.239	2	2	1.894E-03	6.518E-03	PB-GT
DCAF12	DDB1 and CUL4 associated factor 12 [Source:HGNC Symbol;Acc:19911]	chr9	34086385	34127397	168	0.063	1	1	1.904E-03	6.535E-03	PB-GT
ARHGAP40	Rho GTPase activating protein 40 [Source:HGNC Symbol;Acc:16226]	chr20	37230577	37279678	174	0.063	1	1	1.906E-03	6.535E-03	PB-GT
YWHAB	tyrosine 3-monooxygenase/tryptophan 5-monooxygenase activation protein, beta polypeptide [Source:HGNC Symbol;Acc:1284	chr20	43514317	43537173	174	0.063	1	1	1.906E-03	6.535E-03	PB-GT
KCNA1	potassium voltage-gated channel, shaker-related subfamily, member 1 (episodic ataxia with myokymia) [Source:HGNC Symbol;	chr12	5019071	5040527	165	0.063	1	1	1.913E-03	6.544E-03	PB-GT
METTL20	methyltransferase like 20 [Source:HGNC Symbol;Acc:28739]	chr12	31800094	31826048	165	0.063	1	1	1.913E-03	6.544E-03	PB-GT
SPIN1	spindlin 1 [Source:HGNC Symbol;Acc:11243]	chr9	91003334	91093609	639	0.240	2	2	1.919E-03	6.558E-03	PB-GT
EDEM1	ER degradation enhancer, mannosidase alpha-like 1 [Source:HGNC Symbol;Acc:18967]	chr3	5229331	5261642	206	0.063	1	1	1.925E-03	6.563E-03	PB-GT
SPG11	spastic paraplegia 11 (autosomal recessive) [Source:HGNC Symbol;Acc:11226]	chr15	44854894	44955876	690	0.240	2	1	1.926E-03	6.563E-03	PB-GT
ZNF37A	zinc finger protein 37A [Source:HGNC Symbol;Acc:13102]	chr10	38383264	38412276	178	0.064	1	1	1.934E-03	6.585E-03	PB-GT
ANXA1	annexin A1 [Source:HGNC Symbol;Acc:533]	chr9	75766673	75785309	170	0.064	1	1	1.949E-03	6.626E-03	PB-GT
TARS	threonyl-tRNA synthetase [Source:HGNC Symbol;Acc:11572]	chr5	33440802	33469644	237	0.064	1	1	1.955E-03	6.639E-03	PB-GT
GGH	gamma-glutamyl hydrolase (conjugase, folylpolygammaglutamyl hydrolase) [Source:HGNC Symbol;Acc:4248]	chr8	63927638	63951730	187	0.064	1	1	1.967E-03	6.671E-03	PB-GT
TP53TG5	TP53 target 5 [Source:HGNC Symbol;Acc:15856]	chr20	44002526	44036529	177	0.064	1	1	1.971E-03	6.672E-03	PB-GT
DUSP16	dual specificity phosphatase 16 [Source:HGNC Symbol;Acc:17909]	chr12	12628829	12715317	632	0.242	2	2	1.971E-03	6.672E-03	PB-GT
HKDC1	hexokinase domain containing 1 [Source:HGNC Symbol;Acc:23302]	chr10	70980059	71027315	180	0.064	1	1	1.977E-03	6.683E-03	PB-GT
HMGCR	3-hydroxy-3-methylglutaryl-CoA reductase [Source:HGNC Symbol;Acc:5006]	chr5	74632154	74657929	239	0.064	1	1	1.987E-03	6.710E-03	PB-GT
PGAP1	post-GPI attachment to proteins 1 [Source:HGNC Symbol;Acc:25712]	chr2	197699908	197792520	850	0.243	2	1	1.997E-03	6.719E-03	PB-GT
PARK7	parkinson protein 7 [Source:HGNC Symbol;Acc:16369]	chr1	8014351	8045565	182	0.065	1	1	1.999E-03	6.719E-03	PB-GT
GNL2	guanine nucleotide binding protein-like 2 (nucleolar) [Source:HGNC Symbol;Acc:29925]	chr1	38032417	38061536	182	0.065	1	1	1.999E-03	6.719E-03	PB-GT
PDZK1	PDZ domain containing 1 [Source:HGNC Symbol;Acc:8821]	chr1	145726918	145764074	182	0.065	1	1	1.999E-03	6.719E-03	PB-GT
ARHGAP42	Rho GTPase activating protein 42 [Source:HGNC Symbol;Acc:26545]	chr11	100558384	100862668	2500	0.867	4	2	2.003E-03	6.722E-03	PB-GT
ASAH1	N-acylsphingosine amidohydrolase (acid ceramidase) 1 [Source:HGNC Symbol;Acc:735]	chr8	17913934	17942494	189	0.065	1	1	2.008E-03	6.732E-03	PB-GT
AK8	adenylate kinase 8 [Source:HGNC Symbol;Acc:26526]	chr9	135600965	135754164	650	0.244	2	1	2.014E-03	6.732E-03	PB-GT
CASP4	caspase 4, apoptosis-related cysteine peptidase [Source:HGNC Symbol;Acc:1505]	chr11	104813593	104840163	187	0.065	1	1	2.016E-03	6.732E-03	PB-GT
ZNF701	zinc finger protein 701 [Source:HGNC Symbol;Acc:25597]	chr19	53059075	53090427	123	0.065	1	1	2.017E-03	6.732E-03	PB-GT
RARRES1	retinoic acid receptor responder (tazarotene induced) 1 [Source:HGNC Symbol;Acc:9867]	chr3	158414681	158450485	211	0.065	1	1	2.018E-03	6.732E-03	PB-GT
C7orf73	chromosome 7 open reading frame 73 [Source:HGNC Symbol;Acc:41909]	chr7	135347244	135378166	175	0.065	1	1	2.022E-03	6.733E-03	PB-GT
SPG7	spastic paraplegia 7 (pure and complicated autosomal recessive) [Source:HGNC Symbol;Acc:11237]	chr16	89557325	89624176	194	0.065	1	1	2.022E-03	6.733E-03	PB-GT
MANBA	mannosidase, beta A, lysosomal [Source:HGNC Symbol;Acc:6831]	chr4	103552660	103682151	864	0.245	2	1	2.033E-03	6.760E-03	PB-GT
ATP1B1	ATPase, Na+/K+ transporting, beta 1 polypeptide [Source:HGNC Symbol;Acc:804]	chr1	169074935	169101960	184	0.065	1	1	2.043E-03	6.774E-03	PB-GT
DRAM2	DNA-damage regulated autophagy modulator 2 [Source:HGNC Symbol;Acc:28769]	chr1	111659955	111682838	184	0.065	1	1	2.043E-03	6.774E-03	PB-GT
DUSP14	dual specificity phosphatase 14 [Source:HGNC Symbol;Acc:17007]	chr17	35849937	35873603	129	0.065	1	1	2.044E-03	6.774E-03	PB-GT
HESX1	HESX homeobox 1 [Source:HGNC Symbol;Acc:4877]	chr3	57231944	57260549	213	0.066	1	1	2.055E-03	6.802E-03	PB-GT
UBE2V1	ubiquitin-conjugating enzyme E2 variant 1 [Source:HGNC Symbol;Acc:12494]	chr20	48697661	48732496	181	0.066	1	1	2.059E-03	6.807E-03	PB-GT
PEX5L-AS2	PEX5L antisense RNA 2 [Source:HGNC Symbol;Acc:41252]	chr3	179616017	179639683	214	0.066	1	1	2.074E-03	6.841E-03	PB-GT
TIMMDC1	translocase of inner mitochondrial membrane domain containing 1 [Source:HGNC Symbol;Acc:1321]	chr3	119217379	119243937	214	0.066	1	1	2.074E-03	6.841E-03	PB-GT
SEC22B	SEC22 vesicle trafficking protein homolog B (S. cerevisiae) (gene/pseudogene) [Source:HGNC Symbol;Acc:10700]	chr1	145096220	145116922	186	0.066	1	1	2.086E-03	6.859E-03	PB-GT
ARPC2	actin related protein 2/3 complex, subunit 2, 34kDa [Source:HGNC Symbol;Acc:705]	chr2	219081817	219119079	231	0.066	1	1	2.088E-03	6.859E-03	PB-GT
EPCAM	epithelial cell adhesion molecule [Source:HGNC Symbol;Acc:11529]	chr2	47572297	47614740	231	0.066	1	1	2.088E-03	6.859E-03	PB-GT
RCN2	reticulocalbin 2, EF-hand calcium binding domain [Source:HGNC Symbol;Acc:9935]	chr15	77223960	77242601	190	0.066	1	1	2.089E-03	6.859E-03	PB-GT
RNASEH2B-AS1	RNASEH2B antisense RNA 1 [Source:HGNC Symbol;Acc:39967]	chr13	51450822	51484848	273	0.066	1	1	2.102E-03	6.893E-03	PB-GT
ATP10D	ATPase, class V, type 10D [Source:HGNC Symbol;Acc:13549]	chr4	47487305	47595503	875	0.248	2	1	2.107E-03	6.902E-03	PB-GT
TUBE1	tubulin, epsilon 1 [Source:HGNC Symbol;Acc:20775]	chr6	112391980	112408732	194	0.067	1	1	2.124E-03	6.939E-03	PB-GT
GPR115	G protein-coupled receptor 115 [Source:HGNC Symbol;Acc:19011]	chr6	47653600	47689757	194	0.067	1	1	2.124E-03	6.939E-03	PB-GT
PALB2	partner and localizer of BRCA2 [Source:HGNC Symbol;Acc:26144]	chr16	23614488	23652631	199	0.067	1	1	2.126E-03	6.939E-03	PB-GT
POLQ	polymerase (DNA directed), theta [Source:HGNC Symbol;Acc:9186]	chr3	121150278	121264853	809	0.249	2	2	2.134E-03	6.958E-03	PB-GT
SLC15A4	solute carrier family 15, member 4 [Source:HGNC Symbol;Acc:23090]	chr12	129277739	129308528	175	0.067	1	1	2.147E-03	6.987E-03	PB-GT
TGFB1	transforming growth factor, beta 1 [Source:HGNC Symbol;Acc:11766]	chr19	41807492	41859816	127	0.067	1	1	2.148E-03	6.987E-03	PB-GT
CLIC4	chloride intracellular channel 4 [Source:HGNC Symbol;Acc:13518]	chr1	25071848	25170815	704	0.250	2	2	2.159E-03	7.017E-03	PB-GT
AADACL2	arylacetamide deacetylase-like 2 [Source:HGNC Symbol;Acc:24427]	chr3	151451704	151479127	219	0.067	1	1	2.170E-03	7.028E-03	PB-GT
SLC16A6	solute carrier family 16, member 6 (monocarboxylic acid transporter 7) [Source:HGNC Symbol;Acc:10927]	chr17	66263167	66287408	133	0.067	1	1	2.170E-03	7.028E-03	PB-GT
ZSWIM7	zinc finger, SWIM-type containing 7 [Source:HGNC Symbol;Acc:26993]	chr17	15879874	15903031	133	0.067	1	1	2.170E-03	7.028E-03	PB-GT
TFB2M	transcription factor B2, mitochondrial [Source:HGNC Symbol;Acc:18559]	chr1	246703862	246729626	190	0.067	1	1	2.175E-03	7.036E-03	PB-GT
SLC33A1	solute carrier family 33 (acetyl-CoA transporter), member 1 [Source:HGNC Symbol;Acc:95]	chr3	155544305	155572218	220	0.068	1	1	2.189E-03	7.074E-03	PB-GT
DNMT3B	DNA (cytosine-5-)-methyltransferase 3 beta [Source:HGNC Symbol;Acc:2979]	chr20	31350191	31397162	187	0.068	1	1	2.194E-03	7.083E-03	PB-GT
CLK4	CDC-like kinase 4 [Source:HGNC Symbol;Acc:13659]	chr5	178029665	178057616	252	0.068	1	1	2.204E-03	7.107E-03	PB-GT
TAF12	TAF12 RNA polymerase II, TATA box binding protein (TBP)-associated factor, 20kDa [Source:HGNC Symbol;Acc:11545]	chr1	28915835	28969597	192	0.068	1	1	2.220E-03	7.142E-03	PB-GT
NCOA4	nuclear receptor coactivator 4 [Source:HGNC Symbol;Acc:7671]	chr10	51565108	51590734	191	0.068	1	1	2.220E-03	7.142E-03	PB-GT
ESD	esterase D [Source:HGNC Symbol;Acc:3465]	chr13	47345391	47371367	282	0.068	1	1	2.240E-03	7.191E-03	PB-GT
CEP250	centrosomal protein 250kDa [Source:HGNC Symbol;Acc:1859]	chr20	34042985	34099804	189	0.068	1	1	2.241E-03	7.191E-03	PB-GT
ADAT2	adenosine deaminase, tRNA-specific 2 [Source:HGNC Symbol;Acc:21172]	chr6	143748126	143771810	200	0.069	1	1	2.255E-03	7.228E-03	PB-GT
SQLE	squalene epoxidase [Source:HGNC Symbol;Acc:11279]	chr8	126010739	126034525	201	0.069	1	1	2.265E-03	7.253E-03	PB-GT
STMND1	stathmin domain containing 1 [Source:HGNC Symbol;Acc:44668]	chr6	17102489	17131603	201	0.069	1	1	2.277E-03	7.283E-03	PB-GT
CLCN4	chloride channel, voltage-sensitive 4 [Source:HGNC Symbol;Acc:2022]	chrX	10125024	10205700	415	0.069	1	1	2.283E-03	7.295E-03	PB-GT
TRAF3IP2-AS1	TRAF3IP2 antisense RNA 1 [Source:HGNC Symbol;Acc:40005]	chr6	111804714	111919505	743	0.255	2	2	2.291E-03	7.312E-03	PB-GT
NUP214	nucleoporin 214kDa [Source:HGNC Symbol;Acc:8064]	chr9	134000948	134110057	681	0.255	2	1	2.296E-03	7.321E-03	PB-GT
DERL1	derlin 1 [Source:HGNC Symbol;Acc:28454]	chr8	124025404	124054663	203	0.070	1	1	2.309E-03	7.354E-03	PB-GT
THAP6	THAP domain containing 6 [Source:HGNC Symbol;Acc:23189]	chr4	76439156	76475683	246	0.070	1	1	2.316E-03	7.359E-03	PB-GT
ILF3	interleukin enhancer binding factor 3, 90kDa [Source:HGNC Symbol;Acc:6038]	chr19	10764937	10803093	132	0.070	1	1	2.316E-03	7.359E-03	PB-GT
OGN	osteoglycin [Source:HGNC Symbol;Acc:8126]	chr9	95146249	95166978	186	0.070	1	1	2.323E-03	7.363E-03	PB-GT
TYW5	tRNA-yW synthesizing protein 5 [Source:HGNC Symbol;Acc:26754]	chr2	200794698	200820459	244	0.070	1	1	2.324E-03	7.363E-03	PB-GT
RAPGEF1	Rap guanine nucleotide exchange factor (GEF) 1 [Source:HGNC Symbol;Acc:4568]	chr9	134452157	134615461	684	0.257	2	2	2.325E-03	7.363E-03	PB-GT
TAOK1	TAO kinase 1 [Source:HGNC Symbol;Acc:29259]	chr17	27717482	27878922	1072	0.543	3	2	2.356E-03	7.453E-03	PB-GT
ZNF84	zinc finger protein 84 [Source:HGNC Symbol;Acc:13159]	chr12	133613878	133639885	184	0.070	1	1	2.368E-03	7.482E-03	PB-GT
HTRA1	HtrA serine peptidase 1 [Source:HGNC Symbol;Acc:9476]	chr10	124221041	124274424	199	0.071	1	1	2.406E-03	7.594E-03	PB-GT
HSDL2	hydroxysteroid dehydrogenase like 2 [Source:HGNC Symbol;Acc:18572]	chr9	115142217	115234690	694	0.260	2	2	2.422E-03	7.636E-03	PB-GT
SHQ1	SHQ1, H/ACA ribonucleoprotein assembly factor [Source:HGNC Symbol;Acc:25543]	chr3	72798428	72911065	848	0.261	2	2	2.436E-03	7.672E-03	PB-GT
TGIF1	TGFB-induced factor homeobox 1 [Source:HGNC Symbol;Acc:11776]	chr18	3411606	3459976	299	0.072	1	1	2.445E-03	7.673E-03	PB-GT
ZNF732	zinc finger protein 732 [Source:HGNC Symbol;Acc:37138]	chr4	264464	299110	253	0.072	1	1	2.446E-03	7.673E-03	PB-GT
LYSMD2	LysM, putative peptidoglycan-binding, domain containing 2 [Source:HGNC Symbol;Acc:28571]	chr15	52015208	52043782	206	0.072	1	1	2.447E-03	7.673E-03	PB-GT
TSTD2	thiosulfate sulfurtransferase (rhodanese)-like domain containing 2 [Source:HGNC Symbol;Acc:30087]	chr9	100362362	100395962	191	0.072	1	1	2.447E-03	7.673E-03	PB-GT
DNAJC21	DnaJ (Hsp40) homolog, subfamily C, member 21 [Source:HGNC Symbol;Acc:27030]	chr5	34929698	34959069	266	0.072	1	1	2.450E-03	7.674E-03	PB-GT
NSD1	nuclear receptor binding SET domain protein 1 [Source:HGNC Symbol;Acc:14234]	chr5	176560026	176727216	971	0.262	2	2	2.458E-03	7.689E-03	PB-GT
AGXT2	alanine--glyoxylate aminotransferase 2 [Source:HGNC Symbol;Acc:14412]	chr5	34998206	35048198	267	0.072	1	1	2.468E-03	7.714E-03	PB-GT
MS4A4A	membrane-spanning 4-domains, subfamily A, member 4A [Source:HGNC Symbol;Acc:13371]	chr11	60048014	60076445	208	0.072	1	1	2.483E-03	7.747E-03	PB-GT
ZNF718	zinc finger protein 718 [Source:HGNC Symbol;Acc:26889]	chr4	124386	157779	255	0.072	1	1	2.484E-03	7.747E-03	PB-GT
CRHR1-IT1	CRHR1 intronic transcript 1 (non-protein coding) [Source:HGNC Symbol;Acc:26327]	chr17	43697694	43725582	143	0.072	1	1	2.500E-03	7.789E-03	PB-GT
CTR9	Ctr9, Paf1/RNA polymerase II complex component, homolog (S. cerevisiae) [Source:HGNC Symbol;Acc:16850]	chr11	10772534	10801290	209	0.073	1	1	2.506E-03	7.799E-03	PB-GT
PHACTR4	phosphatase and actin regulator 4 [Source:HGNC Symbol;Acc:25793]	chr1	28696114	28826881	743	0.264	2	2	2.513E-03	7.809E-03	PB-GT
NLN	neurolysin (metallopeptidase M3 family) [Source:HGNC Symbol;Acc:16058]	chr5	65018023	65167553	979	0.264	2	1	2.515E-03	7.809E-03	PB-GT
PAIP1	poly(A) binding protein interacting protein 1 [Source:HGNC Symbol;Acc:16945]	chr5	43526369	43557860	270	0.073	1	1	2.522E-03	7.816E-03	PB-GT
RBM18	RNA binding motif protein 18 [Source:HGNC Symbol;Acc:28413]	chr9	124999903	125027118	194	0.073	1	1	2.523E-03	7.816E-03	PB-GT
ANXA5	annexin A5 [Source:HGNC Symbol;Acc:543]	chr4	122589110	122618268	258	0.073	1	1	2.541E-03	7.866E-03	PB-GT
RARS	arginyl-tRNA synthetase [Source:HGNC Symbol;Acc:9870]	chr5	167913450	167946304	272	0.073	1	1	2.559E-03	7.912E-03	PB-GT
LRRC48	leucine rich repeat containing 48 [Source:HGNC Symbol;Acc:25384]	chr17	17876127	17920203	145	0.073	1	1	2.569E-03	7.931E-03	PB-GT
CUL4B	cullin 4B [Source:HGNC Symbol;Acc:2555]	chrX	119658464	119709649	441	0.073	1	1	2.571E-03	7.931E-03	PB-GT
RNF168	ring finger protein 168, E3 ubiquitin protein ligase [Source:HGNC Symbol;Acc:26661]	chr3	196195654	196230639	239	0.074	1	1	2.574E-03	7.932E-03	PB-GT
ZNF630	zinc finger protein 630 [Source:HGNC Symbol;Acc:28855]	chrX	47842756	47931025	442	0.074	1	1	2.582E-03	7.949E-03	PB-GT
ONECUT2	one cut homeobox 2 [Source:HGNC Symbol;Acc:8139]	chr18	55102917	55158530	308	0.074	1	1	2.591E-03	7.967E-03	PB-GT
AP3M1	adaptor-related protein complex 3, mu 1 subunit [Source:HGNC Symbol;Acc:569]	chr10	75881524	75910821	207	0.074	1	1	2.598E-03	7.967E-03	PB-GT
SUCO	SUN domain containing ossification factor [Source:HGNC Symbol;Acc:1240]	chr1	172501489	172580971	752	0.267	2	2	2.599E-03	7.967E-03	PB-GT
PPP1CC	protein phosphatase 1, catalytic subunit, gamma isozyme [Source:HGNC Symbol;Acc:9283]	chr12	111157485	111180744	193	0.074	1	1	2.599E-03	7.967E-03	PB-GT
MAGT1	magnesium transporter 1 [Source:HGNC Symbol;Acc:28880]	chrX	77081861	77151090	444	0.074	1	1	2.605E-03	7.977E-03	PB-GT
HMMR	hyaluronan-mediated motility receptor (RHAMM) [Source:HGNC Symbol;Acc:5012]	chr5	162887175	162918947	275	0.074	1	1	2.614E-03	7.996E-03	PB-GT
FAM76A	family with sequence similarity 76, member A [Source:HGNC Symbol;Acc:28530]	chr1	28052490	28089633	209	0.074	1	1	2.620E-03	8.005E-03	PB-GT
CCDC125	coiled-coil domain containing 125 [Source:HGNC Symbol;Acc:28924]	chr5	68576002	68628636	276	0.074	1	1	2.633E-03	8.036E-03	PB-GT
FAM26D	family with sequence similarity 26, member D [Source:HGNC Symbol;Acc:21094]	chr6	116850174	116880031	217	0.075	1	1	2.644E-03	8.061E-03	PB-GT
C10orf88	chromosome 10 open reading frame 88 [Source:HGNC Symbol;Acc:25822]	chr10	124690419	124713919	209	0.075	1	1	2.647E-03	8.063E-03	PB-GT
SYPL1	synaptophysin-like 1 [Source:HGNC Symbol;Acc:11507]	chr7	105730949	105753022	201	0.075	1	1	2.651E-03	8.065E-03	PB-GT
SLC24A5	solute carrier family 24, member 5 [Source:HGNC Symbol;Acc:20611]	chr15	48413169	48434869	215	0.075	1	1	2.660E-03	8.084E-03	PB-GT
PPP2R3C	protein phosphatase 2, regulatory subunit B'', gamma [Source:HGNC Symbol;Acc:17485]	chr14	35554673	35591723	276	0.075	1	1	2.666E-03	8.091E-03	PB-GT
MTIF2	mitochondrial translational initiation factor 2 [Source:HGNC Symbol;Acc:7441]	chr2	55463731	55496483	262	0.075	1	1	2.670E-03	8.091E-03	PB-GT
SUMO1	SMT3 suppressor of mif two 3 homolog 1 (S. cerevisiae) [Source:HGNC Symbol;Acc:12502]	chr2	203070903	203103331	262	0.075	1	1	2.670E-03	8.091E-03	PB-GT
ZNF10	zinc finger protein 10 [Source:HGNC Symbol;Acc:12879]	chr12	133707161	133736051	196	0.075	1	1	2.679E-03	8.107E-03	PB-GT
GAD1	glutamate decarboxylase 1 (brain, 67kDa) [Source:HGNC Symbol;Acc:4092]	chr2	171669723	171717661	263	0.075	1	1	2.690E-03	8.134E-03	PB-GT
LRRC37A16P	leucine rich repeat containing 37, member A16, pseudogene [Source:HGNC Symbol;Acc:43820]	chr17	66121918	66148609	149	0.075	1	1	2.709E-03	8.160E-03	PB-GT
UNC45B	unc-45 homolog B (C. elegans) [Source:HGNC Symbol;Acc:14304]	chr17	33474836	33516364	149	0.075	1	1	2.709E-03	8.160E-03	PB-GT
NT5M	5',3'-nucleotidase, mitochondrial [Source:HGNC Symbol;Acc:15769]	chr17	17206649	17250977	149	0.075	1	1	2.709E-03	8.160E-03	PB-GT
CENPJ	centromere protein J [Source:HGNC Symbol;Acc:17272]	chr13	25457171	25497018	311	0.076	1	1	2.711E-03	8.160E-03	PB-GT
PKNOX1	PBX/knotted 1 homeobox 1 [Source:HGNC Symbol;Acc:9022]	chr21	44394620	44454172	264	0.076	1	1	2.713E-03	8.160E-03	PB-GT
NAA15	N(alpha)-acetyltransferase 15, NatA auxiliary subunit [Source:HGNC Symbol;Acc:30782]	chr4	140222609	140341187	960	0.272	2	1	2.733E-03	8.211E-03	PB-GT
USP45	ubiquitin specific peptidase 45 [Source:HGNC Symbol;Acc:20080]	chr6	99880190	99969604	792	0.272	2	1	2.740E-03	8.224E-03	PB-GT
CEP55	centrosomal protein 55kDa [Source:HGNC Symbol;Acc:1161]	chr10	95256389	95288849	213	0.076	1	1	2.747E-03	8.232E-03	PB-GT
FANCA	Fanconi anemia, complementation group A [Source:HGNC Symbol;Acc:3582]	chr16	89803957	89883065	227	0.076	1	1	2.749E-03	8.232E-03	PB-GT
SENP8	SUMO/sentrin specific peptidase family member 8 [Source:HGNC Symbol;Acc:22992]	chr15	72406599	72433311	219	0.076	1	1	2.757E-03	8.240E-03	PB-GT
GUF1	GUF1 GTPase homolog (S. cerevisiae) [Source:HGNC Symbol;Acc:25799]	chr4	44680444	44702943	269	0.076	1	1	2.757E-03	8.240E-03	PB-GT
NADSYN1	NAD synthetase 1 [Source:HGNC Symbol;Acc:29832]	chr11	71164155	71235153	220	0.076	1	1	2.770E-03	8.269E-03	PB-GT
HADH	hydroxyacyl-CoA dehydrogenase [Source:HGNC Symbol;Acc:4799]	chr4	108910870	108956331	270	0.076	1	1	2.777E-03	8.272E-03	PB-GT
IFT43	intraflagellar transport 43 homolog (Chlamydomonas) [Source:HGNC Symbol;Acc:29669]	chr14	76368479	76550928	1008	0.273	2	2	2.778E-03	8.272E-03	PB-GT
ZNF286B	zinc finger protein 286B [Source:HGNC Symbol;Acc:33241]	chr17	18561742	18585575	151	0.076	1	1	2.780E-03	8.272E-03	PB-GT
TBC1D2	TBC1 domain family, member 2 [Source:HGNC Symbol;Acc:18026]	chr9	100961311	101017915	204	0.077	1	1	2.782E-03	8.272E-03	PB-GT
NUP93	nucleoporin 93kDa [Source:HGNC Symbol;Acc:28958]	chr16	56764017	56880792	819	0.274	2	2	2.806E-03	8.333E-03	PB-GT
SLC18B1	solute carrier family 18, subfamily B, member 1 [Source:HGNC Symbol;Acc:21573]	chr6	133090509	133119701	224	0.077	1	1	2.813E-03	8.345E-03	PB-GT
ZFP90	ZFP90 zinc finger protein [Source:HGNC Symbol;Acc:23329]	chr16	68563993	68609975	230	0.077	1	1	2.820E-03	8.355E-03	PB-GT

table S2. Genes with significant transposon integrations in RKO *BRAF*$^{wt/-/-}$ cells after selection in low glucose medium

Gene ID	Description	Chrom	Start	End	# TTAA sites	Expected	Observed	Libraries	*P*	FDR	Transposon
C4orf45	chromosome 4 open reading frame 45 [Source:HGNC Symbol;Acc:26342]	chr4	159814286	159959912	971	0.275	2	1	2.822E-03	8.355E-03	PB-GT
PTCHD1	patched domain containing 1 [Source:HGNC Symbol;Acc:26392]	chrX	23352133	23422489	463	0.077	1	1	2.827E-03	8.361E-03	PB-GT
GTF2IRD2	GTF2I repeat domain containing 2 [Source:HGNC Symbol;Acc:30775]	chr7	74210483	74267847	208	0.077	1	1	2.834E-03	8.364E-03	PB-GT
GTF2IRD2B	GTF2I repeat domain containing 2B [Source:HGNC Symbol;Acc:33125]	chr7	74508364	74565623	208	0.077	1	1	2.834E-03	8.364E-03	PB-GT
EIF3M	eukaryotic translation initiation factor 3, subunit M [Source:HGNC Symbol;Acc:24460]	chr11	32605344	32627808	223	0.077	1	1	2.844E-03	8.378E-03	PB-GT
FMO2	flavin containing monooxygenase 2 (non-functional) [Source:HGNC Symbol;Acc:3770]	chr1	171154347	171181822	218	0.077	1	1	2.844E-03	8.378E-03	PB-GT
BIVM	basic, immunoglobulin-like variable motif containing [Source:HGNC Symbol;Acc:16034]	chr13	103451399	103493885	319	0.077	1	1	2.849E-03	8.382E-03	PB-GT
FBXO33	F-box protein 33 [Source:HGNC Symbol;Acc:19833]	chr14	39866873	39901704	287	0.078	1	1	2.877E-03	8.456E-03	PB-GT
BCOR	BCL6 corepressor [Source:HGNC Symbol;Acc:20893]	chrX	39909068	40036582	468	0.078	1	1	2.887E-03	8.476E-03	PB-GT
RNF125	ring finger protein 125, E3 ubiquitin protein ligase [Source:HGNC Symbol;Acc:21150]	chr18	29598335	29653176	326	0.078	1	1	2.894E-03	8.477E-03	PB-GT
WDYHV1	WDYHV motif containing 1 [Source:HGNC Symbol;Acc:25490]	chr8	124428965	124479470	228	0.078	1	1	2.896E-03	8.477E-03	PB-GT
IL13RA1	interleukin 13 receptor, alpha 1 [Source:HGNC Symbol;Acc:5974]	chrX	117861535	117928502	469	0.078	1	1	2.899E-03	8.477E-03	PB-GT
CCNB3	cyclin B3 [Source:HGNC Symbol;Acc:18709]	chrX	49967364	50094909	469	0.078	1	1	2.899E-03	8.477E-03	PB-GT
WDR67	WD repeat domain 67 [Source:HGNC Symbol;Acc:30888]	chr8	124054208	124164393	812	0.278	2	2	2.917E-03	8.521E-03	PB-GT
LRRN2	leucine rich repeat neuronal 2 [Source:HGNC Symbol;Acc:16914]	chr1	204586298	204654861	221	0.078	1	1	2.921E-03	8.525E-03	PB-GT
IYD	iodotyrosine deiodinase [Source:HGNC Symbol;Acc:21071]	chr6	150690028	150727105	229	0.079	1	1	2.936E-03	8.561E-03	PB-GT
PLEKHF2	pleckstrin homology domain containing, family F (with FYVE domain) member 2 [Source:HGNC Symbol;Acc:20757]	chr8	96146032	96168912	230	0.079	1	1	2.946E-03	8.573E-03	PB-GT
EIF3L	eukaryotic translation initiation factor 3, subunit L [Source:HGNC Symbol;Acc:18138]	chr22	38244875	38285414	158	0.079	1	1	2.956E-03	8.573E-03	PB-GT
TUBGCP4	tubulin, gamma complex associated protein 4 [Source:HGNC Symbol;Acc:16691]	chr15	43661419	43699293	227	0.079	1	1	2.957E-03	8.573E-03	PB-GT
SCAMP5	secretory carrier membrane protein 5 [Source:HGNC Symbol;Acc:30386]	chr15	75249560	75313837	227	0.079	1	1	2.957E-03	8.573E-03	PB-GT
TTC33	tetratricopeptide repeat domain 33 [Source:HGNC Symbol;Acc:29959]	chr5	40714577	40756077	293	0.079	1	1	2.958E-03	8.573E-03	PB-GT
UBOX5	U-box domain containing 5 [Source:HGNC Symbol;Acc:17777]	chr20	3088219	3140842	218	0.079	1	1	2.960E-03	8.573E-03	PB-GT
SLC35B3	solute carrier family 35, member B3 [Source:HGNC Symbol;Acc:21601]	chr6	8413301	8435794	230	0.079	1	1	2.961E-03	8.573E-03	PB-GT
FGFR1	fibroblast growth factor receptor 1 [Source:HGNC Symbol;Acc:3688]	chr8	38268656	38326352	231	0.079	1	1	2.971E-03	8.580E-03	PB-GT
ZBTB43	zinc finger and BTB domain containing 43 [Source:HGNC Symbol;Acc:17908]	chr9	129567285	129600489	211	0.079	1	1	2.971E-03	8.580E-03	PB-GT
C1orf132	chromosome 1 open reading frame 132 [Source:HGNC Symbol;Acc:32018]	chr1	207986905	208042495	223	0.079	1	1	2.973E-03	8.580E-03	PB-GT
RBM17	RNA binding motif protein 17 [Source:HGNC Symbol;Acc:16944]	chr10	6130950	6159420	222	0.079	1	1	2.978E-03	8.585E-03	PB-GT
NT5C1B-RDH14	NT5C1B-RDH14 readthrough [Source:HGNC Symbol;Acc:38831]	chr2	18736811	18770830	278	0.079	1	1	2.998E-03	8.620E-03	PB-GT
NT5C1B	5'-nucleotidase, cytosolic IB [Source:HGNC Symbol;Acc:17818]	chr2	18737050	18770838	278	0.079	1	1	2.998E-03	8.620E-03	PB-GT
TMEM206	transmembrane protein 206 [Source:HGNC Symbol;Acc:25593]	chr1	212537273	212588243	224	0.080	1	1	2.999E-03	8.620E-03	PB-GT
NDUFC1	NADH dehydrogenase (ubiquinone) 1, subcomplex unknown, 1, 6kDa [Source:HGNC Symbol;Acc:7705]	chr4	140188034	140223705	281	0.080	1	1	3.002E-03	8.620E-03	PB-GT
PRB1	proline-rich protein BstNI subfamily 1 [Source:HGNC Symbol;Acc:9337]	chr12	11504757	11548500	208	0.080	1	1	3.007E-03	8.628E-03	PB-GT
ACTR10	actin-related protein 10 homolog (S. cerevisiae) [Source:HGNC Symbol;Acc:17372]	chr14	58666798	58701750	294	0.080	1	1	3.015E-03	8.640E-03	PB-GT
FTSJD2	FtsJ methyltransferase domain containing 2 [Source:HGNC Symbol;Acc:21077]	chr6	37400995	37450603	233	0.080	1	1	3.037E-03	8.695E-03	PB-GT
LINC00658	long intergenic non-protein coding RNA 658 [Source:HGNC Symbol;Acc:44315]	chr20	5407541	5451740	221	0.080	1	1	3.040E-03	8.695E-03	PB-GT
TRIM14	tripartite motif containing 14 [Source:HGNC Symbol;Acc:16283]	chr9	100831557	100881494	214	0.080	1	1	3.054E-03	8.727E-03	PB-GT
NOP58	NOP58 ribonucleoprotein [Source:HGNC Symbol;Acc:29926]	chr2	203130439	203168389	281	0.080	1	1	3.061E-03	8.737E-03	PB-GT
STAMBP	STAM binding protein [Source:HGNC Symbol;Acc:16950]	chr2	74056086	74100786	282	0.081	1	1	3.082E-03	8.789E-03	PB-GT
ZNF808	zinc finger protein 808 [Source:HGNC Symbol;Acc:33230]	chr19	53030905	53067717	153	0.081	1	1	3.089E-03	8.799E-03	PB-GT
RDH10	retinol dehydrogenase 10 (all-trans) [Source:HGNC Symbol;Acc:19975]	chr8	74206847	74237516	236	0.081	1	1	3.098E-03	8.816E-03	PB-GT
FNIP2	folliculin interacting protein 2 [Source:HGNC Symbol;Acc:29280]	chr4	159690290	159829201	1006	0.285	2	1	3.115E-03	8.857E-03	PB-GT
AHCTF1	AT hook containing transcription factor 1 [Source:HGNC Symbol;Acc:24618]	chr1	247002400	247095280	805	0.286	2	1	3.144E-03	8.930E-03	PB-GT
B4GALNT2	beta-1,4-N-acetyl-galactosaminyl transferase 2 [Source:HGNC Symbol;Acc:24136]	chr17	47209822	47247351	161	0.082	1	1	3.150E-03	8.938E-03	PB-GT
TLL1	tolloid-like 1 [Source:HGNC Symbol;Acc:11843]	chr4	166794410	167025047	2086	0.591	3	2	3.176E-03	9.000E-03	PB-GT
CDK7	cyclin-dependent kinase 7 [Source:HGNC Symbol;Acc:1778]	chr5	68530668	68573250	304	0.082	1	1	3.178E-03	9.000E-03	PB-GT
MB21D1	Mab-21 domain containing 1 [Source:HGNC Symbol;Acc:21367]	chr6	74123238	74161999	239	0.082	1	1	3.191E-03	9.023E-03	PB-GT
TMEM50B	transmembrane protein 50B [Source:HGNC Symbol;Acc:1280]	chr21	34804792	34852318	287	0.082	1	1	3.193E-03	9.023E-03	PB-GT
SYS1-DBNDD2	SYS1-DBNDD2 readthrough (non-protein coding) [Source:HGNC Symbol;Acc:33535]	chr20	43991840	44039250	227	0.082	1	1	3.203E-03	9.043E-03	PB-GT
NDUFS4	NADH dehydrogenase (ubiquinone) Fe-S protein 4, 18kDa (NADH-coenzyme Q reductase) [Source:HGNC Symbol;Acc:7711]	chr5	52856463	52979168	1070	0.288	2	1	3.224E-03	9.093E-03	PB-GT
MINK1	misshapen-like kinase 1 [Source:HGNC Symbol;Acc:17565]	chr17	4736683	4801356	163	0.083	1	1	3.227E-03	9.093E-03	PB-GT
DNMT3A	DNA (cytosine-5-)-methyltransferase 3 alpha [Source:HGNC Symbol;Acc:2978]	chr2	25455845	25565459	289	0.083	1	1	3.233E-03	9.100E-03	PB-GT
PTPRG-AS1	PTPRG antisense RNA 1 [Source:HGNC Symbol;Acc:44638]	chr3	62247498	62355005	939	0.289	2	1	3.240E-03	9.108E-03	PB-GT
BVES-AS1	BVES antisense RNA 1 [Source:HGNC Symbol;Acc:21223]	chr6	105584183	105617820	241	0.083	1	1	3.243E-03	9.108E-03	PB-GT
ZFX	zinc finger protein, X-linked [Source:HGNC Symbol;Acc:12869]	chrX	24167290	24234372	497	0.083	1	1	3.245E-03	9.108E-03	PB-GT
C5orf56	chromosome 5 open reading frame 56 [Source:HGNC Symbol;Acc:33838]	chr5	131746328	131811736	308	0.083	1	1	3.260E-03	9.142E-03	PB-GT
MROH5	maestro heat-like repeat family member 5 [Source:HGNC Symbol;Acc:42976]	chr8	142443929	142517330	243	0.083	1	1	3.279E-03	9.186E-03	PB-GT
C10orf129	chromosome 10 open reading frame 129 [Source:HGNC Symbol;Acc:31665]	chr10	96953957	96988685	234	0.084	1	1	3.299E-03	9.232E-03	PB-GT
TMEM99	transmembrane protein 99 [Source:HGNC Symbol;Acc:28305]	chr17	38975358	38992522	165	0.084	1	1	3.304E-03	9.238E-03	PB-GT
PSMD1	proteasome (prosome, macropain) 26S subunit, non-ATPase, 1 [Source:HGNC Symbol;Acc:9554]	chr2	231921578	232037541	1018	0.291	2	1	3.311E-03	9.248E-03	PB-GT
BCL2L13	BCL2-like 13 (apoptosis facilitator) [Source:HGNC Symbol;Acc:17164]	chr22	18111621	18213388	583	0.291	2	1	3.316E-03	9.252E-03	PB-GT
ERLEC1	endoplasmic reticulum lectin 1 [Source:HGNC Symbol;Acc:25222]	chr2	54014181	54045956	294	0.084	1	1	3.342E-03	9.311E-03	PB-GT
SRP72	signal recognition particle 72kDa [Source:HGNC Symbol;Acc:11303]	chr4	57333081	57369839	297	0.084	1	1	3.343E-03	9.311E-03	PB-GT
OBFC1	oligonucleotide/oligosaccharide-binding fold containing 1 [Source:HGNC Symbol;Acc:26200]	chr10	105642300	105677963	236	0.084	1	1	3.354E-03	9.332E-03	PB-GT
TCEB1	transcription elongation factor B (SIII), polypeptide 1 (15kDa, elongin C) [Source:HGNC Symbol;Acc:11617]	chr8	74851404	74884522	246	0.084	1	1	3.358E-03	9.334E-03	PB-GT
MKKS	McKusick-Kaufman syndrome [Source:HGNC Symbol;Acc:7108]	chr20	10385832	10414870	233	0.084	1	1	3.369E-03	9.357E-03	PB-GT
TJP2	tight junction protein 2 [Source:HGNC Symbol;Acc:11828]	chr9	71736224	71870124	784	0.294	2	1	3.405E-03	9.431E-03	PB-GT
PRPF39	PRP39 pre-mRNA processing factor 39 homolog (S. cerevisiae) [Source:HGNC Symbol;Acc:20314]	chr14	45553302	45585485	313	0.085	1	1	3.405E-03	9.431E-03	PB-GT
C3orf83	chromosome 3 open reading frame 83 [Source:HGNC Symbol;Acc:40375]	chr3	12556433	12602558	276	0.085	1	1	3.407E-03	9.431E-03	PB-GT
SART3	squamous cell carcinoma antigen recognized by T cells 3 [Source:HGNC Symbol;Acc:16860]	chr12	108916357	108955176	222	0.085	1	1	3.414E-03	9.431E-03	PB-GT
RBM4	RNA binding motif protein 4 [Source:HGNC Symbol;Acc:9901]	chr11	66384097	66434153	245	0.085	1	1	3.415E-03	9.431E-03	PB-GT
ELP2	elongator acetyltransferase complex subunit 2 [Source:HGNC Symbol;Acc:18248]	chr18	33709407	33757909	355	0.085	1	1	3.416E-03	9.431E-03	PB-GT
C3orf33	chromosome 3 open reading frame 33 [Source:HGNC Symbol;Acc:26434]	chr3	155480401	155524140	277	0.085	1	1	3.431E-03	9.462E-03	PB-GT
CRBN	cereblon [Source:HGNC Symbol;Acc:30185]	chr3	3190676	3221394	278	0.086	1	1	3.455E-03	9.520E-03	PB-GT
GAREM	GRB2 associated, regulator of MAPK1 [Source:HGNC Symbol;Acc:26136]	chr18	29704840	30050447	2536	0.607	3	2	3.506E-03	9.652E-03	PB-GT
PRIM2	primase, DNA, polypeptide 2 (58kDa) [Source:HGNC Symbol;Acc:9370]	chr6	57179603	57513375	2886	0.991	4	2	3.529E-03	9.706E-03	PB-GT
ADHFE1	alcohol dehydrogenase, iron containing, 1 [Source:HGNC Symbol;Acc:16354]	chr8	67342420	67383836	253	0.087	1	1	3.546E-03	9.744E-03	PB-GT
LEMD3	LEM domain containing 3 [Source:HGNC Symbol;Acc:28887]	chr12	65563351	65642107	781	0.299	2	1	3.567E-03	9.791E-03	PB-GT
PMP22	peripheral myelin protein 22 [Source:HGNC Symbol;Acc:9118]	chr17	15133095	15168643	172	0.087	1	1	3.582E-03	9.823E-03	PB-GT
PRRC1	proline-rich coiled-coil 1 [Source:HGNC Symbol;Acc:28164]	chr5	126853301	126890781	324	0.087	1	1	3.597E-03	9.849E-03	PB-GT
SLC25A33	solute carrier family 25 (pyrimidine nucleotide carrier), member 33 [Source:HGNC Symbol;Acc:29681]	chr1	9599541	9642831	246	0.087	1	1	3.598E-03	9.849E-03	PB-GT
HIPK3	homeodomain interacting protein kinase 3 [Source:HGNC Symbol;Acc:4915]	chr11	33278218	33378569	865	0.300	2	2	3.605E-03	9.857E-03	PB-GT
HAVCR2	hepatitis A virus cellular receptor 2 [Source:HGNC Symbol;Acc:18437]	chr5	156512843	156569880	325	0.088	1	1	3.619E-03	9.886E-03	PB-GT
LRP8	low density lipoprotein receptor-related protein 8, apolipoprotein e receptor [Source:HGNC Symbol;Acc:6700]	chr1	53711217	53793742	247	0.088	1	1	3.627E-03	9.898E-03	PB-GT
IST1	increased sodium tolerance 1 homolog (yeast) [Source:HGNC Symbol;Acc:28977]	chr16	71919136	71962913	263	0.088	1	1	3.660E-03	9.972E-03	PB-GT
DIP2B	DIP2 disco-interacting protein 2 homolog B (Drosophila) [Source:HGNC Symbol;Acc:29284]	chr12	50898768	51142450	1606	0.615	3	2	3.661E-03	9.972E-03	PB-GT
LYPD6	LY6/PLAUR domain containing 6 [Source:HGNC Symbol;Acc:28751]	chr2	150186499	150330662	1058	0.303	2	2	3.685E-03	1.003E-02	PB-GT
SLC9B1	solute carrier family 9, subfamily B (NHA1, cation proton antiporter 1), member 1 [Source:HGNC Symbol;Acc:24244]	chr4	103806205	103940896	1069	0.303	2	1	3.689E-03	1.003E-02	PB-GT
RNF111	ring finger protein 111 [Source:HGNC Symbol;Acc:17384]	chr15	59157374	59389618	1779	0.619	3	1	3.741E-03	1.016E-02	PB-GT
ARPC1A	actin related protein 2/3 complex, subunit 1A, 41kDa [Source:HGNC Symbol;Acc:703]	chr7	98923521	98985787	240	0.089	1	1	3.743E-03	1.016E-02	PB-GT
TEX14	testis expressed 14 [Source:HGNC Symbol;Acc:11737]	chr17	56634039	56769416	601	0.304	2	2	3.749E-03	1.016E-02	PB-GT
UHRF2	ubiquitin-like with PHD and ring finger domains 2, E3 ubiquitin protein ligase [Source:HGNC Symbol;Acc:12557]	chr9	6413151	6507054	812	0.305	2	1	3.754E-03	1.017E-02	PB-GT
STX11	syntaxin 11 [Source:HGNC Symbol;Acc:11429]	chr6	144471663	144509507	260	0.089	1	1	3.759E-03	1.017E-02	PB-GT
SYT6	synaptotagmin VI [Source:HGNC Symbol;Acc:18638]	chr1	114631914	114696541	252	0.089	1	1	3.771E-03	1.019E-02	PB-GT
DNAJC2	DnaJ (Hsp40) homolog, subfamily C, member 2 [Source:HGNC Symbol;Acc:13192]	chr7	102952921	102985320	241	0.089	1	1	3.773E-03	1.019E-02	PB-GT
LINC00649	long intergenic non-protein coding RNA 649 [Source:HGNC Symbol;Acc:44305]	chr21	35287838	35349992	313	0.090	1	1	3.779E-03	1.019E-02	PB-GT
SEC61A2	Sec61 alpha 2 subunit (S. cerevisiae) [Source:HGNC Symbol;Acc:17702]	chr10	12171636	12211960	251	0.090	1	1	3.780E-03	1.019E-02	PB-GT
DLAT	dihydrolipoamide S-acetyltransferase [Source:HGNC Symbol;Acc:2896]	chr11	111895538	111935114	260	0.090	1	1	3.833E-03	1.032E-02	PB-GT
ANKRD32	ankyrin repeat domain 32 [Source:HGNC Symbol;Acc:25408]	chr5	93954052	94075141	1139	0.307	2	2	3.836E-03	1.032E-02	PB-GT
PDE6D	phosphodiesterase 6D, cGMP-specific, rod, delta [Source:HGNC Symbol;Acc:8788]	chr2	232597135	232650982	317	0.091	1	1	3.869E-03	1.040E-02	PB-GT
SEMA3A	sema domain, immunoglobulin domain (Ig), short basic domain, secreted, (semaphorin) 3A [Source:HGNC Symbol;Acc:10723]	chr7	83587659	84122040	5229	1.942	6	2	3.873E-03	1.040E-02	PB-GT
DSG2	desmoglein 2 [Source:HGNC Symbol;Acc:3049]	chr18	29078006	29128971	379	0.091	1	1	3.879E-03	1.041E-02	PB-GT
MED17	mediator complex subunit 17 [Source:HGNC Symbol;Acc:2375]	chr11	93517393	93547861	262	0.091	1	1	3.891E-03	1.043E-02	PB-GT
STRN	striatin, calmodulin binding protein [Source:HGNC Symbol;Acc:11424]	chr2	37070783	37193615	1080	0.309	2	2	3.902E-03	1.045E-02	PB-GT
SNX9	sorting nexin 9 [Source:HGNC Symbol;Acc:14973]	chr6	158244296	158366109	901	0.309	2	2	3.925E-03	1.050E-02	PB-GT
CCDC111	coiled-coil domain containing 111 [Source:HGNC Symbol;Acc:26575]	chr4	185570767	185616117	323	0.091	1	1	3.935E-03	1.052E-02	PB-GT
SAV1	salvador homolog 1 (Drosophila) [Source:HGNC Symbol;Acc:17795]	chr14	51098776	51135049	338	0.092	1	1	3.953E-03	1.054E-02	PB-GT
EXD2	exonuclease 3'-5' domain containing 2 [Source:HGNC Symbol;Acc:20217]	chr14	69658228	69709075	338	0.092	1	1	3.953E-03	1.054E-02	PB-GT
USP22	ubiquitin specific peptidase 22 [Source:HGNC Symbol;Acc:12621]	chr17	20902910	20947073	181	0.092	1	1	3.955E-03	1.054E-02	PB-GT
CCDC63	coiled-coil domain containing 63 [Source:HGNC Symbol;Acc:26669]	chr12	111284573	111345339	241	0.092	1	1	4.004E-03	1.066E-02	PB-GT
EPB41L2	erythrocyte membrane protein band 4.1-like 2 [Source:HGNC Symbol;Acc:3379]	chr6	131160487	131384462	1837	0.631	3	1	4.010E-03	1.067E-02	PB-GT
CCP110	centriolar coiled coil protein 110kDa [Source:HGNC Symbol;Acc:24342]	chr16	19535133	19564730	276	0.092	1	1	4.020E-03	1.069E-02	PB-GT
CCT3	chaperonin containing TCP1, subunit 3 (gamma) [Source:HGNC Symbol;Acc:1616]	chr1	156278759	156337664	261	0.093	1	1	4.036E-03	1.072E-02	PB-GT
UBE2E3	ubiquitin-conjugating enzyme E2E 3 [Source:HGNC Symbol;Acc:12479]	chr2	181831975	181941312	1096	0.313	2	2	4.064E-03	1.078E-02	PB-GT
TIMM23	translocase of inner mitochondrial membrane 23 homolog (yeast) [Source:HGNC Symbol;Acc:17312]	chr10	51592080	51623365	261	0.093	1	1	4.078E-03	1.081E-02	PB-GT
C17orf64	chromosome 17 open reading frame 64 [Source:HGNC Symbol;Acc:26990]	chr17	58469790	58508782	184	0.093	1	1	4.083E-03	1.081E-02	PB-GT
LAMTOR5-AS1	LAMTOR5 antisense RNA 1 [Source:HGNC Symbol;Acc:40823]	chr1	110925441	110974982	263	0.093	1	1	4.096E-03	1.084E-02	PB-GT
KIN	KIN, antigenic determinant of recA protein homolog (mouse) [Source:HGNC Symbol;Acc:6327]	chr10	7795188	7829990	262	0.094	1	1	4.108E-03	1.086E-02	PB-GT
FAM194B	family with sequence similarity 194, member B [Source:HGNC Symbol;Acc:26523]	chr13	46108657	46189874	387	0.094	1	1	4.147E-03	1.095E-02	PB-GT
S100PBP	S100P binding protein [Source:HGNC Symbol;Acc:25768]	chr1	33282368	33324476	265	0.094	1	1	4.157E-03	1.097E-02	PB-GT
SUDS3	suppressor of defective silencing 3 homolog (S. cerevisiae) [Source:HGNC Symbol;Acc:29545]	chr12	118814185	118855840	246	0.094	1	1	4.166E-03	1.098E-02	PB-GT
HNRNPUL1	heterogeneous nuclear ribonucleoprotein U-like 1 [Source:HGNC Symbol;Acc:17011]	chr19	41768401	41813503	179	0.094	1	1	4.190E-03	1.103E-02	PB-GT
NDUFAF5	NADH dehydrogenase (ubiquinone) complex I, assembly factor 5 [Source:HGNC Symbol;Acc:15899]	chr20	13765596	13799067	261	0.095	1	1	4.200E-03	1.105E-02	PB-GT
PBX4	pre-B-cell leukemia homeobox 4 [Source:HGNC Symbol;Acc:13403]	chr19	19672516	19729725	180	0.095	1	1	4.235E-03	1.113E-02	PB-GT
PON2	paraoxonase 2 [Source:HGNC Symbol;Acc:9205]	chr7	95034175	95064510	256	0.095	1	1	4.242E-03	1.113E-02	PB-GT
PPHLN1	periphilin 1 [Source:HGNC Symbol;Acc:19369]	chr12	42632249	42853517	1675	0.641	3	1	4.243E-03	1.113E-02	PB-GT
TMEM51	transmembrane protein 51 [Source:HGNC Symbol;Acc:25488]	chr1	15479028	15546976	268	0.095	1	1	4.249E-03	1.114E-02	PB-GT
POP1	processing of precursor 1, ribonuclease P/MRP subunit (S. cerevisiae) [Source:HGNC Symbol;Acc:30129]	chr8	99129525	99172062	278	0.095	1	1	4.258E-03	1.115E-02	PB-GT
HABP4	hyaluronan binding protein 4 [Source:HGNC Symbol;Acc:17062]	chr9	99212483	99253618	254	0.095	1	1	4.260E-03	1.115E-02	PB-GT
DNAL1	dynein, axonemal, light chain 1 [Source:HGNC Symbol;Acc:23247]	chr14	74111578	74164463	352	0.095	1	1	4.277E-03	1.118E-02	PB-GT
TMX3	thioredoxin-related transmembrane protein 3 [Source:HGNC Symbol;Acc:24718]	chr18	66340925	66382535	399	0.096	1	1	4.285E-03	1.119E-02	PB-GT
HEATR4	HEAT repeat containing 4 [Source:HGNC Symbol;Acc:16761]	chr14	73945189	74025651	353	0.096	1	1	4.300E-03	1.121E-02	PB-GT
SLFN5	schlafen family member 5 [Source:HGNC Symbol;Acc:28286]	chr17	33570055	33600674	189	0.096	1	1	4.301E-03	1.121E-02	PB-GT
SMCHD1	structural maintenance of chromosomes flexible hinge domain containing 1 [Source:HGNC Symbol;Acc:29090]	chr18	2655737	2805015	1336	0.320	2	2	4.304E-03	1.121E-02	PB-GT
ZNF398	zinc finger protein 398 [Source:HGNC Symbol;Acc:18373]	chr7	148823508	148880116	258	0.096	1	1	4.306E-03	1.121E-02	PB-GT
KIAA2018	KIAA2018 [Source:HGNC Symbol;Acc:30494]	chr3	113367232	113415493	312	0.096	1	1	4.321E-03	1.124E-02	PB-GT
RAD17	RAD17 homolog (S. pombe) [Source:HGNC Symbol;Acc:9807]	chr5	68665120	68710628	358	0.096	1	1	4.365E-03	1.134E-02	PB-GT
RAB5A	RAB5A, member RAS oncogene family [Source:HGNC Symbol;Acc:9783]	chr3	19988571	20026667	314	0.097	1	1	4.375E-03	1.136E-02	PB-GT
ATPAF2	ATP synthase mitochondrial F1 complex assembly factor 2 [Source:HGNC Symbol;Acc:18802]	chr17	17880723	17942523	191	0.097	1	1	4.389E-03	1.138E-02	PB-GT

table S2. Genes with significant transposon integrations in RKO *BRAF*$^{wt/-/-}$ cells after selection in low glucose medium

Gene ID	Description	Chrom	Start	End	# TTAA sites	Expected	Observed	Libraries	*P*	FDR	Transposon
PHF20	PHD finger protein 20 [Source:HGNC Symbol;Acc:16098]	chr20	34359896	34538303	890	0.322	2	2	4.397E-03	1.139E-02	PB-GT
RNF32	ring finger protein 32 [Source:HGNC Symbol;Acc:17118]	chr7	156432975	156469824	261	0.097	1	1	4.404E-03	1.140E-02	PB-GT
ZNF833P	zinc finger protein 833, pseudogene [Source:HGNC Symbol;Acc:33819]	chr19	11750591	11797382	184	0.097	1	1	4.419E-03	1.143E-02	PB-GT
WTAP	Wilms tumor 1 associated protein [Source:HGNC Symbol;Acc:16846]	chr6	160146617	160177351	283	0.097	1	1	4.430E-03	1.145E-02	PB-GT
RNF11	ring finger protein 11 [Source:HGNC Symbol;Acc:10056]	chr1	51701943	51739127	274	0.097	1	1	4.435E-03	1.145E-02	PB-GT
BNIP2	BCL2/adenovirus E1B 19kDa interacting protein 2 [Source:HGNC Symbol;Acc:1083]	chr15	59951345	59981733	280	0.097	1	1	4.444E-03	1.146E-02	PB-GT
ABCE1	ATP-binding cassette, sub-family E (OABP), member 1 [Source:HGNC Symbol;Acc:69]	chr4	146019084	146050331	344	0.097	1	1	4.446E-03	1.146E-02	PB-GT
LITAF	lipopolysaccharide-induced TNF factor [Source:HGNC Symbol;Acc:16841]	chr16	11641853	11730237	291	0.097	1	1	4.454E-03	1.147E-02	PB-GT
SELP	selectin P (granule membrane protein 140kDa, antigen CD62) [Source:HGNC Symbol;Acc:10721]	chr1	169558087	169599431	276	0.098	1	1	4.497E-03	1.157E-02	PB-GT
ZNF724P	zinc finger protein 724, pseudogene [Source:HGNC Symbol;Acc:32460]	chr19	23404401	23433192	186	0.098	1	1	4.513E-03	1.159E-02	PB-GT
SF3B3	splicing factor 3b, subunit 3, 130kDa [Source:HGNC Symbol;Acc:10770]	chr16	70557691	70608820	293	0.098	1	1	4.513E-03	1.159E-02	PB-GT
DIS3L2	DIS3 mitotic control homolog (S. cerevisiae)-like 2 [Source:HGNC Symbol;Acc:28648]	chr2	232825955	233209060	2283	0.653	3	1	4.517E-03	1.159E-02	PB-GT
ANKRD36BP2	ankyrin repeat domain 36B pseudogene 2 [Source:HGNC Symbol;Acc:33607]	chr2	89065324	89106126	344	0.098	1	1	4.533E-03	1.161E-02	PB-GT
PARP16	poly (ADP-ribose) polymerase family, member 16 [Source:HGNC Symbol;Acc:26040]	chr15	65526798	65592956	283	0.098	1	1	4.537E-03	1.161E-02	PB-GT
IFT122	intraflagellar transport 122 homolog (Chlamydomonas) [Source:HGNC Symbol;Acc:13556]	chr3	129158968	129239198	320	0.098	1	1	4.539E-03	1.161E-02	PB-GT
KCTD9	potassium channel tetramerisation domain containing 9 [Source:HGNC Symbol;Acc:22401]	chr8	25285366	25315992	288	0.099	1	1	4.559E-03	1.165E-02	PB-GT
MMD	monocyte to macrophage differentiation-associated [Source:HGNC Symbol;Acc:7153]	chr17	53469974	53499353	195	0.099	1	1	4.569E-03	1.167E-02	PB-GT
APC	adenomatous polyposis coli [Source:HGNC Symbol;Acc:583]	chr5	112043195	112181936	1214	0.327	2	1	4.576E-03	1.168E-02	PB-GT
CDK8	cyclin-dependent kinase 8 [Source:HGNC Symbol;Acc:1779]	chr13	26828276	26979375	1349	0.328	2	2	4.590E-03	1.170E-02	PB-GT
PARD6G	par-6 partitioning defective 6 homolog gamma (C. elegans) [Source:HGNC Symbol;Acc:16076]	chr18	77915115	78005429	415	0.099	1	1	4.624E-03	1.178E-02	PB-GT
MTO1	mitochondrial translation optimization 1 homolog (S. cerevisiae) [Source:HGNC Symbol;Acc:19261]	chr6	74171301	74218959	290	0.100	1	1	4.644E-03	1.181E-02	PB-GT
SDAD1	SDA1 domain containing 1 [Source:HGNC Symbol;Acc:25537]	chr4	76862103	76912115	352	0.100	1	1	4.648E-03	1.181E-02	PB-GT
COPB2	coatomer protein complex, subunit beta 2 (beta prime) [Source:HGNC Symbol;Acc:2232]	chr3	139074442	139108574	324	0.100	1	1	4.649E-03	1.181E-02	PB-GT
PPIL6	peptidylprolyl isomerase (cyclophilin)-like 6 [Source:HGNC Symbol;Acc:21557]	chr6	109711419	109762374	291	0.100	1	1	4.675E-03	1.186E-02	PB-GT
NKIRAS1	NFKB inhibitor interacting Ras-like 1 [Source:HGNC Symbol;Acc:17899]	chr3	23933151	23988082	325	0.100	1	1	4.677E-03	1.186E-02	PB-GT
LYST	lysosomal trafficking regulator [Source:HGNC Symbol;Acc:1968]	chr1	235824341	236046940	1862	0.661	3	1	4.716E-03	1.195E-02	PB-GT
SKIL	SKI-like oncogene [Source:HGNC Symbol;Acc:10897]	chr3	170075466	170114623	327	0.101	1	1	4.733E-03	1.197E-02	PB-GT
PCF11	PCF11, cleavage and polyadenylation factor subunit, homolog (S. cerevisiae) [Source:HGNC Symbol;Acc:30097]	chr11	82868030	82898493	290	0.101	1	1	4.736E-03	1.197E-02	PB-GT
MYH15	myosin, heavy chain 15 [Source:HGNC Symbol;Acc:31073]	chr3	108099216	108248169	1077	0.331	2	2	4.737E-03	1.197E-02	PB-GT
FBXW2	F-box and WD repeat domain containing 2 [Source:HGNC Symbol;Acc:13608]	chr9	123514256	123555690	269	0.101	1	1	4.761E-03	1.202E-02	PB-GT
ZNF727	zinc finger protein 727 [Source:HGNC Symbol;Acc:22785]	chr7	63505821	63538927	272	0.101	1	1	4.770E-03	1.203E-02	PB-GT
TRAPPC13	trafficking protein particle complex 13 [Source:HGNC Symbol;Acc:25828]	chr5	64920543	64962060	375	0.101	1	1	4.775E-03	1.203E-02	PB-GT
MED1	mediator complex subunit 1 [Source:HGNC Symbol;Acc:9234]	chr17	37560538	37607539	200	0.101	1	1	4.798E-03	1.208E-02	PB-GT
ACBD6	acyl-CoA binding domain containing 6 [Source:HGNC Symbol;Acc:23339]	chr1	180244515	180472089	1872	0.665	3	2	4.805E-03	1.208E-02	PB-GT
MTFR1	mitochondrial fission regulator 1 [Source:HGNC Symbol;Acc:29510]	chr8	66556969	66683496	972	0.333	2	2	4.805E-03	1.208E-02	PB-GT
DSP	desmoplakin [Source:HGNC Symbol;Acc:3052]	chr6	7541808	7586950	296	0.102	1	1	4.832E-03	1.213E-02	PB-GT
SMC5	structural maintenance of chromosomes 5 [Source:HGNC Symbol;Acc:20465]	chr9	72873937	72969804	890	0.334	2	1	4.837E-03	1.213E-02	PB-GT
DAGLB	diacylglycerol lipase, beta [Source:HGNC Symbol;Acc:28923]	chr7	6448757	6523821	274	0.102	1	1	4.838E-03	1.213E-02	PB-GT
ACTN1	actinin, alpha 1 [Source:HGNC Symbol;Acc:163]	chr14	69340860	69446157	376	0.102	1	1	4.859E-03	1.217E-02	PB-GT
HPS5	Hermansky-Pudlak syndrome 5 [Source:HGNC Symbol;Acc:17022]	chr11	18300223	18343745	294	0.102	1	1	4.863E-03	1.217E-02	PB-GT
SRPRB	signal recognition particle receptor, B subunit [Source:HGNC Symbol;Acc:24085]	chr3	133502877	133544616	332	0.102	1	1	4.873E-03	1.219E-02	PB-GT
RALBP1	ralA binding protein 1 [Source:HGNC Symbol;Acc:9841]	chr18	9475007	9538114	427	0.102	1	1	4.886E-03	1.221E-02	PB-GT
LRP5	low density lipoprotein receptor-related protein 5 [Source:HGNC Symbol;Acc:6697]	chr11	68080077	68216743	295	0.102	1	1	4.895E-03	1.221E-02	PB-GT
LMNB1	lamin B1 [Source:HGNC Symbol;Acc:6637]	chr5	126112315	126172712	380	0.102	1	1	4.899E-03	1.221E-02	PB-GT
FAF2	Fas associated factor family member 2 [Source:HGNC Symbol;Acc:24666]	chr5	175874629	175937075	380	0.102	1	1	4.899E-03	1.221E-02	PB-GT
SEC11A	SEC11 homolog A (S. cerevisiae) [Source:HGNC Symbol;Acc:17718]	chr15	85212775	85259947	295	0.103	1	1	4.916E-03	1.224E-02	PB-GT
MLL5	myeloid/lymphoid or mixed-lineage leukemia 5 (trithorax homolog, Drosophila) [Source:HGNC Symbol;Acc:18541]	chr7	104654626	104754808	908	0.337	2	1	4.972E-03	1.237E-02	PB-GT
SCCPDH	saccharopine dehydrogenase (putative) [Source:HGNC Symbol;Acc:24275]	chr1	246887349	246931439	292	0.104	1	1	5.015E-03	1.246E-02	PB-GT
FERMT1	fermitin family member 1 [Source:HGNC Symbol;Acc:15889]	chr20	6055492	6104191	287	0.104	1	1	5.046E-03	1.253E-02	PB-GT
ASRGL1	asparaginase like 1 [Source:HGNC Symbol;Acc:16448]	chr11	62104920	62160882	300	0.104	1	1	5.057E-03	1.255E-02	PB-GT
MET	met proto-oncogene (hepatocyte growth factor receptor) [Source:HGNC Symbol;Acc:7029]	chr7	116312444	116438440	915	0.340	2	1	5.078E-03	1.259E-02	PB-GT
PLXNC1	plexin C1 [Source:HGNC Symbol;Acc:9106]	chr12	94542499	94701451	889	0.340	2	1	5.102E-03	1.264E-02	PB-GT
NPC1	Niemann-Pick disease, type C1 [Source:HGNC Symbol;Acc:7897]	chr18	21086307	21166862	437	0.105	1	1	5.110E-03	1.264E-02	PB-GT
RCHY1	ring finger and CHY zinc finger domain containing 1, E3 ubiquitin protein ligase [Source:HGNC Symbol;Acc:17479]	chr4	76404247	76439974	371	0.105	1	1	5.145E-03	1.272E-02	PB-GT
ARL6	ADP-ribosylation factor-like 6 [Source:HGNC Symbol;Acc:13210]	chr3	97483365	97519953	343	0.106	1	1	5.190E-03	1.281E-02	PB-GT
ZNF461	zinc finger protein 461 [Source:HGNC Symbol;Acc:21629]	chr19	37128094	37157755	200	0.106	1	1	5.192E-03	1.281E-02	PB-GT
SETDB2	SET domain, bifurcated 2 [Source:HGNC Symbol;Acc:20263]	chr13	50018429	50069138	435	0.106	1	1	5.199E-03	1.282E-02	PB-GT
ACBD3	acyl-CoA binding domain containing 3 [Source:HGNC Symbol;Acc:15453]	chr1	226332380	226374431	298	0.106	1	1	5.216E-03	1.283E-02	PB-GT
MYB	v-myb myeloblastosis viral oncogene homolog (avian) [Source:HGNC Symbol;Acc:7545]	chr6	135502453	135540311	308	0.106	1	1	5.217E-03	1.283E-02	PB-GT
TMEM189	transmembrane protein 189 [Source:HGNC Symbol;Acc:16735]	chr20	48697663	48770335	292	0.106	1	1	5.218E-03	1.283E-02	PB-GT
GMDS	GDP-mannose 4,6-dehydratase [Source:HGNC Symbol;Acc:4369]	chr6	1624041	2245926	4517	1.552	5	2	5.230E-03	1.285E-02	PB-GT
SNX6	sorting nexin 6 [Source:HGNC Symbol;Acc:14970]	chr14	35030300	35099389	391	0.106	1	1	5.240E-03	1.285E-02	PB-GT
METTL21A	methyltransferase like 21A [Source:HGNC Symbol;Acc:30476]	chr2	208445355	208490652	371	0.106	1	1	5.245E-03	1.285E-02	PB-GT
NEK4	NIMA-related kinase 4 [Source:HGNC Symbol;Acc:11399]	chr3	52744800	52804965	345	0.106	1	1	5.249E-03	1.285E-02	PB-GT
TMEM189-UBE2'	TMEM189-UBE2V1 readthrough [Source:HGNC Symbol;Acc:33521]	chr20	48697661	48770174	293	0.106	1	1	5.252E-03	1.285E-02	PB-GT
ADNP	activity-dependent neuroprotector homeobox [Source:HGNC Symbol;Acc:15766]	chr20	49505453	49547958	293	0.106	1	1	5.252E-03	1.285E-02	PB-GT
FAM92A1	family with sequence similarity 92, member A1 [Source:HGNC Symbol;Acc:30452]	chr8	94710789	94743755	310	0.106	1	1	5.256E-03	1.285E-02	PB-GT
MID2	midline 2 [Source:HGNC Symbol;Acc:7096]	chrX	107068985	107170423	641	0.107	1	1	5.313E-03	1.298E-02	PB-GT
MSL2	male-specific lethal 2 homolog (Drosophila) [Source:HGNC Symbol;Acc:25544]	chr3	135867764	135916083	348	0.107	1	1	5.337E-03	1.303E-02	PB-GT
SLC35E3	solute carrier family 35, member E3 [Source:HGNC Symbol;Acc:20864]	chr12	69139886	69187744	280	0.107	1	1	5.351E-03	1.305E-02	PB-GT
AASDH	aminoadipate-semialdehyde dehydrogenase [Source:HGNC Symbol;Acc:23993]	chr4	57204456	57253666	379	0.107	1	1	5.361E-03	1.306E-02	PB-GT
KIAA0922	KIAA0922 [Source:HGNC Symbol;Acc:29146]	chr4	154387498	154557863	1226	0.347	2	1	5.385E-03	1.309E-02	PB-GT
LONRF1	LON peptidase N-terminal domain and ring finger 1 [Source:HGNC Symbol;Acc:26302]	chr8	12579403	12613582	314	0.108	1	1	5.388E-03	1.309E-02	PB-GT
HERC5	HECT and RLD domain containing E3 ubiquitin protein ligase 5 [Source:HGNC Symbol;Acc:24368]	chr4	89378268	89427314	380	0.108	1	1	5.389E-03	1.309E-02	PB-GT
CEACAM21	carcinoembryonic antigen-related cell adhesion molecule 21 [Source:HGNC Symbol;Acc:28834]	chr19	42055886	42093197	204	0.108	1	1	5.394E-03	1.309E-02	PB-GT
AP4S1	adaptor-related protein complex 4, sigma 1 subunit [Source:HGNC Symbol;Acc:575]	chr14	31494312	31562818	397	0.108	1	1	5.397E-03	1.309E-02	PB-GT
ACTN4	actinin, alpha 4 [Source:HGNC Symbol;Acc:166]	chr19	39138289	39222223	205	0.108	1	1	5.445E-03	1.320E-02	PB-GT
TSGA10	testis specific, 10 [Source:HGNC Symbol;Acc:14927]	chr2	99613724	99771427	1219	0.349	2	1	5.449E-03	1.320E-02	PB-GT
KTN1	kinectin 1 (kinesin receptor) [Source:HGNC Symbol;Acc:6467]	chr14	56025790	56168244	1286	0.349	2	1	5.456E-03	1.320E-02	PB-GT
LCMT1	leucine carboxyl methyltransferase 1 [Source:HGNC Symbol;Acc:17557]	chr16	25123050	25189552	324	0.109	1	1	5.481E-03	1.325E-02	PB-GT
SYNRG	synergin, gamma [Source:HGNC Symbol;Acc:557]	chr17	35874900	35969544	691	0.350	2	1	5.510E-03	1.331E-02	PB-GT
RECQL	RecQ protein-like (DNA helicase Q1-like) [Source:HGNC Symbol;Acc:9948]	chr12	21621845	21654603	285	0.109	1	1	5.537E-03	1.337E-02	PB-GT
NAP1L4	nucleosome assembly protein 1-like 4 [Source:HGNC Symbol;Acc:7640]	chr11	2965667	3013607	315	0.109	1	1	5.556E-03	1.340E-02	PB-GT
DIDO1	death inducer-obliterator 1 [Source:HGNC Symbol;Acc:2680]	chr20	61509090	61569304	302	0.109	1	1	5.568E-03	1.342E-02	PB-GT
C2orf47	chromosome 2 open reading frame 47 [Source:HGNC Symbol;Acc:26198]	chr2	200820040	200873263	383	0.110	1	1	5.578E-03	1.342E-02	PB-GT
FAM133B	family with sequence similarity 133, member B [Source:HGNC Symbol;Acc:28629]	chr7	92190107	92219708	295	0.110	1	1	5.579E-03	1.342E-02	PB-GT
AGAP11	ankyrin repeat and GTPase domain Arf GTPase activating protein 11 [Source:HGNC Symbol;Acc:29421]	chr10	88728247	88784489	307	0.110	1	1	5.581E-03	1.342E-02	PB-GT
FRYL	FRY-like [Source:HGNC Symbol;Acc:29127]	chr4	48499378	48782339	2457	0.696	3	2	5.631E-03	1.352E-02	PB-GT
MOCOS	molybdenum cofactor sulfurase [Source:HGNC Symbol;Acc:18234]	chr18	33767482	33852120	460	0.110	1	1	5.641E-03	1.354E-02	PB-GT
H2AFY2	H2A histone family, member Y2 [Source:HGNC Symbol;Acc:14453]	chr10	71812552	71872036	309	0.110	1	1	5.652E-03	1.355E-02	PB-GT
TRAK1	trafficking protein, kinesin binding 1 [Source:HGNC Symbol;Acc:29947]	chr3	42055294	42267381	1150	0.354	2	1	5.673E-03	1.358E-02	PB-GT
SUB1	SUB1 homolog (S. cerevisiae) [Source:HGNC Symbol;Acc:19985]	chr5	32531739	32604185	410	0.110	1	1	5.673E-03	1.358E-02	PB-GT
DNAJC12	DnaJ (Hsp40) homolog, subfamily C, member 12 [Source:HGNC Symbol;Acc:28908]	chr10	69556427	69597924	310	0.111	1	1	5.687E-03	1.359E-02	PB-GT
NNMT	nicotinamide N-methyltransferase [Source:HGNC Symbol;Acc:7861]	chr11	114128509	114184007	319	0.111	1	1	5.693E-03	1.359E-02	PB-GT
AP1AR	adaptor-related protein complex 1 associated regulatory protein [Source:HGNC Symbol;Acc:28808]	chr4	113152893	113191203	391	0.111	1	1	5.694E-03	1.359E-02	PB-GT
URB1	URB1 ribosome biogenesis 1 homolog (S. cerevisiae) [Source:HGNC Symbol;Acc:17344]	chr21	33683329	33765335	387	0.111	1	1	5.696E-03	1.359E-02	PB-GT
C16orf87	chromosome 16 open reading frame 87 [Source:HGNC Symbol;Acc:33754]	chr16	46830519	46865323	331	0.111	1	1	5.711E-03	1.361E-02	PB-GT
DDHD2	DDHD domain containing 2 [Source:HGNC Symbol;Acc:29106]	chr8	38082736	38133076	324	0.111	1	1	5.723E-03	1.363E-02	PB-GT
UGCG	UDP-glucose ceramide glucosyltransferase [Source:HGNC Symbol;Acc:12524]	chr9	114659046	114697649	296	0.111	1	1	5.726E-03	1.363E-02	PB-GT
MTMR6	myotubularin related protein 6 [Source:HGNC Symbol;Acc:7453]	chr13	25802307	25862147	458	0.111	1	1	5.743E-03	1.366E-02	PB-GT
HLA-AS1	HLA antisense RNA 1 [Source:HGNC Symbol;Acc:42509]	chr1	221006105	221053482	314	0.111	1	1	5.770E-03	1.371E-02	PB-GT
TLR1	toll-like receptor 1 [Source:HGNC Symbol;Acc:11847]	chr4	38792298	38858438	394	0.112	1	1	5.778E-03	1.372E-02	PB-GT
PCDH7	protocadherin 7 [Source:HGNC Symbol;Acc:8659]	chr4	30722037	31148422	3945	1.117	4	1	5.787E-03	1.373E-02	PB-GT
MEMO1	mediator of cell motility 1 [Source:HGNC Symbol;Acc:14014]	chr2	32092878	32236299	1247	0.357	2	1	5.798E-03	1.374E-02	PB-GT
RBFOX2	RNA binding protein, fox-1 homolog (C. elegans) 2 [Source:HGNC Symbol;Acc:9906]	chr22	36134783	36424473	2238	1.118	4	2	5.812E-03	1.376E-02	PB-GT
ERLIN1	ER lipid raft associated 1 [Source:HGNC Symbol;Acc:16947]	chr10	101909851	101948091	314	0.112	1	1	5.829E-03	1.378E-02	PB-GT
FAM175A	family with sequence similarity 175, member A [Source:HGNC Symbol;Acc:25829]	chr4	84382092	84444501	396	0.112	1	1	5.835E-03	1.378E-02	PB-GT
ING3	inhibitor of growth family, member 3 [Source:HGNC Symbol;Acc:14587]	chr7	120590803	120617270	302	0.112	1	1	5.837E-03	1.378E-02	PB-GT
CRYZL1	crystallin, zeta (quinone reductase)-like 1 [Source:HGNC Symbol;Acc:2420]	chr21	34961647	35016232	392	0.112	1	1	5.839E-03	1.378E-02	PB-GT
LINS	lines homolog (Drosophila) [Source:HGNC Symbol;Acc:30922]	chr15	101099574	101143435	323	0.112	1	1	5.856E-03	1.381E-02	PB-GT
POLR2B	polymerase (RNA) II (DNA directed) polypeptide B, 140kDa [Source:HGNC Symbol;Acc:9188]	chr4	57843888	57897334	397	0.112	1	1	5.863E-03	1.381E-02	PB-GT
CLTC	clathrin, heavy chain (Hc) [Source:HGNC Symbol;Acc:2092]	chr17	57697219	57773671	707	0.358	2	1	5.866E-03	1.381E-02	PB-GT
TOP2A	topoisomerase (DNA) II alpha 170kDa [Source:HGNC Symbol;Acc:11989]	chr17	38544768	38574202	222	0.112	1	1	5.868E-03	1.381E-02	PB-GT
LRCH1	leucine-rich repeats and calponin homology (CH) domain containing 1 [Source:HGNC Symbol;Acc:20309]	chr13	47127303	47327175	1477	0.359	2	1	5.888E-03	1.384E-02	PB-GT
RTFDC1	replication termination factor 2 domain containing 1 [Source:HGNC Symbol;Acc:15890]	chr20	55043647	55093943	311	0.113	1	1	5.892E-03	1.384E-02	PB-GT
EIF3A	eukaryotic translation initiation factor 3, subunit A [Source:HGNC Symbol;Acc:3271]	chr10	120794356	120840396	316	0.113	1	1	5.901E-03	1.384E-02	PB-GT
TOR1AIP1	torsin A interacting protein 1 [Source:HGNC Symbol;Acc:29456]	chr1	179851177	179888412	318	0.113	1	1	5.912E-03	1.384E-02	PB-GT
LNP1	leukemia NUP98 fusion partner 1 [Source:HGNC Symbol;Acc:28014]	chr3	100120037	100175163	367	0.113	1	1	5.913E-03	1.384E-02	PB-GT
DCP2	DCP2 decapping enzyme homolog (S. cerevisiae) [Source:HGNC Symbol;Acc:24452]	chr5	112312399	112356667	419	0.113	1	1	5.915E-03	1.384E-02	PB-GT
ZNF85	zinc finger protein 85 [Source:HGNC Symbol;Acc:13160]	chr19	21106028	21133503	214	0.113	1	1	5.915E-03	1.384E-02	PB-GT
CCDC126	coiled-coil domain containing 126 [Source:HGNC Symbol;Acc:22398]	chr7	23636998	23684327	305	0.113	1	1	5.949E-03	1.390E-02	PB-GT
IFNGR2	interferon gamma receptor 2 (interferon gamma transducer 1) [Source:HGNC Symbol;Acc:5440]	chr21	34775202	34851655	396	0.113	1	1	5.954E-03	1.390E-02	PB-GT
FAM217A	family with sequence similarity 217, member A [Source:HGNC Symbol;Acc:21362]	chr6	4049668	4087578	330	0.113	1	1	5.959E-03	1.391E-02	PB-GT
PTGIS	prostaglandin I2 (prostacyclin) synthase [Source:HGNC Symbol;Acc:9603]	chr20	48120411	48184683	313	0.113	1	1	5.965E-03	1.391E-02	PB-GT
SLC25A31	solute carrier family 25 (mitochondrial carrier; adenine nucleotide translocator), member 31 [Source:HGNC Symbol;Acc:25319]	chr4	128651533	128695448	401	0.114	1	1	5.977E-03	1.392E-02	PB-GT
MORC3	MORC family CW-type zinc finger 3 [Source:HGNC Symbol;Acc:23572]	chr21	37692487	37758446	397	0.114	1	1	5.983E-03	1.392E-02	PB-GT
DYNC1H1	dynein, cytoplasmic 1, heavy chain 1 [Source:HGNC Symbol;Acc:2961]	chr14	102430865	102517129	419	0.114	1	1	5.988E-03	1.392E-02	PB-GT
ETV1	ets variant 1 [Source:HGNC Symbol;Acc:3490]	chr7	13930853	14031050	972	0.361	2	1	5.993E-03	1.392E-02	PB-GT
MRPL42	mitochondrial ribosomal protein L42 [Source:HGNC Symbol;Acc:14493]	chr12	93861264	93897545	297	0.114	1	1	5.995E-03	1.392E-02	PB-GT
UBE3C	ubiquitin protein ligase E3C [Source:HGNC Symbol;Acc:16803]	chr7	156931607	157062066	973	0.361	2	2	6.010E-03	1.394E-02	PB-GT
ANXA2	annexin A2 [Source:HGNC Symbol;Acc:537]	chr15	60639333	60695082	328	0.114	1	1	6.032E-03	1.398E-02	PB-GT
SMC4	structural maintenance of chromosomes 4 [Source:HGNC Symbol;Acc:14013]	chr3	160117062	160152750	371	0.114	1	1	6.038E-03	1.399E-02	PB-GT
STK32C	serine/threonine kinase 32C [Source:HGNC Symbol;Acc:21332]	chr10	134020996	134145351	320	0.114	1	1	6.045E-03	1.399E-02	PB-GT
HORMAD2	HORMA domain containing 2 [Source:HGNC Symbol;Acc:28383]	chr22	30476163	30573064	725	0.362	2	1	6.051E-03	1.399E-02	PB-GT
THUMPD2	THUMP domain containing 2 [Source:HGNC Symbol;Acc:14890]	chr2	39963200	40006407	400	0.114	1	1	6.064E-03	1.401E-02	PB-GT
SHROOM2	shroom family member 2 [Source:HGNC Symbol;Acc:630]	chrX	9754496	9917483	688	0.115	1	1	6.089E-03	1.405E-02	PB-GT
ZMYM1	zinc finger, MYM-type 1 [Source:HGNC Symbol;Acc:26253]	chr1	35525387	35581460	323	0.115	1	1	6.092E-03	1.405E-02	PB-GT
RTF1	Rtf1, Paf1/RNA polymerase II complex component, homolog (S. cerevisiae) [Source:HGNC Symbol;Acc:28996]	chr15	41700606	41775761	330	0.115	1	1	6.102E-03	1.407E-02	PB-GT

table S2. Genes with significant transposon integrations in RKO *BRAF*$^{wt/-}$ cells after selection in low glucose medium

Gene ID	Description	Chrom	Start	End	# TTAA sites	Expected	Observed	Libraries	*P*	FDR	Transposon
GTF2H1	general transcription factor IIH, polypeptide 1, 62kDa [Source:HGNC Symbol;Acc:4655]	chr11	18343842	18388591	331	0.115	1	1	6.112E-03	1.408E-02	PB-GT
FHL2	four and a half LIM domains 2 [Source:HGNC Symbol;Acc:3703]	chr2	105974169	106054960	402	0.115	1	1	6.123E-03	1.409E-02	PB-GT
IQCG	IQ motif containing G [Source:HGNC Symbol;Acc:25251]	chr3	197615946	197687013	374	0.115	1	1	6.132E-03	1.409E-02	PB-GT
FYCO1	FYVE and coiled-coil domain containing 1 [Source:HGNC Symbol;Acc:14673]	chr3	45959396	46037316	374	0.115	1	1	6.132E-03	1.409E-02	PB-GT
SPRED1	sprouty-related, EVH1 domain containing 1 [Source:HGNC Symbol;Acc:20249]	chr15	38544527	38649450	1049	0.365	2	1	6.170E-03	1.414E-02	PB-GT
AGO2	argonaute RISC catalytic component 2 [Source:HGNC Symbol;Acc:3263]	chr8	141541264	141645718	337	0.115	1	1	6.174E-03	1.414E-02	PB-GT
PHACTR2	phosphatase and actin regulator 2 [Source:HGNC Symbol;Acc:20956]	chr6	143857982	144152322	2080	0.714	3	2	6.175E-03	1.414E-02	PB-GT
DBF4	DBF4 homolog (S. cerevisiae) [Source:HGNC Symbol;Acc:17364]	chr7	87505531	87538856	311	0.115	1	1	6.177E-03	1.414E-02	PB-GT
HS2ST1	heparan sulfate 2-O-sulfotransferase 1 [Source:HGNC Symbol;Acc:5193]	chr1	87380331	87602334	2013	0.715	3	2	6.178E-03	1.414E-02	PB-GT
SMEK1	SMEK homolog 1, suppressor of mek1 (Dictyostelium) [Source:HGNC Symbol;Acc:20219]	chr14	91923955	91976898	426	0.116	1	1	6.182E-03	1.414E-02	PB-GT
GFM2	G elongation factor, mitochondrial 2 [Source:HGNC Symbol;Acc:29682]	chr5	74017029	74063196	429	0.116	1	1	6.190E-03	1.414E-02	PB-GT
ATR	ataxia telangiectasia and Rad3 related [Source:HGNC Symbol;Acc:882]	chr3	142168077	142297668	1189	0.366	2	1	6.215E-03	1.419E-02	PB-GT
ZFP14	ZFP14 zinc finger protein [Source:HGNC Symbol;Acc:29312]	chr19	36827162	36870101	220	0.116	1	1	6.239E-03	1.423E-02	PB-GT
BBS4	Bardet-Biedl syndrome 4 [Source:HGNC Symbol;Acc:969]	chr15	72978527	73030817	334	0.116	1	1	6.246E-03	1.424E-02	PB-GT
CPT1A	carnitine palmitoyltransferase 1A (liver) [Source:HGNC Symbol;Acc:2328]	chr11	68522088	68611878	335	0.116	1	1	6.255E-03	1.425E-02	PB-GT
SEMA3D	sema domain, immunoglobulin domain (Ig), short basic domain, secreted, (semaphorin) 3D [Source:HGNC Symbol;Acc:10726]	chr7	84624869	84816171	1933	0.718	3	1	6.274E-03	1.428E-02	PB-GT
SMYD4	SET and MYND domain containing 4 [Source:HGNC Symbol;Acc:21067]	chr17	1682779	1733928	230	0.117	1	1	6.282E-03	1.429E-02	PB-GT
DNAJC16	DnaJ (Hsp40) homolog, subfamily C, member 16 [Source:HGNC Symbol;Acc:29157]	chr1	15853308	15918874	329	0.117	1	1	6.312E-03	1.434E-02	PB-GT
ZNF66	zinc finger protein 66 [Source:HGNC Symbol;Acc:13135]	chr19	20959110	20991922	222	0.117	1	1	6.348E-03	1.440E-02	PB-GT
CLEC2D	C-type lectin domain family 2, member D [Source:HGNC Symbol;Acc:14351]	chr12	9809270	9848413	306	0.117	1	1	6.350E-03	1.440E-02	PB-GT
ESCO2	establishment of cohesion 1 homolog 2 (S. cerevisiae) [Source:HGNC Symbol;Acc:27230]	chr8	27629466	27670157	342	0.117	1	1	6.351E-03	1.440E-02	PB-GT
COPS5	COP9 signalosome subunit 5 [Source:HGNC Symbol;Acc:2240]	chr8	67955314	67996018	344	0.118	1	1	6.423E-03	1.454E-02	PB-GT
GNB5	guanine nucleotide binding protein (G protein), beta 5 [Source:HGNC Symbol;Acc:4401]	chr15	52413117	52483566	339	0.118	1	1	6.427E-03	1.454E-02	PB-GT
BMX	BMX non-receptor tyrosine kinase [Source:HGNC Symbol;Acc:1079]	chrX	15482369	15574652	708	0.118	1	1	6.434E-03	1.455E-02	PB-GT
CCDC88A	coiled-coil domain containing 88A [Source:HGNC Symbol;Acc:25523]	chr2	55514978	55647057	1297	0.371	2	1	6.456E-03	1.459E-02	PB-GT
LATS2	large tumor suppressor kinase 2 [Source:HGNC Symbol;Acc:6515]	chr13	21547171	21635686	488	0.118	1	1	6.488E-03	1.464E-02	PB-GT
C6orf89	chromosome 6 open reading frame 89 [Source:HGNC Symbol;Acc:21114]	chr6	36839646	36892331	345	0.119	1	1	6.491E-03	1.464E-02	PB-GT
GTF2A1	general transcription factor IIA, 1, 19/37kDa [Source:HGNC Symbol;Acc:4646]	chr14	81641796	81687721	437	0.119	1	1	6.492E-03	1.464E-02	PB-GT
OCLN	occludin [Source:HGNC Symbol;Acc:8104]	chr5	68788119	68853931	441	0.119	1	1	6.527E-03	1.470E-02	PB-GT
SMARCC1	SWI/SNF related, matrix associated, actin dependent regulator of chromatin, subfamily c, member 1 [Source:HGNC Symbol;Ac	chr3	47626762	47823596	1211	0.373	2	2	6.533E-03	1.471E-02	PB-GT
DSC3	desmocollin 3 [Source:HGNC Symbol;Acc:3037]	chr18	28569974	28622781	497	0.119	1	1	6.547E-03	1.472E-02	PB-GT
MARCH1	membrane-associated ring finger (C3HC4) 1, E3 ubiquitin protein ligase [Source:HGNC Symbol;Acc:26077]	chr4	164445450	165305202	7579	2.146	6	2	6.560E-03	1.473E-02	PB-GT
SGK3	serum/glucocorticoid regulated kinase family, member 3 [Source:HGNC Symbol;Acc:10812]	chr8	67624653	67774257	1089	0.373	2	1	6.560E-03	1.473E-02	PB-GT
NUP62CL	nucleoporin 62kDa C-terminal like [Source:HGNC Symbol;Acc:25960]	chrX	106366657	106449670	716	0.119	1	1	6.574E-03	1.475E-02	PB-GT
SLC9A4	solute carrier family 9, subfamily A (NHE4, cation proton antiporter 4), member 4 [Source:HGNC Symbol;Acc:11077]	chr2	103089762	103150431	419	0.120	1	1	6.630E-03	1.486E-02	PB-GT
SOCS7	suppressor of cytokine signaling 7 [Source:HGNC Symbol;Acc:29846]	chr17	36508111	36556019	237	0.120	1	1	6.655E-03	1.491E-02	PB-GT
ATL3	atlastin GTPase 3 [Source:HGNC Symbol;Acc:24526]	chr11	63391559	63439393	347	0.120	1	1	6.693E-03	1.498E-02	PB-GT
ENTPD7	ectonucleoside triphosphate diphosphohydrolase 7 [Source:HGNC Symbol;Acc:19745]	chr10	101419263	101465997	338	0.121	1	1	6.716E-03	1.502E-02	PB-GT
DLGAP1	discs, large (Drosophila) homolog-associated protein 1 [Source:HGNC Symbol;Acc:2905]	chr18	3496030	4455335	6836	1.637	5	2	6.724E-03	1.502E-02	PB-GT
RAD18	RAD18 homolog (S. cerevisiae) [Source:HGNC Symbol;Acc:18278]	chr3	8817088	9005457	1224	0.377	2	2	6.726E-03	1.502E-02	PB-GT
CHUK	conserved helix-loop-helix ubiquitous kinase [Source:HGNC Symbol;Acc:1974]	chr10	101948055	101989376	339	0.121	1	1	6.754E-03	1.507E-02	PB-GT
HELB	helicase (DNA) B [Source:HGNC Symbol;Acc:17196]	chr12	66696325	66737423	317	0.121	1	1	6.795E-03	1.515E-02	PB-GT
C7orf31	chromosome 7 open reading frame 31 [Source:HGNC Symbol;Acc:21722]	chr7	25174316	25219975	327	0.121	1	1	6.802E-03	1.515E-02	PB-GT
PSMD11	proteasome (prosome, macropain) 26S subunit, non-ATPase, 11 [Source:HGNC Symbol;Acc:9556]	chr17	30771279	30810336	240	0.122	1	1	6.817E-03	1.517E-02	PB-GT
ZNRD1-AS1	ZNRD1 antisense RNA 1 [Source:HGNC Symbol;Acc:13924]	chr6	29968788	30029417	354	0.122	1	1	6.820E-03	1.517E-02	PB-GT
ANKS1A	ankyrin repeat and sterile alpha motif domain containing 1A [Source:HGNC Symbol;Acc:20961]	chr6	34857042	35059179	1102	0.379	2	1	6.825E-03	1.517E-02	PB-GT
MDM2	MDM2 oncogene, E3 ubiquitin protein ligase [Source:HGNC Symbol;Acc:6973]	chr12	69201956	69239214	318	0.122	1	1	6.837E-03	1.518E-02	PB-GT
YAE1D1	Yae1 domain containing 1 [Source:HGNC Symbol;Acc:24857]	chr7	39605975	39649919	328	0.122	1	1	6.842E-03	1.518E-02	PB-GT
HOOK2	hook homolog 2 (Drosophila) [Source:HGNC Symbol;Acc:19885]	chr19	12873817	12983554	231	0.122	1	1	6.852E-03	1.520E-02	PB-GT
PDCD4	programmed cell death 4 (neoplastic transformation inhibitor) [Source:HGNC Symbol;Acc:8763]	chr10	112631565	112659764	342	0.122	1	1	6.870E-03	1.522E-02	PB-GT
LPO	lactoperoxidase [Source:HGNC Symbol;Acc:6678]	chr17	56295909	56345879	242	0.123	1	1	6.927E-03	1.534E-02	PB-GT
IL20RB	interleukin 20 receptor beta [Source:HGNC Symbol;Acc:6004]	chr3	136665072	136729927	399	0.123	1	1	6.944E-03	1.536E-02	PB-GT
SUPT20H	suppressor of Ty 20 homolog (S. cerevisiae) [Source:HGNC Symbol;Acc:20596]	chr13	37583449	37633850	506	0.123	1	1	6.956E-03	1.538E-02	PB-GT
HGSNAT	heparan-alpha-glucosaminide N-acetyltransferase [Source:HGNC Symbol;Acc:26527]	chr8	42995556	43057998	359	0.123	1	1	6.971E-03	1.540E-02	PB-GT
FAM172A	family with sequence similarity 172, member A [Source:HGNC Symbol;Acc:25365]	chr5	92953775	93447404	4339	1.169	4	2	6.976E-03	1.540E-02	PB-GT
COL3A1	collagen, type III, alpha 1 [Source:HGNC Symbol;Acc:2201]	chr2	189839046	189877472	432	0.124	1	1	7.031E-03	1.551E-02	PB-GT
PTBP3	polypyrimidine tract binding protein 3 [Source:HGNC Symbol;Acc:10253]	chr9	114980715	115095947	1021	0.383	2	1	7.044E-03	1.552E-02	PB-GT
TBCA	tubulin folding cofactor A [Source:HGNC Symbol;Acc:11579]	chr5	76986991	77164604	1422	0.383	2	2	7.057E-03	1.554E-02	PB-GT
ADAM17	ADAM metallopeptidase domain 17 [Source:HGNC Symbol;Acc:195]	chr2	9628615	9695921	433	0.124	1	1	7.062E-03	1.554E-02	PB-GT
SMCO4	single-pass membrane protein with coiled-coil domains 4 [Source:HGNC Symbol;Acc:24810]	chr11	93211638	93276674	357	0.124	1	1	7.068E-03	1.554E-02	PB-GT
TMEM14B	transmembrane protein 14B [Source:HGNC Symbol;Acc:21384]	chr6	10747992	10930656	1117	0.384	2	1	7.080E-03	1.556E-02	PB-GT
LINC00630	long intergenic non-protein coding RNA 630 [Source:HGNC Symbol;Acc:44263]	chrX	102024089	102140334	745	0.124	1	1	7.095E-03	1.558E-02	PB-GT
RAB18	RAB18, member RAS oncogene family [Source:HGNC Symbol;Acc:14244]	chr10	27793197	27831143	348	0.124	1	1	7.103E-03	1.558E-02	PB-GT
RNASEH2B	ribonuclease H2, subunit B [Source:HGNC Symbol;Acc:25671]	chr13	51483814	51544592	512	0.124	1	1	7.115E-03	1.560E-02	PB-GT
DHX29	DEAH (Asp-Glu-Ala-His) box polypeptide 29 [Source:HGNC Symbol;Acc:15815]	chr5	54552073	54603550	462	0.125	1	1	7.137E-03	1.563E-02	PB-GT
FAM196A	family with sequence similarity 196, member A [Source:HGNC Symbol;Acc:33859]	chr10	128933694	128994422	349	0.125	1	1	7.142E-03	1.563E-02	PB-GT
RMDN1	regulator of microtubule dynamics 1 [Source:HGNC Symbol;Acc:24285]	chr8	87480486	87526586	364	0.125	1	1	7.159E-03	1.566E-02	PB-GT
RBMS2	RNA binding motif, single stranded interacting protein 2 [Source:HGNC Symbol;Acc:9909]	chr12	56915713	56984745	326	0.125	1	1	7.170E-03	1.567E-02	PB-GT
C7orf60	chromosome 7 open reading frame 60 [Source:HGNC Symbol;Acc:26475]	chr7	112459202	112579971	1041	0.387	2	1	7.225E-03	1.577E-02	PB-GT
CD46	CD46 molecule, complement regulatory protein [Source:HGNC Symbol;Acc:6953]	chr1	207925402	207968858	353	0.125	1	1	7.225E-03	1.577E-02	PB-GT
TMPRSS11A	transmembrane protease, serine 11A [Source:HGNC Symbol;Acc:27954]	chr4	68775103	68829858	444	0.126	1	1	7.269E-03	1.584E-02	PB-GT
WWP1	WW domain containing E3 ubiquitin protein ligase 1 [Source:HGNC Symbol;Acc:17004]	chr8	87354967	87490649	1131	0.387	2	2	7.272E-03	1.584E-02	PB-GT
APBB2	amyloid beta (A4) precursor protein-binding, family B, member 2 [Source:HGNC Symbol;Acc:582]	chr4	40812044	41218731	2649	0.750	3	1	7.292E-03	1.588E-02	PB-GT
ACTR6	ARP6 actin-related protein 6 homolog (yeast) [Source:HGNC Symbol;Acc:24025]	chr12	100592900	100635643	329	0.126	1	1	7.297E-03	1.588E-02	PB-GT
MYO1B	myosin IB [Source:HGNC Symbol;Acc:7596]	chr2	192109911	192290115	1358	0.388	2	2	7.316E-03	1.590E-02	PB-GT
RUNX1	runt-related transcription factor 1 [Source:HGNC Symbol;Acc:10471]	chr21	36160098	37357047	7663	2.193	6	2	7.333E-03	1.593E-02	PB-GT
HERC6	HECT and RLD domain containing E3 ubiquitin protein ligase family member 6 [Source:HGNC Symbol;Acc:26072]	chr4	89299891	89364263	447	0.127	1	1	7.364E-03	1.598E-02	PB-GT
SYNPO2	synaptopodin 2 [Source:HGNC Symbol;Acc:17732]	chr4	119809996	119982402	1377	0.390	2	1	7.394E-03	1.604E-02	PB-GT
SLCO2B1	solute carrier organic anion transporter family, member 2B1 [Source:HGNC Symbol;Acc:10962]	chr11	74811608	74917594	367	0.127	1	1	7.452E-03	1.615E-02	PB-GT
SMAD9	SMAD family member 9 [Source:HGNC Symbol;Acc:6774]	chr13	37418968	37494902	525	0.127	1	1	7.465E-03	1.617E-02	PB-GT
EVA1C	eva-1 homolog C (C. elegans) [Source:HGNC Symbol;Acc:13239]	chr21	33784314	33887707	446	0.128	1	1	7.482E-03	1.619E-02	PB-GT
UBE2V2	ubiquitin-conjugating enzyme E2 variant 2 [Source:HGNC Symbol;Acc:12495]	chr8	48920960	48977268	373	0.128	1	1	7.502E-03	1.622E-02	PB-GT
NMT2	N-myristoyltransferase 2 [Source:HGNC Symbol;Acc:7858]	chr10	15144583	15210692	359	0.128	1	1	7.539E-03	1.628E-02	PB-GT
PEX3	peroxisomal biogenesis factor 3 [Source:HGNC Symbol;Acc:8858]	chr6	143771944	143811147	373	0.128	1	1	7.540E-03	1.628E-02	PB-GT
MGST2	microsomal glutathione S-transferase 2 [Source:HGNC Symbol;Acc:7063]	chr4	140586922	140661899	453	0.128	1	1	7.554E-03	1.630E-02	PB-GT
NLRC5	NLR family, CARD domain containing 5 [Source:HGNC Symbol;Acc:29933]	chr16	57023397	57117443	383	0.128	1	1	7.559E-03	1.630E-02	PB-GT
PPP1CB	protein phosphatase 1, catalytic subunit, beta isozyme [Source:HGNC Symbol;Acc:9282]	chr2	28974506	29025806	449	0.128	1	1	7.571E-03	1.630E-02	PB-GT
FBXO28	F-box protein 28 [Source:HGNC Symbol;Acc:29046]	chr1	224301789	224349749	362	0.129	1	1	7.583E-03	1.630E-02	PB-GT
STX6	syntaxin 6 [Source:HGNC Symbol;Acc:11441]	chr1	180941861	180992047	362	0.129	1	1	7.583E-03	1.630E-02	PB-GT
TRIM36	tripartite motif containing 36 [Source:HGNC Symbol;Acc:16280]	chr5	114460459	114516243	477	0.129	1	1	7.587E-03	1.630E-02	PB-GT
ZNF717	zinc finger protein 717 [Source:HGNC Symbol;Acc:29448]	chr3	75758794	75834734	418	0.129	1	1	7.591E-03	1.630E-02	PB-GT
CRISP1	cysteine-rich secretory protein 1 [Source:HGNC Symbol;Acc:304]	chr6	49801970	49844809	375	0.129	1	1	7.617E-03	1.633E-02	PB-GT
PPARGC1B	peroxisome proliferator-activated receptor gamma, coactivator 1 beta [Source:HGNC Symbol;Acc:30022]	chr5	149109861	149234585	478	0.129	1	1	7.618E-03	1.633E-02	PB-GT
CHAT	choline O-acetyltransferase [Source:HGNC Symbol;Acc:1912]	chr10	50817141	50901925	361	0.129	1	1	7.620E-03	1.633E-02	PB-GT
NUMA1	nuclear mitotic apparatus protein 1 [Source:HGNC Symbol;Acc:8059]	chr11	71713910	71791739	372	0.129	1	1	7.648E-03	1.638E-02	PB-GT
ADK	adenosine kinase [Source:HGNC Symbol;Acc:257]	chr10	75910960	76469061	4718	1.684	5	2	7.655E-03	1.638E-02	PB-GT
SMAD5	SMAD family member 5 [Source:HGNC Symbol;Acc:6771]	chr5	135468534	135524435	480	0.129	1	1	7.679E-03	1.641E-02	PB-GT
YTHDC1	YTH domain containing 1 [Source:HGNC Symbol;Acc:30626]	chr4	69176105	69215807	457	0.129	1	1	7.683E-03	1.641E-02	PB-GT
LYPLA1	lysophospholipase I [Source:HGNC Symbol;Acc:6737]	chr8	54958938	55014577	378	0.130	1	1	7.695E-03	1.642E-02	PB-GT
TGS1	trimethylguanosine synthase 1 [Source:HGNC Symbol;Acc:17843]	chr8	56685701	56738007	378	0.130	1	1	7.695E-03	1.642E-02	PB-GT
DRD3	dopamine receptor D3 [Source:HGNC Symbol;Acc:3024]	chr3	113847499	113918254	422	0.130	1	1	7.731E-03	1.648E-02	PB-GT
ZCCHC4	zinc finger, CCHC domain containing 4 [Source:HGNC Symbol;Acc:22917]	chr4	25314407	25372005	459	0.130	1	1	7.747E-03	1.650E-02	PB-GT
ZCCHC14	zinc finger, CCHC domain containing 14 [Source:HGNC Symbol;Acc:24134]	chr16	87439852	87525651	388	0.130	1	1	7.749E-03	1.650E-02	PB-GT
ITGAE	integrin, alpha E (antigen CD103, human mucosal lymphocyte antigen 1; alpha polypeptide) [Source:HGNC Symbol;Acc:6147]	chr17	3617922	3704537	257	0.130	1	1	7.773E-03	1.653E-02	PB-GT
HNRPLL	heterogeneous nuclear ribonucleoprotein L-like [Source:HGNC Symbol;Acc:25127]	chr2	38789120	38830728	456	0.130	1	1	7.798E-03	1.657E-02	PB-GT
MATR3	matrin 3 [Source:HGNC Symbol;Acc:6912]	chr5	138609441	138667360	484	0.130	1	1	7.802E-03	1.657E-02	PB-GT
ZFYVE28	zinc finger, FYVE domain containing 28 [Source:HGNC Symbol;Acc:29334]	chr4	2271309	2420390	461	0.131	1	1	7.812E-03	1.658E-02	PB-GT
TXNDC11	thioredoxin domain containing 11 [Source:HGNC Symbol;Acc:28030]	chr16	11772936	11836734	390	0.131	1	1	7.826E-03	1.659E-02	PB-GT
C2CD5	C2 calcium-dependent domain containing 5 [Source:HGNC Symbol;Acc:29062]	chr12	22601517	22697480	1040	0.398	2	1	7.829E-03	1.659E-02	PB-GT
WDR20	WD repeat domain 20 [Source:HGNC Symbol;Acc:19667]	chr14	102605840	102691184	482	0.131	1	1	7.835E-03	1.659E-02	PB-GT
POLA1	polymerase (DNA directed), alpha 1, catalytic subunit [Source:HGNC Symbol;Acc:9173]	chrX	24712036	25015103	2392	0.399	2	2	7.848E-03	1.661E-02	PB-GT
RNF217	ring finger protein 217 [Source:HGNC Symbol;Acc:21487]	chr6	125283691	125413779	1161	0.399	2	2	7.863E-03	1.662E-02	PB-GT
DSTYK	dual serine/threonine and tyrosine protein kinase [Source:HGNC Symbol;Acc:29043]	chr1	205111632	205180727	369	0.131	1	1	7.866E-03	1.662E-02	PB-GT
CSPP1	centrosome and spindle pole associated protein 1 [Source:HGNC Symbol;Acc:26193]	chr8	67974661	68108498	1165	0.399	2	2	7.880E-03	1.664E-02	PB-GT
ICK	intestinal cell (MAK-like) kinase [Source:HGNC Symbol;Acc:21219]	chr6	52866077	52926600	382	0.131	1	1	7.892E-03	1.665E-02	PB-GT
ADD1	adducin 1 (alpha) [Source:HGNC Symbol;Acc:243]	chr4	2845584	2931803	464	0.131	1	1	7.909E-03	1.667E-02	PB-GT
MAP4K4	mitogen-activated protein kinase kinase kinase kinase 4 [Source:HGNC Symbol;Acc:6866]	chr2	102313312	102511149	1398	0.400	2	2	7.915E-03	1.667E-02	PB-GT
L2HGDH	L-2-hydroxyglutarate dehydrogenase [Source:HGNC Symbol;Acc:20499]	chr14	50704281	50779266	485	0.132	1	1	7.928E-03	1.669E-02	PB-GT
RTN4IP1	reticulon 4 interacting protein 1 [Source:HGNC Symbol;Acc:18647]	chr6	107018903	107077373	384	0.132	1	1	7.971E-03	1.676E-02	PB-GT
NLRP11	NLR family, pyrin domain containing 11 [Source:HGNC Symbol;Acc:22945]	chr19	56296770	56348166	250	0.132	1	1	7.973E-03	1.676E-02	PB-GT
SH3PXD2B	SH3 and PX domains 2B [Source:HGNC Symbol;Acc:29242]	chr5	171752185	171881527	490	0.132	1	1	7.988E-03	1.678E-02	PB-GT
TAF1D	TATA box binding protein (TBP)-associated factor, RNA polymerase I, D, 41kDa [Source:HGNC Symbol;Acc:28759]	chr11	93463114	93517557	381	0.132	1	1	8.006E-03	1.680E-02	PB-GT
PDP1	pyruvate dehyrogenase phosphatase catalytic subunit 1 [Source:HGNC Symbol;Acc:9279]	chr8	94870035	94938294	386	0.132	1	1	8.010E-03	1.680E-02	PB-GT
ATF3	activating transcription factor 3 [Source:HGNC Symbol;Acc:785]	chr1	212738676	212794119	373	0.132	1	1	8.030E-03	1.683E-02	PB-GT
PDE7A	phosphodiesterase 7A [Source:HGNC Symbol;Acc:8791]	chr8	66629745	66754557	1175	0.403	2	2	8.064E-03	1.689E-02	PB-GT
CDKL1	cyclin-dependent kinase-like 1 (CDC2-related kinase) [Source:HGNC Symbol;Acc:1781]	chr14	50796310	50883179	490	0.133	1	1	8.086E-03	1.692E-02	PB-GT
ZC3H11A	zinc finger CCCH-type containing 11A [Source:HGNC Symbol;Acc:29093]	chr1	203764782	203823252	375	0.133	1	1	8.112E-03	1.697E-02	PB-GT
SH3BP5	SH3-domain binding protein 5 (BTK-associated) [Source:HGNC Symbol;Acc:10827]	chr3	15296360	15382875	433	0.133	1	1	8.121E-03	1.697E-02	PB-GT
SV2B	synaptic vesicle glycoprotein 2B [Source:HGNC Symbol;Acc:16874]	chr15	91643180	91844539	1162	0.404	2	2	8.148E-03	1.702E-02	PB-GT
RAD52	RAD52 homolog (S. cerevisiae) [Source:HGNC Symbol;Acc:9824]	chr12	1021243	1100356	350	0.134	1	1	8.215E-03	1.714E-02	PB-GT
DPY30	dpy-30 homolog (C. elegans) [Source:HGNC Symbol;Acc:24590]	chr2	32092878	32264881	1418	0.406	2	1	8.225E-03	1.714E-02	PB-GT
FAM228B	family with sequence similarity 228, member B [Source:HGNC Symbol;Acc:24736]	chr2	24299396	24392509	469	0.134	1	1	8.229E-03	1.714E-02	PB-GT
TBC1D4	TBC1 domain family, member 4 [Source:HGNC Symbol;Acc:19165]	chr13	75858808	76056250	1671	0.406	2	2	8.235E-03	1.714E-02	PB-GT
MNS1	meiosis-specific nuclear structural 1 [Source:HGNC Symbol;Acc:29636]	chr15	56713742	56757335	386	0.134	1	1	8.243E-03	1.714E-02	PB-GT
AK7	adenylate kinase 7 [Source:HGNC Symbol;Acc:20091]	chr14	96858448	96955764	495	0.134	1	1	8.244E-03	1.714E-02	PB-GT
PRRG1	proline rich Gla (G-carboxyglutamic acid) 1 [Source:HGNC Symbol;Acc:9469]	chrX	37208528	37316548	806	0.134	1	1	8.249E-03	1.714E-02	PB-GT
TMEM144	transmembrane protein 144 [Source:HGNC Symbol;Acc:25633]	chr4	159122756	159176563	475	0.134	1	1	8.272E-03	1.716E-02	PB-GT
TMEM150C	transmembrane protein 150C [Source:HGNC Symbol;Acc:37263]	chr4	83405743	83483510	475	0.134	1	1	8.272E-03	1.716E-02	PB-GT

table S2. Genes with significant transposon integrations in RKO *BRAF*$^{wt/-/-}$ cells after selection in low glucose medium

Gene ID	Description	Chrom	Start	End	# TTAA sites	Expected	Observed	Libraries	*P*	FDR	Transposon
NCOA3	nuclear receptor coactivator 3 [Source:HGNC Symbol;Acc:7670]	chr20	46130601	46285621	1127	0.408	2	2	8.382E-03	1.737E-02	PB-GT
NCOA7	nuclear receptor coactivator 7 [Source:HGNC Symbol;Acc:21081]	chr6	126102307	126252266	1189	0.408	2	1	8.386E-03	1.737E-02	PB-GT
SAP130	Sin3A-associated protein, 130kDa [Source:HGNC Symbol;Acc:29813]	chr2	128698791	128785694	474	0.136	1	1	8.397E-03	1.738E-02	PB-GT
WHSC1	Wolf-Hirschhorn syndrome candidate 1 [Source:HGNC Symbol;Acc:12766]	chr4	1873151	1983934	479	0.136	1	1	8.406E-03	1.739E-02	PB-GT
GOLGA1	golgin A1 [Source:HGNC Symbol;Acc:4424]	chr9	127640646	127710771	362	0.136	1	1	8.425E-03	1.742E-02	PB-GT
EFHB	EF-hand domain family, member B [Source:HGNC Symbol;Acc:26330]	chr3	19920964	19988517	442	0.136	1	1	8.447E-03	1.742E-02	PB-GT
ALDH1L1	aldehyde dehydrogenase 1 family, member L1 [Source:HGNC Symbol;Acc:3978]	chr3	125822412	125916837	442	0.136	1	1	8.447E-03	1.742E-02	PB-GT
CCDC66	coiled-coil domain containing 66 [Source:HGNC Symbol;Acc:27709]	chr3	56591189	56655846	442	0.136	1	1	8.447E-03	1.742E-02	PB-GT
EVC	Ellis van Creveld syndrome [Source:HGNC Symbol;Acc:3497]	chr4	5712924	5830772	481	0.136	1	1	8.473E-03	1.746E-02	PB-GT
STK17A	serine/threonine kinase 17a [Source:HGNC Symbol;Acc:11395]	chr7	43622357	43666385	367	0.136	1	1	8.484E-03	1.748E-02	PB-GT
URI1	URI1, prefoldin-like chaperone [Source:HGNC Symbol;Acc:13236]	chr19	30414551	30506611	779	0.411	2	1	8.532E-03	1.756E-02	PB-GT
DNAJB14	DnaJ (Hsp40) homolog, subfamily B, member 14 [Source:HGNC Symbol;Acc:25881]	chr4	100817405	100867883	483	0.137	1	1	8.540E-03	1.756E-02	PB-GT
TRIP12	thyroid hormone receptor interactor 12 [Source:HGNC Symbol;Acc:12306]	chr2	230628554	230787955	1440	0.412	2	2	8.574E-03	1.762E-02	PB-GT
NINJ2	ninjurin 2 [Source:HGNC Symbol;Acc:7825]	chr12	673462	772945	358	0.137	1	1	8.578E-03	1.762E-02	PB-GT
SLC44A3	solute carrier family 44, member 3 [Source:HGNC Symbol;Acc:28689]	chr1	95285898	95360802	387	0.137	1	1	8.615E-03	1.768E-02	PB-GT
CTNND1	catenin (cadherin-associated protein), delta 1 [Source:HGNC Symbol;Acc:2515]	chr11	57520715	57587018	396	0.137	1	1	8.619E-03	1.768E-02	PB-GT
IL17RD	interleukin 17 receptor D [Source:HGNC Symbol;Acc:17616]	chr3	57124010	57204334	447	0.138	1	1	8.630E-03	1.769E-02	PB-GT
NEIL3	nei endonuclease VIII-like 3 (E. coli) [Source:HGNC Symbol;Acc:24573]	chr4	178230990	178284097	486	0.138	1	1	8.642E-03	1.770E-02	PB-GT
ITPKB	inositol-trisphosphate 3-kinase B [Source:HGNC Symbol;Acc:6179]	chr1	226819391	226927024	388	0.138	1	1	8.658E-03	1.771E-02	PB-GT
RHEB	Ras homolog enriched in brain [Source:HGNC Symbol;Acc:10011]	chr7	151163098	151217206	371	0.138	1	1	8.662E-03	1.771E-02	PB-GT
SCUBE1	signal peptide, CUB domain, EGF-like 1 [Source:HGNC Symbol;Acc:13441]	chr22	43599229	43739394	276	0.138	1	1	8.677E-03	1.772E-02	PB-GT
ACER3	alkaline ceramidase 3 [Source:HGNC Symbol;Acc:16066]	chr11	76571911	76737841	1192	0.414	2	1	8.677E-03	1.772E-02	PB-GT
BFSP1	beaded filament structural protein 1, filensin [Source:HGNC Symbol;Acc:1040]	chr20	17474550	17549865	381	0.138	1	1	8.696E-03	1.774E-02	PB-GT
FAM114A1	family with sequence similarity 114, member A1 [Source:HGNC Symbol;Acc:25087]	chr4	38869298	38947360	488	0.138	1	1	8.710E-03	1.776E-02	PB-GT
HEXB	hexosaminidase B (beta polypeptide) [Source:HGNC Symbol;Acc:4879]	chr5	73935848	74018472	513	0.138	1	1	8.720E-03	1.777E-02	PB-GT
PALM2-AKAP2	PALM2-AKAP2 readthrough [Source:HGNC Symbol;Acc:33529]	chr9	112542589	112934792	2109	0.791	3	2	8.741E-03	1.777E-02	PB-GT
AKAP2	A kinase (PRKA) anchor protein 2 [Source:HGNC Symbol;Acc:372]	chr9	112542769	112934792	2109	0.791	3	2	8.741E-03	1.777E-02	PB-GT
SNX3	sorting nexin 3 [Source:HGNC Symbol;Acc:11174]	chr6	108532426	108582464	403	0.138	1	1	8.742E-03	1.777E-02	PB-GT
FBXW4	F-box and WD repeat domain containing 4 [Source:HGNC Symbol;Acc:10847]	chr10	103370423	103455052	388	0.138	1	1	8.747E-03	1.777E-02	PB-GT
FAM193A	family with sequence similarity 193, member A [Source:HGNC Symbol;Acc:16822]	chr4	2626988	2734292	490	0.139	1	1	8.778E-03	1.782E-02	PB-GT
CSNK1A1	casein kinase 1, alpha 1 [Source:HGNC Symbol;Acc:2451]	chr5	148871760	148931007	515	0.139	1	1	8.785E-03	1.783E-02	PB-GT
PLAA	phospholipase A2-activating protein [Source:HGNC Symbol;Acc:9043]	chr9	26904081	26947461	371	0.139	1	1	8.829E-03	1.790E-02	PB-GT
FAM21A	family with sequence similarity 21, member A [Source:HGNC Symbol;Acc:23416]	chr10	51827648	51893269	390	0.139	1	1	8.833E-03	1.790E-02	PB-GT
PTRH2	peptidyl-tRNA hydrolase 2 [Source:HGNC Symbol;Acc:24265]	chr17	57751997	57784987	275	0.139	1	1	8.847E-03	1.791E-02	PB-GT
VAMP4	vesicle-associated membrane protein 4 [Source:HGNC Symbol;Acc:12645]	chr1	171669300	171711387	393	0.140	1	1	8.872E-03	1.795E-02	PB-GT
RABL3	RAB, member of RAS oncogene family-like 3 [Source:HGNC Symbol;Acc:18072]	chr3	120405528	120461840	454	0.140	1	1	8.890E-03	1.797E-02	PB-GT
ANKRD50	ankyrin repeat domain 50 [Source:HGNC Symbol;Acc:29223]	chr4	125585207	125633887	494	0.140	1	1	8.915E-03	1.800E-02	PB-GT
PDS5B	PDS5, regulator of cohesion maintenance, homolog B (S. cerevisiae) [Source:HGNC Symbol;Acc:20418]	chr13	33160564	33352157	1721	0.418	2	2	8.917E-03	1.800E-02	PB-GT
DECR1	2,4-dienoyl CoA reductase 1, mitochondrial [Source:HGNC Symbol;Acc:2753]	chr8	91013633	91064320	409	0.140	1	1	8.947E-03	1.805E-02	PB-GT
B3GALNT2	beta-1,3-N-acetylgalactosaminyltransferase 2 [Source:HGNC Symbol;Acc:28596]	chr1	235613238	235667781	395	0.140	1	1	8.959E-03	1.805E-02	PB-GT
TPX2	TPX2, microtubule-associated, homolog (Xenopus laevis) [Source:HGNC Symbol;Acc:1249]	chr20	30327074	30389608	387	0.140	1	1	8.959E-03	1.805E-02	PB-GT
CSDE1	cold shock domain containing E1, RNA-binding [Source:HGNC Symbol;Acc:29905]	chr1	115259534	115301297	397	0.141	1	1	9.045E-03	1.821E-02	PB-GT
DCP1A	DCP1 decapping enzyme homolog A (S. cerevisiae) [Source:HGNC Symbol;Acc:18714]	chr3	53317448	53381637	459	0.141	1	1	9.078E-03	1.826E-02	PB-GT
GRHL2	grainyhead-like 2 (Drosophila) [Source:HGNC Symbol;Acc:2799]	chr8	102504660	102681954	1229	0.421	2	2	9.103E-03	1.830E-02	PB-GT
MAP4	microtubule-associated protein 4 [Source:HGNC Symbol;Acc:6862]	chr3	47892182	48130769	1371	0.422	2	1	9.144E-03	1.837E-02	PB-GT
C17orf67	chromosome 17 open reading frame 67 [Source:HGNC Symbol;Acc:27900]	chr17	54869274	54916134	280	0.142	1	1	9.156E-03	1.837E-02	PB-GT
GALNTL5	UDP-N-acetyl-alpha-D-galactosamine:polypeptide N-acetylgalactosaminyltransferase-like 5 [Source:HGNC Symbol;Acc:21725]	chr7	151653464	151717019	382	0.142	1	1	9.158E-03	1.837E-02	PB-GT
NOSTRIN	nitric oxide synthase trafficker [Source:HGNC Symbol;Acc:20203]	chr2	169643049	169722024	497	0.142	1	1	9.192E-03	1.842E-02	PB-GT
SERGEF	secretion regulating guanine nucleotide exchange factor [Source:HGNC Symbol;Acc:17499]	chr11	17809595	18034709	1218	0.423	2	2	9.196E-03	1.842E-02	PB-GT
AHNAK	AHNAK nucleoprotein [Source:HGNC Symbol;Acc:347]	chr11	62201016	62323707	410	0.142	1	1	9.210E-03	1.844E-02	PB-GT
PPP4R1	protein phosphatase 4, regulatory subunit 1 [Source:HGNC Symbol;Acc:9320]	chr18	9546789	9615238	595	0.143	1	1	9.239E-03	1.848E-02	PB-GT
ABCB7	ATP-binding cassette, sub-family B (MDR/TAP), member 7 [Source:HGNC Symbol;Acc:48]	chrX	74273115	74376567	856	0.143	1	1	9.253E-03	1.850E-02	PB-GT
LINC00707	long intergenic non-protein coding RNA 707 [Source:HGNC Symbol;Acc:44691]	chr10	6821560	6884868	400	0.143	1	1	9.270E-03	1.852E-02	PB-GT
LINC00469	long intergenic non-protein coding RNA 469 [Source:HGNC Symbol;Acc:26863]	chr17	71746469	71824659	282	0.143	1	1	9.281E-03	1.853E-02	PB-GT
CHEK2	checkpoint kinase 2 [Source:HGNC Symbol;Acc:16627]	chr22	29083731	29138410	286	0.143	1	1	9.286E-03	1.853E-02	PB-GT
PELI1	pellino E3 ubiquitin protein ligase 1 [Source:HGNC Symbol;Acc:8827]	chr2	64319786	64371588	500	0.143	1	1	9.298E-03	1.854E-02	PB-GT
ABHD12	abhydrolase domain containing 12 [Source:HGNC Symbol;Acc:15868]	chr20	25275379	25371619	396	0.143	1	1	9.360E-03	1.865E-02	PB-GT
FOXJ3	forkhead box J3 [Source:HGNC Symbol;Acc:29178]	chr1	42642210	42801548	1197	0.426	2	1	9.372E-03	1.866E-02	PB-GT
KIAA1324	KIAA1324 [Source:HGNC Symbol;Acc:29618]	chr1	109656301	109749401	405	0.144	1	1	9.396E-03	1.869E-02	PB-GT
SP140L	SP140 nuclear body protein-like [Source:HGNC Symbol;Acc:25105]	chr2	231191899	231268447	503	0.144	1	1	9.405E-03	1.870E-02	PB-GT
TRAPPC10	trafficking protein particle complex 10 [Source:HGNC Symbol;Acc:11868]	chr21	45432200	45526433	503	0.144	1	1	9.415E-03	1.870E-02	PB-GT
BAZ2B	bromodomain adjacent to zinc finger domain, 2B [Source:HGNC Symbol;Acc:963]	chr2	160175490	160473203	2828	0.809	3	2	9.418E-03	1.870E-02	PB-GT
OSBPL6	oxysterol binding protein-like 6 [Source:HGNC Symbol;Acc:16388]	chr2	179059208	179264160	1492	0.427	2	1	9.433E-03	1.872E-02	PB-GT
ATP6V1C1	ATPase, H+ transporting, lysosomal 42kDa, V1 subunit C1 [Source:HGNC Symbol;Acc:856]	chr8	104033291	104085279	421	0.144	1	1	9.454E-03	1.874E-02	PB-GT
NEK5	NIMA-related kinase 5 [Source:HGNC Symbol;Acc:7748]	chr13	52611093	52703214	595	0.144	1	1	9.482E-03	1.879E-02	PB-GT
ZNF782	zinc finger protein 782 [Source:HGNC Symbol;Acc:33110]	chr9	99578754	99637905	386	0.145	1	1	9.523E-03	1.885E-02	PB-GT
ZC3HAV1	zinc finger CCCH-type, antiviral 1 [Source:HGNC Symbol;Acc:23721]	chr7	138728266	138794465	389	0.145	1	1	9.527E-03	1.885E-02	PB-GT
MAP6	microtubule-associated protein 6 [Source:HGNC Symbol;Acc:6868]	chr11	75297963	75380165	418	0.145	1	1	9.555E-03	1.889E-02	PB-GT
TMBIM4	transmembrane BAX inhibitor motif containing 4 [Source:HGNC Symbol;Acc:24257]	chr12	66517709	66563852	380	0.145	1	1	9.611E-03	1.899E-02	PB-GT
GOLM1	golgi membrane protein 1 [Source:HGNC Symbol;Acc:15451]	chr9	88641061	88715088	388	0.146	1	1	9.617E-03	1.899E-02	PB-GT
P4HA3	prolyl 4-hydroxylase, alpha polypeptide III [Source:HGNC Symbol;Acc:30135]	chr11	73946846	74022702	420	0.146	1	1	9.642E-03	1.903E-02	PB-GT
NAMPT	nicotinamide phosphoribosyltransferase [Source:HGNC Symbol;Acc:30092]	chr7	105888731	105926772	393	0.146	1	1	9.667E-03	1.906E-02	PB-GT
UBA2	ubiquitin-like modifier activating enzyme 2 [Source:HGNC Symbol;Acc:30661]	chr19	34919264	34960853	277	0.146	1	1	9.696E-03	1.911E-02	PB-GT
ARNT	aryl hydrocarbon receptor nuclear translocator [Source:HGNC Symbol;Acc:700]	chr1	150782181	150849244	413	0.147	1	1	9.753E-03	1.920E-02	PB-GT
SGPL1	sphingosine-1-phosphate lyase 1 [Source:HGNC Symbol;Acc:10817]	chr10	72575717	72640930	411	0.147	1	1	9.761E-03	1.921E-02	PB-GT
ADORA2A-AS1	ADORA2A antisense RNA 1 [Source:HGNC Symbol;Acc:37122]	chr22	24825178	24891072	294	0.147	1	1	9.787E-03	1.924E-02	PB-GT
PVT1	Pvt1 oncogene (non-protein coding) [Source:HGNC Symbol;Acc:9709]	chr8	128806779	129113499	1263	0.433	2	2	9.796E-03	1.925E-02	PB-GT
ANTXR1	anthrax toxin receptor 1 [Source:HGNC Symbol;Acc:21014]	chr2	69240310	69476459	1514	0.433	2	1	9.811E-03	1.927E-02	PB-GT
LARS	leucyl-tRNA synthetase [Source:HGNC Symbol;Acc:6512]	chr5	145492601	145562223	546	0.147	1	1	9.820E-03	1.927E-02	PB-GT
CC2D2B	coiled-coil and C2 domain containing 2B [Source:HGNC Symbol;Acc:31666]	chr10	97733786	97792441	413	0.147	1	1	9.852E-03	1.930E-02	PB-GT
BRD7	bromodomain containing 7 [Source:HGNC Symbol;Acc:14310]	chr16	50347398	50402845	440	0.147	1	1	9.852E-03	1.930E-02	PB-GT
RAB14	RAB14, member RAS oncogene family [Source:HGNC Symbol;Acc:16524]	chr9	123940415	123985292	393	0.147	1	1	9.854E-03	1.930E-02	PB-GT
FAM9B	family with sequence similarity 9, member B [Source:HGNC Symbol;Acc:18404]	chrX	8992273	9132680	887	0.148	1	1	9.902E-03	1.937E-02	PB-GT
FNIP1	folliculin interacting protein 1 [Source:HGNC Symbol;Acc:29418]	chr5	130761584	131132710	3046	0.821	3	2	9.904E-03	1.937E-02	PB-GT
DDX42	DEAD (Asp-Glu-Ala-Asp) box polypeptide 42 [Source:HGNC Symbol;Acc:18676]	chr17	61850963	61896677	292	0.148	1	1	9.918E-03	1.938E-02	PB-GT
FBXL20	F-box and leucine-rich repeat protein 20 [Source:HGNC Symbol;Acc:24679]	chr17	37415384	37558776	859	0.435	2	2	9.943E-03	1.940E-02	PB-GT
RSF1	remodeling and spacing factor 1 [Source:HGNC Symbol;Acc:18118]	chr11	77371041	77532063	1254	0.435	2	2	9.944E-03	1.940E-02	PB-GT
MAP3K19	mitogen-activated protein kinase kinase kinase 19 [Source:HGNC Symbol;Acc:26249]	chr2	135722061	135805038	518	0.148	1	1	9.946E-03	1.940E-02	PB-GT
NAA16	N(alpha)-acetyltransferase 16, NatA auxiliary subunit [Source:HGNC Symbol;Acc:26164]	chr13	41885341	41951166	611	0.148	1	1	9.973E-03	1.944E-02	PB-GT
TES	testis derived transcript (3 LIM domains) [Source:HGNC Symbol;Acc:14620]	chr7	115850547	115898837	400	0.149	1	1	9.998E-03	1.947E-02	PB-GT
HMGA2	high mobility group AT-hook 2 [Source:HGNC Symbol;Acc:5009]	chr12	66217911	66360075	1139	0.436	2	1	1.000E-02	1.947E-02	PB-GT
LCOR	ligand dependent nuclear receptor corepressor [Source:HGNC Symbol;Acc:29503]	chr10	98592017	98740800	1225	0.437	2	2	1.007E-02	1.959E-02	PB-GT
MIOS	missing oocyte, meiosis regulator, homolog (Drosophila) [Source:HGNC Symbol;Acc:21905]	chr7	7606503	7648560	402	0.149	1	1	1.009E-02	1.962E-02	PB-GT
LIPA	lipase A, lysosomal acid, cholesterol esterase [Source:HGNC Symbol;Acc:6617]	chr10	90973326	91174314	1227	0.438	2	1	1.011E-02	1.965E-02	PB-GT
MGLL	monoglyceride lipase [Source:HGNC Symbol;Acc:17038]	chr3	127407909	127542051	486	0.150	1	1	1.012E-02	1.965E-02	PB-GT
TRPS1	trichorhinophalangeal syndrome I [Source:HGNC Symbol;Acc:12340]	chr8	116420724	116821899	3753	1.286	4	1	1.021E-02	1.980E-02	PB-GT
CCBP2	chemokine binding protein 2 [Source:HGNC Symbol;Acc:1565]	chr3	42846244	42949597	489	0.150	1	1	1.024E-02	1.985E-02	PB-GT
GIPC2	GIPC PDZ domain containing family, member 2 [Source:HGNC Symbol;Acc:18177]	chr1	78445226	78604133	1242	0.441	2	1	1.030E-02	1.995E-02	PB-GT
ZNF583	zinc finger protein 583 [Source:HGNC Symbol;Acc:26427]	chr19	56909335	56947404	286	0.151	1	1	1.030E-02	1.995E-02	PB-GT
YTHDF3	YTH domain family, member 3 [Source:HGNC Symbol;Acc:26465]	chr8	64081112	64125346	441	0.151	1	1	1.033E-02	1.998E-02	PB-GT
METAP1	methionyl aminopeptidase 1 [Source:HGNC Symbol;Acc:15789]	chr4	99916771	99983964	534	0.151	1	1	1.034E-02	1.999E-02	PB-GT
LNX2	ligand of numb-protein X 2 [Source:HGNC Symbol;Acc:20421]	chr13	28120050	28194541	624	0.151	1	1	1.038E-02	2.004E-02	PB-GT
EPT1	ethanolaminephosphotransferase 1 (CDP-ethanolamine-specific) [Source:HGNC Symbol;Acc:29361]	chr2	26531415	26618759	530	0.152	1	1	1.039E-02	2.004E-02	PB-GT
SNAP25-AS1	SNAP25 antisense RNA 1 [Source:HGNC Symbol;Acc:44312]	chr20	10002620	10349424	2298	0.833	3	2	1.039E-02	2.004E-02	PB-GT
TFDP2	transcription factor Dp-2 (E2F dimerization partner 2) [Source:HGNC Symbol;Acc:11751]	chr3	141663277	141868386	1439	0.443	2	1	1.041E-02	2.007E-02	PB-GT
SCRN1	secernin 1 [Source:HGNC Symbol;Acc:22192]	chr7	29959719	30029905	410	0.152	1	1	1.048E-02	2.018E-02	PB-GT
GLMN	glomulin, FKBP associated protein [Source:HGNC Symbol;Acc:14373]	chr1	92711959	92764544	429	0.152	1	1	1.048E-02	2.018E-02	PB-GT
TCEA1	transcription elongation factor A (SII), 1 [Source:HGNC Symbol;Acc:11612]	chr8	54879112	54935089	445	0.152	1	1	1.051E-02	2.021E-02	PB-GT
LHFPL2	lipoma HMGIC fusion partner-like 2 [Source:HGNC Symbol;Acc:6588]	chr5	77781038	78065844	1649	0.444	2	1	1.052E-02	2.023E-02	PB-GT
MALT1	mucosa associated lymphoid tissue lymphoma translocation gene 1 [Source:HGNC Symbol;Acc:6819]	chr18	56338618	56417371	639	0.153	1	1	1.058E-02	2.033E-02	PB-GT
GK5	glycerol kinase 5 (putative) [Source:HGNC Symbol;Acc:28635]	chr3	141882414	141944449	498	0.153	1	1	1.060E-02	2.036E-02	PB-GT
TTLL11-IT1	TTLL11 intronic transcript 1 (non-protein coding) [Source:HGNC Symbol;Acc:24214]	chr9	124646915	124725998	409	0.153	1	1	1.063E-02	2.040E-02	PB-GT
SIAE	sialic acid acetylesterase [Source:HGNC Symbol;Acc:18187]	chr11	124503009	124565603	443	0.154	1	1	1.067E-02	2.046E-02	PB-GT
SPC25	SPC25, NDC80 kinetochore complex component, homolog (S. cerevisiae) [Source:HGNC Symbol;Acc:24031]	chr2	169690642	169769881	538	0.154	1	1	1.069E-02	2.048E-02	PB-GT
ZCCHC17	zinc finger, CCHC domain containing 17 [Source:HGNC Symbol;Acc:30246]	chr1	31769842	31837783	434	0.154	1	1	1.072E-02	2.052E-02	PB-GT
TMX2-CTNND1	TMX2-CTNND1 readthrough (non-protein coding) [Source:HGNC Symbol;Acc:41992]	chr11	57480077	57559058	445	0.154	1	1	1.076E-02	2.059E-02	PB-GT
GCFC2	GC-rich sequence DNA-binding factor 2 [Source:HGNC Symbol;Acc:1317]	chr2	75879126	75938115	540	0.154	1	1	1.076E-02	2.059E-02	PB-GT
SPATA13	spermatogenesis associated 13 [Source:HGNC Symbol;Acc:23222]	chr13	24553839	24895169	1847	0.448	2	2	1.078E-02	2.060E-02	PB-GT
ROCK2	Rho-associated, coiled-coil containing protein kinase 2 [Source:HGNC Symbol;Acc:10252]	chr2	11319887	11488456	1569	0.449	2	2	1.079E-02	2.062E-02	PB-GT
CPSF6	cleavage and polyadenylation specific factor 6, 68kDa [Source:HGNC Symbol;Acc:13871]	chr12	69633317	69668138	405	0.155	1	1	1.085E-02	2.071E-02	PB-GT
FGF2	fibroblast growth factor 2 (basic) [Source:HGNC Symbol;Acc:3676]	chr4	123747863	123819391	548	0.155	1	1	1.086E-02	2.072E-02	PB-GT
STK24	serine/threonine kinase 24 [Source:HGNC Symbol;Acc:11403]	chr13	99102455	99230194	643	0.156	1	1	1.099E-02	2.093E-02	PB-GT
TMLHE-AS1	TMLHE antisense RNA 1 [Source:HGNC Symbol;Acc:44261]	chrX	154695631	154841277	937	0.156	1	1	1.099E-02	2.093E-02	PB-GT
NCEH1	neutral cholesterol ester hydrolase 1 [Source:HGNC Symbol;Acc:29260]	chr3	172348039	172429008	508	0.156	1	1	1.101E-02	2.096E-02	PB-GT
WNK2	WNK lysine deficient protein kinase 2 [Source:HGNC Symbol;Acc:14542]	chr9	95947198	96082854	417	0.156	1	1	1.103E-02	2.097E-02	PB-GT
RBL2	retinoblastoma-like 2 (p130) [Source:HGNC Symbol;Acc:9894]	chr16	53467889	53525561	467	0.156	1	1	1.103E-02	2.097E-02	PB-GT
EIF4ENIF1	eukaryotic translation initiation factor 4E nuclear import factor 1 [Source:HGNC Symbol;Acc:16687]	chr22	31835349	31892094	314	0.157	1	1	1.109E-02	2.107E-02	PB-GT
TCF7L2	transcription factor 7-like 2 (T-cell specific, HMG-box) [Source:HGNC Symbol;Acc:11641]	chr10	114710009	114927437	1271	0.454	2	2	1.111E-02	2.110E-02	PB-GT
GFPT1	glutamine--fructose-6-phosphate transaminase 1 [Source:HGNC Symbol;Acc:4241]	chr2	69546905	69614382	551	0.158	1	1	1.118E-02	2.122E-02	PB-GT
RAB11FIP2	RAB11 family interacting protein 2 (class I) [Source:HGNC Symbol;Acc:29152]	chr10	119764427	119806114	442	0.158	1	1	1.121E-02	2.125E-02	PB-GT
FAM13A	family with sequence similarity 13, member A [Source:HGNC Symbol;Acc:19367]	chr4	89647106	90032549	3009	0.852	3	2	1.122E-02	2.125E-02	PB-GT
REEP1	receptor accessory protein 1 [Source:HGNC Symbol;Acc:25786]	chr2	86441116	86565206	553	0.158	1	1	1.126E-02	2.131E-02	PB-GT
KCMF1	potassium channel modulatory factor 1 [Source:HGNC Symbol;Acc:20589]	chr2	85198216	85282560	553	0.158	1	1	1.126E-02	2.131E-02	PB-GT
LUC7L3	LUC7-like 3 (S. cerevisiae) [Source:HGNC Symbol;Acc:24309]	chr17	48796905	48833574	313	0.159	1	1	1.132E-02	2.140E-02	PB-GT
PROX1	prospero homeobox 1 [Source:HGNC Symbol;Acc:9459]	chr1	214156524	214214853	447	0.159	1	1	1.133E-02	2.142E-02	PB-GT
GUCY1A3	guanylate cyclase 1, soluble, alpha 3 [Source:HGNC Symbol;Acc:4685]	chr4	156587863	156653501	563	0.159	1	1	1.143E-02	2.159E-02	PB-GT
SKA2	spindle and kinetochore associated complex subunit 2 [Source:HGNC Symbol;Acc:28006]	chr17	57187312	57232630	315	0.160	1	1	1.145E-02	2.162E-02	PB-GT
AKR1C3	aldo-keto reductase family 1, member C3 [Source:HGNC Symbol;Acc:386]	chr10	5077546	5149878	449	0.160	1	1	1.155E-02	2.178E-02	PB-GT

table S2. Genes with significant transposon integrations in RKO *BRAF*$^{wt/-/-}$ cells after selection in low glucose medium

Gene ID	Description	Chrom	Start	End	# TTAA sites	Expected	Observed	Libraries	*P*	FDR	Transposon
TMEM71	transmembrane protein 71 [Source:HGNC Symbol;Acc:26572]	chr8	133697253	133772958	468	0.160	1	1	1.156E-02	2.179E-02	PB-GT
CROT	carnitine O-octanoyltransferase [Source:HGNC Symbol;Acc:2366]	chr7	86974997	87029111	432	0.160	1	1	1.157E-02	2.179E-02	PB-GT
KRIT1	KRIT1, ankyrin repeat containing [Source:HGNC Symbol;Acc:1573]	chr7	91828283	91875480	433	0.161	1	1	1.162E-02	2.187E-02	PB-GT
DDX46	DEAD (Asp-Glu-Ala-Asp) box polypeptide 46 [Source:HGNC Symbol;Acc:18681]	chr5	134094469	134190823	597	0.161	1	1	1.163E-02	2.189E-02	PB-GT
C9orf41	chromosome 9 open reading frame 41 [Source:HGNC Symbol;Acc:23435]	chr9	77595936	77643339	430	0.161	1	1	1.169E-02	2.197E-02	PB-GT
GPR126	G protein-coupled receptor 126 [Source:HGNC Symbol;Acc:13841]	chr6	142622991	142767403	1346	0.462	2	2	1.169E-02	2.197E-02	PB-GT
AP1B1	adaptor-related protein complex 1, beta 1 subunit [Source:HGNC Symbol;Acc:554]	chr22	29723669	29819168	323	0.161	1	1	1.170E-02	2.197E-02	PB-GT
ZNF571	zinc finger protein 571 [Source:HGNC Symbol;Acc:25000]	chr19	38045684	38085673	306	0.161	1	1	1.171E-02	2.197E-02	PB-GT
HRH1	histamine receptor H1 [Source:HGNC Symbol;Acc:5182]	chr3	11178779	11305243	525	0.162	1	1	1.172E-02	2.197E-02	PB-GT
APOLD1	apolipoprotein L domain containing 1 [Source:HGNC Symbol;Acc:25268]	chr12	12878865	12944400	422	0.162	1	1	1.173E-02	2.197E-02	PB-GT
PTPN1	protein tyrosine phosphatase, non-receptor type 1 [Source:HGNC Symbol;Acc:9642]	chr20	49126891	49201299	446	0.162	1	1	1.173E-02	2.197E-02	PB-GT
LPIN2	lipin 2 [Source:HGNC Symbol;Acc:14450]	chr18	2916992	3013313	676	0.162	1	1	1.177E-02	2.203E-02	PB-GT
C12orf45	chromosome 12 open reading frame 45 [Source:HGNC Symbol;Acc:28628]	chr12	105380088	105443515	423	0.162	1	1	1.178E-02	2.203E-02	PB-GT
SV2C	synaptic vesicle glycoprotein 2C [Source:HGNC Symbol;Acc:30670]	chr5	75378997	75649764	1726	0.465	2	2	1.188E-02	2.220E-02	PB-GT
CRISPLD1	cysteine-rich secretory protein LCCL domain containing 1 [Source:HGNC Symbol;Acc:18206]	chr8	75896750	75946793	475	0.163	1	1	1.189E-02	2.220E-02	PB-GT
SCAF4	SR-related CTD-associated factor 4 [Source:HGNC Symbol;Acc:19304]	chr21	33043346	33104388	569	0.163	1	1	1.190E-02	2.221E-02	PB-GT
GDI2	GDP dissociation inhibitor 2 [Source:HGNC Symbol;Acc:4227]	chr10	5807186	5884095	457	0.163	1	1	1.194E-02	2.227E-02	PB-GT
ADAM10	ADAM metallopeptidase domain 10 [Source:HGNC Symbol;Acc:188]	chr15	58887403	59042177	1341	0.466	2	1	1.197E-02	2.230E-02	PB-GT
BRCA2	breast cancer 2, early onset [Source:HGNC Symbol;Acc:1101]	chr13	32889611	32973805	674	0.164	1	1	1.201E-02	2.238E-02	PB-GT
EFCAB13	EF-hand calcium binding domain 13 [Source:HGNC Symbol;Acc:26864]	chr17	45400656	45518678	923	0.468	2	1	1.205E-02	2.241E-02	PB-GT
TBL1XR1	transducin (beta)-like 1 X-linked receptor 1 [Source:HGNC Symbol;Acc:29529]	chr3	176737143	176915261	1520	0.468	2	2	1.205E-02	2.241E-02	PB-GT
PRKAR2A	protein kinase, cAMP-dependent, regulatory, type II, alpha [Source:HGNC Symbol;Acc:9391]	chr3	48782030	48885279	533	0.164	1	1	1.206E-02	2.242E-02	PB-GT
UCK2	uridine-cytidine kinase 2 [Source:HGNC Symbol;Acc:12562]	chr1	165796768	165880855	462	0.164	1	1	1.207E-02	2.242E-02	PB-GT
RPRD1A	regulation of nuclear pre-mRNA domain containing 1A [Source:HGNC Symbol;Acc:25560]	chr18	33564350	33647539	685	0.164	1	1	1.207E-02	2.242E-02	PB-GT
WDR37	WD repeat domain 37 [Source:HGNC Symbol;Acc:31406]	chr10	1095478	1178237	460	0.164	1	1	1.209E-02	2.243E-02	PB-GT
CDS1	CDP-diacylglycerol synthase (phosphatidate cytidylyltransferase) 1 [Source:HGNC Symbol;Acc:1800]	chr4	85504132	85572491	580	0.164	1	1	1.209E-02	2.243E-02	PB-GT
HKR1	HKR1, GLI-Kruppel zinc finger family member [Source:HGNC Symbol;Acc:4928]	chr19	37803739	37860267	312	0.165	1	1	1.215E-02	2.252E-02	PB-GT
RFX7	regulatory factor X, 7 [Source:HGNC Symbol;Acc:25777]	chr15	56379478	56535483	1354	0.471	2	2	1.228E-02	2.272E-02	PB-GT
DIXDC1	DIX domain containing 1 [Source:HGNC Symbol;Acc:23695]	chr11	111797868	111893308	477	0.166	1	1	1.228E-02	2.272E-02	PB-GT
LINC00395	long intergenic non-protein coding RNA 395 [Source:HGNC Symbol;Acc:42723]	chr13	64241814	64312151	684	0.166	1	1	1.235E-02	2.285E-02	PB-GT
GPSM2	G-protein signaling modulator 2 [Source:HGNC Symbol;Acc:29501]	chr1	109417972	109473044	470	0.167	1	1	1.246E-02	2.304E-02	PB-GT
MORF4L1	mortality factor 4 like 1 [Source:HGNC Symbol;Acc:16989]	chr15	79102829	79190475	480	0.167	1	1	1.247E-02	2.304E-02	PB-GT
YWHAE	tyrosine 3-monooxygenase/tryptophan 5-monooxygenase activation protein, epsilon polypeptide [Source:HGNC Symbol;Acc:12	chr17	1247566	1303672	331	0.168	1	1	1.258E-02	2.322E-02	PB-GT
NCOR2	nuclear receptor corepressor 2 [Source:HGNC Symbol;Acc:7673]	chr12	124808961	125052135	440	0.168	1	1	1.269E-02	2.340E-02	PB-GT
DCUN1D4	DCN1, defective in cullin neddylation 1, domain containing 4 (S. cerevisiae) [Source:HGNC Symbol;Acc:28998]	chr4	52709166	52783003	595	0.168	1	1	1.269E-02	2.340E-02	PB-GT
LTN1	listerin E3 ubiquitin protein ligase 1 [Source:HGNC Symbol;Acc:13082]	chr21	30300466	30365277	589	0.169	1	1	1.270E-02	2.340E-02	PB-GT
COPB1	coatomer protein complex, subunit beta 1 [Source:HGNC Symbol;Acc:2231]	chr11	14464986	14521573	486	0.169	1	1	1.272E-02	2.341E-02	PB-GT
ANP32AP1	acidic (leucine-rich) nuclear phosphoprotein 32 family, member A pseudogene 1 [Source:HGNC Symbol;Acc:42949]	chr15	35474000	35530398	485	0.169	1	1	1.272E-02	2.341E-02	PB-GT
C14orf159	chromosome 14 open reading frame 159 [Source:HGNC Symbol;Acc:20498]	chr14	91526677	91691976	623	0.169	1	1	1.276E-02	2.346E-02	PB-GT
IWS1	IWS1 homolog (S. cerevisiae) [Source:HGNC Symbol;Acc:25467]	chr2	128193783	128284462	591	0.169	1	1	1.277E-02	2.346E-02	PB-GT
FOXP1	forkhead box P1 [Source:HGNC Symbol;Acc:3823]	chr3	71003844	71633140	4425	1.361	4	1	1.278E-02	2.346E-02	PB-GT
NUP37	nucleoporin 37kDa [Source:HGNC Symbol;Acc:29929]	chr12	102467967	102513902	442	0.169	1	1	1.280E-02	2.349E-02	PB-GT
SGMS1	sphingomyelin synthase 1 [Source:HGNC Symbol;Acc:29799]	chr10	52065360	52384795	2485	0.887	3	2	1.282E-02	2.351E-02	PB-GT
STXBP6	syntaxin binding protein 6 (amisyn) [Source:HGNC Symbol;Acc:19666]	chr14	25278862	25519503	1766	0.479	2	2	1.284E-02	2.354E-02	PB-GT
HSPA4L	heat shock 70kDa protein 4-like [Source:HGNC Symbol;Acc:17041]	chr4	128702976	128755226	599	0.170	1	1	1.285E-02	2.354E-02	PB-GT
PHTF1	putative homeodomain transcription factor 1 [Source:HGNC Symbol;Acc:8939]	chr1	114239453	114302111	478	0.170	1	1	1.287E-02	2.355E-02	PB-GT
PTPN21	protein tyrosine phosphatase, non-receptor type 21 [Source:HGNC Symbol;Acc:9651]	chr14	88932122	89021077	626	0.170	1	1	1.288E-02	2.356E-02	PB-GT
SLC35A3	solute carrier family 35 (UDP-N-acetylglucosamine (UDP-GlcNAc) transporter), member A3 [Source:HGNC Symbol;Acc:11023]	chr1	100435345	100492535	479	0.170	1	1	1.292E-02	2.359E-02	PB-GT
PCBD2	pterin-4 alpha-carbinolamine dehydratase/dimerization cofactor of hepatocyte nuclear factor 1 alpha (TCF1) 2 [Source:HGNC S	chr5	134240596	134343649	631	0.170	1	1	1.292E-02	2.359E-02	PB-GT
HMG20A	high mobility group 20A [Source:HGNC Symbol;Acc:5001]	chr15	77712754	77777949	489	0.170	1	1	1.292E-02	2.359E-02	PB-GT
COMMD1	copper metabolism (Murr1) domain containing 1 [Source:HGNC Symbol;Acc:23024]	chr2	62115859	62374382	1680	0.480	2	1	1.295E-02	2.361E-02	PB-GT
LRP6	low density lipoprotein receptor-related protein 6 [Source:HGNC Symbol;Acc:6698]	chr12	12268959	12419946	1255	0.480	2	1	1.295E-02	2.361E-02	PB-GT
CAMSAP1	calmodulin regulated spectrin-associated protein 1 [Source:HGNC Symbol;Acc:19946]	chr9	138700333	138799074	454	0.170	1	1	1.295E-02	2.361E-02	PB-GT
APLP2	amyloid beta (A4) precursor-like protein 2 [Source:HGNC Symbol;Acc:598]	chr11	129939732	130014699	491	0.170	1	1	1.297E-02	2.361E-02	PB-GT
CCAR1	cell division cycle and apoptosis regulator 1 [Source:HGNC Symbol;Acc:24236]	chr10	70480769	70552134	478	0.171	1	1	1.300E-02	2.366E-02	PB-GT
TPTE2P5	transmembrane phosphoinositide 3-phosphatase and tensin homolog 2 pseudogene 5 [Source:HGNC Symbol;Acc:42356]	chr13	41396432	41495890	703	0.171	1	1	1.301E-02	2.366E-02	PB-GT
MYSM1	Myb-like, SWIRM and MPN domains 1 [Source:HGNC Symbol;Acc:29401]	chr1	59120411	59165764	482	0.171	1	1	1.307E-02	2.375E-02	PB-GT
RSBN1	round spermatid basic protein 1 [Source:HGNC Symbol;Acc:25642]	chr1	114304454	114355098	482	0.171	1	1	1.307E-02	2.375E-02	PB-GT
WWC2	WW and C2 domain containing 2 [Source:HGNC Symbol;Acc:24148]	chr4	184020446	184241930	1705	0.483	2	1	1.311E-02	2.381E-02	PB-GT
ALDH1L2	aldehyde dehydrogenase 1 family, member L2 [Source:HGNC Symbol;Acc:26777]	chr12	105413568	105478355	448	0.172	1	1	1.313E-02	2.382E-02	PB-GT
ATF7IP	activating transcription factor 7 interacting protein [Source:HGNC Symbol;Acc:20092]	chr12	14518610	14651697	1262	0.483	2	2	1.314E-02	2.383E-02	PB-GT
ZNF268	zinc finger protein 268 [Source:HGNC Symbol;Acc:13061]	chr12	133707570	133783698	449	0.172	1	1	1.319E-02	2.389E-02	PB-GT
NT5C3A	5'-nucleotidase, cytosolic IIIA [Source:HGNC Symbol;Acc:17820]	chr7	33053742	33102409	463	0.172	1	1	1.319E-02	2.389E-02	PB-GT
SLC4A5	solute carrier family 4, sodium bicarbonate cotransporter, member 5 [Source:HGNC Symbol;Acc:18168]	chr2	74443369	74570541	602	0.172	1	1	1.322E-02	2.393E-02	PB-GT
ELK3	ELK3, ETS-domain protein (SRF accessory protein 2) [Source:HGNC Symbol;Acc:3325]	chr12	96588160	96663613	450	0.172	1	1	1.324E-02	2.395E-02	PB-GT
KIF11	kinesin family member 11 [Source:HGNC Symbol;Acc:6388]	chr10	94353043	94415150	483	0.172	1	1	1.325E-02	2.396E-02	PB-GT
SP3	Sp3 transcription factor [Source:HGNC Symbol;Acc:11208]	chr2	174771187	174830430	605	0.173	1	1	1.335E-02	2.411E-02	PB-GT
PPP3CA	protein phosphatase 3, catalytic subunit, alpha isozyme [Source:HGNC Symbol;Acc:9314]	chr4	101944566	102269435	3174	0.899	3	2	1.339E-02	2.417E-02	PB-GT
PKIG	protein kinase (cAMP-dependent, catalytic) inhibitor gamma [Source:HGNC Symbol;Acc:9019]	chr20	43160426	43252888	479	0.174	1	1	1.343E-02	2.422E-02	PB-GT
TFB1M	transcription factor B1, mitochondrial [Source:HGNC Symbol;Acc:17037]	chr6	155578643	155635627	506	0.174	1	1	1.346E-02	2.427E-02	PB-GT
KIT	v-kit Hardy-Zuckerman 4 feline sarcoma viral oncogene homolog [Source:HGNC Symbol;Acc:6342]	chr4	55524085	55606881	614	0.174	1	1	1.347E-02	2.427E-02	PB-GT
ZNF81	zinc finger protein 81 [Source:HGNC Symbol;Acc:13156]	chrX	47696301	47861960	1044	0.174	1	1	1.348E-02	2.428E-02	PB-GT
CWC22	CWC22 spliceosome-associated protein homolog (S. cerevisiae) [Source:HGNC Symbol;Acc:29322]	chr2	180809603	180871840	609	0.174	1	1	1.351E-02	2.432E-02	PB-GT
SH3GLB1	SH3-domain GRB2-like endophilin B1 [Source:HGNC Symbol;Acc:10833]	chr1	87170259	87213867	491	0.174	1	1	1.354E-02	2.434E-02	PB-GT
BDNF	brain-derived neurotrophic factor [Source:HGNC Symbol;Acc:1033]	chr11	27676440	27743605	503	0.175	1	1	1.357E-02	2.439E-02	PB-GT
OSBPL3	oxysterol binding protein-like 3 [Source:HGNC Symbol;Acc:16370]	chr7	24836158	25021253	1319	0.490	2	1	1.363E-02	2.447E-02	PB-GT
ARAP2	ArfGAP with RhoGAP domain, ankyrin repeat and PH domain 2 [Source:HGNC Symbol;Acc:16924]	chr4	36067620	36246131	1731	0.490	2	1	1.365E-02	2.450E-02	PB-GT
RAP1B	RAP1B, member of RAS oncogene family [Source:HGNC Symbol;Acc:9857]	chr12	69004619	69054372	458	0.175	1	1	1.369E-02	2.454E-02	PB-GT
OVOL2	ovo-like 2 (Drosophila) [Source:HGNC Symbol;Acc:15804]	chr20	17937623	18039832	484	0.175	1	1	1.369E-02	2.454E-02	PB-GT
ZC2HC1A	zinc finger, C2HC-type containing 1A [Source:HGNC Symbol;Acc:24277]	chr8	79578282	79629935	512	0.175	1	1	1.370E-02	2.454E-02	PB-GT
MAP1B	microtubule-associated protein 1B [Source:HGNC Symbol;Acc:6836]	chr5	71403061	71505395	651	0.175	1	1	1.370E-02	2.454E-02	PB-GT
RASGRP1	RAS guanyl releasing protein 1 (calcium and DAG-regulated) [Source:HGNC Symbol;Acc:9878]	chr15	38780304	38857776	505	0.176	1	1	1.373E-02	2.456E-02	PB-GT
DNAJB6	DnaJ (Hsp40) homolog, subfamily B, member 6 [Source:HGNC Symbol;Acc:14888]	chr7	157128075	157210133	473	0.176	1	1	1.373E-02	2.456E-02	PB-GT
AZIN1	antizyme inhibitor 1 [Source:HGNC Symbol;Acc:16432]	chr8	103838585	103906092	513	0.176	1	1	1.375E-02	2.456E-02	PB-GT
TAPT1-AS1	TAPT1 antisense RNA 1 (head to head) [Source:HGNC Symbol;Acc:26832]	chr4	16228286	16321763	615	0.176	1	1	1.376E-02	2.456E-02	PB-GT
SMEK2	SMEK homolog 2, suppressor of mek1 (Dictyostelium) [Source:HGNC Symbol;Acc:29267]	chr2	55774428	55846015	615	0.176	1	1	1.377E-02	2.456E-02	PB-GT
PUS10	pseudouridylate synthase 10 [Source:HGNC Symbol;Acc:26505]	chr2	61167357	61245394	615	0.176	1	1	1.377E-02	2.456E-02	PB-GT
MDM1	Mdm1 nuclear protein homolog (mouse) [Source:HGNC Symbol;Acc:29917]	chr12	68666223	68726161	460	0.176	1	1	1.380E-02	2.461E-02	PB-GT
CPEB4	cytoplasmic polyadenylation element binding protein 4 [Source:HGNC Symbol;Acc:21747]	chr5	173315283	173388979	654	0.176	1	1	1.382E-02	2.462E-02	PB-GT
IPO7	importin 7 [Source:HGNC Symbol;Acc:9852]	chr11	9406169	9469673	508	0.176	1	1	1.383E-02	2.462E-02	PB-GT
GNB1	guanine nucleotide binding protein (G protein), beta polypeptide 1 [Source:HGNC Symbol;Acc:4396]	chr1	1716729	1822495	498	0.177	1	1	1.390E-02	2.474E-02	PB-GT
C12orf50	chromosome 12 open reading frame 50 [Source:HGNC Symbol;Acc:26665]	chr12	88373816	88427814	462	0.177	1	1	1.392E-02	2.475E-02	PB-GT
IREB2	iron-responsive element binding protein 2 [Source:HGNC Symbol;Acc:6115]	chr15	78729773	78793798	509	0.177	1	1	1.394E-02	2.476E-02	PB-GT
TRMT11	tRNA methyltransferase 11 homolog (S. cerevisiae) [Source:HGNC Symbol;Acc:21080]	chr6	126307576	126360937	516	0.177	1	1	1.397E-02	2.481E-02	PB-GT
LTBP1	latent transforming growth factor beta binding protein 1 [Source:HGNC Symbol;Acc:6714]	chr2	33172039	33624576	3186	0.911	3	1	1.401E-02	2.487E-02	PB-GT
DIAPH1	diaphanous homolog 1 (Drosophila) [Source:HGNC Symbol;Acc:2876]	chr5	140894583	140998622	661	0.178	1	1	1.410E-02	2.502E-02	PB-GT
PFN2	profilin 2 [Source:HGNC Symbol;Acc:8882]	chr3	149682691	149768575	580	0.178	1	1	1.414E-02	2.507E-02	PB-GT
SLC9A2	solute carrier family 9, subfamily A (NHE2, cation proton antiporter 2), member 2 [Source:HGNC Symbol;Acc:11072]	chr2	103236166	103327777	624	0.178	1	1	1.415E-02	2.507E-02	PB-GT
KATNAL2	katanin p60 subunit A-like 2 [Source:HGNC Symbol;Acc:25387]	chr18	44497455	44627658	746	0.179	1	1	1.418E-02	2.511E-02	PB-GT
DEPDC4	DEP domain containing 4 [Source:HGNC Symbol;Acc:22952]	chr12	100597447	100660857	468	0.179	1	1	1.426E-02	2.523E-02	PB-GT
FAM171B	family with sequence similarity 171, member B [Source:HGNC Symbol;Acc:29412]	chr2	187558698	187630685	628	0.180	1	1	1.432E-02	2.531E-02	PB-GT
FAM49A	family with sequence similarity 49, member A [Source:HGNC Symbol;Acc:25373]	chr2	16730727	16847599	628	0.180	1	1	1.432E-02	2.531E-02	PB-GT
SPECC1	sperm antigen with calponin homology and coiled-coil domains 1 [Source:HGNC Symbol;Acc:30615]	chr17	19912657	20222339	1812	0.918	3	1	1.436E-02	2.535E-02	PB-GT
MYH11	myosin, heavy chain 11, smooth muscle [Source:HGNC Symbol;Acc:7569]	chr16	15796992	15950890	537	0.180	1	1	1.436E-02	2.535E-02	PB-GT
KDM1A	lysine (K)-specific demethylase 1A [Source:HGNC Symbol;Acc:29079]	chr1	23345941	23410182	507	0.180	1	1	1.438E-02	2.536E-02	PB-GT
CDC42EP3	CDC42 effector protein (Rho GTPase binding) 3 [Source:HGNC Symbol;Acc:16943]	chr2	37869032	37965611	630	0.180	1	1	1.441E-02	2.540E-02	PB-GT
MAK	male germ cell-associated kinase [Source:HGNC Symbol;Acc:6816]	chr6	10762956	10838764	526	0.181	1	1	1.448E-02	2.552E-02	PB-GT
PCNXL4	pecanex-like 4 (Drosophila) [Source:HGNC Symbol;Acc:20349]	chr14	60558629	60635851	667	0.181	1	1	1.452E-02	2.556E-02	PB-GT
BRD4	bromodomain containing 4 [Source:HGNC Symbol;Acc:13575]	chr19	15347647	15443356	343	0.181	1	1	1.453E-02	2.557E-02	PB-GT
BAIAP2L1	BAI1-associated protein 2-like 1 [Source:HGNC Symbol;Acc:21649]	chr7	97920963	98030380	489	0.182	1	1	1.462E-02	2.571E-02	PB-GT
SNX31	sorting nexin 31 [Source:HGNC Symbol;Acc:28605]	chr8	101585116	101675643	531	0.182	1	1	1.467E-02	2.578E-02	PB-GT
VSTM4	V-set and transmembrane domain containing 4 [Source:HGNC Symbol;Acc:26470]	chr10	50226490	50323554	510	0.182	1	1	1.469E-02	2.579E-02	PB-GT
FAT4	FAT tumor suppressor homolog 4 (Drosophila) [Source:HGNC Symbol;Acc:23109]	chr4	126237554	126414087	1780	0.504	2	1	1.469E-02	2.579E-02	PB-GT
CBFB	core-binding factor, beta subunit [Source:HGNC Symbol;Acc:1539]	chr16	67063019	67134961	544	0.182	1	1	1.472E-02	2.581E-02	PB-GT
INTS8	integrator complex subunit 8 [Source:HGNC Symbol;Acc:26048]	chr8	95825539	95893974	532	0.182	1	1	1.472E-02	2.581E-02	PB-GT
ZNF100	zinc finger protein 100 [Source:HGNC Symbol;Acc:12880]	chr19	21905568	21950430	346	0.183	1	1	1.477E-02	2.587E-02	PB-GT
TEX15	testis expressed 15 [Source:HGNC Symbol;Acc:11738]	chr8	30689060	30748122	533	0.183	1	1	1.478E-02	2.587E-02	PB-GT
GMPS	guanine monphosphate synthetase [Source:HGNC Symbol;Acc:4378]	chr3	155588325	155658457	594	0.183	1	1	1.479E-02	2.587E-02	PB-GT
PIBF1	progesterone immunomodulatory binding factor 1 [Source:HGNC Symbol;Acc:23352]	chr13	73356197	73590591	2082	0.505	2	2	1.481E-02	2.587E-02	PB-GT
LINC00687	long intergenic non-protein coding RNA 687 [Source:HGNC Symbol;Acc:16194]	chr20	11781111	11851363	505	0.183	1	1	1.483E-02	2.587E-02	PB-GT
ZNF131	zinc finger protein 131 [Source:HGNC Symbol;Acc:12915]	chr5	43065278	43192123	679	0.183	1	1	1.483E-02	2.587E-02	PB-GT
KLF7	Kruppel-like factor 7 (ubiquitous) [Source:HGNC Symbol;Acc:6350]	chr2	207938861	208031991	640	0.183	1	1	1.484E-02	2.587E-02	PB-GT
CREB1	cAMP responsive element binding protein 1 [Source:HGNC Symbol;Acc:2345]	chr2	208394461	208468155	640	0.183	1	1	1.484E-02	2.587E-02	PB-GT
AFTPH	aftiphilin [Source:HGNC Symbol;Acc:25951]	chr2	64751465	64820139	640	0.183	1	1	1.484E-02	2.587E-02	PB-GT
PLAC1	placenta-specific 1 [Source:HGNC Symbol;Acc:9044]	chrX	133699868	133898352	1101	0.183	1	1	1.490E-02	2.596E-02	PB-GT
SMARCA2	SWI/SNF related, matrix associated, actin dependent regulator of chromatin, subfamily a, member 2 [Source:HGNC Symbol;Ac	chr9	2015342	2193624	1351	0.507	2	2	1.490E-02	2.596E-02	PB-GT
SMAD3	SMAD family member 3 [Source:HGNC Symbol;Acc:6769]	chr15	67356101	67487533	528	0.184	1	1	1.493E-02	2.597E-02	PB-GT
SLC7A2	solute carrier family 7 (cationic amino acid transporter, y+ system), member 2 [Source:HGNC Symbol;Acc:11060]	chr8	17354597	17428082	536	0.184	1	1	1.493E-02	2.597E-02	PB-GT
BCAR3	breast cancer anti-estrogen resistance 3 [Source:HGNC Symbol;Acc:973]	chr1	94027347	94312706	1430	0.508	2	2	1.497E-02	2.601E-02	PB-GT
PHLPP2	PH domain and leucine rich repeat protein phosphatase 2 [Source:HGNC Symbol;Acc:29149]	chr16	71671738	71758604	549	0.184	1	1	1.497E-02	2.601E-02	PB-GT
TTC26	tetratricopeptide repeat domain 26 [Source:HGNC Symbol;Acc:21882]	chr7	138818490	138876732	496	0.184	1	1	1.502E-02	2.607E-02	PB-GT
GXYLT2	glucoside xylosyltransferase 2 [Source:HGNC Symbol;Acc:33383]	chr3	72937224	73047289	599	0.184	1	1	1.503E-02	2.608E-02	PB-GT
KCNS3	potassium voltage-gated channel, delayed-rectifier, subfamily S, member 3 [Source:HGNC Symbol;Acc:6302]	chr2	18059114	18542882	3258	0.932	3	1	1.508E-02	2.615E-02	PB-GT
PIK3R4	phosphoinositide-3-kinase, regulatory subunit 4 [Source:HGNC Symbol;Acc:8982]	chr3	130397779	130465673	603	0.185	1	1	1.522E-02	2.637E-02	PB-GT
TMEM57	transmembrane protein 57 [Source:HGNC Symbol;Acc:25572]	chr1	25757388	25826700	523	0.186	1	1	1.524E-02	2.638E-02	PB-GT
HERPUD2	HERPUD family member 2 [Source:HGNC Symbol;Acc:21915]	chr7	35672269	35735181	500	0.186	1	1	1.524E-02	2.638E-02	PB-GT
CDC14A	cell division cycle 14A [Source:HGNC Symbol;Acc:1718]	chr1	100810584	100985833	1440	0.511	2	2	1.525E-02	2.638E-02	PB-GT
GNA13	guanine nucleotide binding protein (G protein), alpha 13 [Source:HGNC Symbol;Acc:4381]	chr17	63006833	63052957	367	0.186	1	1	1.528E-02	2.642E-02	PB-GT

table S2. Genes with significant transposon integrations in RKO *BRAF*$^{wt/-/-}$ cells after selection in low glucose medium

Gene ID	Description	Chrom	Start	End	# TTAA sites	Expected	Observed	Libraries	*P*	FDR	Transposon
ATP6AP1L	ATPase, H+ transporting, lysosomal accessory protein 1-like [Source:HGNC Symbol;Acc:28091]	chr5	81575281	81682796	691	0.186	1	1	1.533E-02	2.649E-02	PB-GT
MTMR8	myotubularin related protein 8 [Source:HGNC Symbol;Acc:16825]	chrX	63444187	63615333	1118	0.186	1	1	1.534E-02	2.649E-02	PB-GT
NPTN	neuroplastin [Source:HGNC Symbol;Acc:17867]	chr15	73852355	73926475	536	0.186	1	1	1.536E-02	2.649E-02	PB-GT
ASH1L	ash1 (absent, small, or homeotic)-like (Drosophila) [Source:HGNC Symbol;Acc:19088]	chr1	155305059	155532598	1444	0.513	2	2	1.536E-02	2.649E-02	PB-GT
ERGIC2	ERGIC and golgi 2 [Source:HGNC Symbol;Acc:30208]	chr12	29490285	29534122	487	0.186	1	1	1.537E-02	2.649E-02	PB-GT
CTTNBP2	cortactin binding protein 2 [Source:HGNC Symbol;Acc:15679]	chr7	117350705	117514193	1386	0.515	2	1	1.552E-02	2.674E-02	PB-GT
SYCP2L	synaptonemal complex protein 2-like [Source:HGNC Symbol;Acc:21537]	chr6	10748027	10979553	1501	0.516	2	1	1.560E-02	2.685E-02	PB-GT
AP3S1	adaptor-related protein complex 3, sigma 1 subunit [Source:HGNC Symbol;Acc:2013]	chr5	115177178	115249778	700	0.189	1	1	1.571E-02	2.701E-02	PB-GT
ANKRD18CP	ankyrin repeat domain 18C, pseudogene [Source:HGNC Symbol;Acc:43601]	chr9	99918852	99983517	503	0.189	1	1	1.571E-02	2.701E-02	PB-GT
MYH14	myosin, heavy chain 14, non-muscle [Source:HGNC Symbol;Acc:23212]	chr19	50691443	50813802	358	0.189	1	1	1.575E-02	2.706E-02	PB-GT
ZNF106	zinc finger protein 106 [Source:HGNC Symbol;Acc:12886]	chr15	42705021	42783321	545	0.190	1	1	1.585E-02	2.721E-02	PB-GT
PAPOLA	poly(A) polymerase alpha [Source:HGNC Symbol;Acc:14981]	chr14	96967770	97033448	700	0.190	1	1	1.589E-02	2.726E-02	PB-GT
SH2D4B	SH2 domain containing 4B [Source:HGNC Symbol;Acc:31440]	chr10	82297658	82406316	532	0.190	1	1	1.590E-02	2.726E-02	PB-GT
AK4	adenylate kinase 4 [Source:HGNC Symbol;Acc:363]	chr1	65613232	65697828	535	0.190	1	1	1.591E-02	2.726E-02	PB-GT
JUP	junction plakoglobin [Source:HGNC Symbol;Acc:6207]	chr17	39775692	39943183	375	0.190	1	1	1.591E-02	2.726E-02	PB-GT
STAG1	stromal antigen 1 [Source:HGNC Symbol;Acc:11354]	chr3	136055077	136471220	3082	0.948	3	2	1.597E-02	2.734E-02	PB-GT
CLINT1	clathrin interactor 1 [Source:HGNC Symbol;Acc:23186]	chr5	157212751	157286183	707	0.191	1	1	1.600E-02	2.736E-02	PB-GT
DHX15	DEAH (Asp-Glu-Ala-His) box polypeptide 15 [Source:HGNC Symbol;Acc:2738]	chr4	24519064	24586173	673	0.191	1	1	1.600E-02	2.736E-02	PB-GT
LINC00159	long intergenic non-protein coding RNA 159 [Source:HGNC Symbol;Acc:1285]	chr21	33452629	33570125	666	0.191	1	1	1.601E-02	2.736E-02	PB-GT
C7orf49	chromosome 7 open reading frame 49 [Source:HGNC Symbol;Acc:22432]	chr7	134777115	134855547	514	0.191	1	1	1.606E-02	2.742E-02	PB-GT
GANC	glucosidase, alpha; neutral C [Source:HGNC Symbol;Acc:4139]	chr15	42565431	42645864	549	0.191	1	1	1.606E-02	2.742E-02	PB-GT
HSF2BP	heat shock transcription factor 2 binding protein [Source:HGNC Symbol;Acc:5226]	chr21	44949072	45079374	670	0.192	1	1	1.619E-02	2.762E-02	PB-GT
FOLH1B	folate hydrolase 1B [Source:HGNC Symbol;Acc:13636]	chr11	89371683	89431886	553	0.192	1	1	1.622E-02	2.765E-02	PB-GT
ZPLD1	zona pellucida-like domain containing 1 [Source:HGNC Symbol;Acc:27022]	chr3	101818088	102198685	3103	0.955	3	2	1.633E-02	2.782E-02	PB-GT
TNRC6B	trinucleotide repeat containing 6B [Source:HGNC Symbol;Acc:29190]	chr22	40440821	40731811	1912	0.955	3	2	1.637E-02	2.787E-02	PB-GT
MED13	mediator complex subunit 13 [Source:HGNC Symbol;Acc:22474]	chr17	60019966	60142643	1037	0.525	2	1	1.638E-02	2.788E-02	PB-GT
SPATA7	spermatogenesis associated 7 [Source:HGNC Symbol;Acc:20423]	chr14	88851268	88936694	712	0.193	1	1	1.641E-02	2.791E-02	PB-GT
FBXO42	F-box protein 42 [Source:HGNC Symbol;Acc:29249]	chr1	16573334	16678949	545	0.193	1	1	1.647E-02	2.799E-02	PB-GT
NCK1	NCK adaptor protein 1 [Source:HGNC Symbol;Acc:7664]	chr3	136581050	136668665	629	0.193	1	1	1.647E-02	2.799E-02	PB-GT
PPM1B	protein phosphatase, Mg2+/Mn2+ dependent, 1B [Source:HGNC Symbol;Acc:9276]	chr2	44395108	44471523	679	0.194	1	1	1.658E-02	2.816E-02	PB-GT
NEDD4L	neural precursor cell expressed, developmentally down-regulated 4-like, E3 ubiquitin protein ligase [Source:HGNC Symbol;Acc	chr18	55711599	56068772	2206	0.528	2	1	1.663E-02	2.822E-02	PB-GT
PRPF40A	PRP40 pre-mRNA processing factor 40 homolog A (S. cerevisiae) [Source:HGNC Symbol;Acc:16463]	chr2	153508107	153574511	682	0.195	1	1	1.672E-02	2.835E-02	PB-GT
KLHL20	kelch-like family member 20 [Source:HGNC Symbol;Acc:25056]	chr1	173684080	173755840	550	0.195	1	1	1.675E-02	2.840E-02	PB-GT
WDR25	WD repeat domain 25 [Source:HGNC Symbol;Acc:21064]	chr14	100842755	100996640	724	0.196	1	1	1.693E-02	2.867E-02	PB-GT
PAPD4	PAP associated domain containing 4 [Source:HGNC Symbol;Acc:26776]	chr5	78907943	78982471	729	0.196	1	1	1.695E-02	2.867E-02	PB-GT
MRPL1	mitochondrial ribosomal protein L1 [Source:HGNC Symbol;Acc:14275]	chr4	78783674	78873944	694	0.196	1	1	1.695E-02	2.867E-02	PB-GT
SLX4IP	SLX4 interacting protein [Source:HGNC Symbol;Acc:16225]	chr20	10415951	10617477	1469	0.532	2	2	1.695E-02	2.867E-02	PB-GT
SORT1	sortilin 1 [Source:HGNC Symbol;Acc:11186]	chr1	109852192	109940573	554	0.197	1	1	1.698E-02	2.870E-02	PB-GT
DYNC1I1	dynein, cytoplasmic 1, intermediate chain 1 [Source:HGNC Symbol;Acc:2963]	chr7	95401866	95739634	2603	0.967	3	2	1.701E-02	2.873E-02	PB-GT
DEPDC1B	DEP domain containing 1B [Source:HGNC Symbol;Acc:24902]	chr5	59892739	59996017	731	0.197	1	1	1.704E-02	2.873E-02	PB-GT
SPAST	spastin [Source:HGNC Symbol;Acc:11233]	chr2	32288680	32382706	689	0.197	1	1	1.704E-02	2.873E-02	PB-GT
PAMR1	peptidase domain containing associated with muscle regeneration 1 [Source:HGNC Symbol;Acc:24554]	chr11	35453370	35551848	568	0.197	1	1	1.705E-02	2.873E-02	PB-GT
PIWIL4	piwi-like RNA-mediated gene silencing 4 [Source:HGNC Symbol;Acc:18444]	chr11	94277006	94354587	568	0.197	1	1	1.705E-02	2.873E-02	PB-GT
GABPB1	GA binding protein transcription factor, beta subunit 1 [Source:HGNC Symbol;Acc:4074]	chr15	50569389	50647605	567	0.197	1	1	1.706E-02	2.874E-02	PB-GT
NIPBL	Nipped-B homolog (Drosophila) [Source:HGNC Symbol;Acc:28862]	chr5	36876861	37066515	1982	0.534	2	1	1.711E-02	2.878E-02	PB-GT
KIF13A	kinesin family member 13A [Source:HGNC Symbol;Acc:14566]	chr6	17759414	17987854	1555	0.534	2	2	1.711E-02	2.878E-02	PB-GT
NBPF13P	neuroblastoma breakpoint family, member 13, pseudogene [Source:HGNC Symbol;Acc:31995]	chr1	146490895	146596107	557	0.198	1	1	1.715E-02	2.884E-02	PB-GT
CPOX	coproporphyrinogen oxidase [Source:HGNC Symbol;Acc:2321]	chr3	98239976	98312567	643	0.198	1	1	1.716E-02	2.884E-02	PB-GT
CDK15	cyclin-dependent kinase 15 [Source:HGNC Symbol;Acc:14434]	chr2	202655184	202760273	692	0.198	1	1	1.718E-02	2.884E-02	PB-GT
MPHOSPH9	M-phase phosphoprotein 9 [Source:HGNC Symbol;Acc:7215]	chr12	123636867	123728561	517	0.198	1	1	1.719E-02	2.884E-02	PB-GT
SMAD1	SMAD family member 1 [Source:HGNC Symbol;Acc:6767]	chr4	146402346	146479231	700	0.198	1	1	1.723E-02	2.889E-02	PB-GT
ANKIB1	ankyrin repeat and IBR domain containing 1 [Source:HGNC Symbol;Acc:22215]	chr7	91875548	92030698	1443	0.536	2	1	1.725E-02	2.890E-02	PB-GT
JMY	junction mediating and regulatory protein, p53 cofactor [Source:HGNC Symbol;Acc:28916]	chr5	78532012	78623038	736	0.198	1	1	1.725E-02	2.890E-02	PB-GT
HEPHL1	hephaestin-like 1 [Source:HGNC Symbol;Acc:30477]	chr11	93754527	93846917	572	0.198	1	1	1.728E-02	2.892E-02	PB-GT
CCDC138	coiled-coil domain containing 138 [Source:HGNC Symbol;Acc:26531]	chr2	109403213	109493034	695	0.199	1	1	1.732E-02	2.896E-02	PB-GT
FBXL5	F-box and leucine-rich repeat protein 5 [Source:HGNC Symbol;Acc:13602]	chr4	15606162	15683302	702	0.199	1	1	1.732E-02	2.896E-02	PB-GT
BZW2	basic leucine zipper and W2 domains 2 [Source:HGNC Symbol;Acc:18808]	chr7	16685756	16746148	536	0.199	1	1	1.737E-02	2.902E-02	PB-GT
FOLH1	folate hydrolase (prostate-specific membrane antigen) 1 [Source:HGNC Symbol;Acc:3788]	chr11	49168187	49230222	574	0.199	1	1	1.739E-02	2.905E-02	PB-GT
GPR128	G protein-coupled receptor 128 [Source:HGNC Symbol;Acc:19241]	chr3	100328433	100414323	649	0.200	1	1	1.746E-02	2.916E-02	PB-GT
SASH1	SAM and SH3 domain containing 1 [Source:HGNC Symbol;Acc:19182]	chr6	148593440	148873186	1568	0.539	2	1	1.749E-02	2.917E-02	PB-GT
C11orf80	chromosome 11 open reading frame 80 [Source:HGNC Symbol;Acc:26197]	chr11	66511922	66610987	576	0.200	1	1	1.750E-02	2.918E-02	PB-GT
E2F3	E2F transcription factor 3 [Source:HGNC Symbol;Acc:3115]	chr6	20402398	20493941	582	0.200	1	1	1.751E-02	2.918E-02	PB-GT
ANKRD36B	ankyrin repeat domain 36B [Source:HGNC Symbol;Acc:29333]	chr2	98108978	98206428	702	0.201	1	1	1.765E-02	2.939E-02	PB-GT
ARHGEF11	Rho guanine nucleotide exchange factor (GEF) 11 [Source:HGNC Symbol;Acc:14580]	chr1	156904632	157015162	566	0.201	1	1	1.767E-02	2.942E-02	PB-GT
PRSS12	protease, serine, 12 (neurotrypsin, motopsin) [Source:HGNC Symbol;Acc:9477]	chr4	119201193	119274158	710	0.201	1	1	1.769E-02	2.943E-02	PB-GT
TNKS2	tankyrase, TRF1-interacting ankyrin-related ADP-ribose polymerase 2 [Source:HGNC Symbol;Acc:15677]	chr10	93558069	93625033	564	0.201	1	1	1.773E-02	2.947E-02	PB-GT
WDR11	WD repeat domain 11 [Source:HGNC Symbol;Acc:13831]	chr10	122610687	122669036	564	0.201	1	1	1.773E-02	2.947E-02	PB-GT
ATP8B5P	ATPase, class I, type 8B, member 5, pseudogene [Source:HGNC Symbol;Acc:27245]	chr9	35406752	35483026	538	0.202	1	1	1.782E-02	2.957E-02	PB-GT
IARS	isoleucyl-tRNA synthetase [Source:HGNC Symbol;Acc:5330]	chr9	94972489	95056038	538	0.202	1	1	1.782E-02	2.957E-02	PB-GT
MRPS27	mitochondrial ribosomal protein S27 [Source:HGNC Symbol;Acc:14512]	chr5	71515236	71616473	749	0.202	1	1	1.783E-02	2.957E-02	PB-GT
CEP57L1	centrosomal protein 57kDa-like 1 [Source:HGNC Symbol;Acc:21561]	chr6	109416313	109485135	588	0.202	1	1	1.785E-02	2.959E-02	PB-GT
ARSJ	arylsulfatase family, member J [Source:HGNC Symbol;Acc:26286]	chr4	114821440	114900883	716	0.203	1	1	1.797E-02	2.977E-02	PB-GT
NR2C2	nuclear receptor subfamily 2, group C, member 2 [Source:HGNC Symbol;Acc:7972]	chr3	14989091	15095107	660	0.203	1	1	1.802E-02	2.984E-02	PB-GT
PSPC1	paraspeckle component 1 [Source:HGNC Symbol;Acc:20320]	chr13	20248896	20357142	837	0.203	1	1	1.805E-02	2.988E-02	PB-GT
PPP1R14C	protein phosphatase 1, regulatory (inhibitor) subunit 14C [Source:HGNC Symbol;Acc:14952]	chr6	150464212	150571493	593	0.204	1	1	1.813E-02	2.999E-02	PB-GT
TMED3	transmembrane emp24 protein transport domain containing 3 [Source:HGNC Symbol;Acc:28889]	chr15	79603404	79704334	586	0.204	1	1	1.815E-02	3.000E-02	PB-GT
DPYS	dihydropyrimidinase [Source:HGNC Symbol;Acc:3013]	chr8	105391652	105479281	596	0.204	1	1	1.821E-02	3.009E-02	PB-GT
MFSD1	major facilitator superfamily domain containing 1 [Source:HGNC Symbol;Acc:25874]	chr3	158449987	158547508	666	0.205	1	1	1.833E-02	3.026E-02	PB-GT
CLCN5	chloride channel, voltage-sensitive 5 [Source:HGNC Symbol;Acc:2023]	chrX	49687225	49863892	1237	0.206	1	1	1.853E-02	3.058E-02	PB-GT
EIF4G3	eukaryotic translation initiation factor 4 gamma, 3 [Source:HGNC Symbol;Acc:3298]	chr1	21132963	21503377	2799	0.994	3	1	1.860E-02	3.067E-02	PB-GT
RBL1	retinoblastoma-like 1 (p107) [Source:HGNC Symbol;Acc:9893]	chr20	35624752	35724398	570	0.207	1	1	1.861E-02	3.067E-02	PB-GT
SND1	staphylococcal nuclease and tudor domain containing 1 [Source:HGNC Symbol;Acc:30646]	chr7	127292234	127732661	2682	0.996	3	1	1.874E-02	3.087E-02	PB-GT
ST3GAL6	ST3 beta-galactoside alpha-2,3-sialyltransferase 6 [Source:HGNC Symbol;Acc:18080]	chr3	98451080	98540045	676	0.208	1	1	1.885E-02	3.103E-02	PB-GT
ACSL3	acyl-CoA synthetase long-chain family member 3 [Source:HGNC Symbol;Acc:3570]	chr2	223725652	223809357	728	0.208	1	1	1.889E-02	3.107E-02	PB-GT
WDR35	WD repeat domain 35 [Source:HGNC Symbol;Acc:29250]	chr2	20110021	20189892	729	0.208	1	1	1.893E-02	3.111E-02	PB-GT
ALS2	amyotrophic lateral sclerosis 2 (juvenile) [Source:HGNC Symbol;Acc:443]	chr2	202565277	202645912	729	0.208	1	1	1.893E-02	3.111E-02	PB-GT
LRIG1	leucine-rich repeats and immunoglobulin-like domains 1 [Source:HGNC Symbol;Acc:17360]	chr3	66429221	66551687	678	0.209	1	1	1.895E-02	3.111E-02	PB-GT
SH3TC2	SH3 domain and tetratricopeptide repeats 2 [Source:HGNC Symbol;Acc:29427]	chr5	148303202	148442726	774	0.209	1	1	1.895E-02	3.111E-02	PB-GT
MTDH	metadherin [Source:HGNC Symbol;Acc:29608]	chr8	98656407	98740998	609	0.209	1	1	1.896E-02	3.111E-02	PB-GT
SNAP25	synaptosomal-associated protein, 25kDa [Source:HGNC Symbol;Acc:11132]	chr20	10199478	10288066	577	0.209	1	1	1.903E-02	3.121E-02	PB-GT
PTK2B	protein tyrosine kinase 2 beta [Source:HGNC Symbol;Acc:9612]	chr8	27168999	27316903	611	0.209	1	1	1.908E-02	3.126E-02	PB-GT
REV3L	REV3-like, polymerase (DNA directed), zeta, catalytic subunit [Source:HGNC Symbol;Acc:9968]	chr6	111620234	111804918	1622	0.557	2	1	1.910E-02	3.126E-02	PB-GT
TMCC3	transmembrane and coiled-coil domain family 3 [Source:HGNC Symbol;Acc:29199]	chr12	94960900	95044338	547	0.209	1	1	1.910E-02	3.126E-02	PB-GT
VPS45	vacuolar protein sorting 45 homolog (S. cerevisiae) [Source:HGNC Symbol;Acc:14579]	chr1	150039369	150117505	591	0.210	1	1	1.916E-02	3.133E-02	PB-GT
AASS	aminoadipate-semialdehyde synthase [Source:HGNC Symbol;Acc:17366]	chr7	121715701	121784334	565	0.210	1	1	1.916E-02	3.133E-02	PB-GT
KLHL7	kelch-like family member 7 [Source:HGNC Symbol;Acc:15646]	chr7	23145353	23215040	566	0.210	1	1	1.922E-02	3.140E-02	PB-GT
INMT-FAM188B	INMT-FAM188B readthrough (non-protein coding) [Source:HGNC Symbol;Acc:41995]	chr7	30791753	30931696	566	0.210	1	1	1.922E-02	3.140E-02	PB-GT
ORC3	origin recognition complex, subunit 3 [Source:HGNC Symbol;Acc:8489]	chr6	88299839	88377169	614	0.211	1	1	1.935E-02	3.158E-02	PB-GT
CPEB2	cytoplasmic polyadenylation element binding protein 2 [Source:HGNC Symbol;Acc:21745]	chr4	15004298	15071777	747	0.211	1	1	1.945E-02	3.173E-02	PB-GT
ZNF518A	zinc finger protein 518A [Source:HGNC Symbol;Acc:29009]	chr10	97889472	97965044	593	0.212	1	1	1.947E-02	3.175E-02	PB-GT
C9orf85	chromosome 9 open reading frame 85 [Source:HGNC Symbol;Acc:28784]	chr9	74526426	74600970	565	0.212	1	1	1.952E-02	3.179E-02	PB-GT
DCAF10	DDB1 and CUL4 associated factor 10 [Source:HGNC Symbol;Acc:23686]	chr9	37800499	37867663	565	0.212	1	1	1.952E-02	3.179E-02	PB-GT
PHLDB2	pleckstrin homology-like domain, family B, member 2 [Source:HGNC Symbol;Acc:29573]	chr3	111451344	111695364	1829	0.563	2	1	1.959E-02	3.188E-02	PB-GT
ALKBH8	alkB, alkylation repair homolog 8 (E. coli) [Source:HGNC Symbol;Acc:25189]	chr11	107373452	107436472	612	0.212	1	1	1.960E-02	3.188E-02	PB-GT
LRCH3	leucine-rich repeats and calponin homology (CH) domain containing 3 [Source:HGNC Symbol;Acc:28637]	chr3	197518097	197615307	691	0.213	1	1	1.963E-02	3.192E-02	PB-GT
OSBPL9	oxysterol binding protein-like 9 [Source:HGNC Symbol;Acc:16386]	chr1	52042851	52254889	1587	0.563	2	2	1.965E-02	3.193E-02	PB-GT
ORC4	origin recognition complex, subunit 4 [Source:HGNC Symbol;Acc:8490]	chr2	148691732	148779147	746	0.213	1	1	1.976E-02	3.210E-02	PB-GT
CPNE3	copine III [Source:HGNC Symbol;Acc:2316]	chr8	87497059	87573726	623	0.213	1	1	1.978E-02	3.211E-02	PB-GT
EFHC1	EF-hand domain (C-terminal) containing 1 [Source:HGNC Symbol;Acc:16406]	chr6	52285106	52387892	622	0.214	1	1	1.982E-02	3.215E-02	PB-GT
NBN	nibrin [Source:HGNC Symbol;Acc:7652]	chr8	90945564	91015456	624	0.214	1	1	1.984E-02	3.217E-02	PB-GT
B3GALTL	beta 1,3-galactosyltransferase-like [Source:HGNC Symbol;Acc:20207]	chr13	31774073	31906413	881	0.214	1	1	1.986E-02	3.218E-02	PB-GT
ZYG11B	zyg-11 family member B, cell cycle regulator [Source:HGNC Symbol;Acc:25820]	chr1	53192126	53293014	603	0.214	1	1	1.989E-02	3.221E-02	PB-GT
SLC25A13	solute carrier family 25 (aspartate/glutamate carrier), member 13 [Source:HGNC Symbol;Acc:10983]	chr7	95749532	95951459	1525	0.566	2	1	1.992E-02	3.223E-02	PB-GT
GTF2E2	general transcription factor IIE, polypeptide 2, beta 34kDa [Source:HGNC Symbol;Acc:4651]	chr8	30435835	30515768	626	0.214	1	1	1.996E-02	3.227E-02	PB-GT
MXI1	MAX interactor 1, dimerization protein [Source:HGNC Symbol;Acc:7534]	chr10	111967363	112047123	601	0.214	1	1	1.996E-02	3.227E-02	PB-GT
GLI2	GLI family zinc finger 2 [Source:HGNC Symbol;Acc:4318]	chr2	121493199	121750229	751	0.215	1	1	2.001E-02	3.233E-02	PB-GT
NOTCH2NL	notch 2 N-terminal like [Source:HGNC Symbol;Acc:31862]	chr1	145209119	145291972	606	0.215	1	1	2.007E-02	3.241E-02	PB-GT
REV1	REV1, polymerase (DNA directed) [Source:HGNC Symbol;Acc:14060]	chr2	100016938	100106497	753	0.215	1	1	2.011E-02	3.246E-02	PB-GT
SMAD4	SMAD family member 4 [Source:HGNC Symbol;Acc:6770]	chr18	48494410	48611415	902	0.216	1	1	2.023E-02	3.263E-02	PB-GT
FARSB	phenylalanyl-tRNA synthetase, beta subunit [Source:HGNC Symbol;Acc:17800]	chr2	223435255	223521056	756	0.216	1	1	2.026E-02	3.266E-02	PB-GT
PIGU	phosphatidylinositol glycan anchor biosynthesis, class U [Source:HGNC Symbol;Acc:15791]	chr20	33148346	33264910	597	0.216	1	1	2.028E-02	3.268E-02	PB-GT
MLL	myeloid/lymphoid or mixed-lineage leukemia (trithorax homolog, Drosophila) [Source:HGNC Symbol;Acc:7132]	chr11	118307205	118397539	624	0.217	1	1	2.032E-02	3.272E-02	PB-GT
TMLHE	trimethyllysine hydroxylase, epsilon [Source:HGNC Symbol;Acc:18308]	chrX	154719776	154899605	1301	0.217	1	1	2.036E-02	3.276E-02	PB-GT
CR1L	complement component (3b/4b) receptor 1-like [Source:HGNC Symbol;Acc:2335]	chr1	207818458	207911761	613	0.218	1	1	2.051E-02	3.295E-02	PB-GT
MAP3K2	mitogen-activated protein kinase kinase kinase 2 [Source:HGNC Symbol;Acc:6854]	chr2	128056306	128146041	761	0.218	1	1	2.051E-02	3.295E-02	PB-GT
ATG2B	autophagy related 2B [Source:HGNC Symbol;Acc:20187]	chr14	96747595	96830207	803	0.218	1	1	2.054E-02	3.295E-02	PB-GT
IMPG2	interphotoreceptor matrix proteoglycan 2 [Source:HGNC Symbol;Acc:18362]	chr3	100945570	101039404	708	0.218	1	1	2.054E-02	3.295E-02	PB-GT
SGSM1	small G protein signaling modulator 1 [Source:HGNC Symbol;Acc:29410]	chr22	25202236	25323545	436	0.218	1	1	2.055E-02	3.295E-02	PB-GT
LINC00152	long intergenic non-protein coding RNA 152 [Source:HGNC Symbol;Acc:28717]	chr2	87754887	87906324	762	0.218	1	1	2.056E-02	3.295E-02	PB-GT
SMAD2	SMAD family member 2 [Source:HGNC Symbol;Acc:6768]	chr18	45357922	45457515	910	0.218	1	1	2.057E-02	3.295E-02	PB-GT
RCOR3	REST corepressor 3 [Source:HGNC Symbol;Acc:25594]	chr1	211431719	211489727	614	0.218	1	1	2.057E-02	3.295E-02	PB-GT
CHST11	carbohydrate (chondroitin 4) sulfotransferase 11 [Source:HGNC Symbol;Acc:17422]	chr12	104849073	105155792	1500	0.574	2	1	2.065E-02	3.307E-02	PB-GT
RAB10	RAB10, member RAS oncogene family [Source:HGNC Symbol;Acc:9759]	chr2	26256976	26360323	765	0.219	1	1	2.071E-02	3.314E-02	PB-GT
RGS7BP	regulator of G-protein signaling 7 binding protein [Source:HGNC Symbol;Acc:23271]	chr5	63802084	63908139	812	0.219	1	1	2.072E-02	3.314E-02	PB-GT
CEP63	centrosomal protein 63kDa [Source:HGNC Symbol;Acc:25815]	chr3	134204585	134293859	713	0.219	1	1	2.081E-02	3.326E-02	PB-GT
SPRED2	sprouty-related, EVH1 domain containing 2 [Source:HGNC Symbol;Acc:17722]	chr2	65537985	65659771	770	0.220	1	1	2.096E-02	3.349E-02	PB-GT
CMTM8	CKLF-like MARVEL transmembrane domain containing 8 [Source:HGNC Symbol;Acc:19179]	chr3	32280171	32411817	717	0.221	1	1	2.103E-02	3.357E-02	PB-GT
MFHAS1	malignant fibrous histiocytoma amplified sequence 1 [Source:HGNC Symbol;Acc:16982]	chr8	8640864	8751155	644	0.221	1	1	2.104E-02	3.357E-02	PB-GT

table S2. Genes with significant transposon integrations in RKO *BRAF*$^{wt/-/-}$ cells after selection in low glucose medium

Gene ID	Description	Chrom	Start	End	# TTAA sites	Expected	Observed	Libraries	*P*	FDR	Transposon
FAR1	fatty acyl CoA reductase 1 [Source:HGNC Symbol;Acc:26222]	chr11	13690217	13753893	636	0.221	1	1	2.105E-02	3.357E-02	PB-GT
RCOR1	REST corepressor 1 [Source:HGNC Symbol;Acc:17441]	chr14	103058998	103196913	816	0.221	1	1	2.116E-02	3.373E-02	PB-GT
EFHC2	EF-hand domain (C-terminal) containing 2 [Source:HGNC Symbol;Acc:26233]	chrX	44007128	44202923	1329	0.221	1	1	2.118E-02	3.374E-02	PB-GT
SP140	SP140 nuclear body protein [Source:HGNC Symbol;Acc:17133]	chr2	231090445	231223762	775	0.222	1	1	2.122E-02	3.378E-02	PB-GT
WDR19	WD repeat domain 19 [Source:HGNC Symbol;Acc:18340]	chr4	39184024	39287430	783	0.222	1	1	2.123E-02	3.378E-02	PB-GT
CYTH3	cytohesin 3 [Source:HGNC Symbol;Acc:9504]	chr7	6201407	6312275	598	0.222	1	1	2.129E-02	3.387E-02	PB-GT
KPNA1	karyopherin alpha 1 (importin alpha 5) [Source:HGNC Symbol;Acc:6394]	chr3	122140796	122233792	723	0.222	1	1	2.135E-02	3.395E-02	PB-GT
TLK1	tousled-like kinase 1 [Source:HGNC Symbol;Acc:11841]	chr2	171847333	172087824	2036	0.582	2	1	2.140E-02	3.400E-02	PB-GT
PPP2R5A	protein phosphatase 2, regulatory subunit B', alpha [Source:HGNC Symbol;Acc:9309]	chr1	212458879	212535200	629	0.223	1	1	2.151E-02	3.416E-02	PB-GT
PRPSAP2	phosphoribosyl pyrophosphate synthetase-associated protein 2 [Source:HGNC Symbol;Acc:9467]	chr17	18743398	18834581	442	0.224	1	1	2.162E-02	3.430E-02	PB-GT
RNF144A	ring finger protein 144A [Source:HGNC Symbol;Acc:20457]	chr2	7057523	7208417	783	0.224	1	1	2.162E-02	3.430E-02	PB-GT
ZCCHC6	zinc finger, CCHC domain containing 6 [Source:HGNC Symbol;Acc:25817]	chr9	88902648	88969369	598	0.224	1	1	2.169E-02	3.439E-02	PB-GT
NFIB	nuclear factor I/B [Source:HGNC Symbol;Acc:7785]	chr9	14081842	14398982	2780	1.043	3	1	2.172E-02	3.442E-02	PB-GT
OR4N2	olfactory receptor, family 4, subfamily N, member 2 [Source:HGNC Symbol;Acc:14742]	chr14	20187174	20296531	829	0.225	1	1	2.179E-02	3.450E-02	PB-GT
KDM5B	lysine (K)-specific demethylase 5B [Source:HGNC Symbol;Acc:18039]	chr1	202696526	202778598	634	0.225	1	1	2.183E-02	3.455E-02	PB-GT
NRG2	neuregulin 2 [Source:HGNC Symbol;Acc:7998]	chr5	139226364	139422884	836	0.225	1	1	2.187E-02	3.460E-02	PB-GT
KIAA1715	KIAA1715 [Source:HGNC Symbol;Acc:21610]	chr2	176788620	176867567	789	0.226	1	1	2.193E-02	3.467E-02	PB-GT
CALD1	caldesmon 1 [Source:HGNC Symbol;Acc:1441]	chr7	134429003	134655479	1586	0.589	2	2	2.204E-02	3.482E-02	PB-GT
GPR133	G protein-coupled receptor 133 [Source:HGNC Symbol;Acc:19893]	chr12	131438452	131626014	592	0.227	1	1	2.212E-02	3.492E-02	PB-GT
TMEM192	transmembrane protein 192 [Source:HGNC Symbol;Acc:26775]	chr4	165997230	166129701	801	0.227	1	1	2.214E-02	3.494E-02	PB-GT
TMEM56	transmembrane protein 56 [Source:HGNC Symbol;Acc:26477]	chr1	95582894	95663163	641	0.228	1	1	2.228E-02	3.512E-02	PB-GT
UQCC	ubiquinol-cytochrome c reductase complex chaperone [Source:HGNC Symbol;Acc:15891]	chr20	33890369	33999944	628	0.228	1	1	2.228E-02	3.512E-02	PB-GT
ARHGEF3	Rho guanine nucleotide exchange factor (GEF) 3 [Source:HGNC Symbol;Acc:683]	chr3	56761446	57113357	1923	0.592	2	2	2.229E-02	3.512E-02	PB-GT
DOCK11	dedicator of cytokinesis 11 [Source:HGNC Symbol;Acc:23483]	chrX	117629861	117820126	1367	0.228	1	1	2.232E-02	3.514E-02	PB-GT
FLNB	filamin B, beta [Source:HGNC Symbol;Acc:3755]	chr3	57994127	58157982	742	0.228	1	1	2.241E-02	3.525E-02	PB-GT
TGFB2	transforming growth factor, beta 2 [Source:HGNC Symbol;Acc:11768]	chr1	218519577	218617961	643	0.228	1	1	2.241E-02	3.525E-02	PB-GT
C20orf196	chromosome 20 open reading frame 196 [Source:HGNC Symbol;Acc:26318]	chr20	5731039	5844558	631	0.229	1	1	2.247E-02	3.534E-02	PB-GT
ITGA2	integrin, alpha 2 (CD49B, alpha 2 subunit of VLA-2 receptor) [Source:HGNC Symbol;Acc:6137]	chr5	52285156	52390609	849	0.229	1	1	2.251E-02	3.536E-02	PB-GT
ST7-AS2	ST7 antisense RNA 2 [Source:HGNC Symbol;Acc:16044]	chr7	116712126	116786534	617	0.229	1	1	2.256E-02	3.543E-02	PB-GT
LMBR1	limb region 1 homolog (mouse) [Source:HGNC Symbol;Acc:13243]	chr7	156473571	156685924	1602	0.595	2	1	2.261E-02	3.549E-02	PB-GT
CELF4	CUGBP, Elav-like family member 4 [Source:HGNC Symbol;Acc:14015]	chr18	34823010	35146000	959	0.230	1	1	2.267E-02	3.556E-02	PB-GT
MB21D2	Mab-21 domain containing 2 [Source:HGNC Symbol;Acc:30438]	chr3	192514604	192635950	747	0.230	1	1	2.269E-02	3.557E-02	PB-GT
DENND2A	DENN/MADD domain containing 2A [Source:HGNC Symbol;Acc:22212]	chr7	140218220	140373793	619	0.230	1	1	2.270E-02	3.557E-02	PB-GT
ZNF529	zinc finger protein 529 [Source:HGNC Symbol;Acc:29328]	chr19	37025676	37096178	436	0.230	1	1	2.274E-02	3.561E-02	PB-GT
C7orf63	chromosome 7 open reading frame 63 [Source:HGNC Symbol;Acc:26107]	chr7	89874488	89940377	620	0.230	1	1	2.277E-02	3.564E-02	PB-GT
HDAC2	histone deacetylase 2 [Source:HGNC Symbol;Acc:4853]	chr6	114254192	114332472	671	0.230	1	1	2.281E-02	3.569E-02	PB-GT
BLM	Bloom syndrome, RecQ helicase-like [Source:HGNC Symbol;Acc:1058]	chr15	91260558	91358859	663	0.231	1	1	2.283E-02	3.569E-02	PB-GT
LPP	LIM domain containing preferred translocation partner in lipoma [Source:HGNC Symbol;Acc:6679]	chr3	187871072	188608460	5154	1.585	4	2	2.289E-02	3.577E-02	PB-GT
SAMD13	sterile alpha motif domain containing 13 [Source:HGNC Symbol;Acc:24582]	chr1	84764049	84855640	652	0.231	1	1	2.299E-02	3.591E-02	PB-GT
SLC22A15	solute carrier family 22, member 15 [Source:HGNC Symbol;Acc:20301]	chr1	116519119	116612675	653	0.232	1	1	2.306E-02	3.599E-02	PB-GT
KIAA1549L	KIAA1549-like [Source:HGNC Symbol;Acc:24836]	chr11	33563618	33695648	669	0.232	1	1	2.312E-02	3.607E-02	PB-GT
PI4K2B	phosphatidylinositol 4-kinase type 2 beta [Source:HGNC Symbol;Acc:18215]	chr4	25162263	25280714	821	0.232	1	1	2.317E-02	3.612E-02	PB-GT
SLC26A5	solute carrier family 26, member 5 (prestin) [Source:HGNC Symbol;Acc:9359]	chr7	102993177	103086624	626	0.232	1	1	2.317E-02	3.612E-02	PB-GT
CTDSPL	CTD (carboxy-terminal domain, RNA polymerase II, polypeptide A) small phosphatase-like [Source:HGNC Symbol;Acc:16890]	chr3	37903451	38025960	757	0.233	1	1	2.325E-02	3.622E-02	PB-GT
RHOBTB3	Rho-related BTB domain containing 3 [Source:HGNC Symbol;Acc:18757]	chr5	95049226	95160087	865	0.233	1	1	2.330E-02	3.627E-02	PB-GT
HUNK	hormonally up-regulated Neu-associated kinase [Source:HGNC Symbol;Acc:13326]	chr21	33245628	33416946	816	0.233	1	1	2.336E-02	3.635E-02	PB-GT
EVC2	Ellis van Creveld syndrome 2 [Source:HGNC Symbol;Acc:19747]	chr4	5544499	5711275	827	0.234	1	1	2.349E-02	3.652E-02	PB-GT
HECTD1	HECT domain containing E3 ubiquitin protein ligase 1 [Source:HGNC Symbol;Acc:20157]	chr14	31569318	31677010	864	0.234	1	1	2.352E-02	3.656E-02	PB-GT
MARCH11	membrane-associated ring finger (C3HC4) 11 [Source:HGNC Symbol;Acc:33609]	chr5	16067248	16180871	872	0.235	1	1	2.365E-02	3.673E-02	PB-GT
CUL2	cullin 2 [Source:HGNC Symbol;Acc:2552]	chr10	35297479	35379570	660	0.236	1	1	2.375E-02	3.687E-02	PB-GT
PTPN2	protein tyrosine phosphatase, non-receptor type 2 [Source:HGNC Symbol;Acc:9650]	chr18	12785477	12929642	984	0.236	1	1	2.377E-02	3.689E-02	PB-GT
TARBP1	TAR (HIV-1) RNA binding protein 1 [Source:HGNC Symbol;Acc:11568]	chr1	234527059	234614849	666	0.236	1	1	2.391E-02	3.708E-02	PB-GT
FREM3	FRAS1 related extracellular matrix 3 [Source:HGNC Symbol;Acc:25172]	chr4	144498455	144621828	839	0.238	1	1	2.412E-02	3.736E-02	PB-GT
DDHD1	DDHD domain containing 1 [Source:HGNC Symbol;Acc:19714]	chr14	53510686	53620000	876	0.238	1	1	2.413E-02	3.736E-02	PB-GT
ZNF236	zinc finger protein 236 [Source:HGNC Symbol;Acc:13028]	chr18	74534563	74682683	992	0.238	1	1	2.413E-02	3.736E-02	PB-GT
GALNT11	UDP-N-acetyl-alpha-D-galactosamine:polypeptide N-acetylgalactosaminyltransferase 11 (GalNAc-T11) [Source:HGNC Symbol;	chr7	151722759	151819425	641	0.238	1	1	2.421E-02	3.747E-02	PB-GT
ASPH	aspartate beta-hydroxylase [Source:HGNC Symbol;Acc:757]	chr8	62413116	62627155	1784	0.611	2	1	2.424E-02	3.748E-02	PB-GT
ABHD2	abhydrolase domain containing 2 [Source:HGNC Symbol;Acc:18717]	chr15	89630690	89745591	685	0.238	1	1	2.425E-02	3.748E-02	PB-GT
BTBD10	BTB (POZ) domain containing 10 [Source:HGNC Symbol;Acc:21445]	chr11	13409548	13484844	687	0.238	1	1	2.428E-02	3.751E-02	PB-GT
ZNF83	zinc finger protein 83 [Source:HGNC Symbol;Acc:13158]	chr19	53097313	53193749	452	0.239	1	1	2.430E-02	3.753E-02	PB-GT
SEPT9	septin 9 [Source:HGNC Symbol;Acc:7323]	chr17	75276651	75496678	471	0.239	1	1	2.431E-02	3.753E-02	PB-GT
PROS1	protein S (alpha) [Source:HGNC Symbol;Acc:9456]	chr3	93591881	93692910	776	0.239	1	1	2.434E-02	3.754E-02	PB-GT
FAM5B	family with sequence similarity 5, member B [Source:HGNC Symbol;Acc:13746]	chr1	177140633	177251558	673	0.239	1	1	2.438E-02	3.758E-02	PB-GT
NF2	neurofibromin 2 (merlin) [Source:HGNC Symbol;Acc:7773]	chr22	29999545	30094587	479	0.239	1	1	2.445E-02	3.768E-02	PB-GT
ZNF573	zinc finger protein 573 [Source:HGNC Symbol;Acc:26420]	chr19	38226734	38307940	454	0.240	1	1	2.450E-02	3.772E-02	PB-GT
ADAM19	ADAM metallopeptidase domain 19 [Source:HGNC Symbol;Acc:197]	chr5	156822542	157002783	889	0.240	1	1	2.450E-02	3.772E-02	PB-GT
PIK3AP1	phosphoinositide-3-kinase adaptor protein 1 [Source:HGNC Symbol;Acc:30034]	chr10	98353069	98480271	672	0.240	1	1	2.455E-02	3.777E-02	PB-GT
C7orf55-LUC7L2	C7orf55-LUC7L2 readthrough [Source:HGNC Symbol;Acc:44671]	chr7	139025105	139108198	648	0.241	1	1	2.470E-02	3.798E-02	PB-GT
FSIP2	fibrous sheath interacting protein 2 [Source:HGNC Symbol;Acc:21675]	chr2	186603355	186698017	846	0.242	1	1	2.495E-02	3.834E-02	PB-GT
PPP1R12A	protein phosphatase 1, regulatory subunit 12A [Source:HGNC Symbol;Acc:7618]	chr12	80167343	80329240	1618	0.619	2	2	2.508E-02	3.853E-02	PB-GT
STARD9	StAR-related lipid transfer (START) domain containing 9 [Source:HGNC Symbol;Acc:19162]	chr15	42867857	43013179	698	0.243	1	1	2.510E-02	3.853E-02	PB-GT
STX18	syntaxin 18 [Source:HGNC Symbol;Acc:15942]	chr4	4417469	4544073	859	0.243	1	1	2.519E-02	3.865E-02	PB-GT
DDX60	DEAD (Asp-Glu-Ala-Asp) box polypeptide 60 [Source:HGNC Symbol;Acc:25942]	chr4	169137444	169239958	860	0.243	1	1	2.524E-02	3.871E-02	PB-GT
SPRN	shadow of prion protein homolog (zebrafish) [Source:HGNC Symbol;Acc:16871]	chr10	135234170	135382916	683	0.244	1	1	2.529E-02	3.875E-02	PB-GT
PHEX	phosphate regulating endopeptidase homolog, X-linked [Source:HGNC Symbol;Acc:8918]	chrX	22050559	22269427	1463	0.244	1	1	2.529E-02	3.875E-02	PB-GT
ITGAV	integrin, alpha V [Source:HGNC Symbol;Acc:6150]	chr2	187454792	187545628	853	0.244	1	1	2.533E-02	3.878E-02	PB-GT
NLRP1	NLR family, pyrin domain containing 1 [Source:HGNC Symbol;Acc:14374]	chr17	5402747	5522744	482	0.244	1	1	2.537E-02	3.882E-02	PB-GT
FANCL	Fanconi anemia, complementation group L [Source:HGNC Symbol;Acc:20748]	chr2	58386378	58468507	854	0.244	1	1	2.538E-02	3.882E-02	PB-GT
A1CF	APOBEC1 complementation factor [Source:HGNC Symbol;Acc:24086]	chr10	52566322	52645435	685	0.244	1	1	2.543E-02	3.886E-02	PB-GT
PIKFYVE	phosphoinositide kinase, FYVE finger containing [Source:HGNC Symbol;Acc:23785]	chr2	209130991	209223475	855	0.245	1	1	2.544E-02	3.886E-02	PB-GT
CROCC	ciliary rootlet coiled-coil, rootletin [Source:HGNC Symbol;Acc:21299]	chr1	17066768	17299474	689	0.245	1	1	2.545E-02	3.887E-02	PB-GT
TMTC3	transmembrane and tetratricopeptide repeat containing 3 [Source:HGNC Symbol;Acc:26899]	chr12	88536073	88593664	640	0.245	1	1	2.554E-02	3.898E-02	PB-GT
PPP1R42	protein phosphatase 1, regulatory subunit 42 [Source:HGNC Symbol;Acc:33732]	chr8	67876334	67968839	718	0.246	1	1	2.572E-02	3.923E-02	PB-GT
ACVR2A	activin A receptor, type IIA [Source:HGNC Symbol;Acc:173]	chr2	148602086	148688393	861	0.246	1	1	2.577E-02	3.928E-02	PB-GT
SEC24B	SEC24 family, member B (S. cerevisiae) [Source:HGNC Symbol;Acc:10704]	chr4	110354928	110462052	870	0.246	1	1	2.579E-02	3.929E-02	PB-GT
APAF1	apoptotic peptidase activating factor 1 [Source:HGNC Symbol;Acc:576]	chr12	99038919	99129204	644	0.247	1	1	2.583E-02	3.930E-02	PB-GT
EPHA1-AS1	EPHA1 antisense RNA 1 [Source:HGNC Symbol;Acc:27799]	chr7	143104906	143220542	664	0.247	1	1	2.583E-02	3.930E-02	PB-GT
MAP3K7	mitogen-activated protein kinase kinase kinase 7 [Source:HGNC Symbol;Acc:6859]	chr6	91223292	91296764	718	0.247	1	1	2.585E-02	3.930E-02	PB-GT
USP49	ubiquitin specific peptidase 49 [Source:HGNC Symbol;Acc:20078]	chr6	41757634	41863099	718	0.247	1	1	2.585E-02	3.930E-02	PB-GT
CSNK1E	casein kinase 1, epsilon [Source:HGNC Symbol;Acc:2453]	chr22	38686697	38794527	494	0.247	1	1	2.588E-02	3.933E-02	PB-GT
SYNCRIP	synaptotagmin binding, cytoplasmic RNA interacting protein [Source:HGNC Symbol;Acc:16918]	chr6	86267696	86353510	721	0.248	1	1	2.605E-02	3.956E-02	PB-GT
DENND2C	DENN/MADD domain containing 2C [Source:HGNC Symbol;Acc:24748]	chr1	115125469	115213043	698	0.248	1	1	2.607E-02	3.957E-02	PB-GT
PMS1	PMS1 postmeiotic segregation increased 1 (S. cerevisiae) [Source:HGNC Symbol;Acc:9121]	chr2	190649107	190742355	867	0.248	1	1	2.610E-02	3.960E-02	PB-GT
ZNF540	zinc finger protein 540 [Source:HGNC Symbol;Acc:25331]	chr19	38042308	38104998	470	0.248	1	1	2.611E-02	3.960E-02	PB-GT
FSTL5	follistatin-like 5 [Source:HGNC Symbol;Acc:21386]	chr4	162305049	163085187	7871	2.228	5	2	2.629E-02	3.985E-02	PB-GT
LNPEP	leucyl/cystinyl aminopeptidase [Source:HGNC Symbol;Acc:6656]	chr5	96271098	96373219	925	0.249	1	1	2.636E-02	3.993E-02	PB-GT
RSBN1L	round spermatid basic protein 1-like [Source:HGNC Symbol;Acc:24765]	chr7	77325760	77409008	672	0.250	1	1	2.641E-02	3.998E-02	PB-GT
GTF2I	general transcription factor IIi [Source:HGNC Symbol;Acc:4659]	chr7	74071994	74175026	673	0.250	1	1	2.648E-02	4.007E-02	PB-GT
ARHGEF28	Rho guanine nucleotide exchange factor (GEF) 28 [Source:HGNC Symbol;Acc:30322]	chr5	72921983	73237818	2349	0.633	2	2	2.651E-02	4.009E-02	PB-GT
PON1	paraoxonase 1 [Source:HGNC Symbol;Acc:9204]	chr7	94926988	95025673	674	0.250	1	1	2.655E-02	4.014E-02	PB-GT
PPIP5K2	diphosphoinositol pentakisphosphate kinase 2 [Source:HGNC Symbol;Acc:29035]	chr5	102455853	102538937	930	0.251	1	1	2.662E-02	4.022E-02	PB-GT
ZBTB38	zinc finger and BTB domain containing 38 [Source:HGNC Symbol;Acc:26636]	chr3	141043055	141168634	816	0.251	1	1	2.670E-02	4.031E-02	PB-GT
AFAP1	actin filament associated protein 1 [Source:HGNC Symbol;Acc:24017]	chr4	7760441	7941653	888	0.251	1	1	2.678E-02	4.041E-02	PB-GT
ABCD2	ATP-binding cassette, sub-family D (ALD), member 2 [Source:HGNC Symbol;Acc:66]	chr12	39943835	40013553	657	0.252	1	1	2.680E-02	4.041E-02	PB-GT
C1orf168	chromosome 1 open reading frame 168 [Source:HGNC Symbol;Acc:27295]	chr1	57184477	57285369	709	0.252	1	1	2.683E-02	4.041E-02	PB-GT
KLHL2	kelch-like family member 2 [Source:HGNC Symbol;Acc:6353]	chr4	166128770	166244308	889	0.252	1	1	2.683E-02	4.041E-02	PB-GT
RAB27A	RAB27A, member RAS oncogene family [Source:HGNC Symbol;Acc:9766]	chr15	55495164	55611311	724	0.252	1	1	2.685E-02	4.041E-02	PB-GT
BAGE2	B melanoma antigen family, member 2 [Source:HGNC Symbol;Acc:15723]	chr21	10996026	11098980	880	0.252	1	1	2.685E-02	4.041E-02	PB-GT
CPPED1	calcineurin-like phosphoesterase domain containing 1 [Source:HGNC Symbol;Acc:25632]	chr16	12756919	12897874	756	0.253	1	1	2.714E-02	4.078E-02	PB-GT
MARCH10	membrane-associated ring finger (C3HC4) 10, E3 ubiquitin protein ligase [Source:HGNC Symbol;Acc:26655]	chr17	60778675	60885705	500	0.253	1	1	2.714E-02	4.078E-02	PB-GT
CEP128	centrosomal protein 128kDa [Source:HGNC Symbol;Acc:20359]	chr14	80943330	81425861	4125	1.119	3	2	2.715E-02	4.078E-02	PB-GT
TRAPPC8	trafficking protein particle complex 8 [Source:HGNC Symbol;Acc:29169]	chr18	29409136	29533099	1058	0.253	1	1	2.716E-02	4.078E-02	PB-GT
NRD1	nardilysin (N-arginine dibasic convertase) [Source:HGNC Symbol;Acc:7995]	chr1	52254863	52344477	714	0.253	1	1	2.718E-02	4.078E-02	PB-GT
AGL	amylo-alpha-1, 6-glucosidase, 4-alpha-glucanotransferase [Source:HGNC Symbol;Acc:321]	chr1	100315640	100389579	714	0.253	1	1	2.718E-02	4.078E-02	PB-GT
BIRC6	baculoviral IAP repeat containing 6 [Source:HGNC Symbol;Acc:13516]	chr2	32582096	32843966	2237	0.640	2	1	2.722E-02	4.082E-02	PB-GT
LIMA1	LIM domain and actin binding 1 [Source:HGNC Symbol;Acc:24636]	chr12	50569571	50677329	664	0.254	1	1	2.733E-02	4.096E-02	PB-GT
PEX7	peroxisomal biogenesis factor 7 [Source:HGNC Symbol;Acc:8860]	chr6	137143717	137235075	741	0.255	1	1	2.739E-02	4.103E-02	PB-GT
APP	amyloid beta (A4) precursor protein [Source:HGNC Symbol;Acc:620]	chr21	27252861	27543446	2242	0.641	2	1	2.741E-02	4.105E-02	PB-GT
KIAA1033	KIAA1033 [Source:HGNC Symbol;Acc:29174]	chr12	105501102	105562912	666	0.255	1	1	2.748E-02	4.112E-02	PB-GT
MED13L	mediator complex subunit 13-like [Source:HGNC Symbol;Acc:22962]	chr12	116395711	116715143	2934	1.123	3	2	2.750E-02	4.113E-02	PB-GT
GAPVD1	GTPase activating protein and VPS9 domains 1 [Source:HGNC Symbol;Acc:23375]	chr9	128024073	128129486	681	0.255	1	1	2.757E-02	4.121E-02	PB-GT
COLGALT2	collagen beta(1-O)galactosyltransferase 2 [Source:HGNC Symbol;Acc:16790]	chr1	183898796	184006863	720	0.256	1	1	2.760E-02	4.124E-02	PB-GT
ADCY9	adenylate cyclase 9 [Source:HGNC Symbol;Acc:240]	chr16	4003388	4166186	764	0.256	1	1	2.766E-02	4.132E-02	PB-GT
FBXL13	F-box and leucine-rich repeat protein 13 [Source:HGNC Symbol;Acc:21658]	chr7	102453308	102715286	1735	0.644	2	1	2.771E-02	4.134E-02	PB-GT
USP37	ubiquitin specific peptidase 37 [Source:HGNC Symbol;Acc:20063]	chr2	219314974	219433084	896	0.256	1	1	2.772E-02	4.134E-02	PB-GT
STAMBPL1	STAM binding protein-like 1 [Source:HGNC Symbol;Acc:24105]	chr10	90639491	90734910	718	0.256	1	1	2.773E-02	4.134E-02	PB-GT
ABI2	abl-interactor 2 [Source:HGNC Symbol;Acc:24011]	chr2	204192942	204301606	897	0.257	1	1	2.778E-02	4.140E-02	PB-GT
EDARADD	EDAR-associated death domain [Source:HGNC Symbol;Acc:14341]	chr1	236511562	236648214	724	0.257	1	1	2.788E-02	4.153E-02	PB-GT
P4HA1	prolyl 4-hydroxylase, alpha polypeptide I [Source:HGNC Symbol;Acc:8546]	chr10	74766975	74856732	722	0.258	1	1	2.801E-02	4.170E-02	PB-GT
USP13	ubiquitin specific peptidase 13 (isopeptidase T-3) [Source:HGNC Symbol;Acc:12611]	chr3	179370543	179507189	840	0.258	1	1	2.816E-02	4.189E-02	PB-GT
SCIN	scinderin [Source:HGNC Symbol;Acc:21695]	chr7	12610203	12693228	696	0.258	1	1	2.817E-02	4.189E-02	PB-GT
EDEM2	ER degradation enhancer, mannosidase alpha-like 2 [Source:HGNC Symbol;Acc:15877]	chr20	33703160	33865928	715	0.259	1	1	2.829E-02	4.205E-02	PB-GT
SCAMP1	secretory carrier membrane protein 1 [Source:HGNC Symbol;Acc:10563]	chr5	77656339	77776562	962	0.259	1	1	2.833E-02	4.208E-02	PB-GT
DENND5A	DENN/MADD domain containing 5A [Source:HGNC Symbol;Acc:19344]	chr11	9160372	9286937	748	0.260	1	1	2.839E-02	4.215E-02	PB-GT
NSRP1	nuclear speckle splicing regulatory protein 1 [Source:HGNC Symbol;Acc:25305]	chr17	28442539	28513493	513	0.260	1	1	2.845E-02	4.222E-02	PB-GT
CTAGE5	CTAGE family, member 5 [Source:HGNC Symbol;Acc:7057]	chr14	39734488	39856156	961	0.261	1	1	2.860E-02	4.243E-02	PB-GT
PPP2R2A	protein phosphatase 2, regulatory subunit B, alpha [Source:HGNC Symbol;Acc:9304]	chr8	26149007	26230196	762	0.261	1	1	2.869E-02	4.252E-02	PB-GT
COL6A6	collagen, type VI, alpha 6 [Source:HGNC Symbol;Acc:27023]	chr3	130279178	130396999	849	0.261	1	1	2.871E-02	4.252E-02	PB-GT

table S2. Genes with significant transposon integrations in RKO *BRAF*$^{wt/-/-}$ cells after selection in low glucose medium

Gene ID	Description	Chrom	Start	End	# TTAA sites	Expected	Observed	Libraries	*P*	FDR	Transposon
BAZ1A	bromodomain adjacent to zinc finger domain, 1A [Source:HGNC Symbol;Acc:960]	chr14	35221937	35344853	963	0.261	1	1	2.871E-02	4.252E-02	PB-GT
TEC	tec protein tyrosine kinase [Source:HGNC Symbol;Acc:11719]	chr4	48137800	48271881	925	0.262	1	1	2.886E-02	4.270E-02	PB-GT
UBR4	ubiquitin protein ligase E3 component n-recognin 4 [Source:HGNC Symbol;Acc:30313]	chr1	19401000	19536770	738	0.262	1	1	2.887E-02	4.270E-02	PB-GT
RC3H1	ring finger and CCCH-type domains 1 [Source:HGNC Symbol;Acc:29434]	chr1	173900352	173991435	738	0.262	1	1	2.887E-02	4.270E-02	PB-GT
ANO10	anoctamin 10 [Source:HGNC Symbol;Acc:25519]	chr3	43396351	43733086	2135	0.657	2	2	2.909E-02	4.300E-02	PB-GT
ANXA8L1	annexin A8-like 1 [Source:HGNC Symbol;Acc:23334]	chr10	47011753	47174093	738	0.263	1	1	2.916E-02	4.307E-02	PB-GT
LAMA1	laminin, alpha 1 [Source:HGNC Symbol;Acc:6481]	chr18	6941743	7117813	1100	0.263	1	1	2.917E-02	4.307E-02	PB-GT
SLC12A6	solute carrier family 12 (potassium/chloride transporters), member 6 [Source:HGNC Symbol;Acc:10914]	chr15	34525460	34630261	758	0.264	1	1	2.920E-02	4.310E-02	PB-GT
TXNDC16	thioredoxin domain containing 16 [Source:HGNC Symbol;Acc:19965]	chr14	52897308	53019240	973	0.264	1	1	2.926E-02	4.316E-02	PB-GT
TACC1	transforming, acidic coiled-coil containing protein 1 [Source:HGNC Symbol;Acc:11522]	chr8	38585704	38710546	771	0.264	1	1	2.931E-02	4.321E-02	PB-GT
GRAMD1C	GRAM domain containing 1C [Source:HGNC Symbol;Acc:25252]	chr3	113547029	113666021	860	0.265	1	1	2.939E-02	4.331E-02	PB-GT
PPAPDC1A	phosphatidic acid phosphatase type 2 domain containing 1A [Source:HGNC Symbol;Acc:23531]	chr10	122216466	122349367	742	0.265	1	1	2.945E-02	4.334E-02	PB-GT
DNMBP	dynamin binding protein [Source:HGNC Symbol;Acc:30373]	chr10	101635334	101769676	742	0.265	1	1	2.945E-02	4.334E-02	PB-GT
TFPI	tissue factor pathway inhibitor (lipoprotein-associated coagulation inhibitor) [Source:HGNC Symbol;Acc:11760]	chr2	188328957	188430487	927	0.265	1	1	2.950E-02	4.334E-02	PB-GT
DYSF	dysferlin, limb girdle muscular dystrophy 2B (autosomal recessive) [Source:HGNC Symbol;Acc:3097]	chr2	71680852	71913898	927	0.265	1	1	2.950E-02	4.334E-02	PB-GT
SP4	Sp4 transcription factor [Source:HGNC Symbol;Acc:11209]	chr7	21467652	21554440	714	0.265	1	1	2.951E-02	4.334E-02	PB-GT
SGCE	sarcoglycan, epsilon [Source:HGNC Symbol;Acc:10808]	chr7	94214542	94285521	714	0.265	1	1	2.951E-02	4.334E-02	PB-GT
TPD52	tumor protein D52 [Source:HGNC Symbol;Acc:12005]	chr8	80870571	81143467	1928	0.661	2	2	2.952E-02	4.334E-02	PB-GT
MBNL1	muscleblind-like splicing regulator 1 [Source:HGNC Symbol;Acc:6923]	chr3	151961617	152183569	2148	0.661	2	2	2.954E-02	4.335E-02	PB-GT
ETS1	v-ets erythroblastosis virus E26 oncogene homolog 1 (avian) [Source:HGNC Symbol;Acc:3488]	chr11	128328656	128457437	765	0.265	1	1	2.958E-02	4.336E-02	PB-GT
MTMR2	myotubularin related protein 2 [Source:HGNC Symbol;Acc:7450]	chr11	95566046	95658479	765	0.265	1	1	2.958E-02	4.336E-02	PB-GT
SNX14	sorting nexin 14 [Source:HGNC Symbol;Acc:14977]	chr6	86215214	86303874	773	0.266	1	1	2.959E-02	4.336E-02	PB-GT
NOS1AP	nitric oxide synthase 1 (neuronal) adaptor protein [Source:HGNC Symbol;Acc:16859]	chr1	162039564	162353321	1868	0.663	2	1	2.981E-02	4.365E-02	PB-GT
OTX2-AS1	OTX2 antisense RNA 1 (head to head) [Source:HGNC Symbol;Acc:43906]	chr14	57279901	57618895	2447	0.664	2	1	2.987E-02	4.371E-02	PB-GT
SLC9C2	solute carrier family 9, member C2 (putative) [Source:HGNC Symbol;Acc:28664]	chr1	173469603	173572233	752	0.267	1	1	2.988E-02	4.371E-02	PB-GT
GBA3	glucosidase, beta, acid 3 (cytosolic) [Source:HGNC Symbol;Acc:19069]	chr4	22694537	22821198	943	0.267	1	1	2.989E-02	4.371E-02	PB-GT
LRRK1	leucine-rich repeat kinase 1 [Source:HGNC Symbol;Acc:18608]	chr15	101459420	101610317	768	0.267	1	1	2.991E-02	4.371E-02	PB-GT
UBE2W	ubiquitin-conjugating enzyme E2W (putative) [Source:HGNC Symbol;Acc:25616]	chr8	74692332	74791145	781	0.268	1	1	3.001E-02	4.383E-02	PB-GT
MSH2	mutS homolog 2, colon cancer, nonpolyposis type 1 (E. coli) [Source:HGNC Symbol;Acc:7325]	chr2	47630108	47789450	937	0.268	1	1	3.009E-02	4.392E-02	PB-GT
CNST	consortin, connexin sorting protein [Source:HGNC Symbol;Acc:26486]	chr1	246729746	246831886	755	0.268	1	1	3.010E-02	4.392E-02	PB-GT
ZNF670	zinc finger protein 670 [Source:HGNC Symbol;Acc:28167]	chr1	247108849	247242113	756	0.268	1	1	3.017E-02	4.401E-02	PB-GT
RERE	arginine-glutamic acid dipeptide (RE) repeats [Source:HGNC Symbol;Acc:9965]	chr1	8412457	8877702	3261	1.158	3	2	3.021E-02	4.404E-02	PB-GT
FILIP1L	filamin A interacting protein 1-like [Source:HGNC Symbol;Acc:24589]	chr3	99548985	99833357	2171	0.668	2	1	3.035E-02	4.421E-02	PB-GT
TDRD5	tudor domain containing 5 [Source:HGNC Symbol;Acc:20614]	chr1	179560748	179660407	760	0.270	1	1	3.047E-02	4.437E-02	PB-GT
METTL16	methyltransferase like 16 [Source:HGNC Symbol;Acc:28484]	chr17	2319343	2415185	533	0.270	1	1	3.051E-02	4.440E-02	PB-GT
TRRAP	transformation/transcription domain-associated protein [Source:HGNC Symbol;Acc:12347]	chr7	98475556	98610866	728	0.270	1	1	3.058E-02	4.444E-02	PB-GT
GCNT2	glucosaminyl (N-acetyl) transferase 2, I-branching enzyme (I blood group) [Source:HGNC Symbol;Acc:4204]	chr6	10492456	10629601	787	0.270	1	1	3.058E-02	4.444E-02	PB-GT
UBAP2	ubiquitin associated protein 2 [Source:HGNC Symbol;Acc:14185]	chr9	33921691	34048947	721	0.270	1	1	3.060E-02	4.444E-02	PB-GT
LIMCH1	LIM and calponin homology domains 1 [Source:HGNC Symbol;Acc:29191]	chr4	41361624	41702061	2367	0.670	2	1	3.061E-02	4.444E-02	PB-GT
HEATR5B	HEAT repeat containing 5B [Source:HGNC Symbol;Acc:29273]	chr2	37195526	37311485	946	0.271	1	1	3.062E-02	4.444E-02	PB-GT
TMOD3	tropomodulin 3 (ubiquitous) [Source:HGNC Symbol;Acc:11873]	chr15	52121825	52239492	778	0.271	1	1	3.062E-02	4.444E-02	PB-GT
ATP11C	ATPase, class VI, type 11C [Source:HGNC Symbol;Acc:13554]	chrX	138808505	139027435	1625	0.271	1	1	3.066E-02	4.447E-02	PB-GT
SUSD1	sushi domain containing 1 [Source:HGNC Symbol;Acc:25413]	chr9	114803065	114937688	722	0.271	1	1	3.068E-02	4.447E-02	PB-GT
TINAG	tubulointerstitial nephritis antigen [Source:HGNC Symbol;Acc:14599]	chr6	54172657	54254950	790	0.271	1	1	3.079E-02	4.461E-02	PB-GT
CUL5	cullin 5 [Source:HGNC Symbol;Acc:2556]	chr11	107879459	107978503	783	0.272	1	1	3.086E-02	4.469E-02	PB-GT
ESYT2	extended synaptotagmin-like protein 2 [Source:HGNC Symbol;Acc:22211]	chr7	158523686	158622944	732	0.272	1	1	3.088E-02	4.470E-02	PB-GT
TANK	TRAF family member-associated NFKB activator [Source:HGNC Symbol;Acc:11562]	chr2	161993419	162092732	952	0.272	1	1	3.097E-02	4.481E-02	PB-GT
RFC3	replication factor C (activator 1) 3, 38kDa [Source:HGNC Symbol;Acc:9971]	chr13	34392186	34540695	1122	0.272	1	1	3.101E-02	4.483E-02	PB-GT
INTS7	integrator complex subunit 7 [Source:HGNC Symbol;Acc:24484]	chr1	212113741	212208884	769	0.273	1	1	3.113E-02	4.498E-02	PB-GT
NSMCE2	non-SMC element 2, MMS21 homolog (S. cerevisiae) [Source:HGNC Symbol;Acc:26513]	chr8	126103921	126379362	1971	0.675	2	2	3.120E-02	4.507E-02	PB-GT
FER1L6-AS2	FER1L6 antisense RNA 2 [Source:HGNC Symbol;Acc:26534]	chr8	125058314	125183763	799	0.274	1	1	3.128E-02	4.516E-02	PB-GT
EPB41L4B	erythrocyte membrane protein band 4.1 like 4B [Source:HGNC Symbol;Acc:19818]	chr9	111934255	112083244	730	0.274	1	1	3.130E-02	4.517E-02	PB-GT
DNAH10	dynein, axonemal, heavy chain 10 [Source:HGNC Symbol;Acc:2941]	chr12	124247042	124420753	717	0.275	1	1	3.144E-02	4.535E-02	PB-GT
CCNY	cyclin Y [Source:HGNC Symbol;Acc:23354]	chr10	35535953	35860852	1899	0.678	2	1	3.149E-02	4.539E-02	PB-GT
AGK	acylglycerol kinase [Source:HGNC Symbol;Acc:21869]	chr7	141250989	141355044	742	0.276	1	1	3.166E-02	4.561E-02	PB-GT
GNAL	guanine nucleotide binding protein (G protein), alpha activating activity polypeptide, olfactory type [Source:HGNC Symbol;Acc:4	chr18	11688955	11885684	1152	0.276	1	1	3.174E-02	4.570E-02	PB-GT
EIF3E	eukaryotic translation initiation factor 3, subunit E [Source:HGNC Symbol;Acc:3277]	chr8	109213445	109447562	1986	0.680	2	2	3.180E-02	4.577E-02	PB-GT
NME7	NME/NM23 family member 7 [Source:HGNC Symbol;Acc:20461]	chr1	169101769	169337205	1917	0.681	2	1	3.181E-02	4.577E-02	PB-GT
PSME4	proteasome (prosome, macropain) activator subunit 4 [Source:HGNC Symbol;Acc:20635]	chr2	54091204	54197977	967	0.277	1	1	3.187E-02	4.582E-02	PB-GT
TNPO3	transportin 3 [Source:HGNC Symbol;Acc:17103]	chr7	128594948	128695198	745	0.277	1	1	3.189E-02	4.583E-02	PB-GT
PUM2	pumilio homolog 2 (Drosophila) [Source:HGNC Symbol;Acc:14958]	chr2	20448452	20551995	969	0.277	1	1	3.199E-02	4.595E-02	PB-GT
TWIST1	twist basic helix-loop-helix transcription factor 1 [Source:HGNC Symbol;Acc:12428]	chr7	19060614	19157295	748	0.278	1	1	3.212E-02	4.612E-02	PB-GT
TJP1	tight junction protein 1 [Source:HGNC Symbol;Acc:11827]	chr15	29991571	30261068	1967	0.684	2	2	3.223E-02	4.625E-02	PB-GT
RBM27	RNA binding motif protein 27 [Source:HGNC Symbol;Acc:29243]	chr5	145583113	145718814	1033	0.278	1	1	3.226E-02	4.627E-02	PB-GT
FBXO11	F-box protein 11 [Source:HGNC Symbol;Acc:13590]	chr2	48016455	48132932	974	0.279	1	1	3.229E-02	4.629E-02	PB-GT
PRKCB	protein kinase C, beta [Source:HGNC Symbol;Acc:9395]	chr16	23847322	24231932	2044	0.685	2	2	3.231E-02	4.630E-02	PB-GT
IQGAP1	IQ motif containing GTPase activating protein 1 [Source:HGNC Symbol;Acc:6110]	chr15	90931450	91045475	803	0.279	1	1	3.244E-02	4.646E-02	PB-GT
JAK1	Janus kinase 1 [Source:HGNC Symbol;Acc:6190]	chr1	65298912	65432187	788	0.280	1	1	3.254E-02	4.658E-02	PB-GT
KDM2A	lysine (K)-specific demethylase 2A [Source:HGNC Symbol;Acc:13606]	chr11	66886740	67025141	807	0.280	1	1	3.260E-02	4.665E-02	PB-GT
FBXW8	F-box and WD repeat domain containing 8 [Source:HGNC Symbol;Acc:13597]	chr12	117348761	117468953	732	0.280	1	1	3.265E-02	4.669E-02	PB-GT
TBC1D15	TBC1 domain family, member 15 [Source:HGNC Symbol;Acc:25694]	chr12	72233487	72320629	733	0.281	1	1	3.273E-02	4.679E-02	PB-GT
GLS	glutaminase [Source:HGNC Symbol;Acc:4331]	chr2	191745553	191830278	985	0.282	1	1	3.295E-02	4.708E-02	PB-GT
SLCO6A1	solute carrier organic anion transporter family, member 6A1 [Source:HGNC Symbol;Acc:23613]	chr5	101707486	101834720	1046	0.282	1	1	3.300E-02	4.712E-02	PB-GT
CFDP1	craniofacial development protein 1 [Source:HGNC Symbol;Acc:1873]	chr16	75327596	75467383	846	0.283	1	1	3.332E-02	4.755E-02	PB-GT
RPRD2	regulation of nuclear pre-mRNA domain containing 2 [Source:HGNC Symbol;Acc:29039]	chr1	150335567	150449042	800	0.284	1	1	3.345E-02	4.771E-02	PB-GT
PAM	peptidylglycine alpha-amidating monooxygenase [Source:HGNC Symbol;Acc:8596]	chr5	102089685	102366809	2583	0.696	2	2	3.367E-02	4.801E-02	PB-GT
ADIPOR2	adiponectin receptor 2 [Source:HGNC Symbol;Acc:24041]	chr12	1797740	1897844	746	0.286	1	1	3.379E-02	4.816E-02	PB-GT
PLS1	plastin 1 [Source:HGNC Symbol;Acc:9090]	chr3	142315229	142432506	929	0.286	1	1	3.383E-02	4.818E-02	PB-GT
ERI2	ERI1 exoribonuclease family member 2 [Source:HGNC Symbol;Acc:30541]	chr16	20791515	20911671	855	0.286	1	1	3.397E-02	4.836E-02	PB-GT
ASCC1	activating signal cointegrator 1 complex subunit 1 [Source:HGNC Symbol;Acc:24268]	chr10	73856278	73976892	803	0.287	1	1	3.400E-02	4.838E-02	PB-GT
KIAA1468	KIAA1468 [Source:HGNC Symbol;Acc:29289]	chr18	59854491	59974355	1201	0.288	1	1	3.423E-02	4.865E-02	PB-GT
WAC	WW domain containing adaptor with coiled-coil [Source:HGNC Symbol;Acc:17327]	chr10	28821422	28909928	806	0.288	1	1	3.423E-02	4.865E-02	PB-GT
ACVR1	activin A receptor, type I [Source:HGNC Symbol;Acc:171]	chr2	158592958	158732374	1006	0.288	1	1	3.424E-02	4.865E-02	PB-GT
PHF20L1	PHD finger protein 20-like 1 [Source:HGNC Symbol;Acc:24280]	chr8	133787618	133861052	841	0.288	1	1	3.433E-02	4.876E-02	PB-GT
TNPO1	transportin 1 [Source:HGNC Symbol;Acc:6401]	chr5	72112139	72212560	1070	0.288	1	1	3.439E-02	4.881E-02	PB-GT
NRG1-IT2	NRG1 intronic transcript 2 (non-protein coding) [Source:HGNC Symbol;Acc:43634]	chr8	32028898	32145082	843	0.289	1	1	3.448E-02	4.892E-02	PB-GT
CYB5R4	cytochrome b5 reductase 4 [Source:HGNC Symbol;Acc:20147]	chr6	84569362	84670146	841	0.289	1	1	3.450E-02	4.892E-02	PB-GT
ANKRD36C	ankyrin repeat domain 36C [Source:HGNC Symbol;Acc:32946]	chr2	96514587	96657541	1012	0.289	1	1	3.461E-02	4.905E-02	PB-GT
ZHX2	zinc fingers and homeoboxes 2 [Source:HGNC Symbol;Acc:18513]	chr8	123793633	123986750	847	0.290	1	1	3.478E-02	4.927E-02	PB-GT
CDK6	cyclin-dependent kinase 6 [Source:HGNC Symbol;Acc:1777]	chr7	92234235	92465908	1900	0.706	2	1	3.482E-02	4.930E-02	PB-GT
KITLG	KIT ligand [Source:HGNC Symbol;Acc:6343]	chr12	88886570	88974628	759	0.291	1	1	3.487E-02	4.935E-02	PB-GT
FXR1	fragile X mental retardation, autosomal homolog 1 [Source:HGNC Symbol;Acc:4023]	chr3	180585929	180694950	946	0.291	1	1	3.496E-02	4.945E-02	PB-GT
ZNF114	zinc finger protein 114 [Source:HGNC Symbol;Acc:12894]	chr19	48675575	48790865	553	0.292	1	1	3.513E-02	4.967E-02	PB-GT
VPS13C	vacuolar protein sorting 13 homolog C (S. cerevisiae) [Source:HGNC Symbol;Acc:23594]	chr15	62144588	62352672	2037	0.708	2	1	3.517E-02	4.970E-02	PB-GT
ANAPC10	anaphase promoting complex subunit 10 [Source:HGNC Symbol;Acc:24077]	chr4	145888264	146019693	1033	0.292	1	1	3.528E-02	4.983E-02	PB-GT
CGGBP1	CGG triplet repeat binding protein 1 [Source:HGNC Symbol;Acc:1888]	chr3	88101094	88199035	953	0.293	1	1	3.543E-02	4.999E-02	PB-GT
FAM81A	family with sequence similarity 81, member A [Source:HGNC Symbol;Acc:28379]	chr15	59664892	59815748	843	0.293	1	1	3.543E-02	4.999E-02	PB-GT
C1orf122	chromosome 1 open reading frame 122 [Source:HGNC Symbol;Acc:24789]	1	38267651	38275126	32	0.004	3	1	9.377E-12	1.129E-08	PB-CAG-SD
YRDC	yrdC domain containing (E. coli) [Source:HGNC Symbol;Acc:28905]	1	38268616	38278857	50	0.006	3	1	5.579E-11	3.359E-08	PB-CAG-SD
SLC25A6P6	solute carrier family 25 (mitochondrial carrier; adenine nucleotide translocator), member 6 pseudogene 6 [Source:HGNC Symbo	6	71373205	71378982	33	0.003	2	1	5.855E-09	2.350E-06	PB-CAG-SD
RNU6-706P	RNA, U6 small nuclear 706, pseudogene [Source:HGNC Symbol;Acc:47669]	18	36608971	36614074	39	0.004	2	1	9.614E-09	2.894E-06	PB-CAG-SD
PBXIP1	pre-B-cell leukemia homeobox interacting protein 1 [Source:HGNC Symbol;Acc:21199]	1	154916552	154933599	36	0.004	2	1	1.378E-08	3.317E-06	PB-CAG-SD
ZC3H13	zinc finger CCCH-type containing 13 [Source:HGNC Symbol;Acc:20368]	13	46528600	46631894	1008	0.087	4	1	3.867E-08	6.924E-06	PB-CAG-SD
BCOR	BCL6 corepressor [Source:HGNC Symbol;Acc:20893]	X	39909068	40041582	478	0.033	3	1	4.694E-08	6.924E-06	PB-CAG-SD
HSPA5	heat shock 70kDa protein 5 (glucose-regulated protein, 78kDa) [Source:HGNC Symbol;Acc:5238]	9	127997132	128008609	67	0.007	2	1	4.777E-08	6.924E-06	PB-CAG-SD
RER1	RER1 retention in endoplasmic reticulum 1 homolog (S. cerevisiae) [Source:HGNC Symbol;Acc:30309]	1	2318267	2336883	56	0.007	2	1	5.176E-08	6.924E-06	PB-CAG-SD
RNA5S16	RNA, 5S ribosomal 16 [Source:HGNC Symbol;Acc:34377]	1	228779556	228784674	3	0.000	1	1	6.601E-08	7.948E-06	PB-CAG-SD
SRSF2	serine/arginine-rich splicing factor 2 [Source:HGNC Symbol;Acc:10783]	17	74730197	74738456	52	0.008	2	1	8.386E-08	9.179E-06	PB-CAG-SD
RBM23	RNA binding motif protein 23 [Source:HGNC Symbol;Acc:20155]	14	23369854	23393393	91	0.010	2	1	1.636E-07	1.642E-05	PB-CAG-SD
PIGS	phosphatidylinositol glycan anchor biosynthesis, class S [Source:HGNC Symbol;Acc:14937]	17	26880401	26903890	71	0.011	2	1	2.130E-07	1.973E-05	PB-CAG-SD
RBM15	RNA binding motif protein 15 [Source:HGNC Symbol;Acc:14959]	1	110876128	110889299	110	0.013	2	1	3.903E-07	3.157E-05	PB-CAG-SD
FBXW5	F-box and WD repeat domain containing 5 [Source:HGNC Symbol;Acc:13613]	9	139834887	139844148	9	0.001	1	1	3.933E-07	3.157E-05	PB-CAG-SD
FAM53A	family with sequence similarity 53, member A [Source:HGNC Symbol;Acc:31860]	4	1619642	1691029	155	0.014	2	1	4.218E-07	3.174E-05	PB-CAG-SD
RAP2C	RAP2C, member of RAS oncogene family [Source:HGNC Symbol;Acc:21165]	X	131337053	131358471	222	0.015	2	1	5.821E-07	4.123E-05	PB-CAG-SD
LCN12	lipocalin 12 [Source:HGNC Symbol;Acc:28733]	9	139839003	139849949	12	0.001	1	1	6.990E-07	4.676E-05	PB-CAG-SD
ZKSCAN8	zinc finger with KRAB and SCAN domains 8 [Source:HGNC Symbol;Acc:12983]	6	28104688	28127250	168	0.017	2	1	7.648E-07	4.846E-05	PB-CAG-SD
RPS29	ribosomal protein S29 [Source:HGNC Symbol;Acc:10419]	14	50043390	50070408	158	0.017	2	1	8.518E-07	5.088E-05	PB-CAG-SD
BCL6	B-cell CLL/lymphoma 6 [Source:HGNC Symbol;Acc:1001]	3	187439165	187468515	147	0.018	2	2	8.874E-07	5.088E-05	PB-CAG-SD
PEX10	peroxisomal biogenesis factor 10 [Source:HGNC Symbol;Acc:8851]	1	2336236	2350236	13	0.002	1	1	1.239E-06	6.499E-05	PB-CAG-SD
MIR608	microRNA 608 [Source:HGNC Symbol;Acc:32864]	10	102729742	102734841	14	0.002	1	1	1.277E-06	6.499E-05	PB-CAG-SD
AGTPBP1	ATP/GTP binding protein 1 [Source:HGNC Symbol;Acc:17258]	9	88161455	88361944	1809	0.178	4	2	1.295E-06	6.499E-05	PB-CAG-SD
LCN6	lipocalin 6 [Source:HGNC Symbol;Acc:17337]	9	139638463	139647980	17	0.002	1	1	1.402E-06	6.754E-05	PB-CAG-SD
MIR1237	microRNA 1237 [Source:HGNC Symbol;Acc:33927]	11	64131074	64136175	20	0.002	1	1	1.514E-06	7.010E-05	PB-CAG-SD
NCAPD2	non-SMC condensin I complex, subunit D2 [Source:HGNC Symbol;Acc:24305]	12	6597522	6641121	186	0.022	2	1	1.638E-06	7.089E-05	PB-CAG-SD
RNA5S17	RNA, 5S ribosomal 17 [Source:HGNC Symbol;Acc:34378]	1	228781787	228786905	15	0.002	1	1	1.649E-06	7.089E-05	PB-CAG-SD
ASAP3	ArfGAP with SH3 domain, ankyrin repeat and PH domain 3 [Source:HGNC Symbol;Acc:14987]	1	23755056	23815685	187	0.023	2	1	1.904E-06	7.907E-05	PB-CAG-SD
MADD	MAP-kinase activating death domain [Source:HGNC Symbol;Acc:6766]	11	47285712	47351582	270	0.024	2	1	2.126E-06	8.197E-05	PB-CAG-SD
RNU6-600P	RNA, U6 small nuclear 600, pseudogene [Source:HGNC Symbol;Acc:47563]	12	49285913	49291011	18	0.002	1	1	2.168E-06	8.197E-05	PB-CAG-SD
ZNF25	zinc finger protein 25 [Source:HGNC Symbol;Acc:13043]	10	38238500	38270561	209	0.024	2	1	2.226E-06	8.197E-05	PB-CAG-SD
CHCHD2P2	coiled-coil-helix-coiled-coil-helix domain containing 2 pseudogene 2 [Source:HGNC Symbol;Acc:39586]	5	68624756	68630076	20	0.002	1	1	2.254E-06	8.197E-05	PB-CAG-SD
IGLV3-7	immunoglobulin lambda variable 3-7 (pseudogene) [Source:HGNC Symbol;Acc:5917]	22	23175779	23181049	18	0.002	1	1	2.315E-06	8.197E-05	PB-CAG-SD
RN7SKP92	RNA, 7SK small nuclear pseudogene 92 [Source:HGNC Symbol;Acc:45816]	14	100948881	100954124	20	0.002	1	1	2.394E-06	8.236E-05	PB-CAG-SD
RN7SKP279	RNA, 7SK small nuclear pseudogene 279 [Source:HGNC Symbol;Acc:46003]	11	127272746	127278043	26	0.002	1	1	2.558E-06	8.369E-05	PB-CAG-SD
PVRL2	poliovirus receptor-related 2 (herpesvirus entry mediator B) [Source:HGNC Symbol;Acc:9707]	19	45344432	45392485	115	0.025	2	1	2.572E-06	8.369E-05	PB-CAG-SD
SNORD96A	small nucleolar RNA, C/D box 96A [Source:HGNC Symbol;Acc:32758]	5	180668814	180673892	22	0.002	1	1	2.727E-06	8.435E-05	PB-CAG-SD
RN7SL20P	RNA, 7SL, cytoplasmic 20, pseudogene [Source:HGNC Symbol;Acc:46036]	22	32148188	32153482	20	0.002	1	1	2.857E-06	8.435E-05	PB-CAG-SD
RPL12P2	ribosomal protein L12 pseudogene 2 [Source:HGNC Symbol;Acc:16070]	6	37054088	37059582	25	0.002	1	1	3.078E-06	8.435E-05	PB-CAG-SD
REM2	RAS (RAD and GEM)-like GTP binding 2 [Source:HGNC Symbol;Acc:20248]	14	23347374	23356895	23	0.003	1	1	3.166E-06	8.435E-05	PB-CAG-SD

table S2. Genes with significant transposon integrations in RKO *BRAF*$^{wt/-/-}$ cells after selection in low glucose medium

Gene ID	Description	Chrom	Start	End	# TTAA sites	Expected	Observed	Libraries	*P*	FDR	Transposon
RNA5SP18	RNA, 5S ribosomal pseudogene 18 [Source:HGNC Symbol;Acc:42586]	1	228783659	228788779	21	0.003	1	1	3.230E-06	8.435E-05	PB-CAG-SD
GAPDH	glyceraldehyde-3-phosphate dehydrogenase [Source:HGNC Symbol;Acc:4141]	12	6638093	6647537	22	0.003	1	1	3.238E-06	8.435E-05	PB-CAG-SD
RNU6-1148P	RNA, U6 small nuclear 1148, pseudogene [Source:HGNC Symbol;Acc:48111]	5	137405044	137410147	24	0.003	1	1	3.245E-06	8.435E-05	PB-CAG-SD
C16orf98	chromosome 16 open reading frame 98 [Source:HGNC Symbol;Acc:45036]	16	31208206	31214773	20	0.003	1	1	3.403E-06	8.435E-05	PB-CAG-SD
RPS6KA4	ribosomal protein S6 kinase, 90kDa, polypeptide 4 [Source:HGNC Symbol;Acc:10433]	11	64121620	64139687	30	0.003	1	1	3.404E-06	8.435E-05	PB-CAG-SD
PEX2	peroxisomal biogenesis factor 2 [Source:HGNC Symbol;Acc:9717]	8	77892494	77918280	224	0.028	2	1	3.439E-06	8.435E-05	PB-CAG-SD
C5orf55	chromosome 5 open reading frame 55 [Source:HGNC Symbol;Acc:25175]	5	441645	448258	25	0.003	1	1	3.521E-06	8.435E-05	PB-CAG-SD
RNU6-510P	RNA, U6 small nuclear 510, pseudogene [Source:HGNC Symbol;Acc:47473]	1	38452134	38457241	22	0.003	1	1	3.544E-06	8.435E-05	PB-CAG-SD
LDHBP2	lactate dehydrogenase B pseudogene 2 [Source:HGNC Symbol;Acc:6543]	X	75555240	75561244	39	0.003	1	1	3.573E-06	8.435E-05	PB-CAG-SD
CITED1	Cbp/p300-interacting transactivator, with Glu/Asp-rich carboxy-terminal domain, 1 [Source:HGNC Symbol;Acc:1986]	X	71521488	71532037	39	0.003	1	1	3.573E-06	8.435E-05	PB-CAG-SD
HMGN2P22	high mobility group nucleosomal binding domain 2 pseudogene 22 [Source:HGNC Symbol;Acc:39387]	2	101207612	101212786	31	0.003	1	1	3.980E-06	8.705E-05	PB-CAG-SD
RNU6-795P	RNA, U6 small nuclear 795, pseudogene [Source:HGNC Symbol;Acc:47758]	10	38332801	38337903	25	0.003	1	1	4.068E-06	8.705E-05	PB-CAG-SD
RNA5-8SP2	RNA, 5.8S ribosomal pseudogene 2 [Source:HGNC Symbol;Acc:41956]	16	33960426	33965577	22	0.003	1	1	4.116E-06	8.705E-05	PB-CAG-SD
CDIPT-AS1	CDIPT antisense RNA 1 (head to head) [Source:HGNC Symbol;Acc:48609]	16	29869914	29879371	22	0.003	1	1	4.116E-06	8.705E-05	PB-CAG-SD
RN7SKP121	RNA, 7SK small nuclear pseudogene 121 [Source:HGNC Symbol;Acc:45845]	11	127275923	127281227	33	0.003	1	1	4.118E-06	8.705E-05	PB-CAG-SD
RNU7-14P	RNA, U7 small nuclear 14 pseudogene [Source:HGNC Symbol;Acc:34110]	20	52285236	52290297	20	0.003	1	1	4.121E-06	8.705E-05	PB-CAG-SD
DUSP5P1	dual specificity phosphatase 5 pseudogene 1 [Source:HGNC Symbol;Acc:32020]	1	228739885	228788150	24	0.003	1	1	4.218E-06	8.755E-05	PB-CAG-SD
TIMM8B	translocase of inner mitochondrial membrane 8 homolog B (yeast) [Source:HGNC Symbol;Acc:11818]	11	111955524	111962522	35	0.003	1	1	4.632E-06	9.176E-05	PB-CAG-SD
C9orf50	chromosome 9 open reading frame 50 [Source:HGNC Symbol;Acc:23677]	9	132374504	132388055	31	0.003	1	1	4.659E-06	9.176E-05	PB-CAG-SD
DDX39BP2	DEAD (Asp-Glu-Ala-Asp) box polypeptide 39B pseudogene 2 [Source:HGNC Symbol;Acc:33461]	6	29955986	29961382	31	0.003	1	1	4.731E-06	9.176E-05	PB-CAG-SD
SNORD101	small nucleolar RNA, C/D box 101 [Source:HGNC Symbol;Acc:32764]	6	133131446	133136518	31	0.003	1	1	4.731E-06	9.176E-05	PB-CAG-SD
MST1	macrophage stimulating 1 (hepatocyte growth factor-like) [Source:HGNC Symbol;Acc:7380]	3	49721380	49731934	26	0.003	1	1	4.801E-06	9.176E-05	PB-CAG-SD
GAPDHP34	glyceraldehyde 3 phosphate dehydrogenase pseudogene 34 [Source:HGNC Symbol;Acc:37786]	13	37309178	37315000	37	0.003	1	1	5.090E-06	9.364E-05	PB-CAG-SD
C11orf31	chromosome 11 open reading frame 31 [Source:HGNC Symbol;Acc:18251]	11	57503825	57510986	37	0.003	1	1	5.176E-06	9.364E-05	PB-CAG-SD
SNRNP25	small nuclear ribonucleoprotein 25kDa (U11/U12) [Source:HGNC Symbol;Acc:14161]	16	98010	107669	25	0.003	1	1	5.314E-06	9.364E-05	PB-CAG-SD
CDIPT	CDP-diacylglycerol--inositol 3-phosphatidyltransferase [Source:HGNC Symbol;Acc:1769]	16	29869678	29880057	25	0.003	1	1	5.314E-06	9.364E-05	PB-CAG-SD
USE1	unconventional SNARE in the ER 1 homolog (S. cerevisiae) [Source:HGNC Symbol;Acc:30882]	19	17321155	17330638	15	0.003	1	1	5.327E-06	9.364E-05	PB-CAG-SD
CTBP2P5	C-terminal binding protein 2 pseudogene 5 [Source:HGNC Symbol;Acc:45197]	2	49142406	49148669	36	0.003	1	1	5.366E-06	9.364E-05	PB-CAG-SD
SNORD100	small nucleolar RNA, C/D box 100 [Source:HGNC Symbol;Acc:32763]	6	133132941	133138016	34	0.003	1	1	5.690E-06	9.786E-05	PB-CAG-SD
RNU6-128P	RNA, U6 small nuclear 128, pseudogene [Source:HGNC Symbol;Acc:47091]	4	159715742	159720848	39	0.003	1	1	5.905E-06	1.001E-04	PB-CAG-SD
MIR4771-2	microRNA 4771-2 [Source:HGNC Symbol;Acc:41774]	2	112528638	112533711	39	0.004	1	1	6.297E-06	1.053E-04	PB-CAG-SD
RNU7-158P	RNA, U7 small nuclear 158 pseudogene [Source:HGNC Symbol;Acc:45692]	4	184090501	184095570	43	0.004	1	1	7.177E-06	1.184E-04	PB-CAG-SD
HIST1H1C	histone cluster 1, H1c [Source:HGNC Symbol;Acc:4716]	6	26055968	26061699	39	0.004	1	1	7.484E-06	1.203E-04	PB-CAG-SD
OR10R3P	olfactory receptor, family 10, subfamily R, member 3 pseudogene [Source:HGNC Symbol;Acc:14829]	1	158456009	158461950	32	0.004	1	1	7.493E-06	1.203E-04	PB-CAG-SD
MIR4754	microRNA 4754 [Source:HGNC Symbol;Acc:41786]	19	58898137	58903225	18	0.004	1	1	7.668E-06	1.215E-04	PB-CAG-SD
CA14	carbonic anhydrase XIV [Source:HGNC Symbol;Acc:1372]	1	150224554	150237478	33	0.004	1	1	7.968E-06	1.227E-04	PB-CAG-SD
RAB26	RAB26, member RAS oncogene family [Source:HGNC Symbol;Acc:14259]	16	2185804	2204166	31	0.004	1	1	8.167E-06	1.227E-04	PB-CAG-SD
RPL22P19	ribosomal protein L22 pseudogene 19 [Source:HGNC Symbol;Acc:36567]	12	125420001	125425441	35	0.004	1	1	8.188E-06	1.227E-04	PB-CAG-SD
GLYCTK	glycerate kinase [Source:HGNC Symbol;Acc:24247]	3	52316105	52329272	34	0.004	1	1	8.206E-06	1.227E-04	PB-CAG-SD
RNU6-687P	RNA, U6 small nuclear 687, pseudogene [Source:HGNC Symbol;Acc:47650]	10	56212183	56217271	36	0.004	1	1	8.428E-06	1.227E-04	PB-CAG-SD
USP21	ubiquitin specific peptidase 21 [Source:HGNC Symbol;Acc:12620]	1	161124240	161135513	34	0.004	1	1	8.458E-06	1.227E-04	PB-CAG-SD
ATP5G1	ATP synthase, H+ transporting, mitochondrial Fo complex, subunit C1 (subunit 9) [Source:HGNC Symbol;Acc:841]	17	46965127	46973233	27	0.004	1	1	8.538E-06	1.227E-04	PB-CAG-SD
ATP5G1P5	ATP synthase, H+ transporting, mitochondrial Fo complex, subunit C1 (subunit 9) pseudogene 5 [Source:HGNC Symbol;Acc:39	13	107310785	107316195	48	0.004	1	1	8.561E-06	1.227E-04	PB-CAG-SD
SNORA33	small nucleolar RNA, H/ACA box 33 [Source:HGNC Symbol;Acc:32623]	6	133133358	133138487	42	0.004	1	1	8.677E-06	1.228E-04	PB-CAG-SD
ATP6V1G1P4	ATPase, H+ transporting, lysosomal 13kDa, V1 subunit G1 pseudogene 4 [Source:HGNC Symbol;Acc:37671]	10	35442234	35447568	37	0.004	1	1	8.902E-06	1.228E-04	PB-CAG-SD
KRT18P13	keratin 18 pseudogene 13 [Source:HGNC Symbol;Acc:6432]	9	100456204	100463016	43	0.004	1	1	8.958E-06	1.228E-04	PB-CAG-SD
NUDT19P4	nudix (nucleoside diphosphate linked moiety X)-type motif 19 pseudogene 4 [Source:HGNC Symbol;Acc:43588]	6	44861448	44867032	43	0.004	1	1	9.095E-06	1.228E-04	PB-CAG-SD
MRPL51	mitochondrial ribosomal protein L51 [Source:HGNC Symbol;Acc:14044]	12	6601150	6608007	37	0.004	1	1	9.149E-06	1.228E-04	PB-CAG-SD
GALR2	galanin receptor 2 [Source:HGNC Symbol;Acc:4133]	17	74065875	74073622	28	0.004	1	1	9.182E-06	1.228E-04	PB-CAG-SD
RN7SL653P	RNA, 7SL, cytoplasmic 653, pseudogene [Source:HGNC Symbol;Acc:46669]	1	92295333	92300631	36	0.004	1	1	9.480E-06	1.241E-04	PB-CAG-SD
PPOX	protoporphyrinogen oxidase [Source:HGNC Symbol;Acc:9280]	1	161131200	161147803	36	0.004	1	1	9.480E-06	1.241E-04	PB-CAG-SD
PLEKHG6	pleckstrin homology domain containing, family G (with RhoGef domain) member 6 [Source:HGNC Symbol;Acc:25562]	12	6414602	6437672	38	0.004	1	1	9.649E-06	1.249E-04	PB-CAG-SD
HIST1H2AG	histone cluster 1, H2ag [Source:HGNC Symbol;Acc:4737]	6	27095832	27103070	45	0.004	1	1	9.959E-06	1.260E-04	PB-CAG-SD
TMED5	transmembrane emp24 protein transport domain containing 5 [Source:HGNC Symbol;Acc:24251]	1	93615299	93651285	326	0.039	2	1	9.964E-06	1.260E-04	PB-CAG-SD
RPPH1-2P	ribonuclease P RNA component H1, 2 pseudogene [Source:HGNC Symbol;Acc:47029]	14	65376206	65381498	41	0.004	1	1	1.005E-05	1.260E-04	PB-CAG-SD
RNU6-137P	RNA, U6 small nuclear 137, pseudogene [Source:HGNC Symbol;Acc:47100]	2	42934880	42939987	50	0.005	1	1	1.034E-05	1.273E-04	PB-CAG-SD
RNU1-150P	RNA, U1 small nuclear 150, pseudogene [Source:HGNC Symbol;Acc:48492]	5	40264660	40269821	43	0.005	1	1	1.040E-05	1.273E-04	PB-CAG-SD
SH2D5	SH2 domain containing 5 [Source:HGNC Symbol;Acc:28819]	1	21046225	21064330	38	0.005	1	1	1.056E-05	1.273E-04	PB-CAG-SD
LINC00113	long intergenic non-protein coding RNA 113 [Source:HGNC Symbol;Acc:1264]	21	29089698	29123552	278	0.040	2	1	1.062E-05	1.273E-04	PB-CAG-SD
OR11J2P	olfactory receptor, family 11, subfamily J, member 2 pseudogene [Source:HGNC Symbol;Acc:15370]	15	21161004	21166910	45	0.005	1	1	1.078E-05	1.273E-04	PB-CAG-SD
TUBB8P8	tubulin, beta 8 class VIII pseudogene 8 [Source:HGNC Symbol;Acc:42346]	3	197841592	197847649	39	0.005	1	1	1.079E-05	1.273E-04	PB-CAG-SD
RPL29P14	ribosomal protein L29 pseudogene 14 [Source:HGNC Symbol;Acc:36687]	5	148924946	148930373	44	0.005	1	1	1.089E-05	1.273E-04	PB-CAG-SD
ANO7P1	anoctamin 7 pseudogene 1 [Source:HGNC Symbol;Acc:32248]	1	16542404	16559522	39	0.005	1	1	1.112E-05	1.288E-04	PB-CAG-SD
OTUD1	OTU domain containing 1 [Source:HGNC Symbol;Acc:27346]	10	23723198	23731308	42	0.005	1	1	1.147E-05	1.315E-04	PB-CAG-SD
HIST1H2BF	histone cluster 1, H2bf [Source:HGNC Symbol;Acc:4752]	6	26194748	26200942	49	0.005	1	1	1.181E-05	1.341E-04	PB-CAG-SD
RNU6-431P	RNA, U6 small nuclear 431, pseudogene [Source:HGNC Symbol;Acc:47394]	4	109568306	109573412	56	0.005	1	1	1.216E-05	1.355E-04	PB-CAG-SD
ZNF227	zinc finger protein 227 [Source:HGNC Symbol;Acc:13020]	19	44706700	44741421	194	0.042	2	1	1.219E-05	1.355E-04	PB-CAG-SD
VN1R65P	vomeronasal 1 receptor 65 pseudogene [Source:HGNC Symbol;Acc:37385]	16	31560426	31566233	38	0.005	1	1	1.226E-05	1.355E-04	PB-CAG-SD
LSM7	LSM7 homolog, U6 small nuclear RNA associated (S. cerevisiae) [Source:HGNC Symbol;Acc:20470]	19	2321520	2333615	23	0.005	1	1	1.251E-05	1.369E-04	PB-CAG-SD
POLR3K	polymerase (RNA) III (DNA directed) polypeptide K, 12.3 kDa [Source:HGNC Symbol;Acc:14121]	16	96407	108628	39	0.005	1	1	1.292E-05	1.401E-04	PB-CAG-SD
MFSD11	major facilitator superfamily domain containing 11 [Source:HGNC Symbol;Acc:25458]	17	74726947	74777531	283	0.043	2	1	1.316E-05	1.405E-04	PB-CAG-SD
MICA	MHC class I polypeptide-related sequence A [Source:HGNC Symbol;Acc:7090]	6	31366356	31383092	52	0.005	1	1	1.329E-05	1.405E-04	PB-CAG-SD
MIR628	microRNA 628 [Source:HGNC Symbol;Acc:32884]	15	55665138	55670232	50	0.005	1	1	1.330E-05	1.405E-04	PB-CAG-SD
SRRT	serrate RNA effector molecule homolog (Arabidopsis) [Source:HGNC Symbol;Acc:24101]	7	100467733	100486285	44	0.005	1	1	1.388E-05	1.454E-04	PB-CAG-SD
PRMT5-AS1	PRMT5 antisense RNA 1 [Source:HGNC Symbol;Acc:40533]	14	23383665	23396105	49	0.005	1	1	1.434E-05	1.473E-04	PB-CAG-SD
ATP6V0E2-AS1	ATP6V0E2 antisense RNA 1 [Source:HGNC Symbol;Acc:44180]	7	149564786	149582699	45	0.005	1	1	1.452E-05	1.473E-04	PB-CAG-SD
LTBR	lymphotoxin beta receptor (TNFR superfamily, member 3) [Source:HGNC Symbol;Acc:6718]	12	6479211	6500733	47	0.005	1	1	1.475E-05	1.473E-04	PB-CAG-SD
EPOR	erythropoietin receptor [Source:HGNC Symbol;Acc:3416]	19	11488236	11500018	25	0.005	1	1	1.478E-05	1.473E-04	PB-CAG-SD
RSL24D1P8	ribosomal L24 domain containing 1 pseudogene 8 [Source:HGNC Symbol;Acc:37873]	19	12202239	12207730	25	0.005	1	1	1.478E-05	1.473E-04	PB-CAG-SD
CYR61	cysteine-rich, angiogenic inducer, 61 [Source:HGNC Symbol;Acc:2654]	1	86041444	86049645	45	0.005	1	1	1.480E-05	1.473E-04	PB-CAG-SD
MAD2L1BP	MAD2L1 binding protein [Source:HGNC Symbol;Acc:21059]	6	43592277	43608689	56	0.006	1	1	1.541E-05	1.521E-04	PB-CAG-SD
TRIM26BP	tripartite motif containing 26B, pseudogene [Source:HGNC Symbol;Acc:31338]	6	30201078	30210056	57	0.006	1	1	1.597E-05	1.555E-04	PB-CAG-SD
MIR21	microRNA 21 [Source:HGNC Symbol;Acc:31586]	17	57913627	57918698	37	0.006	1	1	1.602E-05	1.555E-04	PB-CAG-SD
AJUBA	ajuba LIM protein [Source:HGNC Symbol;Acc:20250]	14	23440383	23456851	53	0.006	1	1	1.677E-05	1.594E-04	PB-CAG-SD
LGALS8-AS1	LGALS8 antisense RNA 1 [Source:HGNC Symbol;Acc:40340]	1	236686369	236692808	48	0.006	1	1	1.684E-05	1.594E-04	PB-CAG-SD
MRPL43	mitochondrial ribosomal protein L43 [Source:HGNC Symbol;Acc:14517]	10	102729215	102752272	51	0.006	1	1	1.690E-05	1.594E-04	PB-CAG-SD
KANK4	KN motif and ankyrin repeat domains 4 [Source:HGNC Symbol;Acc:27263]	1	62702651	62790085	390	0.047	2	1	1.696E-05	1.594E-04	PB-CAG-SD
T	T, brachyury homolog (mouse) [Source:HGNC Symbol;Acc:11515]	6	166571144	166587188	59	0.006	1	1	1.710E-05	1.594E-04	PB-CAG-SD
DLX5	distal-less homeobox 5 [Source:HGNC Symbol;Acc:2918]	7	96649704	96659409	49	0.006	1	1	1.721E-05	1.594E-04	PB-CAG-SD
RHEB	Ras homolog enriched in brain [Source:HGNC Symbol;Acc:10011]	7	151163098	151222206	397	0.048	2	1	1.738E-05	1.597E-04	PB-CAG-SD
LINC00525	long intergenic non-protein coding RNA 525 [Source:HGNC Symbol;Acc:40290]	7	47796074	47806370	50	0.006	1	1	1.792E-05	1.603E-04	PB-CAG-SD
GART	phosphoribosylglycinamide formyltransferase, phosphoribosylglycinamide synthetase, phosphoribosylaminoimidazole synthetas	21	34876238	34920797	333	0.048	2	2	1.815E-05	1.603E-04	PB-CAG-SD
C21orf91	chromosome 21 open reading frame 91 [Source:HGNC Symbol;Acc:16459]	21	19161284	19196703	333	0.048	2	2	1.815E-05	1.603E-04	PB-CAG-SD
RPS12	ribosomal protein S12 [Source:HGNC Symbol;Acc:10385]	6	133130580	133138703	61	0.006	1	1	1.828E-05	1.603E-04	PB-CAG-SD
SFRP2	secreted frizzled-related protein 2 [Source:HGNC Symbol;Acc:10777]	4	154701744	154715272	69	0.006	1	1	1.845E-05	1.603E-04	PB-CAG-SD
SRSF8	serine/arginine-rich splicing factor 8 [Source:HGNC Symbol;Acc:16988]	11	94795056	94804388	70	0.006	1	1	1.849E-05	1.603E-04	PB-CAG-SD
EHD1	EH-domain containing 1 [Source:HGNC Symbol;Acc:3242]	11	64619114	64660768	70	0.006	1	1	1.849E-05	1.603E-04	PB-CAG-SD
MIR3175	microRNA 3175 [Source:HGNC Symbol;Acc:38299]	15	93442629	93447705	59	0.006	1	1	1.851E-05	1.603E-04	PB-CAG-SD
TMEM204	transmembrane protein 204 [Source:HGNC Symbol;Acc:14158]	16	1573689	1605581	47	0.006	1	1	1.875E-05	1.612E-04	PB-CAG-SD
HIST1H3D	histone cluster 1, H3d [Source:HGNC Symbol;Acc:4767]	6	26197068	26204521	62	0.006	1	1	1.888E-05	1.613E-04	PB-CAG-SD
NTMT1	N-terminal Xaa-Pro-Lys N-methyltransferase 1 [Source:HGNC Symbol;Acc:23373]	9	132366163	132398209	63	0.006	1	1	1.920E-05	1.628E-04	PB-CAG-SD
ICOSLG	inducible T-cell co-stimulator ligand [Source:HGNC Symbol;Acc:17087]	21	45642874	45665849	43	0.006	1	1	1.939E-05	1.633E-04	PB-CAG-SD
RPS5	ribosomal protein S5 [Source:HGNC Symbol;Acc:10426]	19	58892767	58906173	29	0.006	1	1	1.987E-05	1.650E-04	PB-CAG-SD
DDX39A	DEAD (Asp-Glu-Ala-Asp) box polypeptide 39A [Source:HGNC Symbol;Acc:17821]	19	14519631	14535192	29	0.006	1	1	1.987E-05	1.650E-04	PB-CAG-SD
PDP1	pyruvate dehyrogenase phosphatase catalytic subunit 1 [Source:HGNC Symbol;Acc:9279]	8	94865035	94938294	407	0.050	2	2	2.028E-05	1.663E-04	PB-CAG-SD
KRTAP27-1	keratin associated protein 27-1 [Source:HGNC Symbol;Acc:33864]	21	31709331	31715012	44	0.006	1	1	2.030E-05	1.663E-04	PB-CAG-SD
HIST1H4I	histone cluster 1, H4i [Source:HGNC Symbol;Acc:4793]	6	27102076	27108418	65	0.006	1	1	2.075E-05	1.688E-04	PB-CAG-SD
HMGB3P22	high mobility group box 3 pseudogene 22 [Source:HGNC Symbol;Acc:39314]	5	179101033	179121782	61	0.006	1	1	2.091E-05	1.689E-04	PB-CAG-SD
RNF123	ring finger protein 123 [Source:HGNC Symbol;Acc:21148]	3	49721932	49758962	55	0.007	1	1	2.144E-05	1.721E-04	PB-CAG-SD
ZACN	zinc activated ligand-gated ion channel [Source:HGNC Symbol;Acc:29504]	17	74063042	74079747	44	0.007	1	1	2.264E-05	1.805E-04	PB-CAG-SD
SON	SON DNA binding protein [Source:HGNC Symbol;Acc:11183]	21	34909924	34949812	361	0.052	2	2	2.305E-05	1.826E-04	PB-CAG-SD
ING2	inhibitor of growth family, member 2 [Source:HGNC Symbol;Acc:6063]	4	184421147	184432249	78	0.007	1	1	2.357E-05	1.855E-04	PB-CAG-SD
GNB2L1	guanine nucleotide binding protein (G protein), beta polypeptide 2-like 1 [Source:HGNC Symbol;Acc:4399]	5	180663909	180680096	66	0.007	1	1	2.447E-05	1.913E-04	PB-CAG-SD
CSRNP1	cysteine-serine-rich nuclear protein 1 [Source:HGNC Symbol;Acc:14300]	3	39183346	39201053	60	0.007	1	1	2.550E-05	1.981E-04	PB-CAG-SD
TMEM160	transmembrane protein 160 [Source:HGNC Symbol;Acc:26042]	19	47549165	47556888	33	0.007	1	1	2.572E-05	1.985E-04	PB-CAG-SD
ORAI2	ORAI calcium release-activated calcium modulator 2 [Source:HGNC Symbol;Acc:21667]	7	102068553	102097268	61	0.007	1	1	2.665E-05	2.044E-04	PB-CAG-SD
TIRAP	toll-interleukin 1 receptor (TIR) domain containing adaptor protein [Source:HGNC Symbol;Acc:17192]	11	126147960	126168740	85	0.007	1	1	2.724E-05	2.076E-04	PB-CAG-SD
PPP2R4	protein phosphatase 2A activator, regulatory subunit 4 [Source:HGNC Symbol;Acc:9308]	9	131868229	131911225	76	0.007	1	1	2.792E-05	2.104E-04	PB-CAG-SD
ZBED3-AS1	ZBED3 antisense RNA 1 [Source:HGNC Symbol;Acc:44188]	5	76377565	76462734	527	0.056	2	2	2.805E-05	2.104E-04	PB-CAG-SD
SRSF9	serine/arginine-rich splicing factor 9 [Source:HGNC Symbol;Acc:10791]	12	120899471	120912596	65	0.008	1	1	2.817E-05	2.104E-04	PB-CAG-SD
CXXC5	CXXC finger protein 5 [Source:HGNC Symbol;Acc:26943]	5	139021884	139063467	71	0.008	1	1	2.830E-05	2.104E-04	PB-CAG-SD
SPPL2B	signal peptide peptidase like 2B [Source:HGNC Symbol;Acc:30627]	19	2323617	2355099	35	0.008	1	1	2.892E-05	2.136E-04	PB-CAG-SD
FAM73B	family with sequence similarity 73, member B [Source:HGNC Symbol;Acc:23621]	9	131793900	131834361	78	0.008	1	1	2.941E-05	2.159E-04	PB-CAG-SD
CTNNAL1	catenin (cadherin-associated protein), alpha-like 1 [Source:HGNC Symbol;Acc:2512]	9	111704851	111780809	580	0.057	2	1	2.984E-05	2.176E-04	PB-CAG-SD
SEMA4G	sema domain, immunoglobulin domain (Ig), transmembrane domain (TM) and short cytoplasmic domain, (semaphorin) 4G [Sou	10	102724275	102745628	68	0.008	1	1	3.000E-05	2.176E-04	PB-CAG-SD
POLR2K	polymerase (RNA) II (DNA directed) polypeptide K, 7.0kDa [Source:HGNC Symbol;Acc:9198]	8	101157812	101166230	64	0.008	1	1	3.097E-05	2.233E-04	PB-CAG-SD
DUSP6	dual specificity phosphatase 6 [Source:HGNC Symbol;Acc:3072]	12	89741009	89752048	69	0.008	1	1	3.174E-05	2.275E-04	PB-CAG-SD
RERG-IT1	RERG intronic transcript 1 (non-protein coding) [Source:HGNC Symbol;Acc:39991]	12	15265297	15272632	70	0.008	1	1	3.266E-05	2.324E-04	PB-CAG-SD
FAM57A	family with sequence similarity 57, member A [Source:HGNC Symbol;Acc:29646]	17	630652	646206	53	0.008	1	1	3.281E-05	2.324E-04	PB-CAG-SD
ERGIC2	ERGIC and golgi 2 [Source:HGNC Symbol;Acc:30208]	12	29490285	29539122	516	0.060	2	1	3.398E-05	2.392E-04	PB-CAG-SD
COA5	cytochrome c oxidase assembly factor 5 [Source:HGNC Symbol;Acc:33848]	2	99215773	99229978	91	0.008	1	1	3.417E-05	2.392E-04	PB-CAG-SD
PRSS58	protease, serine, 58 [Source:HGNC Symbol;Acc:39125]	7	141951963	141962878	70	0.008	1	1	3.507E-05	2.440E-04	PB-CAG-SD
UBXN10	UBX domain protein 10 [Source:HGNC Symbol;Acc:26354]	1	20507578	20522541	70	0.008	1	1	3.575E-05	2.473E-04	PB-CAG-SD
SLC1A1	solute carrier family 1 (neuronal/epithelial high affinity glutamate transporter, system Xag), member 1 [Source:HGNC Symbol;Ac	9	4485444	4587469	618	0.061	2	1	3.600E-05	2.477E-04	PB-CAG-SD
RRP15	ribosomal RNA processing 15 homolog (S. cerevisiae) [Source:HGNC Symbol;Acc:24255]	1	218453629	218511325	505	0.061	2	1	3.644E-05	2.493E-04	PB-CAG-SD
C7orf55	chromosome 7 open reading frame 55 [Source:HGNC Symbol;Acc:26946]	7	139019203	139031065	72	0.009	1	1	3.709E-05	2.495E-04	PB-CAG-SD
HIST1H2BJ	histone cluster 1, H2bj [Source:HGNC Symbol;Acc:4761]	6	27093676	27105541	87	0.009	1	1	3.712E-05	2.495E-04	PB-CAG-SD
UNC50	unc-50 homolog (C. elegans) [Source:HGNC Symbol;Acc:16046]	2	99220042	99234978	95	0.009	1	1	3.724E-05	2.495E-04	PB-CAG-SD

table S2. Genes with significant transposon integrations in RKO *BRAF*$^{wt/-/-}$ cells after selection in low glucose medium

Gene ID	Description	Chrom	Start	End	# TTAA sites	Expected	Observed	Libraries	*P*	FDR	Transposon
SMAP2	small ArfGAP2 [Source:HGNC Symbol;Acc:25082]	1	40805522	40888998	509	0.062	2	2	3.730E-05	2.495E-04	PB-CAG-SD
KANSL1-AS1	KANSL1 antisense RNA 1 [Source:HGNC Symbol;Acc:43740]	17	44265942	44274089	57	0.009	1	1	3.794E-05	2.524E-04	PB-CAG-SD
CBL	Cbl proto-oncogene, E3 ubiquitin protein ligase [Source:HGNC Symbol;Acc:1541]	11	119071752	119178859	723	0.063	2	1	3.964E-05	2.622E-04	PB-CAG-SD
CYP4F10P	cytochrome P450, family 4, subfamily F, polypeptide 10, pseudogene [Source:HGNC Symbol;Acc:39943]	19	15769931	15780635	42	0.009	1	1	4.160E-05	2.737E-04	PB-CAG-SD
SRSF7	serine/arginine-rich splicing factor 7 [Source:HGNC Symbol;Acc:10789]	2	38970741	38983636	101	0.009	1	1	4.207E-05	2.739E-04	PB-CAG-SD
FEZ2	fasciculation and elongation protein zeta 2 (zygin II) [Source:HGNC Symbol;Acc:3660]	2	36778570	36878230	705	0.064	2	1	4.208E-05	2.739E-04	PB-CAG-SD
CALCOCO1	calcium binding and coiled-coil domain 1 [Source:HGNC Symbol;Acc:29306]	12	54104903	54126529	80	0.009	1	1	4.263E-05	2.759E-04	PB-CAG-SD
TKTL1	transketolase-like 1 [Source:HGNC Symbol;Acc:11835]	X	153519024	153558700	140	0.010	1	1	4.583E-05	2.951E-04	PB-CAG-SD
C11orf71	chromosome 11 open reading frame 71 [Source:HGNC Symbol;Acc:25937]	11	114262165	114276139	112	0.010	1	1	4.722E-05	3.024E-04	PB-CAG-SD
PHYKPL	5-phosphohydroxy-L-lysine phospho-lyase [Source:HGNC Symbol;Acc:28249]	5	177635498	177664792	93	0.010	1	1	4.849E-05	3.089E-04	PB-CAG-SD
HIST1H2BK	histone cluster 1, H2bk [Source:HGNC Symbol;Acc:13954]	6	27106073	27119619	100	0.010	1	1	4.900E-05	3.105E-04	PB-CAG-SD
SSU72	SSU72 RNA polymerase II CTD phosphatase homolog (S. cerevisiae) [Source:HGNC Symbol;Acc:25016]	1	1477053	1515249	84	0.010	1	1	5.142E-05	3.241E-04	PB-CAG-SD
ZNF888	zinc finger protein 888 [Source:HGNC Symbol;Acc:38695]	19	53418449	53431723	47	0.010	1	1	5.206E-05	3.265E-04	PB-CAG-SD
SAP30	Sin3A-associated protein, 30kDa [Source:HGNC Symbol;Acc:10532]	4	174286120	174298683	118	0.010	1	1	5.381E-05	3.357E-04	PB-CAG-SD
NOA1	nitric oxide associated 1 [Source:HGNC Symbol;Acc:28473]	4	57829536	57849989	121	0.011	1	1	5.657E-05	3.511E-04	PB-CAG-SD
MYL12A	myosin, light chain 12A, regulatory, non-sarcomeric [Source:HGNC Symbol;Acc:16701]	18	3242479	3256234	110	0.011	1	1	5.907E-05	3.647E-04	PB-CAG-SD
ZNHIT6	zinc finger, HIT-type containing 6 [Source:HGNC Symbol;Acc:26089]	1	86115106	86179116	596	0.072	2	2	5.941E-05	3.650E-04	PB-CAG-SD
PHLDA1	pleckstrin homology-like domain, family A, member 1 [Source:HGNC Symbol;Acc:8933]	12	76419227	76432712	95	0.011	1	1	6.004E-05	3.670E-04	PB-CAG-SD
GTPBP2	GTP binding protein 2 [Source:HGNC Symbol;Acc:4670]	6	43573053	43601899	113	0.011	1	1	6.252E-05	3.802E-04	PB-CAG-SD
M6PR	mannose-6-phosphate receptor (cation dependent) [Source:HGNC Symbol;Acc:6752]	12	9092959	9107551	98	0.011	1	1	6.388E-05	3.865E-04	PB-CAG-SD
ZKSCAN3	zinc finger with KRAB and SCAN domains 3 [Source:HGNC Symbol;Acc:13853]	6	28312691	28336947	120	0.012	1	1	7.047E-05	4.225E-04	PB-CAG-SD
SCNN1A	sodium channel, non-voltage-gated 1 alpha subunit [Source:HGNC Symbol;Acc:10599]	12	6456009	6491896	103	0.012	1	1	7.054E-05	4.225E-04	PB-CAG-SD
SATB2-AS1	SATB2 antisense RNA 1 [Source:HGNC Symbol;Acc:26490]	2	200317423	200341658	134	0.012	1	1	7.391E-05	4.371E-04	PB-CAG-SD
BRD9	bromodomain containing 9 [Source:HGNC Symbol;Acc:25818]	5	850406	897939	115	0.012	1	1	7.402E-05	4.371E-04	PB-CAG-SD
LINC00702	long intergenic non-protein coding RNA 702 [Source:HGNC Symbol;Acc:44676]	10	4277752	4290981	107	0.012	1	1	7.406E-05	4.371E-04	PB-CAG-SD
MTDH	metadherin [Source:HGNC Symbol;Acc:29608]	8	98651407	98740998	643	0.079	2	2	7.826E-05	4.596E-04	PB-CAG-SD
ABTB2	ankyrin repeat and BTB (POZ) domain containing 2 [Source:HGNC Symbol;Acc:23842]	11	34172535	34384555	917	0.080	2	1	7.986E-05	4.663E-04	PB-CAG-SD
C9orf64	chromosome 9 open reading frame 64 [Source:HGNC Symbol;Acc:28144]	9	86553226	86576901	129	0.013	1	1	8.017E-05	4.663E-04	PB-CAG-SD
ZNF282	zinc finger protein 282 [Source:HGNC Symbol;Acc:13076]	7	148887577	148923339	107	0.013	1	1	8.169E-05	4.708E-04	PB-CAG-SD
TAF13	TAF13 RNA polymerase II, TATA box binding protein (TBP)-associated factor, 18kDa [Source:HGNC Symbol;Acc:11546]	1	109605108	109623624	106	0.013	1	1	8.173E-05	4.708E-04	PB-CAG-SD
GEMIN8	gem (nuclear organelle) associated protein 8 [Source:HGNC Symbol;Acc:26044]	X	14026398	14053012	188	0.013	1	1	8.247E-05	4.728E-04	PB-CAG-SD
IAH1	isoamyl acetate-hydrolyzing esterase 1 homolog (S. cerevisiae) [Source:HGNC Symbol;Acc:27696]	2	9608787	9636672	143	0.013	1	1	8.412E-05	4.800E-04	PB-CAG-SD
CCT6P1	chaperonin containing TCP1, subunit 6 (zeta) pseudogene 1 [Source:HGNC Symbol;Acc:33094]	7	65211129	65228341	110	0.013	1	1	8.632E-05	4.902E-04	PB-CAG-SD
RPS24	ribosomal protein S24 [Source:HGNC Symbol;Acc:10411]	10	79788518	79816570	116	0.013	1	1	8.698E-05	4.917E-04	PB-CAG-SD
NPNT	nephronectin [Source:HGNC Symbol;Acc:27405]	4	106810932	106925184	943	0.083	2	2	9.017E-05	5.073E-04	PB-CAG-SD
NUBP1	nucleotide binding protein 1 [Source:HGNC Symbol;Acc:8041]	16	10832643	10863208	104	0.014	1	1	9.134E-05	5.115E-04	PB-CAG-SD
RBM7	RNA binding motif protein 7 [Source:HGNC Symbol;Acc:9904]	11	114265752	114284925	158	0.014	1	1	9.373E-05	5.224E-04	PB-CAG-SD
ABI2	abl-interactor 2 [Source:HGNC Symbol;Acc:24011]	2	204187942	204301606	934	0.085	2	1	9.634E-05	5.345E-04	PB-CAG-SD
UCA1	urothelial cancer associated 1 (non-protein coding) [Source:HGNC Symbol;Acc:37126]	19	15934757	15947130	65	0.014	1	1	9.931E-05	5.485E-04	PB-CAG-SD
CUBNP2	cubilin (intrinsic factor-cobalamin receptor) pseudogene 2 [Source:HGNC Symbol;Acc:44984]	10	45727474	45747809	125	0.014	1	1	1.009E-04	5.549E-04	PB-CAG-SD
LINC00458	long intergenic non-protein coding RNA 458 [Source:HGNC Symbol;Acc:42807]	13	54689924	54712001	166	0.014	1	1	1.017E-04	5.566E-04	PB-CAG-SD
PDE12	phosphodiesterase 12 [Source:HGNC Symbol;Acc:25386]	3	57537003	57552571	121	0.014	1	1	1.032E-04	5.597E-04	PB-CAG-SD
TRIP13	thyroid hormone receptor interactor 13 [Source:HGNC Symbol;Acc:12307]	5	887758	919472	136	0.014	1	1	1.034E-04	5.597E-04	PB-CAG-SD
OSTC	oligosaccharyltransferase complex subunit (non-catalytic) [Source:HGNC Symbol;Acc:24448]	4	109566740	109588976	164	0.014	1	1	1.037E-04	5.597E-04	PB-CAG-SD
OXSR1	oxidative stress responsive 1 [Source:HGNC Symbol;Acc:8508]	3	38201580	38296979	733	0.087	2	2	1.044E-04	5.612E-04	PB-CAG-SD
EFCC1	EF-hand and coiled-coil domain containing 1 [Source:HGNC Symbol;Acc:25692]	3	128715472	128759585	123	0.015	1	1	1.066E-04	5.706E-04	PB-CAG-SD
STT3A-AS1	STT3A antisense RNA 1 [Source:HGNC Symbol;Acc:44585]	11	125440180	125467473	169	0.015	1	1	1.072E-04	5.709E-04	PB-CAG-SD
GSG1	germ cell associated 1 [Source:HGNC Symbol;Acc:19716]	12	13236494	13261619	128	0.015	1	1	1.087E-04	5.767E-04	PB-CAG-SD
SNHG5	small nucleolar RNA host gene 5 (non-protein coding) [Source:HGNC Symbol;Acc:21026]	6	86370710	86393451	150	0.015	1	1	1.099E-04	5.803E-04	PB-CAG-SD
CCDC117	coiled-coil domain containing 117 [Source:HGNC Symbol;Acc:26599]	22	29163662	29185283	125	0.015	1	1	1.107E-04	5.820E-04	PB-CAG-SD
KPNA7	karyopherin alpha 7 (importin alpha 8) [Source:HGNC Symbol;Acc:21839]	7	98771197	98810129	125	0.015	1	1	1.113E-04	5.828E-04	PB-CAG-SD
HMGB1	high mobility group box 1 [Source:HGNC Symbol;Acc:4983]	13	31032884	31196734	1046	0.090	2	1	1.147E-04	5.977E-04	PB-CAG-SD
LINC00443	long intergenic non-protein coding RNA 443 [Source:HGNC Symbol;Acc:42780]	13	107301264	107324511	177	0.015	1	1	1.155E-04	5.978E-04	PB-CAG-SD
LTN1	listerin E3 ubiquitin protein ligase 1 [Source:HGNC Symbol;Acc:13082]	21	30300466	30370277	624	0.091	2	1	1.157E-04	5.978E-04	PB-CAG-SD
OXSM	3-oxoacyl-ACP synthase, mitochondrial [Source:HGNC Symbol;Acc:26063]	3	25819408	25836025	129	0.015	1	1	1.172E-04	6.032E-04	PB-CAG-SD
AKIRIN1	akirin 1 [Source:HGNC Symbol;Acc:25744]	1	39451895	39471731	128	0.016	1	1	1.190E-04	6.095E-04	PB-CAG-SD
SULT1C2P1	sulfotransferase family, cytosolic, 1C, member 2 pseudogene 1 [Source:HGNC Symbol;Acc:33545]	2	108933694	108970255	171	0.016	1	1	1.201E-04	6.126E-04	PB-CAG-SD
DDX47	DEAD (Asp-Glu-Ala-Asp) box polypeptide 47 [Source:HGNC Symbol;Acc:18682]	12	12961250	12982915	135	0.016	1	1	1.209E-04	6.132E-04	PB-CAG-SD
MCEE	methylmalonyl CoA epimerase [Source:HGNC Symbol;Acc:16732]	2	71336814	71362369	172	0.016	1	1	1.215E-04	6.132E-04	PB-CAG-SD
ZNF181	zinc finger protein 181 [Source:HGNC Symbol;Acc:12971]	19	35220060	35233777	72	0.016	1	1	1.217E-04	6.132E-04	PB-CAG-SD
STOML3	stomatin (EPB72)-like 3 [Source:HGNC Symbol;Acc:19420]	13	39540062	39570203	184	0.016	1	1	1.248E-04	6.262E-04	PB-CAG-SD
COX20	COX20 cytochrome C oxidase assembly factor [Source:HGNC Symbol;Acc:26970]	1	244993624	245008359	132	0.016	1	1	1.265E-04	6.283E-04	PB-CAG-SD
MPHOSPH10	M-phase phosphoprotein 10 (U3 small nucleolar ribonucleoprotein) [Source:HGNC Symbol;Acc:7213]	2	71352444	71377231	176	0.016	1	1	1.272E-04	6.283E-04	PB-CAG-SD
EXOC3	exocyst complex component 3 [Source:HGNC Symbol;Acc:30378]	5	438273	472052	151	0.016	1	1	1.273E-04	6.283E-04	PB-CAG-SD
CKLF	chemokine-like factor [Source:HGNC Symbol;Acc:13253]	16	66581466	66600331	123	0.016	1	1	1.275E-04	6.283E-04	PB-CAG-SD
SIRPB2	signal-regulatory protein beta 2 [Source:HGNC Symbol;Acc:16247]	20	1451386	1477233	112	0.016	1	1	1.281E-04	6.283E-04	PB-CAG-SD
ZBED3	zinc finger, BED-type containing 3 [Source:HGNC Symbol;Acc:20711]	5	76367897	76388148	152	0.016	1	1	1.290E-04	6.283E-04	PB-CAG-SD
PHKA1-AS1	PHKA1 antisense RNA 1 [Source:HGNC Symbol;Acc:40446]	X	71903800	71932190	236	0.016	1	1	1.297E-04	6.283E-04	PB-CAG-SD
NAA38	N(alpha)-acetyltransferase 38, NatC auxiliary subunit [Source:HGNC Symbol;Acc:20471]	7	117819086	117832878	135	0.016	1	1	1.297E-04	6.283E-04	PB-CAG-SD
DYNLL1	dynein, light chain, LC8-type 1 [Source:HGNC Symbol;Acc:15476]	12	120902653	120936296	140	0.016	1	1	1.299E-04	6.283E-04	PB-CAG-SD
MVB12B	multivesicular body subunit 12B [Source:HGNC Symbol;Acc:23368]	9	129084128	129269320	965	0.095	2	1	1.336E-04	6.434E-04	PB-CAG-SD
SHPRH	SNF2 histone linker PHD RING helicase, E3 ubiquitin protein ligase [Source:HGNC Symbol;Acc:19336]	6	146185381	146290559	959	0.095	2	1	1.341E-04	6.434E-04	PB-CAG-SD
THAP8	THAP domain containing 8 [Source:HGNC Symbol;Acc:23191]	19	36525887	36550664	76	0.017	1	1	1.356E-04	6.476E-04	PB-CAG-SD
ZRSR2	zinc finger (CCCH type), RNA-binding motif and serine/arginine rich 2 [Source:HGNC Symbol;Acc:23019]	X	15803595	15841383	243	0.017	1	1	1.374E-04	6.516E-04	PB-CAG-SD
HNRNPF	heterogeneous nuclear ribonucleoprotein F [Source:HGNC Symbol;Acc:5039]	10	43881065	43909614	146	0.017	1	1	1.375E-04	6.516E-04	PB-CAG-SD
ARHGEF28	Rho guanine nucleotide exchange factor (GEF) 28 [Source:HGNC Symbol;Acc:30322]	5	72916983	73237818	2380	0.253	3	1	1.392E-04	6.574E-04	PB-CAG-SD
CPNE2	copine II [Source:HGNC Symbol;Acc:2315]	16	57121449	57181878	129	0.017	1	1	1.402E-04	6.595E-04	PB-CAG-SD
ERICH2	glutamate-rich 2 [Source:HGNC Symbol;Acc:44395]	2	171635296	171655481	188	0.017	1	1	1.450E-04	6.793E-04	PB-CAG-SD
FAM83F	family with sequence similarity 83, member F [Source:HGNC Symbol;Acc:25148]	22	40385953	40426043	144	0.017	1	1	1.467E-04	6.831E-04	PB-CAG-SD
C20orf96	chromosome 20 open reading frame 96 [Source:HGNC Symbol;Acc:16227]	20	251504	276390	120	0.017	1	1	1.470E-04	6.831E-04	PB-CAG-SD
KCTD15	potassium channel tetramerization domain containing 15 [Source:HGNC Symbol;Acc:23297]	19	34281838	34306666	80	0.017	1	1	1.501E-04	6.925E-04	PB-CAG-SD
VN2R3P	vomeronasal 2 receptor 3 pseudogene [Source:HGNC Symbol;Acc:33209]	9	39011966	39039591	177	0.017	1	1	1.504E-04	6.925E-04	PB-CAG-SD
CASP3	caspase 3, apoptosis-related cysteine peptidase [Source:HGNC Symbol;Acc:1504]	4	185548850	185575663	198	0.017	1	1	1.508E-04	6.925E-04	PB-CAG-SD
EXT1	exostosin glycosyltransferase 1 [Source:HGNC Symbol;Acc:3512]	8	118806729	119129092	2096	0.258	3	2	1.513E-04	6.925E-04	PB-CAG-SD
MTFR2	mitochondrial fission regulator 2 [Source:HGNC Symbol;Acc:21115]	6	136552162	136576473	179	0.018	1	1	1.562E-04	7.123E-04	PB-CAG-SD
NTSR1	neurotensin receptor 1 (high affinity) [Source:HGNC Symbol;Acc:8039]	20	61335189	61394123	124	0.018	1	1	1.569E-04	7.126E-04	PB-CAG-SD
MAK16	MAK16 homolog (S. cerevisiae) [Source:HGNC Symbol;Acc:13703]	8	33337268	33358778	146	0.018	1	1	1.601E-04	7.246E-04	PB-CAG-SD
HOMEZ	homeobox and leucine zipper encoding [Source:HGNC Symbol;Acc:20164]	14	23741666	23773656	167	0.018	1	1	1.652E-04	7.424E-04	PB-CAG-SD
MRPL37	mitochondrial ribosomal protein L37 [Source:HGNC Symbol;Acc:14034]	1	54644714	54691137	151	0.018	1	1	1.653E-04	7.424E-04	PB-CAG-SD
FAM200B	family with sequence similarity 200, member B [Source:HGNC Symbol;Acc:27740]	4	15678285	15707188	208	0.018	1	1	1.663E-04	7.435E-04	PB-CAG-SD
U2SURP	U2 snRNP-associated SURP domain containing [Source:HGNC Symbol;Acc:30855]	3	142678339	142779567	860	0.103	2	2	1.667E-04	7.435E-04	PB-CAG-SD
FAM72B	family with sequence similarity 72, member B [Source:HGNC Symbol;Acc:24805]	1	120832756	120855681	156	0.019	1	1	1.763E-04	7.831E-04	PB-CAG-SD
ST3GAL2	ST3 beta-galactoside alpha-2,3-sialyltransferase 2 [Source:HGNC Symbol;Acc:10863]	16	70413338	70478140	145	0.019	1	1	1.769E-04	7.831E-04	PB-CAG-SD
RBFOX2	RNA binding protein, fox-1 homolog (C. elegans) 2 [Source:HGNC Symbol;Acc:9906]	22	36134783	36429473	2254	0.270	3	1	1.777E-04	7.836E-04	PB-CAG-SD
GEMIN6	gem (nuclear organelle) associated protein 6 [Source:HGNC Symbol;Acc:20044]	2	38973676	39009598	209	0.019	1	1	1.790E-04	7.864E-04	PB-CAG-SD
DCAKD	dephospho-CoA kinase domain containing [Source:HGNC Symbol;Acc:26238]	17	43100708	43143473	125	0.019	1	1	1.812E-04	7.904E-04	PB-CAG-SD
LASP1	LIM and SH3 protein 1 [Source:HGNC Symbol;Acc:6513]	17	37021112	37078023	125	0.019	1	1	1.812E-04	7.904E-04	PB-CAG-SD
ENSA	endosulfine alpha [Source:HGNC Symbol;Acc:3360]	1	150573327	150607088	159	0.019	1	1	1.831E-04	7.959E-04	PB-CAG-SD
COQ5	coenzyme Q5 homolog, methyltransferase (S. cerevisiae) [Source:HGNC Symbol;Acc:28722]	12	120941077	120977237	167	0.019	1	1	1.845E-04	7.991E-04	PB-CAG-SD
ZNF799	zinc finger protein 799 [Source:HGNC Symbol;Acc:28071]	19	12500830	12517085	89	0.019	1	1	1.855E-04	8.007E-04	PB-CAG-SD
SDHD	succinate dehydrogenase complex, subunit D, integral membrane protein [Source:HGNC Symbol;Acc:10683]	11	111952497	111990353	225	0.020	1	1	1.893E-04	8.108E-04	PB-CAG-SD
LAIR1	leukocyte-associated immunoglobulin-like receptor 1 [Source:HGNC Symbol;Acc:6477]	19	54865362	54887165	90	0.020	1	1	1.897E-04	8.108E-04	PB-CAG-SD
XPA	xeroderma pigmentosum, complementation group A [Source:HGNC Symbol;Acc:12814]	9	100437191	100464639	199	0.020	1	1	1.899E-04	8.108E-04	PB-CAG-SD
PLCG1	phospholipase C, gamma 1 [Source:HGNC Symbol;Acc:9065]	20	39760600	39811629	137	0.020	1	1	1.912E-04	8.136E-04	PB-CAG-SD
MTRF1L	mitochondrial translational release factor 1-like [Source:HGNC Symbol;Acc:21051]	6	153308497	153328820	199	0.020	1	1	1.928E-04	8.173E-04	PB-CAG-SD
NLN	neurolysin (metallopeptidase M3 family) [Source:HGNC Symbol;Acc:16058]	5	65013023	65167553	1018	0.108	2	1	1.944E-04	8.214E-04	PB-CAG-SD
EIF3J	eukaryotic translation initiation factor 3, subunit J [Source:HGNC Symbol;Acc:3270]	15	44824255	44855227	193	0.020	1	1	1.963E-04	8.258E-04	PB-CAG-SD
FBXO43	F-box protein 43 [Source:HGNC Symbol;Acc:28521]	8	101145588	101163028	162	0.020	1	1	1.968E-04	8.258E-04	PB-CAG-SD
SNX19	sorting nexin 19 [Source:HGNC Symbol;Acc:21532]	11	130745331	130791404	230	0.020	1	1	1.978E-04	8.268E-04	PB-CAG-SD
ARPC5	actin related protein 2/3 complex, subunit 5, 16kDa [Source:HGNC Symbol;Acc:708]	1	183592401	183609892	166	0.020	1	1	1.995E-04	8.310E-04	PB-CAG-SD
BAG2	BCL2-associated athanogene 2 [Source:HGNC Symbol;Acc:938]	6	57032124	57054631	204	0.020	1	1	2.025E-04	8.409E-04	PB-CAG-SD
PSAT1	phosphoserine aminotransferase 1 [Source:HGNC Symbol;Acc:19129]	9	80907059	80945009	206	0.020	1	1	2.034E-04	8.416E-04	PB-CAG-SD
KCNK12	potassium channel, subfamily K, member 12 [Source:HGNC Symbol;Acc:6274]	2	47747910	47803078	224	0.020	1	1	2.054E-04	8.469E-04	PB-CAG-SD
PACSIN1	protein kinase C and casein kinase substrate in neurons 1 [Source:HGNC Symbol;Acc:8570]	6	34428916	34503006	208	0.021	1	1	2.105E-04	8.650E-04	PB-CAG-SD
CYP4F3	cytochrome P450, family 4, subfamily F, polypeptide 3 [Source:HGNC Symbol;Acc:2646]	19	15746707	15773634	95	0.021	1	1	2.112E-04	8.650E-04	PB-CAG-SD
ALLC	allantoicase [Source:HGNC Symbol;Acc:17377]	2	3700785	3750261	228	0.021	1	1	2.128E-04	8.666E-04	PB-CAG-SD
MEIG1	meiosis expressed gene 1 homolog (mouse) [Source:HGNC Symbol;Acc:23429]	10	14996438	15030049	182	0.021	1	1	2.130E-04	8.666E-04	PB-CAG-SD
NEK3	NIMA-related kinase 3 [Source:HGNC Symbol;Acc:7746]	13	52706775	52738996	242	0.021	1	1	2.152E-04	8.713E-04	PB-CAG-SD
ZNF230	zinc finger protein 230 [Source:HGNC Symbol;Acc:13024]	19	44502100	44518078	96	0.021	1	1	2.157E-04	8.713E-04	PB-CAG-SD
WDR82	WD repeat domain 82 [Source:HGNC Symbol;Acc:28826]	3	52288437	52327036	176	0.021	1	1	2.174E-04	8.755E-04	PB-CAG-SD
CD8B	CD8b molecule [Source:HGNC Symbol;Acc:1707]	2	87042462	87094047	231	0.021	1	1	2.183E-04	8.763E-04	PB-CAG-SD
TBC1D20	TBC1 domain family, member 20 [Source:HGNC Symbol;Acc:16133]	20	416124	448197	147	0.021	1	1	2.200E-04	8.795E-04	PB-CAG-SD
STT3A	STT3A, subunit of the oligosaccharyltransferase complex (catalytic) [Source:HGNC Symbol;Acc:6172]	11	125456607	125495110	243	0.021	1	1	2.206E-04	8.795E-04	PB-CAG-SD
LDHB	lactate dehydrogenase B [Source:HGNC Symbol;Acc:6541]	12	21788276	21915791	979	0.113	2	2	2.230E-04	8.860E-04	PB-CAG-SD
RPL30	ribosomal protein L30 [Source:HGNC Symbol;Acc:10333]	8	99037079	99063697	175	0.022	1	1	2.295E-04	9.088E-04	PB-CAG-SD
SF3A3	splicing factor 3a, subunit 3, 60kDa [Source:HGNC Symbol;Acc:10767]	1	38422647	38461593	179	0.022	1	1	2.317E-04	9.146E-04	PB-CAG-SD
PNPLA7	patatin-like phospholipase domain containing 7 [Source:HGNC Symbol;Acc:24768]	9	140354404	140449986	221	0.022	1	1	2.339E-04	9.164E-04	PB-CAG-SD
ZNF235	zinc finger protein 235 [Source:HGNC Symbol;Acc:12866]	19	44732882	44814199	529	0.115	2	1	2.340E-04	9.164E-04	PB-CAG-SD
CRTAP	cartilage associated protein [Source:HGNC Symbol;Acc:2379]	3	33150471	33189265	183	0.022	1	1	2.349E-04	9.164E-04	PB-CAG-SD
WDR63	WD repeat domain 63 [Source:HGNC Symbol;Acc:30711]	1	85459830	85598821	953	0.115	2	1	2.352E-04	9.164E-04	PB-CAG-SD
IMPA2	inositol(myo)-1(or 4)-monophosphatase 2 [Source:HGNC Symbol;Acc:6051]	18	11976024	12030876	222	0.022	1	1	2.388E-04	9.275E-04	PB-CAG-SD
OSTN-AS1	OSTN antisense RNA 1 [Source:HGNC Symbol;Acc:41250]	3	190931080	190957394	185	0.022	1	1	2.400E-04	9.293E-04	PB-CAG-SD
ZSCAN31	zinc finger and SCAN domain containing 31 [Source:HGNC Symbol;Acc:14097]	6	28292470	28329048	224	0.022	1	1	2.439E-04	9.411E-04	PB-CAG-SD
FAM221A	family with sequence similarity 221, member A [Source:HGNC Symbol;Acc:27977]	7	23714749	23742868	186	0.022	1	1	2.453E-04	9.436E-04	PB-CAG-SD
DHRS4L2	dehydrogenase/reductase (SDR family) member 4 like 2 [Source:HGNC Symbol;Acc:19731]	14	24434148	24475617	205	0.022	1	1	2.482E-04	9.492E-04	PB-CAG-SD
NAA60	N(alpha)-acetyltransferase 60, NatF catalytic subunit [Source:HGNC Symbol;Acc:25875]	16	3488611	3536963	172	0.022	1	1	2.483E-04	9.492E-04	PB-CAG-SD
STARD4	StAR-related lipid transfer (START) domain containing 4 [Source:HGNC Symbol;Acc:18058]	5	110831731	110853288	212	0.023	1	1	2.498E-04	9.519E-04	PB-CAG-SD
SMG9	SMG9 nonsense mediated mRNA decay factor [Source:HGNC Symbol;Acc:25763]	19	44235301	44264142	104	0.023	1	1	2.528E-04	9.576E-04	PB-CAG-SD

table S2. Genes with significant transposon integrations in RKO *BRAF*$^{wt/-/-}$ cells after selection in low glucose medium

Gene ID	Description	Chrom	Start	End	# TTAA sites	Expected	Observed	Libraries	*P*	FDR	Transposon
VGLL4	vestigial like 4 (Drosophila) [Source:HGNC Symbol;Acc:28966]	3	11597544	11767220	992	0.118	2	1	2.529E-04	9.576E-04	PB-CAG-SD
TRIM14	tripartite motif containing 14 [Source:HGNC Symbol;Acc:16283]	9	100831557	100886494	231	0.023	1	1	2.553E-04	9.637E-04	PB-CAG-SD
LSM12	LSM12 homolog (S. cerevisiae) [Source:HGNC Symbol;Acc:26407]	17	42112003	42149987	150	0.023	1	1	2.602E-04	9.792E-04	PB-CAG-SD
PLEKHA3	pleckstrin homology domain containing, family A (phosphoinositide binding specific) member 3 [Source:HGNC Symbol;Acc:143	2	179340195	179370093	257	0.023	1	1	2.698E-04	1.012E-03	PB-CAG-SD
PITPNA	phosphatidylinositol transfer protein, alpha [Source:HGNC Symbol;Acc:9001]	17	1421012	1471110	153	0.023	1	1	2.707E-04	1.012E-03	PB-CAG-SD
STMND1	stathmin domain containing 1 [Source:HGNC Symbol;Acc:44668]	6	17097489	17131603	237	0.024	1	1	2.728E-04	1.017E-03	PB-CAG-SD
BMP3	bone morphogenetic protein 3 [Source:HGNC Symbol;Acc:1070]	4	81947119	81978685	270	0.024	1	1	2.792E-04	1.037E-03	PB-CAG-SD
MTFR1	mitochondrial fission regulator 1 [Source:HGNC Symbol;Acc:29510]	8	66551969	66683496	994	0.123	2	1	2.799E-04	1.037E-03	PB-CAG-SD
BCCIP	BRCA2 and CDKN1A interacting protein [Source:HGNC Symbol;Acc:978]	10	127507115	127542264	210	0.024	1	1	2.830E-04	1.043E-03	PB-CAG-SD
NSFL1C	NSFL1 (p97) cofactor (p47) [Source:HGNC Symbol;Acc:15912]	20	1422807	1453417	167	0.024	1	1	2.833E-04	1.043E-03	PB-CAG-SD
MIER2	mesoderm induction early response 1, family member 2 [Source:HGNC Symbol;Acc:29210]	19	305575	349798	111	0.024	1	1	2.877E-04	1.056E-03	PB-CAG-SD
SUPT5H	suppressor of Ty 5 homolog (S. cerevisiae) [Source:HGNC Symbol;Acc:11469]	19	39921796	39967310	112	0.024	1	1	2.929E-04	1.072E-03	PB-CAG-SD
ATP1A4	ATPase, Na+/K+ transporting, alpha 4 polypeptide [Source:HGNC Symbol;Acc:14073]	1	160116360	160156767	202	0.024	1	1	2.945E-04	1.075E-03	PB-CAG-SD
MCM3AP-AS1	MCM3AP antisense RNA 1 [Source:HGNC Symbol;Acc:16417]	21	47644131	47679304	169	0.025	1	1	2.959E-04	1.075E-03	PB-CAG-SD
ADCY7	adenylate cyclase 7 [Source:HGNC Symbol;Acc:238]	16	50275048	50352046	188	0.025	1	1	2.963E-04	1.075E-03	PB-CAG-SD
AASDHPPT	aminoadipate-semialdehyde dehydrogenase-phosphopantetheinyl transferase [Source:HGNC Symbol;Acc:14235]	11	105941228	105969437	285	0.025	1	1	3.027E-04	1.089E-03	PB-CAG-SD
AAK1	AP2 associated kinase 1 [Source:HGNC Symbol;Acc:19679]	2	69688532	69906481	1382	0.126	2	1	3.027E-04	1.089E-03	PB-CAG-SD
LRRC48	leucine rich repeat containing 48 [Source:HGNC Symbol;Acc:25384]	17	17871127	17920203	162	0.025	1	1	3.032E-04	1.089E-03	PB-CAG-SD
ACIN1	apoptotic chromatin condensation inducer 1 [Source:HGNC Symbol;Acc:17066]	14	23527773	23569823	227	0.025	1	1	3.038E-04	1.089E-03	PB-CAG-SD
UROS	uroporphyrinogen III synthase [Source:HGNC Symbol;Acc:12592]	10	127477146	127516817	218	0.025	1	1	3.048E-04	1.089E-03	PB-CAG-SD
TYW5	tRNA-yW synthesizing protein 5 [Source:HGNC Symbol;Acc:26754]	2	200794698	200825459	275	0.025	1	1	3.086E-04	1.099E-03	PB-CAG-SD
ZNF595	zinc finger protein 595 [Source:HGNC Symbol;Acc:27196]	4	48179	88099	285	0.025	1	1	3.108E-04	1.101E-03	PB-CAG-SD
AAR2	AAR2 splicing factor homolog (S. cerevisiae) [Source:HGNC Symbol;Acc:15886]	20	34819381	34858840	175	0.025	1	1	3.109E-04	1.101E-03	PB-CAG-SD
SLC35B4	solute carrier family 35 (UDP-xylose/UDP-N-acetylglucosamine transporter), member B4 [Source:HGNC Symbol;Acc:20584]	7	133974084	134006803	210	0.025	1	1	3.121E-04	1.102E-03	PB-CAG-SD
MB21D1	Mab-21 domain containing 1 [Source:HGNC Symbol;Acc:21367]	6	74123238	74166999	254	0.025	1	1	3.129E-04	1.102E-03	PB-CAG-SD
EIF5A2	eukaryotic translation initiation factor 5A2 [Source:HGNC Symbol;Acc:3301]	3	170606204	170631482	212	0.025	1	1	3.145E-04	1.104E-03	PB-CAG-SD
TK2	thymidine kinase 2, mitochondrial [Source:HGNC Symbol;Acc:11831]	16	66541906	66591447	195	0.025	1	1	3.186E-04	1.115E-03	PB-CAG-SD
KBTBD3	kelch repeat and BTB (POZ) domain containing 3 [Source:HGNC Symbol;Acc:22934]	11	105921825	105953492	294	0.026	1	1	3.220E-04	1.124E-03	PB-CAG-SD
NT5M	5',3'-nucleotidase, mitochondrial [Source:HGNC Symbol;Acc:15769]	17	17201649	17250977	168	0.026	1	1	3.258E-04	1.132E-03	PB-CAG-SD
AFP	alpha-fetoprotein [Source:HGNC Symbol;Acc:317]	4	74291855	74321891	292	0.026	1	1	3.262E-04	1.132E-03	PB-CAG-SD
CACNA1I	calcium channel, voltage-dependent, T type, alpha 1I subunit [Source:HGNC Symbol;Acc:1396]	22	39961758	40085742	218	0.026	1	1	3.342E-04	1.156E-03	PB-CAG-SD
SYNJ1	synaptojanin 1 [Source:HGNC Symbol;Acc:11503]	21	34001069	34105359	902	0.131	2	2	3.391E-04	1.170E-03	PB-CAG-SD
LRP5	low density lipoprotein receptor-related protein 5 [Source:HGNC Symbol;Acc:6697]	11	68075077	68216743	303	0.026	1	1	3.418E-04	1.176E-03	PB-CAG-SD
CKLF-CMTM1	CKLF-CMTM1 readthrough [Source:HGNC Symbol;Acc:39977]	16	66581490	66613038	203	0.027	1	1	3.450E-04	1.183E-03	PB-CAG-SD
PDCD2L	programmed cell death 2-like [Source:HGNC Symbol;Acc:28194]	19	34890289	34917073	122	0.027	1	1	3.470E-04	1.187E-03	PB-CAG-SD
ANP32B	acidic (leucine-rich) nuclear phosphoprotein 32 family, member B [Source:HGNC Symbol;Acc:16677]	9	100740643	100778225	271	0.027	1	1	3.505E-04	1.192E-03	PB-CAG-SD
PSMA3	proteasome (prosome, macropain) subunit, alpha type, 3 [Source:HGNC Symbol;Acc:9532]	14	58706549	58738730	244	0.027	1	1	3.506E-04	1.192E-03	PB-CAG-SD
NBPF15	neuroblastoma breakpoint family, member 15 [Source:HGNC Symbol;Acc:28791]	1	148550979	148596267	221	0.027	1	1	3.520E-04	1.194E-03	PB-CAG-SD
RP2	retinitis pigmentosa 2 (X-linked recessive) [Source:HGNC Symbol;Acc:10274]	X	46691375	46741793	395	0.027	1	1	3.606E-04	1.220E-03	PB-CAG-SD
CHURC1	churchill domain containing 1 [Source:HGNC Symbol;Acc:20099]	14	65376079	65411309	248	0.027	1	1	3.621E-04	1.221E-03	PB-CAG-SD
ARHGAP11A	Rho GTPase activating protein 11A [Source:HGNC Symbol;Acc:15783]	15	32902345	32932150	264	0.027	1	1	3.654E-04	1.229E-03	PB-CAG-SD
SRP68	signal recognition particle 68kDa [Source:HGNC Symbol;Acc:11302]	17	74035184	74073734	180	0.028	1	1	3.736E-04	1.253E-03	PB-CAG-SD
BCL7C	B-cell CLL/lymphoma 7C [Source:HGNC Symbol;Acc:1006]	16	30844947	30911281	213	0.028	1	1	3.795E-04	1.269E-03	PB-CAG-SD
CANX	calnexin [Source:HGNC Symbol;Acc:1473]	5	179100629	179157926	263	0.028	1	1	3.831E-04	1.277E-03	PB-CAG-SD
HPSE	heparanase [Source:HGNC Symbol;Acc:5164]	4	84213614	84261306	317	0.028	1	1	3.838E-04	1.277E-03	PB-CAG-SD
ZNF141	zinc finger protein 141 [Source:HGNC Symbol;Acc:12926]	4	326603	378653	321	0.028	1	1	3.935E-04	1.305E-03	PB-CAG-SD
MCOLN3	mucolipin 3 [Source:HGNC Symbol;Acc:13358]	1	85483765	85519182	235	0.028	1	1	3.975E-04	1.311E-03	PB-CAG-SD
TXNDC12	thioredoxin domain containing 12 (endoplasmic reticulum) [Source:HGNC Symbol;Acc:24626]	1	52485803	52526843	235	0.028	1	1	3.975E-04	1.311E-03	PB-CAG-SD
CEP55	centrosomal protein 55kDa [Source:HGNC Symbol;Acc:1161]	10	95251389	95288849	250	0.029	1	1	3.999E-04	1.316E-03	PB-CAG-SD
SLC35G2	solute carrier family 35, member G2 [Source:HGNC Symbol;Acc:28480]	3	136532489	136574734	240	0.029	1	1	4.022E-04	1.320E-03	PB-CAG-SD
TFB2M	transcription factor B2, mitochondrial [Source:HGNC Symbol;Acc:18559]	1	246703862	246734626	239	0.029	1	1	4.111E-04	1.336E-03	PB-CAG-SD
PLEKHM1P	pleckstrin homology domain containing, family M (with RUN domain) member 1 pseudogene [Source:HGNC Symbol;Acc:35411	17	62775377	62838272	189	0.029	1	1	4.115E-04	1.336E-03	PB-CAG-SD
PLEKHM1	pleckstrin homology domain containing, family M (with RUN domain) member 1 [Source:HGNC Symbol;Acc:29017]	17	43513266	43573115	189	0.029	1	1	4.115E-04	1.336E-03	PB-CAG-SD
MTERFD1	MTERF domain containing 1 [Source:HGNC Symbol;Acc:24258]	8	97251626	97278838	235	0.029	1	1	4.117E-04	1.336E-03	PB-CAG-SD
ZNF876P	zinc finger protein 876, pseudogene [Source:HGNC Symbol;Acc:32472]	4	201399	249774	333	0.029	1	1	4.232E-04	1.370E-03	PB-CAG-SD
TMEM206	transmembrane protein 206 [Source:HGNC Symbol;Acc:25593]	1	212537273	212593243	244	0.030	1	1	4.283E-04	1.382E-03	PB-CAG-SD
PTGR2	prostaglandin reductase 2 [Source:HGNC Symbol;Acc:20149]	14	74313547	74353530	271	0.030	1	1	4.316E-04	1.390E-03	PB-CAG-SD
PPL	periplakin [Source:HGNC Symbol;Acc:9273]	16	4932508	5015742	229	0.030	1	1	4.381E-04	1.406E-03	PB-CAG-SD
GSR	glutathione reductase [Source:HGNC Symbol;Acc:4623]	8	30535583	30590443	243	0.030	1	1	4.400E-04	1.409E-03	PB-CAG-SD
DHRS4-AS1	DHRS4 antisense RNA 1 [Source:HGNC Symbol;Acc:23175]	14	24407940	24463048	274	0.030	1	1	4.411E-04	1.409E-03	PB-CAG-SD
CARS2	cysteinyl-tRNA synthetase 2, mitochondrial (putative) [Source:HGNC Symbol;Acc:25695]	13	111293759	111370950	348	0.030	1	1	4.423E-04	1.409E-03	PB-CAG-SD
FKBP9	FK506 binding protein 9, 63 kDa [Source:HGNC Symbol;Acc:3725]	7	32992017	33046543	251	0.030	1	1	4.444E-04	1.412E-03	PB-CAG-SD
TIPIN	TIMELESS interacting protein [Source:HGNC Symbol;Acc:30750]	15	66628544	66684084	294	0.030	1	1	4.523E-04	1.433E-03	PB-CAG-SD
MYO16-AS1	MYO16 antisense RNA 1 [Source:HGNC Symbol;Acc:39913]	13	109816250	109858831	353	0.030	1	1	4.550E-04	1.436E-03	PB-CAG-SD
UBLCP1	ubiquitin-like domain containing CTD phosphatase 1 [Source:HGNC Symbol;Acc:28110]	5	158685089	158713044	287	0.030	1	1	4.555E-04	1.436E-03	PB-CAG-SD
CTH	cystathionase (cystathionine gamma-lyase) [Source:HGNC Symbol;Acc:2501]	1	70871901	70905534	253	0.031	1	1	4.601E-04	1.443E-03	PB-CAG-SD
NMT1	N-myristoyltransferase 1 [Source:HGNC Symbol;Acc:7857]	17	43123978	43186384	200	0.031	1	1	4.603E-04	1.443E-03	PB-CAG-SD
CCDC34	coiled-coil domain containing 34 [Source:HGNC Symbol;Acc:25079]	11	27352374	27390415	356	0.031	1	1	4.704E-04	1.466E-03	PB-CAG-SD
LGALS8	lectin, galactoside-binding, soluble, 8 [Source:HGNC Symbol;Acc:6569]	1	236676300	236716281	256	0.031	1	1	4.710E-04	1.466E-03	PB-CAG-SD
CCDC125	coiled-coil domain containing 125 [Source:HGNC Symbol;Acc:28924]	5	68576002	68633636	292	0.031	1	1	4.713E-04	1.466E-03	PB-CAG-SD
CCDC111	coiled-coil domain containing 111 [Source:HGNC Symbol;Acc:26575]	4	185565767	185616117	353	0.031	1	1	4.750E-04	1.474E-03	PB-CAG-SD
CLIP2	CAP-GLY domain containing linker protein 2 [Source:HGNC Symbol;Acc:2586]	7	73698805	73820273	261	0.031	1	1	4.801E-04	1.479E-03	PB-CAG-SD
TTI2	TELO2 interacting protein 2 [Source:HGNC Symbol;Acc:26262]	8	33330904	33376119	254	0.031	1	1	4.803E-04	1.479E-03	PB-CAG-SD
JAG1	jagged 1 [Source:HGNC Symbol;Acc:6188]	20	10618332	10659694	218	0.031	1	1	4.805E-04	1.479E-03	PB-CAG-SD
MBIP	MAP3K12 binding inhibitory protein 1 [Source:HGNC Symbol;Acc:20427]	14	36767770	36794882	290	0.032	1	1	4.936E-04	1.514E-03	PB-CAG-SD
FOXJ3	forkhead box J3 [Source:HGNC Symbol;Acc:29178]	1	42642210	42806548	1231	0.149	2	1	4.943E-04	1.514E-03	PB-CAG-SD
ATPAF2	ATP synthase mitochondrial F1 complex assembly factor 2 [Source:HGNC Symbol;Acc:18802]	17	17880723	17947523	209	0.032	1	1	5.022E-04	1.535E-03	PB-CAG-SD
TEAD1	TEA domain family member 1 (SV40 transcriptional enhancer factor) [Source:HGNC Symbol;Acc:11714]	11	12690969	12966298	1728	0.150	2	1	5.070E-04	1.543E-03	PB-CAG-SD
TYW3	tRNA-yW synthesizing protein 3 homolog (S. cerevisiae) [Source:HGNC Symbol;Acc:24757]	1	75193836	75232362	266	0.032	1	1	5.081E-04	1.543E-03	PB-CAG-SD
ZNF180	zinc finger protein 180 [Source:HGNC Symbol;Acc:12970]	19	44979854	45009576	148	0.032	1	1	5.087E-04	1.543E-03	PB-CAG-SD
FMNL3	formin-like 3 [Source:HGNC Symbol;Acc:23698]	12	50031724	50106948	279	0.032	1	1	5.106E-04	1.545E-03	PB-CAG-SD
ILF3	interleukin enhancer binding factor 3, 90kDa [Source:HGNC Symbol;Acc:6038]	19	10759937	10803093	150	0.033	1	1	5.224E-04	1.576E-03	PB-CAG-SD
IFT140	intraflagellar transport 140 homolog (Chlamydomonas) [Source:HGNC Symbol;Acc:29077]	16	1560428	1667111	251	0.033	1	1	5.253E-04	1.580E-03	PB-CAG-SD
FBXO47	F-box protein 47 [Source:HGNC Symbol;Acc:31969]	17	37092685	37128655	214	0.033	1	1	5.262E-04	1.580E-03	PB-CAG-SD
WWC2	WW and C2 domain containing 2 [Source:HGNC Symbol;Acc:24148]	4	184015446	184241930	1734	0.153	2	1	5.322E-04	1.594E-03	PB-CAG-SD
FMO1	flavin containing monooxygenase 1 [Source:HGNC Symbol;Acc:3769]	1	171212638	171255117	273	0.033	1	1	5.349E-04	1.598E-03	PB-CAG-SD
MCM10	minichromosome maintenance complex component 10 [Source:HGNC Symbol;Acc:18043]	10	13198554	13253104	291	0.033	1	1	5.401E-04	1.606E-03	PB-CAG-SD
ANXA7	annexin A7 [Source:HGNC Symbol;Acc:545]	10	75135203	75178834	291	0.033	1	1	5.401E-04	1.606E-03	PB-CAG-SD
NCOA5	nuclear receptor coactivator 5 [Source:HGNC Symbol;Acc:15909]	20	44689624	44723591	232	0.033	1	1	5.434E-04	1.612E-03	PB-CAG-SD
PPP1R2	protein phosphatase 1, regulatory (inhibitor) subunit 2 [Source:HGNC Symbol;Acc:9288]	3	195241221	195275209	281	0.034	1	1	5.496E-04	1.626E-03	PB-CAG-SD
ZBTB8OS	zinc finger and BTB domain containing 8 opposite strand [Source:HGNC Symbol;Acc:24094]	1	33065773	33121504	278	0.034	1	1	5.544E-04	1.635E-03	PB-CAG-SD
GRHL1	grainyhead-like 1 (Drosophila) [Source:HGNC Symbol;Acc:17923]	2	10080341	10142411	370	0.034	1	1	5.555E-04	1.635E-03	PB-CAG-SD
PFKP	phosphofructokinase, platelet [Source:HGNC Symbol;Acc:8878]	10	3104712	3179904	296	0.034	1	1	5.587E-04	1.641E-03	PB-CAG-SD
RNF10	ring finger protein 10 [Source:HGNC Symbol;Acc:10055]	12	120966283	121015397	295	0.034	1	1	5.701E-04	1.670E-03	PB-CAG-SD
MRC2	mannose receptor, C type 2 [Source:HGNC Symbol;Acc:16875]	17	60699762	60770958	224	0.034	1	1	5.760E-04	1.679E-03	PB-CAG-SD
MED1	mediator complex subunit 1 [Source:HGNC Symbol;Acc:9234]	17	37560538	37612539	224	0.034	1	1	5.760E-04	1.679E-03	PB-CAG-SD
PXT1	peroxisomal, testis specific 1 [Source:HGNC Symbol;Acc:18312]	6	36358328	36415666	351	0.035	1	1	5.938E-04	1.727E-03	PB-CAG-SD
DGKG	diacylglycerol kinase, gamma 90kDa [Source:HGNC Symbol;Acc:2853]	3	185823457	186085026	1338	0.160	2	1	6.018E-04	1.746E-03	PB-CAG-SD
JRKL-AS1	JRKL antisense RNA 1 [Source:HGNC Symbol;Acc:43670]	11	96180296	96244990	404	0.035	1	1	6.041E-04	1.748E-03	PB-CAG-SD
BTBD16	BTB (POZ) domain containing 16 [Source:HGNC Symbol;Acc:26340]	10	124025821	124097677	309	0.035	1	1	6.082E-04	1.756E-03	PB-CAG-SD
CAV1	caveolin 1, caveolae protein, 22kDa [Source:HGNC Symbol;Acc:1527]	7	116159839	116201233	296	0.036	1	1	6.158E-04	1.771E-03	PB-CAG-SD
METTL21A	methyltransferase like 21A [Source:HGNC Symbol;Acc:30476]	2	208445355	208495652	390	0.036	1	1	6.164E-04	1.771E-03	PB-CAG-SD
CLYBL	citrate lyase beta like [Source:HGNC Symbol;Acc:18355]	13	100253919	100549387	1869	0.161	2	1	6.205E-04	1.779E-03	PB-CAG-SD
SLCO3A1	solute carrier organic anion transporter family, member 3A1 [Source:HGNC Symbol;Acc:10952]	15	92391925	92715665	1566	0.162	2	1	6.259E-04	1.789E-03	PB-CAG-SD
SLC28A1	solute carrier family 28 (concentrative nucleoside transporter), member 1 [Source:HGNC Symbol;Acc:11001]	15	85422885	85518876	347	0.036	1	1	6.277E-04	1.789E-03	PB-CAG-SD
ZHX1	zinc fingers and homeoboxes 1 [Source:HGNC Symbol;Acc:12871]	8	124260696	124292781	291	0.036	1	1	6.285E-04	1.789E-03	PB-CAG-SD
SMAP1	small ArfGAP 1 [Source:HGNC Symbol;Acc:19651]	6	71372479	71571718	1635	0.162	2	1	6.323E-04	1.795E-03	PB-CAG-SD
HCP5	HLA complex P5 (non-protein coding) [Source:HGNC Symbol;Acc:21659]	6	31363479	31445283	363	0.036	1	1	6.346E-04	1.798E-03	PB-CAG-SD
GPNMB	glycoprotein (transmembrane) nmb [Source:HGNC Symbol;Acc:4462]	7	23270586	23314727	301	0.036	1	1	6.365E-04	1.799E-03	PB-CAG-SD
KIAA1430	KIAA1430 [Source:HGNC Symbol;Acc:29276]	4	186080819	186135658	410	0.036	1	1	6.386E-04	1.801E-03	PB-CAG-SD
MAP4K3	mitogen-activated protein kinase kinase kinase kinase 3 [Source:HGNC Symbol;Acc:6865]	2	39476407	39669453	1799	0.164	2	2	6.491E-04	1.826E-03	PB-CAG-SD
POLR2B	polymerase (RNA) II (DNA directed) polypeptide B, 140kDa [Source:HGNC Symbol;Acc:9188]	4	57838888	57897334	414	0.037	1	1	6.510E-04	1.827E-03	PB-CAG-SD
TRAM1	translocation associated membrane protein 1 [Source:HGNC Symbol;Acc:20568]	8	71485677	71525622	300	0.037	1	1	6.674E-04	1.867E-03	PB-CAG-SD
ZNF410	zinc finger protein 410 [Source:HGNC Symbol;Acc:20144]	14	74348320	74399214	338	0.037	1	1	6.682E-04	1.867E-03	PB-CAG-SD
MCM3AP	minichromosome maintenance complex component 3 associated protein [Source:HGNC Symbol;Acc:6946]	21	47655047	47711211	257	0.037	1	1	6.786E-04	1.891E-03	PB-CAG-SD
TTC5	tetratricopeptide repeat domain 5 [Source:HGNC Symbol;Acc:19274]	14	20724717	20779153	341	0.037	1	1	6.799E-04	1.891E-03	PB-CAG-SD
CCDC173	coiled-coil domain containing 173 [Source:HGNC Symbol;Acc:25064]	2	170501935	170555943	411	0.037	1	1	6.837E-04	1.897E-03	PB-CAG-SD
C2orf47	chromosome 2 open reading frame 47 [Source:HGNC Symbol;Acc:26198]	2	200815040	200873263	413	0.038	1	1	6.903E-04	1.911E-03	PB-CAG-SD
LINC00969	long intergenic non-protein coding RNA 969 [Source:HGNC Symbol;Acc:48729]	3	195379933	195460422	318	0.038	1	1	7.018E-04	1.934E-03	PB-CAG-SD
CPA3	carboxypeptidase A3 (mast cell) [Source:HGNC Symbol;Acc:2298]	3	148578043	148614983	318	0.038	1	1	7.018E-04	1.934E-03	PB-CAG-SD
CCP110	centriolar coiled coil protein 110kDa [Source:HGNC Symbol;Acc:24342]	16	19530133	19564730	291	0.038	1	1	7.036E-04	1.934E-03	PB-CAG-SD
LACTB2	lactamase, beta 2 [Source:HGNC Symbol;Acc:18512]	8	71547553	71586409	311	0.038	1	1	7.166E-04	1.959E-03	PB-CAG-SD
PDK3	pyruvate dehydrogenase kinase, isozyme 3 [Source:HGNC Symbol;Acc:8811]	X	24478338	24557954	559	0.038	1	1	7.169E-04	1.959E-03	PB-CAG-SD
EIF3A	eukaryotic translation initiation factor 3, subunit A [Source:HGNC Symbol;Acc:3271]	10	120794356	120845396	336	0.038	1	1	7.177E-04	1.959E-03	PB-CAG-SD
FHL2	four and a half LIM domains 2 [Source:HGNC Symbol;Acc:3703]	2	105974169	106059970	422	0.038	1	1	7.203E-04	1.960E-03	PB-CAG-SD
ACSL1	acyl-CoA synthetase long-chain family member 1 [Source:HGNC Symbol;Acc:3569]	4	185676749	185752972	436	0.038	1	1	7.211E-04	1.960E-03	PB-CAG-SD
TULP3	tubby like protein 3 [Source:HGNC Symbol;Acc:12425]	12	2981389	3050306	335	0.039	1	1	7.330E-04	1.988E-03	PB-CAG-SD
EOGT	EGF domain-specific O-linked N-acetylglucosamine (GlcNAc) transferase [Source:HGNC Symbol;Acc:28526]	3	69024365	69068112	326	0.039	1	1	7.371E-04	1.994E-03	PB-CAG-SD
ACAP2	ArfGAP with coiled-coil, ankyrin repeat and PH domains 2 [Source:HGNC Symbol;Acc:16469]	3	194995465	195168807	1438	0.172	2	1	7.405E-04	1.999E-03	PB-CAG-SD
TMEM39A	transmembrane protein 39A [Source:HGNC Symbol;Acc:25600]	3	119148347	119192677	328	0.039	1	1	7.460E-04	2.009E-03	PB-CAG-SD
C2orf69	chromosome 2 open reading frame 69 [Source:HGNC Symbol;Acc:26799]	2	200770979	200820658	430	0.039	1	1	7.475E-04	2.009E-03	PB-CAG-SD
FAM20B	family with sequence similarity 20, member B [Source:HGNC Symbol;Acc:23017]	1	178989939	179045697	324	0.039	1	1	7.503E-04	2.012E-03	PB-CAG-SD
MAPK9	mitogen-activated protein kinase 9 [Source:HGNC Symbol;Acc:6886]	5	179660143	179724099	372	0.040	1	1	7.606E-04	2.035E-03	PB-CAG-SD
ZNF33B	zinc finger protein 33B [Source:HGNC Symbol;Acc:13097]	10	43084555	43138992	348	0.040	1	1	7.691E-04	2.053E-03	PB-CAG-SD
IQSEC3	IQ motif and Sec7 domain 3 [Source:HGNC Symbol;Acc:29193]	12	170931	287626	344	0.040	1	1	7.723E-04	2.056E-03	PB-CAG-SD
ZNF33A	zinc finger protein 33A [Source:HGNC Symbol;Acc:13096]	10	38294578	38354016	349	0.040	1	1	7.735E-04	2.056E-03	PB-CAG-SD
VPS35	vacuolar protein sorting 35 homolog (S. cerevisiae) [Source:HGNC Symbol;Acc:13487]	16	46690054	46728430	306	0.040	1	1	7.770E-04	2.057E-03	PB-CAG-SD
PPP6R2	protein phosphatase 6, regulatory subunit 2 [Source:HGNC Symbol;Acc:19253]	22	50776733	50883514	334	0.040	1	1	7.772E-04	2.057E-03	PB-CAG-SD

table S2. Genes with significant transposon integrations in RKO *BRAF*$^{wt/-/-}$ cells after selection in low glucose medium

Gene ID	Description	Chrom	Start	End	# TTAA sites	Expected	Observed	Libraries	*P*	FDR	Transposon
ZNF713	zinc finger protein 713 [Source:HGNC Symbol;Acc:22043]	7	55950169	56009918	334	0.040	1	1	7.817E-04	2.064E-03	PB-CAG-SD
DSG2	desmoglein 2 [Source:HGNC Symbol;Acc:3049]	18	29073006	29128971	405	0.040	1	1	7.853E-04	2.068E-03	PB-CAG-SD
TMPRSS2	transmembrane protease, serine 2 [Source:HGNC Symbol;Acc:11876]	21	42836478	42908043	277	0.040	1	1	7.868E-04	2.068E-03	PB-CAG-SD
NUP35	nucleoporin 35kDa [Source:HGNC Symbol;Acc:29797]	2	183977241	184026408	443	0.040	1	1	7.928E-04	2.080E-03	PB-CAG-SD
TRIM55	tripartite motif containing 55 [Source:HGNC Symbol;Acc:14215]	8	67034131	67087720	329	0.041	1	1	8.008E-04	2.096E-03	PB-CAG-SD
TMX2-CTNND1	TMX2-CTNND1 readthrough (NMD candidate) [Source:HGNC Symbol;Acc:41992]	11	57475077	57559058	467	0.041	1	1	8.043E-04	2.101E-03	PB-CAG-SD
TM2D1	TM2 domain containing 1 [Source:HGNC Symbol;Acc:24142]	1	62146718	62196095	336	0.041	1	1	8.061E-04	2.101E-03	PB-CAG-SD
MAN2A1	mannosidase, alpha, class 2A, member 1 [Source:HGNC Symbol;Acc:6824]	5	109020067	109205326	1668	0.177	2	1	8.124E-04	2.113E-03	PB-CAG-SD
GARS	glycyl-tRNA synthetase [Source:HGNC Symbol;Acc:4162]	7	30629297	30673649	342	0.041	1	1	8.191E-04	2.125E-03	PB-CAG-SD
H2AFY	H2A histone family, member Y [Source:HGNC Symbol;Acc:4740]	5	134669590	134740604	388	0.041	1	1	8.265E-04	2.140E-03	PB-CAG-SD
ALG5	ALG5, dolichyl-phosphate beta-glucosyltransferase [Source:HGNC Symbol;Acc:20266]	13	37523912	37579398	480	0.041	1	1	8.351E-04	2.158E-03	PB-CAG-SD
ZNF695	zinc finger protein 695 [Source:HGNC Symbol;Acc:30954]	1	247108849	247176395	346	0.042	1	1	8.541E-04	2.202E-03	PB-CAG-SD
RPP30	ribonuclease P/MRP 30kDa subunit [Source:HGNC Symbol;Acc:17688]	10	92626473	92668312	369	0.042	1	1	8.634E-04	2.221E-03	PB-CAG-SD
ADAM17	ADAM metallopeptidase domain 17 [Source:HGNC Symbol;Acc:195]	2	9628615	9700921	464	0.042	1	1	8.686E-04	2.227E-03	PB-CAG-SD
TGFBR1	transforming growth factor, beta receptor 1 [Source:HGNC Symbol;Acc:11772]	9	101861320	101916474	429	0.042	1	1	8.693E-04	2.227E-03	PB-CAG-SD
METAP2	methionyl aminopeptidase 2 [Source:HGNC Symbol;Acc:16672]	12	95862296	95909615	367	0.042	1	1	8.775E-04	2.243E-03	PB-CAG-SD
MAOA	monoamine oxidase A [Source:HGNC Symbol;Acc:6833]	X	43510467	43606068	621	0.043	1	1	8.822E-04	2.250E-03	PB-CAG-SD
SLC17A5	solute carrier family 17 (anion/sugar transporter), member 5 [Source:HGNC Symbol;Acc:10933]	6	74303102	74368878	433	0.043	1	1	8.988E-04	2.283E-03	PB-CAG-SD
SYNJ2	synaptojanin 2 [Source:HGNC Symbol;Acc:11504]	6	158397888	158520208	433	0.043	1	1	8.988E-04	2.283E-03	PB-CAG-SD
CSNK2A2	casein kinase 2, alpha prime polypeptide [Source:HGNC Symbol;Acc:2459]	16	58191811	58236824	330	0.043	1	1	9.017E-04	2.286E-03	PB-CAG-SD
GOLPH3	golgi phosphoprotein 3 (coat-protein) [Source:HGNC Symbol;Acc:15452]	5	32124810	32179456	408	0.043	1	1	9.127E-04	2.308E-03	PB-CAG-SD
CNGB3	cyclic nucleotide gated channel beta 3 [Source:HGNC Symbol;Acc:2153]	8	87566205	87760903	1501	0.185	2	2	9.201E-04	2.322E-03	PB-CAG-SD
ZNF677	zinc finger protein 677 [Source:HGNC Symbol;Acc:28730]	19	53731577	53763151	200	0.044	1	1	9.220E-04	2.322E-03	PB-CAG-SD
MYO18A	myosin XVIIIA [Source:HGNC Symbol;Acc:31104]	17	27400528	27512430	286	0.044	1	1	9.330E-04	2.341E-03	PB-CAG-SD
HDHD1	haloacid dehalogenase-like hydrolase domain containing 1 [Source:HGNC Symbol;Acc:16818]	X	6966961	7071231	639	0.044	1	1	9.333E-04	2.341E-03	PB-CAG-SD
CT60	cancer/testis antigen 60 (non-protein coding) [Source:HGNC Symbol;Acc:28003]	15	21140767	21198632	427	0.044	1	1	9.453E-04	2.366E-03	PB-CAG-SD
FAM151B	family with sequence similarity 151, member B [Source:HGNC Symbol;Acc:33716]	5	79778788	79838382	416	0.044	1	1	9.483E-04	2.369E-03	PB-CAG-SD
HS3ST3B1	heparan sulfate (glucosamine) 3-O-sulfotransferase 3B1 [Source:HGNC Symbol;Acc:5198]	17	14199400	14252721	289	0.044	1	1	9.524E-04	2.370E-03	PB-CAG-SD
ARHGEF37	Rho guanine nucleotide exchange factor (GEF) 37 [Source:HGNC Symbol;Acc:34430]	5	148926510	149014531	417	0.044	1	1	9.528E-04	2.370E-03	PB-CAG-SD
E2F5	E2F transcription factor 5, p130-binding [Source:HGNC Symbol;Acc:3119]	8	86084460	86129387	361	0.045	1	1	9.616E-04	2.387E-03	PB-CAG-SD
ZHX1-C8ORF76	ZHX1-C8ORF76 readthrough [Source:HGNC Symbol;Acc:42975]	8	124238431	124284627	362	0.045	1	1	9.669E-04	2.395E-03	PB-CAG-SD
PRPF4B	PRP4 pre-mRNA processing factor 4 homolog B (yeast) [Source:HGNC Symbol;Acc:17346]	6	4016501	4065217	450	0.045	1	1	9.696E-04	2.396E-03	PB-CAG-SD
EDNRA	endothelin receptor type A [Source:HGNC Symbol;Acc:3179]	4	148397069	148466106	507	0.045	1	1	9.710E-04	2.396E-03	PB-CAG-SD
PWWP2A	PWWP domain containing 2A [Source:HGNC Symbol;Acc:29406]	5	159488808	159551430	422	0.045	1	1	9.754E-04	2.402E-03	PB-CAG-SD
GNAI3	guanine nucleotide binding protein (G protein), alpha inhibiting activity polypeptide 3 [Source:HGNC Symbol;Acc:4387]	1	110086233	110136975	373	0.045	1	1	9.905E-04	2.434E-03	PB-CAG-SD
AAGAB	alpha- and gamma-adaptin binding protein [Source:HGNC Symbol;Acc:25662]	15	67493371	67552533	443	0.046	1	1	1.016E-03	2.492E-03	PB-CAG-SD
C2CD2	C2 calcium-dependent domain containing 2 [Source:HGNC Symbol;Acc:1266]	21	43305221	43378999	316	0.046	1	1	1.020E-03	2.495E-03	PB-CAG-SD
POLR3G	polymerase (RNA) III (DNA directed) polypeptide G (32kD) [Source:HGNC Symbol;Acc:30075]	5	89762565	89810370	432	0.046	1	1	1.021E-03	2.495E-03	PB-CAG-SD
SLC17A8	solute carrier family 17 (sodium-dependent inorganic phosphate cotransporter), member 8 [Source:HGNC Symbol;Acc:20151]	12	100745857	100815837	397	0.046	1	1	1.024E-03	2.497E-03	PB-CAG-SD
TBC1D2B	TBC1 domain family, member 2B [Source:HGNC Symbol;Acc:29183]	15	78276378	78375066	448	0.046	1	1	1.039E-03	2.523E-03	PB-CAG-SD
CLSTN2	calsyntenin 2 [Source:HGNC Symbol;Acc:17448]	3	139649027	140296239	3630	0.433	3	2	1.039E-03	2.523E-03	PB-CAG-SD
DPH5	diphthamide biosynthesis 5 [Source:HGNC Symbol;Acc:24270]	1	101455179	101496644	386	0.047	1	1	1.060E-03	2.567E-03	PB-CAG-SD
ACADSB	acyl-CoA dehydrogenase, short/branched chain [Source:HGNC Symbol;Acc:91]	10	124763495	124817827	410	0.047	1	1	1.063E-03	2.569E-03	PB-CAG-SD
ZNF767	zinc finger family member 767 [Source:HGNC Symbol;Acc:21884]	7	149244245	149326843	391	0.047	1	1	1.066E-03	2.573E-03	PB-CAG-SD
KIAA0226	KIAA0226 [Source:HGNC Symbol;Acc:28991]	3	197398264	197481598	394	0.047	1	1	1.071E-03	2.575E-03	PB-CAG-SD
CSGALNACT2	chondroitin sulfate N-acetylgalactosaminyltransferase 2 [Source:HGNC Symbol;Acc:24292]	10	43628934	43680756	412	0.047	1	1	1.073E-03	2.575E-03	PB-CAG-SD
TERF1	telomeric repeat binding factor (NIMA-interacting) 1 [Source:HGNC Symbol;Acc:11728]	8	73916099	73960357	382	0.047	1	1	1.075E-03	2.575E-03	PB-CAG-SD
STX6	syntaxin 6 [Source:HGNC Symbol;Acc:11441]	1	180941861	180997047	389	0.047	1	1	1.076E-03	2.575E-03	PB-CAG-SD
HGSNAT	heparan-alpha-glucosaminide N-acetyltransferase [Source:HGNC Symbol;Acc:26527]	8	42990556	43057998	384	0.047	1	1	1.086E-03	2.594E-03	PB-CAG-SD
IPO5	importin 5 [Source:HGNC Symbol;Acc:6402]	13	98600912	98676551	553	0.048	1	1	1.104E-03	2.632E-03	PB-CAG-SD
CDC20B	cell division cycle 20B [Source:HGNC Symbol;Acc:24222]	5	54408799	54474005	450	0.048	1	1	1.107E-03	2.633E-03	PB-CAG-SD
C1orf185	chromosome 1 open reading frame 185 [Source:HGNC Symbol;Acc:28096]	1	51562906	51613752	395	0.048	1	1	1.109E-03	2.633E-03	PB-CAG-SD
MRPL3	mitochondrial ribosomal protein L3 [Source:HGNC Symbol;Acc:10379]	3	131181056	131226827	403	0.048	1	1	1.120E-03	2.653E-03	PB-CAG-SD
DDX31	DEAD (Asp-Glu-Ala-Asp) box polypeptide 31 [Source:HGNC Symbol;Acc:16715]	9	135468384	135550788	489	0.048	1	1	1.125E-03	2.661E-03	PB-CAG-SD
EXOC1	exocyst complex component 1 [Source:HGNC Symbol;Acc:30380]	4	56714782	56771200	547	0.048	1	1	1.128E-03	2.662E-03	PB-CAG-SD
MRVI1	murine retrovirus integration site 1 homolog [Source:HGNC Symbol;Acc:7237]	11	10594638	10720535	555	0.048	1	1	1.130E-03	2.663E-03	PB-CAG-SD
ITGAM	integrin, alpha M (complement component 3 receptor 3 subunit) [Source:HGNC Symbol;Acc:6149]	16	31266311	31344213	371	0.048	1	1	1.136E-03	2.671E-03	PB-CAG-SD
MAP3K9	mitogen-activated protein kinase kinase kinase 9 [Source:HGNC Symbol;Acc:6861]	14	71189243	71281251	444	0.049	1	1	1.144E-03	2.685E-03	PB-CAG-SD
COPS5	COP9 signalosome subunit 5 [Source:HGNC Symbol;Acc:2240]	8	67955314	68001018	396	0.049	1	1	1.154E-03	2.688E-03	PB-CAG-SD
ACOT12	acyl-CoA thioesterase 12 [Source:HGNC Symbol;Acc:24436]	5	80625824	80694998	460	0.049	1	1	1.156E-03	2.688E-03	PB-CAG-SD
ZNF366	zinc finger protein 366 [Source:HGNC Symbol;Acc:18316]	5	71738479	71808554	460	0.049	1	1	1.156E-03	2.688E-03	PB-CAG-SD
GALNTL5	UDP-N-acetyl-alpha-D-galactosamine:polypeptide N-acetylgalactosaminyltransferase-like 5 [Source:HGNC Symbol;Acc:21725]	7	151648464	151717019	408	0.049	1	1	1.160E-03	2.688E-03	PB-CAG-SD
LYPLA1	lysophospholipase I [Source:HGNC Symbol;Acc:6737]	8	54958938	55019577	397	0.049	1	1	1.160E-03	2.688E-03	PB-CAG-SD
SBSPON	somatomedin B and thrombospondin, type 1 domain containing [Source:HGNC Symbol;Acc:30362]	8	73976775	74041323	397	0.049	1	1	1.160E-03	2.688E-03	PB-CAG-SD
SGTB	small glutamine-rich tetratricopeptide repeat (TPR)-containing, beta [Source:HGNC Symbol;Acc:23567]	5	64961755	65023862	461	0.049	1	1	1.161E-03	2.688E-03	PB-CAG-SD
SIN3A	SIN3 transcription regulator homolog A (yeast) [Source:HGNC Symbol;Acc:19353]	15	75661720	75753183	475	0.049	1	1	1.166E-03	2.694E-03	PB-CAG-SD
HAT1	histone acetyltransferase 1 [Source:HGNC Symbol;Acc:4821]	2	172773958	172848599	543	0.049	1	1	1.184E-03	2.731E-03	PB-CAG-SD
DGKE	diacylglycerol kinase, epsilon 64kDa [Source:HGNC Symbol;Acc:2852]	17	54906460	54946036	325	0.050	1	1	1.200E-03	2.763E-03	PB-CAG-SD
PRR5-ARHGAP8	PRR5-ARHGAP8 readthrough [Source:HGNC Symbol;Acc:34512]	22	45093113	45258586	418	0.050	1	1	1.209E-03	2.773E-03	PB-CAG-SD
ARHGAP8	Rho GTPase activating protein 8 [Source:HGNC Symbol;Acc:677]	22	45093355	45258665	418	0.050	1	1	1.209E-03	2.773E-03	PB-CAG-SD
C17orf67	chromosome 17 open reading frame 67 [Source:HGNC Symbol;Acc:27900]	17	54869274	54921134	329	0.050	1	1	1.229E-03	2.814E-03	PB-CAG-SD
SHB	Src homology 2 domain containing adaptor protein B [Source:HGNC Symbol;Acc:10838]	9	37919131	38074210	514	0.051	1	1	1.241E-03	2.835E-03	PB-CAG-SD
ANKRD46	ankyrin repeat domain 46 [Source:HGNC Symbol;Acc:27229]	8	101521980	101577012	412	0.051	1	1	1.247E-03	2.844E-03	PB-CAG-SD
TTC19	tetratricopeptide repeat domain 19 [Source:HGNC Symbol;Acc:26006]	17	15897694	15948329	332	0.051	1	1	1.251E-03	2.848E-03	PB-CAG-SD
GLTSCR1L	GLTSCR1-like [Source:HGNC Symbol;Acc:21111]	6	42744765	42836298	519	0.052	1	1	1.284E-03	2.913E-03	PB-CAG-SD
NLRC5	NLR family, CARD domain containing 5 [Source:HGNC Symbol;Acc:29933]	16	57018397	57117443	395	0.052	1	1	1.285E-03	2.913E-03	PB-CAG-SD
MSRB3	methionine sulfoxide reductase B3 [Source:HGNC Symbol;Acc:27375]	12	65667423	65882024	1798	0.208	2	2	1.287E-03	2.913E-03	PB-CAG-SD
C11orf80	chromosome 11 open reading frame 80 [Source:HGNC Symbol;Acc:26197]	11	66506922	66610987	594	0.052	1	1	1.292E-03	2.918E-03	PB-CAG-SD
SEPT10	septin 10 [Source:HGNC Symbol;Acc:14349]	2	110300559	110376783	571	0.052	1	1	1.307E-03	2.947E-03	PB-CAG-SD
SYK	spleen tyrosine kinase [Source:HGNC Symbol;Acc:11491]	9	93559069	93660831	530	0.052	1	1	1.318E-03	2.966E-03	PB-CAG-SD
DIS3L2	DIS3 mitotic control homolog (S. cerevisiae)-like 2 [Source:HGNC Symbol;Acc:28648]	2	232820955	233209060	2313	0.211	2	1	1.333E-03	2.991E-03	PB-CAG-SD
CHD6	chromodomain helicase DNA binding protein 6 [Source:HGNC Symbol;Acc:19057]	20	40030741	40252133	1467	0.211	2	1	1.334E-03	2.991E-03	PB-CAG-SD
KLRG1	killer cell lectin-like receptor subfamily G, member 1 [Source:HGNC Symbol;Acc:6380]	12	9097640	9163356	456	0.053	1	1	1.345E-03	3.011E-03	PB-CAG-SD
CPSF6	cleavage and polyadenylation specific factor 6, 68kDa [Source:HGNC Symbol;Acc:13871]	12	69628317	69668138	457	0.053	1	1	1.351E-03	3.016E-03	PB-CAG-SD
ZNF788	zinc finger family member 788 [Source:HGNC Symbol;Acc:33112]	19	12198078	12248050	243	0.053	1	1	1.353E-03	3.016E-03	PB-CAG-SD
PDE9A	phosphodiesterase 9A [Source:HGNC Symbol;Acc:8795]	21	44068746	44195619	367	0.053	1	1	1.369E-03	3.047E-03	PB-CAG-SD
CSDE1	cold shock domain containing E1, RNA-binding [Source:HGNC Symbol;Acc:29905]	1	115259534	115306297	441	0.053	1	1	1.377E-03	3.052E-03	PB-CAG-SD
TARSL2	threonyl-tRNA synthetase-like 2 [Source:HGNC Symbol;Acc:24728]	15	102193801	102269807	517	0.053	1	1	1.377E-03	3.052E-03	PB-CAG-SD
SMIM14	small integral membrane protein 14 [Source:HGNC Symbol;Acc:27321]	4	39552541	39645710	606	0.053	1	1	1.379E-03	3.052E-03	PB-CAG-SD
MCCC2	methylcrotonoyl-CoA carboxylase 2 (beta) [Source:HGNC Symbol;Acc:6937]	5	70878115	70954531	505	0.054	1	1	1.389E-03	3.068E-03	PB-CAG-SD
SLX4IP	SLX4 interacting protein [Source:HGNC Symbol;Acc:16225]	20	10410951	10617477	1489	0.214	2	1	1.391E-03	3.068E-03	PB-CAG-SD
ZNF85	zinc finger protein 85 [Source:HGNC Symbol;Acc:13160]	19	21101028	21133503	247	0.054	1	1	1.397E-03	3.074E-03	PB-CAG-SD
HEG1	heart development protein with EGF-like domains 1 [Source:HGNC Symbol;Acc:29227]	3	124684554	124779802	452	0.054	1	1	1.403E-03	3.082E-03	PB-CAG-SD
SHFM1	split hand/foot malformation (ectrodactyly) type 1 [Source:HGNC Symbol;Acc:10845]	7	96110938	96344203	1802	0.216	2	1	1.433E-03	3.143E-03	PB-CAG-SD
ZSWIM5	zinc finger, SWIM-type containing 5 [Source:HGNC Symbol;Acc:29299]	1	45482071	45776881	1801	0.218	2	2	1.471E-03	3.219E-03	PB-CAG-SD
NUP107	nucleoporin 107kDa [Source:HGNC Symbol;Acc:29914]	12	69075514	69136785	478	0.055	1	1	1.476E-03	3.225E-03	PB-CAG-SD
UST	uronyl-2-sulfotransferase [Source:HGNC Symbol;Acc:17223]	6	149063464	149398126	2202	0.219	2	1	1.481E-03	3.231E-03	PB-CAG-SD
PPP3CB	protein phosphatase 3, catalytic subunit, beta isozyme [Source:HGNC Symbol;Acc:9315]	10	75196186	75260782	489	0.056	1	1	1.503E-03	3.271E-03	PB-CAG-SD
CCDC66	coiled-coil domain containing 66 [Source:HGNC Symbol;Acc:27709]	3	56586189	56655846	470	0.056	1	1	1.515E-03	3.292E-03	PB-CAG-SD
ZNF20	zinc finger protein 20 [Source:HGNC Symbol;Acc:12992]	19	12203658	12256222	258	0.056	1	1	1.522E-03	3.298E-03	PB-CAG-SD
CCDC15	coiled-coil domain containing 15 [Source:HGNC Symbol;Acc:25798]	11	124819017	124911385	646	0.056	1	1	1.523E-03	3.298E-03	PB-CAG-SD
HSPA4L	heat shock 70kDa protein 4-like [Source:HGNC Symbol;Acc:17041]	4	128697976	128755226	641	0.057	1	1	1.540E-03	3.329E-03	PB-CAG-SD
SREK1	splicing regulatory glutamine/lysine-rich protein 1 [Source:HGNC Symbol;Acc:17882]	5	65430799	65479443	533	0.057	1	1	1.544E-03	3.331E-03	PB-CAG-SD
SMAD3	SMAD family member 3 [Source:HGNC Symbol;Acc:6769]	15	67351101	67487533	549	0.057	1	1	1.550E-03	3.338E-03	PB-CAG-SD
ATP6V1C1	ATPase, H+ transporting, lysosomal 42kDa, V1 subunit C1 [Source:HGNC Symbol;Acc:856]	8	104028291	104085279	461	0.057	1	1	1.555E-03	3.344E-03	PB-CAG-SD
LRIG3	leucine-rich repeats and immunoglobulin-like domains 3 [Source:HGNC Symbol;Acc:30991]	12	59265931	59319303	493	0.057	1	1	1.568E-03	3.364E-03	PB-CAG-SD
HCG17	HLA complex group 17 (non-protein coding) [Source:HGNC Symbol;Acc:31339]	6	30201816	30298911	575	0.057	1	1	1.570E-03	3.364E-03	PB-CAG-SD
HEXB	hexosaminidase B (beta polypeptide) [Source:HGNC Symbol;Acc:4879]	5	73930848	74018472	543	0.058	1	1	1.601E-03	3.424E-03	PB-CAG-SD
NPTN	neuroplastin [Source:HGNC Symbol;Acc:17867]	15	73852355	73931475	559	0.058	1	1	1.606E-03	3.424E-03	PB-CAG-SD
KLHL8	kelch-like family member 8 [Source:HGNC Symbol;Acc:18644]	4	88081255	88166466	655	0.058	1	1	1.607E-03	3.424E-03	PB-CAG-SD
CROT	carnitine O-octanoyltransferase [Source:HGNC Symbol;Acc:2366]	7	86969997	87029111	484	0.058	1	1	1.622E-03	3.450E-03	PB-CAG-SD
NUP155	nucleoporin 155kDa [Source:HGNC Symbol;Acc:8063]	5	37288239	37376283	549	0.058	1	1	1.636E-03	3.472E-03	PB-CAG-SD
BRD7	bromodomain containing 7 [Source:HGNC Symbol;Acc:14310]	16	50347398	50407845	447	0.058	1	1	1.638E-03	3.472E-03	PB-CAG-SD
RPL9P8	ribosomal protein L9 pseudogene 8 [Source:HGNC Symbol;Acc:30334]	15	83041000	83130316	566	0.058	1	1	1.645E-03	3.481E-03	PB-CAG-SD
RPL9P9	ribosomal protein L9 pseudogene 9 [Source:HGNC Symbol;Acc:17251]	15	82664459	82753784	567	0.059	1	1	1.651E-03	3.487E-03	PB-CAG-SD
CSNK1A1	casein kinase 1, alpha 1 [Source:HGNC Symbol;Acc:2451]	5	148871760	148936007	553	0.059	1	1	1.660E-03	3.499E-03	PB-CAG-SD
SUFU	suppressor of fused homolog (Drosophila) [Source:HGNC Symbol;Acc:16466]	10	104258744	104393292	515	0.059	1	1	1.663E-03	3.499E-03	PB-CAG-SD
FN1	fibronectin 1 [Source:HGNC Symbol;Acc:3778]	2	216225163	216305895	646	0.059	1	1	1.665E-03	3.499E-03	PB-CAG-SD
ZNF708	zinc finger protein 708 [Source:HGNC Symbol;Acc:12945]	19	21473962	21517227	271	0.059	1	1	1.676E-03	3.515E-03	PB-CAG-SD
RCL1	RNA terminal phosphate cyclase-like 1 [Source:HGNC Symbol;Acc:17687]	9	4787869	4885917	601	0.059	1	1	1.687E-03	3.533E-03	PB-CAG-SD
TPX2	TPX2, microtubule-associated, homolog (Xenopus laevis) [Source:HGNC Symbol;Acc:1249]	20	30322074	30389608	413	0.059	1	1	1.693E-03	3.538E-03	PB-CAG-SD
TSPAN18	tetraspanin 18 [Source:HGNC Symbol;Acc:20660]	11	44743015	44953972	684	0.060	1	1	1.704E-03	3.555E-03	PB-CAG-SD
CCPG1	cell cycle progression 1 [Source:HGNC Symbol;Acc:24227]	15	55632230	55705708	579	0.060	1	1	1.720E-03	3.583E-03	PB-CAG-SD
ZNF793	zinc finger protein 793 [Source:HGNC Symbol;Acc:33115]	19	37992841	38039664	276	0.060	1	1	1.737E-03	3.611E-03	PB-CAG-SD
NEMF	nuclear export mediator factor [Source:HGNC Symbol;Acc:10663]	14	50249997	50324921	550	0.060	1	1	1.742E-03	3.616E-03	PB-CAG-SD
SCML2	sex comb on midleg-like 2 (Drosophila) [Source:HGNC Symbol;Acc:10581]	X	18257434	18377847	880	0.060	1	1	1.751E-03	3.628E-03	PB-CAG-SD
STAG2	stromal antigen 2 [Source:HGNC Symbol;Acc:11355]	X	123089062	123556514	3408	0.234	2	2	1.789E-03	3.702E-03	PB-CAG-SD
HK1	hexokinase 1 [Source:HGNC Symbol;Acc:4922]	10	71024740	71161638	536	0.061	1	1	1.799E-03	3.715E-03	PB-CAG-SD
TPO	thyroid peroxidase [Source:HGNC Symbol;Acc:12015]	2	1372995	1547483	674	0.061	1	1	1.810E-03	3.725E-03	PB-CAG-SD
DHRS7B	dehydrogenase/reductase (SDR family) member 7B [Source:HGNC Symbol;Acc:24547]	17	21021677	21096578	401	0.061	1	1	1.813E-03	3.725E-03	PB-CAG-SD
RPA1	replication protein A1, 70kDa [Source:HGNC Symbol;Acc:10289]	17	1727996	1803376	401	0.061	1	1	1.813E-03	3.725E-03	PB-CAG-SD
MCOLN2	mucolipin 2 [Source:HGNC Symbol;Acc:13357]	1	85391268	85467796	510	0.062	1	1	1.831E-03	3.756E-03	PB-CAG-SD
CDC42BPB	CDC42 binding protein kinase beta (DMPK-like) [Source:HGNC Symbol;Acc:1738]	14	103398716	103528799	565	0.062	1	1	1.836E-03	3.760E-03	PB-CAG-SD
ISM1	isthmin 1, angiogenesis inhibitor [Source:HGNC Symbol;Acc:16213]	20	13197418	13281298	431	0.062	1	1	1.840E-03	3.761E-03	PB-CAG-SD
SLC25A24	solute carrier family 25 (mitochondrial carrier; phosphate carrier), member 24 [Source:HGNC Symbol;Acc:20662]	1	108676658	108748471	512	0.062	1	1	1.845E-03	3.761E-03	PB-CAG-SD
KIAA1467	KIAA1467 [Source:HGNC Symbol;Acc:29288]	12	13192218	13295455	536	0.062	1	1	1.848E-03	3.761E-03	PB-CAG-SD
ME2	malic enzyme 2, NAD(+)-dependent, mitochondrial [Source:HGNC Symbol;Acc:6984]	18	48400419	48474691	626	0.062	1	1	1.849E-03	3.761E-03	PB-CAG-SD
PPP4R1	protein phosphatase 4, regulatory subunit 1 [Source:HGNC Symbol;Acc:9320]	18	9546789	9620238	628	0.062	1	1	1.861E-03	3.778E-03	PB-CAG-SD

table S2. Genes with significant transposon integrations in RKO *BRAF*$^{wt/-/-}$ cells after selection in low glucose medium

Gene ID	Description	Chrom	Start	End	# TTAA sites	Expected	Observed	Libraries	*P*	FDR	Transposon
TMEM200A	transmembrane protein 200A [Source:HGNC Symbol;Acc:21075]	6	130681879	130764208	630	0.063	1	1	1.878E-03	3.807E-03	PB-CAG-SD
RPSAP52	ribosomal protein SA pseudogene 52 [Source:HGNC Symbol;Acc:35752]	12	66151800	66225754	543	0.063	1	1	1.895E-03	3.830E-03	PB-CAG-SD
GK5	glycerol kinase 5 (putative) [Source:HGNC Symbol;Acc:28635]	3	141882414	141949449	527	0.063	1	1	1.896E-03	3.830E-03	PB-CAG-SD
PARVB	parvin, beta [Source:HGNC Symbol;Acc:14653]	22	44390091	44565106	528	0.063	1	1	1.913E-03	3.857E-03	PB-CAG-SD
SIM1	single-minded homolog 1 (Drosophila) [Source:HGNC Symbol;Acc:10882]	6	100832891	100917805	637	0.063	1	1	1.919E-03	3.864E-03	PB-CAG-SD
CHODL-AS1	CHODL antisense RNA 1 [Source:HGNC Symbol;Acc:1279]	21	19207333	19262925	437	0.063	1	1	1.928E-03	3.869E-03	PB-CAG-SD
TTC26	tetratricopeptide repeat domain 26 [Source:HGNC Symbol;Acc:21882]	7	138813490	138876732	529	0.063	1	1	1.931E-03	3.869E-03	PB-CAG-SD
UBE2U	ubiquitin-conjugating enzyme E2U (putative) [Source:HGNC Symbol;Acc:28559]	1	64664310	64733053	524	0.063	1	1	1.931E-03	3.869E-03	PB-CAG-SD
THBS4	thrombospondin 4 [Source:HGNC Symbol;Acc:11788]	5	79282134	79379110	604	0.064	1	1	1.973E-03	3.945E-03	PB-CAG-SD
SLC1A3	solute carrier family 1 (glial high affinity glutamate transporter), member 3 [Source:HGNC Symbol;Acc:10941]	5	36601457	36688436	609	0.065	1	1	2.005E-03	4.003E-03	PB-CAG-SD
NAA25	N(alpha)-acetyltransferase 25, NatB auxiliary subunit [Source:HGNC Symbol;Acc:25783]	12	112464500	112551826	560	0.065	1	1	2.013E-03	4.013E-03	PB-CAG-SD
PTPN3	protein tyrosine phosphatase, non-receptor type 3 [Source:HGNC Symbol;Acc:9655]	9	112137746	112265590	661	0.065	1	1	2.033E-03	4.045E-03	PB-CAG-SD
ACTR3	ARP3 actin-related protein 3 homolog (yeast) [Source:HGNC Symbol;Acc:170]	2	114642537	114720173	716	0.065	1	1	2.037E-03	4.047E-03	PB-CAG-SD
FBXL5	F-box and leucine-rich repeat protein 5 [Source:HGNC Symbol;Acc:13602]	4	15606162	15688302	751	0.066	1	1	2.100E-03	4.166E-03	PB-CAG-SD
EGLN1	egl nine homolog 1 (C. elegans) [Source:HGNC Symbol;Acc:1232]	1	231499497	231565790	549	0.067	1	1	2.116E-03	4.189E-03	PB-CAG-SD
MTF2	metal response element binding transcription factor 2 [Source:HGNC Symbol;Acc:29535]	1	93539792	93604638	551	0.067	1	1	2.131E-03	4.201E-03	PB-CAG-SD
HS2ST1	heparan sulfate 2-O-sulfotransferase 1 [Source:HGNC Symbol;Acc:5193]	1	87375331	87602334	2054	0.249	2	2	2.132E-03	4.201E-03	PB-CAG-SD
PTPN1	protein tyrosine phosphatase, non-receptor type 1 [Source:HGNC Symbol;Acc:9642]	20	49121891	49201299	465	0.067	1	1	2.135E-03	4.201E-03	PB-CAG-SD
TAS2R1	taste receptor, type 2, member 1 [Source:HGNC Symbol;Acc:14909]	5	9629109	9717490	629	0.067	1	1	2.136E-03	4.201E-03	PB-CAG-SD
TOPBP1	topoisomerase (DNA) II binding protein 1 [Source:HGNC Symbol;Acc:17008]	3	133317019	133385737	562	0.067	1	1	2.150E-03	4.223E-03	PB-CAG-SD
RAB7A	RAB7A, member RAS oncogene family [Source:HGNC Symbol;Acc:9788]	3	128439965	128533639	563	0.067	1	1	2.157E-03	4.225E-03	PB-CAG-SD
SLC37A3	solute carrier family 37, member 3 [Source:HGNC Symbol;Acc:20651]	7	139993493	140109233	560	0.067	1	1	2.158E-03	4.225E-03	PB-CAG-SD
EIF3E	eukaryotic translation initiation factor 3, subunit E [Source:HGNC Symbol;Acc:3277]	8	109213445	109452562	2029	0.250	2	1	2.166E-03	4.233E-03	PB-CAG-SD
SLCO4C1	solute carrier organic anion transporter family, member 4C1 [Source:HGNC Symbol;Acc:23612]	5	101569690	101637253	640	0.068	1	1	2.209E-03	4.311E-03	PB-CAG-SD
PPP1R13B	protein phosphatase 1, regulatory subunit 13B [Source:HGNC Symbol;Acc:14950]	14	104200089	104318927	622	0.068	1	1	2.217E-03	4.318E-03	PB-CAG-SD
TMEM67	transmembrane protein 67 [Source:HGNC Symbol;Acc:28396]	8	94762072	94831462	553	0.068	1	1	2.221E-03	4.321E-03	PB-CAG-SD
NUP153	nucleoporin 153kDa [Source:HGNC Symbol;Acc:8062]	6	17615269	17711656	687	0.068	1	1	2.225E-03	4.321E-03	PB-CAG-SD
PAPSS2	3'-phosphoadenosine 5'-phosphosulfate synthase 2 [Source:HGNC Symbol;Acc:8604]	10	89414370	89507462	600	0.069	1	1	2.243E-03	4.349E-03	PB-CAG-SD
ETS1	v-ets avian erythroblastosis virus E26 oncogene homolog 1 [Source:HGNC Symbol;Acc:3488]	11	128328656	128462437	795	0.069	1	1	2.287E-03	4.427E-03	PB-CAG-SD
IGF1	insulin-like growth factor 1 (somatomedin C) [Source:HGNC Symbol;Acc:5464]	12	102789645	102879423	600	0.069	1	1	2.304E-03	4.452E-03	PB-CAG-SD
HDAC2	histone deacetylase 2 [Source:HGNC Symbol;Acc:4853]	6	114254192	114337472	701	0.070	1	1	2.314E-03	4.466E-03	PB-CAG-SD
MPP7	membrane protein, palmitoylated 7 (MAGUK p55 subfamily member 7) [Source:HGNC Symbol;Acc:26542]	10	28339922	28628415	2246	0.257	2	1	2.323E-03	4.476E-03	PB-CAG-SD
NDEL1	nudE nuclear distribution E homolog (A. nidulans)-like 1 [Source:HGNC Symbol;Acc:17620]	17	8311449	8393729	456	0.070	1	1	2.331E-03	4.484E-03	PB-CAG-SD
BLM	Bloom syndrome, RecQ helicase-like [Source:HGNC Symbol;Acc:1058]	15	91255558	91358859	679	0.070	1	1	2.349E-03	4.511E-03	PB-CAG-SD
RAD54L2	RAD54-like 2 (S. cerevisiae) [Source:HGNC Symbol;Acc:29123]	3	51570160	51697610	589	0.070	1	1	2.357E-03	4.518E-03	PB-CAG-SD
PDE7B	phosphodiesterase 7B [Source:HGNC Symbol;Acc:8792]	6	136167834	136516712	2605	0.259	2	1	2.381E-03	4.557E-03	PB-CAG-SD
PHKA1	phosphorylase kinase, alpha 1 (muscle) [Source:HGNC Symbol;Acc:8925]	X	71798664	71939167	1032	0.071	1	1	2.391E-03	4.569E-03	PB-CAG-SD
GPR133	G protein-coupled receptor 133 [Source:HGNC Symbol;Acc:19893]	12	131433452	131626014	612	0.071	1	1	2.395E-03	4.569E-03	PB-CAG-SD
BEND7	BEN domain containing 7 [Source:HGNC Symbol;Acc:23514]	10	13480484	13575974	621	0.071	1	1	2.399E-03	4.570E-03	PB-CAG-SD
FPR1	formyl peptide receptor 1 [Source:HGNC Symbol;Acc:3826]	19	52248425	52312363	326	0.071	1	1	2.406E-03	4.575E-03	PB-CAG-SD
VKORC1L1	vitamin K epoxide reductase complex, subunit 1-like 1 [Source:HGNC Symbol;Acc:21492]	7	65333254	65424550	595	0.071	1	1	2.430E-03	4.611E-03	PB-CAG-SD
PTPRR	protein tyrosine phosphatase, receptor type, R [Source:HGNC Symbol;Acc:9680]	12	71031853	71319623	2253	0.261	2	2	2.435E-03	4.611E-03	PB-CAG-SD
DNAH7	dynein, axonemal, heavy chain 7 [Source:HGNC Symbol;Acc:18661]	2	196602427	196938536	2864	0.261	2	1	2.437E-03	4.611E-03	PB-CAG-SD
UGT8	UDP glycosyltransferase 8 [Source:HGNC Symbol;Acc:12555]	4	115514611	115599380	811	0.072	1	1	2.441E-03	4.611E-03	PB-CAG-SD
OXNAD1	oxidoreductase NAD-binding domain containing 1 [Source:HGNC Symbol;Acc:25128]	3	16301706	16391806	600	0.072	1	1	2.443E-03	4.611E-03	PB-CAG-SD
MKX	mohawk homeobox [Source:HGNC Symbol;Acc:23729]	10	27961804	28039989	632	0.072	1	1	2.483E-03	4.676E-03	PB-CAG-SD
KLHL7	kelch-like family member 7 [Source:HGNC Symbol;Acc:15646]	7	23140353	23215040	602	0.072	1	1	2.486E-03	4.676E-03	PB-CAG-SD
TNFRSF19	tumor necrosis factor receptor superfamily, member 19 [Source:HGNC Symbol;Acc:11915]	13	24139509	24250232	842	0.073	1	1	2.517E-03	4.712E-03	PB-CAG-SD
ORC4	origin recognition complex, subunit 4 [Source:HGNC Symbol;Acc:8490]	2	148691732	148784147	798	0.073	1	1	2.518E-03	4.712E-03	PB-CAG-SD
PITPNB	phosphatidylinositol transfer protein, beta [Source:HGNC Symbol;Acc:9002]	22	28247657	28321122	608	0.073	1	1	2.520E-03	4.712E-03	PB-CAG-SD
CHRM5	cholinergic receptor, muscarinic 5 [Source:HGNC Symbol;Acc:1954]	15	34255921	34357291	704	0.073	1	1	2.521E-03	4.712E-03	PB-CAG-SD
XPR1	xenotropic and polytropic retrovirus receptor 1 [Source:HGNC Symbol;Acc:12827]	1	180596140	180859387	2181	0.264	2	2	2.524E-03	4.712E-03	PB-CAG-SD
SLK	STE20-like kinase [Source:HGNC Symbol;Acc:11088]	10	105721959	105788991	642	0.073	1	1	2.560E-03	4.771E-03	PB-CAG-SD
ZNF81	zinc finger protein 81 [Source:HGNC Symbol;Acc:13156]	X	47691301	47861960	1070	0.073	1	1	2.566E-03	4.775E-03	PB-CAG-SD
ARHGEF11	Rho guanine nucleotide exchange factor (GEF) 11 [Source:HGNC Symbol;Acc:14580]	1	156904632	157020162	609	0.074	1	1	2.591E-03	4.814E-03	PB-CAG-SD
PLXNA4	plexin A4 [Source:HGNC Symbol;Acc:9102]	7	131808091	132338447	2229	0.267	2	2	2.612E-03	4.845E-03	PB-CAG-SD
AKAP12	A kinase (PRKA) anchor protein 12 [Source:HGNC Symbol;Acc:370]	6	151556134	151679692	750	0.074	1	1	2.641E-03	4.891E-03	PB-CAG-SD
DPY19L4	dpy-19-like 4 (C. elegans) [Source:HGNC Symbol;Acc:27829]	8	95726931	95806064	606	0.075	1	1	2.656E-03	4.912E-03	PB-CAG-SD
JRKL	jerky homolog-like (mouse) [Source:HGNC Symbol;Acc:6200]	11	96118153	96240738	860	0.075	1	1	2.666E-03	4.924E-03	PB-CAG-SD
SEPT9	septin 9 [Source:HGNC Symbol;Acc:7323]	17	75271651	75496678	490	0.075	1	1	2.683E-03	4.937E-03	PB-CAG-SD
LRMP	lymphoid-restricted membrane protein [Source:HGNC Symbol;Acc:6690]	12	25168936	25261268	649	0.075	1	1	2.685E-03	4.937E-03	PB-CAG-SD
EGR2	early growth response 2 [Source:HGNC Symbol;Acc:3239]	10	64571756	64684660	658	0.075	1	1	2.686E-03	4.937E-03	PB-CAG-SD
RGS21	regulator of G-protein signaling 21 [Source:HGNC Symbol;Acc:26839]	1	192281122	192336415	625	0.076	1	1	2.725E-03	5.002E-03	PB-CAG-SD
LIN54	lin-54 homolog (C. elegans) [Source:HGNC Symbol;Acc:25397]	4	83831126	83939079	864	0.076	1	1	2.761E-03	5.061E-03	PB-CAG-SD
PCGF5	polycomb group ring finger 5 [Source:HGNC Symbol;Acc:28264]	10	92974908	93044088	669	0.076	1	1	2.774E-03	5.076E-03	PB-CAG-SD
LINC00687	long intergenic non-protein coding RNA 687 [Source:HGNC Symbol;Acc:16194]	20	11781111	11856363	534	0.077	1	1	2.797E-03	5.111E-03	PB-CAG-SD
PPP4R2	protein phosphatase 4, regulatory subunit 2 [Source:HGNC Symbol;Acc:18296]	3	73040936	73118350	644	0.077	1	1	2.805E-03	5.117E-03	PB-CAG-SD
PEX7	peroxisomal biogenesis factor 7 [Source:HGNC Symbol;Acc:8860]	6	137138717	137235075	776	0.077	1	1	2.822E-03	5.136E-03	PB-CAG-SD
INTU	inturned planar cell polarity protein [Source:HGNC Symbol;Acc:29239]	4	128539426	128637930	874	0.077	1	1	2.824E-03	5.136E-03	PB-CAG-SD
TNIP3	TNFAIP3 interacting protein 3 [Source:HGNC Symbol;Acc:19315]	4	122052563	122153621	875	0.077	1	1	2.830E-03	5.140E-03	PB-CAG-SD
ZNF451	zinc finger protein 451 [Source:HGNC Symbol;Acc:21091]	6	56946642	57035105	785	0.078	1	1	2.886E-03	5.234E-03	PB-CAG-SD
NSMAF	neutral sphingomyelinase (N-SMase) activation associated factor [Source:HGNC Symbol;Acc:8017]	8	59496063	59577403	634	0.078	1	1	2.901E-03	5.252E-03	PB-CAG-SD
OSTN	osteocrin [Source:HGNC Symbol;Acc:29961]	3	190912030	190983404	658	0.079	1	1	2.925E-03	5.288E-03	PB-CAG-SD
NFIB	nuclear factor I/B [Source:HGNC Symbol;Acc:7785]	9	14081842	14403982	2835	0.279	2	1	2.954E-03	5.332E-03	PB-CAG-SD
ARHGAP25	Rho GTPase activating protein 25 [Source:HGNC Symbol;Acc:28951]	2	68901733	69053965	867	0.079	1	1	2.960E-03	5.335E-03	PB-CAG-SD
PDCD6IP	programmed cell death 6 interacting protein [Source:HGNC Symbol;Acc:8766]	3	33834844	33911194	663	0.079	1	1	2.968E-03	5.342E-03	PB-CAG-SD
RAB27A	RAB27A, member RAS oncogene family [Source:HGNC Symbol;Acc:9766]	15	55495164	55616311	767	0.079	1	1	2.980E-03	5.355E-03	PB-CAG-SD
MIER1	mesoderm induction early response 1 homolog (Xenopus laevis) [Source:HGNC Symbol;Acc:29657]	1	67385578	67454302	659	0.080	1	1	3.021E-03	5.414E-03	PB-CAG-SD
CACUL1	CDK2-associated, cullin domain 1 [Source:HGNC Symbol;Acc:23727]	10	120433679	120519761	699	0.080	1	1	3.022E-03	5.414E-03	PB-CAG-SD
NGLY1	N-glycanase 1 [Source:HGNC Symbol;Acc:17646]	3	25760435	25836530	671	0.080	1	1	3.039E-03	5.436E-03	PB-CAG-SD
C7orf63	chromosome 7 open reading frame 63 [Source:HGNC Symbol;Acc:26107]	7	89869488	89940377	670	0.080	1	1	3.063E-03	5.465E-03	PB-CAG-SD
RIF1	RAP1 interacting factor homolog (yeast) [Source:HGNC Symbol;Acc:23207]	2	152261397	152364527	883	0.080	1	1	3.067E-03	5.465E-03	PB-CAG-SD
DEPDC5	DEP domain containing 5 [Source:HGNC Symbol;Acc:18423]	22	32144944	32303001	673	0.081	1	1	3.072E-03	5.465E-03	PB-CAG-SD
TMEM56	transmembrane protein 56 [Source:HGNC Symbol;Acc:26477]	1	95577894	95663163	665	0.081	1	1	3.075E-03	5.465E-03	PB-CAG-SD
SLC38A1	solute carrier family 38, member 1 [Source:HGNC Symbol;Acc:13447]	12	46576846	46668800	696	0.081	1	1	3.077E-03	5.465E-03	PB-CAG-SD
LUC7L2	LUC7-like 2 (S. cerevisiae) [Source:HGNC Symbol;Acc:21608]	7	139021106	139107345	673	0.081	1	1	3.089E-03	5.478E-03	PB-CAG-SD
NPAS2	neuronal PAS domain protein 2 [Source:HGNC Symbol;Acc:7895]	2	101431614	101613291	888	0.081	1	1	3.101E-03	5.490E-03	PB-CAG-SD
IMMP2L	IMP2 inner mitochondrial membrane peptidase-like (S. cerevisiae) [Source:HGNC Symbol;Acc:14598]	7	110303110	111207573	8019	0.962	4	1	3.111E-03	5.499E-03	PB-CAG-SD
WDR25	WD repeat domain 25 [Source:HGNC Symbol;Acc:21064]	14	100837755	100996640	741	0.081	1	1	3.119E-03	5.506E-03	PB-CAG-SD
C7orf55-LUC7L2	C7orf55-LUC7L2 readthrough [Source:HGNC Symbol;Acc:44671]	7	139020105	139108198	681	0.082	1	1	3.161E-03	5.572E-03	PB-CAG-SD
TRPC4AP	transient receptor potential cation channel, subfamily C, member 4 associated protein [Source:HGNC Symbol;Acc:16181]	20	33590207	33685674	569	0.082	1	1	3.165E-03	5.572E-03	PB-CAG-SD
RDX	radixin [Source:HGNC Symbol;Acc:9944]	11	110045605	110172447	942	0.082	1	1	3.184E-03	5.594E-03	PB-CAG-SD
SLC30A9	solute carrier family 30 (zinc transporter), member 9 [Source:HGNC Symbol;Acc:1329]	4	41987489	42089551	930	0.082	1	1	3.187E-03	5.594E-03	PB-CAG-SD
EPRS	glutamyl-prolyl-tRNA synthetase [Source:HGNC Symbol;Acc:3418]	1	220141943	220225000	681	0.082	1	1	3.221E-03	5.643E-03	PB-CAG-SD
REPS1	RALBP1 associated Eps domain containing 1 [Source:HGNC Symbol;Acc:15578]	6	139224630	139314398	831	0.083	1	1	3.225E-03	5.643E-03	PB-CAG-SD
PARVA	parvin, alpha [Source:HGNC Symbol;Acc:14652]	11	12393732	12552348	949	0.083	1	1	3.230E-03	5.645E-03	PB-CAG-SD
GSK3B	glycogen synthase kinase 3 beta [Source:HGNC Symbol;Acc:4617]	3	119540170	119818264	2430	0.290	2	1	3.273E-03	5.711E-03	PB-CAG-SD
IL23R	interleukin 23 receptor [Source:HGNC Symbol;Acc:19100]	1	67627083	67725662	689	0.083	1	1	3.295E-03	5.741E-03	PB-CAG-SD
CREM	cAMP responsive element modulator [Source:HGNC Symbol;Acc:2352]	10	35410719	35501886	732	0.084	1	1	3.305E-03	5.751E-03	PB-CAG-SD
EFCAB4B	EF-hand calcium binding domain 4B [Source:HGNC Symbol;Acc:28657]	12	3715799	3878985	723	0.084	1	1	3.314E-03	5.757E-03	PB-CAG-SD
NEK11	NIMA-related kinase 11 [Source:HGNC Symbol;Acc:18593]	3	130740694	131069309	2449	0.292	2	1	3.345E-03	5.797E-03	PB-CAG-SD
COL4A2	collagen, type IV, alpha 2 [Source:HGNC Symbol;Acc:2203]	13	110953159	111165374	975	0.084	1	1	3.349E-03	5.797E-03	PB-CAG-SD
SLC22A15	solute carrier family 22, member 15 [Source:HGNC Symbol;Acc:20301]	1	116514119	116612675	695	0.084	1	1	3.351E-03	5.797E-03	PB-CAG-SD
LRIG1	leucine-rich repeats and immunoglobulin-like domains 1 [Source:HGNC Symbol;Acc:17360]	3	66429221	66556687	711	0.085	1	1	3.401E-03	5.875E-03	PB-CAG-SD
ARHGEF4	Rho guanine nucleotide exchange factor (GEF) 4 [Source:HGNC Symbol;Acc:684]	2	131589489	131804836	933	0.085	1	1	3.414E-03	5.889E-03	PB-CAG-SD
DIRC2	disrupted in renal carcinoma 2 [Source:HGNC Symbol;Acc:16628]	3	122508642	122599986	716	0.085	1	1	3.448E-03	5.938E-03	PB-CAG-SD
GPR176	G protein-coupled receptor 176 [Source:HGNC Symbol;Acc:32370]	15	40091233	40218093	834	0.086	1	1	3.507E-03	6.032E-03	PB-CAG-SD
MSH2	mutS homolog 2, colon cancer, nonpolyposis type 1 (E. coli) [Source:HGNC Symbol;Acc:7325]	2	47625108	47789450	956	0.087	1	1	3.579E-03	6.148E-03	PB-CAG-SD
ANAPC1	anaphase promoting complex subunit 1 [Source:HGNC Symbol;Acc:19988]	2	112523848	112647267	957	0.087	1	1	3.587E-03	6.151E-03	PB-CAG-SD
PLAGL1	pleiomorphic adenoma gene-like 1 [Source:HGNC Symbol;Acc:9046]	6	144261437	144390735	880	0.087	1	1	3.605E-03	6.173E-03	PB-CAG-SD
SORL1	sortilin-related receptor, L(DLR class) A repeats containing [Source:HGNC Symbol;Acc:11185]	11	121317912	121504402	1012	0.088	1	1	3.660E-03	6.258E-03	PB-CAG-SD
COX10-AS1	COX10 antisense RNA 1 [Source:HGNC Symbol;Acc:38873]	17	13659795	13977812	1970	0.302	2	1	3.664E-03	6.258E-03	PB-CAG-SD
PPA2	pyrophosphatase (inorganic) 2 [Source:HGNC Symbol;Acc:28883]	4	106290234	106400238	1000	0.088	1	1	3.670E-03	6.259E-03	PB-CAG-SD
TBC1D15	TBC1 domain family, member 15 [Source:HGNC Symbol;Acc:25694]	12	72228487	72320629	763	0.088	1	1	3.679E-03	6.266E-03	PB-CAG-SD
CHURC1-FNTB	CHURC1-FNTB readthrough [Source:HGNC Symbol;Acc:42960]	14	65376203	65529368	808	0.088	1	1	3.690E-03	6.275E-03	PB-CAG-SD
C12orf56	chromosome 12 open reading frame 56 [Source:HGNC Symbol;Acc:26967]	12	64660217	64789972	766	0.089	1	1	3.707E-03	6.294E-03	PB-CAG-SD
CCDC150	coiled-coil domain containing 150 [Source:HGNC Symbol;Acc:26834]	2	197499278	197628214	974	0.089	1	1	3.711E-03	6.294E-03	PB-CAG-SD
MIR31HG	MIR31 host gene (non-protein coding) [Source:HGNC Symbol;Acc:37187]	9	21455641	21564668	903	0.089	1	1	3.734E-03	6.323E-03	PB-CAG-SD
PACSIN2	protein kinase C and casein kinase substrate in neurons 2 [Source:HGNC Symbol;Acc:8571]	22	43231418	43416151	745	0.089	1	1	3.743E-03	6.329E-03	PB-CAG-SD
STAT4	signal transducer and activator of transcription 4 [Source:HGNC Symbol;Acc:11365]	2	191894302	192021322	984	0.090	1	1	3.786E-03	6.393E-03	PB-CAG-SD
ADAMTSL1	ADAMTS-like 1 [Source:HGNC Symbol;Acc:14632]	9	18468892	18910948	3106	0.306	2	1	3.809E-03	6.423E-03	PB-CAG-SD
ADIPOR2	adiponectin receptor 2 [Source:HGNC Symbol;Acc:24041]	12	1792740	1897844	778	0.090	1	1	3.821E-03	6.429E-03	PB-CAG-SD
SGCE	sarcoglycan, epsilon [Source:HGNC Symbol;Acc:10808]	7	94214542	94290521	751	0.090	1	1	3.823E-03	6.429E-03	PB-CAG-SD
C2CD3	C2 calcium-dependent domain containing 3 [Source:HGNC Symbol;Acc:24564]	11	73723763	73887255	1039	0.090	1	1	3.852E-03	6.468E-03	PB-CAG-SD
SCAF11	SR-related CTD-associated factor 11 [Source:HGNC Symbol;Acc:10784]	12	46312914	46390903	783	0.091	1	1	3.869E-03	6.488E-03	PB-CAG-SD
XXYLT1	xyloside xylosyltransferase 1 [Source:HGNC Symbol;Acc:26639]	3	194789008	194996896	762	0.091	1	1	3.891E-03	6.515E-03	PB-CAG-SD
FNIP2	folliculin interacting protein 2 [Source:HGNC Symbol;Acc:29280]	4	159685290	159829201	1037	0.091	1	1	3.938E-03	6.583E-03	PB-CAG-SD
RCOR1	REST corepressor 1 [Source:HGNC Symbol;Acc:17441]	14	103053998	103196913	836	0.092	1	1	3.942E-03	6.583E-03	PB-CAG-SD
GTDC1	glycosyltransferase-like domain containing 1 [Source:HGNC Symbol;Acc:20887]	2	144703321	145095135	3406	0.310	2	1	3.953E-03	6.591E-03	PB-CAG-SD
KIAA1217	KIAA1217 [Source:HGNC Symbol;Acc:25428]	10	23978675	24836772	5512	0.629	3	1	3.976E-03	6.612E-03	PB-CAG-SD
AGL	amylo-alpha-1, 6-glucosidase, 4-alpha-glucanotransferase [Source:HGNC Symbol;Acc:321]	1	100310640	100389579	759	0.092	1	1	3.976E-03	6.612E-03	PB-CAG-SD
SNX9	sorting nexin 9 [Source:HGNC Symbol;Acc:14973]	6	158239296	158366109	928	0.092	1	1	3.996E-03	6.629E-03	PB-CAG-SD
NFKB1	nuclear factor of kappa light polypeptide gene enhancer in B-cells 1 [Source:HGNC Symbol;Acc:7794]	4	103417486	103538459	1045	0.092	1	1	3.997E-03	6.629E-03	PB-CAG-SD
SPTLC2	serine palmitoyltransferase, long chain base subunit 2 [Source:HGNC Symbol;Acc:11278]	14	77972340	78088116	845	0.093	1	1	4.025E-03	6.666E-03	PB-CAG-SD
SGK1	serum/glucocorticoid regulated kinase 1 [Source:HGNC Symbol;Acc:10810]	6	134490384	134644250	939	0.093	1	1	4.088E-03	6.761E-03	PB-CAG-SD
FOXO3	forkhead box O3 [Source:HGNC Symbol;Acc:3821]	6	108876038	109005977	940	0.093	1	1	4.097E-03	6.766E-03	PB-CAG-SD
TBL1X	transducin (beta)-like 1X-linked [Source:HGNC Symbol;Acc:11585]	X	9426335	9687780	1365	0.094	1	1	4.121E-03	6.787E-03	PB-CAG-SD
GLS	glutaminase [Source:HGNC Symbol;Acc:4331]	2	191740553	191830278	1028	0.094	1	1	4.121E-03	6.787E-03	PB-CAG-SD

table S2. Genes with significant transposon integrations in RKO *BRAF*$^{wt/-/-}$ cells after selection in low glucose medium

Gene ID	Description	Chrom	Start	End	# TTAA sites	Expected	Observed	Libraries	*P*	FDR	Transposon
PPAP2A	phosphatidic acid phosphatase type 2A [Source:HGNC Symbol;Acc:9228]	5	54720682	54835878	884	0.094	1	1	4.143E-03	6.815E-03	PB-CAG-SD
LEMD3	LEM domain containing 3 [Source:HGNC Symbol;Acc:28887]	12	65558351	65642107	813	0.094	1	1	4.161E-03	6.835E-03	PB-CAG-SD
GSAP	gamma-secretase activating protein [Source:HGNC Symbol;Acc:28042]	7	76940068	77050717	786	0.094	1	1	4.176E-03	6.850E-03	PB-CAG-SD
GOLIM4	golgi integral membrane protein 4 [Source:HGNC Symbol;Acc:15448]	3	167726465	167818763	791	0.094	1	1	4.183E-03	6.852E-03	PB-CAG-SD
PPP1R42	protein phosphatase 1, regulatory subunit 42 [Source:HGNC Symbol;Acc:33732]	8	67876334	67973839	767	0.095	1	1	4.199E-03	6.870E-03	PB-CAG-SD
SLC9C2	solute carrier family 9, member C2 (putative) [Source:HGNC Symbol;Acc:28664]	1	173469603	173577233	784	0.095	1	1	4.234E-03	6.916E-03	PB-CAG-SD
PDZD8	PDZ domain containing 8 [Source:HGNC Symbol;Acc:26974]	10	119040000	119139978	833	0.095	1	1	4.248E-03	6.930E-03	PB-CAG-SD
RHOBTB3	Rho-related BTB domain containing 3 [Source:HGNC Symbol;Acc:18757]	5	95044226	95160087	897	0.095	1	1	4.262E-03	6.939E-03	PB-CAG-SD
ARL13B	ADP-ribosylation factor-like 13B [Source:HGNC Symbol;Acc:25419]	3	93693983	93774512	799	0.095	1	1	4.265E-03	6.939E-03	PB-CAG-SD
ECT2	epithelial cell transforming sequence 2 oncogene [Source:HGNC Symbol;Acc:3155]	3	172463472	172539264	800	0.095	1	1	4.275E-03	6.947E-03	PB-CAG-SD
GABRB1	gamma-aminobutyric acid (GABA) A receptor, beta 1 [Source:HGNC Symbol;Acc:4081]	4	46990740	47428461	3621	0.319	2	2	4.284E-03	6.951E-03	PB-CAG-SD
CNST	consortin, connexin sorting protein [Source:HGNC Symbol;Acc:26486]	1	246724746	246831886	792	0.096	1	1	4.318E-03	6.987E-03	PB-CAG-SD
ZNF670	zinc finger protein 670 [Source:HGNC Symbol;Acc:28167]	1	247108849	247247113	792	0.096	1	1	4.318E-03	6.987E-03	PB-CAG-SD
DCAF8L2	DDB1 and CUL4 associated factor 8-like 2 [Source:HGNC Symbol;Acc:31811]	X	27603499	27766908	1411	0.097	1	1	4.394E-03	7.101E-03	PB-CAG-SD
ZMYND8	zinc finger, MYND-type containing 8 [Source:HGNC Symbol;Acc:9397]	20	45837859	45990567	675	0.097	1	1	4.410E-03	7.118E-03	PB-CAG-SD
CELF4	CUGBP, Elav-like family member 4 [Source:HGNC Symbol;Acc:14015]	18	34823010	35151000	979	0.097	1	1	4.419E-03	7.119E-03	PB-CAG-SD
KDM5A	lysine (K)-specific demethylase 5A [Source:HGNC Symbol;Acc:9886]	12	389295	503620	839	0.097	1	1	4.423E-03	7.119E-03	PB-CAG-SD
RNF216	ring finger protein 216 [Source:HGNC Symbol;Acc:21698]	7	5659678	5826370	811	0.097	1	1	4.437E-03	7.133E-03	PB-CAG-SD
ADAMTS6	ADAM metallopeptidase with thrombospondin type 1 motif, 6 [Source:HGNC Symbol;Acc:222]	5	64444563	64782747	3048	0.324	2	1	4.447E-03	7.140E-03	PB-CAG-SD
TACC1	transforming, acidic coiled-coil containing protein 1 [Source:HGNC Symbol;Acc:11522]	8	38580704	38710546	791	0.098	1	1	4.458E-03	7.146E-03	PB-CAG-SD
XPO4	exportin 4 [Source:HGNC Symbol;Acc:17796]	13	21351469	21482187	1134	0.098	1	1	4.490E-03	7.189E-03	PB-CAG-SD
ORC5	origin recognition complex, subunit 5 [Source:HGNC Symbol;Acc:8491]	7	103766788	103853495	817	0.098	1	1	4.501E-03	7.197E-03	PB-CAG-SD
NUF2	NUF2, NDC80 kinetochore complex component [Source:HGNC Symbol;Acc:14621]	1	163231366	163325554	812	0.098	1	1	4.531E-03	7.219E-03	PB-CAG-SD
EHMT1	euchromatic histone-lysine N-methyltransferase 1 [Source:HGNC Symbol;Acc:24650]	9	140508444	140764468	998	0.098	1	1	4.533E-03	7.219E-03	PB-CAG-SD
DYX1C1-CCPG1	DYX1C1-CCPG1 readthrough (NMD candidate) [Source:HGNC Symbol;Acc:43019]	15	55647446	55795558	952	0.098	1	1	4.533E-03	7.219E-03	PB-CAG-SD
GARNL3	GTPase activating Rap/RanGAP domain-like 3 [Source:HGNC Symbol;Acc:25425]	9	129981544	130155939	1004	0.099	1	1	4.586E-03	7.294E-03	PB-CAG-SD
SESN1	sestrin 1 [Source:HGNC Symbol;Acc:21595]	6	109307640	109421022	1000	0.099	1	1	4.618E-03	7.335E-03	PB-CAG-SD
HSF2BP	heat shock transcription factor 2 binding protein [Source:HGNC Symbol;Acc:5226]	21	44949072	45084374	686	0.100	1	1	4.639E-03	7.360E-03	PB-CAG-SD
FKBP5	FK506 binding protein 5 [Source:HGNC Symbol;Acc:3721]	6	35541362	35701360	1004	0.100	1	1	4.654E-03	7.373E-03	PB-CAG-SD
CEP192	centrosomal protein 192kDa [Source:HGNC Symbol;Acc:25515]	18	12986361	13125051	1007	0.100	1	1	4.667E-03	7.383E-03	PB-CAG-SD
CRY1	cryptochrome 1 (photolyase-like) [Source:HGNC Symbol;Acc:2384]	12	107385142	107492607	867	0.100	1	1	4.713E-03	7.445E-03	PB-CAG-SD
KSR1	kinase suppressor of ras 1 [Source:HGNC Symbol;Acc:6465]	17	25778670	25953461	656	0.101	1	1	4.728E-03	7.445E-03	PB-CAG-SD
GRIN2B	glutamate receptor, ionotropic, N-methyl D-aspartate 2B [Source:HGNC Symbol;Acc:4586]	12	13714144	14138053	2860	0.331	2	1	4.730E-03	7.445E-03	PB-CAG-SD
RFC3	replication factor C (activator 1) 3, 38kDa [Source:HGNC Symbol;Acc:9971]	13	34387186	34540695	1165	0.101	1	1	4.730E-03	7.445E-03	PB-CAG-SD
ANKRD13C	ankyrin repeat domain 13C [Source:HGNC Symbol;Acc:25374]	1	70726271	70825417	832	0.101	1	1	4.750E-03	7.466E-03	PB-CAG-SD
SHROOM4	shroom family member 4 [Source:HGNC Symbol;Acc:29215]	X	50334647	50562302	1472	0.101	1	1	4.769E-03	7.482E-03	PB-CAG-SD
ADCY9	adenylate cyclase 9 [Source:HGNC Symbol;Acc:240]	16	4003388	4171186	774	0.101	1	1	4.774E-03	7.482E-03	PB-CAG-SD
RGS17	regulator of G-protein signaling 17 [Source:HGNC Symbol;Acc:14088]	6	153325594	153457384	1018	0.101	1	1	4.780E-03	7.482E-03	PB-CAG-SD
CGNL1	cingulin-like 1 [Source:HGNC Symbol;Acc:25931]	15	57663165	57842925	979	0.101	1	1	4.785E-03	7.482E-03	PB-CAG-SD
MSANTD3-TMEF	MSANTD3-TMEFF1 readthrough [Source:HGNC Symbol;Acc:38838]	9	103199553	103339918	1030	0.102	1	1	4.818E-03	7.514E-03	PB-CAG-SD
TMEFF1	transmembrane protein with EGF-like and two follistatin-like domains 1 [Source:HGNC Symbol;Acc:11866]	9	103199560	103339918	1030	0.102	1	1	4.818E-03	7.514E-03	PB-CAG-SD
FER1L6-AS2	FER1L6 antisense RNA 2 [Source:HGNC Symbol;Acc:26534]	8	125058314	125188763	824	0.102	1	1	4.824E-03	7.514E-03	PB-CAG-SD
TNRC6A	trinucleotide repeat containing 6A [Source:HGNC Symbol;Acc:11969]	16	24736016	24838953	780	0.102	1	1	4.845E-03	7.537E-03	PB-CAG-SD
CPPED1	calcineurin-like phosphoesterase domain containing 1 [Source:HGNC Symbol;Acc:25632]	16	12756919	12902874	781	0.102	1	1	4.857E-03	7.546E-03	PB-CAG-SD
ROR1	receptor tyrosine kinase-like orphan receptor 1 [Source:HGNC Symbol;Acc:10256]	1	64234693	64647181	2788	0.338	2	2	4.994E-03	7.748E-03	PB-CAG-SD
PIK3R3	phosphoinositide-3-kinase, regulatory subunit 3 (gamma) [Source:HGNC Symbol;Acc:8981]	1	46505812	46647160	856	0.104	1	1	5.018E-03	7.776E-03	PB-CAG-SD
SCAMP1	secretory carrier membrane protein 1 [Source:HGNC Symbol;Acc:10563]	5	77651339	77776562	983	0.104	1	1	5.088E-03	7.873E-03	PB-CAG-SD
MYOF	myoferlin [Source:HGNC Symbol;Acc:3656]	10	95066186	95247074	920	0.105	1	1	5.148E-03	7.956E-03	PB-CAG-SD
ARHGAP11B	Rho GTPase activating protein 11B [Source:HGNC Symbol;Acc:15782]	15	30911697	31065196	1018	0.105	1	1	5.160E-03	7.965E-03	PB-CAG-SD
MTMR7	myotubularin related protein 7 [Source:HGNC Symbol;Acc:7454]	8	17155539	17276037	860	0.106	1	1	5.240E-03	8.077E-03	PB-CAG-SD
C16orf62	chromosome 16 open reading frame 62 [Source:HGNC Symbol;Acc:24641]	16	19561562	19718115	814	0.106	1	1	5.262E-03	8.101E-03	PB-CAG-SD
RTN4	reticulon 4 [Source:HGNC Symbol;Acc:14085]	2	55199325	55344757	1181	0.108	1	1	5.389E-03	8.286E-03	PB-CAG-SD
ABI1	abl-interactor 1 [Source:HGNC Symbol;Acc:11320]	10	27035522	27155016	946	0.108	1	1	5.432E-03	8.337E-03	PB-CAG-SD
FRMD6-AS2	FRMD6 antisense RNA 2 [Source:HGNC Symbol;Acc:43637]	14	51921230	52071670	987	0.108	1	1	5.435E-03	8.337E-03	PB-CAG-SD
LRRC8D	leucine rich repeat containing 8 family, member D [Source:HGNC Symbol;Acc:16992]	1	90281573	90402170	894	0.108	1	1	5.457E-03	8.358E-03	PB-CAG-SD
NUP93	nucleoporin 93kDa [Source:HGNC Symbol;Acc:28958]	16	56759017	56878797	830	0.108	1	1	5.463E-03	8.358E-03	PB-CAG-SD
PCDH7	protocadherin 7 [Source:HGNC Symbol;Acc:8659]	4	30717037	31148422	3978	0.351	2	1	5.550E-03	8.480E-03	PB-CAG-SD
BTAF1	BTAF1 RNA polymerase II, B-TFIID transcription factor-associated, 170kDa [Source:HGNC Symbol;Acc:17307]	10	93678526	93790082	965	0.110	1	1	5.644E-03	8.613E-03	PB-CAG-SD
ATM	ataxia telangiectasia mutated [Source:HGNC Symbol;Acc:795]	11	108088211	108239829	1268	0.110	1	1	5.662E-03	8.620E-03	PB-CAG-SD
CHM	choroideremia (Rab escort protein 1) [Source:HGNC Symbol;Acc:1940]	X	85116185	85307566	1610	0.110	1	1	5.669E-03	8.620E-03	PB-CAG-SD
BAZ1A	bromodomain adjacent to zinc finger domain, 1A [Source:HGNC Symbol;Acc:960]	14	35221937	35349853	1009	0.110	1	1	5.671E-03	8.620E-03	PB-CAG-SD
TNIK	TRAF2 and NCK interacting kinase [Source:HGNC Symbol;Acc:30765]	3	170779128	171183197	2966	0.354	2	1	5.678E-03	8.620E-03	PB-CAG-SD
XKR9	XK, Kell blood group complex subunit-related family, member 9 [Source:HGNC Symbol;Acc:20937]	8	71576600	71702606	898	0.111	1	1	5.695E-03	8.636E-03	PB-CAG-SD
EFTUD1	elongation factor Tu GTP binding domain containing 1 [Source:HGNC Symbol;Acc:25789]	15	82422571	82560104	1079	0.111	1	1	5.773E-03	8.743E-03	PB-CAG-SD
DDR2	discoidin domain receptor tyrosine kinase 2 [Source:HGNC Symbol;Acc:2731]	1	162596163	162750237	923	0.112	1	1	5.803E-03	8.778E-03	PB-CAG-SD
MYBPC1	myosin binding protein C, slow type [Source:HGNC Symbol;Acc:7549]	12	101957131	102079796	974	0.113	1	1	5.900E-03	8.908E-03	PB-CAG-SD
PKHD1	polycystic kidney and hepatic disease 1 (autosomal recessive) [Source:HGNC Symbol;Acc:9016]	6	51480098	51957423	3614	0.359	2	2	5.904E-03	8.908E-03	PB-CAG-SD
TRIM2	tripartite motif containing 2 [Source:HGNC Symbol;Acc:15974]	4	154068494	154260472	1280	0.113	1	1	5.916E-03	8.914E-03	PB-CAG-SD
KMT2E	lysine (K)-specific methyltransferase 2E [Source:HGNC Symbol;Acc:18541]	7	104649626	104754808	945	0.113	1	1	5.961E-03	8.971E-03	PB-CAG-SD
KCNQ1	potassium voltage-gated channel, KQT-like subfamily, member 1 [Source:HGNC Symbol;Acc:6294]	11	2460914	2870339	1322	0.115	1	1	6.135E-03	9.222E-03	PB-CAG-SD
CLTC	clathrin, heavy chain (Hc) [Source:HGNC Symbol;Acc:2092]	17	57692219	57773671	753	0.115	1	1	6.168E-03	9.253E-03	PB-CAG-SD
STIM1	stromal interaction molecule 1 [Source:HGNC Symbol;Acc:11386]	11	3870757	4114439	1326	0.115	1	1	6.171E-03	9.253E-03	PB-CAG-SD
FBXW11	F-box and WD repeat domain containing 11 [Source:HGNC Symbol;Acc:13607]	5	171288553	171438877	1093	0.116	1	1	6.242E-03	9.340E-03	PB-CAG-SD
ANK1	ankyrin 1, erythrocytic [Source:HGNC Symbol;Acc:492]	8	41510739	41759280	942	0.116	1	1	6.245E-03	9.340E-03	PB-CAG-SD
SNRK	SNF related kinase [Source:HGNC Symbol;Acc:30598]	3	43323004	43466256	977	0.117	1	1	6.288E-03	9.382E-03	PB-CAG-SD
FBXL2	F-box and leucine-rich repeat protein 2 [Source:HGNC Symbol;Acc:13598]	3	33313517	33445154	977	0.117	1	1	6.288E-03	9.382E-03	PB-CAG-SD
DENND4C	DENN/MADD domain containing 4C [Source:HGNC Symbol;Acc:26079]	9	19225433	19374139	1184	0.117	1	1	6.303E-03	9.392E-03	PB-CAG-SD
ZNF827	zinc finger protein 827 [Source:HGNC Symbol;Acc:27193]	4	146678779	146864787	1326	0.117	1	1	6.331E-03	9.423E-03	PB-CAG-SD
MDN1	MDN1, midasin homolog (yeast) [Source:HGNC Symbol;Acc:18302]	6	90352218	90534442	1179	0.117	1	1	6.344E-03	9.430E-03	PB-CAG-SD
SPECC1L	sperm antigen with calponin homology and coiled-coil domains 1-like [Source:HGNC Symbol;Acc:29022]	22	24661786	24813708	982	0.117	1	1	6.382E-03	9.475E-03	PB-CAG-SD
NDUFS4	NADH dehydrogenase (ubiquinone) Fe-S protein 4, 18kDa (NADH-coenzyme Q reductase) [Source:HGNC Symbol;Acc:7711]	5	52851463	52979168	1111	0.118	1	1	6.441E-03	9.550E-03	PB-CAG-SD
STAG1	stromal antigen 1 [Source:HGNC Symbol;Acc:11354]	3	136055077	136476220	3111	0.371	2	1	6.469E-03	9.580E-03	PB-CAG-SD
SEMA5A	sema domain, seven thrombospondin repeats (type 1 and type 1-like), transmembrane domain (TM) and short cytoplasmic dor	5	9035138	9551187	3500	0.372	2	2	6.500E-03	9.614E-03	PB-CAG-SD
TNPO1	transportin 1 [Source:HGNC Symbol;Acc:6401]	5	72107139	72212560	1118	0.119	1	1	6.519E-03	9.631E-03	PB-CAG-SD
BMPR2	bone morphogenetic protein receptor, type II (serine/threonine kinase) [Source:HGNC Symbol;Acc:1078]	2	203236659	203432474	1313	0.120	1	1	6.608E-03	9.750E-03	PB-CAG-SD
TPD52L1	tumor protein D52-like 1 [Source:HGNC Symbol;Acc:12006]	6	125435195	125585553	1206	0.120	1	1	6.627E-03	9.762E-03	PB-CAG-SD
DEPTOR	DEP domain containing MTOR-interacting protein [Source:HGNC Symbol;Acc:22953]	8	120880957	121063152	972	0.120	1	1	6.632E-03	9.762E-03	PB-CAG-SD
FOXP2	forkhead box P2 [Source:HGNC Symbol;Acc:13875]	7	113721382	114333827	6086	0.730	3	1	6.652E-03	9.779E-03	PB-CAG-SD
SLC24A3	solute carrier family 24 (sodium/potassium/calcium exchanger), member 3 [Source:HGNC Symbol;Acc:10977]	20	19188290	19703581	2611	0.375	2	1	6.660E-03	9.779E-03	PB-CAG-SD
ZNF568	zinc finger protein 568 [Source:HGNC Symbol;Acc:25392]	19	37402231	37489602	552	0.120	1	1	6.676E-03	9.790E-03	PB-CAG-SD
ERCC6L2	excision repair cross-complementing rodent repair deficiency, complementation group 6-like 2 [Source:HGNC Symbol;Acc:2692	9	98632983	98776842	1227	0.121	1	1	6.751E-03	9.888E-03	PB-CAG-SD
RAI14	retinoic acid induced 14 [Source:HGNC Symbol;Acc:14873]	5	34651342	34832732	1142	0.121	1	1	6.790E-03	9.932E-03	PB-CAG-SD
PRKAR1A	protein kinase, cAMP-dependent, regulatory, type I, alpha [Source:HGNC Symbol;Acc:9388]	17	66404764	66547460	792	0.121	1	1	6.797E-03	9.932E-03	PB-CAG-SD
RPS11P6	ribosomal protein S11 pseudogene 6 [Source:HGNC Symbol;Acc:36693]	12	64611117	64790845	1052	0.122	1	1	6.841E-03	9.984E-03	PB-CAG-SD
SV2B	synaptic vesicle glycoprotein 2B [Source:HGNC Symbol;Acc:16874]	15	91638180	91844539	1189	0.123	1	1	6.957E-03	1.014E-02	PB-CAG-SD
C5orf42	chromosome 5 open reading frame 42 [Source:HGNC Symbol;Acc:25801]	5	37106330	37254530	1160	0.123	1	1	6.997E-03	1.019E-02	PB-CAG-SD
RALGAPB	Ral GTPase activating protein, beta subunit (non-catalytic) [Source:HGNC Symbol;Acc:29221]	20	37096459	37207504	859	0.123	1	1	7.018E-03	1.021E-02	PB-CAG-SD
KIAA1468	KIAA1468 [Source:HGNC Symbol;Acc:29289]	18	59849491	59974355	1248	0.124	1	1	7.055E-03	1.025E-02	PB-CAG-SD
CREBBP	CREB binding protein [Source:HGNC Symbol;Acc:2348]	16	3775055	3935727	949	0.124	1	1	7.069E-03	1.025E-02	PB-CAG-SD
C2CD5	C2 calcium-dependent domain containing 5 [Source:HGNC Symbol;Acc:29062]	12	22601517	22702480	1073	0.124	1	1	7.106E-03	1.030E-02	PB-CAG-SD
ZHX3	zinc fingers and homeoboxes 3 [Source:HGNC Symbol;Acc:15935]	20	39807088	39951312	866	0.124	1	1	7.128E-03	1.032E-02	PB-CAG-SD
CHD2	chromodomain helicase DNA binding protein 2 [Source:HGNC Symbol;Acc:1917]	15	93420937	93571237	1207	0.125	1	1	7.161E-03	1.035E-02	PB-CAG-SD
MLLT4	myeloid/lymphoid or mixed-lineage leukemia (trithorax homolog, Drosophila); translocated to, 4 [Source:HGNC Symbol;Acc:713	6	168222602	168372703	1259	0.125	1	1	7.197E-03	1.039E-02	PB-CAG-SD
SEPT7	septin 7 [Source:HGNC Symbol;Acc:1717]	7	35835542	35944917	1045	0.125	1	1	7.232E-03	1.043E-02	PB-CAG-SD
PAWR	PRKC, apoptosis, WT1, regulator [Source:HGNC Symbol;Acc:8614]	12	79968759	80089877	1085	0.126	1	1	7.259E-03	1.045E-02	PB-CAG-SD
GLG1	golgi glycoprotein 1 [Source:HGNC Symbol;Acc:4316]	16	74485856	74646012	964	0.126	1	1	7.285E-03	1.047E-02	PB-CAG-SD
WHSC1L1	Wolf-Hirschhorn syndrome candidate 1-like 1 [Source:HGNC Symbol;Acc:12767]	8	38127215	38244790	1021	0.126	1	1	7.289E-03	1.047E-02	PB-CAG-SD
SETD2	SET domain containing 2 [Source:HGNC Symbol;Acc:18420]	3	47057919	47210457	1057	0.126	1	1	7.314E-03	1.050E-02	PB-CAG-SD
ASCC3	activating signal cointegrator 1 complex subunit 3 [Source:HGNC Symbol;Acc:18697]	6	100956070	101334248	3937	0.391	2	1	7.455E-03	1.069E-02	PB-CAG-SD
C1orf141	chromosome 1 open reading frame 141 [Source:HGNC Symbol;Acc:32044]	1	67557848	67702536	1053	0.128	1	1	7.475E-03	1.070E-02	PB-CAG-SD
VAT1L	vesicle amine transport protein 1 homolog (T. californica)-like [Source:HGNC Symbol;Acc:29315]	16	77817427	78014004	980	0.128	1	1	7.518E-03	1.075E-02	PB-CAG-SD
KANSL1L	KAT8 regulatory NSL complex subunit 1-like [Source:HGNC Symbol;Acc:26310]	2	210886147	211041107	1415	0.129	1	1	7.628E-03	1.089E-02	PB-CAG-SD
ENPEP	glutamyl aminopeptidase (aminopeptidase A) [Source:HGNC Symbol;Acc:3355]	4	111281889	111486441	1467	0.129	1	1	7.686E-03	1.096E-02	PB-CAG-SD
TMEM163	transmembrane protein 163 [Source:HGNC Symbol;Acc:25380]	2	135213330	135481570	1426	0.130	1	1	7.742E-03	1.103E-02	PB-CAG-SD
HIPK2	homeodomain interacting protein kinase 2 [Source:HGNC Symbol;Acc:14402]	7	139246316	139482577	1087	0.130	1	1	7.799E-03	1.110E-02	PB-CAG-SD
LRCH1	leucine-rich repeats and calponin homology (CH) domain containing 1 [Source:HGNC Symbol;Acc:20309]	13	47122303	47327175	1513	0.131	1	1	7.822E-03	1.112E-02	PB-CAG-SD
GRB10	growth factor receptor-bound protein 10 [Source:HGNC Symbol;Acc:4564]	7	50657760	50866159	1098	0.132	1	1	7.951E-03	1.129E-02	PB-CAG-SD
AVEN	apoptosis, caspase activation inhibitor [Source:HGNC Symbol;Acc:13509]	15	34158428	34336377	1276	0.132	1	1	7.965E-03	1.130E-02	PB-CAG-SD
SLC38A9	solute carrier family 38, member 9 [Source:HGNC Symbol;Acc:26907]	5	54921673	55074022	1245	0.132	1	1	8.013E-03	1.134E-02	PB-CAG-SD
KCTD1	potassium channel tetramerization domain containing 1 [Source:HGNC Symbol;Acc:18249]	18	24034874	24242365	1334	0.132	1	1	8.016E-03	1.134E-02	PB-CAG-SD
APC	adenomatous polyposis coli [Source:HGNC Symbol;Acc:583]	5	112038195	112181936	1246	0.132	1	1	8.025E-03	1.134E-02	PB-CAG-SD
RNF220	ring finger protein 220 [Source:HGNC Symbol;Acc:25552]	1	44865866	45117396	1097	0.133	1	1	8.084E-03	1.141E-02	PB-CAG-SD
ARHGAP12	Rho GTPase activating protein 12 [Source:HGNC Symbol;Acc:16348]	10	32094365	32222742	1164	0.133	1	1	8.090E-03	1.141E-02	PB-CAG-SD
PPP1R9A	protein phosphatase 1, regulatory subunit 9A [Source:HGNC Symbol;Acc:14946]	7	94531514	94925727	3384	0.406	2	1	8.251E-03	1.162E-02	PB-CAG-SD
FMN2	formin 2 [Source:HGNC Symbol;Acc:14074]	1	240172648	240638489	3359	0.407	2	1	8.300E-03	1.167E-02	PB-CAG-SD
ARHGAP21	Rho GTPase activating protein 21 [Source:HGNC Symbol;Acc:23725]	10	24872538	25017597	1181	0.135	1	1	8.318E-03	1.169E-02	PB-CAG-SD
KIF1B	kinesin family member 1B [Source:HGNC Symbol;Acc:16636]	1	10265863	10441661	1117	0.135	1	1	8.368E-03	1.174E-02	PB-CAG-SD
HMGA2	high mobility group AT-hook 2 [Source:HGNC Symbol;Acc:5009]	12	66212911	66360075	1182	0.137	1	1	8.552E-03	1.199E-02	PB-CAG-SD
SGK3	serum/glucocorticoid regulated kinase family, member 3 [Source:HGNC Symbol;Acc:10812]	8	67619653	67774257	1116	0.138	1	1	8.641E-03	1.210E-02	PB-CAG-SD
ATAD2B	ATPase family, AAA domain containing 2B [Source:HGNC Symbol;Acc:29230]	2	23971534	24154984	1517	0.138	1	1	8.714E-03	1.218E-02	PB-CAG-SD
EEFSEC	eukaryotic elongation factor, selenocysteine-tRNA-specific [Source:HGNC Symbol;Acc:24614]	3	127867297	128127485	1163	0.139	1	1	8.781E-03	1.226E-02	PB-CAG-SD
PIGN	phosphatidylinositol glycan anchor biosynthesis, class N [Source:HGNC Symbol;Acc:8967]	18	59710800	59859351	1404	0.139	1	1	8.839E-03	1.233E-02	PB-CAG-SD
MTA3	metastasis associated 1 family, member 3 [Source:HGNC Symbol;Acc:23784]	2	42716709	42984087	1540	0.140	1	1	8.968E-03	1.250E-02	PB-CAG-SD
AFF1	AF4/FMR2 family, member 1 [Source:HGNC Symbol;Acc:7135]	4	87851154	88062206	1593	0.141	1	1	8.997E-03	1.252E-02	PB-CAG-SD
FAM135A	family with sequence similarity 135, member A [Source:HGNC Symbol;Acc:21084]	6	71117644	71270877	1423	0.141	1	1	9.096E-03	1.265E-02	PB-CAG-SD
IGF2BP3	insulin-like growth factor 2 mRNA binding protein 3 [Source:HGNC Symbol;Acc:28868]	7	23349828	23515086	1182	0.142	1	1	9.153E-03	1.271E-02	PB-CAG-SD
CHCHD6	coiled-coil-helix-coiled-coil-helix domain containing 6 [Source:HGNC Symbol;Acc:28184]	3	126418063	126679249	1190	0.142	1	1	9.174E-03	1.273E-02	PB-CAG-SD
RFX7	regulatory factor X, 7 [Source:HGNC Symbol;Acc:25777]	15	56379478	56540483	1378	0.142	1	1	9.226E-03	1.278E-02	PB-CAG-SD

table S2. Genes with significant transposon integrations in RKO *BRAF*$^{wt/-/-}$ cells after selection in low glucose medium

Gene ID	Description	Chrom	Start	End	# TTAA sites	Expected	Observed	Libraries	*P*	FDR	Transposon
ADAM10	ADAM metallopeptidase domain 10 [Source:HGNC Symbol;Acc:188]	15	58887403	59047177	1382	0.143	1	1	9.277E-03	1.284E-02	PB-CAG-SD
SEL1L2	sel-1 suppressor of lin-12-like 2 (C. elegans) [Source:HGNC Symbol;Acc:15897]	20	13829893	13982089	995	0.143	1	1	9.296E-03	1.285E-02	PB-CAG-SD
LGR5	leucine-rich repeat containing G protein-coupled receptor 5 [Source:HGNC Symbol;Acc:4504]	12	71828550	71980090	1236	0.143	1	1	9.312E-03	1.286E-02	PB-CAG-SD
HFM1	HFM1, ATP-dependent DNA helicase homolog (S. cerevisiae) [Source:HGNC Symbol;Acc:20193]	1	91726323	91875426	1184	0.143	1	1	9.352E-03	1.290E-02	PB-CAG-SD
ZZZ3	zinc finger, ZZ-type containing 3 [Source:HGNC Symbol;Acc:24523]	1	78028101	78154104	1189	0.144	1	1	9.427E-03	1.299E-02	PB-CAG-SD
PLCXD2	phosphatidylinositol-specific phospholipase C, X domain containing 2 [Source:HGNC Symbol;Acc:26462]	3	111388523	111565294	1213	0.145	1	1	9.515E-03	1.309E-02	PB-CAG-SD
RFTN1	raftlin, lipid raft linker 1 [Source:HGNC Symbol;Acc:30278]	3	16355081	16560533	1214	0.145	1	1	9.530E-03	1.310E-02	PB-CAG-SD
NMD3	NMD3 homolog (S. cerevisiae) [Source:HGNC Symbol;Acc:24250]	3	160817484	160971320	1219	0.145	1	1	9.605E-03	1.319E-02	PB-CAG-SD
CCDC59	coiled-coil domain containing 59 [Source:HGNC Symbol;Acc:25005]	12	82617460	82757584	1261	0.146	1	1	9.674E-03	1.327E-02	PB-CAG-SD
DTD1	D-tyrosyl-tRNA deacylase 1 [Source:HGNC Symbol;Acc:16219]	20	18563537	18744561	1020	0.147	1	1	9.746E-03	1.335E-02	PB-CAG-SD
SMARCC1	SWI/SNF related, matrix associated, actin dependent regulator of chromatin, subfamily c, member 1 [Source:HGNC Symbol;Ac	3	47626762	47828596	1232	0.147	1	1	9.801E-03	1.341E-02	PB-CAG-SD
INVS	inversin [Source:HGNC Symbol;Acc:17870]	9	102856538	103063282	1497	0.148	1	1	9.874E-03	1.348E-02	PB-CAG-SD
PPP2R5E	protein phosphatase 2, regulatory subunit B', epsilon isoform [Source:HGNC Symbol;Acc:9313]	14	63838075	64015092	1348	0.148	1	1	9.878E-03	1.348E-02	PB-CAG-SD
C1orf101	chromosome 1 open reading frame 101 [Source:HGNC Symbol;Acc:28491]	1	244612679	244804479	1233	0.149	1	1	1.010E-02	1.378E-02	PB-CAG-SD
AXDND1	axonemal dynein light chain domain containing 1 [Source:HGNC Symbol;Acc:26564]	1	179329855	179523870	1237	0.150	1	1	1.017E-02	1.384E-02	PB-CAG-SD
DNAH3	dynein, axonemal, heavy chain 3 [Source:HGNC Symbol;Acc:2949]	16	20944433	21175762	1150	0.150	1	1	1.020E-02	1.388E-02	PB-CAG-SD
ANKRD31	ankyrin repeat domain 31 [Source:HGNC Symbol;Acc:26853]	5	74364100	74537703	1414	0.150	1	1	1.021E-02	1.388E-02	PB-CAG-SD
GRIA3	glutamate receptor, ionotropic, AMPA 3 [Source:HGNC Symbol;Acc:4573]	X	122313006	122624766	2191	0.150	1	1	1.023E-02	1.388E-02	PB-CAG-SD
PIK3CB	phosphatidylinositol-4,5-bisphosphate 3-kinase, catalytic subunit beta [Source:HGNC Symbol;Acc:8976]	3	138372860	138558780	1261	0.150	1	1	1.024E-02	1.389E-02	PB-CAG-SD
CACNB4	calcium channel, voltage-dependent, beta 4 subunit [Source:HGNC Symbol;Acc:1404]	2	152689290	152960593	1659	0.151	1	1	1.033E-02	1.399E-02	PB-CAG-SD
STON1-GTF2A1L	STON1-GTF2A1L readthrough [Source:HGNC Symbol;Acc:30651]	2	48752064	49003654	1660	0.151	1	1	1.034E-02	1.399E-02	PB-CAG-SD
SLC25A12	solute carrier family 25 (aspartate/glutamate carrier), member 12 [Source:HGNC Symbol;Acc:10982]	2	172640880	172869766	1671	0.152	1	1	1.048E-02	1.414E-02	PB-CAG-SD
COL8A1	collagen, type VIII, alpha 1 [Source:HGNC Symbol;Acc:2215]	3	99352319	99518070	1276	0.152	1	1	1.048E-02	1.414E-02	PB-CAG-SD
AOAH	acyloxyacyl hydrolase (neutrophil) [Source:HGNC Symbol;Acc:548]	7	36552456	36769154	1278	0.153	1	1	1.062E-02	1.432E-02	PB-CAG-SD
SPIRE1	spire homolog 1 (Drosophila) [Source:HGNC Symbol;Acc:30622]	18	12446511	12663133	1551	0.154	1	1	1.068E-02	1.439E-02	PB-CAG-SD
WNK1	WNK lysine deficient protein kinase 1 [Source:HGNC Symbol;Acc:14540]	12	856759	1020618	1335	0.155	1	1	1.078E-02	1.450E-02	PB-CAG-SD
ADAM32	ADAM metallopeptidase domain 32 [Source:HGNC Symbol;Acc:15479]	8	38959509	39142430	1254	0.155	1	1	1.079E-02	1.450E-02	PB-CAG-SD
SAMD4A	sterile alpha motif domain containing 4A [Source:HGNC Symbol;Acc:23023]	14	55028815	55260033	1418	0.155	1	1	1.088E-02	1.460E-02	PB-CAG-SD
GIPC2	GIPC PDZ domain containing family, member 2 [Source:HGNC Symbol;Acc:18177]	1	78440226	78604133	1285	0.156	1	1	1.093E-02	1.465E-02	PB-CAG-SD
TBCA	tubulin folding cofactor A [Source:HGNC Symbol;Acc:11579]	5	76986991	77169604	1467	0.156	1	1	1.095E-02	1.467E-02	PB-CAG-SD
NR2F2-AS1	NR2F2 antisense RNA 1 [Source:HGNC Symbol;Acc:44222]	15	96670598	96875590	1510	0.156	1	1	1.098E-02	1.469E-02	PB-CAG-SD
ALDH1A1	aldehyde dehydrogenase 1 family, member A1 [Source:HGNC Symbol;Acc:402]	9	75515578	75700358	1587	0.156	1	1	1.103E-02	1.474E-02	PB-CAG-SD
USP6NL	USP6 N-terminal like [Source:HGNC Symbol;Acc:16858]	10	11502509	11658753	1377	0.157	1	1	1.114E-02	1.487E-02	PB-CAG-SD
VMP1	vacuole membrane protein 1 [Source:HGNC Symbol;Acc:29559]	17	57779553	57919616	1031	0.158	1	1	1.124E-02	1.499E-02	PB-CAG-SD
SASH1	SAM and SH3 domain containing 1 [Source:HGNC Symbol;Acc:19182]	6	148588440	148873186	1608	0.160	1	1	1.147E-02	1.528E-02	PB-CAG-SD
EML6	echinoderm microtubule associated protein like 6 [Source:HGNC Symbol;Acc:35412]	2	54945636	55199157	1756	0.160	1	1	1.151E-02	1.531E-02	PB-CAG-SD
PPP2R3A	protein phosphatase 2, regulatory subunit B'', alpha [Source:HGNC Symbol;Acc:9307]	3	135679515	135866733	1343	0.160	1	1	1.155E-02	1.534E-02	PB-CAG-SD
NEK1	NIMA-related kinase 1 [Source:HGNC Symbol;Acc:7744]	4	170314426	170538780	1824	0.161	1	1	1.164E-02	1.545E-02	PB-CAG-SD
COL22A1	collagen, type XXII, alpha 1 [Source:HGNC Symbol;Acc:22989]	8	139600478	139931249	1310	0.162	1	1	1.172E-02	1.554E-02	PB-CAG-SD
SAMD3	sterile alpha motif domain containing 3 [Source:HGNC Symbol;Acc:21574]	6	130465460	130691570	1638	0.163	1	1	1.188E-02	1.574E-02	PB-CAG-SD
PVRL3-AS1	PVRL3 antisense RNA 1 [Source:HGNC Symbol;Acc:40813]	3	110612555	110795400	1369	0.163	1	1	1.197E-02	1.584E-02	PB-CAG-SD
FAM49B	family with sequence similarity 49, member B [Source:HGNC Symbol;Acc:25216]	8	130851839	131034375	1330	0.164	1	1	1.206E-02	1.594E-02	PB-CAG-SD
DGKH	diacylglycerol kinase, eta [Source:HGNC Symbol;Acc:2854]	13	42609176	42830714	1903	0.164	1	1	1.210E-02	1.596E-02	PB-CAG-SD
DMXL2	Dmx-like 2 [Source:HGNC Symbol;Acc:2938]	15	51739908	51920030	1591	0.164	1	1	1.212E-02	1.596E-02	PB-CAG-SD
SMG6	SMG6 nonsense mediated mRNA decay factor [Source:HGNC Symbol;Acc:17809]	17	1963133	2212065	1073	0.164	1	1	1.213E-02	1.596E-02	PB-CAG-SD
TRPS1	trichorhinophalangeal syndrome I [Source:HGNC Symbol;Acc:12340]	8	116420724	116826899	3802	0.469	2	2	1.213E-02	1.596E-02	PB-CAG-SD
SATB2	SATB homeobox 2 [Source:HGNC Symbol;Acc:21637]	2	200134223	200340989	1807	0.165	1	1	1.215E-02	1.597E-02	PB-CAG-SD
STARD4-AS1	STARD4 antisense RNA 1 [Source:HGNC Symbol;Acc:44117]	5	110842924	111075423	1553	0.165	1	1	1.220E-02	1.602E-02	PB-CAG-SD
NDUFAF6	NADH dehydrogenase (ubiquinone) complex I, assembly factor 6 [Source:HGNC Symbol;Acc:28625]	8	95902995	96128683	1340	0.165	1	1	1.223E-02	1.605E-02	PB-CAG-SD
CTNNBL1	catenin, beta like 1 [Source:HGNC Symbol;Acc:15879]	20	36317408	36500531	1151	0.165	1	1	1.226E-02	1.606E-02	PB-CAG-SD
ITGA1	integrin, alpha 1 [Source:HGNC Symbol;Acc:6134]	5	52078730	52255040	1565	0.166	1	1	1.238E-02	1.620E-02	PB-CAG-SD
RUNX1T1	runt-related transcription factor 1; translocated to, 1 (cyclin D-related) [Source:HGNC Symbol;Acc:1535]	8	92967203	93120514	1353	0.167	1	1	1.246E-02	1.629E-02	PB-CAG-SD
RBPJ	recombination signal binding protein for immunoglobulin kappa J region [Source:HGNC Symbol;Acc:5724]	4	26160077	26436541	1895	0.167	1	1	1.251E-02	1.633E-02	PB-CAG-SD
CDYL	chromodomain protein, Y-like [Source:HGNC Symbol;Acc:1811]	6	4701393	4955785	1684	0.167	1	1	1.252E-02	1.633E-02	PB-CAG-SD
KLHL32	kelch-like family member 32 [Source:HGNC Symbol;Acc:21221]	6	97367605	97588630	1686	0.167	1	1	1.255E-02	1.635E-02	PB-CAG-SD
ACSM3	acyl-CoA synthetase medium-chain family member 3 [Source:HGNC Symbol;Acc:10522]	16	20616565	20808903	1291	0.169	1	1	1.270E-02	1.653E-02	PB-CAG-SD
LINC00693	long intergenic non-protein coding RNA 693 [Source:HGNC Symbol;Acc:44526]	3	28611282	28799831	1425	0.170	1	1	1.291E-02	1.678E-02	PB-CAG-SD
QKI	QKI, KH domain containing, RNA binding [Source:HGNC Symbol;Acc:21100]	6	163830032	163999628	1712	0.170	1	1	1.292E-02	1.678E-02	PB-CAG-SD
CPS1	carbamoyl-phosphate synthase 1, mitochondrial [Source:HGNC Symbol;Acc:2323]	2	211337406	211543831	1871	0.170	1	1	1.298E-02	1.684E-02	PB-CAG-SD
PLCXD3	phosphatidylinositol-specific phospholipase C, X domain containing 3 [Source:HGNC Symbol;Acc:31822]	5	41307056	41515730	1613	0.171	1	1	1.311E-02	1.699E-02	PB-CAG-SD
DOCK9	dedicator of cytokinesis 9 [Source:HGNC Symbol;Acc:14132]	13	99445741	99743879	2000	0.173	1	1	1.329E-02	1.721E-02	PB-CAG-SD
CTTNBP2	cortactin binding protein 2 [Source:HGNC Symbol;Acc:15679]	7	117350705	117519193	1440	0.173	1	1	1.331E-02	1.721E-02	PB-CAG-SD
C8orf44-SGK3	C8orf44-SGK3 readthrough [Source:HGNC Symbol;Acc:48354]	8	67574831	67772707	1402	0.173	1	1	1.333E-02	1.721E-02	PB-CAG-SD
UBR5	ubiquitin protein ligase E3 component n-recognin 5 [Source:HGNC Symbol;Acc:16806]	8	103265240	103430069	1403	0.173	1	1	1.334E-02	1.722E-02	PB-CAG-SD
PKP4	plakophilin 4 [Source:HGNC Symbol;Acc:9026]	2	159308476	159539391	1904	0.173	1	1	1.341E-02	1.729E-02	PB-CAG-SD
TGFBR3	transforming growth factor, beta receptor III [Source:HGNC Symbol;Acc:11774]	1	92145902	92376892	1436	0.174	1	1	1.348E-02	1.736E-02	PB-CAG-SD
TAF3	TAF3 RNA polymerase II, TATA box binding protein (TBP)-associated factor, 140kDa [Source:HGNC Symbol;Acc:17303]	10	7855467	8058590	1527	0.174	1	1	1.355E-02	1.742E-02	PB-CAG-SD
BRWD1	bromodomain and WD repeat domain containing 1 [Source:HGNC Symbol;Acc:12760]	21	40556102	40698485	1202	0.174	1	1	1.356E-02	1.742E-02	PB-CAG-SD
CDH10	cadherin 10, type 2 (T2-cadherin) [Source:HGNC Symbol;Acc:1749]	5	24487209	24650087	1645	0.175	1	1	1.360E-02	1.746E-02	PB-CAG-SD
TFDP2	transcription factor Dp-2 (E2F dimerization partner 2) [Source:HGNC Symbol;Acc:11751]	3	141663277	141873386	1466	0.175	1	1	1.362E-02	1.747E-02	PB-CAG-SD
EEA1	early endosome antigen 1 [Source:HGNC Symbol;Acc:3185]	12	93164413	93328107	1512	0.175	1	1	1.365E-02	1.748E-02	PB-CAG-SD
WDPCP	WD repeat containing planar cell polarity effector [Source:HGNC Symbol;Acc:28027]	2	63348518	64059977	5431	0.495	2	1	1.399E-02	1.790E-02	PB-CAG-SD
KAT6B	K(lysine) acetyltransferase 6B [Source:HGNC Symbol;Acc:17582]	10	76580340	76792380	1554	0.177	1	1	1.400E-02	1.790E-02	PB-CAG-SD
LHFPL2	lipoma HMGIC fusion partner-like 2 [Source:HGNC Symbol;Acc:6588]	5	77781038	78070844	1674	0.178	1	1	1.406E-02	1.795E-02	PB-CAG-SD
HS6ST3	heparan sulfate 6-O-sulfotransferase 3 [Source:HGNC Symbol;Acc:19134]	13	96738093	97485671	5750	0.496	2	2	1.411E-02	1.800E-02	PB-CAG-SD
SLMAP	sarcolemma associated protein [Source:HGNC Symbol;Acc:16643]	3	57736177	57914895	1498	0.179	1	1	1.419E-02	1.808E-02	PB-CAG-SD
PARN	poly(A)-specific ribonuclease [Source:HGNC Symbol;Acc:8609]	16	14529558	14731585	1371	0.179	1	1	1.423E-02	1.811E-02	PB-CAG-SD
SUPT3H	suppressor of Ty 3 homolog (S. cerevisiae) [Source:HGNC Symbol;Acc:11466]	6	44777054	45350690	5031	0.500	2	2	1.437E-02	1.826E-02	PB-CAG-SD
NCAM1	neural cell adhesion molecule 1 [Source:HGNC Symbol;Acc:7656]	11	112826997	113149158	2069	0.180	1	1	1.440E-02	1.829E-02	PB-CAG-SD
PALM2	paralemmin 2 [Source:HGNC Symbol;Acc:15845]	9	112398068	112713755	1834	0.181	1	1	1.450E-02	1.840E-02	PB-CAG-SD
WWTR1	WW domain containing transcription regulator 1 [Source:HGNC Symbol;Acc:24042]	3	149235022	149459501	1518	0.181	1	1	1.455E-02	1.844E-02	PB-CAG-SD
HUS1	HUS1 checkpoint homolog (S. pombe) [Source:HGNC Symbol;Acc:5309]	7	47735328	48024178	1520	0.182	1	1	1.474E-02	1.866E-02	PB-CAG-SD
METTL15	methyltransferase like 15 [Source:HGNC Symbol;Acc:26606]	11	28124795	28355054	2101	0.183	1	1	1.482E-02	1.874E-02	PB-CAG-SD
UGGT2	UDP-glucose glycoprotein glucosyltransferase 2 [Source:HGNC Symbol;Acc:15664]	13	96453834	96710736	2122	0.183	1	1	1.486E-02	1.878E-02	PB-CAG-SD
HMBOX1	homeobox containing 1 [Source:HGNC Symbol;Acc:26137]	8	28742911	28922281	1495	0.184	1	1	1.504E-02	1.898E-02	PB-CAG-SD
SENP7	SUMO1/sentrin specific peptidase 7 [Source:HGNC Symbol;Acc:30402]	3	101043049	101237085	1547	0.185	1	1	1.508E-02	1.901E-02	PB-CAG-SD
ATP8A1	ATPase, aminophospholipid transporter (APLT), class I, type 8A, member 1 [Source:HGNC Symbol;Acc:13531]	4	42410390	42664122	2099	0.185	1	1	1.517E-02	1.910E-02	PB-CAG-SD
SLC25A13	solute carrier family 25 (aspartate/glutamate carrier), member 13 [Source:HGNC Symbol;Acc:10983]	7	95749532	95956459	1558	0.187	1	1	1.544E-02	1.942E-02	PB-CAG-SD
RGS7	regulator of G-protein signaling 7 [Source:HGNC Symbol;Acc:10003]	1	240931554	241525530	4242	0.514	2	1	1.546E-02	1.943E-02	PB-CAG-SD
CHD7	chromodomain helicase DNA binding protein 7 [Source:HGNC Symbol;Acc:20626]	8	61586337	61779465	1544	0.190	1	1	1.598E-02	2.006E-02	PB-CAG-SD
FBN1	fibrillin 1 [Source:HGNC Symbol;Acc:3603]	15	48700503	48943046	1852	0.191	1	1	1.614E-02	2.024E-02	PB-CAG-SD
PDGFD	platelet derived growth factor D [Source:HGNC Symbol;Acc:30620]	11	103777914	104040107	2213	0.193	1	1	1.634E-02	2.047E-02	PB-CAG-SD
IQCH	IQ motif containing H [Source:HGNC Symbol;Acc:25721]	15	67542138	67794598	1866	0.193	1	1	1.637E-02	2.048E-02	PB-CAG-SD
NDUFAF2	NADH dehydrogenase (ubiquinone) complex I, assembly factor 2 [Source:HGNC Symbol;Acc:28086]	5	60235956	60448853	1820	0.193	1	1	1.645E-02	2.057E-02	PB-CAG-SD
RSPO2	R-spondin 2 [Source:HGNC Symbol;Acc:28583]	8	108911544	109100913	1579	0.195	1	1	1.666E-02	2.078E-02	PB-CAG-SD
FBXW7	F-box and WD repeat domain containing 7, E3 ubiquitin protein ligase [Source:HGNC Symbol;Acc:16712]	4	153242410	153462253	2207	0.195	1	1	1.667E-02	2.078E-02	PB-CAG-SD
RAPGEF6	Rap guanine nucleotide exchange factor (GEF) 6 [Source:HGNC Symbol;Acc:20655]	5	130759614	130975929	1833	0.195	1	1	1.667E-02	2.078E-02	PB-CAG-SD
CNKSR2	connector enhancer of kinase suppressor of Ras 2 [Source:HGNC Symbol;Acc:19701]	X	21387536	21672813	2840	0.195	1	1	1.669E-02	2.078E-02	PB-CAG-SD
CALD1	caldesmon 1 [Source:HGNC Symbol;Acc:1441]	7	134424003	134655479	1626	0.195	1	1	1.672E-02	2.080E-02	PB-CAG-SD
UNC13C	unc-13 homolog C (C. elegans) [Source:HGNC Symbol;Acc:23149]	15	54300101	54920806	5157	0.533	2	1	1.701E-02	2.113E-02	PB-CAG-SD
NOVA1-AS1	NOVA1 antisense RNA 1 (head to head) [Source:HGNC Symbol;Acc:19827]	14	27062618	27275673	1799	0.197	1	1	1.703E-02	2.114E-02	PB-CAG-SD
OCA2	oculocutaneous albinism II [Source:HGNC Symbol;Acc:8101]	15	28000021	28349504	1912	0.198	1	1	1.713E-02	2.124E-02	PB-CAG-SD
PPP1R3A	protein phosphatase 1, regulatory subunit 3A [Source:HGNC Symbol;Acc:9291]	7	113516832	113720975	1659	0.199	1	1	1.736E-02	2.151E-02	PB-CAG-SD
GABBR2	gamma-aminobutyric acid (GABA) B receptor, 2 [Source:HGNC Symbol;Acc:4507]	9	101050391	101476479	2031	0.200	1	1	1.756E-02	2.172E-02	PB-CAG-SD
FGD4	FYVE, RhoGEF and PH domain containing 4 [Source:HGNC Symbol;Acc:19125]	12	32547463	32798984	1732	0.201	1	1	1.761E-02	2.176E-02	PB-CAG-SD
FER1L6	fer-1-like 6 (C. elegans) [Source:HGNC Symbol;Acc:28065]	8	124859227	125132302	1627	0.201	1	1	1.762E-02	2.176E-02	PB-CAG-SD
ROR2	receptor tyrosine kinase-like orphan receptor 2 [Source:HGNC Symbol;Acc:10257]	9	94325373	94717444	2048	0.202	1	1	1.783E-02	2.200E-02	PB-CAG-SD
FRMD6	FERM domain containing 6 [Source:HGNC Symbol;Acc:19839]	14	51950818	52197445	1845	0.202	1	1	1.785E-02	2.200E-02	PB-CAG-SD
USP34	ubiquitin specific peptidase 34 [Source:HGNC Symbol;Acc:20066]	2	61414598	61702904	2230	0.203	1	1	1.804E-02	2.221E-02	PB-CAG-SD
PHLPP1	PH domain and leucine rich repeat protein phosphatase 1 [Source:HGNC Symbol;Acc:20610]	18	60377672	60647666	2057	0.204	1	1	1.818E-02	2.236E-02	PB-CAG-SD
BIRC6	baculoviral IAP repeat containing 6 [Source:HGNC Symbol;Acc:13516]	2	32577096	32843966	2252	0.205	1	1	1.838E-02	2.258E-02	PB-CAG-SD
SLC26A7	solute carrier family 26, member 7 [Source:HGNC Symbol;Acc:14467]	8	92216722	92410378	1675	0.207	1	1	1.860E-02	2.282E-02	PB-CAG-SD
BRAF	v-raf murine sarcoma viral oncogene homolog B [Source:HGNC Symbol;Acc:1097]	7	140424943	140629564	1722	0.207	1	1	1.862E-02	2.282E-02	PB-CAG-SD
PCNX	pecanex homolog (Drosophila) [Source:HGNC Symbol;Acc:19740]	14	71369122	71582099	1890	0.207	1	1	1.868E-02	2.286E-02	PB-CAG-SD
MYO5B	myosin VB [Source:HGNC Symbol;Acc:7603]	18	47349183	47726463	2087	0.207	1	1	1.868E-02	2.286E-02	PB-CAG-SD
CDH2	cadherin 2, type 1, N-cadherin (neuronal) [Source:HGNC Symbol;Acc:1759]	18	25530930	25762410	2093	0.208	1	1	1.878E-02	2.296E-02	PB-CAG-SD
RGS5	regulator of G-protein signaling 5 [Source:HGNC Symbol;Acc:10001]	1	163080911	163296577	1725	0.209	1	1	1.901E-02	2.320E-02	PB-CAG-SD
PHACTR2	phosphatase and actin regulator 2 [Source:HGNC Symbol;Acc:20956]	6	143852982	144152322	2104	0.209	1	1	1.902E-02	2.320E-02	PB-CAG-SD
MCTP2	multiple C2 domains, transmembrane 2 [Source:HGNC Symbol;Acc:25636]	15	94769767	95023632	2028	0.210	1	1	1.912E-02	2.330E-02	PB-CAG-SD
AKAP2	A kinase (PRKA) anchor protein 2 [Source:HGNC Symbol;Acc:372]	9	112537769	112934792	2137	0.211	1	1	1.930E-02	2.350E-02	PB-CAG-SD
PALM2-AKAP2	PALM2-AKAP2 readthrough [Source:HGNC Symbol;Acc:33529]	9	112537589	112934792	2138	0.211	1	1	1.932E-02	2.350E-02	PB-CAG-SD
GRIA1	glutamate receptor, ionotropic, AMPA 1 [Source:HGNC Symbol;Acc:4571]	5	152864175	153193429	1998	0.212	1	1	1.958E-02	2.379E-02	PB-CAG-SD
CAV2	caveolin 2 [Source:HGNC Symbol;Acc:1528]	7	115922434	116148595	1779	0.213	1	1	1.978E-02	2.401E-02	PB-CAG-SD
VRK2	vaccinia related kinase 2 [Source:HGNC Symbol;Acc:12719]	2	58129786	58387055	2350	0.214	1	1	1.989E-02	2.412E-02	PB-CAG-SD
GPC3	glypican 3 [Source:HGNC Symbol;Acc:4451]	X	132669773	133124922	3161	0.217	1	1	2.038E-02	2.468E-02	PB-CAG-SD
FRMD5	FERM domain containing 5 [Source:HGNC Symbol;Acc:28214]	15	44162962	44492450	2107	0.218	1	1	2.053E-02	2.484E-02	PB-CAG-SD
C5orf17	chromosome 5 open reading frame 17 [Source:HGNC Symbol;Acc:26630]	5	23946457	24178372	2060	0.219	1	1	2.072E-02	2.505E-02	PB-CAG-SD
C9orf3	chromosome 9 open reading frame 3 [Source:HGNC Symbol;Acc:1361]	9	97483983	97849441	2237	0.221	1	1	2.102E-02	2.538E-02	PB-CAG-SD
ARHGAP42	Rho GTPase activating protein 42 [Source:HGNC Symbol;Acc:26545]	11	100553384	100862668	2538	0.221	1	1	2.109E-02	2.545E-02	PB-CAG-SD
KANSL1	KAT8 regulatory NSL complex subunit 1 [Source:HGNC Symbol;Acc:24565]	17	44107282	44307733	1444	0.221	1	1	2.116E-02	2.550E-02	PB-CAG-SD
RAPGEF5	Rap guanine nucleotide exchange factor (GEF) 5 [Source:HGNC Symbol;Acc:16862]	7	22157856	22401763	1851	0.222	1	1	2.129E-02	2.564E-02	PB-CAG-SD
PHLDB2	pleckstrin homology-like domain, family B, member 2 [Source:HGNC Symbol;Acc:29573]	3	111446344	111695364	1863	0.222	1	1	2.133E-02	2.566E-02	PB-CAG-SD
ANKRD44	ankyrin repeat domain 44 [Source:HGNC Symbol;Acc:25259]	2	197831741	198180897	2450	0.223	1	1	2.149E-02	2.583E-02	PB-CAG-SD
ASPH	aspartate beta-hydroxylase [Source:HGNC Symbol;Acc:757]	8	62413116	62632155	1818	0.224	1	1	2.167E-02	2.601E-02	PB-CAG-SD
ZCCHC7	zinc finger, CCHC domain containing 7 [Source:HGNC Symbol;Acc:26209]	9	37115536	37358146	2280	0.225	1	1	2.177E-02	2.611E-02	PB-CAG-SD
RNF150	ring finger protein 150 [Source:HGNC Symbol;Acc:23138]	4	141786725	142139031	2553	0.225	1	1	2.186E-02	2.619E-02	PB-CAG-SD
CUX1	cut-like homeobox 1 [Source:HGNC Symbol;Acc:2557]	7	101453959	101927249	1947	0.234	1	1	2.338E-02	2.798E-02	PB-CAG-SD
CDK6	cyclin-dependent kinase 6 [Source:HGNC Symbol;Acc:1777]	7	92234235	92470908	1950	0.234	1	1	2.345E-02	2.804E-02	PB-CAG-SD

table S2. Genes with significant transposon integrations in RKO *BRAF*$^{wt/-/-}$ cells after selection in low glucose medium

Gene ID	Description	Chrom	Start	End	# TTAA sites	Expected	Observed	Libraries	*P*	FDR	Transposon
C14orf37	chromosome 14 open reading frame 37 [Source:HGNC Symbol;Acc:19846]	14	58466453	58769857	2149	0.235	1	1	2.370E-02	2.831E-02	PB-CAG-SD
RERG	RAS-like, estrogen-regulated, growth inhibitor [Source:HGNC Symbol;Acc:15980]	12	15260717	15506609	2045	0.237	1	1	2.397E-02	2.861E-02	PB-CAG-SD
TTLL5	tubulin tyrosine ligase-like family, member 5 [Source:HGNC Symbol;Acc:19963]	14	76094968	76421421	2188	0.240	1	1	2.450E-02	2.921E-02	PB-CAG-SD
CADPS2	Ca++-dependent secretion activator 2 [Source:HGNC Symbol;Acc:16018]	7	121958481	122531813	5135	0.616	2	2	2.473E-02	2.945E-02	PB-CAG-SD
HELZ	helicase with zinc finger [Source:HGNC Symbol;Acc:16878]	17	65066554	65247105	1573	0.241	1	1	2.479E-02	2.949E-02	PB-CAG-SD
ANO2	anoctamin 2 [Source:HGNC Symbol;Acc:1183]	12	5641035	6060398	2088	0.242	1	1	2.491E-02	2.961E-02	PB-CAG-SD
DNAJC1	DnaJ (Hsp40) homolog, subfamily C, member 1 [Source:HGNC Symbol;Acc:20090]	10	22045466	22297698	2121	0.242	1	1	2.500E-02	2.969E-02	PB-CAG-SD
C2orf88	chromosome 2 open reading frame 88 [Source:HGNC Symbol;Acc:28191]	2	190739335	191068210	2672	0.243	1	1	2.523E-02	2.993E-02	PB-CAG-SD
LDB2	LIM domain binding 2 [Source:HGNC Symbol;Acc:6533]	4	16503164	16905432	2768	0.244	1	1	2.538E-02	3.007E-02	PB-CAG-SD
PCSK2	proprotein convertase subtilisin/kexin type 2 [Source:HGNC Symbol;Acc:8744]	20	17201752	17465223	1709	0.246	1	1	2.564E-02	3.035E-02	PB-CAG-SD
NSMCE2	non-SMC element 2, MMS21 homolog (S. cerevisiae) [Source:HGNC Symbol;Acc:26513]	8	126098921	126379362	1998	0.246	1	1	2.579E-02	3.050E-02	PB-CAG-SD
CUBN	cubilin (intrinsic factor-cobalamin receptor) [Source:HGNC Symbol;Acc:2548]	10	16865963	17176830	2163	0.247	1	1	2.592E-02	3.063E-02	PB-CAG-SD
DDAH1	dimethylarginine dimethylaminohydrolase 1 [Source:HGNC Symbol;Acc:2715]	1	85784164	86048933	2051	0.248	1	1	2.620E-02	3.092E-02	PB-CAG-SD
ADAM12	ADAM metallopeptidase domain 12 [Source:HGNC Symbol;Acc:190]	10	127700950	128082024	2197	0.251	1	1	2.668E-02	3.146E-02	PB-CAG-SD
ADAMTS12	ADAM metallopeptidase with thrombospondin type 1 motif, 12 [Source:HGNC Symbol;Acc:14605]	5	33523640	33897297	2368	0.252	1	1	2.680E-02	3.158E-02	PB-CAG-SD
RGL1	ral guanine nucleotide dissociation stimulator-like 1 [Source:HGNC Symbol;Acc:30281]	1	183600208	183897666	2087	0.253	1	1	2.705E-02	3.183E-02	PB-CAG-SD
ZNF280D	zinc finger protein 280D [Source:HGNC Symbol;Acc:25953]	15	56922379	57215769	2450	0.253	1	1	2.712E-02	3.189E-02	PB-CAG-SD
TIAM2	T-cell lymphoma invasion and metastasis 2 [Source:HGNC Symbol;Acc:11806]	6	155148831	155578857	2556	0.254	1	1	2.726E-02	3.202E-02	PB-CAG-SD
AK5	adenylate kinase 5 [Source:HGNC Symbol;Acc:365]	1	77742736	78025651	2115	0.256	1	1	2.772E-02	3.252E-02	PB-CAG-SD
CFTR	cystic fibrosis transmembrane conductance regulator (ATP-binding cassette sub-family C, member 7) [Source:HGNC Symbol;A	7	117100838	117356025	2140	0.257	1	1	2.782E-02	3.262E-02	PB-CAG-SD
MYO3A	myosin IIIA [Source:HGNC Symbol;Acc:7601]	10	26218196	26501456	2253	0.257	1	1	2.794E-02	3.272E-02	PB-CAG-SD
CAMK2D	calcium/calmodulin-dependent protein kinase II delta [Source:HGNC Symbol;Acc:1462]	4	114372188	114688083	2918	0.257	1	1	2.796E-02	3.272E-02	PB-CAG-SD
ERG	v-ets avian erythroblastosis virus E26 oncogene homolog [Source:HGNC Symbol;Acc:3446]	21	39751949	40038704	1777	0.258	1	1	2.806E-02	3.280E-02	PB-CAG-SD
EYA4	eyes absent homolog 4 (Drosophila) [Source:HGNC Symbol;Acc:3522]	6	133556736	133853258	2604	0.259	1	1	2.820E-02	3.294E-02	PB-CAG-SD
ANK3	ankyrin 3, node of Ranvier (ankyrin G) [Source:HGNC Symbol;Acc:494]	10	61788159	62498248	5690	0.650	2	2	2.832E-02	3.304E-02	PB-CAG-SD
ANO10	anoctamin 10 [Source:HGNC Symbol;Acc:25519]	3	43396351	43738086	2176	0.260	1	1	2.840E-02	3.310E-02	PB-CAG-SD
FAM189A1	family with sequence similarity 189, member A1 [Source:HGNC Symbol;Acc:29075]	15	29412457	29867927	2519	0.260	1	1	2.854E-02	3.323E-02	PB-CAG-SD
TNR	tenascin R [Source:HGNC Symbol;Acc:11953]	1	175291935	175717906	2166	0.262	1	1	2.895E-02	3.368E-02	PB-CAG-SD
ZFAND3	zinc finger, AN1-type domain 3 [Source:HGNC Symbol;Acc:18019]	6	37782275	38122400	2647	0.263	1	1	2.906E-02	3.377E-02	PB-CAG-SD
BAZ2B	bromodomain adjacent to zinc finger domain, 2B [Source:HGNC Symbol;Acc:963]	2	160175490	160478203	2903	0.264	1	1	2.938E-02	3.407E-02	PB-CAG-SD
FILIP1L	filamin A interacting protein 1-like [Source:HGNC Symbol;Acc:24589]	3	99548985	99838357	2217	0.265	1	1	2.939E-02	3.407E-02	PB-CAG-SD
LRRC16A	leucine rich repeat containing 16A [Source:HGNC Symbol;Acc:21581]	6	25274306	25620758	2664	0.265	1	1	2.940E-02	3.407E-02	PB-CAG-SD
PID1	phosphotyrosine interaction domain containing 1 [Source:HGNC Symbol;Acc:26084]	2	229715242	230141001	2966	0.270	1	1	3.055E-02	3.537E-02	PB-CAG-SD
ANGPT1	angiopoietin 1 [Source:HGNC Symbol;Acc:484]	8	108261721	108515283	2195	0.271	1	1	3.064E-02	3.541E-02	PB-CAG-SD
RALGAPA1	Ral GTPase activating protein, alpha subunit 1 (catalytic) [Source:HGNC Symbol;Acc:17770]	14	36007558	36283510	2472	0.271	1	1	3.065E-02	3.541E-02	PB-CAG-SD
MARCH1	membrane-associated ring finger (C3HC4) 1, E3 ubiquitin protein ligase [Source:HGNC Symbol;Acc:26077]	4	164445450	165310202	7616	0.672	2	2	3.081E-02	3.557E-02	PB-CAG-SD
ADARB2	adenosine deaminase, RNA-specific, B2 (non-functional) [Source:HGNC Symbol;Acc:227]	10	1228073	1784670	2432	0.278	1	1	3.212E-02	3.704E-02	PB-CAG-SD
SLC4A4	solute carrier family 4, sodium bicarbonate cotransporter, member 4 [Source:HGNC Symbol;Acc:11030]	4	72048003	72437804	3156	0.278	1	1	3.226E-02	3.717E-02	PB-CAG-SD
STXBP4	syntaxin binding protein 4 [Source:HGNC Symbol;Acc:19694]	17	53041088	53241646	1826	0.280	1	1	3.257E-02	3.749E-02	PB-CAG-SD
HIVEP3	human immunodeficiency virus type I enhancer binding protein 3 [Source:HGNC Symbol;Acc:13561]	1	41972036	42506596	2313	0.280	1	1	3.263E-02	3.753E-02	PB-CAG-SD
VWA8	von Willebrand factor A domain containing 8 [Source:HGNC Symbol;Acc:29071]	13	42140973	42540256	3250	0.281	1	1	3.271E-02	3.758E-02	PB-CAG-SD
EVI5	ecotropic viral integration site 5 [Source:HGNC Symbol;Acc:3501]	1	92974253	93262961	2318	0.281	1	1	3.276E-02	3.760E-02	PB-CAG-SD
MACF1	microtubule-actin crosslinking factor 1 [Source:HGNC Symbol;Acc:13664]	1	39541988	39952849	2351	0.285	1	1	3.361E-02	3.854E-02	PB-CAG-SD
SYN3	synapsin III [Source:HGNC Symbol;Acc:11496]	22	32908539	33459358	2391	0.286	1	1	3.388E-02	3.881E-02	PB-CAG-SD
SLC35F3	solute carrier family 35, member F3 [Source:HGNC Symbol;Acc:23616]	1	234035679	234460262	2365	0.286	1	1	3.398E-02	3.889E-02	PB-CAG-SD
CRB1	crumbs homolog 1 (Drosophila) [Source:HGNC Symbol;Acc:2343]	1	197165592	197447585	2369	0.287	1	1	3.408E-02	3.897E-02	PB-CAG-SD
FRMPD4	FERM and PDZ domain containing 4 [Source:HGNC Symbol;Acc:29007]	X	12151585	12742642	4186	0.287	1	1	3.413E-02	3.898E-02	PB-CAG-SD
DLG1	discs, large homolog 1 (Drosophila) [Source:HGNC Symbol;Acc:2900]	3	196769431	197031171	2408	0.287	1	1	3.416E-02	3.898E-02	PB-CAG-SD
SLC39A11	solute carrier family 39, member 11 [Source:HGNC Symbol;Acc:14463]	17	70642088	71093851	1876	0.288	1	1	3.420E-02	3.900E-02	PB-CAG-SD
C10orf68	chromosome 10 open reading frame 68 [Source:HGNC Symbol;Acc:25779]	10	32827297	33171802	2531	0.289	1	1	3.453E-02	3.934E-02	PB-CAG-SD
SGCD	sarcoglycan, delta (35kDa dystrophin-associated glycoprotein) [Source:HGNC Symbol;Acc:10807]	5	155292354	156194799	6673	0.709	2	1	3.524E-02	4.010E-02	PB-CAG-SD
AP3B1	adaptor-related protein complex 3, beta 1 subunit [Source:HGNC Symbol;Acc:566]	5	77296349	77595579	2771	0.294	1	1	3.570E-02	4.058E-02	PB-CAG-SD
C3orf67	chromosome 3 open reading frame 67 [Source:HGNC Symbol;Acc:24763]	3	58703092	59040810	2473	0.295	1	1	3.584E-02	4.071E-02	PB-CAG-SD
PPM1L	protein phosphatase, Mg2+/Mn2+ dependent, 1L [Source:HGNC Symbol;Acc:16381]	3	160468390	160796695	2502	0.299	1	1	3.661E-02	4.154E-02	PB-CAG-SD
FRAS1	Fraser syndrome 1 [Source:HGNC Symbol;Acc:19185]	4	78973724	79465423	3386	0.299	1	1	3.665E-02	4.155E-02	PB-CAG-SD
ARHGAP26	Rho GTPase activating protein 26 [Source:HGNC Symbol;Acc:17073]	5	142144949	142608576	2840	0.302	1	1	3.732E-02	4.227E-02	PB-CAG-SD
KIF16B	kinesin family member 16B [Source:HGNC Symbol;Acc:15869]	20	16252749	16559078	2101	0.302	1	1	3.736E-02	4.227E-02	PB-CAG-SD
ASAP1	ArfGAP with SH3 domain, ankyrin repeat and PH domain 1 [Source:HGNC Symbol;Acc:2720]	8	131064353	131460906	2475	0.305	1	1	3.809E-02	4.306E-02	PB-CAG-SD
ZNF804A	zinc finger protein 804A [Source:HGNC Symbol;Acc:21711]	2	185458093	185804219	3387	0.309	1	1	3.886E-02	4.389E-02	PB-CAG-SD
PAK7	p21 protein (Cdc42/Rac)-activated kinase 7 [Source:HGNC Symbol;Acc:15916]	20	9518036	9824689	2151	0.309	1	1	3.897E-02	4.398E-02	PB-CAG-SD
TPRG1	tumor protein p63 regulated 1 [Source:HGNC Symbol;Acc:24759]	3	188660003	189043093	2600	0.310	1	1	3.923E-02	4.423E-02	PB-CAG-SD
NUBPL	nucleotide binding protein-like [Source:HGNC Symbol;Acc:20278]	14	31954162	32330430	2862	0.313	1	1	3.995E-02	4.500E-02	PB-CAG-SD
DYNC1I1	dynein, cytoplasmic 1, intermediate chain 1 [Source:HGNC Symbol;Acc:2963]	7	95396866	95739634	2640	0.317	1	1	4.072E-02	4.582E-02	PB-CAG-SD
PIEZO2	piezo-type mechanosensitive ion channel component 2 [Source:HGNC Symbol;Acc:26270]	18	10666480	11153587	3233	0.321	1	1	4.163E-02	4.680E-02	PB-CAG-SD
NCOA2	nuclear receptor coactivator 2 [Source:HGNC Symbol;Acc:7669]	8	71021997	71321040	2615	0.322	1	1	4.205E-02	4.722E-02	PB-CAG-SD
EXOC4	exocyst complex component 4 [Source:HGNC Symbol;Acc:30389]	7	132932829	133751342	6350	0.762	2	2	4.209E-02	4.723E-02	PB-CAG-SD
MCC	mutated in colorectal cancers [Source:HGNC Symbol;Acc:6935]	5	112357796	112829527	3059	0.325	1	1	4.265E-02	4.781E-02	PB-CAG-SD
SNX29	sorting nexin 29 [Source:HGNC Symbol;Acc:30542]	16	12065594	12668146	2494	0.326	1	1	4.279E-02	4.792E-02	PB-CAG-SD
FNIP1	folliculin interacting protein 1 [Source:HGNC Symbol;Acc:29418]	5	130761584	131137710	3074	0.327	1	1	4.302E-02	4.814E-02	PB-CAG-SD
NPAS3	neuronal PAS domain protein 3 [Source:HGNC Symbol;Acc:19311]	14	33399139	34273382	7051	0.772	2	1	4.349E-02	4.862E-02	PB-CAG-SD
CD96	CD96 molecule [Source:HGNC Symbol;Acc:16892]	3	111006566	111384597	2757	0.329	1	1	4.358E-02	4.867E-02	PB-CAG-SD
KIF26B	kinesin family member 26B [Source:HGNC Symbol;Acc:25484]	1	245313287	245872733	2720	0.329	1	1	4.371E-02	4.877E-02	PB-CAG-SD
STAU2	staufen double-stranded RNA binding protein 2 [Source:HGNC Symbol;Acc:11371]	8	74332604	74664943	2676	0.330	1	1	4.382E-02	4.885E-02	PB-CAG-SD
DNAH11	dynein, axonemal, heavy chain 11 [Source:HGNC Symbol;Acc:2942]	7	21577833	21941457	2759	0.331	1	1	4.407E-02	4.908E-02	PB-CAG-SD
CMSS1	cms1 ribosomal small subunit homolog (yeast) [Source:HGNC Symbol;Acc:28666]	3	99531678	99897447	2785	0.332	1	1	4.437E-02	4.938E-02	PB-CAG-SD

table S2. Genes with significant transposon integrations in RKO *BRAF*$^{wt/-/-}$ cells after selection in low glucose medium. Two libraries were generated by mutagenesis with gene trap (PB-GT) and two by promoter containing (PB-CAG-SD) transposon constructs followed by serial selection in low glucose and hygromycin**.** Gene IDs, descriptions and chromosomal locations were obtained from Ensembl. The number of TTAA sites corresponds to intronic TTAA sites in case of PB-GT and also includes TTAA sites within 5 kB of the gene for PB-CAG-SD transposon libraries. Expected, the number of expected random integrations; Observed, the number of independent integration sites observed by sequencing of splinkerette PCR products from the libraries; Libraries, number of libraries (out of 2) containing integrations in the gene; P, P value calculated using Poisson statistics, FDR, False Discovery Rate correction for multiple testing. An FDR adjusted P value <0.05 was used as cutoff for selecting significant genes.

table S3. Genes with significant transposon integrations in both RKO *BRAF*$^{wt/-/-}$ and *DLD-1 KRAS*$^{wt/-}$ libraries

Gene ID	description	Chrom	Start	End
MARCH6	membrane-associated ring finger (C3HC4) 6, E3 ubiquitin protein ligase [Source:HGNC Symbol;Acc:3055[illegible]	chr5	10353815	10435491
42256	septin 9 [Source:HGNC Symbol;Acc:7323]	chr17	75276651	75496678
AAGAB	alpha- and gamma-adaptin binding protein [Source:HGNC Symbol;Acc:25662]	chr15	67493371	67552533
ABHD2	abhydrolase domain containing 2 [Source:HGNC Symbol;Acc:18717]	chr15	89630690	89745591
ABI1	abl-interactor 1 [Source:HGNC Symbol;Acc:11320]	chr10	27035522	27155016
ACAP2	ArfGAP with coiled-coil, ankyrin repeat and PH domains 2 [Source:HGNC Symbol;Acc:16469]	chr3	194995465	195168807
ACER3	alkaline ceramidase 3 [Source:HGNC Symbol;Acc:16066]	chr11	76571911	76737841
ACIN1	apoptotic chromatin condensation inducer 1 [Source:HGNC Symbol;Acc:17066]	chr14	23527773	23569823
ACTN4	actinin, alpha 4 [Source:HGNC Symbol;Acc:166]	chr19	39138289	39222223
ACTR8	ARP8 actin-related protein 8 homolog (yeast) [Source:HGNC Symbol;Acc:14672]	chr3	53901093	53916229
ACVR1	activin A receptor, type I [Source:HGNC Symbol;Acc:171]	chr2	158592958	158732374
ADAM10	ADAM metallopeptidase domain 10 [Source:HGNC Symbol;Acc:188]	chr15	58887403	59042177
ADAM17	ADAM metallopeptidase domain 17 [Source:HGNC Symbol;Acc:195]	chr2	9628615	9695921
ADAM32	ADAM metallopeptidase domain 32 [Source:HGNC Symbol;Acc:15479]	chr8	38959509	39142430
ADAMTS6	ADAM metallopeptidase with thrombospondin type 1 motif, 6 [Source:HGNC Symbol;Acc:222]	chr5	64444563	64782747
ADARB2	adenosine deaminase, RNA-specific, B2 (non-functional) [Source:HGNC Symbol;Acc:227]	chr10	1228073	1784670
ADCY7	adenylate cyclase 7 [Source:HGNC Symbol;Acc:238]	chr16	50275048	50352046
ADCY9	adenylate cyclase 9 [Source:HGNC Symbol;Acc:240]	chr16	4003388	4166186
ADNP	activity-dependent neuroprotector homeobox [Source:HGNC Symbol;Acc:15766]	chr20	49505453	49547958
ADORA2A-AS1	ADORA2A antisense RNA 1 [Source:HGNC Symbol;Acc:37122]	chr22	24825178	24891042
AGA	aspartylglucosaminidase [Source:HGNC Symbol;Acc:318]	chr4	178351924	178363657
AHNAK	AHNAK nucleoprotein [Source:HGNC Symbol;Acc:347]	chr11	62201016	62323707
AK7	adenylate kinase 7 [Source:HGNC Symbol;Acc:20091]	chr14	96858448	96955764
AKAP12	A kinase (PRKA) anchor protein 12 [Source:HGNC Symbol;Acc:370]	chr6	151556134	151679692
AKR1C3	aldo-keto reductase family 1, member C3 [Source:HGNC Symbol;Acc:386]	chr10	5077546	5149878
ALDH1L2	aldehyde dehydrogenase 1 family, member L2 [Source:HGNC Symbol;Acc:26777]	chr12	105413568	105478355
ALDH2	aldehyde dehydrogenase 2 family (mitochondrial) [Source:HGNC Symbol;Acc:404]	chr12	112204691	112247782
ALG10	ALG10, alpha-1,2-glucosyltransferase [Source:HGNC Symbol;Acc:23162]	chr12	34175216	34182629
ANGPT1	angiopoietin 1 [Source:HGNC Symbol;Acc:484]	chr8	108261721	108515283
ANK1	ankyrin 1, erythrocytic [Source:HGNC Symbol;Acc:492]	chr8	41510739	41759280
ANKRD30BL	ankyrin repeat domain 30B-like [Source:HGNC Symbol;Acc:35167]	chr2	132905164	133015542
ANKRD50	ankyrin repeat domain 50 [Source:HGNC Symbol;Acc:29223]	chr4	125585207	125633887
ANXA2	annexin A2 [Source:HGNC Symbol;Acc:537]	chr15	60639333	60695082
ANXA4	annexin A4 [Source:HGNC Symbol;Acc:542]	chr2	69947923	70053596
AP1B1	adaptor-related protein complex 1, beta 1 subunit [Source:HGNC Symbol;Acc:554]	chr22	29723669	29819168
AP1S3	adaptor-related protein complex 1, sigma 3 subunit [Source:HGNC Symbol;Acc:18971]	chr2	224616403	224702744
AP3M1	adaptor-related protein complex 3, mu 1 subunit [Source:HGNC Symbol;Acc:569]	chr10	75881524	75910821
APLP2	amyloid beta (A4) precursor-like protein 2 [Source:HGNC Symbol;Acc:598]	chr11	129939732	130014699
APP	amyloid beta (A4) precursor protein [Source:HGNC Symbol;Acc:620]	chr21	27252861	27543446
ARHGAP12	Rho GTPase activating protein 12 [Source:HGNC Symbol;Acc:16348]	chr10	32094365	32222742
ARHGAP21	Rho GTPase activating protein 21 [Source:HGNC Symbol;Acc:23725]	chr10	24872538	25017597
ARHGEF37	Rho guanine nucleotide exchange factor (GEF) 37 [Source:HGNC Symbol;Acc:34430]	chr5	148926510	149014531
ARNT	aryl hydrocarbon receptor nuclear translocator [Source:HGNC Symbol;Acc:700]	chr1	150782181	150849244
ARNTL	aryl hydrocarbon receptor nuclear translocator-like [Source:HGNC Symbol;Acc:701]	chr11	13298199	13408813
ARPC2	actin related protein 2/3 complex, subunit 2, 34kDa [Source:HGNC Symbol;Acc:705]	chr2	219081817	219119079
ARRDC3	arrestin domain containing 3 [Source:HGNC Symbol;Acc:29263]	chr5	90664541	90679176
ASAP3	ArfGAP with SH3 domain, ankyrin repeat and PH domain 3 [Source:HGNC Symbol;Acc:14987]	chr1	23755056	23815685
ASCC1	activating signal cointegrator 1 complex subunit 1 [Source:HGNC Symbol;Acc:24268]	chr10	73856278	73976892
ATG3	autophagy related 3 [Source:HGNC Symbol;Acc:20962]	chr3	112251356	112280893
ATL3	atlastin GTPase 3 [Source:HGNC Symbol;Acc:24526]	chr11	63391559	63439393
ATP6V1G2-DDX39B	ATP6V1G2-DDX39B readthrough (non-protein coding) [Source:HGNC Symbol;Acc:41999]	chr6	31497996	31514385
ATP8B5P	ATPase, class I, type 8B, member 5, pseudogene [Source:HGNC Symbol;Acc:27245]	chr9	35406752	35483026
BCL2L1	BCL2-like 1 [Source:HGNC Symbol;Acc:992]	chr20	30252255	30311792
BCL2L13	BCL2-like 13 (apoptosis facilitator) [Source:HGNC Symbol;Acc:17164]	chr22	18111621	18213388
BCORP1	BCL6 corepressor pseudogene 1 [Source:HGNC Symbol;Acc:23953]	chrY	21617317	21665039
BDNF	brain-derived neurotrophic factor [Source:HGNC Symbol;Acc:1033]	chr11	27676440	27743605
BEND7	BEN domain containing 7 [Source:HGNC Symbol;Acc:23514]	chr10	13480484	13575974
BFSP1	beaded filament structural protein 1, filensin [Source:HGNC Symbol;Acc:1040]	chr20	17474550	17549865
BICD2	bicaudal D homolog 2 (Drosophila) [Source:HGNC Symbol;Acc:17208]	chr9	95473645	95527094
BTBD10	BTB (POZ) domain containing 10 [Source:HGNC Symbol;Acc:21445]	chr11	13409548	13484844
BZW2	basic leucine zipper and W2 domains 2 [Source:HGNC Symbol;Acc:18808]	chr7	16685756	16746148
C10orf129	chromosome 10 open reading frame 129 [Source:HGNC Symbol;Acc:31665]	chr10	96953957	96988685
C11orf80	chromosome 11 open reading frame 80 [Source:HGNC Symbol;Acc:26197]	chr11	66511922	66610987
C12orf45	chromosome 12 open reading frame 45 [Source:HGNC Symbol;Acc:28628]	chr12	105380088	105443515
C14orf182	chromosome 14 open reading frame 182 [Source:HGNC Symbol;Acc:27503]	chr14	50448430	50474238
C1orf198	chromosome 1 open reading frame 198 [Source:HGNC Symbol;Acc:25900]	chr1	230972865	231005335
C20orf96	chromosome 20 open reading frame 96 [Source:HGNC Symbol;Acc:16227]	chr20	251504	276390
C2CD3	C2 calcium-dependent domain containing 3 [Source:HGNC Symbol;Acc:24564]	chr11	73723763	73887255
C3orf67	chromosome 3 open reading frame 67 [Source:HGNC Symbol;Acc:24763]	chr3	58703092	59040810
C4orf45	chromosome 4 open reading frame 45 [Source:HGNC Symbol;Acc:26342]	chr4	159814286	159959912
C5orf42	chromosome 5 open reading frame 42 [Source:HGNC Symbol;Acc:25801]	chr5	37106330	37254530
C6orf183	chromosome 6 open reading frame 183 [Source:HGNC Symbol;Acc:21562]	chr6	109487036	109592217
C7orf49	chromosome 7 open reading frame 49 [Source:HGNC Symbol;Acc:22432]	chr7	134777115	134855547
C7orf55-LUC7L2	C7orf55-LUC7L2 readthrough [Source:HGNC Symbol;Acc:44671]	chr7	139025105	139108198
C7orf63	chromosome 7 open reading frame 63 [Source:HGNC Symbol;Acc:26107]	chr7	89874488	89940377
C9orf3	chromosome 9 open reading frame 3 [Source:HGNC Symbol;Acc:1361]	chr9	97488983	97849441
CACNB4	calcium channel, voltage-dependent, beta 4 subunit [Source:HGNC Symbol;Acc:1404]	chr2	152689290	152960593
CAMK2D	calcium/calmodulin-dependent protein kinase II delta [Source:HGNC Symbol;Acc:1462]	chr4	114372188	114683083
CAV1	caveolin 1, caveolae protein, 22kDa [Source:HGNC Symbol;Acc:1527]	chr7	116159839	116201233
CCAT1	colon cancer associated transcript 1 (non-protein coding) [Source:HGNC Symbol;Acc:45128]	chr8	128220111	128231333
CCND3	cyclin D3 [Source:HGNC Symbol;Acc:1585]	chr6	41902671	42018095
CD8B	CD8b molecule [Source:HGNC Symbol;Acc:1707]	chr2	87042462	87094047
CD96	CD96 molecule [Source:HGNC Symbol;Acc:16892]	chr3	111006566	111384597
CDC42EP3	CDC42 effector protein (Rho GTPase binding) 3 [Source:HGNC Symbol;Acc:16943]	chr2	37869032	37965611
CDKN2C	cyclin-dependent kinase inhibitor 2C (p18, inhibits CDK4) [Source:HGNC Symbol;Acc:1789]	chr1	51426417	51440305
CDX2	caudal type homeobox 2 [Source:HGNC Symbol;Acc:1806]	chr13	28536274	28545276
CELF4	CUGBP, Elav-like family member 4 [Source:HGNC Symbol;Acc:14015]	chr18	34823010	35146000
CEP55	centrosomal protein 55kDa [Source:HGNC Symbol;Acc:1161]	chr10	95256389	95288849
CHM	choroideremia (Rab escort protein 1) [Source:HGNC Symbol;Acc:1940]	chrX	85116185	85307566
CIRH1A	cirrhosis, autosomal recessive 1A (cirhin) [Source:HGNC Symbol;Acc:1983]	chr16	69165194	69202941

table S3. Genes with significant transposon integrations in both RKO *BRAF*$^{wt/-/-}$ and *DLD-1 KRAS*$^{wt/-}$ libraries

Gene ID	description	Chrom	Start	End
CKS2	CDC28 protein kinase regulatory subunit 2 [Source:HGNC Symbol;Acc:2000]	chr9	91926113	91931618
CLIP2	CAP-GLY domain containing linker protein 2 [Source:HGNC Symbol;Acc:2586]	chr7	73698805	73820273
CMTM8	CKLF-like MARVEL transmembrane domain containing 8 [Source:HGNC Symbol;Acc:19179]	chr3	32280171	32411817
COA1	cytochrome c oxidase assembly factor 1 homolog (S. cerevisiae) [Source:HGNC Symbol;Acc:21868]	chr7	43648055	43769316
COL22A1	collagen, type XXII, alpha 1 [Source:HGNC Symbol;Acc:22989]	chr8	139600478	139931249
COL4A2	collagen, type IV, alpha 2 [Source:HGNC Symbol;Acc:2203]	chr13	110953159	111165374
COMMD7	COMM domain containing 7 [Source:HGNC Symbol;Acc:16223]	chr20	31290493	31331814
COPS5	COP9 signalosome subunit 5 [Source:HGNC Symbol;Acc:2240]	chr8	67955314	67996018
CPNE3	copine III [Source:HGNC Symbol;Acc:2316]	chr8	87497059	87573726
CPOX	coproporphyrinogen oxidase [Source:HGNC Symbol;Acc:2321]	chr3	98239976	98312567
CR1L	complement component (3b/4b) receptor 1-like [Source:HGNC Symbol;Acc:2335]	chr1	207818458	207911761
CROCC	ciliary rootlet coiled-coil, rootletin [Source:HGNC Symbol;Acc:21299]	chr1	17066768	17299474
CSNK1E	casein kinase 1, epsilon [Source:HGNC Symbol;Acc:2453]	chr22	38686697	38794527
CUX1	cut-like homeobox 1 [Source:HGNC Symbol;Acc:2557]	chr7	101453959	101927249
CXXC5	CXXC finger protein 5 [Source:HGNC Symbol;Acc:26943]	chr5	139021884	139063467
CYB5B	cytochrome b5 type B (outer mitochondrial membrane) [Source:HGNC Symbol;Acc:24374]	chr16	69458428	69500169
CYTH3	cytohesin 3 [Source:HGNC Symbol;Acc:9504]	chr7	6201407	6312275
DCAF12	DDB1 and CUL4 associated factor 12 [Source:HGNC Symbol;Acc:19911]	chr9	34086385	34127397
DCP2	DCP2 decapping enzyme homolog (S. cerevisiae) [Source:HGNC Symbol;Acc:24452]	chr5	112312399	112356667
DDX39B	DEAD (Asp-Glu-Ala-Asp) box polypeptide 39B [Source:HGNC Symbol;Acc:13917]	chr6	31497996	31510225
DHRS7B	dehydrogenase/reductase (SDR family) member 7B [Source:HGNC Symbol;Acc:24547]	chr17	21021677	21096578
DHX16	DEAH (Asp-Glu-Ala-His) box polypeptide 16 [Source:HGNC Symbol;Acc:2739]	chr6	30620896	30640814
DIRC2	disrupted in renal carcinoma 2 [Source:HGNC Symbol;Acc:16628]	chr3	122508642	122599986
DIS3L2	DIS3 mitotic control homolog (S. cerevisiae)-like 2 [Source:HGNC Symbol;Acc:28648]	chr2	232825955	233209060
DLG1	discs, large homolog 1 (Drosophila) [Source:HGNC Symbol;Acc:2900]	chr3	196769431	197031171
DLGAP1	discs, large (Drosophila) homolog-associated protein 1 [Source:HGNC Symbol;Acc:2905]	chr18	3496030	4455335
DLGAP1-AS2	DLGAP1 antisense RNA 2 [Source:HGNC Symbol;Acc:28146]	chr18	3602998	3608334
DNAH7	dynein, axonemal, heavy chain 7 [Source:HGNC Symbol;Acc:18661]	chr2	196602427	196938536
DNAJB4	DnaJ (Hsp40) homolog, subfamily B, member 4 [Source:HGNC Symbol;Acc:14886]	chr1	78444859	78483648
DNAJB6	DnaJ (Hsp40) homolog, subfamily B, member 6 [Source:HGNC Symbol;Acc:14888]	chr7	157128075	157210133
DNAJC2	DnaJ (Hsp40) homolog, subfamily C, member 2 [Source:HGNC Symbol;Acc:13192]	chr7	102952921	102985320
DNAJC9	DnaJ (Hsp40) homolog, subfamily C, member 9 [Source:HGNC Symbol;Acc:19123]	chr10	74943120	75008620
DOCK11	dedicator of cytokinesis 11 [Source:HGNC Symbol;Acc:23483]	chrX	117629861	117820126
DSCR3	Down syndrome critical region gene 3 [Source:HGNC Symbol;Acc:3044]	chr21	38595721	38640262
DYNC1I1	dynein, cytoplasmic 1, intermediate chain 1 [Source:HGNC Symbol;Acc:2963]	chr7	95401866	95739634
EEF1A1	eukaryotic translation elongation factor 1 alpha 1 [Source:HGNC Symbol;Acc:3189]	chr6	74225473	74233520
EEFSEC	eukaryotic elongation factor, selenocysteine-tRNA-specific [Source:HGNC Symbol;Acc:24614]	chr3	127867297	128127485
EFHC1	EF-hand domain (C-terminal) containing 1 [Source:HGNC Symbol;Acc:16406]	chr6	52285106	52387892
EIF3A	eukaryotic translation initiation factor 3, subunit A [Source:HGNC Symbol;Acc:3271]	chr10	120794356	120840396
EIF4A1	eukaryotic translation initiation factor 4A1 [Source:HGNC Symbol;Acc:3282]	chr17	7476024	7482323
EIF4A2	eukaryotic translation initiation factor 4A2 [Source:HGNC Symbol;Acc:3284]	chr3	186500994	186507689
ELK3	ELK3, ETS-domain protein (SRF accessory protein 2) [Source:HGNC Symbol;Acc:3325]	chr12	96588160	96663613
EMP1	epithelial membrane protein 1 [Source:HGNC Symbol;Acc:3333]	chr12	13349650	13369708
ENTPD7	ectonucleoside triphosphate diphosphohydrolase 7 [Source:HGNC Symbol;Acc:19745]	chr10	101419263	101465997
EPB41L2	erythrocyte membrane protein band 4.1-like 2 [Source:HGNC Symbol;Acc:3379]	chr6	131160487	131384462
EPT1	ethanolaminephosphotransferase 1 (CDP-ethanolamine-specific) [Source:HGNC Symbol;Acc:29361]	chr2	26531415	26618759
ERGIC2	ERGIC and golgi 2 [Source:HGNC Symbol;Acc:30208]	chr12	29490285	29534122
ERLEC1	endoplasmic reticulum lectin 1 [Source:HGNC Symbol;Acc:25222]	chr2	54014181	54045956
ERRFI1	ERBB receptor feedback inhibitor 1 [Source:HGNC Symbol;Acc:18185]	chr1	8064464	8086368
ESCO2	establishment of cohesion 1 homolog 2 (S. cerevisiae) [Source:HGNC Symbol;Acc:27230]	chr8	27629466	27670157
EVA1C	eva-1 homolog C (C. elegans) [Source:HGNC Symbol;Acc:13239]	chr21	33784314	33887707
EXOC5	exocyst complex component 5 [Source:HGNC Symbol;Acc:10696]	chr14	57670518	57735726
EXOSC8	exosome component 8 [Source:HGNC Symbol;Acc:17035]	chr13	37572953	37583750
EYA4	eyes absent homolog 4 (Drosophila) [Source:HGNC Symbol;Acc:3522]	chr6	133556736	133853258
FAM114A1	family with sequence similarity 114, member A1 [Source:HGNC Symbol;Acc:25087]	chr4	38869298	38947360
FAM115A	family with sequence similarity 115, member A [Source:HGNC Symbol;Acc:22201]	chr7	143548468	143599291
FAM149B1	family with sequence similarity 149, member B1 [Source:HGNC Symbol;Acc:29162]	chr10	74927924	75004262
FAM178A	family with sequence similarity 178, member A [Source:HGNC Symbol;Acc:17814]	chr10	102672720	102724893
FAM193A	family with sequence similarity 193, member A [Source:HGNC Symbol;Acc:16822]	chr4	2626988	2734292
FAM49B	family with sequence similarity 49, member B [Source:HGNC Symbol;Acc:25216]	chr8	130851839	131034375
FAM5B	family with sequence similarity 5, member B [Source:HGNC Symbol;Acc:13746]	chr1	177140633	177251558
FAT4	FAT tumor suppressor homolog 4 (Drosophila) [Source:HGNC Symbol;Acc:23109]	chr4	126237554	126414087
FBXL5	F-box and leucine-rich repeat protein 5 [Source:HGNC Symbol;Acc:13602]	chr4	15606162	15683302
FBXO34	F-box protein 34 [Source:HGNC Symbol;Acc:20201]	chr14	55738021	55828636
FBXO42	F-box protein 42 [Source:HGNC Symbol;Acc:29249]	chr1	16573334	16678949
FHL2	four and a half LIM domains 2 [Source:HGNC Symbol;Acc:3703]	chr2	105974169	106054960
FOXO3	forkhead box O3 [Source:HGNC Symbol;Acc:3821]	chr6	108876038	109005977
FPR1	formyl peptide receptor 1 [Source:HGNC Symbol;Acc:3826]	chr19	52248425	52312363
FREM3	FRAS1 related extracellular matrix 3 [Source:HGNC Symbol;Acc:25172]	chr4	144498455	144621828
FRMD5	FERM domain containing 5 [Source:HGNC Symbol;Acc:28214]	chr15	44162962	44492450
FRYL	FRY-like [Source:HGNC Symbol;Acc:29127]	chr4	48499378	48782339
FTSJD2	FtsJ methyltransferase domain containing 2 [Source:HGNC Symbol;Acc:21077]	chr6	37400995	37450603
GABPB1	GA binding protein transcription factor, beta subunit 1 [Source:HGNC Symbol;Acc:4074]	chr15	50569389	50647605
GAD1	glutamate decarboxylase 1 (brain, 67kDa) [Source:HGNC Symbol;Acc:4092]	chr2	171669723	171717661
GAPVD1	GTPase activating protein and VPS9 domains 1 [Source:HGNC Symbol;Acc:23375]	chr9	128024073	128129486
GARNL3	GTPase activating Rap/RanGAP domain-like 3 [Source:HGNC Symbol;Acc:25425]	chr9	129981544	130155939
GEMIN6	gem (nuclear organelle) associated protein 6 [Source:HGNC Symbol;Acc:20044]	chr2	38973676	39009598
GIPC2	GIPC PDZ domain containing family, member 2 [Source:HGNC Symbol;Acc:18177]	chr1	78445226	78604133
GNAI3	guanine nucleotide binding protein (G protein), alpha inhibiting activity polypeptide 3 [Source:HGNC Symb	chr1	110086233	110136975
GPR113	G protein-coupled receptor 113 [Source:HGNC Symbol;Acc:18989]	chr2	26531041	26569685
GPSM2	G-protein signaling modulator 2 [Source:HGNC Symbol;Acc:29501]	chr1	109417972	109473044
GRB10	growth factor receptor-bound protein 10 [Source:HGNC Symbol;Acc:4564]	chr7	50657760	50866159
GSN	gelsolin [Source:HGNC Symbol;Acc:4620]	chr9	123970078	124095121
GTF2IRD2	GTF2I repeat domain containing 2 [Source:HGNC Symbol;Acc:30775]	chr7	74210483	74267847
GXYLT2	glucoside xylosyltransferase 2 [Source:HGNC Symbol;Acc:33383]	chr3	72937224	73047289
HEG1	heart development protein with EGF-like domains 1 [Source:HGNC Symbol;Acc:29227]	chr3	124684554	124779802
HERC5	HECT and RLD domain containing E3 ubiquitin protein ligase 5 [Source:HGNC Symbol;Acc:24368]	chr4	89378268	89427314
HERPUD2	HERPUD family member 2 [Source:HGNC Symbol;Acc:21915]	chr7	35672269	35735181
HIPK2	homeodomain interacting protein kinase 2 [Source:HGNC Symbol;Acc:14402]	chr7	139246316	139482577
HIPK3	homeodomain interacting protein kinase 3 [Source:HGNC Symbol;Acc:4915]	chr11	33278218	33378569

table S3. Genes with significant transposon integrations in both RKO *BRAF*$^{wt/-/-}$ and *DLD-1 KRAS*$^{wt/-}$ libraries

Gene ID	description	Chrom	Start	End
HIST1H1C	histone cluster 1, H1c [Source:HGNC Symbol;Acc:4716]	chr6	26055968	26061699
HLA-AS1	HLA antisense RNA 1 [Source:HGNC Symbol;Acc:42509]	chr1	221006105	221053482
HMG20A	high mobility group 20A [Source:HGNC Symbol;Acc:5001]	chr15	77712754	77777949
HMGA2	high mobility group AT-hook 2 [Source:HGNC Symbol;Acc:5009]	chr12	66217911	66360075
HMGB1	high mobility group box 1 [Source:HGNC Symbol;Acc:4983]	chr13	31032884	31196734
HNRNPA3	heterogeneous nuclear ribonucleoprotein A3 [Source:HGNC Symbol;Acc:24941]	chr2	178077291	178088686
HOOK2	hook homolog 2 (Drosophila) [Source:HGNC Symbol;Acc:19885]	chr19	12873817	12983554
HPSE	heparanase [Source:HGNC Symbol;Acc:5164]	chr4	84213614	84261306
HSDL2	hydroxysteroid dehydrogenase like 2 [Source:HGNC Symbol;Acc:18572]	chr9	115142217	115234690
HSPA4L	heat shock 70kDa protein 4-like [Source:HGNC Symbol;Acc:17041]	chr4	128702976	128755226
HUNK	hormonally up-regulated Neu-associated kinase [Source:HGNC Symbol;Acc:13326]	chr21	33245628	33416946
HUS1	HUS1 checkpoint homolog (S. pombe) [Source:HGNC Symbol;Acc:5309]	chr7	47735328	48024178
IAH1	isoamyl acetate-hydrolyzing esterase 1 homolog (S. cerevisiae) [Source:HGNC Symbol;Acc:27696]	chr2	9608787	9636672
IARS	isoleucyl-tRNA synthetase [Source:HGNC Symbol;Acc:5330]	chr9	94972489	95056038
IARS2	isoleucyl-tRNA synthetase 2, mitochondrial [Source:HGNC Symbol;Acc:29685]	chr1	220267444	220321380
ICK	intestinal cell (MAK-like) kinase [Source:HGNC Symbol;Acc:21219]	chr6	52866077	52926600
IL13RA1	interleukin 13 receptor, alpha 1 [Source:HGNC Symbol;Acc:5974]	chrX	117861535	117928502
ING3	inhibitor of growth family, member 3 [Source:HGNC Symbol;Acc:14587]	chr7	120590803	120617270
INMT-FAM188B	INMT-FAM188B readthrough (non-protein coding) [Source:HGNC Symbol;Acc:41995]	chr7	30791753	30931696
INTU	inturned planar cell polarity protein [Source:HGNC Symbol;Acc:29239]	chr4	128544426	128637930
IPO5	importin 5 [Source:HGNC Symbol;Acc:6402]	chr13	98605912	98676551
IQCB1	IQ motif containing B1 [Source:HGNC Symbol;Acc:28949]	chr3	121488610	121553926
IQCH	IQ motif containing H [Source:HGNC Symbol;Acc:25721]	chr15	67542138	67794598
ISM1	isthmin 1, angiogenesis inhibitor [Source:HGNC Symbol;Acc:16213]	chr20	13197418	13281298
IWS1	IWS1 homolog (S. cerevisiae) [Source:HGNC Symbol;Acc:25467]	chr2	128193783	128284462
JAK1	Janus kinase 1 [Source:HGNC Symbol;Acc:6190]	chr1	65298912	65432187
**JUN**	jun proto-oncogene [Source:HGNC Symbol;Acc:6204]	chr1	59246465	59249785
KANK4	KN motif and ankyrin repeat domains 4 [Source:HGNC Symbol;Acc:27263]	chr1	62702651	62790085
KCTD1	potassium channel tetramerization domain containing 1 [Source:HGNC Symbol;Acc:18249]	chr18	24034874	24242365
KIAA0922	KIAA0922 [Source:HGNC Symbol;Acc:29146]	chr4	154387498	154557863
KIAA1324	KIAA1324 [Source:HGNC Symbol;Acc:29618]	chr1	109656301	109749401
KIAA1468	KIAA1468 [Source:HGNC Symbol;Acc:29289]	chr18	59854491	59974355
KIAA1549L	KIAA1549-like [Source:HGNC Symbol;Acc:24836]	chr11	33563618	33695648
KIF13A	kinesin family member 13A [Source:HGNC Symbol;Acc:14566]	chr6	17759414	17987854
KIFC3	kinesin family member C3 [Source:HGNC Symbol;Acc:6326]	chr16	57792129	57896957
KITLG	KIT ligand [Source:HGNC Symbol;Acc:6343]	chr12	88886570	88974628
LACTB2	lactamase, beta 2 [Source:HGNC Symbol;Acc:18512]	chr8	71547553	71586409
LAMTOR5-AS1	LAMTOR5 antisense RNA 1 [Source:HGNC Symbol;Acc:40823]	chr1	110925441	110974982
LCOR	ligand dependent nuclear receptor corepressor [Source:HGNC Symbol;Acc:29503]	chr10	98592017	98740800
LGALS8	lectin, galactoside-binding, soluble, 8 [Source:HGNC Symbol;Acc:6569]	chr1	236676300	236716281
LHFPL2	lipoma HMGIC fusion partner-like 2 [Source:HGNC Symbol;Acc:6588]	chr5	77781038	78065844
LIMCH1	LIM and calponin homology domains 1 [Source:HGNC Symbol;Acc:29191]	chr4	41361624	41702061
LINC00159	long intergenic non-protein coding RNA 159 [Source:HGNC Symbol;Acc:1285]	chr21	33452629	33570125
LINC00472	long intergenic non-protein coding RNA 472 [Source:HGNC Symbol;Acc:21380]	chr6	72054047	72130472
LINC00525	long intergenic non-protein coding RNA 525 [Source:HGNC Symbol;Acc:40290]	chr7	47796074	47806370
LIPA	lipase A, lysosomal acid, cholesterol esterase [Source:HGNC Symbol;Acc:6617]	chr10	90973326	91174314
LNPEP	leucyl/cystinyl aminopeptidase [Source:HGNC Symbol;Acc:6656]	chr5	96271098	96373219
LPP	LIM domain containing preferred translocation partner in lipoma [Source:HGNC Symbol;Acc:6679]	chr3	187871072	188608460
LRCH3	leucine-rich repeats and calponin homology (CH) domain containing 3 [Source:HGNC Symbol;Acc:28637]	chr3	197518097	197615307
LRIG1	leucine-rich repeats and immunoglobulin-like domains 1 [Source:HGNC Symbol;Acc:17360]	chr3	66429221	66551687
LRRC37A16P	leucine rich repeat containing 37, member A16, pseudogene [Source:HGNC Symbol;Acc:43820]	chr17	66121918	66148609
LRRK1	leucine-rich repeat kinase 1 [Source:HGNC Symbol;Acc:18608]	chr15	101459420	101610317
LUC7L3	LUC7-like 3 (S. cerevisiae) [Source:HGNC Symbol;Acc:24309]	chr17	48796905	48833574
LURAP1L	leucine rich adaptor protein 1-like [Source:HGNC Symbol;Acc:31452]	chr9	12775020	12822130
LYPLAL1	lysophospholipase-like 1 [Source:HGNC Symbol;Acc:20440]	chr1	219347186	219386207
LYSMD2	LysM, putative peptidoglycan-binding, domain containing 2 [Source:HGNC Symbol;Acc:28571]	chr15	52015208	52043782
MALAT1	metastasis associated lung adenocarcinoma transcript 1 (non-protein coding) [Source:HGNC Symbol;Acc:	chr11	65265233	65273940
MALT1	mucosa associated lymphoid tissue lymphoma translocation gene 1 [Source:HGNC Symbol;Acc:6819]	chr18	56338618	56417371
MAP3K1	mitogen-activated protein kinase kinase kinase 1, E3 ubiquitin protein ligase [Source:HGNC Symbol;Acc:6	chr5	56111401	56191979
MAP3K7	mitogen-activated protein kinase kinase kinase 7 [Source:HGNC Symbol;Acc:6859]	chr6	91223292	91296764
MAP3K9	mitogen-activated protein kinase kinase kinase 9 [Source:HGNC Symbol;Acc:6861]	chr14	71189243	71281251
MAP4K3	mitogen-activated protein kinase kinase kinase kinase 3 [Source:HGNC Symbol;Acc:6865]	chr2	39476407	39664453
MAPK9	mitogen-activated protein kinase 9 [Source:HGNC Symbol;Acc:6886]	chr5	179660143	179724099
MAPKAPK2	mitogen-activated protein kinase-activated protein kinase 2 [Source:HGNC Symbol;Acc:6887]	chr1	206858289	206907628
MATR3	matrin 3 [Source:HGNC Symbol;Acc:6912]	chr5	138609441	138667360
MB21D2	Mab-21 domain containing 2 [Source:HGNC Symbol;Acc:30438]	chr3	192514604	192635950
MBNL1	muscleblind-like splicing regulator 1 [Source:HGNC Symbol;Acc:6923]	chr3	151961617	152183569
MCC	mutated in colorectal cancers [Source:HGNC Symbol;Acc:6935]	chr5	112357796	112829527
MDM1	Mdm1 nuclear protein homolog (mouse) [Source:HGNC Symbol;Acc:29917]	chr12	68666223	68726161
MET	met proto-oncogene (hepatocyte growth factor receptor) [Source:HGNC Symbol;Acc:7029]	chr7	116312444	116438440
METAP1	methionyl aminopeptidase 1 [Source:HGNC Symbol;Acc:15789]	chr4	99916771	99983964
MFHAS1	malignant fibrous histiocytoma amplified sequence 1 [Source:HGNC Symbol;Acc:16982]	chr8	8640864	8751155
MFSD1	major facilitator superfamily domain containing 1 [Source:HGNC Symbol;Acc:25874]	chr3	158449987	158547508
MIR31HG	MIR31 host gene (non-protein coding) [Source:HGNC Symbol;Acc:37187]	chr9	21455641	21559668
MKKS	McKusick-Kaufman syndrome [Source:HGNC Symbol;Acc:7108]	chr20	10385832	10414870
MKX	mohawk homeobox [Source:HGNC Symbol;Acc:23729]	chr10	27961804	28039989
MORF4L1	mortality factor 4 like 1 [Source:HGNC Symbol;Acc:16989]	chr15	79102829	79190475
MPP7	membrane protein, palmitoylated 7 (MAGUK p55 subfamily member 7) [Source:HGNC Symbol;Acc:26542	chr10	28339922	28628415
MRVI1	murine retrovirus integration site 1 homolog [Source:HGNC Symbol;Acc:7237]	chr11	10594638	10720535
MSRB3	methionine sulfoxide reductase B3 [Source:HGNC Symbol;Acc:27375]	chr12	65672423	65882024
MXI1	MAX interactor 1, dimerization protein [Source:HGNC Symbol;Acc:7534]	chr10	111967363	112047123
MYO16-AS1	MYO16 antisense RNA 1 [Source:HGNC Symbol;Acc:39913]	chr13	109816250	109858831
MYO1B	myosin IB [Source:HGNC Symbol;Acc:7596]	chr2	192109911	192290115
MYO5B	myosin VB [Source:HGNC Symbol;Acc:7603]	chr18	47349183	47726463
MYSM1	Myb-like, SWIRM and MPN domains 1 [Source:HGNC Symbol;Acc:29401]	chr1	59120411	59165764
NAA15	N(alpha)-acetyltransferase 15, NatA auxiliary subunit [Source:HGNC Symbol;Acc:30782]	chr4	140222609	140341187
NAA25	N(alpha)-acetyltransferase 25, NatB auxiliary subunit [Source:HGNC Symbol;Acc:25783]	chr12	112464500	112551826
NAA60	N(alpha)-acetyltransferase 60, NatF catalytic subunit [Source:HGNC Symbol;Acc:25875]	chr16	3493611	3536963
NCK1	NCK adaptor protein 1 [Source:HGNC Symbol;Acc:7664]	chr3	136581050	136668665
NCOA3	nuclear receptor coactivator 3 [Source:HGNC Symbol;Acc:7670]	chr20	46130601	46285621

table S3. Genes with significant transposon integrations in both RKO *BRAF*$^{wt/-/-}$ and *DLD-1 KRAS*$^{wt/-}$ libraries

Gene ID	description	Chrom	Start	End
NCOR2	nuclear receptor corepressor 2 [Source:HGNC Symbol;Acc:7673]	chr12	124808961	125052135
NDUFAF2	NADH dehydrogenase (ubiquinone) complex I, assembly factor 2 [Source:HGNC Symbol;Acc:28086]	chr5	60235956	60448853
NEDD4L	neural precursor cell expressed, developmentally down-regulated 4-like, E3 ubiquitin protein ligase [Sourc	chr18	55711599	56068772
NF2	neurofibromin 2 (merlin) [Source:HGNC Symbol;Acc:7773]	chr22	29999545	30094587
NKIRAS1	NFKB inhibitor interacting Ras-like 1 [Source:HGNC Symbol;Acc:17899]	chr3	23933151	23988082
NLRP11	NLR family, pyrin domain containing 11 [Source:HGNC Symbol;Acc:22945]	chr19	56296770	56348166
NMNAT1	nicotinamide nucleotide adenylyltransferase 1 [Source:HGNC Symbol;Acc:17877]	chr1	10003486	10045559
NOSTRIN	nitric oxide synthase trafficker [Source:HGNC Symbol;Acc:20203]	chr2	169643049	169722024
NOVA1-AS1	NOVA1 antisense RNA 1 (head to head) [Source:HGNC Symbol;Acc:19827]	chr14	27062618	27275673
NPAS2	neuronal PAS domain protein 2 [Source:HGNC Symbol;Acc:7895]	chr2	101431614	101613291
NPTN	neuroplastin [Source:HGNC Symbol;Acc:17867]	chr15	73852355	73926475
NR2C2	nuclear receptor subfamily 2, group C, member 2 [Source:HGNC Symbol;Acc:7972]	chr3	14989091	15095107
NR2F2-AS1	NR2F2 antisense RNA 1 [Source:HGNC Symbol;Acc:44222]	chr15	96670598	96875590
NSMAF	neutral sphingomyelinase (N-SMase) activation associated factor [Source:HGNC Symbol;Acc:8017]	chr8	59496063	59577403
NSMCE2	non-SMC element 2, MMS21 homolog (S. cerevisiae) [Source:HGNC Symbol;Acc:26513]	chr8	126103921	126379362
NUDT5	nudix (nucleoside diphosphate linked moiety X)-type motif 5 [Source:HGNC Symbol;Acc:8052]	chr10	12207324	12238143
NUP214	nucleoporin 214kDa [Source:HGNC Symbol;Acc:8064]	chr9	134000948	134110057
NUP35	nucleoporin 35kDa [Source:HGNC Symbol;Acc:29797]	chr2	183977241	184026408
NUP62CL	nucleoporin 62kDa C-terminal like [Source:HGNC Symbol;Acc:25960]	chrX	106366657	106449670
OSBPL6	oxysterol binding protein-like 6 [Source:HGNC Symbol;Acc:16388]	chr2	179059208	179264160
OSTCP1	oligosaccharyltransferase complex subunit pseudogene 1 [Source:HGNC Symbol;Acc:30530]	chr6	159262149	159278664
OXSR1	oxidative stress responsive 1 [Source:HGNC Symbol;Acc:8508]	chr3	38201580	38296979
PACSIN2	protein kinase C and casein kinase substrate in neurons 2 [Source:HGNC Symbol;Acc:8571]	chr22	43231418	43416151
PAK2	p21 protein (Cdc42/Rac)-activated kinase 2 [Source:HGNC Symbol;Acc:8591]	chr3	196466728	196559518
PARN	poly(A)-specific ribonuclease [Source:HGNC Symbol;Acc:8609]	chr16	14529558	14731585
PAWR	PRKC, apoptosis, WT1, regulator [Source:HGNC Symbol;Acc:8614]	chr12	79968759	80089877
PCBP1-AS1	PCBP1 antisense RNA 1 [Source:HGNC Symbol;Acc:42948]	chr2	70189395	70315978
PCDH7	protocadherin 7 [Source:HGNC Symbol;Acc:8659]	chr4	30722037	31148422
PCNXL4	pecanex-like 4 (Drosophila) [Source:HGNC Symbol;Acc:20349]	chr14	60558629	60635851
PDCD2	programmed cell death 2 [Source:HGNC Symbol;Acc:8762]	chr6	170884383	170893780
PDE12	phosphodiesterase 12 [Source:HGNC Symbol;Acc:25386]	chr3	57537003	57552571
PDE6D	phosphodiesterase 6D, cGMP-specific, rod, delta [Source:HGNC Symbol;Acc:8788]	chr2	232597135	232650982
PDP1	pyruvate dehyrogenase phosphatase catalytic subunit 1 [Source:HGNC Symbol;Acc:9279]	chr8	94870035	94938294
PDSS1	prenyl (decaprenyl) diphosphate synthase, subunit 1 [Source:HGNC Symbol;Acc:17759]	chr10	26986588	27035727
PELI1	pellino E3 ubiquitin protein ligase 1 [Source:HGNC Symbol;Acc:8827]	chr2	64319786	64371588
PER2	period circadian clock 2 [Source:HGNC Symbol;Acc:8846]	chr2	239152679	239198743
PFKP	phosphofructokinase, platelet [Source:HGNC Symbol;Acc:8878]	chr10	3104712	3179904
PHACTR4	phosphatase and actin regulator 4 [Source:HGNC Symbol;Acc:25793]	chr1	28696114	28826881
PHEX	phosphate regulating endopeptidase homolog, X-linked [Source:HGNC Symbol;Acc:8918]	chrX	22050559	22269427
PHLDB2	pleckstrin homology-like domain, family B, member 2 [Source:HGNC Symbol;Acc:29573]	chr3	111451344	111695364
PI4K2B	phosphatidylinositol 4-kinase type 2 beta [Source:HGNC Symbol;Acc:18215]	chr4	25162263	25280714
PIK3CB	phosphatidylinositol-4,5-bisphosphate 3-kinase, catalytic subunit beta [Source:HGNC Symbol;Acc:8976]	chr3	138372860	138558780
PISD	phosphatidylserine decarboxylase [Source:HGNC Symbol;Acc:8999]	chr22	32014477	32058418
PJA2	praja ring finger 2, E3 ubiquitin protein ligase [Source:HGNC Symbol;Acc:17481]	chr5	108670410	108745695
PKM	pyruvate kinase, muscle [Source:HGNC Symbol;Acc:9021]	chr15	72491370	72524164
PKP4	plakophilin 4 [Source:HGNC Symbol;Acc:9026]	chr2	159308476	159539391
PLAC1	placenta-specific 1 [Source:HGNC Symbol;Acc:9044]	chrX	133699868	133898352
PLAC1L	placenta-specific 1-like [Source:HGNC Symbol;Acc:26699]	chr11	59807748	59815517
PPP1R42	protein phosphatase 1, regulatory subunit 42 [Source:HGNC Symbol;Acc:33732]	chr8	67876334	67968839
PPP3CA	protein phosphatase 3, catalytic subunit, alpha isozyme [Source:HGNC Symbol;Acc:9314]	chr4	101944566	102269435
PPP4R1	protein phosphatase 4, regulatory subunit 1 [Source:HGNC Symbol;Acc:9320]	chr18	9546789	9615238
PRDX1	peroxiredoxin 1 [Source:HGNC Symbol;Acc:9352]	chr1	45976708	45988719
PREP	prolyl endopeptidase [Source:HGNC Symbol;Acc:9358]	chr6	105725440	105850959
PRIM2	primase, DNA, polypeptide 2 (58kDa) [Source:HGNC Symbol;Acc:9370]	chr6	57179603	57513375
PRRC1	proline-rich coiled-coil 1 [Source:HGNC Symbol;Acc:28164]	chr5	126853301	126890781
PRRG1	proline rich Gla (G-carboxyglutamic acid) 1 [Source:HGNC Symbol;Acc:9469]	chrX	37208528	37316548
PSMC6	proteasome (prosome, macropain) 26S subunit, ATPase, 6 [Source:HGNC Symbol;Acc:9553]	chr14	53173890	53195305
PTCHD1	patched domain containing 1 [Source:HGNC Symbol;Acc:26392]	chrX	23352133	23422489
PTPN1	protein tyrosine phosphatase, non-receptor type 1 [Source:HGNC Symbol;Acc:9642]	chr20	49126891	49201299
PVT1	Pvt1 oncogene (non-protein coding) [Source:HGNC Symbol;Acc:9709]	chr8	128806779	129113499
RAB10	RAB10, member RAS oncogene family [Source:HGNC Symbol;Acc:9759]	chr2	26256976	26360323
RAB11FIP2	RAB11 family interacting protein 2 (class I) [Source:HGNC Symbol;Acc:29152]	chr10	119764427	119806114
RAB5A	RAB5A, member RAS oncogene family [Source:HGNC Symbol;Acc:9783]	chr3	19988571	20026667
RAD23B	RAD23 homolog B (S. cerevisiae) [Source:HGNC Symbol;Acc:9813]	chr9	110045418	110094475
RAD52	RAD52 homolog (S. cerevisiae) [Source:HGNC Symbol;Acc:9824]	chr12	1021243	1100356
RAPGEF5	Rap guanine nucleotide exchange factor (GEF) 5 [Source:HGNC Symbol;Acc:16862]	chr7	22157856	22401763
RBM12B	RNA binding motif protein 12B [Source:HGNC Symbol;Acc:32310]	chr8	94741584	94753245
RBM14-RBM4	RBM14-RBM4 readthrough [Source:HGNC Symbol;Acc:38840]	chr11	66384097	66413940
RBM17	RNA binding motif protein 17 [Source:HGNC Symbol;Acc:16944]	chr10	6130950	6159420
RBM4	RNA binding motif protein 4 [Source:HGNC Symbol;Acc:9901]	chr11	66384097	66434153
RBM6	RNA binding motif protein 6 [Source:HGNC Symbol;Acc:9903]	chr3	49977440	50137478
RCOR3	REST corepressor 3 [Source:HGNC Symbol;Acc:25594]	chr1	211431719	211489727
RDX	radixin [Source:HGNC Symbol;Acc:9944]	chr11	110045605	110172447
RGS7	regulator of G-protein signaling 7 [Source:HGNC Symbol;Acc:10003]	chr1	240931554	241525530
RHOBTB3	Rho-related BTB domain containing 3 [Source:HGNC Symbol;Acc:18757]	chr5	95049226	95160087
RNF11	ring finger protein 11 [Source:HGNC Symbol;Acc:10056]	chr1	51701943	51739127
RNF150	ring finger protein 150 [Source:HGNC Symbol;Acc:23138]	chr4	141786725	142139031
RNF168	ring finger protein 168, E3 ubiquitin protein ligase [Source:HGNC Symbol;Acc:26661]	chr3	196195654	196230639
RNF217	ring finger protein 217 [Source:HGNC Symbol;Acc:21487]	chr6	125283691	125413779
ROCK2	Rho-associated, coiled-coil containing protein kinase 2 [Source:HGNC Symbol;Acc:10252]	chr2	11319887	11488456
ROR1	receptor tyrosine kinase-like orphan receptor 1 [Source:HGNC Symbol;Acc:10256]	chr1	64234693	64647181
RPL7	ribosomal protein L7 [Source:HGNC Symbol;Acc:10363]	chr8	74202506	74208024
RPSAP52	ribosomal protein SA pseudogene 52 [Source:HGNC Symbol;Acc:35752]	chr12	66151800	66225754
RTN3	reticulon 3 [Source:HGNC Symbol;Acc:10469]	chr11	63448918	63527363
S100PBP	S100P binding protein [Source:HGNC Symbol;Acc:25768]	chr1	33282368	33324476
SATB2	SATB homeobox 2 [Source:HGNC Symbol;Acc:21637]	chr2	200134223	200340989
SATB2-AS1	SATB2 antisense RNA 1 [Source:HGNC Symbol;Acc:26490]	chr2	200317423	200341658
SBSPON	somatomedin B and thrombospondin, type 1 domain containing [Source:HGNC Symbol;Acc:30362]	chr8	73976775	74041323
SCAF11	SR-related CTD-associated factor 11 [Source:HGNC Symbol;Acc:10784]	chr12	46312914	46385903
SEMA5A	sema domain, seven thrombospondin repeats (type 1 and type 1-like), transmembrane domain (TM) and s	chr5	9035138	9551187

table S3. Genes with significant transposon integrations in both RKO *BRAF*$^{wt/-/-}$ and *DLD-1 KRAS*$^{wt/-}$ libraries

Gene ID	description	Chrom	Start	End
SETD5	SET domain containing 5 [Source:HGNC Symbol;Acc:25566]	chr3	9439299	9520924
SETD5-AS1	SETD5 antisense RNA 1 [Source:HGNC Symbol;Acc:44478]	chr3	9391373	9440263
SETD9	SET domain containing 9 [Source:HGNC Symbol;Acc:28508]	chr5	56205087	56221359
SF3A3	splicing factor 3a, subunit 3, 60kDa [Source:HGNC Symbol;Acc:10767]	chr1	38422647	38461593
SF3B3	splicing factor 3b, subunit 3, 130kDa [Source:HGNC Symbol;Acc:10770]	chr16	70557691	70608820
SFR1	SWI5-dependent recombination repair 1 [Source:HGNC Symbol;Acc:29574]	chr10	105881816	105886143
SGCE	sarcoglycan, epsilon [Source:HGNC Symbol;Acc:10808]	chr7	94214542	94285521
SGPL1	sphingosine-1-phosphate lyase 1 [Source:HGNC Symbol;Acc:10817]	chr10	72575717	72640930
SH3BP5	SH3-domain binding protein 5 (BTK-associated) [Source:HGNC Symbol;Acc:10827]	chr3	15296360	15382875
SH3TC2	SH3 domain and tetratricopeptide repeats 2 [Source:HGNC Symbol;Acc:29427]	chr5	148303202	148442726
SHROOM2	shroom family member 2 [Source:HGNC Symbol;Acc:630]	chrX	9754496	9917483
SIAH1	siah E3 ubiquitin protein ligase 1 [Source:HGNC Symbol;Acc:10857]	chr16	48390275	48482313
SLC17A5	solute carrier family 17 (anion/sugar transporter), member 5 [Source:HGNC Symbol;Acc:10933]	chr6	74303102	74363878
SLC1A3	solute carrier family 1 (glial high affinity glutamate transporter), member 3 [Source:HGNC Symbol;Acc:109	chr5	36606457	36688436
SLC25A33	solute carrier family 25 (pyrimidine nucleotide carrier), member 33 [Source:HGNC Symbol;Acc:29681]	chr1	9599541	9642831
SLC28A1	solute carrier family 28 (concentrative nucleoside transporter), member 1 [Source:HGNC Symbol;Acc:110(	chr15	85422885	85518876
SLC35F3	solute carrier family 35, member F3 [Source:HGNC Symbol;Acc:23616]	chr1	234035679	234460262
SLC44A3	solute carrier family 44, member 3 [Source:HGNC Symbol;Acc:28689]	chr1	95285898	95360802
SLC9A4	solute carrier family 9, subfamily A (NHE4, cation proton antiporter 4), member 4 [Source:HGNC Symbol;A	chr2	103089762	103150431
SLFN5	schlafen family member 5 [Source:HGNC Symbol;Acc:28286]	chr17	33570055	33600674
SMAD2	SMAD family member 2 [Source:HGNC Symbol;Acc:6768]	chr18	45357922	45457515
SMAP2	small ArfGAP2 [Source:HGNC Symbol;Acc:25082]	chr1	40805522	40888998
SMARCE1	SWI/SNF related, matrix associated, actin dependent regulator of chromatin, subfamily e, member 1 [Sour	chr17	38781214	38804760
SMEK2	SMEK homolog 2, suppressor of mek1 (Dictyostelium) [Source:HGNC Symbol;Acc:29267]	chr2	55774428	55846015
SMYD4	SET and MYND domain containing 4 [Source:HGNC Symbol;Acc:21067]	chr17	1682779	1733928
SNRNP200	small nuclear ribonucleoprotein 200kDa (U5) [Source:HGNC Symbol;Acc:30859]	chr2	96940074	96971297
SOD2	superoxide dismutase 2, mitochondrial [Source:HGNC Symbol;Acc:11180]	chr6	160100096	160183561
SORL1	sortilin-related receptor, L(DLR class) A repeats containing [Source:HGNC Symbol;Acc:11185]	chr11	121317912	121504402
SPC25	SPC25, NDC80 kinetochore complex component, homolog (S. cerevisiae) [Source:HGNC Symbol;Acc:24(	chr2	169690642	169769881
SPEN	spen homolog, transcriptional regulator (Drosophila) [Source:HGNC Symbol;Acc:17575]	chr1	16174359	16266955
SPIRE1	spire homolog 1 (Drosophila) [Source:HGNC Symbol;Acc:30622]	chr18	12446511	12663133
SPTAN1	spectrin, alpha, non-erythrocytic 1 [Source:HGNC Symbol;Acc:11273]	chr9	131314866	131395941
SRSF11	serine/arginine-rich splicing factor 11 [Source:HGNC Symbol;Acc:10782]	chr1	70671365	70718735
SRSF3	serine/arginine-rich splicing factor 3 [Source:HGNC Symbol;Acc:10785]	chr6	36562145	36571209
ST3GAL6	ST3 beta-galactoside alpha-2,3-sialyltransferase 6 [Source:HGNC Symbol;Acc:18080]	chr3	98451080	98540045
STAG1	stromal antigen 1 [Source:HGNC Symbol;Acc:11354]	chr3	136055077	136471220
STARD4	StAR-related lipid transfer (START) domain containing 4 [Source:HGNC Symbol;Acc:18058]	chr5	110831731	110853288
STAU2	staufen double-stranded RNA binding protein 2 [Source:HGNC Symbol;Acc:11371]	chr8	74332604	74664943
STK24	serine/threonine kinase 24 [Source:HGNC Symbol;Acc:11403]	chr13	99102455	99230194
STX17	syntaxin 17 [Source:HGNC Symbol;Acc:11432]	chr9	102668915	102732618
SUSD1	sushi domain containing 1 [Source:HGNC Symbol;Acc:25413]	chr9	114803065	114937688
SYDE2	synapse defective 1, Rho GTPase, homolog 2 (C. elegans) [Source:HGNC Symbol;Acc:25841]	chr1	85622556	85666729
SYK	spleen tyrosine kinase [Source:HGNC Symbol;Acc:11491]	chr9	93559069	93660831
SYN3	synapsin III [Source:HGNC Symbol;Acc:11496]	chr22	32908539	33459358
SYNCRIP	synaptotagmin binding, cytoplasmic RNA interacting protein [Source:HGNC Symbol;Acc:16918]	chr6	86267696	86353510
SYNJ2	synaptojanin 2 [Source:HGNC Symbol;Acc:11504]	chr6	158397888	158520208
SYNJ2BP	synaptojanin 2 binding protein [Source:HGNC Symbol;Acc:18955]	chr14	70838148	70883778
SYNRG	synergin, gamma [Source:HGNC Symbol;Acc:557]	chr17	35874900	35969544
TBC1D2	TBC1 domain family, member 2 [Source:HGNC Symbol;Acc:18026]	chr9	100961311	101017915
TBL1X	transducin (beta)-like 1X-linked [Source:HGNC Symbol;Acc:11585]	chrX	9431335	9687780
TCEB1	transcription elongation factor B (SIII), polypeptide 1 (15kDa, elongin C) [Source:HGNC Symbol;Acc:1161	chr8	74851404	74884522
TCF7L2	transcription factor 7-like 2 (T-cell specific, HMG-box) [Source:HGNC Symbol;Acc:11641]	chr10	114710009	114927437
TCP1	t-complex 1 [Source:HGNC Symbol;Acc:11655]	chr6	160199530	160210781
TEAD1	TEA domain family member 1 (SV40 transcriptional enhancer factor) [Source:HGNC Symbol;Acc:11714]	chr11	12690969	12966298
TEC	tec protein tyrosine kinase [Source:HGNC Symbol;Acc:11719]	chr4	48137800	48271881
TES	testis derived transcript (3 LIM domains) [Source:HGNC Symbol;Acc:14620]	chr7	115850547	115898837
TFPI	tissue factor pathway inhibitor (lipoprotein-associated coagulation inhibitor) [Source:HGNC Symbol;Acc:11	chr2	188328957	188430487
THUMPD3	THUMP domain containing 3 [Source:HGNC Symbol;Acc:24493]	chr3	9404526	9428475
TIAM2	T-cell lymphoma invasion and metastasis 2 [Source:HGNC Symbol;Acc:11806]	chr6	155148831	155578857
TLK1	tousled-like kinase 1 [Source:HGNC Symbol;Acc:11841]	chr2	171847333	172087824
TMA16	translation machinery associated 16 homolog (S. cerevisiae) [Source:HGNC Symbol;Acc:25638]	chr4	164415594	164441691
TMCO1	transmembrane and coiled-coil domains 1 [Source:HGNC Symbol;Acc:18188]	chr1	165696032	165796992
TMCO3	transmembrane and coiled-coil domains 3 [Source:HGNC Symbol;Acc:20329]	chr13	114145310	114204542
TMED3	transmembrane emp24 protein transport domain containing 3 [Source:HGNC Symbol;Acc:28889]	chr15	79603404	79704334
TMEM51	transmembrane protein 51 [Source:HGNC Symbol;Acc:25488]	chr1	15479028	15546976
TMEM67	transmembrane protein 67 [Source:HGNC Symbol;Acc:28396]	chr8	94762072	94831462
TMLHE	trimethyllysine hydroxylase, epsilon [Source:HGNC Symbol;Acc:18308]	chrX	154719776	154899605
TMOD3	tropomodulin 3 (ubiquitous) [Source:HGNC Symbol;Acc:11873]	chr15	52121825	52239492
TMTC3	transmembrane and tetratricopeptide repeat containing 3 [Source:HGNC Symbol;Acc:26899]	chr12	88536073	88593664
TMX2-CTNND1	TMX2-CTNND1 readthrough (non-protein coding) [Source:HGNC Symbol;Acc:41992]	chr11	57480077	57559058
TNIK	TRAF2 and NCK interacting kinase [Source:HGNC Symbol;Acc:30765]	chr3	170779128	171183197
TNPO3	transportin 3 [Source:HGNC Symbol;Acc:17103]	chr7	128594948	128695198
TNRC18	trinucleotide repeat containing 18 [Source:HGNC Symbol;Acc:11962]	chr7	5346421	5465045
TNRC6A	trinucleotide repeat containing 6A [Source:HGNC Symbol;Acc:11969]	chr16	24736016	24838953
TOPBP1	topoisomerase (DNA) II binding protein 1 [Source:HGNC Symbol;Acc:17008]	chr3	133317019	133385737
TRAK1	trafficking protein, kinesin binding 1 [Source:HGNC Symbol;Acc:29947]	chr3	42055294	42267381
TRAM1	translocation associated membrane protein 1 [Source:HGNC Symbol;Acc:20568]	chr8	71485677	71525622
TRIM2	tripartite motif containing 2 [Source:HGNC Symbol;Acc:15974]	chr4	154068494	154260472
TTLL11-IT1	TTLL11 intronic transcript 1 (non-protein coding) [Source:HGNC Symbol;Acc:24214]	chr9	124646915	124725998
TTLL5	tubulin tyrosine ligase-like family, member 5 [Source:HGNC Symbol;Acc:19963]	chr14	76094968	76421421
TWIST1	twist basic helix-loop-helix transcription factor 1 [Source:HGNC Symbol;Acc:12428]	chr7	19060614	19157295
TXNRD1	thioredoxin reductase 1 [Source:HGNC Symbol;Acc:12437]	chr12	104609557	104744061
UBE2U	ubiquitin-conjugating enzyme E2U (putative) [Source:HGNC Symbol;Acc:28559]	chr1	64664310	64733053
UBE2V2	ubiquitin-conjugating enzyme E2 variant 2 [Source:HGNC Symbol;Acc:12495]	chr8	48920960	48977268
UBR5	ubiquitin protein ligase E3 component n-recognin 5 [Source:HGNC Symbol;Acc:16806]	chr8	103265240	103425069
UCA1	urothelial cancer associated 1 (non-protein coding) [Source:HGNC Symbol;Acc:37126]	chr19	15939757	15947130
UGGT2	UDP-glucose glycoprotein glucosyltransferase 2 [Source:HGNC Symbol;Acc:15664]	chr13	96453834	96710736
UNC50	unc-50 homolog (C. elegans) [Source:HGNC Symbol;Acc:16046]	chr2	99225042	99234978
UQCC	ubiquinol-cytochrome c reductase complex chaperone [Source:HGNC Symbol;Acc:15891]	chr20	33890369	33999944
USP34	ubiquitin specific peptidase 34 [Source:HGNC Symbol;Acc:20066]	chr2	61414598	61702904

table S3. Genes with significant transposon integrations in both RKO *BRAF*$^{wt/-/-}$ and *DLD-1 KRAS*$^{wt/-}$ libraries

Gene ID	description	Chrom	Start	End
VKORC1L1	vitamin K epoxide reductase complex, subunit 1-like 1 [Source:HGNC Symbol;Acc:21492]	chr7	65333254	65424550
VMP1	vacuole membrane protein 1 [Source:HGNC Symbol;Acc:29559]	chr17	57784553	57919616
VPS45	vacuolar protein sorting 45 homolog (S. cerevisiae) [Source:HGNC Symbol;Acc:14579]	chr1	150039369	150117505
WDR11	WD repeat domain 11 [Source:HGNC Symbol;Acc:13831]	chr10	122610687	122669036
WDR82	WD repeat domain 82 [Source:HGNC Symbol;Acc:28826]	chr3	52288437	52327036
WTAP	Wilms tumor 1 associated protein [Source:HGNC Symbol;Acc:16846]	chr6	160146617	160177351
XXYLT1	xyloside xylosyltransferase 1 [Source:HGNC Symbol;Acc:26639]	chr3	194789008	194996896
YARS	tyrosyl-tRNA synthetase [Source:HGNC Symbol;Acc:12840]	chr1	33240840	33283754
YBX1	Y box binding protein 1 [Source:HGNC Symbol;Acc:8014]	chr1	43148098	43168020
YTHDF3	YTH domain family, member 3 [Source:HGNC Symbol;Acc:26465]	chr8	64081112	64125346
ZBTB38	zinc finger and BTB domain containing 38 [Source:HGNC Symbol;Acc:26636]	chr3	141043055	141168634
ZBTB43	zinc finger and BTB domain containing 43 [Source:HGNC Symbol;Acc:17908]	chr9	129567285	129600489
ZBTB8OS	zinc finger and BTB domain containing 8 opposite strand [Source:HGNC Symbol;Acc:24094]	chr1	33065773	33121504
ZC3H6	zinc finger CCCH-type containing 6 [Source:HGNC Symbol;Acc:24762]	chr2	113033171	113097640
ZFAND1	zinc finger, AN1-type domain 1 [Source:HGNC Symbol;Acc:25858]	chr8	82613569	82645138
ZFHX3	zinc finger homeobox 3 [Source:HGNC Symbol;Acc:777]	chr16	72816784	73093597
ZMYND8	zinc finger, MYND-type containing 8 [Source:HGNC Symbol;Acc:9397]	chr20	45837859	45990567
ZNF106	zinc finger protein 106 [Source:HGNC Symbol;Acc:12886]	chr15	42705021	42783321
ZNF131	zinc finger protein 131 [Source:HGNC Symbol;Acc:12915]	chr5	43065278	43192123
ZNF182	zinc finger protein 182 [Source:HGNC Symbol;Acc:13001]	chrX	47834250	47863377
ZNF184	zinc finger protein 184 [Source:HGNC Symbol;Acc:12975]	chr6	27418522	27440897
ZNF217	zinc finger protein 217 [Source:HGNC Symbol;Acc:13009]	chr20	52183604	52226446
ZNF283	zinc finger protein 283 [Source:HGNC Symbol;Acc:13077]	chr19	44331444	44353307
ZNF410	zinc finger protein 410 [Source:HGNC Symbol;Acc:20144]	chr14	74348320	74399214
ZNF516	zinc finger protein 516 [Source:HGNC Symbol;Acc:28990]	chr18	74069644	74207146
ZNF573	zinc finger protein 573 [Source:HGNC Symbol;Acc:26420]	chr19	38226734	38307940
ZNF630	zinc finger protein 630 [Source:HGNC Symbol;Acc:28855]	chrX	47842756	47931025
ZNF713	zinc finger protein 713 [Source:HGNC Symbol;Acc:22043]	chr7	55950169	56009918
ZNF727	zinc finger protein 727 [Source:HGNC Symbol;Acc:22785]	chr7	63505821	63538927
ZNF775	zinc finger protein 775 [Source:HGNC Symbol;Acc:28501]	chr7	150065879	150109558
ZNF81	zinc finger protein 81 [Source:HGNC Symbol;Acc:13156]	chrX	47696301	47861960
ZPLD1	zona pellucida-like domain containing 1 [Source:HGNC Symbol;Acc:27022]	chr3	101818088	102198685
ZSWIM5	zinc finger, SWIM-type containing 5 [Source:HGNC Symbol;Acc:29299]	chr1	45482071	45776881

table S3. Genes with significant transposon integrations in both RKO BRAF$^{wt/-/-}$ and DLD-1 KRAS$^{wt/-}$ libraries after selection in low glucose. The transposon target genes in DLD-1 (Table S1) and RKO (Table S2) were intersected to obtain genes targeted in both genetic backgrounds.

table S4. KEGG pathway analysis of 483 common transposon targeted genes with significant integrations in DLD-1 *KRAS*$^{wt/-}$ and RKO *BRAF*$^{wt/-/-}$ libraries

KEGG ID	Pathway name	Genes in pathway	Genes found	*P*	Genes
hsa04310	Wnt signaling pathway	151	12	5.232E-05	CAMK2D CCND3 CSNK1E JUN MAP3K7 MAPK9 PPP3CA ROCK2 SIAH1 SMAD2 TBL1X TCF7L2
hsa04660	T cell receptor signaling pathway	108	11	8.424E-06	CD8B DLG1 JUN MALT1 MAP3K7 MAPK9 NCK1 PAK2 PIK3CB PPP3CA TEC
hsa04710	Circadian rhythm - mammal	23	4	1.826E-04	ARNTL CSNK1E NPAS2 PER2
hsa04380	Osteoclast differentiation	128	9	8.743E-04	FHL2 JAK1 JUN MAP3K7 MAPK9 PIK3CB PPP3CA SYK TEC
hsa03040	Spliceosome	128	9	8.743E-04	ACIN1 DDX39B DHX16 HNRNPA3 RBM17 SF3A3 SF3B3 SNRNP200 SRSF3
hsa05211	Renal cell carcinoma	70	6	1.343E-03	ARNT JUN MET PAK2 PIK3CB TCEB1
hsa04520	Adherens junction	73	6	1.714E-03	ACTN4 MAP3K7 MET PTPN1 SMAD2 TCF7L2
hsa00290	Valine, leucine and isoleucine biosynthesis	11	2	1.991E-03	IARS IARS2
hsa03013	RNA transport	152	9	3.095E-03	ACIN1 DDX39B EEF1A1 EIF3A EIF4A1 EIF4A2 GEMIN6 NUP214 NUP35
hsa04722	Neurotrophin signaling pathway	127	8	3.100E-03	BDNF CAMK2D FOXO3 JUN MAP3K1 MAPK9 MAPKAPK2 PIK3CB
hsa05210	Colorectal cancer	62	5	3.524E-03	JUN MAPK9 PIK3CB SMAD2 TCF7L2
hsa04012	ErbB signaling pathway	87	6	4.598E-03	CAMK2D JUN MAPK9 NCK1 PAK2 PIK3CB
hsa05120	Epithelial cell signaling in Helicobacter pylori infection	68	5	5.553E-03	ADAM10 ADAM17 JUN MAPK9 MET
hsa05212	Pancreatic cancer	70	5	6.388E-03	BCL2L1 JAK1 MAPK9 PIK3CB SMAD2
hsa04510	Focal adhesion	200	10	7.175E-03	ACTN4 CAV1 CCND3 COL4A2 JUN MAPK9 MET PAK2 PIK3CB ROCK2
hsa04144	Endocytosis	203	10	7.967E-03	ACAP2 ASAP3 CAV1 MET NEDD4L RAB11FIP2 RAB5A SMAD2 SMAP2 VPS45
hsa04971	Gastric acid secretion	74	5	8.322E-03	ADCY7 ADCY9 CAMK2D GNAI3 SLC9A4
hsa04662	B cell receptor signaling pathway	75	5	8.865E-03	JUN MALT1 PIK3CB PPP3CA SYK
hsa04912	GnRH signaling pathway	101	6	1.015E-02	ADCY7 ADCY9 CAMK2D JUN MAP3K1 MAPK9
hsa04916	Melanogenesis	101	6	1.015E-02	ADCY7 ADCY9 CAMK2D GNAI3 KITLG TCF7L2
hsa04360	Axon guidance	130	7	1.188E-02	GNAI3 MET NCK1 PAK2 PPP3CA ROCK2 SEMA5A
hsa00410	beta-Alanine metabolism	22	2	1.522E-02	ALDH2 GAD1
hsa04914	Progesterone-mediated oocyte maturation	87	5	1.746E-02	ADCY7 ADCY9 GNAI3 MAPK9 PIK3CB
hsa04962	Vasopressin-regulated water reabsorption	44	3	2.081E-02	ADCY9 DYNC1I1 RAB5A
hsa04142	Lysosome	121	6	2.479E-02	AGA AP1B1 AP1S3 AP3M1 LIPA SLC17A5
hsa04666	Fc gamma R-mediated phagocytosis	95	5	2.561E-02	ARPC2 ASAP3 GSN PIK3CB SYK
hsa05100	Bacterial invasion of epithelial cells	71	4	2.720E-02	ARPC2 CAV1 MET PIK3CB
hsa04930	Type II diabetes mellitus	48	3	2.766E-02	MAPK9 PIK3CB PKM
hsa05213	Endometrial cancer	52	3	3.569E-02	FOXO3 PIK3CB TCF7L2
hsa05142	Chagas disease (American trypanosomiasis)	104	5	3.741E-02	GNAI3 JUN MAPK9 PIK3CB SMAD2
hsa00230	Purine metabolism	162	7	3.750E-02	ADCY7 ADCY9 AK7 NUDT5 PDE6D PKM PRIM2
hsa05200	Pathways in cancer	327	12	3.756E-02	ARNT BCL2L1 COL4A2 JAK1 JUN KITLG MAPK9 MET PIK3CB SMAD2 TCEB1 TCF7L2
hsa05145	Toxoplasmosis	133	6	3.836E-02	BCL2L1 GNAI3 JAK1 MAP3K7 MAPK9 PIK3CB
hsa05014	Amyotrophic lateral sclerosis (ALS)	54	3	4.016E-02	BCL2L1 PPP3CA RAB5A
hsa04141	Protein processing in endoplasmic reticulum	168	7	4.471E-02	ERLEC1 HSPA4L MAPK9 MARCH6 RAD23B TRAM1 UGGT2
hsa04120	Ubiquitin mediated proteolysis	139	6	4.666E-02	MAP3K1 NEDD4L SIAH1 TCEB1 UBE2U UBR5
hsa04010	MAPK signaling pathway	268	10	4.683E-02	BDNF CACNB4 JUN MAP3K1 MAP3K7 MAP4K3 MAPK9 MAPKAPK2 PAK2 PPP3CA
hsa00562	Inositol phosphate metabolism	57	3	4.743E-02	PI4K2B PIK3CB SYNJ2

table S4. KEGG pathway analysis of 483 common transposon targeted genes with significant integrations in DLD-1 *KRAS*$^{wt/-}$ and RKO *BRAF*$^{wt/-/-}$ libraries. A KEGG pathway analysis of the 483 common transposon targeted genes with significantly high count of transposon integrations (table S3).

table S5a. Assignment of 163 common CIS genes in DLD-1 KRAS$^{wt/-}$ and RKO BRAF$^{wt/-/-}$ to colorectal cancer pathways

Gene symbol	Cancer gene	Pathway										Mutual exclusivity with KRAS mutation in TCGA COAD
		Wnt	EGFR/ Ras/MAPK	PI3K	TGFB	TP53	Hippo	Rap1	Cellular glucose metabolism	Other	KEGG Pathway	
ACAP2											hsa04144 Endocytosis	
ACER3											hsa00600 Sphingolipid metabolism	
ADK											hsa00230 Purine metabolism<br>hsa01100 Metabolic pathways	
AGAP1										Vesicle sorting 22453919	hsa04144 Endocytosis	
AP1S3											hsa04142 Lysosome	
APBB2												0.35
ARHGAP42												
ATP8A2												
BCAS3	12378525											
C14orf182												
C4orf45												0.13
C9orf3												
CAMK2D		KEGG	KEGG								hsa04012 ErbB signaling pathway<br>hsa04020 Calcium signaling pathway<br>hsa04066 HIF-1 signaling pathway<br>hsa04114 Oocyte meiosis<br>hsa04261 Adrenergic signaling in cardiomyocytes<br>hsa04310 Wnt signaling pathway<br>hsa04713 Circadian entrainment<br>hsa04720 Long-term potentiation<br>hsa04722 Neurotrophin signaling pathway<br>hsa04725 Cholinergic synapse<br>hsa04728 Dopaminergic synapse<br>hsa04740 Olfactory transduction<br>hsa04911 Insulin secretion<br>hsa04912 GnRH signaling pathway<br>hsa04916 Melanogenesis<br>hsa04971 Gastric acid secretion<br>hsa05031 Amphetamine addiction<br>hsa05152 Tuberculosis<br>hsa05205 Proteoglycans in cancer<br>hsa05214 Glioma	
CCSER1	23665203											
CDK14	22922871	20627573									hsa05202 Transcriptional misregulation in ca	0.277
CDKAL1									23840313			
CHST11			22317973		19937589						hsa00532 Glycosaminoglycan biosynthesis	0.22
CLSTN2												0.09
CNBD1												
CNTNAP2											hsa04514 Cell adhesion molecules (CAMs)	
COG5												
CPNE4												0.22
CSMD1	22367537<br>18614856											
CSMD3	24576404											
DAB1			12877983									
DGKB			19540930						20081858<br>20185807<br>17588539		hsa00561 Glycerolipid metabolism<br>hsa00564 Glycerophospholipid metabolism<br>hsa01100 Metabolic pathways<br>hsa04070 Phosphatidylinositol signaling system	
DIAPH3			22593025								hsa04810 Regulation of actin cytoskeleton	
DIP2B												
DIS3L2	22306653		15766527<br>24141620						21962509			
DLG1			16511562	16511562			KEGG				hsa04390 Hippo signaling pathway<br>hsa04660 T cell receptor signaling pathway<br>hsa05166 HTLV-I infection<br>hsa05203 Viral carcinogenesis	0.118
DLG2	24703847						KEGG				hsa04390 Hippo signaling pathway	
DLGAP1											hsa04724<br>Glutamatergic synapse	
DLGAP1-AS2												
DNAH7												0.045
DOCK4	19383911<br>12628187	18641688						19159611				

table S5a. Assignment of 163 common CIS genes in DLD-1 KRAS$^{wt/-}$ and RKO BRAF$^{wt/-/-}$ to colorectal cancer pathways

Gene symbol	Cancer gene	Pathway										Mutual exclusivity with KRAS mutation in TCGA COAD
		Wnt	EGFR/ Ras/MAPK	PI3K	TGFB	TP53	Hippo	Rap1	Cellular glucose metabolis m	Other	KEGG Pathway	
DST												
DTNA												
DYNC2H1											hsa04145 Phagosome<br>hsa04962 Vasopressin-regulated water reabsorption<br>hsa05132 Salmonella infection	
ENTPD1-AS1			22859060						18567823		hsa00230 Purine metabolism<br>hsa00240 Pyrimidine metabolism<br>hsa05169 Epstein-Barr virus infection	
EPHA6											hsa04360 Axon guidance	0.221
ERC1										NF-KB 15218148 Exocytosis 17681140	hsa04064 NF-kappa B signaling pathway	
EXOC6B										Exocyst complex 16227995		
FAM149B1												
FAT4	24150215<br>22484628						25151357					
FBXL17												
FHIT		18077326									hsa00230 Purine metabolism<br>hsa05222 Small cell lung cancer<br>hsa05223 Non-small cell lung cancer	
FMN2										p21 23375502	hsa04320 Dorso-ventral axis formation	0.042
FNDC3B	20799341			22510613	22510613							
FOXN3												0.07
FOXP1	24121790<br>20579941<br>18765795		24436148								hsa05206 MicroRNAs in cancer	
FRYL	17854671											
GALNTL6											hsa00512 Mucin type O-Glycan biosynthesis<br>hsa01100 Metabolic pathways	
GIPC2		17873379										0.145
GPC5	17210683											
GPC6	21949389											0.172
GPR75-ASB3												
GRIN2B			(21207374<br>23535601)								hsa04724 Glutamatergic synapse	
GULP1					22451657							0.221
HDAC9											hsa05034 Alcoholism<br>hsa05203 Viral carcinogenesis	
HIPK3									22983607	RUNX2 20484411		
HMGA2	23738515		19179606								hsa05202 Transcriptional misregulation in cancer<br>hsa05206 MicroRNAs in cancer	
HSD17B12											hsa00062 Fatty acid elongation<br>hsa00140 Steroid hormone biosynthesis<br>hsa01040 Biosynthesis of unsaturated fatty acids<br>hsa01100 Metabolic pathways<br>hsa01212 Fatty acid metabolism	
IMMP2L											hsa03060 Protein export	
INPP4B	21127264			21127264					24051093		hsa00562 Inositol phosphate metabolism<br>hsa01100 Metabolic pathways<br>hsa04070 Phosphatidylinositol signaling system	
JMJD1C	24896186									De-methylation 24240613	hsa05202 Transcriptional misregulation in cancer	
KCNIP4												
KCNMA1	24367615								21660954			
KIAA0825												
KIAA1217												
KIF13A	11861365			20208530								
LCOR												0.27
LIMCH1												0.07
LINC00472												

table S5a. Assignment of 163 common CIS genes in DLD-1 KRAS$^{wt/-}$ and RKO BRAF$^{wt/-/-}$ to colorectal cancer pathways

Gene symbol	Cancer gene	Pathway										Mutual exclusivity with KRAS mutation in TCGA COAD
		Wnt	EGFR/ Ras/MAPK	PI3K	TGFB	TP53	Hippo	Rap1	Cellular glucose metabolism	Other	KEGG Pathway	
LPP	11433529<br>8812423				23447672							0.27
LRP1B	18948947											
LSAMP	20048075<br>19724913<br>14667507											
LTBP1					KEGG						hsa04350 TGF-beta signaling pathway	
MAGI2	21307934		15310755	21307934				KEGG			hsa04015 Rap1 signaling pathway<br>hsa04530 Tight junction<br>hsa04668 TNF signaling pathway	
MAML2	23821214									Notch<br>12370315,<br>22069191	hsa04330 Notch signaling pathway	
MARCH1												
MED13L										Rb<br>22249253		
MET	18636147		8387483	KEGG							hsa04060 Cytokine-cytokine receptor interaction<br>hsa04144 Endocytosis<br>hsa04151 PI3K-Akt signaling pathway<br>hsa04360 Axon guidance<br>hsa04510 Focal adhesion<br>hsa04520 Adherens junction<br>hsa05100 Bacterial invasion of epithelial cells<br>hsa05120 Epithelial cell signaling in Helicobacter pylori infection<br>hsa05144 Malaria<br>hsa05200 Pathways in cancer<br>hsa05202 Transcriptional misregulation in cancer<br>hsa05205 Proteoglycans in cancer<br>hsa05206 MicroRNAs in cancer<br>hsa05211 Renal cell carcinoma<br>hsa05218 Melanoma	
MGAT4C	22496589										hsa00510 N-Glycan biosynthesis<br>hsa01100 Metabolic pathways	
MIR31HG			23322774				24779718					
MYO16												
NAALADL2												
NAV3												
NCOA3	16084947			15380517					24584933			0.00017
NEDD4L		24312311<br>23396981									hsa04120 Ubiquitin mediated proteolysis<br>hsa04144 Endocytosis<br>hsa04960 Aldosterone-regulated sodium reabsorption	
NFIB	23685749<br>22829077<br>19841262											
NRG1	15466169		8453670								hsa04012 ErbB signaling pathway	
NRG3			9275162								hsa04012 ErbB signaling pathway	
NSMCE2										DNA repair<br>16055714		0.035
OPCML			16384911									
PARD3	20215515		24425787				KEGG	KEGG			hsa04015 Rap1 signaling pathway<br>hsa04062 Chemokine signaling pathway<br>hsa04080 Neuroactive ligand-receptor interaction<br>hsa04144 Endocytosis<br>hsa04390 Hippo signaling pathway<br>hsa04520 Adherens junction<br>hsa04530 Tight junction	0.118
PCDH15												
PCDH7												
PCDH9												
PDE4D			21478863								hsa00230 Purine metabolism<br>hsa05032 Morphine addiction	
PDP1									24486017			
PDZRN4												
PHACTR4	23319639<br>20824714									Rb<br>23319639		
PHLDB2				20587420					20587420			
PKHD1												0.038

table S5a. Assignment of 163 common CIS genes in DLD-1 KRAS$^{wt/-}$ and RKO BRAF$^{wt/-/-}$ to colorectal cancer pathways

Gene symbol	Cancer gene	Pathway										Mutual exclusivity with KRAS mutation in TCGA COAD
		Wnt	EGFR/ Ras/MAPK	PI3K	TGFB	TP53	Hippo	Rap1	Cellular glucose metabolism	Other	KEGG Pathway	
PPHLN1												
PPP3CA		KEGG	KEGG								hsa04010 MAPK signaling pathway<br>hsa04020 Calcium signaling pathway<br>hsa04114 Oocyte meiosis<br>hsa04210 Apoptosis<br>hsa04310 Wnt signaling pathway<br>hsa04360 Axon guidance<br>hsa04370 VEGF signaling pathway<br>hsa04380 Osteoclast differentiation<br>hsa04650 Natural killer cell mediated cytotoxicity<br>hsa04660 T cell receptor signaling pathway<br>hsa04662 B cell receptor signaling pathway<br>hsa04720 Long-term potentiation<br>hsa04724 Glutamatergic synapse<br>hsa04728 Dopaminergic synapse<br>hsa05010 Alzheimer's disease<br>hsa05014 Amyotrophic lateral sclerosis (ALS)<br>hsa05031 Amphetamine addiction<br>hsa05152 Tuberculosis<br>hsa05166 HTLV-I infection	
PREP				24244481<br>24936056								
PTPRD	19478061									STAT3<br>19478061		
PTPRG	15155950		20651337									
PVT1	22869583											
RBFOX1	23918370											
RGS6			23995786									
RNF111			20965945		23467611							
RNF217	25298122											
ROBO1	24429703										hsa04360 Axon guidance	
ROBO2	22561520										hsa04360 Axon guidance	
ROCK2	22510280	18784435							22355071		hsa04062 Chemokine signaling pathway<br>hsa04270 Vascular smooth muscle contraction<br>hsa04310 Wnt signaling pathway<br>hsa04360 Axon guidance<br>hsa04510 Focal adhesion<br>hsa04670 Leukocyte transendothelial migration<br>hsa04810 Regulation of actin cytoskeleton<br>hsa05130 Pathogenic Escherichia coli infection<br>hsa05131 Shigellosis<br>hsa05132 Salmonella infection<br>hsa05205 Proteoglycans in cancer	0.07
ROR1		22343533									hsa04710 Circadian rhythm<br>hsa05321 Inflammatory bowel disease	
RSRC1	24214394											
RUNX1	22722202<br>22237106	23180629			23180629		23180629				hsa05200 Pathways in cancer<br>hsa05202 Transcriptional misregulation in cancer<br>hsa05220 Chronic myeloid leukemia<br>hsa05221 Acute myeloid leukemia	
RUNX2		23180629	22710434		23180629		23180629		21913213		hsa05202 Transcriptional misregulation in cancer	0.05
SBF2			19912440	19912440								
SCAF11												
SEMA5A											hsa04360 Axon guidance	0.02
SETD5												0.14
SIAH1		KEGG	17998205<br>18089810<br>7972125			KEGG			21734459		hsa04115 p53 signaling pathway<br>hsa04120 Ubiquitin mediated proteolysis<br>hsa04310 Wnt signaling pathway	
SIPA1L1		17336901						KEGG			hsa04015 Rap1 signaling pathway	0.038

table S5a. Assignment of 163 common CIS genes in DLD-1 KRAS$^{wt/-}$ and RKO BRAF$^{wt/-/-}$ to colorectal cancer pathways

Gene symbol	Cancer gene	Pathway										Mutual exclusivity with KRAS mutation in TCGA COAD
		Wnt	EGFR/ Ras/MAPK	PI3K	TGFB	TP53	Hippo	Rap1	Cellular glucose metabolism	Other	KEGG Pathway	
SLC1A3			(21207374 23535601)								hsa04724 Glutamatergic synapse	0.22
SLC24A3												
SNAP25-AS1									17947242		hsa04130 SNARE interactions in vesicular transport<br>hsa04721 Synaptic vesicle cycle<br>hsa04911 Insulin secretion	
SND1	25266736										hsa05169 Epstein-Barr virus infection<br>hsa05203 Viral carcinogenesis	
SORCS3												
SPECC1												
STAG1			14639658								hsa04110 Cell cycle	
STPG2												
STXBP5L												0.27
SUPT3H											hsa05202 Transcriptional misregulation in cancer	
SYT1				19255425					19255425			
TANC2												
TCF7L2	21892161	KEGG					KEGG		25398947		hsa04310 Wnt signaling pathway<br>hsa04390 Hippo signaling pathway<br>hsa04520 Adherens junction<br>hsa04916 Melanogenesis<br>hsa05200 Pathways in cancer<br>hsa05210 Colorectal cancer<br>hsa05213 Endometrial cancer<br>hsa05215 Prostate cancer<br>hsa05216 Thyroid cancer<br>hsa05217 Basal cell carcinoma<br>hsa05221 Acute myeloid leukemia<br>hsa05412 Arrhythmogenic right ventricular cardiomyopathy (ARVC)	
TEAD1							KEGG				hsa04390 Hippo signaling pathway	
THADA	12955091 25209362											
THSD4												
TNIK		19816403 20530691										
TNRC6B										miRNA 19898466		
TPRG1												
TRAPPC9			23474757							NFKB 23474757		
TRHDE												
TRPS1	23028188		21868360							EMT 21673316		
TXNRD1									23743293		hsa00240 Pyrimidine metabolism<br>hsa00450 Selenocompound metabolism	
UBE2E2											hsa04120 Ubiquitin mediated proteolysis<br>hsa04141 Protein processing in endoplasmic reticulum	0.17
UBR5	23407552								22096030	DSB 22884692, 17074762	hsa04120 Ubiquitin mediated proteolysis	
UCA1		24495014			24648515							
UNC13C	24292195										hsa04721 Synaptic vesicle cycle	
UTRN	24416132 17384672											
VMP1	23637631		22535956							Autophagy 20550938, 23884233, 17940279		
VPS13B										Vesicle sorting/ transport 12730828, 22442115		
WDFY3										Autophagy 15292400, 23445924		

table S5a. Assignment of 163 common CIS genes in DLD-1 KRAS$^{wt/-}$ and RKO BRAF$^{wt/-/-}$ to colorectal cancer pathways

Gene symbol	Cancer gene	Pathway										Mutual exclusivity with KRAS mutation in TCGA COAD
		Wnt	EGFR/ Ras/MAPK	PI3K	TGFB	TP53	Hippo	Rap1	Cellular glucose metabolism	Other	KEGG Pathway	
WDPCP												
WWOX	11572989									Hh 24393846		
ZCCHC7										TRAMP 21878619		
ZFHX3	20854080 15750593				20599712					RUNX3 20599712		
ZPLD1												
Fraction of genes	31%	10%	19%	7%	6%	1%	6%	2%	11%	14%	37%	

table S5. Assignment of 163 common CIS genes in DLD-1 KRAS$^{wt/-}$ and RKO BRAF$^{wt/-/-}$ to colorectal cancer pathways. Primary publications in PubMed along with the Kyoto Encyclopedia of Genes and Genomes (KEGG) were searched (i) to determine whether the CIS genes were cancer genes (defined as recurringly somatically mutated by point mutations, amplifications, deletions or translocations) in any human tumor type, (ii) for genetic or functional evidence for assignment to any of the canonical CRC pathways Wnt, EGFR/Ras/MAPK, PIK3CA, or TGFB, or to the Hippo and Rap1 pathways associated with EGFR/Ras/MAPK signalling, (iii) for evidence for a role in intracellular glucose metabolism. Numbers, PubMed PMIDs of publication(s) supporting the assignment. KEGG, assignment to pathway based solely on KEGG. Numbers within parentheses indicate a weaker link (glutamate pathway in case of Ras pathway assignments). Mutual exclusivity was analysed as described by Gao et al (26). Briefly, CNA, somatic mutations and mRNA expression data from MSS tumors in the ICGC COAD dataset were included and mutual exclusivity Fisher's Exact test as Odds Ratio < 0.1 with a P < 0.05.

table S5b. Pathway assignments of 163 randomly sampled ENSEMBL genes

Gene symbol	Cancer gene	Wnt	EGFR/ErbB/ Ras/MAPK	PI3K	TGFB	TP53	Hippo	Rap1	Cellular glucose metabolism or transport	Other pathway	KEGG Pathway
AATK											
ABI2											hsa04810 Regulation of actin cytoskeleton
ACAT2											hsa01212 Fatty acid metabolism + related pathways
ACKR6											
AKR1C3											hsa00140 Steroid hormone biosynthesis
ANGPTL2											
ANPEP											hsa00480 Glutathione metabolism
AOC4P											
APOBEC3A	24728294										
ARRDC1											
ATG3P1											
ATP5A1P2											
ATP5I											hsa00190 Oxidative phosphorylation
ATXN2L											
BEST4											
BNIP2											
C12orf61											
CACNA1A			KEGG								hsa04726 Serotonergic synapse + other
CCL28											hsa04062 Chemokine signalling pathway
CCNB1IP1P2											
CCR8			12645948								hsa04062 Chemokine signalling
CCSER2											
CD52											
CDK4	24793136			KEGG		KEGG					hsa05200 Pathways in cancer<br>hsa04151 PI3K-Akt signaling pathway<br>hsa04115 p53 signaling pathway
CEACAM7											
CELSR2									23177622		
CEP164											
CHMP1AP1											
CIB4											
CORO1B											
COX10-AS1											
CYP2D6											hsa00982 Drug metabolism - cytochrome P450
CYP4F22											
DHX32											
DNMBP-AS1											
EDN3											
EMC4											
EML4	19170230		19170230								hsa05223 Non-small cell lung cancer
ETV3			22028470								
FAM73B											
FAM86HP											
FARP1											
FBXL19-AS1											
FBXO7										22212761<br>NF-KB	
FHP1											
FUNDC2P3											
GALNS											hsa00531 Glycosaminoglycan degradation
GLA											
GLIPR1L2											
GPR160											
GRM6											hsa04724 Glutamatergic synapse
GTDC1											
HMBOX1											
HMGN2P28											
HNRNPDL											
IFI35											
IGHD1OR15-1A											
IGHVII-67-1											
IGKV3-20	24504345										
ISCA1P1											
KIAA1841											
KLHDC9											
KRT27											
KRT31											
LINC00523											
LINC00560											
LINC00604											
LINC00605											
LRP5	24706814	1029006, KEGG									hsa04310 Wnt signalling pathway
LSM4											hsa03018 RNA degradation<br>hsa03040 Spliceosome
MAGEE2											
MEGF10											
METTL7B											
MIR125B1											hsa05206 MicroRNAs in cancer
MIR142											
MIR3182											
MIR4312											
MIR4451											
MIR4767											
MROH6											
NEBL-AS1											
NEUROD2											
NOP14-AS1											
NOTCH2P1											
NPC1											hsa04975 Fat digestion and absorption
NPNT											hsa04512 ECM-receptor interaction
NTNG1											hsa04360 Axon guidance
NUSAP1											
OCSTAMP											
OR10A5											hsa04740 Olfactory transduction
OR1S2											hsa04740 Olfactory transduction
OR2U1P											
OR51A4											hsa04740 Olfactory transduction
OTOP1											
P2RX7				19540829							hsa04020 Calcium signalling<br>hsa04080 Neuroactive ligand-receptor interaction
PAICSP3											
PAPPA2											
PF4V1											hsa04062 Chemokine signaling pathway
POM121L7											
PPEF2											
PRSS35											
PRYP6											
PSENEN											
PSG9											
RAB27B											hsa04972 Pancreatic secretion
RABEP2											
RAC1P4											
RBM17											hsa03040 Spliceosome
RN7SKP28											

table S5b. Pathway assignments of 163 randomly sampled ENSEMBL genes

Gene symbol	Cancer gene	Wnt	EGFR/ErbB/ Ras/MAPK	PI3K	TGFB	TP53	Hippo	Rap1	Cellular glucose metabolism or transport	Other pathway	KEGG Pathway
RN7SL552P											
RN7SL686P											
RNA5SP208											
RNA5SP299											
RNU1-132P											
RNU1-98P											
RNU2-8P											
RNU4ATAC14P											
RNU6-494P											
RNU6-858P											
RNU6-898P											
RNU7-134P											
RPL12P33											
RPL39P29											
RPL7AP6											
RPL7L1											
RPS3AP30											
RPS4XP19											
S100A16											
S1PR3			21984966	21984966							hsa04080 Neuroactive ligand-receptor interaction
SCRN2											
SEMA6A											hsa04360 Axon guidance
SEPHS1P5											
SNAP25											
SNORA3											
SNORA55											
SPSB4											
SS18	7539744	24166495									hsa05202 Transcriptional misregulation in cancer
SSR4											hsa04141 Protein processing in ER
STAC											hsa00052 Galactose metabolism
SUGT1P3											
SULT1E1											hsa00140 Steroid hormone biosynthesis
TAGLN		18782851	11773051								
TBC1D10C			23248241<br>17230191								
TBC1D4									25043022		hsa04919 Thyroid hormone signaling pathway
TCAIM											
TCTEX1D2											
THSD7B											
THY1											hsa04670 Leukocyte transendothelial migration
TMEM51-AS1											
TMTC2											
TRGV7											
UNC93B7											
UQCRHP1											
USP17L25											
VAC14-AS1											
WBSCR16											
WDR41											
WDR54											
VTRNA2-1											
ZC3H7A											
ZFPM1											
ZNF486											
ZNF526											
Fraction of genes	4%	2%	4%	2%	0%	1%	0%	0%	1%	1%	21%

table S5c. Pathway assignments of 163 randomly sampled ENSEMBL genes.

Gene symbol	Cancer gene	Wnt	EGFR/ErbB/ Ras/MAPK	PI3K	TGFB	TP53	Hippo	Rap1	Cellular glucose metabolis m or transport	Other pathway	KEGG Pathway
AADACL2											
ABCC5-AS1											
ACOXL	23770605, 20731705										
APIP											hsa00270 Cysteine and methionine metabolism, hsa01100 Metabolic pathways
ARHGEF12	10681437, 19734946, 21147910		16565089							RhoA: 11373293, 11799111	hsa04270 Vascular smooth muscle contraction, hsa04360 Axon guidance, hsa04611 Platelet activation, hsa04810 Regulation of actin cytoskeleton, hsa05152 Tuberculosis , hsa05200 pathways in cancer, hsa05205 Proteoglycans in cancer
ARMCX4											
BCORL1	21989985, 24047651, 24515802, 24896186, 25326804										
BUB1	23747338, 9521327, 10969775										hsa04110 cell cycle, hsa04114 oocyte meiosis, hsa04914 Progesterone-mediated oocyte maturation, hsa05166 HTLV-I infection
C10orf115											
C11orf49											
C12orf80											
C4orf29											
CACNG6											hsa04010 MAPK signaling pathway , hsa04260 Cardiac muscle contraction , hsa04261 Adrenergic signaling in cardiomyocytes , hsa04921 Oxytocin signaling pathway, hsa05410 Hypertrophic cardiomyopathy (HCM), hsa05412 Arrhythmogenic right ventricular cardiomyopathy (ARVC), hsa05414 Dilated cardiomyopathy
CALB1											hsa04961 Endocrine and other factor-regulated calcium reabsorption
CBX7	23955597, 17374722	25351982	24194518								
CCR9			11493434	11493434						Jak/Stat: 15147562, CCL25-CCR9: 23371851	hsa04672 Intestinal immune network for IgA production, hsa04060 Cytokine-cytokine receptor interaction, hsa04062 Chemokine signaling pathway
CD276											hsa04514 Cell adhesion molecules (CAMs)
CD40											hsa04514 Cell adhesion molecules (CAMs)<br>hsa05320 Autoimmune thyroid disease<br>hsa05330 Allograft rejection<br>hsa04060 Cytokine-cytokine receptor interaction<br>hsa04064 NF-kappa B signaling pathway<br>hsa04672 Intestinal immune network for IgA production<br>hsa05144 Malaria<br>hsa05310 Asthma<br>hsa05322 Systemic lupus erythematosus<br>hsa05340 Primary immunodeficiency<br>hsa05416 Viral myocarditis<br>hsa05145 Toxoplasmosis<br>hsa04620 Toll-like receptor signaling pathway<br>hsa04660 T cell receptor signaling pathway<br>hsa05166 HTLV-I infection<br>hsa05169 Epstein-Barr virus infection<br>hsa05202 Transcriptional misregulation in cancer
CD48										NFκB: 21792887	hsa04650 Natural killer cell mediated cytotoxicity
CEP85											
CER1										Nodal/Activin: 24585688	
CGB5											
CHAF1B											
CTDSPL	22491060 , 15051889										
CXorf21											
DEFB104A											
DLEU1	23047824										
DLGAP1-AS3											
DNAJA1P3											
DPAGT1		23703614, 23178939									
EBAG9										RCAS1-RCS1-R: 20372814	
EIF2B5-AS1											
ERVFRD-1											
EXTL1										hedgehog/parathyroid hormone-related peptide (PTHrP): 10878610	hsa00534 Glycosaminoglycan biosynthesis - heparan sulfate , hsa01100 Metabolic pathways
FAM188B											

table S5c. Pathway assignments of 163 randomly sampled ENSEMBL genes.

Gene symbol	Cancer gene	Wnt	EGFR/ErbB/ Ras/MAPK	PI3K	TGFB	TP53	Hippo	Rap1	Cellular glucose metabolism or transport	Other pathway	KEGG Pathway
FCGR2C											hsa04145 phagosome, hsa04380 osteoclast differenatiation, hsa05140 leishmaniasis, hsa05150 Staphylococcus aureus infection
FNTAP2											
FTH1P12											
GABRR2											hsa05032 Morphine addiction, hsa05033 Nicotine addiction, hsa04080 Neuroactive ligand-receptor interaction, hsa04723 Retrograde endocannabinoid signaling, hsa04727 GABAergic synapse
GAPDHP65											
GATA3-AS1	15361840										
GDI2											
GNB4											hsa05032 Morphine addiction, hsa04713 Circadian entrainment , hsa04723 Retrograde endocannabinoid signaling, hsa04725, hsa04727 GABAergic synapse, hsa04014 Ras signaling pathway , hsa04062 Chemokine signaling pathway , hsa04151 PI3K-Akt signaling pathway , hsa04724 Glutamatergic synapse, hsa04726 Serotonergic synapse , hsa04728 Dopaminergic synapse , hsa05034 Alcoholism, hsa05200 Pathways in cancer
HDAC10										ROS: 20680488	hsa05034 Alcoholism, hsa05203 Viral carcinogenesis
HMGN1P28											
HPS4											
ICA1											hsa04940 Type I diabetes mellitus
IDO1											hsa00380 tryptophan metabolism, hsa01100 metabolic pathways, hsa05143 African trypanosomiasis
IFITM4P											
IFT122											
IGLV1-62											
ILKAP		11331582								ILK signaling: 14990992	
INF2										Rho/mDia 23620398	hsa04610 Complement and coagulation cascades
IREB2											
KIAA0895											
LANCL1											
LCP1	10469447										
LINC00324											
LINC00344											
LINC00440											
LRRC25											
MIR16-1						25040181					hsa05206 MicroRNAs in cancer
MIR320B1											
MIR33B									23716591		
MIR449A										Notch: 21569010	
MIR4675											
MIR4757											
MIR504											
MKRN9P											
MORC1											
MPDZ											hsa04530 tight junction
MPTX1											
MURC											hsa00510 N-Glycan biosynthesis , hsa01100 metabolic pathways
N6AMT1											
NANOGP4											
NCOA6											
NFE2L3											
NKX2-4											
NUP188											hsa03013 RNA transport
NUTM2E	25481751										
OR10A7											hsa04740 Olfactory transduction
OR10J1											hsa04740 Olfactory transduction
OR1D5											

table S5c. Pathway assignments of 163 randomly sampled ENSEMBL genes.

Gene symbol	Cancer gene	Wnt	EGFR/ErbB/ Ras/MAPK	PI3K	TGFB	TP53	Hippo	Rap1	Cellular glucose metabolism or transport	Other pathway	KEGG Pathway
OR4G1P											
PRKX-AS1											
PRR24											
PSMC1P10											
PSMD12											hsa03050 Proteasome, hsa05169 Epstein-Barr virus infection
PTGR1											
PTPRN											hsa04940 Type I diabetes mellitus
RAB30-AS1											
REREP1Y											
RFX2			15024578								
RILPL1											
RMND5B											
RN7SKP85											
RN7SL133P											
RN7SL205P											
RN7SL369P											
RN7SL425P											
RN7SL446P											
RN7SL480P											
RN7SL496P											
RN7SL518P											
RN7SL537P											
RN7SL557P											
RNF11					20676133						
RNF121										NFkB: 25388546	
RNU2-55P											
RNU6-1002P											
RNU6-1108P											
RNU6-1281P											
RNU6-208P											
RNU6-420P											
RNU6-510P											
RNU6ATAC38P											
RNU7-80P											
RPL7P45											
RPL9P7											
RPRML											
RPS24P17											
SART1										antiviral effector genes: 25481564	hsa03040 splicesosome
SEC61A2											
SLC17A6											hsa04721 Synaptic vesicle cycle, hsa05033 Nicotine addiction, hsa04723 Retrograde endocannabinoid signaling, hsa04724 Glutamatergic synapse
SLC1A6											hsa04724 Glutamatergic synapse
SMC3	25006131, 23955599										hsa04114 oocyte meiosis, hsa04110 cell cycle, hsa03050 proteasome, hsa05169 Epstein-Barr virus infection
SMIM2											
SNHG14											
SNORD24											
SPANXC											
SPG7											
SRC			KEGG					KEGG			hsa04510, hsa04520, hsa04917, hsa04012,hsa05120, hsa04662, hsa04144, hsa04915, hsa05205, hsa04540, hsa04919, hsa04015, hsa04062, hsa04370, hsa04530, hsa04611, hsa04727, hsa04750, hsa04810, hsa04912

table S5c. Pathway assignments of 163 randomly sampled ENSEMBL genes.

Gene symbol	Cancer gene	Wnt	EGFR/ErbB/ Ras/MAPK	PI3K	TGFB	TP53	Hippo	Rap1	Cellular glucose metabolism or transport	Other pathway	KEGG Pathway
TAS1R2									22552794		hsa04742 Taste transduction, hsa04973 Carbohydrate digestion and absorption
TCEAL1											
TCEB3C											
TCERG1											hsa03040 Spliceosome
TEX101											
TIGD7											
TLR1											
TMCO1											
TMEM132C											
TMEM52											
TOX4											
TPM3P2											
TPTE2P3											
TRAJ48											
TRBV6-1											hsa04620 Toll-like receptor signaling pathway , hsa05152 Tuberculosis, hsa05145 Toxoplasmosis
TRIM14											
TRIM23											
TSPY20P											
TTC36											
TTC39A											
TTTY9A											
USE1											hsa04130
USP9YP15											
USP9YP21											
WBP1LP6											
VN1R40P											
YIPF2											hsa05206 micro RNAs in cancer
ZFAND6P1											
ZNF639											
ZNF879											
ZSCAN16											
Fraction of genes	8%	3%	3%	1%	1%	1%	0%	1%	1%	7%	21%

table S5d. Pathway assignments of 163 randomly sampled ENSEMBL genes.

Gene symbol	PubMed papers	Cancer gene	Wnt	EGFR/ErbB/ Ras/MAPK	PI3K	TGFB	TP53	Hippo	Rap1	Cellular glucose metabolism or transport	Other pathway	KEGG Pathway
ABCC10	68											hsa02010: ABC transporters
ACR	945											
ADAMTS4	91					17900349					JNK: 23438438 NF-κB: 23602832	
ADC	23											
AKR1C6P	0											
ALG3P1	0											
AMICA1	4											
ANKZF1	2											
ARMCX3	3		23844091									
ASIC3	271									18466760		hsa04750 Inflammatory mediator regulation of TRP channels
ATP2C2	25											
AWAT1	2											
B4GALT7	11											
BAZ2B	6											
C17orf66	0											
C1GALT1	53											hsa00512:Mucin type O-Glycan biosynthesis , hsa01100:Metabolic pathways
C20orf195	0											
C21orf59	2											
CCDC163P	0											
CCDC180	0											
CCDC34	0											
CDC42P1	0											
CDV3	6											
CHRNA4	207											hsa05033: Nicotine addiction, hsa04080: Neuroactive ligand-receptor interaction, hsa04725: Cholinergic synapse
CHST2	13											hsa00533: Glycosaminoglycan biosynthesis - keratan sulfate
CLRN1-AS1	0											
CTGLF12P	0											
CYCSP10	0											
CYCSP39	0											
CYFIP2	38			21252116			17245118					hsa03013: RNA transport , hsa04810: Regulation of actin cytoskeleton
CYP46A4P	0											
CYP4A22-AS1	0											
DDRGK1	3										NF-κB: 23675531	
DDX11L2	0											
DEFB108B	0											
DNAH12	4											hsa05016:Huntington's disease
DOCK11	13											
EGOT	55											
FAM135A	0											
FAM21C	0											
FAM222A-AS1	0											
FAM224B	0											
FBXL21	4											
FOXN3-AS2	0											
FRMPD4-AS1	0											
GAGE2E	0											
GANC	613									7018580		hsa01100: Metabolic pathways , hsa00500:Starch and sucrose metabolism , hsa00052:Galactose metabolism
GHITM	7											
GNG5P1	0											
GRM4	22										glutamate/GABA: 20338729	hsa04724: Glutamatergic synapse, hsa04080 Neuroactive ligand-receptor interaction, hsa04742 Taste transduction
HMGN2P17	0											
HMX1	35		9435283									
HSD11B1L	1											
IDH3G	6									24265765		
IFI30	61											hsa04612: Antigen processing and presentation
IGHV3-74	5											
IGLV6-57	2											
IGLVI-20	0											
IL9	145					24108699					Notch: 22503540 JAK/STAT: 24550509	hsa04060:Cytokine-cytokine receptor interaction, hsa04630: Jak-STAT signaling pathway, hsa04640: Hematopoietic cell lineage, hsa05310: Asthma - Homo sapiens
INCENP	47										survivin/ABK: 21057000	

table S5d. Pathway assignments of 163 randomly sampled ENSEMBL genes.

Gene symbol	PubMed papers	Cancer gene	Wnt	EGFR/ErbB/ Ras/MAPK	PI3K	TGFB	TP53	Hippo	Rap1	Cellular glucose metabolism or transport	Other pathway	KEGG Pathway
ISM2	1											
ITPA	343											hsa00230: Purine metabolism, hsa00240: Pyrimidine metabolism, hsa01100: Metabolic pathways, hsa00983: Drug metabolism - other enzymes
KCNQ5-IT1	0											
KIAA1377	4											
KRT19P6	0											
KRTAP4-7	0											
KYNUP1	0											
LAMTOR5P1	0											
LANCL2	20				25273559						ABA: 19667068, 24628287	
LDLRAD4-AS1	0											
LGALS4	151					21370740						
LINC00279	0											
LINC00362	0											
LINC00610	0											
LIPF	49											hsa00561:Glycerolipid metabolism, hsa01100:Metabolic pathways , hsa04975:Fat digestion and absorption
LRFN2	5											
LRRC4	23			16723503, 17541939 , 17549698	17549698						NF-kappaB, STAT3, JNK2/c-Jun: 16723503	hsa04514: Cell adhesion molecules (CAMs), hsa04360: Axon guidance
MEF2AP1	0											
METTL21AP1	0											
MIR1234	0											
MIR30C2	0											hsa05206: MicroRNAs in cancer
MIR4464	0											
MIR455	1											
MIR503	2					25164015						
MIR941-1	0											
MRPL4	4										HIF-1α: 21625490	hsa03010:Ribosome
MSGN1	11		21750544									
MYBPC1	21											
MYC	24954	6285209	KEGG	KEGG		KEGG						hsa05230, hsa04550, hsa05200, hsa05161, hsa05166, hsa05210, hsa04010, hsa04110, hsa04310, hsa04350, hsa04630, hsa04919, hsa05169, hsa05202, hsa05213, hsa05216, hsa05219, hsa05221
MYL5	3											hsa04670: Leukocyte transendothelial migration, hsa04510: Focal adhesion, hsa04530: Tight junction , hsa04810: Regulation of actin cytoskeleton
MYOCD	66	19276386						25087979				
NEK2	204		23804296	20581865	24018644			21076410				
OR51H2P	0											
OR5BN1P	0											
OR6C74	0											hsa04740: Olfactory transduction
PDE4D	412	23536305, 18519675, 20651033, 22362584, 24358143		25289091						17991719	cAMP: 24518597, 19371330	hsa00230: Purine metabolism , hsa04024: cAMP signaling pathway , hsa05032: Morphine addiction
PECR	8											hsa01040: Biosynthesis of unsaturated fatty acids, hsa01212:Fatty acid metabolism, hsa04146:Peroxisome, hsa01100: Metabolic pathways
PGK1	468									18097579, 15225644	Notch: 24878247	hsa01200: Carbon metabolism , hsa01230: Biosynthesis of amino acids , hsa01100: Metabolic pathways , hsa04066:HIF-1 signaling pathway , hsa00010:Glycolysis / Gluconeogenesis
PI15	11	25117815										
PLEKHA1	45				12101241					21204784		
PPAPDC3	2											
PSMA3	26											hsa03050: Proteasome
PSMD5-AS1	0											
PSPC1P2	0											
RBM22P7	0											
RN7SKP77	0											
RN7SKP93	0											
RN7SL25P	0											
RN7SL297P	0											
RN7SL684P	0											
RN7SL842P	0											
RNA5SP114	0											
RNA5SP115	0											
RNF113B	2											
RNF130	3											
RNGTTP1	0											
RNU1-28P	0											
RNU6-1081P	0											

table S5d. Pathway assignments of 163 randomly sampled ENSEMBL genes.

Gene symbol	PubMed papers	Cancer gene	Wnt	EGFR/ErbB/ Ras/MAPK	PI3K	TGFB	TP53	Hippo	Rap1	Cellular glucose metabolism or transport	Other pathway	KEGG Pathway
RNU6-1157P	0											
RNU6-1171P	9											
RNU6-1175P	0											
RNU6-483P	0											
RNU6-490P	0											
RNU6-984P	0											
RNU7-96P	0											
RNY3P6	0											
RPL7P48	0											
RPL7P56	0											
SCAMP3	13			9658162								
SCARNA1	0											
SERPINH1P1	1											
SLC25A1P4	0											
SNHG5	2	18406879										
SNORD125	0											
SOX17	418		10549281			19479035						hsa04310: Wnt signaling pathway
SPRTN	5	25261934										
SSR3	9											hsa04141: Protein processing in endoplasmic reticulum
SSR4P1	0											
SSX1	211	7539744, 7495284, 9428816.	24166495				22550415					hsa05202: Transcriptional misregulation in cancer
STX19	5											hsa04130: SNARE interactions in vesicular transport
SURF2	8											
SYT10	4											
TBXAS1	24										prostaglandin: 16250911	hsa04611: Platelet activation, hsa00590: Arachidonic acid metabolism, hsa01100: Metabolic pathways
THYN1	4											
TLX1	193	1681546, 1683261, 11520572, [illegible]										hsa05202:Transcriptional misregulation in cancer
TMEM144	2											
TPMTP1	0											
TRAJ9	1											
UBE2M	9		19888210									hsa04120 Ubiquitin mediated proteolysis
UCP1	1236		15684380	2030762, 20307629								
UGT1A6	474											hsa00140, hsa00982, hsa00980, hsa05204, hsa00040, hsa00053, hsa00500, hsa00830, hsa00860, hsa00983, hsa01100
UQCC	14											
USP17L28	0											
VCAN	391	24951259		16648628		23824740						hsa04514: Cell adhesion molecules (CAMs)
WEE1	774					12669309					CDK : 23051732	hsa04110: Cell cycle
XKR6	8											
XKRYP3	0											
XPNPEP3	5											
ZFYVE9P2	0											
ZNF112	0											
ZNF552	1											
ZNF844	0											
ZNF92P3	0											
Fraction of genes		6%	6%	5%	2%	5%	1%	1%	0%	4%	7%	20%

table S6. Barcoded primers for amplification of transposon integration sites

Barcoded oligos	Sequence 5' to 3'
sp3-1-aacgca	aacgcaTTAACGTACGTCACAATATGATTATCTTTC
sp3-2-aagggt	aagggtTTAACGTACGTCACAATATGATTATCTTTC
sp3-3-acactg	acactgTTAACGTACGTCACAATATGATTATCTTTC
sp3-4-agcata	agcataTTAACGTACGTCACAATATGATTATCTTTC
sp3-5-attacc	attaccTTAACGTACGTCACAATATGATTATCTTTC
sp3-6-atttgg	atttggTTAACGTACGTCACAATATGATTATCTTTC
sp3-7-ccgttt	ccgtttTTAACGTACGTCACAATATGATTATCTTTC
sp3-8-gattga	gattgaTTAACGTACGTCACAATATGATTATCTTTC
Splink2-1-aacgca	aacgcaGTGGCTGAATGAGACTGGTGTCGAC
Splink2-2-aagggt	aagggtGTGGCTGAATGAGACTGGTGTCGAC
Splink2-3-acactg	acactgGTGGCTGAATGAGACTGGTGTCGAC
Splink2-4-agcata	agcataGTGGCTGAATGAGACTGGTGTCGAC
Splink2-5-attacc	attaccGTGGCTGAATGAGACTGGTGTCGAC
Splink2-6-atttgg	atttggGTGGCTGAATGAGACTGGTGTCGAC
Splink2-7-ccgttt	ccgtttGTGGCTGAATGAGACTGGTGTCGAC
Splink2-8-gattga	gattgaGTGGCTGAATGAGACTGGTGTCGAC

table S6. Barcoded primers for amplification of transposon integration sites. Barcodes in lowercase and primer sequences in capital letters.

table S7. Target sequences and TaqMan probes for siRNA mediated gene knock-down

Gene	ON-TARGETplus SMARTpool target sequences	TaqMan Assay ID
CLSTN2	GUGGUAACCUUAUACAUGG, GGAAAUCUGUGCGUUCAAG, GAAAUGAACCGGCAUCACU and GCACUACUAUGUCAUCAAU	Hs00223248_m1
CSMD3	AAUAAUAGCAGAAGAACGA, GGAUUAUAAAUUAGGGUCA, CGGAUAAGAUAUAUAGCUU and CGAAGUGGAUCCUCAGAAA	Hs00287451_m1
FOXO3	GCACAGAGUUGGAUGAAGU, GUACUCAACUAGUGCAAAC, CGAAUCAGCUGACGACAGU and UAACUUUGAUUCCCUCAUC	Hs00818121_m1
NAV3	GCUGUUAGCUCAGAUAUUU, CAGGGAGCCUCUAAUUUAA, GAGAGGGUCUUCAGAUGUA and GGACUUAACCUAUAUACUA	Hs00372108_m1
NCOA3	GCAAUGAUGUCGUCAAGAA, GAAUAAUGCACUUCUUAGA, GACAGGCACUUGAAUUGAA and AAAGCAAACUCUUCCGAAA	Hs01105251_m1
PTPRD	UUAAGGAGAUAUCUAGGAA, GAUGAUAUGCCAAUAGGAA, UCAGAGAUUUGAGGUAAUA and GAAUGGAGCUCGAAUUUAA	Hs00369913_m1
ROBO1	GGAUGUAUUUGCAACAAGA, GAGGGCAGCUAAUGCAUAU, GCAGGUACUUGGAGGAUAU and GAAUCAGACUGGUUAGUUU	Hs00268049_m1
ROCK2	CAAACUUGGUAAAGAAUUG, GAAACUAAUAGGACACUAA, UAGAAUAUGUGGCCUAGAA and GCAACUGGCUCGUUCAAUU	Hs00178154_m1
SEMA5A	GGCAAGAUCCAGUAGCGUA, GAGCAACGAUUCCGAUACA, UGGAGGAAAUAGUCGGAUA and CGGGUGUGCAAGAACGAUA	Hs01549381_m1
SIPA1L1	UGGAAAUGAUAUCGUAACA, GAUUUAUGGACACGAGAAA, AGACAAGACCGUCGGAGAA and CCGUACAACUACCGAAUAA	Hs00210194_m1
SND1	UCAUGGUGGAGGUGCGCAA, CGAGAGUUCCUUCGAAAGA, UGAUGGAGAACAUGCGCAA and GGAAGUCUGUUUCACGAUA	Hs00205182_m1
TCF7L2	GAUGUCGGCUCACUCCAUA, ACACUUACCAGCCGACGUA, CGAGACAAAUCCCGGGAAA and CAGCGAAUGUUUCCUAAAU	Hs01009044_m1
ZCCHC7	GAAGAUGGACCCAGCGGUA, GGAUAAACAGUGUGACCGA, UCACAUUACCACACGUCAA and CCAGAUAGCUAAUAACCGA	Hs00540261_g1
Non-targeting pool	UGGUUUACAUGUCGACUAA, UGGUUUACAUGUUGUGUGA, UGGUUUACAUGUUUUCUGA and UGGUUUACAUGUUUUCCUA	N/A
KRAS	GGAGGGCUUUCUUUGUGUA, UCAAAGACAAAGUGUGUAA, GAAGUUAUGGAAUUCCUUU and GAGAUAACACGAUGCGUAU	Hs00364284_g1
BRAF	CAUGAAGACCUCACAGUAA, UCAGUAAGGUACGGAGUAA, AGACGGGACUCGAGUGAUG and UUACCUGGCUCACUAACUA	Hs00269944_m1
ACTB	N/A	Hs01060665_g1 (VIC PL),<br>Hs01060665_g1 (FAM)
SLC2A1 (GLUT1)	N/A	Hs00892681_m1

table S7. Target sequences and TaqMan probes for siRNA mediated gene knock-down. SMARTpools of three siRNAs for each gene and TaqMan assays used for analysing gene expression levels.

table S8. GIPZ lentivirus clones for shRNA mediated gene knock-down

Gene	GIPZ shRNA lentiviral particle sets
BRAF	VGH5518-200207314 - V2LHS_240239<br>VGH5518-200238511 - V3LHS_355031<br>VGH5518-200234344 - V3LHS_355032
FOXO3	VGH5518-200278770 - V3LHS_375381<br>VGH5518-200223947 - V3LHS_641760<br>VGH5518-200228089 - V3LHS_641765
KRAS	VGH5518-200196451 - V2LHS_275818<br>VGH5518-200243083 - V3LHS_314004<br>VGH5518-200251719 - V3LHS_314006
NCOA3	VGH5518-200161516 - V2LHS_207128<br>VGH5518-200206608 - V2LHS_261936<br>VGH5518-200297500 - V3LHS_335962
TCF7L2	VGH5518-200206198 - V2LHS_116683<br>VGH5518-200257275 - V3LHS_312392<br>VGH5518-200257051 - V3LHS_312395

table S8. GIPZ lentivirus clones for shRNA mediated gene knock-down. GIPZ shRNA lentiviral particle sets for three validated genes (FOXO3, NCOA3 and TCF7L2) along with two known Ras pathway genes (KRAS and BRAF).

Table S9. Alterations in Ras pathway protein expression levels by knockdown of KRAS, FOXO3, NCOA3, and TCF7L2

Symbol	Protein name	Pathway(s)	Majority protein Ids	Significant changes (Student's T-test)
KBTBD7	Kelch repeat and BTB domain-containing protein 7	Ras signaling pathway - Homo sapiens (human)#path:hsa04014#KEGG	Q8WVZ9	KRASwt shNCOA3 vs shControl
BAD	Bcl2-associated agonist of cell death	Ras signaling pathway - Homo sapiens (human)#path:hsa04014#KEGG	Q92934	KRASwt shTCF7L2 vs shControl
CAMK2D	Calcium/calmodulin-dependent protein kinase type II subunit d	Ras activation uopn Ca2+ infux through NMDA receptor#R-HSA-442982#Reactome#CREB phosphorylation through the activation of Ras#R-HSA-442742#Reactome#Signalling to RAS#R-HSA-167044#Reactome	Q13557-12	KRAS G13D shFOXO3 vs shControl
FGF2	Fibroblast growth factor 2;Fibroblast growth factor	Ras signaling pathway - Homo sapiens (human)#path:hsa04014#KEGG#ras signaling pathway#raspathway#BioCarta#influence of ras and rho proteins on g1 to s transition#raccycdpathway#BioCarta	P09038;A0A087WUF6;P09038-2;P09038-3;P09038-1	KRAS G13D shKRAS vs shControl
LGALS1	Galectin-1	Ras signaling pathway - Homo sapiens (human)#path:hsa04014#KEGG	P09382	KRAS G13D shKRAS vs shControl
PLCG2	1-phosphatidylinositol 4,5-bisphosphate phosphodiesterase ga	Signalling to RAS#R-HSA-167044#Reactome	P16885;A0A0A0MRF9	KRAS G13D shKRAS vs shControl
CDKN1A	Cyclin-dependent kinase inhibitor 1	Ras signaling pathway - Homo sapiens (human)#path:hsa04014#KEGG	P38936	KRAS G13D shKRAS vs shControl; KRAS G13D shNCOA3 vs shControl
EPHA2	Ephrin type-A receptor 2	Signalling to RAS#R-HSA-167044#Reactome	P29317	KRAS G13D shNCOA3 vs shControl
ERBB2	Receptor tyrosine-protein kinase erbB-2;Receptor protein-tyros	Signalling to RAS#R-HSA-167044#Reactome	P04626;P04626-5;P04626-4;B4DTR1;J3QLU9;P04626-3;P04626-2	KRAS G13D shNCOA3 vs shControl
FYN	Tyrosine-protein kinase Fyn	ras-independent pathway in nk cell-mediated cytotoxicity#nkcellspathway#BioCarta	P06241;P06241-2;P06241-3	KRAS G13D shNCOA3 vs shControl
IRS2	Insulin receptor substrate 2	Ras signaling pathway - Homo sapiens (human)#path:hsa04014#KEGG#ras signaling pathway#raspathway#BioCarta	Q9Y4H2	KRAS G13D shNCOA3 vs shControl
RAB8A	Ras-related protein Rab-8A	Ras signaling pathway - Homo sapiens (human)#path:hsa04014#KEGG#ras signaling pathway#raspathway#BioCarta	P61006;P61006-2	KRAS G13D shNCOA3 vs shControl
ABL1	Tyrosine-protein kinase ABL1	Ras-Raf-MEK-Erk/JNK signalling#cBIOPORTAL (http://www.cbioportal.org/) in the COAD dataset#cBIOPORTAL (http://www.cbioportal.org/)	P00519;P00519-2	N/A
ABL2	Abelson tyrosine-protein kinase 2	Ras signaling pathway - Homo sapiens (human)#path:hsa04014#KEGG#Signalling to RAS#R-HSA-167044#Reactome	P42684;P42684-6;P42684-7;P42684-5;P42684-4;P42684-10;P42684-2;P42684-3	N/A
AKAP9	A-kinase anchor protein 9	Ras signaling pathway - Homo sapiens (human)#path:hsa04014#KEGG#Ras activation uopn Ca2+ infux through NMDA receptor#R-HSA-442982#Reactome#CREB phosphorylation through the activation of Ras#R-HSA-442742#Reactome#Signalling to RAS#R-HSA-167044#Reactome#Regulation of Ras family activation#ras_pathway#PID	Q99996;A0A0A0MRF6;Q99996-5;Q99996-3;Q99996-2;Q99996-6;A0A0A0MRE9	N/A
AKT1	RAC-alpha serine/threonine-protein kinase	Ras signaling pathway - Homo sapiens (human)#path:hsa04014#KEGG	P31749;P31749-2	N/A
AKT2	RAC-beta serine/threonine-protein kinase	Ras signaling pathway - Homo sapiens (human)#path:hsa04014#KEGG	P31751;M0R0P9;P31751-2	N/A
ARAF	Serine/threonine-protein kinase A-Raf	Ras activation uopn Ca2+ infux through NMDA receptor#R-HSA-442982#Reactome#CREB phosphorylation through the activation of Ras#R-HSA-442742#Reactome#Signalling to RAS#R-HSA-167044#Reactome	P10398;Q96II5	N/A
ARF6	ADP-ribosylation factor 6	Ras activation uopn Ca2+ infux through NMDA receptor#R-HSA-442982#Reactome#CREB phosphorylation through the activation of Ras#R-HSA-442742#Reactome#Signalling to RAS#R-HSA-167044#Reactome	P62330	N/A
ARRB1	Beta-arrestin-1	Ras signaling pathway - Homo sapiens (human)#path:hsa04014#KEGG#ras signaling pathway#raspathway#BioCarta	P49407-2;P49407;E9PM35	N/A
ARRB2;DKFZp686L0365	Beta-arrestin-2	influence of ras and rho proteins on g1 to s transition#raccycdpathway#BioCarta	P32121;P32121-5;P32121-2;P32121-3;P32121-4;Q68DZ5;K7ENA6	N/A
B2M	Beta-2-microglobulin;Beta-2-microglobulin form pI 5.3;Beta-2-n	influence of ras and rho proteins on g1 to s transition#raccycdpathway#BioCarta	P61769;H0YL18;F5H6I0	N/A
BCL2L1	Bcl-2-like protein 1	influence of ras and rho proteins on g1 to s transition#raccycdpathway#BioCarta	Q07817;Q5QP56;Q9H1R6;Q5TE64;Q07817-3;Q07817-2;Q5QP59	N/A
BRAF	Serine/threonine-protein kinase B-raf	influence of ras and rho proteins on g1 to s transition#raccycdpathway#BioCarta	P15056;H7C560	N/A
BRAP	BRCA1-associated protein	influence of ras and rho proteins on g1 to s transition#raccycdpathway#BioCarta	Q7Z569;J3KNN7;Q7Z569-2	N/A
CALM1;CALM2;CALM3	Calmodulin	Ras signaling pathway - Homo sapiens (human)#path:hsa04014#KEGG#ras signaling pathway#raspathway#BioCarta#influence of ras and rho proteins on g1 to s transition#raccycdpathway#BioCarta	P62158;H0Y7A7;E7ETZ0;E7EMB3;M0QZ52;F8WBR5;G3V479;Q96HY3	N/A
CALML3	Calmodulin-like protein 3	Signalling to RAS#R-HSA-167044#Reactome	P27482	N/A
CALML5	Calmodulin-like protein 5	CREB phosphorylation through the activation of Ras#R-HSA-442742#Reactome	Q9NZT1	N/A
CAMK2G	Calcium/calmodulin-dependent protein kinase type II subunit g	Signalling to RAS#R-HSA-167044#Reactome	A0A0A0MS52;Q13555-6;Q13555;Q13555-5;Q5SWX3;Q13555-2;A0A0A0MT11;Q13555-4;Q13555-8;Q13555-9;Q13555-10;Q13555-3;Q13555-7;Q8WU40;H0Y6G2	N/A
CDC42	Cell division control protein 42 homolog	Signalling to RAS#R-HSA-167044#Reactome#Regulation of RAS by GAPs#R-HSA-5658442#Reactome	P60953;P60953-1	N/A
CDK2	Cyclin-dependent kinase 2	Signalling to RAS#R-HSA-167044#Reactome#Regulation of RAS by GAPs#R-HSA-5658442#Reactome#Regulation of Ras family activation#ras_pathway#PID	P24941;G3V5T9;P24941-2;E7ESI2	N/A
CDK4	Cyclin-dependent kinase 4	Signalling to RAS#R-HSA-167044#Reactome	P11802;F8VYH9;F8VTV8;F8VWX7;F8W1L8;F8VZ51	N/A
CDK6	Cyclin-dependent kinase 6	Ras signaling pathway - Homo sapiens (human)#path:hsa04014#KEGG#Signalling to RAS#R-HSA-167044#Reactome	Q00534	N/A
CDKN1B	Cyclin-dependent kinase inhibitor 1B	Ras signaling pathway - Homo sapiens (human)#path:hsa04014#KEGG	P46527;E7ES52;H7C2T1	N/A
CHUK	Inhibitor of nuclear factor kappa-B kinase subunit alpha	Signalling to RAS#R-HSA-167044#Reactome	O15111	N/A
CNKSR1	Connector enhancer of kinase suppressor of ras 1	Signalling to RAS#R-HSA-167044#Reactome	Q969H4;Q969H4-2;G3V160	N/A
CREB1	Cyclic AMP-responsive element-binding protein 1	Ras signaling pathway - Homo sapiens (human)#path:hsa04014#KEGG	P16220;P16220-2;C9J896;P16220-3;C9JBT4	N/A
CSK	Tyrosine-protein kinase CSK	Ras signaling pathway - Homo sapiens (human)#path:hsa04014#KEGG#Signalling to RAS#R-HSA-167044#Reactome	P41240	N/A
CUL3	Cullin-3	Signalling to RAS#R-HSA-167044#Reactome	Q13618;Q13618-2;Q13618-3	N/A
DAB2IP	Disabled homolog 2-interacting protein	Ras signaling pathway - Homo sapiens (human)#path:hsa04014#KEGG	Q5VWQ8;H0Y3A3;Q5VWQ8-3;Q5VWQ8-4;Q5VWQ8-2;Q5VWQ8-5	N/A
DUSP9	Dual specificity protein phosphatase 9	Ras signaling pathway - Homo sapiens (human)#path:hsa04014#KEGG	Q99956	N/A
EGFR	Epidermal growth factor receptor;Receptor protein-tyrosine kin	Ras signaling pathway - Homo sapiens (human)#path:hsa04014#KEGG	P00533;E9PFD7;Q504U8	N/A
ERBB3	Receptor tyrosine-protein kinase erbB-3	Ras signaling pathway - Homo sapiens (human)#path:hsa04014#KEGG	P21860;P21860-4;F8VW56;F8VYK4;P21860-5;B3KWG5;B4DGQ7	N/A
EXOC2	Exocyst complex component 2	Ras signaling pathway - Homo sapiens (human)#path:hsa04014#KEGG	Q96KP1	N/A
GAB1	GRB2-associated-binding protein 1	Ras signaling pathway - Homo sapiens (human)#path:hsa04014#KEGG	Q13480;Q13480-2;B7Z3B9	N/A
GNB2;GNB4	Guanine nucleotide-binding protein G(I)/G(S)/G(T) subunit beta	Ras signaling pathway - Homo sapiens (human)#path:hsa04014#KEGG#Signalling to RAS#R-HSA-167044#Reactome#Regulation of Ras family activation#ras_pathway#PID	P62879;C9JZN1;C9JIS1;C9JXA5;Q9HAV0	N/A
GNG10	Guanine nucleotide-binding protein G(I)/G(S)/G(O) subunit gan	ras-independent pathway in nk cell-mediated cytotoxicity#nkcellspathway#BioCarta	P50151	N/A
GNG12	Guanine nucleotide-binding protein G(I)/G(S)/G(O) subunit gan	Ras signaling pathway - Homo sapiens (human)#path:hsa04014#KEGG#ras signaling pathway#raspathway#BioCarta#Ras activation uopn Ca2+ infux through NMDA receptor#R-HSA-442982#Reactome#CREB phosphorylation through the activation of Ras#R-HSA-442742#Reactome#Ras signaling in the CD4+ TCR pathway#tcrraspathway#PID#Signalling to RAS#R-HSA-167044#Reactome#Regulation of RAS by GAPs#R-HSA-5658442#Reactome#Activation of RAS in B cells#R-HSA-1169092#Reactome#influence of ras and rho proteins on g1 to s transition#raccycdpathway#BioCarta#Regulation of Ras family activation#ras_pathway#PID#Ras-Raf-MEK-Erk/JNK signalling#cBIOPORTAL (http://www.cbioportal.org/) in the COAD dataset#cBIOPORTAL (http://www.cbioportal.org/)	Q9UBI6	N/A
GNG5	Guanine nucleotide-binding protein G(I)/G(S)/G(O) subunit gan	RAS gene family#RAB amnd RABL gene family#HGNC	P63218	N/A
GNG7	Guanine nucleotide-binding protein G(I)/G(S)/G(O) subunit gan	Ras signaling pathway - Homo sapiens (human)#path:hsa04014#KEGG	O60262;K7ESC6	N/A
GRB2	Growth factor receptor-bound protein 2	Ras signaling pathway - Homo sapiens (human)#path:hsa04014#KEGG#influence of ras and rho proteins on g1 to s transition#raccycdpathway#BioCarta	P62993;P62993-2	N/A
HLA-A	HLA class I histocompatibility antigen, A-1 alpha chain;HLA cla	Ras signaling pathway - Homo sapiens (human)#path:hsa04014#KEGG#influence of ras and rho proteins on g1 to s transition#raccycdpathway#BioCarta	A0A0G2JIF2;A0A0G2JL56;P30443;P30455;A0A0G2JI36	N/A
HRAS	GTPase HRas;GTPase HRas, N-terminally processed	ras-independent pathway in nk cell-mediated cytotoxicity#nkcellspathway#BioCarta	P01112;P01112-2	N/A
IFT27	Intraflagellar transport protein 27 homolog	Ras signaling pathway - Homo sapiens (human)#path:hsa04014#KEGG	Q9BW83;Q9BW83-2;F5GZ09;B1AH58;H0Y6C7	N/A
IGF1R	Insulin-like growth factor 1 receptor;Insulin-like growth factor 1	Signalling to RAS#R-HSA-167044#Reactome	P08069;C9J5X1	N/A
IKBKB	Inhibitor of nuclear factor kappa-B kinase subunit beta	Signalling to RAS#R-HSA-167044#Reactome	O14920;O14920-4;O14920-2;G3V105	N/A
IKBKG	NF-kappa-B essential modulator	Signalling to RAS#R-HSA-167044#Reactome	Q9Y6K9;A0A087X0G7;A0A087WUW6;A0A087X1B1;Q9Y6K9-2;C9J2V2;C9JN51;A0A087WV30;C9JH59;Q9Y6K9-3	N/A
IL18	Interleukin-18	Signalling to RAS#R-HSA-167044#Reactome	Q14116;Q14116-2	N/A
INSR	Insulin receptor;Insulin receptor subunit alpha;Insulin receptor s	Ras signaling pathway - Homo sapiens (human)#path:hsa04014#KEGG	P06213;P06213-2	N/A
IQGAP1	Ras GTPase-activating-like protein IQGAP1	Signalling to RAS#R-HSA-167044#Reactome#Regulation of RAS by GAPs#R-HSA-5658442#Reactome	P46940;H0YLE8	N/A
IRS1	Insulin receptor substrate 1	Ras signaling pathway - Homo sapiens (human)#path:hsa04014#KEGG#Signalling to RAS#R-HSA-167044#Reactome	P35568	N/A
JAK1	Tyrosine-protein kinase JAK1	Ras signaling pathway - Homo sapiens (human)#path:hsa04014#KEGG#Ras signaling in the CD4+ TCR pathway#tcrraspathway#PID#Signalling to RAS#R-HSA-167044#Reactome#Regulation of RAS by GAPs#R-HSA-5658442#Reactome#Activation of RAS in B cells#R-HSA-1169092#Reactome#Regulation of Ras family activation#ras_pathway#PID#Ras-Raf-MEK-Erk/JNK signalling#cBIOPORTAL (http://www.cbioportal.org/) in the COAD dataset#cBIOPORTAL (http://www.cbioportal.org/)	P23458	N/A
JMJD7	JmjC domain-containing protein 7	Ras signaling pathway - Homo sapiens (human)#path:hsa04014#KEGG#Signalling to RAS#R-HSA-167044#Reactome	P0C870;P0C869-8;P0C869-7;P0C869-6;B5MC20	N/A
KIT	Mast/stem cell growth factor receptor Kit	Signalling to RAS#R-HSA-167044#Reactome	P10721;P10721-2	N/A
KRAS	GTPase KRas;GTPase KRas, N-terminally processed	Signalling to RAS#R-HSA-167044#Reactome	P01116-2	N/A
KSR1	Kinase suppressor of Ras 1	Regulation of Ras family activation#ras_pathway#PID	Q8IVT5;Q8IVT5-2;A0A0A0MQW1;Q8IVT5-4;Q8IVT5-3	N/A
LAMTOR2	Ragulator complex protein LAMTOR2	Regulation of Ras family activation#ras_pathway#PID	Q9Y2Q5;Q9Y2Q5-2;Q9Y2Q5-3	N/A
LAMTOR3	Ragulator complex protein LAMTOR3	Ras-Raf-MEK-Erk/JNK signalling#cBIOPORTAL (http://www.cbioportal.org/) in the COAD dataset#cBIOPORTAL (http://www.cbioportal.org/)	Q9UHA4;Q9UHA4-2	N/A
LGALS3	Galectin-3;Galectin	Ras-Raf-MEK-Erk/JNK signalling#cBIOPORTAL (http://www.cbioportal.org/) in the COAD dataset#cBIOPORTAL (http://www.cbioportal.org/)	P17931;G3V3R6	N/A
MAP2K1	Dual specificity mitogen-activated protein kinase kinase 1	Ras-Raf-MEK-Erk/JNK signalling#cBIOPORTAL (http://www.cbioportal.org/) in the COAD dataset#cBIOPORTAL (http://www.cbioportal.org/)	Q02750;Q02750-2	N/A
MAP2K2	Dual specificity mitogen-activated protein kinase kinase 2	Ras-Raf-MEK-Erk/JNK signalling#cBIOPORTAL (http://www.cbioportal.org/) in the COAD dataset#cBIOPORTAL (http://www.cbioportal.org/)	P36507;G5E9C7	N/A
MAP2K3	Dual specificity mitogen-activated protein kinase kinase 3	Signalling to RAS#R-HSA-167044#Reactome	P46734;P46734-2;P46734-3	N/A
MAP2K4	Dual specificity mitogen-activated protein kinase kinase 4	Ras-Raf-MEK-Erk/JNK signalling#cBIOPORTAL (http://www.cbioportal.org/) in the COAD dataset#cBIOPORTAL (http://www.cbioportal.org/)	P45985;P45985-2	N/A
MAP3K11	Mitogen-activated protein kinase kinase kinase 11	Ras-Raf-MEK-Erk/JNK signalling#cBIOPORTAL (http://www.cbioportal.org/) in the COAD dataset#cBIOPORTAL (http://www.cbioportal.org/)	Q16584;Q16584-2	N/A
MAP3K2	Mitogen-activated protein kinase kinase kinase 2	Ras-Raf-MEK-Erk/JNK signalling#cBIOPORTAL (http://www.cbioportal.org/) in the COAD dataset#cBIOPORTAL (http://www.cbioportal.org/)	Q9Y2U5	N/A
MAP3K4	Mitogen-activated protein kinase kinase kinase 4	Ras signaling pathway - Homo sapiens (human)#path:hsa04014#KEGG#CREB phosphorylation through the activation of Ras#R-HSA-442742#Reactome#Ras signaling in the CD4+ TCR pathway#tcrraspathway#PID#Signalling to RAS#R-HSA-167044#Reactome#influence of ras and rho proteins on g1 to s transition#raccycdpathway#BioCarta#Ras-Raf-MEK-Erk/JNK signalling#cBIOPORTAL (http://www.cbioportal.org/) in the COAD dataset#cBIOPORTAL (http://www.cbioportal.org/)	Q9Y6R4;J3KNB8;F5H538;Q9Y6R4-2;F5H4R1	N/A
MAP3K5	Mitogen-activated protein kinase kinase kinase 5	Ras-Raf-MEK-Erk/JNK signalling#cBIOPORTAL (http://www.cbioportal.org/) in the COAD dataset#cBIOPORTAL (http://www.cbioportal.org/)	Q99683;Q99683-2	N/A
MAPK1	Mitogen-activated protein kinase 1	Ras-Raf-MEK-Erk/JNK signalling#cBIOPORTAL (http://www.cbioportal.org/) in the COAD dataset#cBIOPORTAL (http://www.cbioportal.org/)	P28482;P28482-2	N/A
MAPK12	Mitogen-activated protein kinase 12	Ras-Raf-MEK-Erk/JNK signalling#cBIOPORTAL (http://www.cbioportal.org/) in the COAD dataset#cBIOPORTAL (http://www.cbioportal.org/)	P53778;P53778-2;B5MDL5	N/A
MAPK14	Mitogen-activated protein kinase 14	Ras-Raf-MEK-Erk/JNK signalling#cBIOPORTAL (http://www.cbioportal.org/) in the COAD dataset#cBIOPORTAL (http://www.cbioportal.org/)	Q16539;Q16539-3;B4E0K5;Q16539-5;Q16539-4	N/A
MAPK3	Mitogen-activated protein kinase 3;Mitogen-activated protein ki	Ras-Raf-MEK-Erk/JNK signalling#cBIOPORTAL (http://www.cbioportal.org/) in the COAD dataset#cBIOPORTAL (http://www.cbioportal.org/)	P27361;E9PQW4;P27361-3;E9PBK7;E9PJF0;P27361-2;B3KR49	N/A
MAPK7	Mitogen-activated protein kinase 7	Ras signaling pathway - Homo sapiens (human)#path:hsa04014#KEGG#Ras-Raf-MEK-Erk/JNK signalling#cBIOPORTAL (http://www.cbioportal.org/) in the COAD dataset#cBIOPORTAL (http://www.cbioportal.org/)	Q13164;Q13164-3;Q13164-2;C9JUK9;Q13164-4	N/A
MAPK8	Mitogen-activated protein kinase 8	Signalling to RAS#R-HSA-167044#Reactome	P45983-3;P45983-4;P45983;P45983-2;P45983-5;A6NF29	N/A
MAPK9	Mitogen-activated protein kinase 9	Signalling to RAS#R-HSA-167044#Reactome	P45984;P45984-3;P45984-2;P45984-4;J3KNK1;D7R525;P45984-5	N/A
MAPKAPK2	MAP kinase-activated protein kinase 2	Signalling to RAS#R-HSA-167044#Reactome	P49137;P49137-2	N/A
MAPKAPK3	MAP kinase-activated protein kinase 3	Ras signaling pathway - Homo sapiens (human)#path:hsa04014#KEGG	Q16644;C9J8E1	N/A
MARK3	MAP/microtubule affinity-regulating kinase 3	Ras signaling pathway - Homo sapiens (human)#path:hsa04014#KEGG	P27448;P27448-3;A0A0A0MSZ1;A0A0A0MT23;J3KNR0;P27448-4;P27448-2;A0A0A0MST9;P27448-8	N/A
MET	Hepatocyte growth factor receptor	Signalling to RAS#R-HSA-167044#Reactome	P08581;P08581-2	N/A
MLLT4	Afadin	Ras signaling pathway - Homo sapiens (human)#path:hsa04014#KEGG#Signalling to RAS#R-HSA-167044#Reactome#Regulation of RAS by GAPs#R-HSA-5658442#Reactome#Regulation of Ras family activation#ras_pathway#PID	P55196;P55196-5;J3KN01;Q5TIG5;P55196-6	N/A
NCAM1	Neural cell adhesion molecule 1	Ras signaling pathway - Homo sapiens (human)#path:hsa04014#KEGG	P13591;P13591-1;A0A087WWD4;A0A087WTF6;A0A087WX77;A0A087WV75;P13591-4	N/A
NF1	Neurofibromin;Neurofibromin truncated	influence of ras and rho proteins on g1 to s transition#raccycdpathway#BioCarta	P21359;P21359-2;P21359-6;H0Y465;J3KSB5;P21359-4	N/A
NFKB1	Nuclear factor NF-kappa-B p105 subunit;Nuclear factor NF-kap	Ras signaling pathway - Homo sapiens (human)#path:hsa04014#KEGG#ras-independent pathway in nk cell-mediated cytotoxicity#nkcellspathway#BioCarta#influence of ras and rho proteins on g1 to s transition#raccycdpathway#BioCarta	P19838;P19838-2;P19838-3	N/A
NFKBIA	NF-kappa-B inhibitor alpha	Ras signaling pathway - Homo sapiens (human)#path:hsa04014#KEGG	P25963;G3V3I4;G3V2A2	N/A
PAK1	Serine/threonine-protein kinase PAK 1;Non-specific serine/thre	Ras signaling pathway - Homo sapiens (human)#path:hsa04014#KEGG	Q13153;B3KNX7	N/A
PAK2	Serine/threonine-protein kinase PAK 2;PAK-2p27;PAK-2p34	Ras signaling pathway - Homo sapiens (human)#path:hsa04014#KEGG	Q13177	N/A
PAK4	Serine/threonine-protein kinase PAK 4	ras signaling pathway#raspathway#BioCarta#CREB phosphorylation through the activation of Ras#R-HSA-442742#Reactome#influence of ras and rho proteins on g1 to s transition#raccycdpathway#BioCarta	O96013;O96013-2;O96013-4;O96013-3	N/A
PAK6	Serine/threonine-protein kinase PAK 6	Signalling to RAS#R-HSA-167044#Reactome	Q9NQU5;Q9NQU5-2	N/A
PDPK1;PDPK2P	3-phosphoinositide-dependent protein kinase 1;Putative 3-phos	Signalling to RAS#R-HSA-167044#Reactome	O15530;E9PER6;O15530-3;O15530-4;O15530-2;Q6A1A2;O15530-5	N/A
PEA15	Astrocytic phosphoprotein PEA-15	Signalling to RAS#R-HSA-167044#Reactome	Q15121;Q15121-2;B1AKZ5	N/A
PEBP1	Phosphatidylethanolamine-binding protein 1;Hippocampal chol	Ras signaling pathway - Homo sapiens (human)#path:hsa04014#KEGG#ras-independent pathway in nk cell-mediated cytotoxicity#nkcellspathway#BioCarta#ras signaling pathway#raspathway#BioCarta#influence of ras and rho proteins on g1 to s transition#raccycdpathway#BioCarta	P30086	N/A

Symbol	DLD-1 (KRAS G13D) shControl								DLD-1 *KRASwt/-* shControl							
	shKRAS		shNCOA3		shFOXO3		shTCF7L2		shKRAS		shNCOA3		shFOXO3		shTCF7L2	
	-Log *P*	Log2 fold change	-Log *P*	Log2 fold change	-Log *P*	Log2 fold change	-Log *P*	Log2 fold change	-Log *P*	Log2 fold change	-Log *P*	Log2 fold change	-Log *P*	Log2 fold change	-Log *P*	Log2 fold change
KBTBD7	0,19	0,51	0,23	-0,67	0,30	0,79	0,08	-0,22	0,37	-0,81	2,43	-3,92	0,07	-0,12	0,30	-0,49
BAD	1,89	-0,51	0,65	-0,94	0,22	-0,27	1,60	-1,17	0,02	0,03	0,01	-0,01	1,15	-0,69	3,23	-0,49
CAMK2D	1,74	-0,29	0,13	0,02	3,46	-0,83	0,10	-0,01	0,12	0,03	0,86	-0,15	0,36	-0,22	1,10	0,23
FGF2	2,40	2,01	0,12	0,30	0,22	0,66	0,09	0,26	1,18	0,88	0,56	0,33	0,35	0,21	0,56	-0,92
LGALS1	1,98	-3,59	1,01	0,47	1,66	-1,02	0,36	-1,46	0,55	-0,18	0,71	0,37	0,66	0,39	1,02	-0,52
PLCG2	1,98	1,82	0,33	0,23	0,48	1,28	1,14	0,63	0,10	0,19	0,49	-0,23	0,19	0,24	0,08	0,04
CDKN1A	2,32	2,76	2,34	3,74	1,30	2,43	2,06	2,36	0,40	-1,01	0,10	-0,17	0,71	-0,54	0,20	0,53
EPHA2	0,91	-1,18	2,36	2,11	0,75	-0,29	2,13	-1,65	0,60	-0,59	0,03	0,03	1,32	0,52	0,74	-0,27
ERBB2	0,43	-0,30	3,24	-0,80	1,09	-0,39	0,66	-0,13	0,37	0,53	0,27	0,40	0,59	1,05	0,06	-0,12
FYN	1,53	-1,18	1,96	-4,67	0,38	-1,08	0,03	-0,06	0,55	-0,80	0,12	-0,18	0,09	-0,46	0,02	0,03
IRS2	1,56	0,53	2,58	1,35	0,04	0,12	0,58	0,61	1,32	0,78	0,54	0,35	0,19	0,10	0,46	0,23
RAB8A	1,64	-0,45	4,51	0,78	1,26	0,30	1,69	-0,34	0,41	-0,21	0,32	-0,13	0,31	-0,24	0,43	0,22
ABL1	1,85	-0,65	0,18	-1,15	0,95	0,46	0,86	-0,39	0,27	-1,00	0,05	0,32	0,69	2,48	0,46	-1,99
ABL2	0,59	-0,26	0,94	0,70	1,45	-0,93	0,38	-0,23	0,06	-0,04	0,45	-0,47	0,50	-0,42	0,04	0,05
AKAP9	1,67	0,20	1,03	-0,37	1,45	-0,28	0,35	0,07	0,10	0,02	0,91	0,18	0,24	-0,12	1,62	0,10
AKT1	1,58	-0,18	1,09	-0,61	2,43	0,05	0,55	-0,22	1,01	0,68	1,46	0,77	0,97	0,52	1,21	-0,76
AKT2	0,12	0,17	0,35	0,44	0,71	0,84	0,30	0,41	0,57	0,36	0,60	0,31	0,09	0,11	0,67	0,29
ARAF	0,18	0,13	0,59	0,21	0,09	-0,19	0,81	0,66	0,09	0,16	0,19	0,35	0,04	0,08	0,22	-0,42
ARF6	0,07	0,05	0,25	0,15	0,33	-0,20	0,26	0,16	0,24	-0,09	0,14	-0,08	0,11	0,05	0,15	0,07
ARRB1	0,36	0,17	0,80	-0,57	0,27	0,15	0,81	0,42	0,14	0,20	0,68	-0,31	1,01	-0,32	0,42	0,27
ARRB2;DKFZp686L0365	1,23	1,09	1,03	-0,63	1,97	0,64	0,34	-0,22	1,04	0,37	0,90	0,24	0,23	-0,07	0,15	-0,04
B2M	0,15	-0,52	0,50	-1,38	0,56	-1,60	0,02	0,08	1,07	0,62	0,66	0,30	0,73	-0,46	0,12	-0,06
BCL2L1	0,63	0,79	0,50	0,92	0,31	0,42	0,12	-0,25	0,21	-0,23	0,15	0,16	0,02	-0,04	0,36	-0,34
BRAF	1,09	-0,49	0,44	-0,63	0,31	-0,81	0,41	-0,16	0,16	0,25	0,14	0,20	0,17	0,14	0,40	0,19
BRAP	0,02	0,08	0,05	-0,22	0,12	0,87	0,06	-0,23	0,31	0,84	0,41	1,05	0,58	1,43	0,27	-0,70
CALM1;CALM2;CALM3	0,28	-0,15	0,32	0,07	0,64	-0,11	0,21	-0,05	0,65	0,17	0,22	0,06	0,57	0,22	0,39	-0,11
CALML3	0,01	-0,05	0,32	-0,60	0,08	-0,38	0,74	-2,38	0,28	-1,69	0,35	-2,02	0,05	0,49	0,14	0,89
CALML5	0,05	-0,15	0,36	2,55	0,23	-1,15	0,42	-1,43	0,25	1,05	0,02	0,15	0,47	4,92	0,17	0,71
CAMK2G	0,19	0,19	1,00	0,86	0,13	-0,23	0,75	0,61	0,98	0,27	1,57	0,69	0,45	-0,16	0,06	0,04
CDC42	0,19	0,12	0,17	0,14	0,48	-0,29	0,34	-0,22	0,24	-0,13	0,73	-0,48	1,19	-0,83	0,28	0,23
CDK2	0,96	-0,44	0,59	-0,28	1,27	0,54	0,70	-0,61	1,39	0,36	2,54	0,40	1,94	0,60	1,76	-0,65
CDK4	1,09	1,23	0,07	0,08	1,45	1,80	0,91	0,94	0,45	0,43	0,12	-0,13	0,27	0,44	0,53	-0,84
CDK6	0,15	-0,26	1,08	-1,03	1,07	0,58	1,24	-0,64	0,30	1,47	0,09	0,59	0,13	0,71	0,29	1,52
CDKN1B	0,91	-0,51	1,15	-0,58	0,95	-0,81	0,23	-0,10	0,44	-0,52	0,66	-0,32	1,26	-1,37	1,85	1,53
CHUK	0,13	-0,07	0,34	0,16	0,23	-0,44	0,13	-0,22	0,10	-0,19	0,32	0,41	0,49	-0,65	0,01	0,03
CNKSR1	0,16	0,19	0,70	-1,04	0,22	-0,54	0,75	-0,75	0,59	-0,24	0,01	0,00	0,07	-0,04	0,86	-0,24
CREB1	0,21	0,37	0,23	0,40	0,19	0,37	0,34	0,59	1,33	-0,41	0,43	-2,41	1,11	-1,66	0,34	0,63
CSK	0,63	0,32	0,46	-0,23	0,12	0,14	0,70	0,37	0,93	0,17	0,31	0,07	0,26	-0,31	0,32	0,39
CUL3	0,21	-0,03	1,16	0,20	0,72	-0,26	0,86	-0,14	0,94	-0,26	0,15	0,03	1,11	-0,32	0,89	0,41
DAB2IP	0,45	-0,81	0,68	-2,95	0,15	0,59	0,64	1,68	1,09	0,26	0,77	0,35	1,26	0,39	0,47	-0,38
DUSP9	0,29	0,86	0,46	1,61	0,52	1,51	0,05	-0,17	0,67	0,70	0,45	0,89	0,90	1,17	1,42	-0,63
EGFR	1,40	-0,76	0,30	0,24	0,59	-0,25	2,44	-0,65	0,02	-0,01	1,33	0,12	0,90	-0,27	0,19	-0,36
ERBB3	0,04	-0,05	0,60	-0,46	0,95	-0,73	0,17	-0,17	0,27	-0,83	0,48	1,00	0,01	-0,02	0,03	0,09
EXOC2	1,78	0,25	0,00	0,00	0,81	-0,37	0,21	-0,06	0,25	-0,13	0,50	-0,27	0,76	-0,36	0,78	0,32
GAB1	0,10	-0,45	0,13	-0,34	0,58	1,29	0,34	0,77	0,08	0,82	0,34	-2,22	0,06	0,69	0,07	0,97
GNB2;GNB4	0,41	-0,10	0,56	-0,10	0,00	0,00	0,28	-0,09	0,59	0,32	0,13	0,08	0,14	0,09	0,64	-0,15
GNG10	0,66	0,40	1,31	-0,93	0,19	-0,09	0,97	0,50	0,94	0,25	0,73	-0,11	0,11	-0,02	0,21	-0,04
GNG12	0,47	0,44	0,25	0,18	0,59	0,40	0,59	0,41	2,48	0,53	0,69	0,21	1,12	0,37	0,00	0,00
GNG5	0,29	0,22	0,47	-0,13	0,61	0,46	0,80	0,63	0,30	0,43	0,57	0,98	0,01	-0,02	0,52	-0,70
GNG7	0,07	0,25	0,24	0,96	0,09	-0,35	0,05	0,24	0,49	-0,47	0,65	-0,40	0,58	-0,36	0,28	0,19
GRB2	1,61	-0,51	0,65	0,13	1,38	-0,83	0,66	-0,30	0,01	0,01	0,23	-0,22	0,67	-0,52	0,08	-0,07
HLA-A	1,61	0,67	0,91	0,27	0,30	0,11	1,58	0,71	1,25	0,71	1,68	0,50	0,47	-0,13	0,32	-0,06
HRAS	0,27	2,33	0,01	0,14	0,33	2,83	0,22	1,94	0,01	0,01	0,53	0,58	0,01	0,04	0,26	0,32
IFT27	0,41	-0,46	1,21	-1,78	1,10	0,99	0,91	0,76	0,08	0,11	0,29	-0,40	0,16	-0,22	0,26	0,42
IGF1R	1,61	0,93	0,03	-0,01	2,78	0,42	0,22	-0,19	0,27	-0,17	0,08	-0,06	0,53	-1,27	1,09	1,22
IKBKB	0,82	0,58	0,18	-0,17	0,30	-0,31	2,86	0,63	0,24	0,23	0,16	-0,18	0,32	0,36	0,13	-0,11
IKBKG	0,32	-0,64	0,74	-0,83	0,65	0,63	0,27	-0,19	0,35	1,40	0,61	2,28	0,44	1,84	0,41	-1,94
IL18	0,31	0,18	1,30	0,71	0,10	0,09	0,29	-0,13	0,02	0,03	0,04	-0,07	0,01	0,01	0,07	0,12
INSR	0,14	-0,09	0,37	0,43	0,64	-2,76	0,69	-1,77	0,25	-0,07	0,57	0,14	0,50	-0,67	0,38	0,21
IQGAP1	0,42	0,04	1,21	-0,17	1,02	-0,27	0,74	0,10	0,41	0,04	0,16	0,02	0,90	0,08	1,08	-0,13
IRS1	1,19	-0,35	0,52	-0,30	1,59	0,45	0,08	-0,02	0,12	-0,08	0,07	0,06	0,26	0,18	0,09	-0,09
JAK1	0,46	0,61	0,70	0,98	0,46	0,60	0,28	0,38	0,28	0,27	1,36	-0,77	1,08	-0,26	0,86	0,19
JMJD7	1,63	1,31	0,62	-0,43	0,60	-1,11	0,44	-0,26	0,35	-0,26	0,52	0,38	0,40	0,30	0,11	-0,09
KIT	0,59	0,97	0,33	0,46	0,85	1,24	0,15	0,22	0,15	0,26	0,30	0,50	0,18	-1,02	1,33	2,68
KRAS	0,93	-0,40	0,65	0,28	0,71	-0,42	0,78	-0,37	0,01	0,02	0,01	0,01	0,47	-0,57	0,14	-0,16
KSR1	0,52	1,82	0,45	1,37	0,62	1,73	0,35	1,61	0,60	-0,29	0,44	-1,17	0,29	-1,67	0,17	0,12
LAMTOR2	0,17	0,10	0,15	0,17	0,20	-0,19	0,28	0,20	0,02	0,02	0,19	-0,16	0,08	0,06	0,27	0,29
LAMTOR3	0,53	-0,17	0,67	-0,25	0,17	-0,08	0,03	0,03	0,28	-0,13	1,18	-0,70	0,58	-0,49	0,45	0,40
LGALS3	0,11	0,03	0,23	-0,08	0,63	0,18	1,25	0,37	0,34	-0,23	0,35	0,07	0,94	-0,15	0,96	0,13
MAP2K1	0,54	-0,13	0,63	0,10	0,21	-0,10	1,00	-0,26	0,21	-0,15	0,37	-0,29	0,52	-0,17	0,21	0,10
MAP2K2	0,40	0,18	0,44	-0,17	0,04	0,02	0,57	0,21	1,38	-0,04	0,57	-0,05	0,45	0,03	0,54	0,10
MAP2K3	0,25	-0,20	0,13	-0,09	0,36	-0,46	0,44	-0,27	0,26	0,07	2,36	-0,84	0,35	-0,50	0,80	0,22
MAP2K4	0,35	-0,35	0,09	-0,10	0,37	-0,35	0,16	-0,17	0,15	-0,14	0,28	-0,33	0,45	-0,33	0,15	-0,13
MAP3K11	0,02	0,04	0,00	-0,01	0,04	-0,12	0,31	-0,74	0,27	0,43	1,06	1,43	0,10	0,18	0,50	-0,88
MAP3K2	0,47	-0,41	1,39	0,79	0,96	-0,55	0,16	0,12	0,21	-0,37	0,46	-0,32	1,19	-0,64	0,09	0,20
MAP3K4	0,59	-1,07	1,36	-1,49	0,20	-0,06	0,82	-0,96	0,19	-0,23	0,23	0,30	0,40	0,45	0,61	-0,71
MAP3K5	0,01	0,01	0,66	-0,67	0,12	-0,19	0,47	-0,43	0,73	2,23	0,68	2,32	0,70	2,09	0,92	-3,21
MAPK1	0,46	0,12	1,26	-0,62	0,86	0,25	0,77	0,24	0,10	-0,07	0,27	0,18	0,17	-0,11	0,33	0,21
MAPK12	0,34	3,49	1,35	1,52	0,84	1,73	0,48	-0,41	0,57	0,22	0,16	0,24	0,34	0,13	0,34	-0,44
MAPK14	1,41	0,26	0,74	-0,19	0,75	0,13	1,93	0,47	0,50	0,08	0,48	-0,34	0,07	-0,03	1,60	-0,39
MAPK3	0,23	-0,05	1,24	-0,95	1,28	-0,30	0,47	-0,08	0,11	0,08	0,10	-0,18	0,11	-0,12	0,54	-0,36
MAPK7	0,02	0,03	0,59	-0,90	0,12	0,16	0,49	1,10	0,44	0,78	0,28	0,34	0,99	1,00	0,57	-0,57
MAPK8	0,73	-0,63	0,11	-0,11	0,22	-0,21	0,27	-0,25	0,71	0,13	0,21	0,10	0,14	0,06	0,76	0,17
MAPK9	0,04	-0,05	0,11	0,11	0,43	-0,47	0,01	0,01	0,40	-0,10	0,67	-0,45	0,62	-0,29	0,53	0,88
MAPKAPK2	0,20	0,18	1,46	1,67	1,68	2,57	1,59	1,85	1,76	1,03	0,59	0,76	1,60	0,80	0,18	0,11
MAPKAPK3	0,44	0,49	0,24	-0,43	0,56	0,60	0,03	0,04	0,03	0,06	0,02	-0,03	0,30	0,42	0,25	-0,33
MARK3	1,11	-0,33	0,23	0,13	0,25	-0,19	0,80	-0,70	0,04	-0,05	0,13	0,10	0,21	-0,26	0,20	-0,16
MET	0,54	-0,70	0,82	-0,95	0,08	-0,05	0,34	-0,25	0,60	0,20	1,02	0,37	0,35	0,28	0,71	-0,26
MLLT4	0,06	-0,02	0,47	-0,12	0,50	-0,17	0,02	0,02	0,61	0,15	0,60	0,15	1,42	-0,45	1,34	0,42
NCAM1	0,08	0,25	0,08	-0,19	0,12	0,27	0,40	0,90	1,09	0,57	0,89	0,37	0,86	-1,02	0,67	0,26
NF1	0,74	-0,25	1,11	-0,76	0,16	0,11	0,89	0,21	0,39	0,67	0,18	0,50	0,20	0,37	0,26	0,66
NFKB1	0,16	-0,14	0,46	-0,55	0,16	-0,21	0,55	-0,47	0,48	-0,24	2,00	-0,71	0,41	-0,30	0,02	0,01
NFKBIA	0,74	0,71	0,04	-0,03	0,13	0,21	1,20	0,82	0,03	-0,15	0,03	-0,16	0,75	-2,55	1,07	2,44
PAK1	0,22	0,04	0,36	-0,21	0,33	-0,31	1,05	0,32	0,20	0,19	0,32	0,26	0,38	-0,37	0,39	0,41
PAK2	0,03	0,02	0,01	0,00	0,31	0,09	0,04	0,02	0,54	-0,13	0,40	-0,10	0,75	-0,32	0,30	0,08
PAK4	0,35	-0,08	1,40	0,46	0,12	-0,07	0,21	-0,12	0,16	-0,24	0,61	0,34	0,72	0,40	0,41	-0,30
PAK6	0,40	-0,59	0,41	0,32	0,85	0,67	1,06	-0,93	0,10	-0,16	0,18	-0,24	0,09	-0,25	0,38	0,64
PDPK1;PDPK2P	0,11	0,22	0,56	-0,32	2,12	0,76	0,13	-0,20	0,39	0,39	0,20	0,21	0,35	0,40	0,39	0,60
PEA15	0,27	0,11	1,23	0,54	0,37	0,65	1,01	-0,58	0,12	0,19	0,21	0,31	0,06	-0,10	0,47	-0,63
PEBP1	0,65	0,13	0,55	-0,12	1,86	-0,17	1,14	0,30	0,81	0,25	0,61	0,25	1,55	0,25	1,01	-0,16

Table S9. Alterations in Ras pathway protein expression levels by knockdown of KRAS, FOXO3, NCOA3, and TCF7L2

Symbol	Protein name	Pathway(s)	Majority protein Ids	Significant changes (Student's T-test)	DLD-1 (KRAS G13D) shControl								DLD-1 *KRASwt/-* shControl							
					shKRAS		shNCOA3		shFOXO3		shTCF7L2		shKRAS		shNCOA3		shFOXO3		shTCF7L2	
					-Log *P*	Log2 fold change	-Log *P*	Log2 fold change	-Log *P*	Log2 fold change	-Log *P*	Log2 fold change	-Log *P*	Log2 fold change	-Log *P*	Log2 fold change	-Log *P*	Log2 fold change	-Log *P*	Log2 fold change
PHB	Prohibitin	Ras signaling pathway - Homo sapiens (human)#path:hsa04014#KEGG	P35232;C9JW96;E7ESE2;E9PCW0;C9JZ20	N/A	0,00	0,00	0,26	0,15	0,02	0,01	0,03	0,02	0,02	0,02	0,32	0,15	0,46	0,23	0,04	-0,04
PIK3CA	Phosphatidylinositol 4,5-bisphosphate 3-kinase catalytic subun	Ras signaling pathway - Homo sapiens (human)#path:hsa04014#KEGG	P42336	N/A	2,15	-0,58	0,39	-0,33	0,74	-0,48	0,99	-0,24	0,34	0,30	0,47	0,76	0,29	0,28	0,01	-0,02
PIK3CB;DKFZp779K1237	Phosphatidylinositol 4,5-bisphosphate 3-kinase catalytic subun	Ras signaling pathway - Homo sapiens (human)#path:hsa04014#KEGG	P42338;Q68DL0;H0Y871	N/A	0,07	-0,05	0,03	-0,03	2,05	-1,03	0,55	-0,25	0,66	-0,49	0,77	-0,59	0,86	-0,68	0,27	0,30
PIK3R1	Phosphatidylinositol 3-kinase regulatory subunit alpha	Ras signaling pathway - Homo sapiens (human)#path:hsa04014#KEGG	P27986;P27986-4;P27986-3;P27986-2;P27986-5	N/A	0,06	0,04	0,58	-0,29	0,40	0,25	1,10	0,51	0,11	0,08	0,27	-0,17	0,87	-0,57	0,40	0,33
PIK3R2	Phosphatidylinositol 3-kinase regulatory subunit beta	Ras signaling pathway - Homo sapiens (human)#path:hsa04014#KEGG	O00459;E9PFP1	N/A	0,54	0,22	0,02	-0,01	0,59	-0,25	0,21	-0,09	0,64	0,27	0,10	-0,05	1,07	0,51	0,43	-0,36
PLA2G4A	Cytosolic phospholipase A2;Phospholipase A2;Lysophospholip	Ras signaling pathway - Homo sapiens (human)#path:hsa04014#KEGG	P47712	N/A	0,02	-0,05	0,10	-0,25	0,24	0,70	0,14	0,92	0,66	1,27	0,74	1,36	0,29	0,54	0,64	-1,17
PLA2G6	85/88 kDa calcium-independent phospholipase A2	Ras signaling pathway - Homo sapiens (human)#path:hsa04014#KEGG	O60733;O60733-2;F8WEQ9;F2Z3G2;F8WEN3;B0QYE9;O60733-4;O60733-3	N/A	0,43	0,99	0,34	-0,82	0,19	-0,40	0,64	1,26	0,21	0,71	0,20	0,51	0,01	-0,04	0,22	-0,57
PLCG1	1-phosphatidylinositol 4,5-bisphosphate phosphodiesterase ga	Ras signaling pathway - Homo sapiens (human)#path:hsa04014#KEGG#ras signaling pathway#raspathway#BioCarta	P19174;P19174-2	N/A	0,22	0,09	0,21	-0,08	0,59	0,22	0,09	-0,04	0,27	0,14	0,36	0,19	0,55	0,26	0,07	-0,04
PLD1	Phospholipase D1	Ras signaling pathway - Homo sapiens (human)#path:hsa04014#KEGG	Q13393;Q13393-2;Q13393-4	N/A	0,07	-0,06	0,15	-0,14	0,25	-0,21	1,02	-1,02	1,72	2,32	0,19	-0,35	0,28	0,19	0,64	1,11
PLD2	Phospholipase D2	Signalling to RAS#R-HSA-167044#Reactome	O14939;O14939-4	N/A	1,77	0,80	2,09	-1,20	0,28	0,16	0,00	0,01	0,85	0,40	0,63	0,27	0,58	0,20	0,05	-0,02
PPP2CA	Serine/threonine-protein phosphatase 2A catalytic subunit alph	Signalling to RAS#R-HSA-167044#Reactome	P67775;P67775-2	N/A	0,13	-0,10	0,43	-0,26	0,42	0,22	0,33	-0,18	0,27	0,33	0,23	0,29	0,35	0,41	0,02	-0,03
PPP2R1A	Serine/threonine-protein phosphatase 2A 65 kDa regulatory su	Signalling to RAS#R-HSA-167044#Reactome	P30153;B3KQV6	N/A	2,56	-0,20	0,90	-0,16	0,93	-0,19	0,48	-0,11	0,77	-0,13	1,88	-0,30	0,34	-0,24	0,18	-0,05
PPP2R1B	Serine/threonine-protein phosphatase 2A 65 kDa regulatory su	Signalling to RAS#R-HSA-167044#Reactome	P30154;P30154-2;P30154-5;P30154-4;P30154-3	N/A	1,02	-0,29	0,58	0,14	1,15	0,33	0,83	-0,22	0,41	-0,89	0,69	-1,33	1,49	-1,90	1,14	1,57
PPP2R5A	Serine/threonine-protein phosphatase 2A 56 kDa regulatory su	Signalling to RAS#R-HSA-167044#Reactome	Q15172;Q15172-2	N/A	0,09	-0,07	0,62	-0,38	0,30	-0,19	0,07	0,05	0,09	0,17	1,54	0,59	0,46	-0,16	0,46	-0,30
PPP2R5B	Serine/threonine-protein phosphatase 2A 56 kDa regulatory su	Signalling to RAS#R-HSA-167044#Reactome	Q15173;Q15173-2;E9PNY3	N/A	0,76	-1,37	0,43	0,66	0,84	-1,45	0,97	-1,60	0,96	0,36	0,84	-0,43	0,25	-0,48	0,20	0,17
PPP2R5C	Serine/threonine-protein phosphatase 2A 56 kDa regulatory su	Signalling to RAS#R-HSA-167044#Reactome	Q13362-4;Q13362;Q13362-5;Q13362-3;H0YJ75;Q13362-2;Q96B13	N/A	0,89	-0,80	0,25	-0,38	0,63	0,59	0,91	0,50	0,43	-0,33	0,26	-0,11	0,34	-1,19	1,32	0,68
PPP2R5D	Serine/threonine-protein phosphatase 2A 56 kDa regulatory su	Signalling to RAS#R-HSA-167044#Reactome	Q14738;E9PFR3;Q14738-2;H0Y8C4;Q14738-3	N/A	0,85	0,23	0,33	-0,08	1,15	0,37	0,39	0,17	0,45	0,09	0,38	0,18	0,79	0,16	0,89	-0,21
PPP2R5E	Serine/threonine-protein phosphatase 2A 56 kDa regulatory su	Signalling to RAS#R-HSA-167044#Reactome	Q16537;Q16537-2;Q16537-3	N/A	0,08	-0,04	0,68	-0,21	1,57	0,52	1,12	0,47	0,02	0,01	0,20	-0,12	0,11	0,10	0,71	-0,37
PPP5C	Serine/threonine-protein phosphatase 5;Serine/threonine-prote	Ras signaling pathway - Homo sapiens (human)#path:hsa04014#KEGG	P53041;H0YDU8;A8MU39	N/A	0,47	-0,16	1,33	-0,67	0,71	-0,30	0,79	-0,29	0,67	-0,17	0,27	-0,12	0,14	-0,11	0,09	0,06
PRKACA;KIN27	cAMP-dependent protein kinase catalytic subunit alpha	Ras signaling pathway - Homo sapiens (human)#path:hsa04014#KEGG	P17612;P17612-2;Q15136;K7ERP6	N/A	0,62	-0,52	0,34	-0,22	0,66	-0,93	0,93	-0,54	0,61	0,74	1,05	0,89	0,16	0,17	0,13	-0,16
PRKACB	cAMP-dependent protein kinase catalytic subunit beta	Ras signaling pathway - Homo sapiens (human)#path:hsa04014#KEGG#Ras signaling in the CD4+ TCR pathway#tcrraspathway#PID#Regulation of Ras family activation#ras_pathway#PID	P22694;P22694-2;A0A0A0MS54;P22694-4;P22694-3;P22694-5;P22694-7;P22694-6;P22694-9;P22694-10;A0A087WVC4;B1APG1;B1APG0;B1APG2;B1APF8;B1APF9;B1APG3;P22694-8;B1APF7	N/A	0,24	0,09	0,04	-0,02	0,06	-0,07	0,23	-0,09	0,05	-0,03	0,16	0,11	0,36	0,11	1,83	-0,29
PRKCA	Protein kinase C alpha type	Regulation of Ras family activation#ras_pathway#PID	P17252;J3KRN5	N/A	0,63	0,47	0,79	0,52	0,68	-0,50	0,52	-1,48	0,29	-0,33	0,72	-0,73	0,03	-0,04	0,32	-0,17
PRKCZ	Protein kinase C zeta type	Signalling to RAS#R-HSA-167044#Reactome	Q05513;Q05513-3;E9PBE1;Q05513-2	N/A	0,51	-1,06	0,52	-1,07	0,71	-1,67	0,44	-0,92	0,34	0,80	0,24	-0,17	0,01	-0,01	1,57	1,65
PTK2	Focal adhesion kinase 1	Ras signaling pathway - Homo sapiens (human)#path:hsa04014#KEGG	Q05397;Q05397-5;Q05397-7;H0YBP1;E7ESA6;Q05397-2;H0YB16;H0YBZ1;E9PEI4	N/A	0,29	0,07	0,34	-0,05	0,96	-0,55	0,32	0,07	0,28	-0,12	0,28	0,09	0,07	0,03	0,19	0,06
PTPN11	Tyrosine-protein phosphatase non-receptor type 11	ras-independent pathway in nk cell-mediated cytotoxicity#nkcellspathway#BioCarta	Q06124;Q06124-2;Q06124-3	N/A	0,77	-0,15	0,16	0,03	0,97	-0,43	0,78	-0,16	0,07	-0,03	0,61	-0,24	0,50	-0,16	0,16	-0,06
PTPN6	Tyrosine-protein phosphatase non-receptor type 6	Signalling to RAS#R-HSA-167044#Reactome	P29350-3;P29350;P29350-4;P29350-2	N/A	0,20	0,21	0,67	-0,52	0,00	0,00	0,25	0,20	1,27	0,31	0,23	-0,17	0,22	0,26	0,89	-0,45
PTPRA	Receptor-type tyrosine-protein phosphatase alpha	RAS gene family#RAB amnd RABL gene family#HGNC	P18433;P18433-2;P18433-4;P18433-3	N/A	0,46	0,40	0,35	-0,22	1,62	-1,23	0,50	-0,37	0,54	0,32	0,38	0,21	0,43	0,48	0,17	0,12
RAB10	Ras-related protein Rab-10	RAS gene family#RAB amnd RABL gene family#HGNC	P61026	N/A	0,25	-0,17	0,25	-0,17	0,43	-0,29	0,13	-0,09	1,51	-0,45	0,56	-0,33	0,41	-0,27	0,06	0,02
RAB11A	Ras-related protein Rab-11A	RAS gene family#RAB amnd RABL gene family#HGNC	P62491;H3BSC1;B4DQU5	N/A	0,30	0,67	0,14	0,33	0,02	-0,07	0,69	1,55	0,83	1,22	0,49	0,80	0,10	0,31	0,46	-0,59
RAB11B;RAB11A	Ras-related protein Rab-11B;Ras-related protein Rab-11A	RAS gene family#RAB amnd RABL gene family#HGNC	Q15907;Q15907-2;P62491-2;H3BMH2	N/A	1,47	0,32	0,10	-0,02	0,06	0,03	1,77	0,59	0,47	0,18	0,23	0,11	0,85	0,22	0,76	-0,18
RAB12	Ras-related protein Rab-12	RAS gene family#RAB amnd RABL gene family#HGNC	Q6IQ22	N/A	0,23	-0,62	1,21	1,01	1,44	0,84	1,59	-1,26	0,44	-0,32	0,26	-0,15	0,29	0,26	0,16	0,12
RAB13	Ras-related protein Rab-13	RAS gene family#RAB amnd RABL gene family#HGNC	P51153	N/A	2,38	-0,38	0,98	-0,77	1,39	0,15	0,45	-0,15	0,05	0,03	0,53	-0,25	0,61	-0,57	0,25	-0,12
RAB14	Ras-related protein Rab-14	RAS gene family#RAB amnd RABL gene family#HGNC	P61106;X6RFL8	N/A	0,54	0,29	0,39	0,22	0,33	-0,18	0,33	0,17	0,04	0,03	0,14	-0,10	0,11	-0,09	0,03	0,02
RAB17	Ras-related protein Rab-17	RAS gene family#RAB amnd RABL gene family#HGNC	Q9H0T7;C9J0T6	N/A	0,21	-0,12	0,11	0,04	2,01	-0,58	0,04	-0,06	1,09	-0,55	1,31	-0,88	0,19	0,83	0,08	-0,25
RAB18	Ras-related protein Rab-18	RAS gene family#RAB amnd RABL gene family#HGNC	Q9NP72;Q9NP72-2;A0A087X163;Q5W0J0;H0Y6T8;Q9NP72-3	N/A	1,78	-0,32	0,03	0,01	0,37	-0,15	1,96	-0,51	0,30	0,37	0,13	-0,27	0,04	0,04	0,10	0,10
RAB19	Ras-related protein Rab-19	RAS gene family#RAB amnd RABL gene family#HGNC	A4D1S5;A4D1S5-2;C9JJQ5	N/A	1,25	0,82	1,05	-1,17	0,12	-0,20	0,66	0,52	1,32	0,91	0,37	0,19	0,95	-0,87	0,03	0,09
RAB1A	Ras-related protein Rab-1A	RAS gene family#RAB amnd RABL gene family#HGNC	P62820;E7END7;P62820-2;P62820-3	N/A	0,92	-0,15	1,82	0,20	0,12	0,08	0,80	-0,14	1,02	-0,25	1,15	-0,33	0,66	-0,33	0,43	0,13
RAB1B	Ras-related protein Rab-1B	RAS gene family#RAB amnd RABL gene family#HGNC	Q9H0U4;E9PLD0;A0A087WTI1	N/A	0,00	0,00	0,31	0,18	0,35	-0,22	0,04	0,03	0,27	-0,06	0,27	0,24	0,94	-0,34	0,27	0,14
RAB20	Ras-related protein Rab-20	RAS gene family#RAB amnd RABL gene family#HGNC	Q9NX57	N/A	0,48	0,19	0,22	-0,16	1,41	0,71	0,20	-0,23	1,95	0,34	1,18	0,62	1,39	0,67	0,21	-0,34
RAB21	Ras-related protein Rab-21	RAS gene family#RAB amnd RABL gene family#HGNC	Q9UL25	N/A	0,51	0,16	0,94	0,45	0,69	-0,21	0,36	-0,36	0,01	0,01	0,29	0,26	0,07	-0,06	0,09	0,05
RAB22A	Ras-related protein Rab-22A	RAS gene family#RAB amnd RABL gene family#HGNC	Q9UL26	N/A	0,10	-0,14	0,22	-0,35	0,42	-0,37	0,80	-1,15	0,96	-0,16	0,34	-1,36	0,38	-2,40	0,69	-0,09
RAB23	Ras-related protein Rab-23	RAS gene family#RAB amnd RABL gene family#HGNC	Q9ULC3	N/A	0,01	-0,01	0,90	0,47	0,23	-0,12	0,14	-0,07	0,96	-0,13	0,43	-0,08	0,95	0,66	0,20	0,03
RAB24	Ras-related protein Rab-24	Ras-Raf-MEK-Erk/JNK signalling#cBIOPORTAL (http://www.cbioportal.org/) in the COAD dataset#cBIOPORTAL (http://www.cbioportal.org/) #RAS gene family#RAB amnd RABL gene family#HGNC	Q969Q5;F8W8H5	N/A	1,04	-0,34	0,31	0,40	0,69	-1,46	0,51	-2,14	0,71	-1,30	0,11	-0,40	0,02	0,03	0,21	-0,34
RAB25	Ras-related protein Rab-25	RAS gene family#RAB amnd RABL gene family#HGNC	P57735;A0A0C4DGX5	N/A	0,13	0,17	0,24	0,28	0,33	-0,36	0,14	-0,17	0,07	0,08	0,19	0,22	0,05	-0,06	0,10	0,13
RAB27A	Ras-related protein Rab-27A	RAS gene family#RAB amnd RABL gene family#HGNC	P51159;H3BN55;H3BVH7;P51159-2;H3BUD9;H3BS49;H3BU81	N/A	0,42	0,15	1,54	0,83	1,26	1,03	0,06	0,05	0,57	-0,07	1,29	0,21	0,78	-0,79	0,72	0,59
RAB27B	Ras-related protein Rab-27B	RAS gene family#RAB amnd RABL gene family#HGNC	O00194;K7ES41	N/A	0,07	0,04	0,10	-0,02	1,16	0,18	0,56	-0,07	0,11	-0,20	0,04	-0,05	0,57	-0,65	0,22	0,60
RAB28	Ras-related protein Rab-28	RAS gene family#RAB amnd RABL gene family#HGNC	P51157;P51157-3;P51157-2	N/A	0,29	0,31	0,08	0,09	0,02	0,03	0,18	0,25	0,09	-0,22	0,57	0,83	0,10	-0,16	0,14	0,28
RAB29	Ras-related protein Rab-7L1	RAS gene family#RAB amnd RABL gene family#HGNC	O14966;O14966-2	N/A	0,54	0,34	0,36	0,14	0,32	-0,12	0,07	0,04	0,26	0,11	0,17	-0,11	0,11	0,07	0,19	0,09
RAB2A	Ras-related protein Rab-2A	RAS gene family#RAB amnd RABL gene family#HGNC	P61019;P61019-2;E9PKL7	N/A	1,19	-0,06	1,82	0,26	0,69	-0,20	1,02	-0,17	0,02	0,00	0,27	0,12	0,35	0,16	0,23	-0,16
RAB2B;DKFZp313C1541	Ras-related protein Rab-2B	RAS gene family#RAB amnd RABL gene family#HGNC	Q8WUD1;Q5HYI5;Q8WUD1-2	N/A	0,18	-0,14	0,99	-0,28	0,72	-0,54	1,65	-0,58	0,98	-0,36	0,68	0,13	0,59	0,27	0,21	-0,14
RAB30	Ras-related protein Rab-30	RAS gene family#RAB amnd RABL gene family#HGNC	Q15771;E9PJQ5;E9PRF7;E9PNB9;E9PS06;H0YDK7;E9PMJ1;Q15771-2	N/A	0,14	-0,45	0,07	0,15	0,01	-0,01	1,35	0,81	0,53	-1,20	0,27	-1,00	0,04	0,35	0,20	0,40
RAB31	Ras-related protein Rab-31	RAS gene family#RAB amnd RABL gene family#HGNC	Q13636	N/A	0,71	-1,17	0,89	-1,91	0,18	-0,89	0,34	-1,23	0,73	-0,43	0,57	-0,80	0,51	-0,55	0,58	0,40
RAB33B	Ras-related protein Rab-33B	RAS gene family#RAB amnd RABL gene family#HGNC	Q9H082	N/A	0,10	-0,11	0,52	0,64	0,16	-0,43	0,22	-0,23	0,19	-0,42	0,74	-0,97	0,70	-1,14	0,35	0,46
RAB35	Ras-related protein Rab-35	RAS gene family#RAB amnd RABL gene family#HGNC	Q15286;F5H157;Q15286-2	N/A	2,15	0,39	0,61	0,29	0,62	0,51	2,72	0,52	0,46	0,22	0,28	-0,16	0,83	-0,43	0,06	-0,04
RAB3A	Ras-related protein Rab-3A	RAS gene family#RAB amnd RABL gene family#HGNC	P20336;M0R257	N/A	0,70	1,78	0,10	0,30	1,45	4,72	1,69	4,34	0,42	1,74	0,10	0,55	0,49	2,04	0,33	-1,49
RAB3B	Ras-related protein Rab-3B	RAS gene family#RAB amnd RABL gene family#HGNC	P20337	N/A	1,61	-1,57	2,03	1,22	1,16	1,04	0,88	0,16	0,50	-0,65	0,60	-0,82	0,17	0,24	0,33	0,43
RAB3D	Ras-related protein Rab-3D	RAS gene family#RAB amnd RABL gene family#HGNC	O95716	N/A	0,44	0,49	0,59	0,40	0,82	0,66	0,75	1,22	0,72	0,50	0,59	0,44	0,32	0,19	0,13	-0,07
RAB43	Ras-related protein Rab-43	RAS gene family#RAB amnd RABL gene family#HGNC	Q86YS6;Q86YS6-2	N/A	1,17	-0,85	0,75	0,43	0,26	1,46	0,56	-0,68	0,28	-0,19	0,15	-0,39	0,30	1,97	0,58	-0,64
RAB4A	Ras-related protein Rab-4A	RAS gene family#RAB amnd RABL gene family#HGNC	P20338	N/A	0,20	0,16	0,36	-0,45	0,04	0,03	0,57	0,43	0,53	0,40	0,30	-0,13	0,88	0,35	0,60	-0,08
RAB4B	Ras-related protein Rab-4B	RAS gene family#RAB amnd RABL gene family#HGNC	P61018;P61018-2;M0R1E0;M0R0X1	N/A	0,39	-0,35	0,20	-0,15	0,72	-0,54	0,59	-0,43	0,09	0,22	0,21	-0,27	0,06	0,14	0,34	0,86
RAB5A	Ras-related protein Rab-5A	RAS gene family#RAB amnd RABL gene family#HGNC	P20339;P20339-2	N/A	1,44	-0,71	0,32	-0,12	1,36	-0,79	1,05	-0,27	0,68	0,35	0,13	0,14	0,11	0,07	0,14	0,08
RAB5B	Ras-related protein Rab-5B	Ras signaling pathway - Homo sapiens (human)#path:hsa04014#KEGG#RAS gene family#RAB amnd RABL gene family#HGNC	P61020;P61020-2;F8VUA5	N/A	0,90	0,22	1,52	0,48	0,95	-0,87	0,24	-0,12	0,07	-0,03	0,67	-0,45	0,96	-0,41	0,65	-0,23
RAB5C	Ras-related protein Rab-5C	RAS gene family#RAB amnd RABL gene family#HGNC	P51148;P51148-2;K7ERI8;K7ERQ8;F8VVK3;K7ENY4	N/A	1,17	-0,40	0,59	-0,17	0,80	0,15	0,91	-0,41	0,09	-0,05	0,29	-0,09	0,12	-0,03	0,42	-0,11
RAB6A	Ras-related protein Rab-6A	RAS gene family#RAB amnd RABL gene family#HGNC	P20340-2;P20340-4	N/A	0,84	-0,37	0,68	0,29	0,79	-0,36	1,03	-0,47	0,11	-0,05	0,32	0,13	0,17	0,07	0,04	-0,02
RAB6B	Ras-related protein Rab-6B	RAS gene family#RAB amnd RABL gene family#HGNC	Q9NRW1;Q9NRW1-2;J3KR73	N/A	1,31	-0,40	1,26	0,63	1,13	-0,92	0,28	-0,50	0,01	0,01	1,04	0,75	0,06	0,07	0,16	0,12
RAB7A	Ras-related protein Rab-7a	RAS gene family#RAB amnd RABL gene family#HGNC	P51149;C9J592;C9J8S3;C9J4V0;C9IZZ0	N/A	0,16	-0,05	0,92	0,23	0,14	-0,10	0,12	-0,03	0,52	-0,06	0,15	-0,03	0,62	-0,42	0,96	0,10
RAB8B	Ras-related protein Rab-8B	RAS gene family#RAB amnd RABL gene family#HGNC	Q92930;H0YNE9;H0YMN7	N/A	0,36	-0,04	2,51	-0,32	0,20	0,14	0,08	-0,07	0,95	-0,65	1,45	-1,06	0,06	0,21	0,01	0,01
RAB9A	Ras-related protein Rab-9A	RAS gene family#RAB amnd RABL gene family#HGNC	P51151	N/A	0,88	0,94	0,82	0,86	0,68	0,71	0,98	1,11	0,30	-0,44	0,86	-0,36	0,66	-0,28	0,32	0,26
RABGEF1	Rab5 GDP/GTP exchange factor	Regulation of Ras family activation#ras_pathway#PID	Q9UJ41;E9PFK9;Q9UJ41-2;Q9UJ41-4;Q9UJ41-3;Q3HKR1;A0A0A0MSJ3	N/A	0,21	-0,19	0,03	-0,03	0,53	-0,85	0,06	0,06	0,37	0,67	0,27	-0,48	0,39	0,77	0,32	-0,75
RABL2B;RABL2A	Rab-like protein 2B;Rab-like protein 2A	RAS gene family#RAB amnd RABL gene family#HGNC	Q9UNT1;Q9UBK7;Q9UBK7-2;Q9UNT1-2;Q9UNT1-3;Q9UBK7-3	N/A	0,66	2,48	0,28	-1,10	0,67	2,53	0,21	1,01	0,96	0,40	0,22	0,10	0,12	-0,50	0,00	0,00
RABL3	Rab-like protein 3	RAS gene family#RAB amnd RABL gene family#HGNC	Q5HYI8;C9JXM3	N/A	0,98	-0,59	0,03	-0,07	0,71	-0,63	1,39	-1,14	0,03	-0,04	0,17	-0,26	0,33	-0,93	0,57	0,70
RABL6	Rab-like protein 6	RAS gene family#RAB amnd RABL gene family#HGNC	Q3YEC7;Q3YEC7-2;F2Z2T0	N/A	0,11	-0,05	0,86	-0,31	0,84	-0,34	0,29	-0,10	0,01	0,01	0,24	0,20	0,12	-0,15	0,04	-0,04
RAC1	Ras-related C3 botulinum toxin substrate 1	Ras signaling pathway - Homo sapiens (human)#path:hsa04014#KEGG#ras-independent pathway in nk cell-mediated cytotoxicity#nkcellspathway#BioCarta#ras signaling pathway#raspathway#BioCarta#influence of ras and rho proteins on g1 to s transition#raccycdpathway#BioCarta	P63000-2;P63000	N/A	0,18	0,16	0,45	0,41	0,18	-0,21	0,03	0,04	2,71	-1,00	2,70	-1,03	0,30	-0,37	0,36	0,53
RAF1	RAF proto-oncogene serine/threonine-protein kinase	Ras signaling pathway - Homo sapiens (human)#path:hsa04014#KEGG#ras signaling pathway#raspathway#BioCarta#CREB phosphorylation through the activation of Ras#R-HSA-442742#Reactome#Ras signaling in the CD4+ TCR pathway#tcrraspathway#PID#Signalling to RAS#R-HSA-167044#Reactome#influence of ras and rho proteins on g1 to s transition#raccycdpathway#BioCarta#Ras-Raf-MEK-Erk/JNK signalling#cBIOPORTAL (http://www.cbioportal.org/) in the COAD dataset#cBIOPORTAL (http://www.cbioportal.org/)	P04049;P04049-2;H7C155	N/A	0,10	-0,07	0,36	0,17	0,45	0,21	0,07	-0,05	0,01	0,00	0,19	-0,07	0,71	-0,37	0,51	-0,17
RALA	Ras-related protein Ral-A	Ras signaling pathway - Homo sapiens (human)#path:hsa04014#KEGG#ras signaling pathway#raspathway#BioCarta#Signalling to RAS#R-HSA-167044#Reactome	P11233;H7C3P7;C9JPE8	N/A	0,47	-0,11	0,10	0,10	0,25	0,17	0,42	-0,14	0,87	0,68	0,56	0,59	0,78	-0,94	0,76	-0,55
RALB	Ras-related protein Ral-B	Ras signaling pathway - Homo sapiens (human)#path:hsa04014#KEGG#Signalling to RAS#R-HSA-167044#Reactome	P11234;P11234-2;P11234-3;C9J6B1;C9JQB3	N/A	0,07	-0,05	0,27	0,18	0,13	-0,09	0,35	0,23	0,16	-0,09	0,48	0,25	0,01	0,01	0,17	0,12
RALBP1	RalA-binding protein 1	Ras signaling pathway - Homo sapiens (human)#path:hsa04014#KEGG#ras signaling pathway#raspathway#BioCarta	Q15311	N/A	0,01	0,00	1,08	-1,03	0,04	0,02	0,55	-0,13	0,05	-0,05	0,62	-0,52	0,16	-0,27	0,59	0,71
RAP1B	Ras-related protein Rap-1b;Ras-related protein Rap-1b-like pro	Ras signaling pathway - Homo sapiens (human)#path:hsa04014#KEGG#Signalling to RAS#R-HSA-167044#Reactome	P61224;P61224-3;A6NIZ1;F5H6R7;F5H004;F5GX62;E7ESV4;F5H7Y6;F5GYB5;P61224-2;P61224-4;F5H0B7;F5H500;F5H823;B7ZB78	N/A	1,09	0,51	0,59	0,25	0,63	0,26	0,73	0,31	0,08	0,04	0,70	-0,15	0,73	-0,19	0,24	-0,05
RAPGEF2	Rap guanine nucleotide exchange factor 2	Signalling to RAS#R-HSA-167044#Reactome	Q9Y4G8	N/A	0,02	-0,05	0,09	-0,17	0,13	0,33	1,19	-0,99	0,52	-2,00	0,63	-2,36	0,32	-1,30	0,33	1,47
RAPH1	Ras-associated and pleckstrin homology domains-containing p	Signalling to RAS#R-HSA-167044#Reactome	Q70E73;C9J164;C9K0J5;Q70E73-5;Q70E73-2;Q70E73-8;Q70E73-7;Q70E73-4;Q70E73-3;Q70E73-9;Q70E73-6	N/A	0,83	-0,59	0,61	0,35	0,01	-0,01	0,14	0,08	0,23	-0,25	0,06	0,04	0,75	-0,39	0,63	1,05
RASA1	Ras GTPase-activating protein 1	Ras signaling pathway - Homo sapiens (human)#path:hsa04014#KEGG#Signalling to RAS#R-HSA-167044#Reactome#Regulation of RAS by GAPs#R-HSA-5658442#Reactome#Regulation of Ras family activation#ras_pathway#PID	P20936;E9PGC0;P20936-2;P20936-4	N/A	0,45	0,38	0,07	0,07	0,37	-0,31	0,99	0,13	0,17	-0,11	0,30	0,35	0,24	0,34	0,12	-0,08
RASA2	Ras GTPase-activating protein 2	Ras signaling pathway - Homo sapiens (human)#path:hsa04014#KEGG#Signalling to RAS#R-HSA-167044#Reactome#Regulation of RAS by GAPs#R-HSA-5658442#Reactome#Regulation of Ras family activation#ras_pathway#PID	Q15283;A0A0A0MSJ9;Q15283-2	N/A	0,01	0,01	0,32	-2,32	0,03	-0,03	0,15	0,12	0,95	-0,70	0,12	0,45	0,90	-1,23	1,64	-1,20
RASA3	Ras GTPase-activating protein 3	Ras signaling pathway - Homo sapiens (human)#path:hsa04014#KEGG#Signalling to RAS#R-HSA-167044#Reactome#Regulation of RAS by GAPs#R-HSA-5658442#Reactome	Q14644;Q14644-2	N/A	0,26	-0,76	0,27	-0,56	0,14	-0,36	0,03	0,12	0,34	0,38	0,75	0,66	0,67	-0,59	0,49	0,43
RASAL1	RasGAP-activating-like protein 1	Ras signaling pathway - Homo sapiens (human)#path:hsa04014#KEGG#Signalling to RAS#R-HSA-167044#Reactome#Regulation of RAS by GAPs#R-HSA-5658442#Reactome#Regulation of Ras family activation#ras_pathway#PID	O95294-4;O95294-3;O95294;O95294-2	N/A	0,00	-0,01	0,49	-0,89	1,19	-1,63	0,25	-0,57	0,07	0,04	0,11	0,23	1,36	0,63	1,85	-0,67
RASAL2	Ras GTPase-activating protein nGAP	Ras signaling pathway - Homo sapiens (human)#path:hsa04014#KEGG#Signalling to RAS#R-HSA-167044#Reactome#Regulation of RAS by GAPs#R-HSA-5658442#Reactome#Regulation of Ras family activation#ras_pathway#PID	Q9UJF2-2;Q9UJF2;H0Y638	N/A	1,85	-0,81	2,21	-0,30	0,88	-0,51	0,45	-0,57	0,81	0,49	0,21	0,18	0,37	0,24	1,11	0,77
RASGRP2	RAS guanyl-releasing protein 2	Ras signaling pathway - Homo sapiens (human)#path:hsa04014#KEGG#Regulation of Ras family activation#ras_pathway#PID	Q7LDG7-2;Q7LDG7;Q7LDG7-4	N/A	0,18	-0,63	0,16	-1,04	0,11	-0,47	0,23	0,79	0,47	1,02	0,40	0,86	0,68	1,49	0,29	-0,65
RB1	Retinoblastoma-associated protein	influence of ras and rho proteins on g1 to s transition#raccycdpathway#BioCarta	P06400	N/A	1,37	0,43	0,83	-0,24	2,12	1,76	1,52	0,50	0,32	-0,21	0,17	-0,09	0,51	0,28	0,33	-0,27
REL	Proto-oncogene c-Rel	Ras signaling pathway - Homo sapiens (human)#path:hsa04014#KEGG	Q04864;Q04864-2	N/A	0,31	-0,68	0,37	-0,60	0,25	-0,52	1,13	-1,04	0,20	-0,47	0,30	-0,55	0,09	0,18	0,11	0,21
RELA	Transcription factor p65	Ras signaling pathway - Homo sapiens (human)#path:hsa04014#KEGG#influence of ras and rho proteins on g1 to s transition#raccycdpathway#BioCarta	Q04206;Q2TAM5;A0A087X0W8;Q04206-3;Q04206-4;Q04206-2;E9PKH5;E9PKV4;A0A087WVP0	N/A	0,70	-0,08	0,87	-0,24	0,32	0,07	0,88	-0,11	0,34	0,30	0,60	0,45	0,09	0,07	0,17	0,14
RGL2	Ral guanine nucleotide dissociation stimulator-like 2	Ras signaling pathway - Homo sapiens (human)#path:hsa04014#KEGG	O15211;O15211-2	N/A	0,00	0,00	0,51	-0,11	0,75	-0,52	1,19	-0,51	0,70	1,00	0,32	-0,45	0,28	-1,00	0,10	-0,16
RHOA	Transforming protein RhoA	Ras signaling pathway - Homo sapiens (human)#path:hsa04014#KEGG#ras signaling pathway#raspathway#BioCarta#influence of ras and rho proteins on g1 to s transition#raccycdpathway#BioCarta	P61586;C9JX21	N/A	0,11	-0,13	0,08	0,09	0,69	-0,65	0,59	-0,57	0,58	-0,37	0,49	-0,49	1,51	-0,69	0,81	0,44
RIN1	Ras and Rab interactor 1	Ras signaling pathway - Homo sapiens (human)#path:hsa04014#KEGG#Regulation of Ras family activation#ras_pathway#PID	Q13671;Q13671-2;E9PNR2	N/A	0,15	-0,07	0,14	-0,30	0,03	-0,19	1,29	-0,93	0,95	0,59	0,97	0,64	0,36	0,31	0,12	-0,08
RPS6KA1	Ribosomal protein S6 kinase alpha-1;Ribosomal protein S6 kin	CREB phosphorylation through the activation of Ras#R-HSA-442742#Reactome	Q15418;Q15418-2;E9PGT3;Q15418-4;Q15418-3	N/A	0,42	0,12	0,74	-0,22	0,67	-0,20	0,25	-0,11	0,37	-0,32	0,37	-0,20	0,57	-0,27	0,43	0,26
RPS6KA3	Ribosomal protein S6 kinase alpha-3	CREB phosphorylation through the activation of Ras#R-HSA-442742#Reactome	P51812	N/A	0,43	0,22	0,15	-0,05	0,63	0,21	0,81	0,26	0,70	-0,19	0,77	-0,31	0,62	-0,20	0,97	0,23
RRAS	Ras-related protein R-Ras	Ras signaling pathway - Homo sapiens (human)#path:hsa04014#KEGG#CREB phosphorylation through the activation of Ras#R-HSA-442742#Reactome#Regulation of Ras family activation#ras_pathway#PID	P10301	N/A	1,19	-0,15	0,08	0,07	1,77	-0,94	0,84	-0,46	0,37	-0,09	0,72	0,20	1,16	-0,32	0,31	0,17
RRAS2	Ras-related protein R-Ras2	Ras signaling pathway - Homo sapiens (human)#path:hsa04014#KEGG	P62070;P62070-4;A0A0C4DGH2;E9PK85;P62070-3;P62070-2	N/A	0,43	-0,21	0,48	-0,24	0,02	-0,01	0,61	-0,29	0,01	-0,01	0,27	-0,22	0,43	0,34	0,12	-0,10
SHC1	SHC-transforming protein 1	Ras signaling pathway - Homo sapiens (human)#path:hsa04014#KEGG#Signalling to RAS#R-HSA-167044#Reactome	P29353-7;P29353-6;P29353;P29353-2	N/A	1,08	-0,26	0,37	-0,24	0,47	-0,21	1,23	-0,33	0,24	-0,09	0,14	-0,06	1,42	0,39	0,15	-0,06
SOS1	Son of sevenless homolog 1	Ras signaling pathway - Homo sapiens (human)#path:hsa04014#KEGG#Regulation of Ras family activation#ras_pathway#PID	Q07889;G5E9C8	N/A	0,73	-0,28	0,02	-0,02	0,33	-0,20	1,08	-0,46	1,05	0,32	0,16	0,13	0,20	0,06	0,71	0,48
SPTAN1	Spectrin alpha chain, non-erythrocytic 1	Signalling to RAS#R-HSA-167044#Reactome	Q13813;Q13813-2;A0A0D9SGF6;Q13813-3;A0A0D9SF54	N/A	0,84	-0,09	0,51	-0,09	0,80	-0,10	0,22	0,02	0,57	-0,25	0,44	-0,21	0,73	-0,32	0,54	0,23
SPTB	Spectrin beta chain, erythrocytic	Signalling to RAS#R-HSA-167044#Reactome	P11277-2;P11277;P11277-3	N/A	0,61	0,95	0,22	-0,38	0,30	-0,74	0,12	-0,24	1,66	0,25	0,07	0,03	0,50	0,43	0,14	-0,05
SPTBN1	Spectrin beta chain, non-erythrocytic 1	Signalling to RAS#R-HSA-167044#Reactome	Q01082;A0A087WUZ3;Q01082-2	N/A	0,54	-0,06	0,85	-0,08	0,11	0,02	0,53	0,04	0,86	-0,29	0,89	-0,33	1,04	-0,38	0,80	0,29
SPTBN2	Spectrin beta chain, non-erythrocytic 2	Signalling to RAS#R-HSA-167044#Reactome	O15020;O15020-2	N/A	1,32	0,59	1,40	-0,79	0,04	0,02	0,28	0,10	0,67	0,36	0,33	0,13	0,05	0,04	0,01	0,01
SRC	Proto-oncogene tyrosine-protein kinase Src	Signalling to RAS#R-HSA-167044#Reactome	P12931;P12931-2	N/A	0,16	0,12	0,88	-0,86	0,35	-0,19	1,52	0,42	0,33	0,31	0,11	0,10	0,39	0,34	0,03	-0,04
STK4	Serine/threonine-protein kinase 4;Serine/threonine-protein kina	Ras signaling pathway - Homo sapiens (human)#path:hsa04014#KEGG	Q13043;Q13043-2;F5H5B4	N/A	1,76	-1,10	0,11	-0,05	0,36	-0,40	1,94	-1,14	0,16	0,04	1,31	0,42	0,48	0,34	0,23	0,44
SYK	Tyrosine-protein kinase SYK	ras-independent pathway in nk cell-mediated cytotoxicity#nkcellspathway#BioCarta	P43405;P43405-2	N/A	1,58	0,29	1,81	-0,58	0,62	0,40	0,43	0,21	0,47	-0,43	0,38	-0,32	0,12	-0,22	0,36	0,67
TBK1	Serine/threonine-protein kinase TBK1	Ras signaling pathway - Homo sapiens (human)#path:hsa04014#KEGG	Q9UHD2	N/A	0,21	-0,19	1,14	1,14	1,11	1,18	0,79	1,03	0,26	0,47	0,34	0,46	0,13	0,22	0,06	-0,11
TLN1	Talin-1	Signalling to RAS#R-HSA-167044#Reactome	Q9Y490	N/A	1,64	-0,86	0,43	-0,08	1,21	-0,22	1,13	-0,17	0,46	0,16	0,19	0,05	0,79	-0,10	1,19	-0,26
UBB;RPS27A;UBA52;UBC;UBE	Polyubiquitin-B;Ubiquitin;Ubiquitin-40S ribosomal protein S27a	Signalling to RAS#R-HSA-167044#Reactome#Regulation of RAS by GAPs#R-HSA-5658442#Reactome	P0CG47;P62979;P62987;J3QS39;J3QTR3;F5H6Q2;F5GYU3;F5H2Z3;F5H265;B4DV12;F5H388;F5H747;F5GXK7;J3QKN0;Q5PY61;Q96C32;P0CG48;M0R1V7;M0R1M6;M0R2S1;J3QLP7;J3QRK5;F5GZ39	N/A	0,92	-0,27	0,48	-0,20	0,33	-0,16	0,99	-0,37	0,07	0,04	0,02	-0,01	0,04	0,02	0,25	-0,14
VCL	Vinculin	Signalling to RAS#R-HSA-167044#Reactome	P18206;P18206-2;Q5JQ13	N/A	0,57	-0,16	0,06	0,01	0,79	-0,20	0,46	-0,09	0,08	-0,05	0,60	-0,27	0,16	-0,08	0,16	0,08
YWHAB	14-3-3 protein beta/alpha;14-3-3 protein beta/alpha, N-terminal	Signalling to RAS#R-HSA-167044#Reactome	P31946;P31946-2	N/A	0,39	-0,08	0,71	-0,28	0,19	0,04	0,84	-0,18	0,17	0,10	0,11	0,05	0,03	-0,02	0,41	0,19

Table S9. Alterations in Ras pathway protein expression levels by stable knockdown of KRAS, FOXO3, NCOA3, and TCF7L2. Lysates from stable shRNA knockdown cell lines in DLD-1 cells with or without mutant KRAS were subjected to global proteomics analysis. All proteins that were quantified with LC-MS and associated with the RAS pathway were subjected to a two sample two-tailed t-test corrected with permutation FDR against a control. The Pathway column is divided into 3 parts, pathway name, id and source, with the "#" as a delimiter. Proteins associated with several pathways have them given after each other also with "#" as the delimiter. Fold changes in the expression as compared to the shControl in respective genetic backgrounds are shown with significant changes (FDR ≤0.05) indicated.

Table S10: *In silico* analysis of GLUT1 (SLC2A1) promoter, 3Kb upstream of its initiation site, for finding direct bindind sites of TCF7L2, FOXO3 and NCOA3.

Gene	No. of sites	Median Score
TCF7L2	16	5,34
FOXO3	13	5,36
NCOA3	N/A	N/A
HIF1A	38	5,27

Table S10. *In silico* analyseswas performed by JASPER (http://jasper.genereg.net) to find out if binding sites for FOXO3, NCOA3 and TCF7L2 exist in the GLUT1 promoter (3kb upstream sequence of the initiation site of the GLUT1 gene). These analyses revealed 16 and 13 putative binding sites for TCF7L2 and FOXO3, respectively, with a median score of 5.34 and 5.36, respectively. HIF1A, a positive regulator of GLUT1 (SLC2A1), has 38 binding sites with a median score of 5.27 similar to TCF7L2 and FOXO3 genes.
